# Integrative Computational Framework for Understanding Metabolic Modulation in Leishmania

**DOI:** 10.3389/fbioe.2019.00336

**Published:** 2019-11-19

**Authors:** Nutan Chauhan, Shailza Singh

**Affiliations:** National Centre for Cell Science, Pune, India

**Keywords:** metabolic network, Forman curvature, Forman-Ricci curvature, network topology, Leishmania, flux balance analysis

## Abstract

**Background:** The integration of computational and mathematical approaches is used to provide a key insight into the biological systems. Through systems biology approaches we seek to find detailed and more robust information on *Leishmanial* metabolic network. Forman/Forman-Ricci curvature measures were applied to identify important nodes in the network(s). This was followed by flux balance analysis (FBA) to decipher important drug targets.

**Results:** Our results revealed several key high curvature nodes (metabolites) belonging to common yet crucial metabolic networks, thus, maintaining the integrity of the network which signifies its robustness. Further analysis revealed the presence of some of these metabolites, MGO, in redox metabolism of the parasite. Being a component in the glyoxalase pathway and highly cytotoxic, we further attempted to study the outcome of the deletion of the key enzyme (GLOI) mainly involved in the neutralization of MGO by utilizing FBA. The model and the objective function kept as simple as possible demonstrated an interesting emergent behavior. The non-functional GLOI in the model contributed to “zero” flux which signifies the key role of GLOI as a rate limiting enzyme. This has led to several fold increase production of MGO, thereby, causing an increased level of MGO^•−^ generation.

**Conclusions:** The integrated computational approaches have deciphered GLOI as a potential target both from curvature measures as well as FBA which could further be explored for kinetic modeling by implying various redox-dependent constraints on the model. Furthermore, a constraint-based FBA on a larger model could further be explored to get broader picture to understand the exact underlying mechanisms. Designing various *in vitro* experimental perspectives could churn the therapeutic importance of GLOI.

## Introduction

Metabolic networks are becoming of much interest amongst the researchers due to their unique feature of connecting every node (the metabolites) with the links (the reactions) that are catalyzed by specific gene products responsible for growth and maintenance of a cell (Mahadevan and Palson, 2005). Metabolic networks can be used to shed light on finding various disease mechanisms via the identification of essential genes by implying perturbations on networks, thereby, characterizing the structure-function relationships (Watts and Strogatz, 1998; Barabási and Oltvai, 2004; Dorogovtsev and Mendes, 2013). Structure-function relationship of a network in terms of network *robustness* and reliability is strongly linked to its geometric and topological properties and can be studied by using graph theory (Barabási and Oltvai, 2004). Recent technological advances in graph theory have allowed us to analyze and describe highly complex systems at smaller and more detailed scales and have been utilized to study global impact of long term sickle cell disease on brain (Case et al., 2018), to diagnose pre-symptomatic Alzheimer's disease (Bytautiene, 2013), to understand the human brain network (Vecchio et al., 2017). Recently, the development of geometry based measures is significantly attracting the focus of researchers to characterize the structural aspect of complex networks.

### Curvature-Based Methods for Complex Networks

In geometry based measures of complex networks, *curvature* is one of the notions that are being explored to understand the complexity of graphs. Curvature plays central role in Riemannian geometry since it represents a measure to quantify the deviation of a geometrical object from being flat (Tannenbaum et al., 2016). Curvature measures can be used to quantify the robustness and thereby the functionality of networks.

Among several types of curvature notions, *Ricci curvature* is known to be the most useful for analyzing the complex networks (Burago et al., 2001; Saucan and Appleboim, 2005; Ollivier, 2009, 2010, 2011; Ni et al., 2015; Sandhu et al., 2015; Sreejith et al., 2016). Ricci curvature measures deviance of geodesics (shortest path) relative to Euclidean shortest-paths and is related to mass transport or entropy (Wasserstein metrics) (Evans, 2001; Villani, 2003). High Ricci curvature is typically found near network hubs, where an anchor node (high curvature node) is connected to many existing nodes in close proximity. Principally, curvature is very close to robustness; therefore, removal of high curvature node/edge will result in collapse of network.

*Ollivier-Ricci* (Ollivier, 2009, 2011) and *Forman-Ricci* (Forman, 2003), two different discretization of Ricci curvature, give efficient solutions for network geometrizations. In case of undirected networks, Ollivier-Ricci curvature has proved its applicability in various network analyses (Loisel and Romon, 2014; Ni et al., 2015; Sandhu et al., 2015; Gao et al., 2016). On the other hand, Forman-Ricci curvature has been introduced as a tool for undirected (Sreejith et al., 2016, 2017a,b) as well as directed (Weber et al., 2017a,b) network analyses. Irrespective of their applicability and handling the large networks, the two curvature notions demonstrated high correlation (Samal et al., 2018). However, Forman-Ricci curvature is a faster computation method and can be utilized in larger real-networks. Current study exploits the application of Forman-Ricci curvature and systems biology to capture the behavior of Leishmanial metabolic network.

### Leishmaniasis and Systems-Biology

Neglected infectious diseases have affected at least a billion of human populations worldwide (Narain et al., 2010; WHO, 2011) and are of primary concern due to the lack of effective and affordable drug regimens. Among them, cutaneous leishmaniasis (CL), a very common clinical form of leishmaniasis, has always been overlooked as a major public health problem due to its non-fatality. The causative agent of CL, a protozoan parasite, *Leishmania major* has a digenetic lifecycle and lives in two hosts, sandfly, *Phlebotomus argentipes*, and human, in the form of flagellated promastigotes (procyclic phase) and non-flagellated amastigotes (metacyclic phase), respectively.

**Figure 1:**
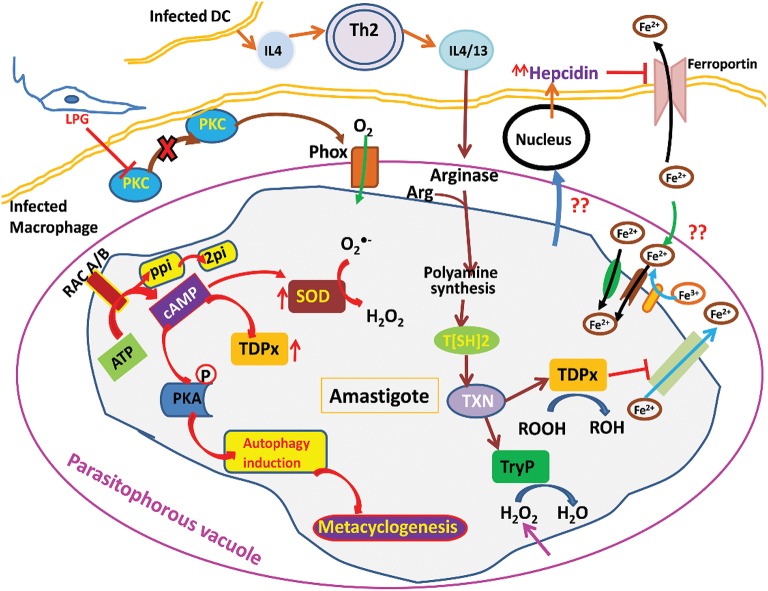
Illustration of *Leishmania* parasite survival from oxidative stress.

When inside the human host, the parasite has to undergo the oxidative stress generated by host macrophages (Figure 1). This stress is also attained from free radical generation within the parasite in many ways mainly via electron transport chain. Moreover, there are several highly reactive metabolites such as glyoxals that contribute to the generation of free radicals. These metabolites can react with the other metabolites and enzymes and not only cause inactivation of enzymes but also the formation of free radicals. To deal with these types of internal and external trauma the parasite has evolved with its unique redox machinery that helps in neutralization of free radicals. The main component of this antioxidant defense mechanism are trypanothione synthase (TryS), trypanothione reductase (TryR), tryparedoxin peroxidase (TryP), tryparedoxin-dependent peroxidase (TDPx), and tryparedoxin (TXN). The center of these components is a central reductant, T[SH]_2_ that regulates the activation of these enzymes (Krauth-Siegel and Inhoff, 2003).

Due to the incapability and increasing drug resistance of the current therapy regime techniques, the necessity to develop novel, more efficient and affordable anti-leishmanial drugs along with potential molecular drug targets with therapeutic value are needed. The difference between *Leishmania* and human host redox metabolism attracts researcher's interest to explore this mechanism for discovering novel drug targets and design potential inhibitors against the redox enzymes without crossing the host machinery (Ascenzi et al., 2003).

To study the role and effect of other intermediates in the redox network, systems biology approaches are nowadays being used (Kabra et al., 2018). Systems biology approaches have already been utilized to study systems dynamics of complex redox metabolic pathways in many organisms (Hädicke et al., 2011; Pillay et al., 2013; Wang et al., 2018). Before implementing many available network analyses techniques like, Genome scale metabolic modeling (GEMs) (Hädicke et al., 2011; Pillay et al., 2013), constraint based methods, kinetic pathway modeling (Ho, 2008; Resat et al., 2009; Kumar et al., 2019), the knowledge and availability of all possible components and their inter-connections is a pre-requisite. Recently, kinetic modeling and constraint-based metabolic modeling have gained researcher's focus. In case of kinetic modeling, the availability of kinetic data and rate laws is required. However, due to the lack of sufficient kinetic data the use of metabolic kinetic modeling on larger scale networks is limited. On the other hand, constraint-based analysis such as *flux-based analysis (FBA)* requires only the knowledge of stoichiometry of the metabolites and can be easily applied to even larger scale metabolic networks. FBA calculations rely on the assumption of steady-state growth and mass balance and can be used to predict the flux of a metabolite flowing through a metabolic network. To predict the growth rate or to find a specific cellular function, FBA tries to find possible solutions to optimize the stated objective function(s) and gives quantitative insights into the genotype-phenotype relationship of the model. Although, FBA is incapable of predicting the concentrations of metabolites and does not account for any regulatory effects like activation/inactivation of enzymes or gene-expression regulations, there are extensive embodiment of literature showing its applications in many fields including drug target identification (Chavali et al., 2008; Fatumo et al., 2009; O'Brien et al., 2015; Sharma et al., 2017; Subramanian and Sarkar, 2017; Tewari et al., 2017).

**Figure 2:**
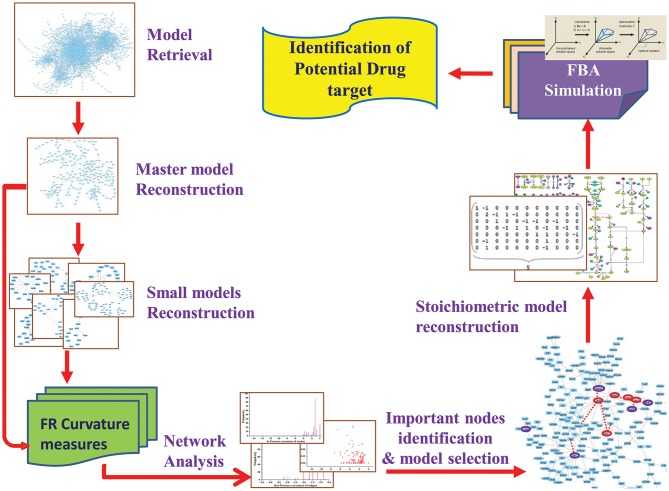
Overview of the workflow strategy adopted.

In current work, we attempted to explore the application of Forman and Forman-Ricci curvature on metabolic network of Leishmania. Using curvature measures, we tried to identify important metabolic pathways contributing to the overall network robustness by recognizing “important” metabolic hubs in the network. Through this, we seek to spot metabolites and enzymes having essential role in the survival of the parasite. Further, to predict the effect of the absence of these “important” metabolites, constraint-based FBA is used to uncover the importance of enzymes and their probable role as drug target for therapeutic significance (Figure 2). Also, our systems pharmacology modeling on the kinetic model of the selected drug target using known inhibitors may provide significant insight for further clinical interventions.

## Materials and Methodology

### Data Sets and Model Reconstruction

Initially, *Leishmania major* metabolic network **iAC560** (Chavali et al., 2008) was retrieved from BioModels Database and imported to Cell Designer (4.4) (Funahashi et al., 2003). The model consisted of 1112 reactions and 1147 metabolites. This model was used to construct a bipartite directed graph that considered as master model. The master model was further used for constructing small scale models (Figure 2).

### Generation of Connectivity Matrix

Connectivity matrices for master and other models were generated by considering the connections between metabolites (substrate to product). A value of “1” was assigned for connection, if a metabolite (substrate) is connected (producing or converting into) to other metabolite (product), and “0” for no connection.

### Network Analysis

#### Network Topology

The reconstructed bipartite directed graphs were visualized in Cytoscape (3.6.0) (Shannon et al., 2003) and their topological information (network properties) of nodes and edges were obtained using graph theory followed by calculation of various network properties. We used Forman curvature and Forman-Ricci curvature for the analysis of our bipartite directed graphs. For any given directed bipartite graph we calculated (Samal et al., 2018):

(1) $$\begin{array}{l} F\left(e\right)=w_{e}\;\left(\frac{w_{v_{1}}}{w_{e}}-\;\sum_{e_{v1\;}\sim \;e}^{}\frac{w_{v1}}{\sqrt{w_{e}w_{e_{v1}}}}\right)\\ \;+\;w_{e}\;\left(\frac{w_{v_{2}}}{w_{e}}-\;\sum_{e_{v2\;}\sim \;e}^{}\frac{w_{v2}}{\sqrt{w_{e}w_{e_{v2}}}}\right) \end{array}$$

Where, *F* is the Forman curvature of the directed edge $e=\;\vec{v_{1}v_{2}}$ that originates from node *v*_1_and terminates at node *v*_2_. Only those directed edges were considered for calculation that either terminate at node *v*_1_ or originate at node *v*_2_. *w*_*e*_ denotes the weight of the edge *e* under consideration. *w*_*v*1_ and *w*_*v*2_ denote the weights associated with the nodes *v*_1_ and *v*_2_, respectively. *e*_*v*1_ ~ *e* and *e*_*v*2_ ~ *e* denote the set of edges incident on nodes *v*_1_ and *v*_2_, respectively, after excluding the edge *e* under consideration which connects the two nodes *v*_1_ and *v*_2_. Furthermore, self-loops or self-edges on nodes *v*_1_ and *v*_2_, edges facing opposite direction, were completely ignored.

#### Forman Curvature on Networks

To distinguish between incoming, *E*_*I,v*_, and outgoing, *E*_*O,v*_, edges for a given node *v*, unnormalized Forman curvature (the sum of the curvature of all edges incident or outgoing on that node), *In Forman curvature* ***F***_***I*(*v*)**_ and *Out Forman curvature* ***F***_***O*(*v*)**_ were computed as follows:

(2) $$\begin{array}{r} F_{I}\left(v\right)=\;\underset{e\in E_{I,v}}{\sum }F\left(e\right) \end{array}$$

(3) $$\begin{array}{r} F_{O}\left(v\right)=\underset{e\in E_{O,v}}{\sum }F\left(e\right) \end{array}$$

And, the total flow on a given node was obtained as:

(4) $$\begin{array}{r} F_{T}\left(v\right)=\;F_{I}\left(v\right)-\;F_{O}\left(v\right) \end{array}$$

#### Forman-Ricci Curvature on Networks

Moreover, for determining Forman-Ricci curvature, *FR(v)* (normalized Forman curvature) (Sreejith et al., 2017a,b) of a node *v*, the sum of the curvature of all edges incident or outgoing on that node was divided by the degree of that node:

(5) $$\begin{array}{r} FR_{I}\left(v\right)=\;\frac{1}{deg\left(v\right)}\;\underset{e_{v}\;\sim \;v}{\sum }F\left(e_{v}\right) \end{array}$$

(6) $$\begin{array}{r} FR_{O}\left(v\right)=\;\frac{1}{deg\left(v\right)}\;\underset{e_{v}\;\sim \;v}{\sum }F\left(e_{v}\right) \end{array}$$

Where, *FR*_*I*(*v*)_ and *FR*_*O*(*v*)_ are *In* and *out Forman-Ricci curvature*, respectively, *deg(v)* is *in* and *out-degree* and *F(e*_*v*_*)* is the Forman curvature of edge *e*_*v*_, and *e*_*v*_ ~ *v* represents the set of edges incoming or outgoing on that node *v*. Subsequently, total flow on node *v* was obtained as follows:

(7) $$\begin{array}{r} FR_{T}\left(v\right)=\;FR_{I}\left(v\right)-\;FR_{O}\left(v\right) \end{array}$$

#### Flux Balance Analysis

##### Reconstruction of the stoichiometric model

For the reconstruction of stoichiometric model COBRA Toolbox (Vlassis et al., 2014; Heirendt et al., 2018) was used. Necessary exchange reactions and transport reactions for uptake and excretion of typical substrates and products, respectively, were introduced. The network was devoid of any organism specific internal compartments and only external and internal environment were taken into account. Stoichiometric model is represented in the form of stoichiometric matrix, S, that is a mathematical representation of the network. In this matrix, each reaction is a column and each metabolite is a row. And the participation of the metabolites in the corresponding reactions is denoted by their stoichiometric coefficients where “−1” is used for the consumption and “+1” for the production of the metabolite. In our model, the upper and lower bounds of reversible reactions were set between +1,000 and −1,000, and for irreversible reaction between 0 and +1,000. Uptake and excretion reactions were also set between −1,000 and +1,000. The upper and lower bounds for any reaction define the total space allowed for flux distribution (Orth et al., 2010).

##### FBA simulation

Once the model is reconstructed, constraint-based FBA simulation was performed in COBRA Toolbox in MATLAB (Becker et al., 2007). FBA assumes steady-state kinetics and uses linear programming (LP) based optimization to determine the flux distribution to solve a given metabolic objective function by maximizing or minimizing it (Orth et al., 2010). Hence, the LP problem was formulated as,

Maximize Z,

Subject to

$$\begin{array}{l} \;S•\text{v}=0\\ v_{min}\leq v\leq v_{max} \end{array}$$

Where, Z is the objective function to be maximized (or minimized), S is the stoichiometric matrix of *m* x *n, v* represents the flux vector that is controlled through enzyme capacity constraints *v*_*min*_ and *v*_*max*_ representing lower and upper bounds, respectively. After conversion into mathematical form, the simulation was performed to maximize the objective function. Initially the objective function was carrying zero flux indicating metabolic gaps in the model. The required demand reactions were added to the network to refine the model till the non-zero value of the objective function is attained.

### Systems Pharmacology Modeling

The reactions for the model were input in COPASI v4.19.140 (Hoops et al., 2006) followed by assignment of appropriate kinetic laws. All the reactions were considered as irreversible and the reversible reactions were broken into two reactions for maintaining uniformity in the kinetic model (Table S1). The concentrations, time units and reaction fluxes in the model are in molar (M), seconds and M/s, respectively. All the kinetic parameters were obtained and/or calculated from published literature (Data Sheets 1, 2, Table S2). To observe the effect of different parameters on the models sensitivity analyses was performed using deterministic LSODA ODE solver (Hindmarsh, 1983).

## Results

### Model Reconstruction and Network Topology

**Figure 3:**
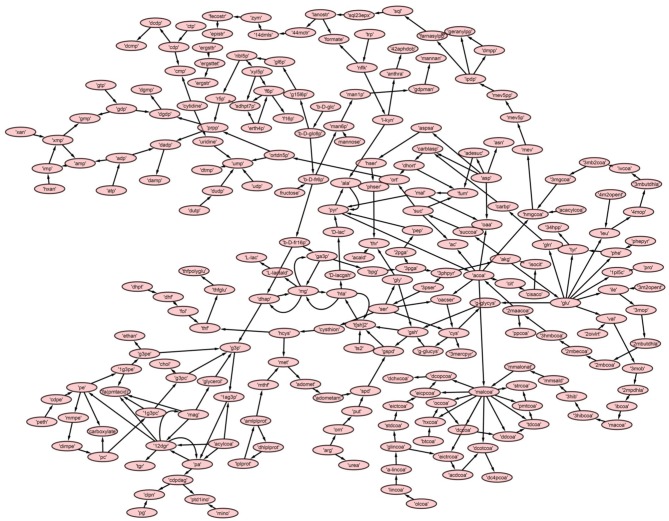
The reconstructed master model, M, after removing currency metabolites and self-loops.

A directed master network, *M*, was built after editing **iAC560** by doing following: all transport and exchange reactions were removed; all self-loops, currency metabolites (ATP, ADP, CO_2_, etc.), electron carriers (NADP^+^, NADPH, NAD, NADH), Waters (H_2_O), and electron transporters (H^+^, ${\text{PO}}_{4}^{+3}$, ${\text{HCO}}_{3}^{-}$) were removed; each reversible reaction in the network was converted to two irreversible backward and forward reactions; then, each irreversible reaction was considered as directed edge connecting the substrate metabolite to the reaction or reaction to the product metabolite; duplicate reactions and metabolites were carefully observed in the network and removed. Moreover, each directed edge was assigned with a weight corresponding to the stoichiometry of the involved metabolite (substrate or product) in the reaction under consideration. This model consisted of 226 metabolites (Table 1, Figure 3, Table S5).

**Table 1:** Topological properties of the networks (Table S5).

**Model**	**Main components**	**No. of metabolites**	**Clustering coefficient**	**Network diameter**
M	–	226	0.038	31
M1	Glycolysis, TCA cycle, PPP pathway	39	0.026	17
M2	Amino acid metabolism	81	0.022	13
M3	Fatty acid synthesis and degradation	23	0.118	8
M4	Sterol biosynthesis	23	0.051	17
M5	Glycerolipid and glycerophospholipid metabolism	27	0.046	12
M6	Nucleic acid metabolism	36	0.017	8
M7	MGO and T[SH]_2_ metabolism	33	0.010	12

Further, to get a deeper insight of the *Leishmanial* metabolic network, the master model, *M*, was subdivided into small directed bipartite graphs (Table 1, Figure S1). The network topology of networks is summarized in Table 1 and Table S3. The clustering coefficient (ranges between 0 to 1; no to high interconnectivity) of the master network, *M*, was 0.038. Among the models M1-M7, only M7 had the lowest clustering coefficient 0.01 (Table 1). The clustering coefficient of M7 indicates that the network has lesser connectivity in comparison to other networks, showing the transmission of the signal is linear and in one direction.

Further, betweenness centrality calculated for each node in M network pointed out important metabolites. Nodes having high betweenness centrality are known to act as bridge between nodes for transmission of information in the network. In our M model several of these metabolites (HTA, MG, Pyr, T[SH]_2_) with high betweenness centrality were part of M7 model (Table S3) suggesting the importance of M7 model (Chauhan and Singh, 2019).

### Curvature Distribution in Master Network

**Figure 4:**
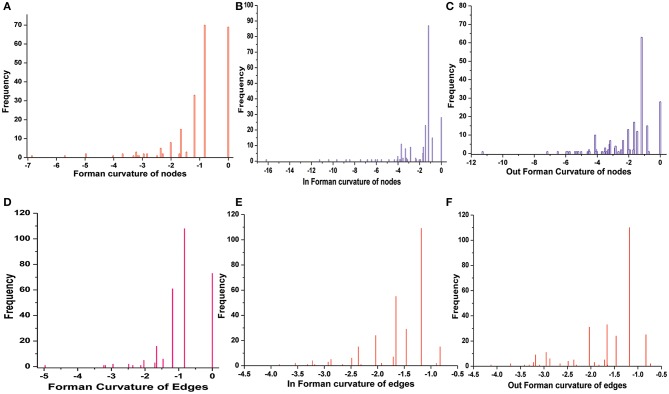
Distribution of Forman curvature of directed edges and nodes in master (M) network. **(A)** Forman curvature of nodes, **(B)** In Forman curvature of nodes, **(C)** Out Forman curvature of nodes, **(D)** Forman curvature of Edges, **(E)** In Forman curvature of edges, **(F)** Out Forman curvature of Edges.

**Figure 5:**
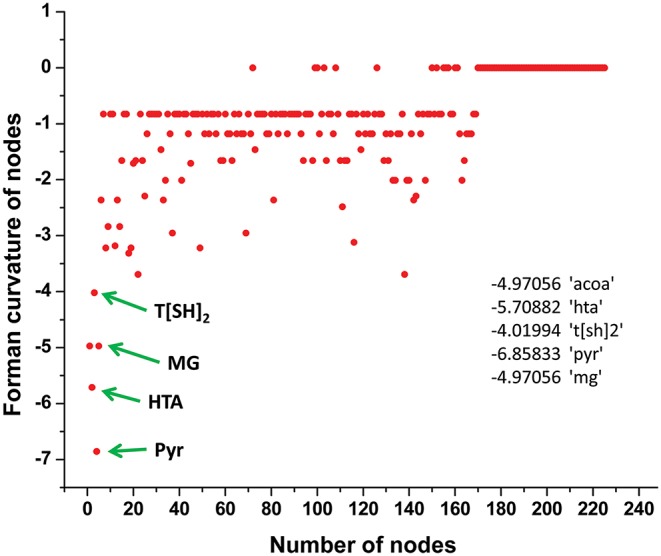
Demonstration of Forman curvature of nodes in master model. Metabolites with high curvature values are labeled and pointed with green arrows.

Normalized (Forman) and unnormalized (Forman-Ricci) Forman curvatures of directed edges and nodes were calculated in all reconstructed models (Table S4). Forman curvature of nodes and edges in network “M” was computed and their distribution was plotted to analyze their frequency (Figure 4). From the plots, it was observed that most of the nodes and edges were comprised of negative curvature values. Although, the distribution of Forman curvature of both nodes and edges was broad, the peaks with high frequency were concentrated toward (or at) “zero.” The distribution of Fi and Fo of nodes were found to be slightly different than Forman curvature of nodes however the peaks between 0 and −2 were found to be with frequency >50. Similar was the case with Fi and Fo of edges where one peak in both the plots had frequency >50. Further extraction of these peaks demonstrated that metabolites belonging to amino acid metabolism, sterol synthesis, fatty acid biosynthesis and degradation, glycerolipid and glycerophospholipid metabolism pathways were part of these high peaks. Interestingly, peaks appearing far from “zero,” those with higher Forman curvature, consisted of edges and nodes related to glycolysis, MG metabolism and T[SH]_2_ metabolism pathways. Furthermore, these metabolites were also spotted as important vertices from Forman curvature plot of nodes in “M” model (Figure 5) (Chauhan and Singh, 2019) (Table S4).

### Correlation Between Forman Curvature and Common Network Measures

Various common statistical topological measures were calculated for all networks and studied to find any significant correlation with the curvature measures (Table 2).

**Table 2:** Summary of *R*-values for different models.

	***R*****-value**													
	**Fi**		**FRi**		**Fo**		**FRo**		**F(v)**			**Betweenness centrality**	
	**Betweenness centrality**	**In- degree**	**Betweenness centrality**	**In- degree**	**Betweenness centrality**	**Out- degree**	**Betweenness centrality**	**Out- degree**	**Betweenness centrality**	**Closeness centrality**	**Degree**	**Clustering coefficient**	**In- degree**	**Out- degree**
M	−0.457	**−0.855**	−0.388	**−0.672**	−0.422	−0.215	−0.294	−0.014	**−0.688**	−0.060	−0.091	−0.146	0.387	0.403
M1	−0.335	−0.069	−0.490	−0.114	−0.394	−0.210	−0.230	−0.040	−0.350	0.109	−0.312	−0.117	0.062	0.330
M2	−0.140	**−0.851**	−0.289	**−0.507**	−0.206	−0.122	−0.276	−0.062	−0.409	−0.337	−0.704	−0.198	0.155	0.434
M3	−0.134	**−0.816**	−0.360	**−0.599**	−0.299	−0.127	−0.265	0.160	−0.347	−0.444	0.0281	**−0.576**	0.226	0.277
M4	−0.263	**−0.970**	−0.508	**−0.776**	−0.264	−0.131	−0.264	−0.054	**−0.596**	−0.209	−0.572	−0.426	0.290	0.469
M5	−0.614	**−0.941**	0.017	0.160	**−0.698**	−0.498	−0.039	−0.023	**−0.849**	−0.116	**−0.854**	−0.057	0.504	0.544
M6	−0.297	**−0.945**	−0.169	**−0.807**	−0.438	−0.001	−0.027	0.249	**−0.520**	−0.449	**−0.782**	−0.459	0.372	0.424
M7	**−0.847**	**−0.920**	**−0.766**	**−0.860**	**−0.865**	−0.600	**−0.658**	−0.177	**−0.897**	−0.240	**−0.903**	−0.242	**0.793**	**0.670**

*Fi and Fo, In and Out Forman curvature; FRi and FRo, In and Out Forman-Ricci curvature; F(v), Forman curvature of nodes. Bold values represents the high negative correlation values compared to other values in the table*.

#### Master Model

**Figure 6:**
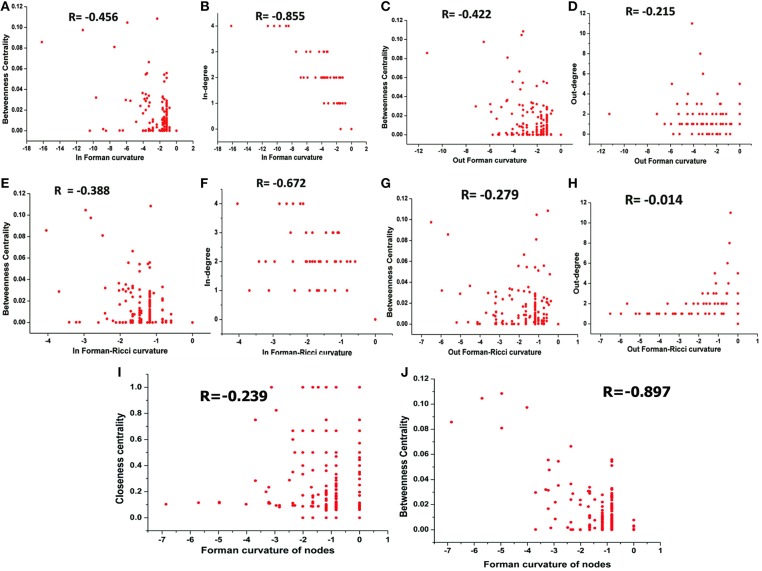
Correlation between **(A)** Fi-Betweenness centrality; **(B)** Fi-In-degree; **(C)** Fo-Betweenness centrality; **(D)** Fo-Out-degree; **(E)** FRi-Betweenness centrality; **(F)** FRi-In-degree; **(G)** FRo-Betweenness centrality; **(H)** FRo-Out-degree; **(I)** Fv-Closeness Centrality; and **(J)** Fv-Betweenness Centrality of nodes in Master “M” network. Also, Spearman correlation coefficient is indicated for each.

The correlation between Forman curvatures and betweenness centrality, closeness centrality, In-degree and Out-degree were computed in “M” model (Table 2, Figure 6). A high negative correlation was observed for Fi and FRi with In-degree (Figures 6B,F). Moderate negative correlation was reported for Fi, FRi, Fo with betweenness centrality (Figures 6A,C,E). Interestingly, In-degree and Out-degree have also shown moderate correlation with betweenness centrality but the R-value was shifted to positive side (Table 2). Moreover, in contrast, Fo and FRo have shown very less or near negligible correlation with Out-degree and betweenness centrality, respectively (Figures 6D,G,H). Forman curvature of nodes was also negatively correlated with high magnitude to betweenness centrality than that of closeness centrality suggesting the importance of the former in finding important nodes in the network (Table 2, Table S4).

#### Small Scale Models

It was noted that, in all models (M1-M7) except M3 (−0.576), the correlation between Forman curvature of nodes and clustering coefficient was very weak negative in comparison to correlation between Forman curvature and degree. Interestingly, the highest negative correlation between Forman curvature and degree was mainly noted for model M5, M6 and M7 (−0.854, −0.782, and −0.903, respectively) (Table 2). Further, weak negative correlation coefficient between closeness centrality and Forman curvature of nodes was obtained.

On the other hand, a good negative correlation of betweenness centrality with Fi and FRi was observed only in case of M5, M7 and M4, M7, respectively. Surprisingly, in contrast to Fi and FRi, correlation for Fo and FRo with out-degree and betweenness centrality was noted very weak to moderate negative. Among the small scale models, only M7 indicated high negative correlation for Fo and FRo with betweenness centrality. It was found similar to the correlation for betweenness centrality and In- and Out-degree for M7 model (high positive correlation) (Table S4).

### Robustness of Models

**Figure 7:**
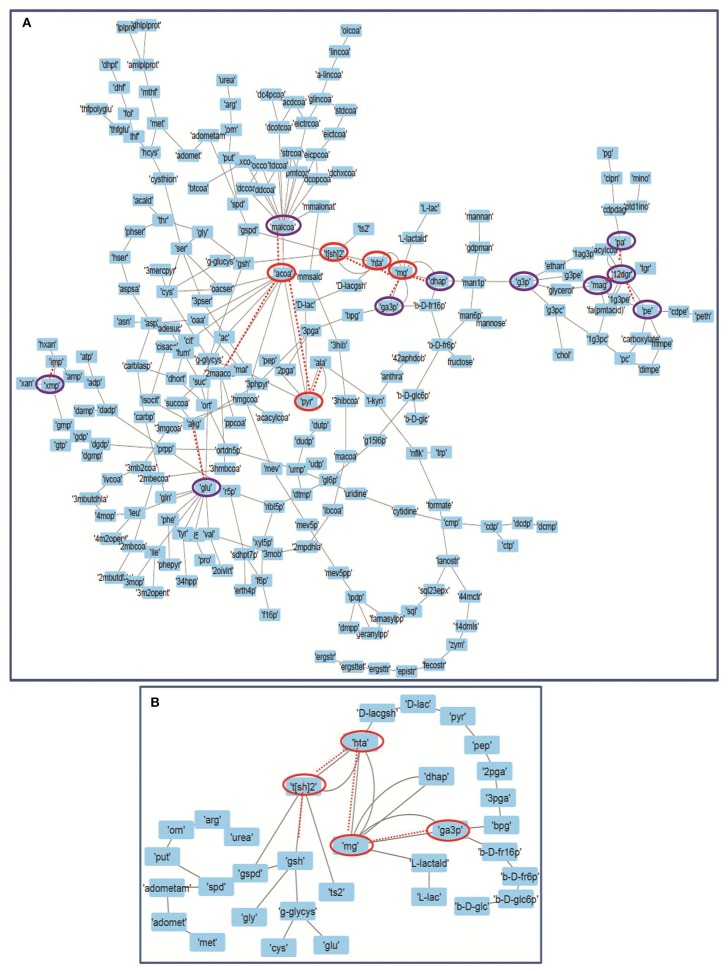
Depicting the internal connectivity in **(A)** Master model, and **(B)** M7 model. The components are connected by *backbone* forming high curvature nodes [encircled in red color (top 5) and purple (next top 10)]. Low curvature nodes are shown without encircled nodes. High curvature nodes act as *bridges* between major network communities and play important role in determining structural and functional organization of network. Dotted lines depicting the connection of high curvature nodes with important individual network communities.

**Figure 8:**
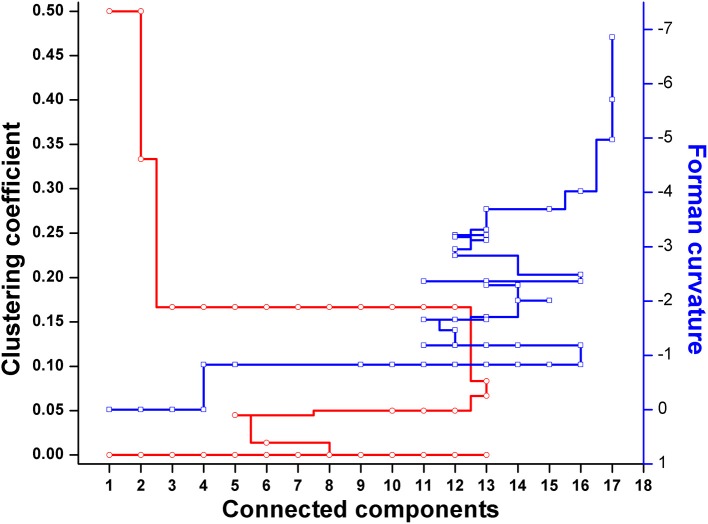
Robustness analysis of the Master model (M) using nodes deletion on the basis of Forman and Forman-Ricci curvature, or clustering coefficient.

Forman and Forman-Ricci curvatures across all edges and nodes of the network were computed. High curvature nodes are said to be the *backbone* of the network those act as *bridges* between major network communities (Varma and Palsson, 1993) (Figures 6, 7, Figure S1). Hence, for investigating the robustness in terms of “network connectivity,” the nodes were removed on the basis of decreasing strength in terms of Forman curvature, Forman-Ricci curvature and clustering coefficient. Nodes with low curvature and low clustering coefficient were removed first. Highly connected network is represented as “1,” and a higher value is an indication of disconnected network. In Figure 8, we plotted network connectivity against the nodes removed on the basis of Forman curvature and clustering coefficient. It was observed that removal of high curvature nodes caused faster disintegration of the network. Nodes deletion according to Forman-Ricci curvature gave similar result as with Forman curvature. However, nodes removal on the basis of clustering coefficient has also caused network destruction but slower than Forman curvature (Figure 8) (Sreejith et al., 2016).

In our master model we found more than 25 nodes with high curvature values (Table S3). We noted that most of the nodes from these were related to “M7” model that consists of glycolysis, T[SH]_2_ synthesis, and methylglyoxal metabolism pathways, signifying the importance of “M7” model (Chauhan and Singh, 2019).

### Flux Balance Analysis

#### Characteristics of the Reconstructed Network

As stated in previous section, the role of several metabolites including MGO and T[SH]_2_ (part of M7 model) via Forman curvature and Forman-Ricci curvature measures was found important for maintaining the integrity of the master model (Table S3). Since our focus for performing FBA was to investigate the importance of glyoxalase pathway in redox metabolism, we kept our model at simple metabolic level. The proposed stoichiometric model accounts for the reactions of glycolysis, glyoxalase pathway, and thiol metabolism (Table 3). It is well known that MGO is highly toxic entity and irreversibly modifies DNA, proteins/enzymes (Thornalley, 1993; Lo et al., 1994; Westwood et al., 1997; Greig et al., 2009) by reacting with Arg and Lys residues to form Advanced Glycation End products (AGEs) and promotes the formation of free radicals (Fraval and McBrien, 1980; Desai and Wu, 2008; Kalapos, 2008). Since, the formation of AGEs is a complex process in which MGO reacts with Lys and Arg residues of the proteins/enzymes, in our model this process was added in generalized form in which MGO directly reacts with Lys and Arg.

**Table 3:** Properties of FBA model constructed from M7 model.

**Property**	**Count**
Reactions	52
Transport reactions	22
Exchange reactions	24
Metabolites	90
Compartments	2 (Internal and External)

The exchange reactions were added to allow input-output exchange of the extracellular metabolites to enter the system from the medium or excretion of end products of any reaction out of the system. All reactions were set to the maximum for upper and lower bound limits for reversible/irreversible, uptake/excretion reactions except for glucose (Edwards and Palsson, 2000). For this small scale model, we took utmost care in including only the reactions those are required for maintaining the production of desired metabolites (Table S4). This model was submitted to BioModel Database (Chelliah et al., 2015) with the id: MODEL1909200001.

#### Formulation of Objective Function

We integrated the objective function (a mathematical equation providing drain of essential metabolites) consisting of the pseudo consumption of precursors from different pathways present in the model. To observe the effect of metabolism of MGO, substrate for glyoxalase I (GLO I), on the system; hence, we focused on the formulation of objective functions (OF) by taking into account the metabolites only used in the model:

$$\begin{array}{l} Objective\;Function:pyr+D-lac+atp+h2o->adp+pi+h \end{array}$$

The end product of glyoxalase pathway is D-lactate that ultimately leads to the formation of pyruvate. For simplicity, the stoichiometric coefficients of the precursors and metabolites used in formulation were kept as default. We considered, for our model, the end products of both the pathways, glycolysis (pyruvate) and glyoxalase pathway (D-lactate), contributing to the total biomass production. The system was investigated under two scenarios, first, functional GLO I, and second, nonfunctional (absent from the system) GLO I (Chauhan and Singh, 2019).

##### Scenario 1: Functional GLO I

**Figure 9:**
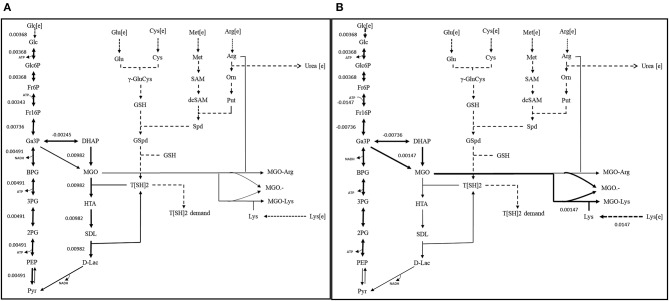
Reaction fluxes for **(A)** scenario 1 (Functional GLOI), and **(B)** scenario 2 (Non-functional GLOI). The depicted scenarios are illustrating the flow of flux for the objective function (OF). The simulation was performed for the production of pyruvate (from glycolysis and glyoxalase pathway) and role of MGO in producing AGEs (MGO-Arg and MGO-Lys) and MGO•− free radical. Solid thick black arrows represent the direction of main flux flow through the model. Gray arrows are an indication of undetermined (zero) flux through the respective reaction. Dotted arrows represent the transport of the metabolites.

It was noticed that glycolysis carried constant flux to produce pyruvate. However, glyoxalase pathway, comparatively, carried maximum flux for the production of D-lactate. It was also noticed that in presence of functional GLO I, advanced glycation end products (AGEs) formed at very minimal level. Although, both the pathways carried different flux levels, the maximization of OF was seemed to be majorly contributed through MGO synthesis and GLO I/II activities (Figure 9A). Other metabolites synthesized at very minimal level probably due to smaller scale of our model and lack of other reactions for the compensation of their consumption (Chauhan and Singh, 2019).

##### Scenario 2: Non-functional GLO I

In, second scenario, we blocked the flux through glyoxalase pathway by setting GLO I flux to “zero” making it inactive. In this case, we observed that glucose entered the system at same rate as in scenario 1. Even the flux rate was found to be more or less similar for glycolysis but only upto the synthesis of GA3P and DHAP. In fact, the flux through TPI enzyme was more than that of scenario 1 that probably contributed to the synthesis of MGO. It is noticeable that MGO was produced at twice the rate than the previous case. This higher level of accumulation of MGO was found to contribute to equal level of the formation of AGEs and MGO^•−^ free radicals (Figure 9B). It was worth noticing that in case of inactive GLO I, the flux moved from glycolysis to MGO synthesis. Although in real network system, pyruvate production will be happening at normal rate except for that the inactivation of GLO I should lead to the increased level (~1.5 fold) of accumulation of MGO (Chauhan and Singh, 2019).

### Systems Pharmacology Modeling

Initially, the basal kinetic model (non-inhibited model) was constructed by incorporating the reactions from glycolysis, Glyoxalase system and T[SH]_2_ synthesis via arginine, glycine, glutamate and methionine. Further, considering the important role of T[SH]_2_, its demand reactions were also included in the form of TXN, TDPx, and TryP. As mentioned in earlier sections that due to the highly reactive nature of MGO, it irreversibly modifies Arg and Lys residues proteins/enzymes (Thornalley, 1993; Lo et al., 1994; Westwood et al., 1997) leading to formation of AGEs and free radicals (Fraval and McBrien, 1980; Desai and Wu, 2008; Kalapos, 2008). Hence, these reactions were included in generalized form in which MGO directly reacts with Lys and Arg, and AGEs were represented in the form of modified Arg and Lys (MGO-Arg and MGO-Lys, respectively). The model also consisted of various reactions for free radical metabolism. Further, to examine the effect of inhibited GLO I reaction on the system, a perturbation was imposed using previous studied competitive LmGLO I inhibitors, S-2,4-dinitrophenylglutathionylspermidine (DNPGS) and S-4-bromobenzylglutathionylspermidine (BBGS) (ki: 669 ± 57 and 0.536 ± 0.040 μM, respectively) (Ariza et al., 2006). Hence, two different perturbed kinetic models were constructed by introducing their inhibition reactions in the non-inhibited model. These inhibition kinetic models were submitted to BioModel database (MODEL1909200002 and MODEL1909200003, BBGS and DNPGS, respectively) (Chelliah et al., 2015).

**Figure 10:**
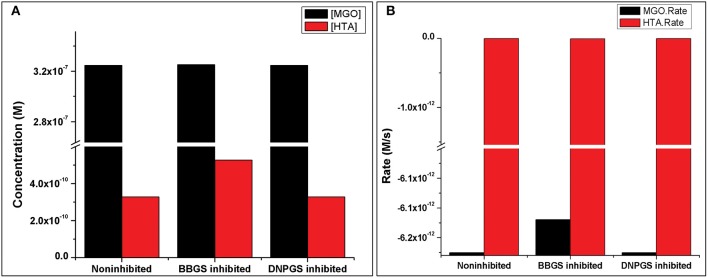
Illustration of effect of two LmGLO I competitive inhibitors, BBGS and DNPGS on **(A)** MGO and HTA concentration, **(B)** on MGO and HTA formation rates. BBGS, S-4-bromobenzylglutathionylspermidine; DNPGS, S-2,4-dinitrophenylglutathionylspermidine.

Analysis of the time course simulation of non-inhibited model has showed the maximum formation and degradation of MGO via glycolysis and GLOI enzyme, respectively. the maximum concentration of MGO was considered as initial concentration for both the inhibitors. GLOI reaction involves conversion of HTA to SDL. Although HTA is formed in reversible manner from MGO and T[SH]_2_ non-enzymatically, we assumed that the introduced inhibition reaction, along with level of HTA, should also affect the level of MGO. However, when the comparison was made between non-inhibited and inhibited models, it was observed that even after the incorporation of inhibition reaction for GLOI, only HTA was accumulated in the system and not MGO (Figure 10, Figures S2, S3).

Comparison of metabolites' concentration and their production rates among the models revealed that the synthesis rate was higher in non-inhibited and DNPGS inhibited model as compared to BBGS inhibited model (Tables 4A,B). Both the inhibitors affected the level of few metabolites (free radicals) but BBGS was found to have greater effect. In BBGS inhibited model, the HTA accumulation was observed to increase by 1.6 fold in comparison to DNPGS inhibited model. Although, we did not observe any change in the level of Arg-MGO and Lys-MGO concentration in the system, however, the rate of their formation is increased by 1–3 folds in case of BBGS inhibited model. Through our kinetic modeling study, we are able to shed an important light on the mechanism of inhibition of GLO I reaction.

**Table 4A:** Effect of GLO I inhibition on metabolites produced.

**Metabolite**	**Fold increase**	
	**BBGS^**#**^**	**DNPGS^**#**^**
[MGO]	0x	1.0x
[HTA]	1.6x	1.0x
[DL]	1.0x	1.0x
[SDLTSH]	1.0x	1.0x
[Arg-MG]	1.0x	1.0x
[Lys-MG]	1.0x	1.0x
[H_2_O_2_]	1089195.2x	573.8x
[^•^OH]	1029612.2x	242.0x
[O_2_^•^−]	1.0x	1.0x
[L^•^]	1.0x	1.0x
[LO_2_^•^]	1.0x	1.0x
[LOOH]	1.0x	1.0x
[NO^•^]	1.0x	1.0x
[${\text{NO}}_{2}^{-}$]	1080227.9x	569.1x
[ONOOH]	1.0x	1.0x
[ONOO^−^]	1066456.4x	569.3x
[${\text{NO}}_{2}^{-}$]	1.0x	1.0x
[MGO^•−^]	1.0x	1.0x

# *BBGS, S-4-bromobenzylglutathionylspermidine; DNPGS, S-2,4-dinitrophenylglutathionylspermidine*.

**Table 4B:** Effect of GLO I inhibition on metabolite rates.

**Rates**	**Fold increase**	
	**BBGS^**#**^**	**DNPGS^**#**^**
MGO	1.0x	1.0x
HTA	1.6x	1.0x
DL	1.0x	1.0x
SDLTSH	1.0x	1.0x
Arg-MG	3.2x	0.3x
Lys-MG	1.0x	1.0x
H_2_O_2_	−16612060.7x	−8734.8x
^•^OH	−91357.6x	−5999.1x
O_2_^•^-	21950717.4x	−2122.4x
LO_2_	1.0x	1.0x
LOOH	1.0x	1.0x
NO_2_	−11634149.8x	−45781.9x
ONOOH	1080215.6x	569.0x
ONOO^−^	1532627984.1x	−19610.4x
${\text{NO}}_{2}^{-}$	1080230.2x	569.1x
MGO^•−^	1.0x	1.0x

# *BBGS, S-4-bromobenzylglutathionylspermidine; DNPGS, S-2,4-dinitrophenylglutathionylspermidine*.

**Figure 11:**
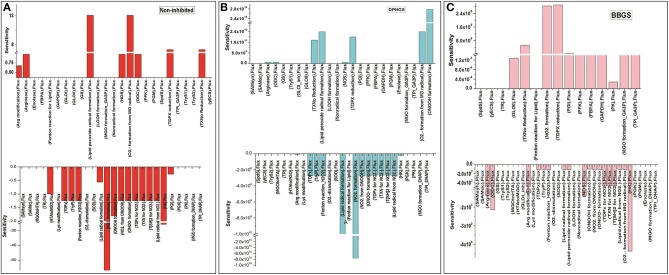
Illustration of Sensitivity analysis of kinetic models. For sensitivity analyses in these models, Vmax of GLO I was used to observe its influence on the flux of other reactions, **(A)** non-inhibited model; **(B)** BBGS inhibited model; **(C)** DNPGS inhibited model.

Further, to observe how GLO I inhibition has influence on the perturbed models; sensitivity analysis of the networks was carried out. For this purpose, GLO I reaction parameters, GLOI_Vmax_ was used. Although perturbation did not greatly influence the accumulation of MGO in the system, the sensitivity analysis demonstrated that the inhibited GLOI_Vmax_ affected other reactions fluxes. It seems like BBGS had more effect on other reaction fluxes than DNPGS when compared with the non-inhibited model. Between the two perturbed models, GLO I inhibition using BBGS negatively affected GLO I reaction flux while DNPGS did not have any effect (Figure 11). Also, despite of no effect of inhibition on MGO level in the system, the rate of BBGS inhibited GLO I reaction influenced the flux of MGO formation from 2 to 10 folds.

## Discussion

To discover the robustness and reliability of a biological network it is important to understand its basic structural framework by quantitatively analyzing it through mathematical tools (Watts and Strogatz, 1998; Barabási and Oltvai, 2004; Dorogovtsev and Mendes, 2013; Weber et al., 2017a,b). This can help in uncovering the meaningful information by reducing the system to a smaller level to understand the relationship among its various components (Burago et al., 2001; Ollivier, 2009, 2011; Ni et al., 2015; Sandhu et al., 2015). In current work, we motivate the use of systems biology driven approaches including Forman curvature and Forman-Ricci curvature measures along with FBA for the analysis of complex metabolic network of Leishmania parasite.

Forman and Forman-Ricci curvature of master network has shown that nodes with high curvatures differ drastically in structure from the ones with low curvature values and appear to be in denser clusters acting as bridges between the sub-clusters in the network. This measure not only helped us to identify the relatively more important parts of the network but also provided the information on identifying the sub-networks. This gave us the idea to break the network in sub-models to study the properties of various pathways at smaller scale and has helped us to detect important substructures inside the network belonging to particular classes of vertices or edges. Importantly, our analyses have pointed out several central nodes belonging to very common yet crucial metabolic pathways including glyoxalase pathway and T[SH]_2_ metabolism pathways (Figures 5, 7, Figure S1). The significant importance of these high curvature nodes has also been demonstrated while performing the robustness analysis on the network (Figure 8). In fact, high correlation between Forman/Forman-Ricci curvature measures and common network properties has also demonstrated and pointed out the structural and functional importance of “M7” model comprising these metabolites (Table 2). The assumptions made, here, are in congruence with the validated experimental literature and are an inherent feature of the model which signifies the importance of the metabolites undertaken to study the impact of the curvature measures. However, any modification of the connectivity matrices would certainly impact the curvature measures for a particular built up resampled model and would also impact the bridging of the metabolites.

It is worth noticing that several of these metabolites (MGO and T[SH]_2_) have very important role in the regulation of redox homeostasis of the parasite. MGO is highly cytotoxic carbonyl and can irreversibly modifies DNA, proteins/enzymes (Thornalley, 1993; Lo et al., 1994; Westwood et al., 1997) causing the formation of advanced glycation end products (AGEs) and free radicals.

The neutralization of MGO is carried out by glyoxalase system comprising two key enzymes, GLO I and GLO II (Vickers et al., 2004; Greig et al., 2009; Wyllie and Fairlamb, 2011; Sousa Silva M et al., 2012). These two enzymes sequentially convert MGO into D-lactate in a two steps process: GLO I mediated isomerization of spontaneously formed MGO-T[SH]_2_ conjugate, hemi-thioacetal (HTA), to S-D-lactoyltrypanothione, followed by GLOII mediated D-lactate formation (Thornalley, 1990). GLO I is reported as an essential and rate limiting enzyme in *Leishmania*. The essential role of GLOI has been demonstrated earlier in GLOI attenuated *Leishmania donovani* strain (Chauhan and Madhubala, 2009).

Our FBA result has also demonstrated similar information when we applied two different conditions to the model. Although, the OF we designed only serves as a tool to identify feasible flux across the model system, this has demonstrated the key role of GLO I. The non-functional GLO I has caused “zero” flux and led to several fold increased production of MGO (Figures 9A,B). The increased production of MGO in the system caused its accumulation due to under-determined flux in the downstream glyoxalase pathway and, hence, lack of its detoxification. The accumulation of MGO in the system consequently led to the formation of AGEs and MGO^•−^ free radicals. Moreover, in scenario 2, glyoxalase pathway has also reflected the rate limiting step in very agreeable manner in the form of carrying the “zero” flux through the glyoxalase pathway reactions. These findings revealed the importance of GLO I and are in the agreement with the previous reports. Although, due to maintaining the simplicity of the model, we have not shown the effect of MGO^•−^ free radical on other components in the system, however it has been demonstrated earlier that the excess amount of MGO causes the formation of increased level of AGEs and MGO^•−^ free radicals (Nohara et al., 2002; Desai and Wu, 2008; Kalapos, 2008) and can inhibit the growth of *Leishmania*.

In our model, emergent metabolic properties of GLO(I) have been studied in a piecewise manner considering functional and non-functional GLO(I). We are well aware that there might be a change in metabolite vs. enzyme levels and any feedback introduced at all enzyme levels in the pathway of interest may give rise to different phenotype and correspondingly stable or sustained oscillations. Having said that, with our simplified model using a mathematical formalism wherein output depends on only two conditions in the pathway: functional and non-functional GLO I, these studies have been taken into consideration. Moreover, a measure of robustness in metabolic network accounts for the relative changes in the concentration of metabolites to all other metabolites. Our reduced model with local perturbations has a larger impact in a restricted sub-network model system which is able to absorb the impact which a whole system model might not be able to bear.

Having stated above, for most part of the reconstructed network, taking into account the different point of observations, interaction dynamics does not drastically changed. The integrated analysis laid in this paper from computational perspectives helped pinpoint an interesting phenomenon that the underlined network model structure is largely preserved across the large common substructure models. Nonetheless, there might be instances at which major changes can occur in the network often in response to the metabolic or environmental stress. With the theoretical framework laid in this manuscript we are able to represent an *in silico* analog of the curvature measure and FBA. Therefore, the strategy outlined here may become an indispensable tool in future for analyzing smaller sub-network models created from a larger model. Using a model like this amounts to endowing the system with enhanced robustness and investigate how the dynamics might get affected. GLO I as a target deciphered in present work can further be validated through kinetic modeling and experimental validation to pinpoint its influence on direct or indirect metabolites.

## Conclusion

In the quest to improve the current understanding of biological networks we have devised a newer strategy of formulation of curvature measure along with FBA in order to explain and predict biological targets. The methodology adopted in this paper has enabled us to determine the implicit relationship between metabolites. The model prototype developed in current paper largely depends on its structure and topological components. On the broader note, we have developed our mathematical model in an iterative fashion to optimize the flux of the metabolic system under limitation of certain enzymatic reactions thus leveraging the unifying framework for curvature measures along with FBA. GLOI deciphered as an important target both from curvature as well as FBA enabled our deeper understanding into the parasite redox metabolism.

## Data Availability Statement

The raw data supporting the conclusions of this manuscript will be made available by the authors, without undue reservation, to any qualified researcher.

## Author Contributions

SS laid the design idea and implementation of the project. NC performed the computational modeling. NC and SS wrote the original manuscript and approved the final version of the manuscript.

### Conflict of Interest

The authors declare that the research was conducted in the absence of any commercial or financial relationships that could be construed as a potential conflict of interest.

## Supplementary Material

Figure S1 Depicting the internal connectivity in small scale models. The components are connected by *backbone* forming high curvature nodes (encircled in red color). Low curvature nodes are shown without encircled nodes. High curvature nodes act as *bridges* between major network communities and play important role in determining structural and functional organization of network.

Figure S2 Illustration of effect of two LmGLO I competitive inhibitors, BBGS and DNPGS on other metabolites concentrations in non-inhibited and inhibited kinetic models BBGS, S-4-bromobenzylglutathionylspermidine; DNPGS, S-2,4-dinitrophenylglutathionylspermidine.

Figure S3 Illustration of effect of two LmGLO I competitive inhibitors, BBGS and DNPGS on production rates of other metabolites in non-inhibited and inhibited kinetic models BBGS, S-4-bromobenzylglutathionylspermidine; DNPGS, S-2,4-dinitrophenylglutathionylspermidine.

Table S1 List of various reactions and assigned rate laws used for kinetic modeling.

Table S2 Kinetic parameters and initial concentration of metabolites used in the kinetic model system.

Table S3 Topological properties of the Master model.

Table S4 Cytoscape statistics calculation and curvature measurement of Master and M7 model.

Table S5 List of various metabolic components of Master and M1 to M7 models.

## Abbreviations

FBA: Flux Balance Analysis
MGO: Methylglyoxal
GLOI: Glyoxalase I
GLOII: Glyoxalase II
MGO^•−^: Methylglyoxal Free Radical
CL: Cutaneous Leishmaniasis
DHAP: Dihydroxyacetone phosphate
GA3P: Glyceraldehyde 3-phosphate
T[SH]_2_: Trypanothione
TryS: Trypanothione synthase
TryR: Trypanothione reductase
TryP: Tryparedoxin peroxidase
TDPx: Tryparedoxin-dependent peroxidase
TXN: Tryparedoxin
GR: Glutathione reductase
GEMs: Genome scale metabolic modeling
ODE: Ordinary differential equations
FR: Forman-Ricci curvature
FR_(v)_: Forman-Ricci curvature of nodes
FR_I(v)_: In Forman-Ricci curvature of nodes
FR_O(v)_: Out Forman-Ricci curvature of nodes
Fv: Forman curvature of nodes
Fo: Out Forman
Fi: In Forman
OF: Objective functions
BBGS: S-4-bromobenzylglutathionylspermidine
DNPGS: S-2,4-dinitrophenylglutathionylspermidine.

## References

[B1] Ariza A. Vickers T. J. Greig N. Armour K. A. Dixon M. J. Eggleston I. M.. (2006). Specificity of the trypanothione-dependent Leishmania major glyoxalase I: structure and biochemical comparison with the human enzyme. Mol. Microbiol. 59, 1239–1248. 10.1111/j.1365-2958.2006.05022.x 16430697

[B2] Ascenzi P. Bocedi A. Visca P. Antonini G. Gradoni L. (2003). Catalytic properties of cysteine proteinases from *Trypanosoma cruzi* and *Leishmania infantum*: a pre-steady-state and steady-state study. Biochem. Biophys. Res. Commun. 309, 659–665. 10.1016/j.bbrc.2003.08.015 12963041

[B3] Barabási A. L. Oltvai Z. N. (2004). Network biology: understanding the cell's functional organization. Nat. Rev. Genet. 5, 101–113. 10.1038/nrg1272 14735121

[B4] Becker S. A. Feist A. M. Mo M. L. Hannum G. Palsson B. Ø. Herrgard M. J. (2007). Quantitative prediction of cellular metabolism with constraint-based models: the COBRA Toolbox. Nat. Protoc. 2, 727–738. 10.1038/nprot.2007.99 17406635

[B5] Burago D. Burago Y. Ivanov S. (2001). A Course in Metric Geometry, Vol. 33. Providence, RI: American Mathematical Society Providence 10.1090/gsm/033

[B6] Bytautiene B. (2013). Graph theory modeling for diagnosing presymptomatic Alzheimer's disease. Sci. Transl. Med. 5:210ec182. 10.1126/scitranslmed.3007931

[B7] Case M. Shirinpour S. Vijayakumar V. Zhang H. Datta Y. Nelson S.. (2018). Graph theory analysis reveals how sickle cell disease impacts neural networks of patients with more severe disease. Neuroimage Clin. 21:101599. 10.1016/j.nicl.2018.11.009 30477765 PMC6411610

[B8] Chauhan N. Singh S. (2019). Integrative computational framework for understanding metabolic modulation in leishmania. bioRxiv [Preprint]. 10.1101/512277 PMC6877600 31803732

[B9] Chauhan S. C. Madhubala R. (2009). Glyoxalase I gene deletion mutants of *Leishmania donovani* exhibit reduced methylglyoxal detoxification. PLoS ONE 4:e6805. 10.1371/journal.pone.0006805 19710909 PMC2728510

[B10] Chavali A. K. Whittemore J. D. Eddy J. A. Williams K. T. Papin J. A. (2008). Systems analysis of metabolism in the pathogenic trypanosomatid *Leishmania major*. Mol. Syst. Biol. 4:177. 10.1038/msb.2008.15 18364711 PMC2290936

[B11] Chelliah V. Juty N. Ajmera I. Ali R. Dumousseau M. Glont M.. (2015). BioModels: ten-year anniversary. Nucleic Acids Res. 43, D542–D548. 10.1093/nar/gku1181 25414348 PMC4383975

[B12] Desai K. M. Wu L. (2008). Free radical generation by methylglyoxal in tissues. Drug Metabol. Drug Interact. 23, 151–173. 10.1515/DMDI.2008.23.1-2.151 18533368

[B13] Dorogovtsev S. N. Mendes J. F. F. (2013). Evolution of Networks: From Biological Nets to the Internet and WWW. New York, NY: Oxford University Press.

[B14] Edwards J. S. Palsson B. O. (2000). The *Escherichia coli* MG1655 *in silico* metabolic genotype: its definition, characteristics, and capabilities. Proc. Natl. Acad. Sci. U.S.A. 97, 5528–5533. 10.1073/pnas.97.10.5528 10805808 PMC25862

[B15] Evans L. C. (2001). Partial differential equations and Monge-Kantorovich mass transfer. Curr. Dev. Math. 1997, 65–126. 10.4310/CDM.1997.v1997.n1.a2

[B16] Fatumo S. Plaimas K. Mallm J. P. Schramm G. Adebiyi E. Oswald M.. (2009). Estimating novel potential drug targets of *Plasmodium falciparum* by analysing the metabolic network of knock-out strains *in silico*. Infect. Genet. Evol. 9:351.e358. 10.1016/j.meegid.2008.01.007 18313365

[B17] Forman R. (2003). Bochner's method for cell complexes and combinatorial Ricci curvature. Discret Comput Geom. 29, 323–374. 10.1007/s00454-002-0743-x

[B18] Fraval H. N. McBrien D. C. (1980). The effect of methyl glyoxal on cell division and the synthesis of protein and DNA in synchronous and asynchronous cultures of Escherichia coli B/r. J. Gen. Microbiol. 117, 127–134. 10.1099/00221287-117-1-127 6993622

[B19] Funahashi A. Tanimura N. Morohashi M. Kitano H. (2003). CellDesigner: a process diagram editor for gene-regulatory and biochemical networks. BIOSILICO 1, 159–162. 10.1016/S1478-5382(03)02370-9

[B20] Gao J. Gu D. X. Luo F. (2016). Discrete ricci flow for geometric routing, in Encyclopedia of Algorithms, ed Kao M. Y. (New York, NY: Springer).

[B21] Greig N. Wyllie S. Patterson S. Fairlamb A. H. (2009). A comparative study of methylglyoxal metabolism in trypanosomatids. FEBS J. 276, 376–386. 10.1111/j.1742-4658.2008.06788.x 19076214 PMC2702497

[B22] Hädicke O. Grammel H. Klamt S. (2011). Metabolic network modeling of redox balancing and biohydrogen production in purple nonsulfur bacteria. BMC Syst. Biol. 5:150. 10.1186/1752-0509-5-150 21943387 PMC3203349

[B23] Heirendt L. Arreckx S. Pfau T. Mendoza S. N. Richelle A. Heinken A. (2018). Creation and analysis of biochemical constraint-based models: the COBRA Toolbox v3.0. Nat. Protoc. 14, 639–702. 10.1038/s41596-018-0098-2 PMC6635304 30787451

[B24] Hindmarsh A. C. (1983). ODEPACK, a systematized collection of ODE solvers. Sci. Comput. 1, 55–64.

[B25] Ho T. C. (2008). Kinetic modeling of large-scale reaction systems. Sci. Eng. 50, 287–378. 10.1080/01614940802019425

[B26] Hoops S. Sahle S. Gauges R. Lee C. Pahle J. Simus N.. (2006). COPASI. A COmplex Pathway SImulator. Bioinformatics 22, 3067–3074. 10.1093/bioinformatics/btl485 17032683

[B27] Kabra R. Chauhan N. Kumar A. Ingale P. Singh S. (2018). Efflux pumps and antimicrobial resistance: paradoxical components in systems genomics. Prog. Biophys. Mol. Biol. 141, 15–24. 10.1016/j.pbiomolbio.2018.07.008 30031023 PMC7173168

[B28] Kalapos M. P. (2008). The tandem of free radicals and methylglyoxal. Chem. Biol. Interact. 171, 251–271. 10.1016/j.cbi.2007.11.009 18164697

[B29] Krauth-Siegel R. L. Inhoff O. (2003). Parasite-specific trypanothione reductase as a drug target molecule. Parasitol. Res. 90, S77–S85. 10.1007/s00436-002-0771-8 12709793

[B30] Kumar A. Chauhan N. Singh S. (2019). Understanding the cross-talk of redox metabolism and fe-s cluster biogenesis in leishmania through systems biology approach. Front. Cell. Infect. Microbiol. 9:15. 10.3389/fcimb.2019.00015 30778378 PMC6369582

[B31] Lo T. W. Westwood M. E. McLellan A. C. Selwood T. Thornalley P. J. (1994). Binding and modification of proteins by methylglyoxal under physiological conditions. A kinetic and mechanistic study with N alpha-acetylarginine, N alpha-acetylcysteine, and N alpha-acetyllysine, and bovine serum albumin. J. Biol. Chem. 269, 32299–32305. 7798230

[B32] Loisel B. Romon P. (2014). Ricci curvature on polyhedral surfaces via optimal transportation. Axioms 3, 119–139. 10.3390/axioms3010119

[B33] Mahadevan R. Palson B. O. (2005). Properties of metabolic networks: structure versus function. Biophys. J. 88, L07–L09. 10.1529/biophysj.104.055723 15574705 PMC1305059

[B34] Narain J. P. Dash A. P. Parnell B. Bhattacharya S. K. Barua S. Bhatia R.. (2010). Elimination of neglected tropical diseases in the South-East Asia Region of the World Health Organization. Bull. World Health Organ. 88, 206–210. 10.2471/BLT.09.072322 20428388 PMC2828791

[B35] Ni C. C. Lin Y. Y. Gao J. Gu X. D. Saucan E. (2015). Ricci curvature of the internet topology, in 2015 IEEE Conference on Computer Communications (INFOCOM). arXiv[Preprint]. arXiv:1501.04138v1. 10.1109/INFOCOM.2015.7218668

[B36] Nohara Y. Usui T. Kinoshita T. Watanabe M. (2002). Generation of superoxide anions during the reaction of guanidino compounds with methylglyoxal. Chem. Pharm. Bull. 50, 179–184. 10.1248/cpb.50.179 11848206

[B37] O'Brien E. J. Monk J. M. Palsson B. O. (2015). Using genome-scale models to predict biological capabilities. Cell 161, 971–987. 10.1016/j.cell.2015.05.019 26000478 PMC4451052

[B38] Ollivier Y. (2009). Ricci curvature of Markov chains on metric spaces. Journal of Functional Analysis. 256, 810–864. 10.1016/j.jfa.2008.11.001

[B39] Ollivier Y. (2010). A survey of Ricci curvature for metric spaces and Markov chains. Adv. Stud. Pure Math. 57, 343–381. 10.2969/aspm/05710343

[B40] Ollivier Y. (2011). A visual introduction to Reimannian curvatures and some discrete generalizations, in Analysis and Geometry of Metric Measure Spaces: Lecture Notes of the 50th Séminaire de Mathématiques Supérieures (SMS), Montréal, 2011, Vol. 56 (Providence, RI: American Mathematical Society), 220.

[B41] Orth J. D. Thiele I. Palsson B. Ø. (2010). What is flux balance analysis? Nat. Biotechnol. 28, 245–248. 10.1038/nbt.1614 20212490 PMC3108565

[B42] Pillay C. S. Hofmeyr J. H. Mashamaite L. N. Rohwer J. M. (2013). From top-down to bottom-up: computational modeling approaches for cellular redoxin networks. Antioxid. Redox Signal. 18, 2075–2086. 10.1089/ars.2012.4771 23249367

[B43] Resat H. Petzold L. Pettigrew M. F. (2009). Kinetic modeling of biological systems. Methods Mol. Biol. 541, 311–335. 10.1007/978-1-59745-243-4_14 19381542 PMC2877599

[B44] Samal A. Sreejith R. P. Gu J. Liu S. Saucan E. Jost J. (2018). Comparative analysis of two discretizations of Ricci curvature for complex networks. Sci. Rep. 8:8650. 10.1038/s41598-018-27001-3 29872167 PMC5988801

[B45] Sandhu R. Georgiou T. Reznik E. Zhu L. Kolesov I. Senbabaoglu Y.. (2015). Graph curvature for differentiating cancer networks. Sci. Rep. 5:12323. 10.1038/srep12323 26169480 PMC4500997

[B46] Saucan E. Appleboim E. (2005). Curvature based clustering for DNA microarray data analysis, in Pattern Recognition and Image Analysis, ed MarquesNicolás Pérez de la BlancaPedro Pina J. S. (Berlin; Heidelberg: Springer), 405–412. 10.1007/11492542_50

[B47] Shannon P. Markiel A. Ozier O. Baliga N. S. Wang J. T. Ramage D.. (2003). Cytoscape: a software environment for integrated models of biomolecular interaction networks. Genome Res. 13, 2498–2504. 10.1101/gr.1239303 14597658 PMC403769

[B48] Sharma M. Shaikh N. Yadav S. Singh S. Garg P. (2017). A systematic reconstruction and constraint-based analysis of Leishmania donovani metabolic network: identification of potential antileishmanial drug targets. Mol. Biosyst. 13:955–969. 10.1039/C6MB00823B 28367572

[B49] Sousa Silva M. Ferreira A. E. Gomes R. Tomás A. M. Ponces Freire A. Cordeiro C. (2012). The glyoxalase pathway in protozoan parasites. Int. J. Med. Microbiol. 302, 225–229. 10.1016/j.ijmm.2012.07.005 22901378

[B50] Sreejith R. P. Jost J. Saucan E. Samal A. (2017a). Forman curvature for directed networks. arXiv:1605.04662.

[B51] Sreejith R. P. Jost J. Saucan E. Samal A. (2017b). Systematic evaluation of a new combinatorial curvature for complex networks. Chao, Solitons Fractals. 101, 50–67. 10.1016/j.chaos.2017.05.021

[B52] Sreejith R. P. Mohanraj K. Jost J. Saucan E. Samal A. (2016). Forman curvature for complex networks. J. Stat. Mech. 2016:063206 10.1088/1742-5468/2016/06/063206

[B53] Subramanian A. Sarkar R. R. (2017). Revealing the mystery of metabolic adaptations using a genome scale model of *Leishmania infantum*. Sci. Rep. 7, 1–12. 10.1038/s41598-017-10743-x 28860532 PMC5579285

[B54] Tannenbaum A. Sander C. Zhu L. Sandhu R. Kolesov I. Reznik E. (2016). Graph curvature and the robustness of cancer networks. arXiv:1502.04512.

[B55] Tewari S. G. Prigge S. T. Reifman J. Wallqvist A. (2017). Using a genome-scale metabolic network model to elucidate the mechanism of chloroquine action in Plasmodium falciparum. Int. J. Parasitol. Drugs Drug Resist. 7, 138–146. 10.1016/j.ijpddr.2017.03.004 28355531 PMC5376308

[B56] Thornalley P. (1990). The glyoxalase system: New developments towards functional characterization of a metabolic pathway fundamental to biological life. Biochem. J. 269, 1–11. 10.1042/bj2690001 2198020 PMC1131522

[B57] Thornalley P. J. (1993). The glyoxalase system in health and disease. Mol. Aspects Med. 14:287. 10.1016/0098-2997(93)90002-U 8277832

[B58] Varma A. Palsson B. O. (1993). Metabolic capabilities of *Escherichia coli* 0.2. Optimal-growth patterns. J. Theor. Biol. 165, 503–522. 10.1006/jtbi.1993.1203 21322280

[B59] Vecchio F. Miraglia F. Rossini P. M. (2017). Connectome: Graph theory application in functional brain network architecture. Clin. Neurophysiol. Pract. 2, 206–213. 10.1016/j.cnp.2017.09.003 30214997 PMC6123924

[B60] Vickers T. J. Greig N. Fairlamb A. H. (2004). A trypanothione-dependent glyoxalase I with a prokaryotic ancestry in Leishmania major. Proc. Natl. Acad. Sci. U.S.A. 101, 13186–13191. 10.1073/pnas.0402918101 15329410 PMC516525

[B61] Villani C. (2003). Topics in Optimal Transportation. Providence, RI: American Mathematical Society Publications 10.1090/gsm/058

[B62] Vlassis N. Pacheco M. P. Sauter T. (2014). Fast reconstruction of compact context-specific metabolic network models. PLoS Comput. Biol. 10:e1003424. 10.1371/journal.pcbi.1003424 24453953 PMC3894152

[B63] Wang R. S. Oldham W. M. Maron B. A. Loscalzo J. (2018). Systems biology approaches to redox metabolism in stress and disease states. Antioxid. Redox Signal. 29, 953–972. 10.1089/ars.2017.7256 29121773 PMC6104248

[B64] Watts D. J. Strogatz S. H. (1998). Collective dynamics of ‘small-world’ networks. Nature 393:440. 10.1038/30918 9623998

[B65] Weber M. Saucan E. Jost J. (2017a). Characterizing complex networks with forman-ricci curvature and associated geometric flows. J. Complex Netw. 5, 527–550. 10.1093/comnet/cnw030

[B66] Weber M. Stelzer J. Saucan E. Naitsat A. Lohmann G. Jost J. (2017b). Curvature-based methods for brain network analysis. arXiv:1707.00180.

[B67] Westwood M. E. Argirov O. K. Abordo E. A. Thornalley P. J. (1997). Methylglyoxal-modified arginine residues–a signal for receptor-mediated endocytosis and degradation of proteins by monocytic THP-1 cells. Biochim. Biophys. Acta 1356, 84–94. 10.1016/S0167-4889(96)00154-1 9099994

[B68] WHO (2011). Accelerating Work to Overcome the Global Impact of Neglected Tropical Dieases: A Roadmap for implementation. Available onle at: http://www.who.int/neglected_diseases/NTD_RoadMap_2012_Fullversion.pdf

[B69] Wyllie S. Fairlamb A. H. (2011). Methylglyoxal metabolism in trypanosomes and leishmania. Semin. Cell Dev. Biol. 22, 271–277. 10.1016/j.semcdb.2011.02.001 21310261 PMC3107426

