# Supplementary material for: Integrative Computational Framework for Understanding Metabolic Modulation in Leishmania
Source: Front Bioeng Biotechnol. 2019 Nov 19;7:336. doi: 10.3389/fbioe.2019.00336 (PMC6877600; doi:10.3389/fbioe.2019.00336)
Supplement: Table S1 — List of various reactions and assigned rate laws used for kinetic modeling. [file Table_1.docx]

Table S1: List of various reactions and assigned rate laws used for kinetic modeling

|  | **Reaction name** | **Reaction** | **Rate Law** |
| --- | --- | --- | --- |
| 1 | HK | D-GLC -> GLC6P | Henri-Michaelis-Menten (irreversible) |
| 2 | PGI | GLC6P -> FR6P | Henri-Michaelis-Menten (irreversible) |
| 3 | PFK | FR6P -> FR16BP | Henri-Michaelis-Menten (irreversible) |
| 4 | FBPA | FR16BP -> GA3P + DHAP | Henri-Michaelis-Menten (irreversible) |
| 5 | GAPDH | GA3P -> BPG | Henri-Michaelis-Menten (irreversible) |
| 6 | PGK | BPG -> 3PGA | Henri-Michaelis-Menten (irreversible) |
| 7 | PGM | 3PGA -> 2PGA | Henri-Michaelis-Menten (irreversible) |
| 8 | Enolase | 2PGA -> PEP | Henri-Michaelis-Menten (irreversible) |
| 9 | PK | PEP -> PYR | Hill Cooperativity |
| 10 | MGO from DHAP | DHAP -> MGO | Mass action (irreversible) |
| 11 | MGO from GA3P | GA3P -> MGO | Mass action (irreversible) |
| 12 | TPI_GA3P | GA3P -> DHAP | Henri-Michaelis-Menten (irreversible) |
| 13 | TPI_DHAP | DHAP -> GA3P | Henri-Michaelis-Menten (irreversible) |
| 14 | SAMsyn | Met -> SAM | Hill Cooperativity |
| 15 | SAMdc | SAM -> dcSAM | Henri-Michaelis-Menten (irreversible) |
| 16 | Arginase | Arg -> Orn | Henri-Michaelis-Menten (irreversible) |
| 17 | ODC | Orn -> Put + CO_2_ | Henri-Michaelis-Menten (irreversible) |
| 18 | SpdS | Put + dcSAM -> Spd | Two substrate Henri-Michaelis-Menten |
| 19 | yECS | Glu + Cys -> GluCys | Two substrate Henri-Michaelis-Menten |
| 20 | GS | GluCys + Gly -> GSH | Two substrate Henri-Michaelis-Menten |
| 21 | TryS1 | Spd + GSH -> Gspd | Bi (irreversible) |
| 22 | TryS2 | Gspd + GSH -> T[SH]_2_ | Bi (irreversible) |
| 23 | TR | TS2 + NADPH -> T[SH]2 + NADP | Two substrate Henri-Michaelis-Menten |
| 24 | MGOtoHTA | MGO + T[SH]2 -> HTA | Mass action (irreversible) |
| 25 | HTAtoMGO | HTA -> MGO + T[SH]_2_ | Mass action (irreversible) |
| 26 | GLOI | HTA -> SDLTSH | Henri-Michaelis-Menten (irreversible) |
| 27 | GLOII | SDLTSH -> DL + T[SH]_2_ | Henri-Michaelis-Menten (irreversible) |
| 28 | Arg modification | Arg + MGO = Arg-MG + MGO^●-^ | Mass action (reversible) |
| 29 | Lys modification | Lys + MGO = Lys-MG + MGO^●-^ | Mass action (reversible) |
| 30 | TXNo Reduction | T[SH]_2_ + TXNo -> TXNr + TS_2_ | Mass action (irreversible) |
| 31 | TDPx | H_2_O_2_ + TXNr -> TXNo + H_2_O; TDPx | BiBiPingPong |
| 32 | TryP | H_2_O_2_ + TXNr -> TXNo + H_2_O; TryP | BiBiPingPong |
| 33 | Fenton reaction_ H_2_O_2_ | H_2_O_2_ + Fe^2^ -> .OH + Fe^3^ | Mass action (irreversible) |
| 34 | O_2_^●-^ dismutation | 2 * O_2_^●-^ + H+ -> H_2_O_2_ + O_2_ | Mass action (irreversible) |
| 35 | SOD | 2 * O_2_^●-^ -> H_2_O_2_ + O_2_ | Mass action (irreversible) |
| 36 | Lipid radical formation | ^●^OH + LH -> L^●^ + H_2_O | Mass action (irreversible) |
| 37 | Lipid peroxide radical formation | L^●^ + O_2_ -> LO_2_^●^ | Mass action (irreversible) |
| 38 | LOOH formation | LO_2_^●^ + LH -> LOOH + L. | Mass action (irreversible) |
| 39 | Fenton reaction for Lipid | LOOH + Fe^2^ -> LO_2_^●^ + Fe^3^ | Mass action (irreversible) |
| 40 | Nonradical formation | 2 * LO_2_^-^ -> MGO | Mass action (irreversible) |
| 41 | Nitric oxide synthase | Arg + NADPH -> NO. + Citrulline + NADP | Two substrate Henri-Michaelis-Menten |
| 42 | NO_2_^●^ formation | NO^●^ + O -> NO_2_^-^ | Mass action (irreversible) |
| 43 | ONOOH formation | NO_2_^-^ + ^●^OH -> ONOOH | Mass action (irreversible) |
| 44 | NO_2_^●^ from ONOOH | ONOOH + H_2_O_2_ -> O_2_^●-^ + NO_2_^●^ + H_2_O + H+ | Mass action (irreversible) |
| 45 | ONOO^-^ formation | NO_2_^●^ + O_2_^●^- -> ONOO^-^ | Mass action (irreversible) |
| 46 | TDPX reduction | TXNr + TDPxo -> TXNo + TDPxr | Mass action (irreversible) |
| 47 | TDPx for NO_2_^-^ | TDPxr + ONOO^-^ -> TDPxo + NO_2_^-^ | Mass action (irreversible) |
| 48 | TXN for NO_2_^-^ | TXNr + ONOO^-^ -> TXNo + NO_2_^-^ | Mass action (irreversible) |
| 49 | T[SH]_2_ for NO_2_^-^ | T[SH]_2_ + ONOO^-^ -> TS2 + NO_2_^-^ | Mass action (irreversible) |
| 50 | Lipid radical from NO_2_^●^ | NO_2_^●^ + LH -> NO_2_^-^ + L^●^ + H+ | Mass action (irreversible) |
| 51 | O_2_^●-^ formation from MGO^●-^ | MGO^●-^ + O_2_ -> MGO + O_2_^●-^ | Mass action (irreversible) |
| **52** | **GLOI inhibition** | **HTA -> SDLTSH; BBGS** | **Competitive inhibition (irr)** |
| **53** | **GLOI inhibition** | **HTA -> SDLTSH; DNPGS** | **Competitive inhibition (irr)** |
