# Supplementary material for: Integrative Computational Framework for Understanding Metabolic Modulation in Leishmania
Source: Front Bioeng Biotechnol. 2019 Nov 19;7:336. doi: 10.3389/fbioe.2019.00336 (PMC6877600; doi:10.3389/fbioe.2019.00336)
Supplement: Table S2 — Kinetic parameters and initial concentration of metabolites used in the kinetic model system. [file Table_2.docx]

**Table S2. Kinetic parameters and initial concentration of metabolites used in the kinetic model system.**

| **Reaction/ Enzyme name** | **Initial concentration (M)** | **k1**  **(s-1)** | **k-1 (s-1)** | **k2**  **(M-1.s-1)** | **Km**  **(M)** | **V**  **(M.s-1.mg-1)** | **s0.5 (M)** | **n**  **(Hill coefficient)** | **Kd (M)** | **Kcat** |
| --- | --- | --- | --- | --- | --- | --- | --- | --- | --- | --- |
| TXN reduction |  |  |  | 2.00E+02 |  |  |  |  |  |  |
| TDPx | 28E-6 (TXNr) |  |  |  | 2.2E-6 (txn) 193E-6 (H2O2) |  |  |  |  | 15.4 |
| TryP | 28E-6 (TXNr) |  |  |  | 4.9+-0.6 (TXN) 6.3+-0.8 (H_2_O_2_) |  |  |  |  | 8.8 |
| Fenton Reaction | 8.8E-9 (Fe^+2^) |  |  | 7.60E+01 |  |  |  |  |  |  |
| O2.- formation from MGO.- | 1E-9 (O) |  |  | 1.31E-10 |  |  |  |  |  |  |
| O2.- dismutation |  |  |  | 5x10E+5 |  |  |  |  |  |  |
| SOD |  |  |  | 1.6-1.8x10E+9 |  |  |  |  |  |  |
| NOS |  |  |  |  | 4.9E-6 (Arg) 0.7E-6 (NADPH) | 3.50E-06 |  |  |  |  |
| NO2. formation | 1E-9 (O) |  |  | 3.5x10E+9 |  |  |  |  |  |  |
| ONOOH formation |  |  |  | 1.4x10E+10 |  |  |  |  |  |  |
| NO2. formation |  |  |  | 1x10E+5 |  |  |  |  |  |  |
| ONOO- formation |  |  |  | 3.4x10E+7 to 7x10E+9 |  |  |  |  |  |  |
| TDPx reduction | 2E-7 (TDPx) |  |  | 2.2x10E+5 |  |  |  |  |  |  |
| TDPx for NO^2-^ |  |  |  | 9x10E+5 |  |  |  |  |  |  |
| TXN for NO^2-^ |  |  |  | 3.5x10E+3 |  |  |  |  |  |  |
| T[SH]2 for NO^2-^ |  |  |  | 7.2x10E+3 |  |  |  |  |  |  |
| Lipid radical | 0.025 (LH) |  |  | 1.00E+10 |  |  |  |  |  |  |
| Lipid peroxide radical | 0.00E+00 |  |  | 3x10E+8 |  |  |  |  |  |  |
| LOOH formation |  |  |  | 5.00E+01 |  |  |  |  |  |  |
|  |  |  |  |  |  |  |  |  |  |  |
| Lipid radical from NO_2_^●^ |  |  |  | 1.00E+06 |  |  |  |  |  |  |
|  |  |  |  |  |  |  |  |  |  |  |
| Fenton Reaction |  |  |  | 1.00E+04 |  |  |  |  |  |  |
| MGO synthesis |  |  |  | 10E+6-7 |  |  |  |  |  |  |
| GSH1 (y-ECS) | 1e-05 (Cys) 1e-05 (Glu) |  |  |  | 9.2E-3 (Glu) 1.7E-3 (Cys) | 1.80E-06 |  |  | 9.37E-05 |  |
| GSH2 (GS) | 1e-05 (Gly) |  |  |  | 4E-5 (yEC) 1.2E-3 (Gly) | 3.40E-08 |  |  | 8.00E-05 |  |
| TR | 5e-05 (TS2 and NADPH) |  |  |  | 50E-6 (TS2) 20E-6 (NADPH) | 5E-5 (TS2) 2E-5 (NADPH) |  |  |  |  |
| Arginase |  |  |  |  | 0.0215 | 2.40E-03 |  |  |  |  |
| ODC |  |  |  |  | 4.20E-04 | 2.53E-02  (calculated) |  |  |  | 7.7 +- 0.2 |
| SAMsyn (MAT) | 1.00E-05 |  |  |  |  | 3.40E-09 | 2.50E-04 | 2.3 |  | 0.32 |
| SAMdc (AdoMetDC) |  |  |  |  | 0.00038 | 5.20E-09 |  |  |  | 0.0013+- 0.0004 |
| SpdS |  |  |  |  | 205E-6 (Put) 0.09E-6 (dcSAM) | 1.98E-13 |  |  |  |  |
| TryS1 |  |  |  |  | 940E-6 (Spd) 89E-6 (GSH) | 8E-07 (calculated) |  |  |  | 2 |
| TryS2 |  |  |  |  | 40E-6 (Gspd) 89E-6 (GSH) |  |  |  |  | 2 |
| MGO to HTA | 9.00E-01 |  |  | 5.60E-03 |  |  |  |  |  |  |
| HTA to MGO |  | 0.016 |  |  |  |  |  |  |  |  |
| GLOI |  |  |  |  | 3.20E-05 | 1.59E-4 (calculated) |  |  |  | 800 |
| GLOII |  |  |  |  | 3.90E-05 | 2.30E-09 |  |  |  |  |
| Arginine modification | 2.00E-03 |  | 4.40E-06 | 8.50E-03 |  |  |  |  |  |  |
| Lysine modification | 2.00E-03 |  | 5.00E-04 | 6.80E-03 |  |  |  |  |  |  |
| HK | 1.70E-05 |  |  |  | 6.60E-08 | 4.30E-07 |  |  |  |  |
| PGI |  |  |  |  | 1.43E-04 | 8.33E-06 |  |  |  |  |
| PFK | 3.2E-4 (AMP) |  |  |  | 5.00E-04 | 7.20E-09 |  |  |  |  |
| FBPA |  |  |  |  | 4.90E-05 | 6.20E-07 |  |  |  | 8.2 |
| GAPDH |  |  |  |  | 1.65E-04 | 2.55E-06 |  |  |  |  |
| PGK |  |  |  |  | 2.02E-03 | 1.65E-04 |  |  |  |  |
| PGM |  |  |  |  | 0.00027 | 1.67E-9 (calculated) |  |  |  | 434 ± 54 |
| Enolase |  |  |  |  | 5.10E-05 | 1.34E-06 |  |  |  |  |
| PK |  |  |  |  |  | 8.20E-06 | 1.16E-03 | 1.56±0.17 |  |  |
|  |  |  |  |  |  |  |  |  |  |  |
| TPI |  |  |  |  | 0.26mM [2.6e-4M GAP] 0.9 mM [9e-3M DHAP] |  | 15.88e-12Ms-1 (GAP) 28.70e-11 Ms-1 (DHAP) calculated |  |  | 3570s-1 (GAP)  645s-1 (DHAP) |
