# Supplementary material for: Integrative Computational Framework for Understanding Metabolic Modulation in Leishmania
Source: Front Bioeng Biotechnol. 2019 Nov 19;7:336. doi: 10.3389/fbioe.2019.00336 (PMC6877600; doi:10.3389/fbioe.2019.00336)
Supplement: Table S3 — Topological properties of the Master model. [file Table_3.docx]

**Table S3.** Topological properties of the Master model

| **Name** | **Betweenness centrality** | **Closeness centrality** | **Clustering coefficient** | **Edge**  **count** | **In degree** | **Neighborhood connectivity** | **Out degree** | **Forman curvature of nodes** |
| --- | --- | --- | --- | --- | --- | --- | --- | --- |
| 'pyr' | 0.086 | 0.103 | 0 | 6 | 4 | 3.4 | 2 | -6.858 |
| 'hta' | 0.105 | 0.115 | 0 | 5 | 2 | 3.333 | 3 | -5.709 |
| 'acoa' | 0.108 | 0.113 | 0 | 8 | 2 | 4.375 | 6 | -4.971 |
| 'mg' | 0.081 | 0.119 | 0 | 7 | 3 | 2.75 | 4 | -4.971 |
| 't[sh]2' | 0.097 | 0.103 | 0.083 | 5 | 4 | 3 | 1 | -4.020 |
| 'pa' | 0.030 | 0.284 | 0.167 | 5 | 3 | 3.75 | 2 | -3.692 |
| 'occoa' | 0 | 0.333 | 0.333 | 3 | 2 | 6.33 | 1 | -3.692 |
| 'g3p' | 0.032 | 0.198 | 0 | 5 | 4 | 2.8 | 1 | -3.314 |
| 'ga3p' | 0.056 | 0.119 | 0 | 4 | 2 | 3 | 2 | -3.217 |
| 'dhap' | 0.031 | 0.110 | 0 | 4 | 2 | 4 | 2 | -3.217 |
| 'pe' | 0.017 | 0.233 | 0 | 5 | 2 | 3 | 3 | -3.217 |
| 'glu' | 0.048 | 0.110 | 0.014 | 9 | 1 | 2.889 | 8 | -3.180 |
| 'ump' | 0.002 | 1 | 0 | 5 | 4 | 1.8 | 1 | -3.121 |
| 'ala' | 0.009 | 0.093 | 0 | 3 | 2 | 4 | 1 | -2.955 |
| 'malcoa' | 0.022 | 0.824 | 0.045 | 13 | 2 | 3.231 | 11 | -2.953 |
| 'gsh' | 0.054 | 0.095 | 0.05 | 5 | 3 | 3 | 2 | -2.838 |
| 'cys' | 0.035 | 0.081 | 0.05 | 5 | 2 | 2.8 | 3 | -2.838 |
| 'mag' | 0.002 | 0.288 | 0.167 | 4 | 1 | 4 | 3 | -2.485 |
| 'akg' | 0.066 | 0.112 | 0 | 4 | 2 | 4 | 2 | -2.363 |
| 'gspd' | 0.036 | 0.094 | 0.167 | 3 | 2 | 4 | 1 | -2.363 |
| '12dgr' | 0.024 | 0.350 | 0.067 | 8 | 3 | 3.333 | 5 | -2.363 |
| 'prpp' | 0.006 | 0.667 | 0 | 4 | 2 | 3 | 2 | -2.363 |
| 'imp' | 0 | 0.6 | 0.167 | 3 | 2 | 2.67 | 1 | -2.363 |
| 'mal' | 0.029 | 0.094 | 0 | 3 | 1 | 4 | 2 | -2.293 |
| 'glycerol' | 0 | 0.172 | 0 | 2 | 1 | 4 | 1 | -2.293 |
| 'g-glycys' | 0.024 | 0.087 | 0 | 3 | 2 | 6.333 | 1 | -2.010 |
| 'farnasylpp' | 0.020 | 0.171 | 0.167 | 3 | 2 | 3 | 1 | -2.010 |
| 'man1p' | 0 | 0.667 | 0 | 2 | 1 | 2.5 | 1 | -2.010 |
| 'dccoa' | 0 | 0.4 | 0.333 | 3 | 2 | 6.33 | 1 | -2.010 |
| 'acdcoa' | 0 | 0.667 | 0 | 2 | 1 | 3 | 1 | -2.010 |
| 'tdcoa' | 0 | 0.667 | 0.333 | 3 | 2 | 6.33 | 1 | -2.010 |
| 'hxcoa' | 0 | 0.286 | 0.167 | 3 | 2 | 5.67 | 1 | -2.010 |
| 'b-D-glc' | 0 | 0.090 | 0 | 1 | 0 | 3 | 1 | -2.010 |
| '1ag3p' | 0.031 | 0.233 | 0.167 | 3 | 2 | 4 | 1 | -1.708 |
| 'ser' | 0.018 | 0.088 | 0.05 | 5 | 1 | 3 | 4 | -1.708 |
| 'spd' | 0.034 | 0.087 | 0 | 3 | 2 | 2.333 | 1 | -1.657 |
| 'D-lacgsh' | 0.031 | 0.086 | 0 | 2 | 1 | 2.5 | 1 | -1.657 |
| 'hmgcoa' | 0.029 | 0.123 | 0 | 4 | 3 | 3.25 | 1 | -1.657 |
| 'b-D-fr16p' | 0.011 | 0.110 | 0 | 3 | 1 | 3.333 | 2 | -1.657 |
| 'ort' | 0.011 | 0.364 | 0 | 3 | 2 | 3.333 | 1 | -1.657 |
| 'ortdn5p' | 0.010 | 0.5 | 0 | 3 | 1 | 4 | 2 | -1.657 |
| '1g3pe' | 0.004 | 0.169 | 0 | 3 | 1 | 4 | 2 | -1.657 |
| 'cdpdag' | 0.003 | 1 | 0 | 3 | 1 | 2.667 | 2 | -1.657 |
| 'thf' | 0.003 | 0.667 | 0 | 3 | 2 | 2.333 | 1 | -1.657 |
| 'eictrcoa' | 0.002 | 0.5 | 0 | 3 | 2 | 6 | 1 | -1.657 |
| 'dcopcoa' | 0.002 | 1 | 0.167 | 3 | 2 | 5.667 | 1 | -1.657 |
| 'dcotcoa' | 0.002 | 1 | 0 | 3 | 2 | 5.333 | 1 | -1.657 |
| 'man6p' | 0 | 0.5 | 0 | 3 | 2 | 2.33 | 1 | -1.657 |
| 'geranylpp' | 0 | 0.157 | 0.333 | 3 | 2 | 3 | 1 | -1.657 |
| 'mannan' | 0 | 0 | 0 | 1 | 1 | 2 | 0 | -1.657 |
| 'ipdp' | 0.024 | 0.176 | 0.167 | 4 | 1 | 2.5 | 3 | -1.464 |
| 'gly' | 0.008 | 0.088 | 0 | 3 | 1 | 4.333 | 2 | -1.464 |
| 'L-lactald' | 0.001 | 1 | 0 | 2 | 1 | 2.5 | 1 | -1.464 |
| 'mev' | 0.028 | 0.132 | 0 | 2 | 1 | 3 | 1 | -1.182 |
| 'adomet' | 0.022 | 0.075 | 0 | 2 | 1 | 2.5 | 1 | -1.182 |
| 'sql' | 0.019 | 0.190 | 0 | 2 | 1 | 2.5 | 1 | -1.182 |
| 'lanostr' | 0.015 | 0.243 | 0 | 3 | 1 | 2 | 2 | -1.182 |
| '3mop' | 0.013 | 0.070 | 0 | 2 | 1 | 2.5 | 1 | -1.182 |
| 'hcys' | 0.012 | 0.068 | 0 | 3 | 1 | 2.667 | 2 | -1.182 |
| '3mob' | 0.010 | 0.133 | 0 | 2 | 1 | 2.5 | 1 | -1.182 |
| 'cysthion' | 0.010 | 0.064 | 0 | 2 | 1 | 4 | 1 | -1.182 |
| 'dhort' | 0.009 | 0.084 | 0 | 3 | 1 | 3.333 | 2 | -1.182 |
| 'b-D-fr6p' | 0.009 | 0.102 | 0 | 4 | 2 | 2.5 | 2 | -1.182 |
| 'mthf' | 0.009 | 0.066 | 0 | 2 | 1 | 3 | 1 | -1.182 |
| 'mmpe' | 0.008 | 0.146 | 0 | 2 | 1 | 3.5 | 1 | -1.182 |
| '4mop' | 0.007 | 0.091 | 0 | 2 | 1 | 2.5 | 1 | -1.182 |
| 'pep' | 0.006 | 0.094 | 0 | 2 | 1 | 3.5 | 1 | -1.182 |
| 'amlplprot' | 0.006 | 0.063 | 0.167 | 3 | 1 | 2 | 2 | -1.182 |
| 'pc' | 0.006 | 0.175 | 0 | 3 | 1 | 2.333 | 2 | -1.182 |
| '1g3pc' | 0.004 | 0.183 | 0 | 3 | 1 | 3.333 | 2 | -1.182 |
| 'g3pc' | 0.003 | 0.194 | 0 | 3 | 1 | 3 | 2 | -1.182 |
| 'g3pe' | 0.003 | 0.184 | 0 | 3 | 1 | 3 | 2 | -1.182 |
| 'tyr' | 0.001 | 1 | 0.167 | 3 | 2 | 4.333 | 1 | -1.182 |
| 'ribl5p' | 0.001 | 0.4 | 0 | 3 | 1 | 2.67 | 2 | -1.182 |
| 'r5p' | 0.001 | 0.5 | 0 | 3 | 1 | 3.67 | 2 | -1.182 |
| 'glincoa' | 0.001 | 0.36 | 0 | 3 | 1 | 2.33 | 2 | -1.182 |
| 'eictcoa' | 0 | 0.5 | 0 | 2 | 1 | 2.5 | 1 | -1.182 |
| 'xyl5p' | 0 | 0.75 | 0.167 | 3 | 1 | 3.67 | 2 | -1.182 |
| 'ddcoa' | 0 | 0.5 | 0.333 | 3 | 2 | 6.33 | 1 | -1.182 |
| 'eicpcoa' | 0 | 0.667 | 0.167 | 3 | 2 | 6 | 1 | -1.182 |
| 'xmp' | 0 | 0.75 | 0.083 | 4 | 2 | 2.25 | 2 | -1.182 |
| 'gdpman' | 0 | 1 | 0 | 2 | 1 | 1.5 | 1 | -1.182 |
| 'dhlplprot' | 0 | 0.056 | 0.5 | 2 | 1 | 2.5 | 1 | -1.182 |
| 'oaa' | 0 | 0 | 0 | 3 | 3 | 5.33 | 0 | -1.182 |
| 'sdhpt7p' | 0 | 0 | 0.17 | 4 | 4 | 3 | 0 | -1.182 |
| 'erth4p' | 0 | 0.75 | 0.5 | 2 | 0 | 4 | 2 | -1.182 |
| '3pga' | 0.056 | 0.120 | 0 | 3 | 1 | 2.333 | 2 | -0.828 |
| 'bpg' | 0.054 | 0.108 | 0 | 2 | 1 | 3 | 1 | -0.828 |
| '3phpyr' | 0.051 | 0.115 | 0 | 3 | 1 | 3 | 2 | -0.828 |
| 'oacser' | 0.033 | 0.075 | 0.167 | 3 | 2 | 6 | 1 | -0.828 |
| 'D-lac' | 0.032 | 0.094 | 0 | 2 | 1 | 3.5 | 1 | -0.828 |
| 'isocit' | 0.029 | 0.101 | 0 | 2 | 1 | 3 | 1 | -0.828 |
| 'cisaco' | 0.028 | 0.092 | 0 | 2 | 1 | 2 | 1 | -0.828 |
| 'fum' | 0.027 | 0.086 | 0 | 4 | 3 | 3 | 1 | -0.828 |
| 'mev5p' | 0.027 | 0.144 | 0 | 2 | 1 | 2 | 1 | -0.828 |
| 'cit' | 0.026 | 0.085 | 0 | 2 | 1 | 5 | 1 | -0.828 |
| 'mev5pp' | 0.025 | 0.158 | 0 | 2 | 1 | 3 | 1 | -0.828 |
| 'adometam' | 0.024 | 0.080 | 0 | 2 | 1 | 2.5 | 1 | -0.828 |
| '2maacoa' | 0.021 | 0.103 | 0 | 3 | 1 | 3.667 | 2 | -0.828 |
| 'met' | 0.021 | 0.070 | 0 | 3 | 2 | 2.333 | 1 | -0.828 |
| 'suc' | 0.021 | 0.084 | 0 | 4 | 1 | 2.75 | 3 | -0.828 |
| '3hmbcoa' | 0.019 | 0.095 | 0 | 2 | 1 | 2.5 | 1 | -0.828 |
| 'succoa' | 0.019 | 0.078 | 0 | 2 | 1 | 4 | 1 | -0.828 |
| '2mbecoa' | 0.018 | 0.087 | 0 | 2 | 1 | 2 | 1 | -0.828 |
| 'sql23epx' | 0.017 | 0.213 | 0 | 2 | 1 | 2.5 | 1 | -0.828 |
| '3pser' | 0.017 | 0.081 | 0 | 2 | 1 | 4 | 1 | -0.828 |
| '2mbcoa' | 0.016 | 0.081 | 0 | 2 | 1 | 2 | 1 | -0.828 |
| '2mbutdhla' | 0.015 | 0.075 | 0 | 2 | 1 | 2 | 1 | -0.828 |
| '44mctr' | 0.012 | 0.25 | 0 | 2 | 1 | 2.5 | 1 | -0.828 |
| 'ile' | 0.012 | 0.066 | 0 | 3 | 2 | 4 | 1 | -0.828 |
| 'val' | 0.011 | 0.122 | 0 | 3 | 2 | 4 | 1 | -0.828 |
| '14dmls' | 0.011 | 0.286 | 0 | 2 | 1 | 2 | 1 | -0.828 |
| '2mpdhla' | 0.009 | 0.147 | 0 | 2 | 1 | 2 | 1 | -0.828 |
| 'zym' | 0.009 | 0.333 | 0 | 2 | 1 | 2 | 1 | -0.828 |
| 'ibcoa' | 0.008 | 0.164 | 0 | 2 | 1 | 2 | 1 | -0.828 |
| 'carblasp' | 0.008 | 0.078 | 0 | 3 | 2 | 3.333 | 1 | -0.828 |
| 'macoa' | 0.007 | 0.186 | 0 | 2 | 1 | 2 | 1 | -0.828 |
| 'fecostr' | 0.007 | 0.4 | 0 | 2 | 1 | 2 | 1 | -0.828 |
| 'dimpe' | 0.007 | 0.159 | 0 | 2 | 1 | 2.5 | 1 | -0.828 |
| '3hibcoa' | 0.006 | 0.217 | 0 | 2 | 1 | 2 | 1 | -0.828 |
| '3mbutdhla' | 0.006 | 0.095 | 0 | 2 | 1 | 2 | 1 | -0.828 |
| 'carbp' | 0.006 | 0.073 | 0 | 2 | 1 | 2.5 | 1 | -0.828 |
| 'l-kyn' | 0.006 | 0.087 | 0 | 3 | 1 | 2.333 | 2 | -0.828 |
| 'put' | 0.006 | 0.080 | 0 | 2 | 1 | 2.5 | 1 | -0.828 |
| 'epistr' | 0.005 | 0.5 | 0 | 2 | 1 | 2 | 1 | -0.828 |
| '3hib' | 0.005 | 0.262 | 0 | 2 | 1 | 2 | 1 | -0.828 |
| 'ivcoa' | 0.005 | 0.101 | 0 | 2 | 1 | 2 | 1 | -0.828 |
| '2pga' | 0.005 | 0.087 | 0 | 2 | 1 | 2.5 | 1 | -0.828 |
| 'gln' | 0.004 | 0.068 | 0 | 2 | 1 | 5.5 | 1 | -0.828 |
| 'mmsald' | 0.004 | 0.333 | 0 | 2 | 1 | 2 | 1 | -0.828 |
| '3mb2coa' | 0.004 | 0.107 | 0 | 2 | 1 | 2 | 1 | -0.828 |
| 'ergsttr' | 0.004 | 0.667 | 0 | 2 | 1 | 2 | 1 | -0.828 |
| 'lplprot' | 0.003 | 0.059 | 0.5 | 2 | 1 | 2.5 | 1 | -0.828 |
| '3mgcoa' | 0.003 | 0.114 | 0 | 2 | 1 | 3 | 1 | -0.828 |
| 'mmalonat' | 0.003 | 0.469 | 0 | 2 | 1 | 7.5 | 1 | -0.828 |
| 'ergsttet' | 0.002 | 1 | 0 | 2 | 1 | 1.5 | 1 | -0.828 |
| 'dgdp' | 0.002 | 1 | 0 | 3 | 2 | 2.667 | 1 | -0.828 |
| 'dadp' | 0.002 | 1 | 0 | 3 | 2 | 2.667 | 1 | -0.828 |
| 'thfglu' | 0.001 | 1 | 0 | 2 | 1 | 2 | 1 | -0.828 |
| '1pl5c' | 0.001 | 1 | 0 | 2 | 1 | 5 | 1 | -0.828 |
| 'gl6p' | 0.001 | 0.306 | 0 | 2 | 1 | 2.5 | 1 | -0.828 |
| 'g15l6p' | 0 | 0.25 | 0 | 2 | 1 | 2.5 | 1 | -0.828 |
| 'a-lincoa' | 0 | 0.286 | 0 | 2 | 1 | 2.5 | 1 | -0.828 |
| 'stdcoa' | 0 | 0.4 | 0 | 2 | 1 | 2.5 | 1 | -0.828 |
| 'lincoa' | 0 | 0.239 | 0 | 2 | 1 | 1.5 | 1 | -0.828 |
| 'amp' | 0 | 0.5 | 0.167 | 3 | 2 | 3.33 | 1 | -0.828 |
| 'f6p' | 0 | 1 | 0.167 | 4 | 2 | 2.5 | 2 | -0.828 |
| 'pmtcoa' | 0 | 1 | 0.333 | 3 | 2 | 6 | 1 | -0.828 |
| 'adp' | 0 | 0.462 | 0 | 3 | 1 | 2.33 | 2 | -0.828 |
| 'dhf' | 0 | 0.4 | 0 | 2 | 1 | 1.5 | 1 | -0.828 |
| 'cdp' | 0 | 0.75 | 0 | 3 | 1 | 1.67 | 2 | -0.828 |
| 'gdp' | 0 | 0.75 | 0 | 3 | 1 | 2 | 2 | -0.828 |
| 'uridine' | 0 | 0.667 | 0 | 2 | 1 | 3.5 | 1 | -0.828 |
| 'dcdp' | 0 | 1 | 0 | 2 | 1 | 2 | 1 | -0.828 |
| 'f16p' | 0 | 0 | 0 | 1 | 1 | 4 | 0 | -0.828 |
| 'ac' | 0 | 0 | 0 | 2 | 2 | 6 | 0 | -0.828 |
| 'leu' | 0.008 | 0.086 | 0 | 3 | 2 | 4 | 1 | 0 |
| 'b-D-glc6p' | 0.003 | 0.098 | 0 | 3 | 1 | 2.333 | 2 | 0 |
| 'nflk' | 0.003 | 0.081 | 0 | 3 | 1 | 2 | 2 | 0 |
| 'orn' | 0.003 | 0.075 | 0 | 2 | 1 | 2 | 1 | 0 |
| 'adesuc' | 0.003 | 0.080 | 0 | 2 | 1 | 4.5 | 1 | 0 |
| 'cdpe' | 0 | 0.196 | 0 | 2 | 1 | 3 | 1 | 0 |
| 'fol' | 0 | 0.5 | 0 | 2 | 1 | 2.5 | 1 | 0 |
| 'hser' | 0 | 0.667 | 0 | 2 | 1 | 2 | 1 | 0 |
| 'anthra' | 0 | 1 | 0 | 2 | 1 | 2 | 1 | 0 |
| 'aspsa' | 0 | 0.5 | 0 | 2 | 1 | 3.5 | 1 | 0 |
| 'phser' | 0 | 1 | 0 | 2 | 1 | 2.5 | 1 | 0 |
| 'dudp' | 0 | 0.667 | 0 | 2 | 1 | 3 | 1 | 0 |
| 'phe' | 0 | 0.667 | 0.17 | 3 | 2 | 4.33 | 1 | 0 |
| mannose' | 0 | 0.4 | 0 | 1 | 0 | 3 | 1 | 0 |
| fructose' | 0 | 0.09 | 0 | 1 | 0 | 4 | 1 | 0 |
| 'btcoa' | 0 | 0.25 | 0 | 1 | 0 | 3 | 1 | 0 |
| 'dc4pcoa' | 0 | 0 | 0 | 1 | 1 | 3 | 0 | 0 |
| 'dchxcoa' | 0 | 0 | 0 | 1 | 1 | 3 | 0 | 0 |
| 'strcoa' | 0 | 0 | 0.5 | 2 | 2 | 8 | 0 | 0 |
| 'olcoa' | 0 | 0.207 | 0 | 1 | 0 | 2 | 1 | 0 |
| 'dmpp' | 0 | 0.144 | 0.5 | 2 | 1 | 3.5 | 1 | 0 |
| 'acacylcoa' | 0 | 0.114 | 0 | 1 | 0 | 4 | 1 | 0 |
| 'ergstr' | 0 | 0 | 0 | 1 | 1 | 2 | 0 | 0 |
| 'formate' | 0 | 0 | 0 | 2 | 2 | 3 | 0 | 0 |
| 'clpn' | 0 | 0 | 0 | 2 | 2 | 2 | 0 | 0 |
| 'pg' | 0 | 1 | 0 | 1 | 0 | 2 | 1 | 0 |
| 'acylcoa' | 0 | 0.297 | 0.5 | 3 | 0 | 4.33 | 3 | 0 |
| 'mino' | 0 | 1 | 0 | 1 | 0 | 2 | 1 | 0 |
| 'ptd1ino' | 0 | 0 | 0 | 2 | 2 | 2 | 0 | 0 |
| 'fa' | 0 | 0 | 0.17 | 4 | 4 | 3.75 | 0 | 0 |
| 'tgr' | 0 | 0 | 0 | 1 | 1 | 6 | 0 | 0 |
| 'peth' | 0 | 0.170 | 0 | 1 | 0 | 2 | 1 | 0 |
| 'ethan' | 0 | 0 | 0 | 1 | 1 | 3 | 0 | 0 |
| 'carboxylate' | 0 | 0 | 0 | 2 | 2 | 4 | 0 | 0 |
| 'chol' | 0 | 0 | 0 | 1 | 1 | 3 | 0 | 0 |
| 'cytidine' | 0 | 0.571 | 0 | 2 | 0 | 2 | 2 | 0 |
| 'hxan' | 0 | 0.444 | 0 | 1 | 0 | 3 | 1 | 0 |
| 'xan' | 0 | 0.5 | 0 | 1 | 0 | 4 | 1 | 0 |
| 'asp' | 0 | 0.081 | 0 | 5 | 0 | 2.2 | 5 | 0 |
| 'cmp' | 0 | 0 | 0 | 2 | 2 | 2.5 | 0 | 0 |
| 'ctp' | 0 | 0.5 | 0 | 1 | 0 | 3 | 1 | 0 |
| 'gmp' | 0 | 0 | 0 | 2 | 2 | 3.5 | 0 | 0 |
| 'dgmp' | 0 | 0 | 0 | 1 | 1 | 3 | 0 | 0 |
| 'damp' | 0 | 0 | 0 | 1 | 1 | 3 | 0 | 0 |
| 'dcmp' | 0 | 0 | 0 | 1 | 1 | 2 | 0 | 0 |
| 'dtmp' | 0 | 0 | 0 | 1 | 1 | 5 | 0 | 0 |
| 'gtp' | 0 | 0.5 | 0 | 1 | 0 | 3 | 1 | 0 |
| 'atp' | 0 | 0.35 | 0 | 1 | 0 | 3 | 1 | 0 |
| 'udp' | 0 | 0.667 | 0 | 1 | 0 | 5 | 1 | 0 |
| 'dutp' | 0 | 0.5 | 0 | 1 | 0 | 2 | 1 | 0 |
| 'arg' | 0 | 0.070 | 0 | 2 | 0 | 1.5 | 2 | 0 |
| 'urea' | 0 | 0 | 0 | 1 | 1 | 2 | 0 | 0 |
| 'ts2' | 0 | 0.094 | 0 | 1 | 0 | 4 | 1 | 0 |
| 'L-lac' | 0 | 0 | 0 | 1 | 1 | 2 | 0 | 0 |
| 'pro' | 0 | 0 | 0 | 1 | 1 | 2 | 0 | 0 |
| 'acald' | 0 | 1 | 0 | 1 | 0 | 3 | 1 | 0 |
| 'thr' | 0 | 0 | 0 | 3 | 3 | 2 | 0 | 0 |
| 'asn' | 0 | 0 | 0 | 1 | 1 | 5 | 0 | 0 |
| 'trp' | 0 | 0.076 | 0 | 1 | 0 | 3 | 1 | 0 |
| 'dhpt' | 0 | 0.333 | 0 | 1 | 0 | 2 | 1 | 0 |
| 'thfpolyglu' | 0 | 0 | 0 | 1 | 1 | 2 | 0 | 0 |
| 'phepyr' | 0 | 0.5 | 0 | 1 | 0 | 3 | 1 | 0 |
| '34hpp' | 0 | 0 | 0 | 1 | 1 | 3 | 0 | 0 |
| '4m2opent' | 0 | 0.0823 | 0 | 1 | 0 | 3 | 1 | 0 |
| '2oivlrt' | 0 | 0.1132 | 0 | 1 | 0 | 3 | 1 | 0 |
| '3m2opent' | 0 | 0.0623 | 0 | 1 | 0 | 3 | 1 | 0 |
| 'ppcoa' | 0 | 0 | 0 | 1 | 1 | 3 | 0 | 0 |
| '3mercpyr' | 0 | 0 | 0 | 1 | 1 | 5 | 0 | 0 |
| '42aphdob' | 0 | 0 | 0 | 1 | 1 | 2 | 0 | 0 |
