# Supplementary material for: Integrative Computational Framework for Understanding Metabolic Modulation in Leishmania
Source: Front Bioeng Biotechnol. 2019 Nov 19;7:336. doi: 10.3389/fbioe.2019.00336 (PMC6877600; doi:10.3389/fbioe.2019.00336)
Supplement: Supplementary file 11 [file Data_Sheet_3.PDF]

```
<?xml version="1.0" encoding="UTF-8"?>
<sbml xmlns="http://www.sbml.org/sbml/level2/version4"
xmlns:celldesigner="http://www.sbml.org/2001/ns/celldesigner" level="2" version="4">
<model metaid="untitled" id="untitled">
<annotation>
<celldesigner:extension>
<celldesigner:modelVersion>4.0</celldesigner:modelVersion>
<celldesigner:modelDisplay sizeX="6020" sizeY="4020"/>
<celldesigner:listOfCompartmentAliases/>
<celldesigner:listOfComplexSpeciesAliases/>
<celldesigner:listOfSpeciesAliases>
<celldesigner:speciesAlias id="sa3" species="s52">
<celldesigner:activity>inactive</celldesigner:activity>
<celldesigner:bounds x="269.625" y="17.0" w="70.0" h="25.0"/>
<celldesigner:font size="12"/>
<celldesigner:view state="usual"/>
<celldesigner:usualView>
<celldesigner:innerPosition x="0.0" y="0.0"/>
<celldesigner:boxSize width="70.0" height="25.0"/>
<celldesigner:singleLine width="1.0"/>
<celldesigner:paint color="ffccff66" scheme="Color"/>
</celldesigner:usualView>
<celldesigner:briefView>
<celldesigner:innerPosition x="0.0" y="0.0"/>
<celldesigner:boxSize width="80.0" height="60.0"/>
<celldesigner:singleLine width="1.0"/>
<celldesigner:paint color="3fff0000" scheme="Color"/>
</celldesigner:briefView>
```

<celldesigner:info state="empty" angle="-1.5707963267948966"/>

</celldesigner:speciesAlias>

<celldesigner:speciesAlias id="sa17" species="s84">

<celldesigner:activity>inactive</celldesigner:activity>

<celldesigner:bounds x="13.5" y="148.75" w="45.0" h="22.5"/>

<celldesigner:font size="12"/>

<celldesigner:view state="usual"/>

<celldesigner:usualView>

<celldesigner:innerPosition x="0.0" y="0.0"/>

<celldesigner:boxSize width="45.0" height="22.5"/>

<celldesigner:singleLine width="1.0"/>

<celldesigner:paint color="ffccff66" scheme="Color"/>

</celldesigner:usualView>

<celldesigner:briefView>

<celldesigner:innerPosition x="0.0" y="0.0"/>

<celldesigner:boxSize width="80.0" height="60.0"/>

<celldesigner:singleLine width="1.0"/>

<celldesigner:paint color="3fff0000" scheme="Color"/>

</celldesigner:briefView>

<celldesigner:info state="empty" angle="-1.5707963267948966"/>

</celldesigner:speciesAlias>

<celldesigner:speciesAlias id="sa18" species="s83">

<celldesigner:activity>inactive</celldesigner:activity>

<celldesigner:bounds x="93.5" y="148.75" w="45.0" h="22.5"/>

<celldesigner:font size="12"/>

<celldesigner:view state="usual"/>

<celldesigner:usualView>

<celldesigner:innerPosition x="0.0" y="0.0"/>

<celldesigner:boxSize width="45.0" height="22.5"/>

<celldesigner:singleLine width="1.0"/>

<celldesigner:paint color="ffccff66" scheme="Color"/>

</celldesigner:usualView>

<celldesigner:briefView>

<celldesigner:innerPosition x="0.0" y="0.0"/>

<celldesigner:boxSize width="80.0" height="60.0"/>

<celldesigner:singleLine width="1.0"/>

<celldesigner:paint color="3fff0000" scheme="Color"/>

</celldesigner:briefView>

<celldesigner:info state="empty" angle="-1.5707963267948966"/>

</celldesigner:speciesAlias>

<celldesigner:speciesAlias id="sa19" species="s82">

<celldesigner:activity>inactive</celldesigner:activity>

<celldesigner:bounds x="193.5" y="148.75" w="45.0" h="22.5"/>

<celldesigner:font size="12"/>

<celldesigner:view state="usual"/>

<celldesigner:usualView>

<celldesigner:innerPosition x="0.0" y="0.0"/>

<celldesigner:boxSize width="45.0" height="22.5"/>

<celldesigner:singleLine width="1.0"/>

<celldesigner:paint color="ffccff66" scheme="Color"/>

</celldesigner:usualView>

<celldesigner:briefView>

<celldesigner:innerPosition x="0.0" y="0.0"/>

<celldesigner:boxSize width="80.0" height="60.0"/>

<celldesigner:singleLine width="1.0"/>

<celldesigner:paint color="3fff0000" scheme="Color"/>

</celldesigner:briefView>

<celldesigner:info state="empty" angle="-1.5707963267948966"/>

</celldesigner:speciesAlias>

<celldesigner:speciesAlias id="sa22" species="s57">

<celldesigner:activity>inactive</celldesigner:activity>

<celldesigner:bounds x="269.625" y="147.0" w="70.0" h="25.0"/>

<celldesigner:font size="12"/>

<celldesigner:view state="usual"/>

<celldesigner:usualView>

<celldesigner:innerPosition x="0.0" y="0.0"/>

<celldesigner:boxSize width="70.0" height="25.0"/>

<celldesigner:singleLine width="1.0"/>

<celldesigner:paint color="ffccff66" scheme="Color"/>

</celldesigner:usualView>

<celldesigner:briefView>

<celldesigner:innerPosition x="0.0" y="0.0"/>

<celldesigner:boxSize width="80.0" height="60.0"/>

<celldesigner:singleLine width="1.0"/>

<celldesigner:paint color="3fff0000" scheme="Color"/>

</celldesigner:briefView>

<celldesigner:info state="empty" angle="-1.5707963267948966"/>

</celldesigner:speciesAlias>

<celldesigner:speciesAlias id="sa23" species="s54">

<celldesigner:activity>inactive</celldesigner:activity>

<celldesigner:bounds x="271.0" y="77.5" w="70.0" h="25.0"/>

<celldesigner:font size="12"/>

<celldesigner:view state="usual"/>

<celldesigner:usualView>

<celldesigner:innerPosition x="0.0" y="0.0"/>

<celldesigner:boxSize width="70.0" height="25.0"/>

<celldesigner:singleLine width="1.0"/>

<celldesigner:paint color="ffccff66" scheme="Color"/>

</celldesigner:usualView>

<celldesigner:briefView>

<celldesigner:innerPosition x="0.0" y="0.0"/>

<celldesigner:boxSize width="80.0" height="60.0"/>

<celldesigner:singleLine width="1.0"/>

<celldesigner:paint color="3fff0000" scheme="Color"/>

</celldesigner:briefView>

<celldesigner:info state="empty" angle="-1.5707963267948966"/>

</celldesigner:speciesAlias>

<celldesigner:speciesAlias id="sa30" species="s85">

<celldesigner:activity>inactive</celldesigner:activity>

<celldesigner:bounds x="13.5" y="238.75" w="45.0" h="22.5"/>

<celldesigner:font size="12"/>

<celldesigner:view state="usual"/>

<celldesigner:usualView>

<celldesigner:innerPosition x="0.0" y="0.0"/>

<celldesigner:boxSize width="45.0" height="22.5"/>

<celldesigner:singleLine width="1.0"/>

<celldesigner:paint color="ffccff66" scheme="Color"/>

</celldesigner:usualView>

<celldesigner:briefView>

<celldesigner:innerPosition x="0.0" y="0.0"/>

<celldesigner:boxSize width="80.0" height="60.0"/>

<celldesigner:singleLine width="1.0"/>

<celldesigner:paint color="3fff0000" scheme="Color"/>

</celldesigner:briefView>

<celldesigner:info state="empty" angle="-1.5707963267948966"/>

</celldesigner:speciesAlias>

<celldesigner:speciesAlias id="sa35" species="s58">

<celldesigner:activity>inactive</celldesigner:activity>

<celldesigner:bounds x="281.0" y="237.5" w="70.0" h="25.0"/>

<celldesigner:font size="12"/>

<celldesigner:view state="usual"/>

<celldesigner:usualView>

<celldesigner:innerPosition x="0.0" y="0.0"/>

<celldesigner:boxSize width="70.0" height="25.0"/>

<celldesigner:singleLine width="1.0"/>

<celldesigner:paint color="ffccff66" scheme="Color"/>

</celldesigner:usualView>

<celldesigner:briefView>

<celldesigner:innerPosition x="0.0" y="0.0"/>

<celldesigner:boxSize width="80.0" height="60.0"/>

<celldesigner:singleLine width="1.0"/>

<celldesigner:paint color="3fff0000" scheme="Color"/>

</celldesigner:briefView>

<celldesigner:info state="empty" angle="-1.5707963267948966"/>

</celldesigner:speciesAlias>

<celldesigner:speciesAlias id="sa43" species="s60">
<celldesigner:activity>inactive</celldesigner:activity>
<celldesigner:bounds x="397.5" y="338.75" w="45.0" h="22.5"/>
<celldesigner:font size="12"/>
<celldesigner:view state="usual"/>
<celldesigner:usualView>
<celldesigner:innerPosition x="0.0" y="0.0"/>
<celldesigner:boxSize width="45.0" height="22.5"/>
<celldesigner:singleLine width="1.0"/>
<celldesigner:paint color="ffccff66" scheme="Color"/>
</celldesigner:usualView>
<celldesigner:briefView>
<celldesigner:innerPosition x="0.0" y="0.0"/>
<celldesigner:boxSize width="80.0" height="60.0"/>
<celldesigner:singleLine width="1.0"/>
<celldesigner:paint color="3fff0000" scheme="Color"/>
</celldesigner:briefView>
<celldesigner:info state="empty" angle="-1.5707963267948966"/>
</celldesigner:speciesAlias>
<celldesigner:speciesAlias id="sa44" species="s59">
<celldesigner:activity>inactive</celldesigner:activity>
<celldesigner:bounds x="232.5" y="298.75" w="55.0" h="22.5"/>
<celldesigner:font size="12"/>
<celldesigner:view state="usual"/>
<celldesigner:usualView>
<celldesigner:innerPosition x="0.0" y="0.0"/>
<celldesigner:boxSize width="55.0" height="22.5"/>

<celldesigner:singleLine width="1.0"/>

<celldesigner:paint color="ffccff66" scheme="Color"/>

</celldesigner:usualView>

<celldesigner:briefView>

<celldesigner:innerPosition x="0.0" y="0.0"/>

<celldesigner:boxSize width="80.0" height="60.0"/>

<celldesigner:singleLine width="1.0"/>

<celldesigner:paint color="3fff0000" scheme="Color"/>

</celldesigner:briefView>

<celldesigner:info state="empty" angle="-1.5707963267948966"/>

</celldesigner:speciesAlias>

<celldesigner:speciesAlias id="sa60" species="s63">

<celldesigner:activity>inactive</celldesigner:activity>

<celldesigner:bounds x="247.5" y="368.75" w="45.0" h="22.5"/>

<celldesigner:font size="12"/>

<celldesigner:view state="usual"/>

<celldesigner:usualView>

<celldesigner:innerPosition x="0.0" y="0.0"/>

<celldesigner:boxSize width="45.0" height="22.5"/>

<celldesigner:singleLine width="1.0"/>

<celldesigner:paint color="ffccff66" scheme="Color"/>

</celldesigner:usualView>

<celldesigner:briefView>

<celldesigner:innerPosition x="0.0" y="0.0"/>

<celldesigner:boxSize width="80.0" height="60.0"/>

<celldesigner:singleLine width="1.0"/>

<celldesigner:paint color="3fff0000" scheme="Color"/>

</celldesigner:briefView>

<celldesigner:info state="empty" angle="-1.5707963267948966"/>

</celldesigner:speciesAlias>

<celldesigner:speciesAlias id="sa74" species="s64">

<celldesigner:activity>inactive</celldesigner:activity>

<celldesigner:bounds x="247.5" y="428.75" w="45.0" h="22.5"/>

<celldesigner:font size="12"/>

<celldesigner:view state="usual"/>

<celldesigner:usualView>

<celldesigner:innerPosition x="0.0" y="0.0"/>

<celldesigner:boxSize width="45.0" height="22.5"/>

<celldesigner:singleLine width="1.0"/>

<celldesigner:paint color="ffccff66" scheme="Color"/>

</celldesigner:usualView>

<celldesigner:briefView>

<celldesigner:innerPosition x="0.0" y="0.0"/>

<celldesigner:boxSize width="80.0" height="60.0"/>

<celldesigner:singleLine width="1.0"/>

<celldesigner:paint color="3fff0000" scheme="Color"/>

</celldesigner:briefView>

<celldesigner:info state="empty" angle="-1.5707963267948966"/>

</celldesigner:speciesAlias>

<celldesigner:speciesAlias id="sa89" species="s65">

<celldesigner:activity>inactive</celldesigner:activity>

<celldesigner:bounds x="247.5" y="478.75" w="45.0" h="22.5"/>

<celldesigner:font size="12"/>

<celldesigner:view state="usual"/>

<celldesigner:usualView>

<celldesigner:innerPosition x="0.0" y="0.0"/>

<celldesigner:boxSize width="45.0" height="22.5"/>

<celldesigner:singleLine width="1.0"/>

<celldesigner:paint color="ffccff66" scheme="Color"/>

</celldesigner:usualView>

<celldesigner:briefView>

<celldesigner:innerPosition x="0.0" y="0.0"/>

<celldesigner:boxSize width="80.0" height="60.0"/>

<celldesigner:singleLine width="1.0"/>

<celldesigner:paint color="3fff0000" scheme="Color"/>

</celldesigner:briefView>

<celldesigner:info state="empty" angle="-1.5707963267948966"/>

</celldesigner:speciesAlias>

<celldesigner:speciesAlias id="sa108" species="s66">

<celldesigner:activity>inactive</celldesigner:activity>

<celldesigner:bounds x="247.5" y="528.75" w="45.0" h="22.5"/>

<celldesigner:font size="12"/>

<celldesigner:view state="usual"/>

<celldesigner:usualView>

<celldesigner:innerPosition x="0.0" y="0.0"/>

<celldesigner:boxSize width="45.0" height="22.5"/>

<celldesigner:singleLine width="1.0"/>

<celldesigner:paint color="ffccff66" scheme="Color"/>

</celldesigner:usualView>

<celldesigner:briefView>

<celldesigner:innerPosition x="0.0" y="0.0"/>

<celldesigner:boxSize width="80.0" height="60.0"/>

<celldesigner:singleLine width="1.0"/>

<celldesigner:paint color="3fff0000" scheme="Color"/>

</celldesigner:briefView>

<celldesigner:info state="empty" angle="-1.5707963267948966"/>

</celldesigner:speciesAlias>

<celldesigner:speciesAlias id="sa143" species="s2">

<celldesigner:activity>inactive</celldesigner:activity>

<celldesigner:bounds x="252.5" y="578.75" w="35.0" h="22.5"/>

<celldesigner:font size="12"/>

<celldesigner:view state="usual"/>

<celldesigner:usualView>

<celldesigner:innerPosition x="0.0" y="0.0"/>

<celldesigner:boxSize width="35.0" height="22.5"/>

<celldesigner:singleLine width="1.0"/>

<celldesigner:paint color="ffccff66" scheme="Color"/>

</celldesigner:usualView>

<celldesigner:briefView>

<celldesigner:innerPosition x="0.0" y="0.0"/>

<celldesigner:boxSize width="80.0" height="60.0"/>

<celldesigner:singleLine width="1.0"/>

<celldesigner:paint color="3fff0000" scheme="Color"/>

</celldesigner:briefView>

<celldesigner:info state="empty" angle="-1.5707963267948966"/>

</celldesigner:speciesAlias>

<celldesigner:speciesAlias id="sa222" species="s37">

<celldesigner:activity>inactive</celldesigner:activity>

<celldesigner:bounds x="154.75" y="608.0" w="30.5" h="24.0"/>

<celldesigner:font size="12"/>

<celldesigner:view state="usual"/>

<celldesigner:usualView>

<celldesigner:innerPosition x="0.0" y="0.0"/>

<celldesigner:boxSize width="30.5" height="24.0"/>

<celldesigner:singleLine width="1.0"/>

<celldesigner:paint color="ffccff66" scheme="Color"/>

</celldesigner:usualView>

<celldesigner:briefView>

<celldesigner:innerPosition x="0.0" y="0.0"/>

<celldesigner:boxSize width="80.0" height="60.0"/>

<celldesigner:singleLine width="1.0"/>

<celldesigner:paint color="3fff0000" scheme="Color"/>

</celldesigner:briefView>

<celldesigner:info state="empty" angle="-1.5707963267948966"/>

</celldesigner:speciesAlias>

<celldesigner:speciesAlias id="sa226" species="s33">

<celldesigner:activity>inactive</celldesigner:activity>

<celldesigner:bounds x="338.5" y="670.75" w="43.0" h="18.5"/>

<celldesigner:font size="12"/>

<celldesigner:view state="usual"/>

<celldesigner:usualView>

<celldesigner:innerPosition x="0.0" y="0.0"/>

<celldesigner:boxSize width="43.0" height="18.5"/>

<celldesigner:singleLine width="1.0"/>

<celldesigner:paint color="ffccff66" scheme="Color"/>

</celldesigner:usualView>

<celldesigner:briefView>

<celldesigner:innerPosition x="0.0" y="0.0"/>

<celldesigner:boxSize width="80.0" height="60.0"/>

<celldesigner:singleLine width="1.0"/>

<celldesigner:paint color="3fff0000" scheme="Color"/>

</celldesigner:briefView>

<celldesigner:info state="empty" angle="-1.5707963267948966"/>

</celldesigner:speciesAlias>

<celldesigner:speciesAlias id="sa230" species="s28">

<celldesigner:activity>inactive</celldesigner:activity>

<celldesigner:bounds x="383.25" y="740.625" w="33.5" h="18.75"/>

<celldesigner:font size="12"/>

<celldesigner:view state="usual"/>

<celldesigner:usualView>

<celldesigner:innerPosition x="0.0" y="0.0"/>

<celldesigner:boxSize width="33.5" height="18.75"/>

<celldesigner:singleLine width="1.0"/>

<celldesigner:paint color="ffccff66" scheme="Color"/>

</celldesigner:usualView>

<celldesigner:briefView>

<celldesigner:innerPosition x="0.0" y="0.0"/>

<celldesigner:boxSize width="80.0" height="60.0"/>

<celldesigner:singleLine width="1.0"/>

<celldesigner:paint color="3fff0000" scheme="Color"/>

</celldesigner:briefView>

<celldesigner:info state="empty" angle="-1.5707963267948966"/>

</celldesigner:speciesAlias>

<celldesigner:speciesAlias id="sa234" species="s24">

<celldesigner:activity>inactive</celldesigner:activity>

<celldesigner:bounds x="382.5" y="798.75" w="35.0" h="22.5"/>

<celldesigner:font size="12"/>

<celldesigner:view state="usual"/>

<celldesigner:usualView>

<celldesigner:innerPosition x="0.0" y="0.0"/>

<celldesigner:boxSize width="35.0" height="22.5"/>

<celldesigner:singleLine width="1.0"/>

<celldesigner:paint color="ffccff66" scheme="Color"/>

</celldesigner:usualView>

<celldesigner:briefView>

<celldesigner:innerPosition x="0.0" y="0.0"/>

<celldesigner:boxSize width="80.0" height="60.0"/>

<celldesigner:singleLine width="1.0"/>

<celldesigner:paint color="3fff0000" scheme="Color"/>

</celldesigner:briefView>

<celldesigner:info state="empty" angle="-1.5707963267948966"/>

</celldesigner:speciesAlias>

<celldesigner:speciesAlias id="sa235" species="s22">

<celldesigner:activity>inactive</celldesigner:activity>

<celldesigner:bounds x="327.5" y="851.25" w="65.0" h="17.5"/>

<celldesigner:font size="12"/>

<celldesigner:view state="usual"/>

<celldesigner:usualView>

<celldesigner:innerPosition x="0.0" y="0.0"/>

<celldesigner:boxSize width="65.0" height="17.5"/>

<celldesigner:singleLine width="1.0"/>

<celldesigner:paint color="ffccff66" scheme="Color"/>

</celldesigner:usualView>

<celldesigner:briefView>

<celldesigner:innerPosition x="0.0" y="0.0"/>

<celldesigner:boxSize width="80.0" height="60.0"/>

<celldesigner:singleLine width="1.0"/>

<celldesigner:paint color="3fff0000" scheme="Color"/>

</celldesigner:briefView>

<celldesigner:info state="empty" angle="-1.5707963267948966"/>

</celldesigner:speciesAlias>

<celldesigner:speciesAlias id="sa238" species="s16">

<celldesigner:activity>inactive</celldesigner:activity>

<celldesigner:bounds x="227.5" y="839.0" w="45.0" h="22.0"/>

<celldesigner:font size="12"/>

<celldesigner:view state="usual"/>

<celldesigner:usualView>

<celldesigner:innerPosition x="0.0" y="0.0"/>

<celldesigner:boxSize width="45.0" height="22.0"/>

<celldesigner:singleLine width="1.0"/>

<celldesigner:paint color="ffff00ff" scheme="Color"/>

</celldesigner:usualView>

<celldesigner:briefView>

<celldesigner:innerPosition x="0.0" y="0.0"/>

<celldesigner:boxSize width="80.0" height="60.0"/>

<celldesigner:singleLine width="1.0"/>

<celldesigner:paint color="3fff0000" scheme="Color"/>

</celldesigner:briefView>

<celldesigner:info state="empty" angle="-1.5707963267948966"/>

</celldesigner:speciesAlias>

<celldesigner:speciesAlias id="sa241" species="s15">

<celldesigner:activity>inactive</celldesigner:activity>

<celldesigner:bounds x="177.5" y="798.75" w="45.0" h="22.5"/>

<celldesigner:font size="12"/>

<celldesigner:view state="usual"/>

<celldesigner:usualView>

<celldesigner:innerPosition x="0.0" y="0.0"/>

<celldesigner:boxSize width="45.0" height="22.5"/>

<celldesigner:singleLine width="1.0"/>

<celldesigner:paint color="ffccff66" scheme="Color"/>

</celldesigner:usualView>

<celldesigner:briefView>

<celldesigner:innerPosition x="0.0" y="0.0"/>

<celldesigner:boxSize width="80.0" height="60.0"/>

<celldesigner:singleLine width="1.0"/>

<celldesigner:paint color="3fff0000" scheme="Color"/>

</celldesigner:briefView>

<celldesigner:info state="empty" angle="-1.5707963267948966"/>

</celldesigner:speciesAlias>

<celldesigner:speciesAlias id="sa242" species="s14">

<celldesigner:activity>inactive</celldesigner:activity>

<celldesigner:bounds x="183.5" y="748.75" w="45.0" h="22.5"/>

<celldesigner:font size="12"/>

<celldesigner:view state="usual"/>

<celldesigner:usualView>

<celldesigner:innerPosition x="0.0" y="0.0"/>

<celldesigner:boxSize width="45.0" height="22.5"/>

<celldesigner:singleLine width="1.0"/>

<celldesigner:paint color="ffccff66" scheme="Color"/>

</celldesigner:usualView>

<celldesigner:briefView>

<celldesigner:innerPosition x="0.0" y="0.0"/>

<celldesigner:boxSize width="80.0" height="60.0"/>

<celldesigner:singleLine width="1.0"/>

<celldesigner:paint color="3fff0000" scheme="Color"/>

</celldesigner:briefView>

<celldesigner:info state="empty" angle="-1.5707963267948966"/>

</celldesigner:speciesAlias>

<celldesigner:speciesAlias id="sa243" species="s13">

<celldesigner:activity>inactive</celldesigner:activity>

<celldesigner:bounds x="285.25" y="688.9375" w="41.5" h="22.125"/>

<celldesigner:font size="12"/>

<celldesigner:view state="usual"/>

<celldesigner:usualView>

<celldesigner:innerPosition x="0.0" y="0.0"/>

<celldesigner:boxSize width="41.5" height="22.125"/>

<celldesigner:singleLine width="1.0"/>

<celldesigner:paint color="ffccff66" scheme="Color"/>

</celldesigner:usualView>

<celldesigner:briefView>

<celldesigner:innerPosition x="0.0" y="0.0"/>

<celldesigner:boxSize width="80.0" height="60.0"/>

<celldesigner:singleLine width="1.0"/>

<celldesigner:paint color="3fff0000" scheme="Color"/>

</celldesigner:briefView>

<celldesigner:info state="empty" angle="-1.5707963267948966"/>

</celldesigner:speciesAlias>

<celldesigner:speciesAlias id="sa245" species="s11">

<celldesigner:activity>inactive</celldesigner:activity>

<celldesigner:bounds x="198.5" y="688.75" w="35.0" h="22.5"/>

<celldesigner:font size="12"/>

<celldesigner:view state="usual"/>

<celldesigner:usualView>

<celldesigner:innerPosition x="0.0" y="0.0"/>

<celldesigner:boxSize width="35.0" height="22.5"/>

<celldesigner:singleLine width="1.0"/>

<celldesigner:paint color="ffccff66" scheme="Color"/>

</celldesigner:usualView>

<celldesigner:briefView>

<celldesigner:innerPosition x="0.0" y="0.0"/>

<celldesigner:boxSize width="80.0" height="60.0"/>

<celldesigner:singleLine width="1.0"/>

<celldesigner:paint color="3fff0000" scheme="Color"/>

</celldesigner:briefView>

<celldesigner:info state="empty" angle="-1.5707963267948966"/>

</celldesigner:speciesAlias>

<celldesigner:speciesAlias id="sa247" species="s7">

<celldesigner:activity>inactive</celldesigner:activity>

<celldesigner:bounds x="233.5" y="638.75" w="45.0" h="22.5"/>

<celldesigner:font size="12"/>

<celldesigner:view state="usual"/>

<celldesigner:usualView>

<celldesigner:innerPosition x="0.0" y="0.0"/>

<celldesigner:boxSize width="45.0" height="22.5"/>

<celldesigner:singleLine width="1.0"/>

<celldesigner:paint color="ffccff66" scheme="Color"/>

</celldesigner:usualView>

<celldesigner:briefView>

<celldesigner:innerPosition x="0.0" y="0.0"/>

<celldesigner:boxSize width="80.0" height="60.0"/>

<celldesigner:singleLine width="1.0"/>

<celldesigner:paint color="3fff0000" scheme="Color"/>

</celldesigner:briefView>

<celldesigner:info state="empty" angle="-1.5707963267948966"/>

</celldesigner:speciesAlias>

<celldesigner:speciesAlias id="sa321" species="s1">

<celldesigner:activity>inactive</celldesigner:activity>

<celldesigner:bounds x="377.5" y="78.75" w="65.0" h="22.5"/>

<celldesigner:font size="12"/>

<celldesigner:view state="usual"/>

<celldesigner:usualView>

<celldesigner:innerPosition x="0.0" y="0.0"/>

<celldesigner:boxSize width="65.0" height="22.5"/>

<celldesigner:singleLine width="1.0"/>

<celldesigner:paint color="ffccff66" scheme="Color"/>
</celldesigner:usualView>
<celldesigner:briefView>
<celldesigner:innerPosition x="0.0" y="0.0"/>
<celldesigner:boxSize width="80.0" height="60.0"/>
<celldesigner:singleLine width="1.0"/>
<celldesigner:paint color="3fff0000" scheme="Color"/>
</celldesigner:briefView>
<celldesigner:info state="empty" angle="-1.5707963267948966"/>
</celldesigner:speciesAlias>
<celldesigner:speciesAlias id="sa322" species="s3">
<celldesigner:activity>inactive</celldesigner:activity>
<celldesigner:bounds x="375.0" y="147.5" w="70.0" h="25.0"/>
<celldesigner:font size="12"/>
<celldesigner:view state="usual"/>
<celldesigner:usualView>
<celldesigner:innerPosition x="0.0" y="0.0"/>
<celldesigner:boxSize width="70.0" height="25.0"/>
<celldesigner:singleLine width="1.0"/>
<celldesigner:paint color="ffccff66" scheme="Color"/>
</celldesigner:usualView>
<celldesigner:briefView>
<celldesigner:innerPosition x="0.0" y="0.0"/>
<celldesigner:boxSize width="80.0" height="60.0"/>
<celldesigner:singleLine width="1.0"/>
<celldesigner:paint color="3fff0000" scheme="Color"/>
</celldesigner:briefView>

<celldesigner:info state="empty" angle="-1.5707963267948966"/>

</celldesigner:speciesAlias>

<celldesigner:speciesAlias id="sa333" species="s5">

<celldesigner:activity>inactive</celldesigner:activity>

<celldesigner:bounds x="375.0" y="237.5" w="70.0" h="25.0"/>

<celldesigner:font size="12"/>

<celldesigner:view state="usual"/>

<celldesigner:usualView>

<celldesigner:innerPosition x="0.0" y="0.0"/>

<celldesigner:boxSize width="70.0" height="25.0"/>

<celldesigner:singleLine width="1.0"/>

<celldesigner:paint color="ffccff66" scheme="Color"/>

</celldesigner:usualView>

<celldesigner:briefView>

<celldesigner:innerPosition x="0.0" y="0.0"/>

<celldesigner:boxSize width="80.0" height="60.0"/>

<celldesigner:singleLine width="1.0"/>

<celldesigner:paint color="3fff0000" scheme="Color"/>

</celldesigner:briefView>

<celldesigner:info state="empty" angle="-1.5707963267948966"/>

</celldesigner:speciesAlias>

<celldesigner:speciesAlias id="sa349" species="s432">

<celldesigner:activity>inactive</celldesigner:activity>

<celldesigner:bounds x="502.5" y="358.75" w="55.0" h="22.5"/>

<celldesigner:font size="12"/>

<celldesigner:view state="usual"/>

<celldesigner:usualView>

<celldesigner:innerPosition x="0.0" y="0.0"/>

<celldesigner:boxSize width="55.0" height="22.5"/>

<celldesigner:singleLine width="1.0"/>

<celldesigner:paint color="ffccff66" scheme="Color"/>

</celldesigner:usualView>

<celldesigner:briefView>

<celldesigner:innerPosition x="0.0" y="0.0"/>

<celldesigner:boxSize width="80.0" height="60.0"/>

<celldesigner:singleLine width="1.0"/>

<celldesigner:paint color="3fff0000" scheme="Color"/>

</celldesigner:briefView>

<celldesigner:info state="empty" angle="-1.5707963267948966"/>

</celldesigner:speciesAlias>

<celldesigner:speciesAlias id="sa351" species="s21">

<celldesigner:activity>inactive</celldesigner:activity>

<celldesigner:bounds x="632.5" y="258.75" w="55.0" h="22.5"/>

<celldesigner:font size="12"/>

<celldesigner:view state="usual"/>

<celldesigner:usualView>

<celldesigner:innerPosition x="0.0" y="0.0"/>

<celldesigner:boxSize width="55.0" height="22.5"/>

<celldesigner:singleLine width="1.0"/>

<celldesigner:paint color="ffccff66" scheme="Color"/>

</celldesigner:usualView>

<celldesigner:briefView>

<celldesigner:innerPosition x="0.0" y="0.0"/>

<celldesigner:boxSize width="80.0" height="60.0"/>

<celldesigner:singleLine width="1.0"/>
<celldesigner:paint color="3fff0000" scheme="Color"/>
</celldesigner:briefView>
<celldesigner:info state="empty" angle="-1.5707963267948966"/>
</celldesigner:speciesAlias>
<celldesigner:speciesAlias id="sa352" species="s23">
<celldesigner:activity>inactive</celldesigner:activity>
<celldesigner:bounds x="577.5" y="398.75" w="45.0" h="22.5"/>
<celldesigner:font size="12"/>
<celldesigner:view state="usual"/>
<celldesigner:usualView>
<celldesigner:innerPosition x="0.0" y="0.0"/>
<celldesigner:boxSize width="45.0" height="22.5"/>
<celldesigner:singleLine width="1.0"/>
<celldesigner:paint color="ffccff66" scheme="Color"/>
</celldesigner:usualView>
<celldesigner:briefView>
<celldesigner:innerPosition x="0.0" y="0.0"/>
<celldesigner:boxSize width="80.0" height="60.0"/>
<celldesigner:singleLine width="1.0"/>
<celldesigner:paint color="3fff0000" scheme="Color"/>
</celldesigner:briefView>
<celldesigner:info state="empty" angle="-1.5707963267948966"/>
</celldesigner:speciesAlias>
<celldesigner:speciesAlias id="sa353" species="s34">
<celldesigner:activity>inactive</celldesigner:activity>
<celldesigner:bounds x="557.5" y="268.75" w="45.0" h="22.5"/>

<celldesigner:font size="12"/>

<celldesigner:view state="usual"/>

<celldesigner:usualView>

<celldesigner:innerPosition x="0.0" y="0.0"/>

<celldesigner:boxSize width="45.0" height="22.5"/>

<celldesigner:singleLine width="1.0"/>

<celldesigner:paint color="ffccff66" scheme="Color"/>

</celldesigner:usualView>

<celldesigner:briefView>

<celldesigner:innerPosition x="0.0" y="0.0"/>

<celldesigner:boxSize width="80.0" height="60.0"/>

<celldesigner:singleLine width="1.0"/>

<celldesigner:paint color="3fff0000" scheme="Color"/>

</celldesigner:briefView>

<celldesigner:info state="empty" angle="-1.5707963267948966"/>

</celldesigner:speciesAlias>

<celldesigner:speciesAlias id="sa354" species="s35">

<celldesigner:activity>inactive</celldesigner:activity>

<celldesigner:bounds x="672.5" y="368.75" w="35.0" h="22.5"/>

<celldesigner:font size="12"/>

<celldesigner:view state="usual"/>

<celldesigner:usualView>

<celldesigner:innerPosition x="0.0" y="0.0"/>

<celldesigner:boxSize width="35.0" height="22.5"/>

<celldesigner:singleLine width="1.0"/>

<celldesigner:paint color="ffccff66" scheme="Color"/>

</celldesigner:usualView>

<celldesigner:briefView>

<celldesigner:innerPosition x="0.0" y="0.0"/>

<celldesigner:boxSize width="80.0" height="60.0"/>

<celldesigner:singleLine width="1.0"/>

<celldesigner:paint color="3fff0000" scheme="Color"/>

</celldesigner:briefView>

<celldesigner:info state="empty" angle="-1.5707963267948966"/>

</celldesigner:speciesAlias>

<celldesigner:speciesAlias id="sa355" species="s36">

<celldesigner:activity>inactive</celldesigner:activity>

<celldesigner:bounds x="662.5" y="418.75" w="35.0" h="22.5"/>

<celldesigner:font size="12"/>

<celldesigner:view state="usual"/>

<celldesigner:usualView>

<celldesigner:innerPosition x="0.0" y="0.0"/>

<celldesigner:boxSize width="35.0" height="22.5"/>

<celldesigner:singleLine width="1.0"/>

<celldesigner:paint color="ffccff66" scheme="Color"/>

</celldesigner:usualView>

<celldesigner:briefView>

<celldesigner:innerPosition x="0.0" y="0.0"/>

<celldesigner:boxSize width="80.0" height="60.0"/>

<celldesigner:singleLine width="1.0"/>

<celldesigner:paint color="3fff0000" scheme="Color"/>

</celldesigner:briefView>

<celldesigner:info state="empty" angle="-1.5707963267948966"/>

</celldesigner:speciesAlias>

<celldesigner:speciesAlias id="sa359" species="s434">
<celldesigner:activity>inactive</celldesigner:activity>
<celldesigner:bounds x="85.0" y="97.5" w="70.0" h="25.0"/>
<celldesigner:font size="12"/>
<celldesigner:view state="usual"/>
<celldesigner:usualView>
<celldesigner:innerPosition x="0.0" y="0.0"/>
<celldesigner:boxSize width="70.0" height="25.0"/>
<celldesigner:singleLine width="1.0"/>
<celldesigner:paint color="ffccff66" scheme="Color"/>
</celldesigner:usualView>
<celldesigner:briefView>
<celldesigner:innerPosition x="0.0" y="0.0"/>
<celldesigner:boxSize width="80.0" height="60.0"/>
<celldesigner:singleLine width="1.0"/>
<celldesigner:paint color="3fff0000" scheme="Color"/>
</celldesigner:briefView>
<celldesigner:info state="empty" angle="-1.5707963267948966"/>
</celldesigner:speciesAlias>
<celldesigner:speciesAlias id="sa360" species="s435">
<celldesigner:activity>inactive</celldesigner:activity>
<celldesigner:bounds x="185.0" y="77.5" w="70.0" h="25.0"/>
<celldesigner:font size="12"/>
<celldesigner:view state="usual"/>
<celldesigner:usualView>
<celldesigner:innerPosition x="0.0" y="0.0"/>
<celldesigner:boxSize width="70.0" height="25.0"/>

<celldesigner:singleLine width="1.0"/>

<celldesigner:paint color="ffccff66" scheme="Color"/>

</celldesigner:usualView>

<celldesigner:briefView>

<celldesigner:innerPosition x="0.0" y="0.0"/>

<celldesigner:boxSize width="80.0" height="60.0"/>

<celldesigner:singleLine width="1.0"/>

<celldesigner:paint color="3fff0000" scheme="Color"/>

</celldesigner:briefView>

<celldesigner:info state="empty" angle="-1.5707963267948966"/>

</celldesigner:speciesAlias>

<celldesigner:speciesAlias id="sa384" species="s456">

<celldesigner:activity>inactive</celldesigner:activity>

<celldesigner:bounds x="99.0" y="431.0" w="54.0" h="16.0"/>

<celldesigner:font size="12"/>

<celldesigner:view state="usual"/>

<celldesigner:usualView>

<celldesigner:innerPosition x="0.0" y="0.0"/>

<celldesigner:boxSize width="54.0" height="16.0"/>

<celldesigner:singleLine width="1.0"/>

<celldesigner:paint color="ffccff66" scheme="Color"/>

</celldesigner:usualView>

<celldesigner:briefView>

<celldesigner:innerPosition x="0.0" y="0.0"/>

<celldesigner:boxSize width="80.0" height="60.0"/>

<celldesigner:singleLine width="1.0"/>

<celldesigner:paint color="3fff0000" scheme="Color"/>

</celldesigner:briefView>
<celldesigner:info state="empty" angle="-1.5707963267948966"/>
</celldesigner:speciesAlias>
<celldesigner:speciesAlias id="sa385" species="s459">
<celldesigner:activity>inactive</celldesigner:activity>
<celldesigner:bounds x="144.5" y="481.5" w="71.0" h="17.0"/>
<celldesigner:font size="12"/>
<celldesigner:view state="usual"/>
<celldesigner:usualView>
<celldesigner:innerPosition x="0.0" y="0.0"/>
<celldesigner:boxSize width="71.0" height="17.0"/>
<celldesigner:singleLine width="1.0"/>
<celldesigner:paint color="ffccff66" scheme="Color"/>
</celldesigner:usualView>
<celldesigner:briefView>
<celldesigner:innerPosition x="0.0" y="0.0"/>
<celldesigner:boxSize width="80.0" height="60.0"/>
<celldesigner:singleLine width="1.0"/>
<celldesigner:paint color="3fff0000" scheme="Color"/>
</celldesigner:briefView>
<celldesigner:info state="empty" angle="-1.5707963267948966"/>
</celldesigner:speciesAlias>
<celldesigner:speciesAlias id="sa387" species="s159">
<celldesigner:activity>inactive</celldesigner:activity>
<celldesigner:bounds x="76.5" y="550.5" w="65.0" h="17.0"/>
<celldesigner:font size="12"/>
<celldesigner:view state="usual"/>

```
<celldesigner:usualView>
<celldesigner:innerPosition x="0.0" y="0.0"/>
<celldesigner:boxSize width="65.0" height="17.0"/>
<celldesigner:singleLine width="1.0"/>
<celldesigner:paint color="ffccff66" scheme="Color"/>
</celldesigner:usualView>
<celldesigner:briefView>
<celldesigner:innerPosition x="0.0" y="0.0"/>
<celldesigner:boxSize width="80.0" height="60.0"/>
<celldesigner:singleLine width="1.0"/>
<celldesigner:paint color="3fff0000" scheme="Color"/>
</celldesigner:briefView>
<celldesigner:info state="empty" angle="-1.5707963267948966"/>
</celldesigner:speciesAlias>
<celldesigner:speciesAlias id="sa388" species="s461">
<celldesigner:activity>inactive</celldesigner:activity>
<celldesigner:bounds x="77.5" y="623.5" w="65.0" h="17.0"/>
<celldesigner:font size="12"/>
<celldesigner:view state="usual"/>
<celldesigner:usualView>
<celldesigner:innerPosition x="0.0" y="0.0"/>
<celldesigner:boxSize width="65.0" height="17.0"/>
<celldesigner:singleLine width="1.0"/>
<celldesigner:paint color="ffccff66" scheme="Color"/>
</celldesigner:usualView>
<celldesigner:briefView>
<celldesigner:innerPosition x="0.0" y="0.0"/>
```

<celldesigner:boxSize width="80.0" height="60.0"/>
<celldesigner:singleLine width="1.0"/>
<celldesigner:paint color="3fff0000" scheme="Color"/>
</celldesigner:briefView>
<celldesigner:info state="empty" angle="-1.5707963267948966"/>
</celldesigner:speciesAlias>
<celldesigner:speciesAlias id="sa389" species="s163">
<celldesigner:activity>inactive</celldesigner:activity>
<celldesigner:bounds x="81.5" y="691.5" w="65.0" h="17.0"/>
<celldesigner:font size="12"/>
<celldesigner:view state="usual"/>
<celldesigner:usualView>
<celldesigner:innerPosition x="0.0" y="0.0"/>
<celldesigner:boxSize width="65.0" height="17.0"/>
<celldesigner:singleLine width="1.0"/>
<celldesigner:paint color="ffccff66" scheme="Color"/>
</celldesigner:usualView>
<celldesigner:briefView>
<celldesigner:innerPosition x="0.0" y="0.0"/>
<celldesigner:boxSize width="80.0" height="60.0"/>
<celldesigner:singleLine width="1.0"/>
<celldesigner:paint color="3fff0000" scheme="Color"/>
</celldesigner:briefView>
<celldesigner:info state="empty" angle="-1.5707963267948966"/>
</celldesigner:speciesAlias>
<celldesigner:speciesAlias id="sa390" species="s462">
<celldesigner:activity>inactive</celldesigner:activity>

<celldesigner:bounds x="83.5" y="762.5" w="65.0" h="17.0"/>

<celldesigner:font size="12"/>

<celldesigner:view state="usual"/>

<celldesigner:usualView>

<celldesigner:innerPosition x="0.0" y="0.0"/>

<celldesigner:boxSize width="65.0" height="17.0"/>

<celldesigner:singleLine width="1.0"/>

<celldesigner:paint color="ffccff66" scheme="Color"/>

</celldesigner:usualView>

<celldesigner:briefView>

<celldesigner:innerPosition x="0.0" y="0.0"/>

<celldesigner:boxSize width="80.0" height="60.0"/>

<celldesigner:singleLine width="1.0"/>

<celldesigner:paint color="3fff0000" scheme="Color"/>

</celldesigner:briefView>

<celldesigner:info state="empty" angle="-1.5707963267948966"/>

</celldesigner:speciesAlias>

<celldesigner:speciesAlias id="sa391" species="s165">

<celldesigner:activity>inactive</celldesigner:activity>

<celldesigner:bounds x="81.5" y="826.5" w="65.0" h="17.0"/>

<celldesigner:font size="12"/>

<celldesigner:view state="usual"/>

<celldesigner:usualView>

<celldesigner:innerPosition x="0.0" y="0.0"/>

<celldesigner:boxSize width="65.0" height="17.0"/>

<celldesigner:singleLine width="1.0"/>

<celldesigner:paint color="ffccff66" scheme="Color"/>

</celldesigner:usualView>

<celldesigner:briefView>

<celldesigner:innerPosition x="0.0" y="0.0"/>

<celldesigner:boxSize width="80.0" height="60.0"/>

<celldesigner:singleLine width="1.0"/>

<celldesigner:paint color="3fff0000" scheme="Color"/>

</celldesigner:briefView>

<celldesigner:info state="empty" angle="-1.5707963267948966"/>

</celldesigner:speciesAlias>

<celldesigner:speciesAlias id="sa392" species="s476">

<celldesigner:activity>inactive</celldesigner:activity>

<celldesigner:bounds x="143.0" y="1262.5" w="54.0" h="19.0"/>

<celldesigner:font size="12"/>

<celldesigner:view state="usual"/>

<celldesigner:usualView>

<celldesigner:innerPosition x="0.0" y="0.0"/>

<celldesigner:boxSize width="54.0" height="19.0"/>

<celldesigner:singleLine width="1.0"/>

<celldesigner:paint color="ffccff66" scheme="Color"/>

</celldesigner:usualView>

<celldesigner:briefView>

<celldesigner:innerPosition x="0.0" y="0.0"/>

<celldesigner:boxSize width="80.0" height="60.0"/>

<celldesigner:singleLine width="1.0"/>

<celldesigner:paint color="3fff0000" scheme="Color"/>

</celldesigner:briefView>

<celldesigner:info state="empty" angle="-1.5707963267948966"/>

</celldesigner:speciesAlias>

<celldesigner:speciesAlias id="sa393" species="s172">

<celldesigner:activity>inactive</celldesigner:activity>

<celldesigner:bounds x="83.5" y="1232.5" w="73.0" h="19.0"/>

<celldesigner:font size="12"/>

<celldesigner:view state="usual"/>

<celldesigner:usualView>

<celldesigner:innerPosition x="0.0" y="0.0"/>

<celldesigner:boxSize width="73.0" height="19.0"/>

<celldesigner:singleLine width="1.0"/>

<celldesigner:paint color="ffccff66" scheme="Color"/>

</celldesigner:usualView>

<celldesigner:briefView>

<celldesigner:innerPosition x="0.0" y="0.0"/>

<celldesigner:boxSize width="80.0" height="60.0"/>

<celldesigner:singleLine width="1.0"/>

<celldesigner:paint color="3fff0000" scheme="Color"/>

</celldesigner:briefView>

<celldesigner:info state="empty" angle="-1.5707963267948966"/>

</celldesigner:speciesAlias>

<celldesigner:speciesAlias id="sa394" species="s173">

<celldesigner:activity>inactive</celldesigner:activity>

<celldesigner:bounds x="83.5" y="1292.5" w="73.0" h="19.0"/>

<celldesigner:font size="12"/>

<celldesigner:view state="usual"/>

<celldesigner:usualView>

<celldesigner:innerPosition x="0.0" y="0.0"/>

<celldesigner:boxSize width="73.0" height="19.0"/>

<celldesigner:singleLine width="1.0"/>

<celldesigner:paint color="ffccff66" scheme="Color"/>

</celldesigner:usualView>

<celldesigner:briefView>

<celldesigner:innerPosition x="0.0" y="0.0"/>

<celldesigner:boxSize width="80.0" height="60.0"/>

<celldesigner:singleLine width="1.0"/>

<celldesigner:paint color="3fff0000" scheme="Color"/>

</celldesigner:briefView>

<celldesigner:info state="empty" angle="-1.5707963267948966"/>

</celldesigner:speciesAlias>

<celldesigner:speciesAlias id="sa395" species="s174">

<celldesigner:activity>inactive</celldesigner:activity>

<celldesigner:bounds x="83.5" y="1362.5" w="73.0" h="19.0"/>

<celldesigner:font size="12"/>

<celldesigner:view state="usual"/>

<celldesigner:usualView>

<celldesigner:innerPosition x="0.0" y="0.0"/>

<celldesigner:boxSize width="73.0" height="19.0"/>

<celldesigner:singleLine width="1.0"/>

<celldesigner:paint color="ffccff66" scheme="Color"/>

</celldesigner:usualView>

<celldesigner:briefView>

<celldesigner:innerPosition x="0.0" y="0.0"/>

<celldesigner:boxSize width="80.0" height="60.0"/>

<celldesigner:singleLine width="1.0"/>

<celldesigner:paint color="3fff0000" scheme="Color"/>

</celldesigner:briefView>

<celldesigner:info state="empty" angle="-1.5707963267948966"/>

</celldesigner:speciesAlias>

<celldesigner:speciesAlias id="sa396" species="s1099">

<celldesigner:activity>inactive</celldesigner:activity>

<celldesigner:bounds x="94.75" y="1421.75" w="50.5" h="20.5"/>

<celldesigner:font size="12"/>

<celldesigner:view state="usual"/>

<celldesigner:usualView>

<celldesigner:innerPosition x="0.0" y="0.0"/>

<celldesigner:boxSize width="50.5" height="20.5"/>

<celldesigner:singleLine width="1.0"/>

<celldesigner:paint color="ffffff00" scheme="Color"/>

</celldesigner:usualView>

<celldesigner:briefView>

<celldesigner:innerPosition x="0.0" y="0.0"/>

<celldesigner:boxSize width="80.0" height="60.0"/>

<celldesigner:singleLine width="1.0"/>

<celldesigner:paint color="3fff0000" scheme="Color"/>

</celldesigner:briefView>

<celldesigner:info state="empty" angle="-1.5707963267948966"/>

</celldesigner:speciesAlias>

<celldesigner:speciesAlias id="sa397" species="s463">

<celldesigner:activity>inactive</celldesigner:activity>

<celldesigner:bounds x="93.5" y="1484.0" w="57.0" h="16.0"/>

<celldesigner:font size="12"/>

<celldesigner:view state="usual"/>

<celldesigner:usualView>

<celldesigner:innerPosition x="0.0" y="0.0"/>

<celldesigner:boxSize width="57.0" height="16.0"/>

<celldesigner:singleLine width="1.0"/>

<celldesigner:paint color="ffccff66" scheme="Color"/>

</celldesigner:usualView>

<celldesigner:briefView>

<celldesigner:innerPosition x="0.0" y="0.0"/>

<celldesigner:boxSize width="80.0" height="60.0"/>

<celldesigner:singleLine width="1.0"/>

<celldesigner:paint color="3fff0000" scheme="Color"/>

</celldesigner:briefView>

<celldesigner:info state="empty" angle="-1.5707963267948966"/>

</celldesigner:speciesAlias>

<celldesigner:speciesAlias id="sa398" species="s178">

<celldesigner:activity>inactive</celldesigner:activity>

<celldesigner:bounds x="96.75" y="1602.25" w="46.5" h="19.5"/>

<celldesigner:font size="12"/>

<celldesigner:view state="usual"/>

<celldesigner:usualView>

<celldesigner:innerPosition x="0.0" y="0.0"/>

<celldesigner:boxSize width="46.5" height="19.5"/>

<celldesigner:singleLine width="1.0"/>

<celldesigner:paint color="ffccff66" scheme="Color"/>

</celldesigner:usualView>

<celldesigner:briefView>

<celldesigner:innerPosition x="0.0" y="0.0"/>

<celldesigner:boxSize width="80.0" height="60.0"/>

<celldesigner:singleLine width="1.0"/>

<celldesigner:paint color="3fff0000" scheme="Color"/>

</celldesigner:briefView>

<celldesigner:info state="empty" angle="-1.5707963267948966"/>

</celldesigner:speciesAlias>

<celldesigner:speciesAlias id="sa399" species="s464">

<celldesigner:activity>inactive</celldesigner:activity>

<celldesigner:bounds x="83.5" y="1662.5" w="73.0" h="19.0"/>

<celldesigner:font size="12"/>

<celldesigner:view state="usual"/>

<celldesigner:usualView>

<celldesigner:innerPosition x="0.0" y="0.0"/>

<celldesigner:boxSize width="73.0" height="19.0"/>

<celldesigner:singleLine width="1.0"/>

<celldesigner:paint color="ffccff66" scheme="Color"/>

</celldesigner:usualView>

<celldesigner:briefView>

<celldesigner:innerPosition x="0.0" y="0.0"/>

<celldesigner:boxSize width="80.0" height="60.0"/>

<celldesigner:singleLine width="1.0"/>

<celldesigner:paint color="3fff0000" scheme="Color"/>

</celldesigner:briefView>

<celldesigner:info state="empty" angle="-1.5707963267948966"/>

</celldesigner:speciesAlias>

<celldesigner:speciesAlias id="sa400" species="s465">

<celldesigner:activity>inactive</celldesigner:activity>

<celldesigner:bounds x="83.5" y="1712.5" w="73.0" h="19.0"/>

<celldesigner:font size="12"/>

<celldesigner:view state="usual"/>

<celldesigner:usualView>

<celldesigner:innerPosition x="0.0" y="0.0"/>

<celldesigner:boxSize width="73.0" height="19.0"/>

<celldesigner:singleLine width="1.0"/>

<celldesigner:paint color="ffffff00" scheme="Color"/>

</celldesigner:usualView>

<celldesigner:briefView>

<celldesigner:innerPosition x="0.0" y="0.0"/>

<celldesigner:boxSize width="80.0" height="60.0"/>

<celldesigner:singleLine width="1.0"/>

<celldesigner:paint color="3fff0000" scheme="Color"/>

</celldesigner:briefView>

<celldesigner:info state="empty" angle="-1.5707963267948966"/>

</celldesigner:speciesAlias>

<celldesigner:speciesAlias id="sa401" species="s166">

<celldesigner:activity>inactive</celldesigner:activity>

<celldesigner:bounds x="82.5" y="897.5" w="65.0" h="17.0"/>

<celldesigner:font size="12"/>

<celldesigner:view state="usual"/>

<celldesigner:usualView>

<celldesigner:innerPosition x="0.0" y="0.0"/>

<celldesigner:boxSize width="65.0" height="17.0"/>

<celldesigner:singleLine width="1.0"/>

<celldesigner:paint color="ffccff66" scheme="Color"/>

</celldesigner:usualView>

<celldesigner:briefView>

<celldesigner:innerPosition x="0.0" y="0.0"/>

<celldesigner:boxSize width="80.0" height="60.0"/>

<celldesigner:singleLine width="1.0"/>

<celldesigner:paint color="3fff0000" scheme="Color"/>

</celldesigner:briefView>

<celldesigner:info state="empty" angle="-1.5707963267948966"/>

</celldesigner:speciesAlias>

<celldesigner:speciesAlias id="sa402" species="s167">

<celldesigner:activity>inactive</celldesigner:activity>

<celldesigner:bounds x="80.5" y="962.5" w="74.0" h="17.0"/>

<celldesigner:font size="12"/>

<celldesigner:view state="usual"/>

<celldesigner:usualView>

<celldesigner:innerPosition x="0.0" y="0.0"/>

<celldesigner:boxSize width="74.0" height="17.0"/>

<celldesigner:singleLine width="1.0"/>

<celldesigner:paint color="ffccff66" scheme="Color"/>

</celldesigner:usualView>

<celldesigner:briefView>

<celldesigner:innerPosition x="0.0" y="0.0"/>

<celldesigner:boxSize width="80.0" height="60.0"/>

<celldesigner:singleLine width="1.0"/>

<celldesigner:paint color="3fff0000" scheme="Color"/>

</celldesigner:briefView>

<celldesigner:info state="empty" angle="-1.5707963267948966"/>
</celldesigner:speciesAlias>
<celldesigner:speciesAlias id="sa403" species="s168">
<celldesigner:activity>inactive</celldesigner:activity>
<celldesigner:bounds x="81.5" y="1031.5" w="76.0" h="16.0"/>
<celldesigner:font size="12"/>
<celldesigner:view state="usual"/>
<celldesigner:usualView>
<celldesigner:innerPosition x="0.0" y="0.0"/>
<celldesigner:boxSize width="76.0" height="16.0"/>
<celldesigner:singleLine width="1.0"/>
<celldesigner:paint color="ffccff66" scheme="Color"/>
</celldesigner:usualView>
<celldesigner:briefView>
<celldesigner:innerPosition x="0.0" y="0.0"/>
<celldesigner:boxSize width="80.0" height="60.0"/>
<celldesigner:singleLine width="1.0"/>
<celldesigner:paint color="3fff0000" scheme="Color"/>
</celldesigner:briefView>
<celldesigner:info state="empty" angle="-1.5707963267948966"/>
</celldesigner:speciesAlias>
<celldesigner:speciesAlias id="sa404" species="s169">
<celldesigner:activity>inactive</celldesigner:activity>
<celldesigner:bounds x="92.0" y="1112.0" w="65.0" h="17.0"/>
<celldesigner:font size="12"/>
<celldesigner:view state="usual"/>
<celldesigner:usualView>

<celldesigner:innerPosition x="0.0" y="0.0"/>

<celldesigner:boxSize width="65.0" height="17.0"/>

<celldesigner:singleLine width="1.0"/>

<celldesigner:paint color="ffccff66" scheme="Color"/>

</celldesigner:usualView>

<celldesigner:briefView>

<celldesigner:innerPosition x="0.0" y="0.0"/>

<celldesigner:boxSize width="80.0" height="60.0"/>

<celldesigner:singleLine width="1.0"/>

<celldesigner:paint color="3fff0000" scheme="Color"/>

</celldesigner:briefView>

<celldesigner:info state="empty" angle="-1.5707963267948966"/>

</celldesigner:speciesAlias>

<celldesigner:speciesAlias id="sa405" species="s466">

<celldesigner:activity>inactive</celldesigner:activity>

<celldesigner:bounds x="83.5" y="1172.5" w="73.0" h="19.0"/>

<celldesigner:font size="12"/>

<celldesigner:view state="usual"/>

<celldesigner:usualView>

<celldesigner:innerPosition x="0.0" y="0.0"/>

<celldesigner:boxSize width="73.0" height="19.0"/>

<celldesigner:singleLine width="1.0"/>

<celldesigner:paint color="ffccff66" scheme="Color"/>

</celldesigner:usualView>

<celldesigner:briefView>

<celldesigner:innerPosition x="0.0" y="0.0"/>

<celldesigner:boxSize width="80.0" height="60.0"/>

<celldesigner:singleLine width="1.0"/>

<celldesigner:paint color="3fff0000" scheme="Color"/>

</celldesigner:briefView>

<celldesigner:info state="empty" angle="-1.5707963267948966"/>

</celldesigner:speciesAlias>

<celldesigner:speciesAlias id="sa406" species="s467">

<celldesigner:activity>inactive</celldesigner:activity>

<celldesigner:bounds x="97.5" y="1542.0" w="46.5" h="19.5"/>

<celldesigner:font size="12"/>

<celldesigner:view state="usual"/>

<celldesigner:usualView>

<celldesigner:innerPosition x="0.0" y="0.0"/>

<celldesigner:boxSize width="46.5" height="19.5"/>

<celldesigner:singleLine width="1.0"/>

<celldesigner:paint color="ffccff66" scheme="Color"/>

</celldesigner:usualView>

<celldesigner:briefView>

<celldesigner:innerPosition x="0.0" y="0.0"/>

<celldesigner:boxSize width="80.0" height="60.0"/>

<celldesigner:singleLine width="1.0"/>

<celldesigner:paint color="3fff0000" scheme="Color"/>

</celldesigner:briefView>

<celldesigner:info state="empty" angle="-1.5707963267948966"/>

</celldesigner:speciesAlias>

<celldesigner:speciesAlias id="sa407" species="s420">

<celldesigner:activity>inactive</celldesigner:activity>

<celldesigner:bounds x="955.5" y="82.0" w="49.0" h="16.0"/>

<celldesigner:font size="12"/>

<celldesigner:view state="usual"/>

<celldesigner:usualView>

<celldesigner:innerPosition x="0.0" y="0.0"/>

<celldesigner:boxSize width="49.0" height="16.0"/>

<celldesigner:singleLine width="1.0"/>

<celldesigner:paint color="ffccff66" scheme="Color"/>

</celldesigner:usualView>

<celldesigner:briefView>

<celldesigner:innerPosition x="0.0" y="0.0"/>

<celldesigner:boxSize width="80.0" height="60.0"/>

<celldesigner:singleLine width="1.0"/>

<celldesigner:paint color="3fff0000" scheme="Color"/>

</celldesigner:briefView>

<celldesigner:info state="empty" angle="-1.5707963267948966"/>

</celldesigner:speciesAlias>

<celldesigner:speciesAlias id="sa408" species="s62">

<celldesigner:activity>inactive</celldesigner:activity>

<celldesigner:bounds x="778.0" y="71.5" w="64.0" h="17.0"/>

<celldesigner:font size="12"/>

<celldesigner:view state="usual"/>

<celldesigner:usualView>

<celldesigner:innerPosition x="0.0" y="0.0"/>

<celldesigner:boxSize width="64.0" height="17.0"/>

<celldesigner:singleLine width="1.0"/>

<celldesigner:paint color="ffccff66" scheme="Color"/>

</celldesigner:usualView>

<celldesigner:briefView>

<celldesigner:innerPosition x="0.0" y="0.0"/>

<celldesigner:boxSize width="80.0" height="60.0"/>

<celldesigner:singleLine width="1.0"/>

<celldesigner:paint color="3fff0000" scheme="Color"/>

</celldesigner:briefView>

<celldesigner:info state="empty" angle="-1.5707963267948966"/>

</celldesigner:speciesAlias>

<celldesigner:speciesAlias id="sa409" species="s61">

<celldesigner:activity>inactive</celldesigner:activity>

<celldesigner:bounds x="907.5" y="38.75" w="45.0" h="22.5"/>

<celldesigner:font size="12"/>

<celldesigner:view state="usual"/>

<celldesigner:usualView>

<celldesigner:innerPosition x="0.0" y="0.0"/>

<celldesigner:boxSize width="45.0" height="22.5"/>

<celldesigner:singleLine width="1.0"/>

<celldesigner:paint color="ffccff66" scheme="Color"/>

</celldesigner:usualView>

<celldesigner:briefView>

<celldesigner:innerPosition x="0.0" y="0.0"/>

<celldesigner:boxSize width="80.0" height="60.0"/>

<celldesigner:singleLine width="1.0"/>

<celldesigner:paint color="3fff0000" scheme="Color"/>

</celldesigner:briefView>

<celldesigner:info state="empty" angle="-1.5707963267948966"/>

</celldesigner:speciesAlias>

```
<celldesigner:speciesAlias id="sa410" species="s120">
<celldesigner:activity>inactive</celldesigner:activity>
<celldesigner:bounds x="712.75" y="77.25" w="66.5" h="19.5"/>
<celldesigner:font size="12"/>
<celldesigner:view state="usual"/>
<celldesigner:usualView>
<celldesigner:innerPosition x="0.0" y="0.0"/>
<celldesigner:boxSize width="66.5" height="19.5"/>
<celldesigner:singleLine width="1.0"/>
<celldesigner:paint color="ffccff66" scheme="Color"/>
</celldesigner:usualView>
<celldesigner:briefView>
<celldesigner:innerPosition x="0.0" y="0.0"/>
<celldesigner:boxSize width="80.0" height="60.0"/>
<celldesigner:singleLine width="1.0"/>
<celldesigner:paint color="3fff0000" scheme="Color"/>
</celldesigner:briefView>
<celldesigner:info state="empty" angle="-1.5707963267948966"/>
</celldesigner:speciesAlias>
<celldesigner:speciesAlias id="sa411" species="s175">
<celldesigner:activity>inactive</celldesigner:activity>
<celldesigner:bounds x="904.5" y="121.0" w="51.0" h="18.0"/>
<celldesigner:font size="12"/>
<celldesigner:view state="usual"/>
<celldesigner:usualView>
<celldesigner:innerPosition x="0.0" y="0.0"/>
<celldesigner:boxSize width="51.0" height="18.0"/>
```

<celldesigner:singleLine width="1.0"/>

<celldesigner:paint color="ffccff66" scheme="Color"/>

</celldesigner:usualView>

<celldesigner:briefView>

<celldesigner:innerPosition x="0.0" y="0.0"/>

<celldesigner:boxSize width="80.0" height="60.0"/>

<celldesigner:singleLine width="1.0"/>

<celldesigner:paint color="3fff0000" scheme="Color"/>

</celldesigner:briefView>

<celldesigner:info state="empty" angle="-1.5707963267948966"/>

</celldesigner:speciesAlias>

<celldesigner:speciesAlias id="sa412" species="s139">

<celldesigner:activity>inactive</celldesigner:activity>

<celldesigner:bounds x="1044.5" y="111.0" w="51.0" h="18.0"/>

<celldesigner:font size="12"/>

<celldesigner:view state="usual"/>

<celldesigner:usualView>

<celldesigner:innerPosition x="0.0" y="0.0"/>

<celldesigner:boxSize width="51.0" height="18.0"/>

<celldesigner:singleLine width="1.0"/>

<celldesigner:paint color="ffffff33" scheme="Color"/>

</celldesigner:usualView>

<celldesigner:briefView>

<celldesigner:innerPosition x="0.0" y="0.0"/>

<celldesigner:boxSize width="80.0" height="60.0"/>

<celldesigner:singleLine width="1.0"/>

<celldesigner:paint color="3fff0000" scheme="Color"/>

</celldesigner:briefView>

<celldesigner:info state="empty" angle="-1.5707963267948966"/>

</celldesigner:speciesAlias>

<celldesigner:speciesAlias id="sa413" species="s140">

<celldesigner:activity>inactive</celldesigner:activity>

<celldesigner:bounds x="1091.5" y="170.0" w="37.0" h="20.0"/>

<celldesigner:font size="12"/>

<celldesigner:view state="usual"/>

<celldesigner:usualView>

<celldesigner:innerPosition x="0.0" y="0.0"/>

<celldesigner:boxSize width="37.0" height="20.0"/>

<celldesigner:singleLine width="1.0"/>

<celldesigner:paint color="ffccff66" scheme="Color"/>

</celldesigner:usualView>

<celldesigner:briefView>

<celldesigner:innerPosition x="0.0" y="0.0"/>

<celldesigner:boxSize width="80.0" height="60.0"/>

<celldesigner:singleLine width="1.0"/>

<celldesigner:paint color="3fff0000" scheme="Color"/>

</celldesigner:briefView>

<celldesigner:info state="empty" angle="-1.5707963267948966"/>

</celldesigner:speciesAlias>

<celldesigner:speciesAlias id="sa414" species="s137">

<celldesigner:activity>inactive</celldesigner:activity>

<celldesigner:bounds x="1104.5" y="211.0" w="51.0" h="18.0"/>

<celldesigner:font size="12"/>

<celldesigner:view state="usual"/>

<celldesigner:usualView>

<celldesigner:innerPosition x="0.0" y="0.0"/>

<celldesigner:boxSize width="51.0" height="18.0"/>

<celldesigner:singleLine width="1.0"/>

<celldesigner:paint color="ffffff33" scheme="Color"/>

</celldesigner:usualView>

<celldesigner:briefView>

<celldesigner:innerPosition x="0.0" y="0.0"/>

<celldesigner:boxSize width="80.0" height="60.0"/>

<celldesigner:singleLine width="1.0"/>

<celldesigner:paint color="3fff0000" scheme="Color"/>

</celldesigner:briefView>

<celldesigner:info state="empty" angle="-1.5707963267948966"/>

</celldesigner:speciesAlias>

<celldesigner:speciesAlias id="sa415" species="s176">

<celldesigner:activity>inactive</celldesigner:activity>

<celldesigner:bounds x="917.5" y="201.0" w="45.0" h="18.0"/>

<celldesigner:font size="12"/>

<celldesigner:view state="usual"/>

<celldesigner:usualView>

<celldesigner:innerPosition x="0.0" y="0.0"/>

<celldesigner:boxSize width="45.0" height="18.0"/>

<celldesigner:singleLine width="1.0"/>

<celldesigner:paint color="ffccff66" scheme="Color"/>

</celldesigner:usualView>

<celldesigner:briefView>

<celldesigner:innerPosition x="0.0" y="0.0"/>

<celldesigner:boxSize width="80.0" height="60.0"/>
<celldesigner:singleLine width="1.0"/>
<celldesigner:paint color="3fff0000" scheme="Color"/>
</celldesigner:briefView>
<celldesigner:info state="empty" angle="-1.5707963267948966"/>
</celldesigner:speciesAlias>
<celldesigner:speciesAlias id="sa416" species="s135">
<celldesigner:activity>inactive</celldesigner:activity>
<celldesigner:bounds x="990.0" y="201.5" w="60.0" h="17.0"/>
<celldesigner:font size="12"/>
<celldesigner:view state="usual"/>
<celldesigner:usualView>
<celldesigner:innerPosition x="0.0" y="0.0"/>
<celldesigner:boxSize width="60.0" height="17.0"/>
<celldesigner:singleLine width="1.0"/>
<celldesigner:paint color="ffccff66" scheme="Color"/>
</celldesigner:usualView>
<celldesigner:briefView>
<celldesigner:innerPosition x="0.0" y="0.0"/>
<celldesigner:boxSize width="80.0" height="60.0"/>
<celldesigner:singleLine width="1.0"/>
<celldesigner:paint color="3fff0000" scheme="Color"/>
</celldesigner:briefView>
<celldesigner:info state="empty" angle="-1.5707963267948966"/>
</celldesigner:speciesAlias>
<celldesigner:speciesAlias id="sa417" species="s1101">
<celldesigner:activity>inactive</celldesigner:activity>

<celldesigner:bounds x="834.5" y="141.0" w="51.0" h="18.0"/>

<celldesigner:font size="12"/>

<celldesigner:view state="usual"/>

<celldesigner:usualView>

<celldesigner:innerPosition x="0.0" y="0.0"/>

<celldesigner:boxSize width="51.0" height="18.0"/>

<celldesigner:singleLine width="1.0"/>

<celldesigner:paint color="ffccff66" scheme="Color"/>

</celldesigner:usualView>

<celldesigner:briefView>

<celldesigner:innerPosition x="0.0" y="0.0"/>

<celldesigner:boxSize width="80.0" height="60.0"/>

<celldesigner:singleLine width="1.0"/>

<celldesigner:paint color="3fff0000" scheme="Color"/>

</celldesigner:briefView>

<celldesigner:info state="empty" angle="-1.5707963267948966"/>

</celldesigner:speciesAlias>

<celldesigner:speciesAlias id="sa418" species="s138">

<celldesigner:activity>inactive</celldesigner:activity>

<celldesigner:bounds x="1056.5" y="241.5" w="47.0" h="17.0"/>

<celldesigner:font size="12"/>

<celldesigner:view state="usual"/>

<celldesigner:usualView>

<celldesigner:innerPosition x="0.0" y="0.0"/>

<celldesigner:boxSize width="47.0" height="17.0"/>

<celldesigner:singleLine width="1.0"/>

<celldesigner:paint color="ffccff66" scheme="Color"/>

</celldesigner:usualView>

<celldesigner:briefView>

<celldesigner:innerPosition x="0.0" y="0.0"/>

<celldesigner:boxSize width="80.0" height="60.0"/>

<celldesigner:singleLine width="1.0"/>

<celldesigner:paint color="3fff0000" scheme="Color"/>

</celldesigner:briefView>

<celldesigner:info state="empty" angle="-1.5707963267948966"/>

</celldesigner:speciesAlias>

<celldesigner:speciesAlias id="sa419" species="s118">

<celldesigner:activity>inactive</celldesigner:activity>

<celldesigner:bounds x="1094.5" y="331.0" w="51.0" h="18.0"/>

<celldesigner:font size="12"/>

<celldesigner:view state="usual"/>

<celldesigner:usualView>

<celldesigner:innerPosition x="0.0" y="0.0"/>

<celldesigner:boxSize width="51.0" height="18.0"/>

<celldesigner:singleLine width="1.0"/>

<celldesigner:paint color="ffffff33" scheme="Color"/>

</celldesigner:usualView>

<celldesigner:briefView>

<celldesigner:innerPosition x="0.0" y="0.0"/>

<celldesigner:boxSize width="80.0" height="60.0"/>

<celldesigner:singleLine width="1.0"/>

<celldesigner:paint color="3fff0000" scheme="Color"/>

</celldesigner:briefView>

<celldesigner:info state="empty" angle="-1.5707963267948966"/>

</celldesigner:speciesAlias>

<celldesigner:speciesAlias id="sa420" species="s177">

<celldesigner:activity>inactive</celldesigner:activity>

<celldesigner:bounds x="920.5" y="328.0" w="51.0" h="18.0"/>

<celldesigner:font size="12"/>

<celldesigner:view state="usual"/>

<celldesigner:usualView>

<celldesigner:innerPosition x="0.0" y="0.0"/>

<celldesigner:boxSize width="51.0" height="18.0"/>

<celldesigner:singleLine width="1.0"/>

<celldesigner:paint color="ffffff33" scheme="Color"/>

</celldesigner:usualView>

<celldesigner:briefView>

<celldesigner:innerPosition x="0.0" y="0.0"/>

<celldesigner:boxSize width="80.0" height="60.0"/>

<celldesigner:singleLine width="1.0"/>

<celldesigner:paint color="3fff0000" scheme="Color"/>

</celldesigner:briefView>

<celldesigner:info state="empty" angle="-1.5707963267948966"/>

</celldesigner:speciesAlias>

<celldesigner:speciesAlias id="sa421" species="s119">

<celldesigner:activity>inactive</celldesigner:activity>

<celldesigner:bounds x="794.5" y="321.0" w="51.0" h="18.0"/>

<celldesigner:font size="12"/>

<celldesigner:view state="usual"/>

<celldesigner:usualView>

<celldesigner:innerPosition x="0.0" y="0.0"/>

<celldesigner:boxSize width="51.0" height="18.0"/>

<celldesigner:singleLine width="1.0"/>

<celldesigner:paint color="ffffff33" scheme="Color"/>

</celldesigner:usualView>

<celldesigner:briefView>

<celldesigner:innerPosition x="0.0" y="0.0"/>

<celldesigner:boxSize width="80.0" height="60.0"/>

<celldesigner:singleLine width="1.0"/>

<celldesigner:paint color="3fff0000" scheme="Color"/>

</celldesigner:briefView>

<celldesigner:info state="empty" angle="-1.5707963267948966"/>

</celldesigner:speciesAlias>

<celldesigner:speciesAlias id="sa422" species="s120">

<celldesigner:activity>inactive</celldesigner:activity>

<celldesigner:bounds x="724.875" y="389.125" w="70.25" h="21.75"/>

<celldesigner:font size="12"/>

<celldesigner:view state="usual"/>

<celldesigner:usualView>

<celldesigner:innerPosition x="0.0" y="0.0"/>

<celldesigner:boxSize width="70.25" height="21.75"/>

<celldesigner:singleLine width="1.0"/>

<celldesigner:paint color="ffccff66" scheme="Color"/>

</celldesigner:usualView>

<celldesigner:briefView>

<celldesigner:innerPosition x="0.0" y="0.0"/>

<celldesigner:boxSize width="80.0" height="60.0"/>

<celldesigner:singleLine width="1.0"/>

<celldesigner:paint color="3fff0000" scheme="Color"/>
</celldesigner:briefView>
<celldesigner:info state="empty" angle="-1.5707963267948966"/>
</celldesigner:speciesAlias>
<celldesigner:speciesAlias id="sa423" species="s125">
<celldesigner:activity>inactive</celldesigner:activity>
<celldesigner:bounds x="1094.5" y="381.0" w="51.0" h="18.0"/>
<celldesigner:font size="12"/>
<celldesigner:view state="usual"/>
<celldesigner:usualView>
<celldesigner:innerPosition x="0.0" y="0.0"/>
<celldesigner:boxSize width="51.0" height="18.0"/>
<celldesigner:singleLine width="1.0"/>
<celldesigner:paint color="ffccff66" scheme="Color"/>
</celldesigner:usualView>
<celldesigner:briefView>
<celldesigner:innerPosition x="0.0" y="0.0"/>
<celldesigner:boxSize width="80.0" height="60.0"/>
<celldesigner:singleLine width="1.0"/>
<celldesigner:paint color="3fff0000" scheme="Color"/>
</celldesigner:briefView>
<celldesigner:info state="empty" angle="-1.5707963267948966"/>
</celldesigner:speciesAlias>
<celldesigner:speciesAlias id="sa424" species="s122">
<celldesigner:activity>inactive</celldesigner:activity>
<celldesigner:bounds x="984.5" y="361.0" w="51.0" h="18.0"/>
<celldesigner:font size="12"/>

<celldesigner:view state="usual"/>

<celldesigner:usualView>

<celldesigner:innerPosition x="0.0" y="0.0"/>

<celldesigner:boxSize width="51.0" height="18.0"/>

<celldesigner:singleLine width="1.0"/>

<celldesigner:paint color="ffccff66" scheme="Color"/>

</celldesigner:usualView>

<celldesigner:briefView>

<celldesigner:innerPosition x="0.0" y="0.0"/>

<celldesigner:boxSize width="80.0" height="60.0"/>

<celldesigner:singleLine width="1.0"/>

<celldesigner:paint color="3fff0000" scheme="Color"/>

</celldesigner:briefView>

<celldesigner:info state="empty" angle="-1.5707963267948966"/>

</celldesigner:speciesAlias>

<celldesigner:speciesAlias id="sa425" species="s422">

<celldesigner:activity>inactive</celldesigner:activity>

<celldesigner:bounds x="858.5" y="550.0" w="83.0" h="20.0"/>

<celldesigner:font size="12"/>

<celldesigner:view state="usual"/>

<celldesigner:usualView>

<celldesigner:innerPosition x="0.0" y="0.0"/>

<celldesigner:boxSize width="83.0" height="20.0"/>

<celldesigner:singleLine width="1.0"/>

<celldesigner:paint color="ffccff66" scheme="Color"/>

</celldesigner:usualView>

<celldesigner:briefView>

<celldesigner:innerPosition x="0.0" y="0.0"/>
<celldesigner:boxSize width="80.0" height="60.0"/>
<celldesigner:singleLine width="1.0"/>
<celldesigner:paint color="3fff0000" scheme="Color"/>
</celldesigner:briefView>
<celldesigner:info state="empty" angle="-1.5707963267948966"/>
</celldesigner:speciesAlias>
<celldesigner:speciesAlias id="sa426" species="s423">
<celldesigner:activity>inactive</celldesigner:activity>
<celldesigner:bounds x="826.5" y="410.5" w="67.0" h="19.0"/>
<celldesigner:font size="12"/>
<celldesigner:view state="usual"/>
<celldesigner:usualView>
<celldesigner:innerPosition x="0.0" y="0.0"/>
<celldesigner:boxSize width="67.0" height="19.0"/>
<celldesigner:singleLine width="1.0"/>
<celldesigner:paint color="ffccff66" scheme="Color"/>
</celldesigner:usualView>
<celldesigner:briefView>
<celldesigner:innerPosition x="0.0" y="0.0"/>
<celldesigner:boxSize width="80.0" height="60.0"/>
<celldesigner:singleLine width="1.0"/>
<celldesigner:paint color="3fff0000" scheme="Color"/>
</celldesigner:briefView>
<celldesigner:info state="empty" angle="-1.5707963267948966"/>
</celldesigner:speciesAlias>
<celldesigner:speciesAlias id="sa427" species="s121">

<celldesigner:activity>inactive</celldesigner:activity>

<celldesigner:bounds x="914.5" y="401.0" w="51.0" h="18.0"/>

<celldesigner:font size="12"/>

<celldesigner:view state="usual"/>

<celldesigner:usualView>

<celldesigner:innerPosition x="0.0" y="0.0"/>

<celldesigner:boxSize width="51.0" height="18.0"/>

<celldesigner:singleLine width="1.0"/>

<celldesigner:paint color="ffffff33" scheme="Color"/>

</celldesigner:usualView>

<celldesigner:briefView>

<celldesigner:innerPosition x="0.0" y="0.0"/>

<celldesigner:boxSize width="80.0" height="60.0"/>

<celldesigner:singleLine width="1.0"/>

<celldesigner:paint color="3fff0000" scheme="Color"/>

</celldesigner:briefView>

<celldesigner:info state="empty" angle="-1.5707963267948966"/>

</celldesigner:speciesAlias>

<celldesigner:speciesAlias id="sa428" species="s128">

<celldesigner:activity>inactive</celldesigner:activity>

<celldesigner:bounds x="914.5" y="461.0" w="51.0" h="18.0"/>

<celldesigner:font size="12"/>

<celldesigner:view state="usual"/>

<celldesigner:usualView>

<celldesigner:innerPosition x="0.0" y="0.0"/>

<celldesigner:boxSize width="51.0" height="18.0"/>

<celldesigner:singleLine width="1.0"/>

<celldesigner:paint color="ffccff66" scheme="Color"/>

</celldesigner:usualView>

<celldesigner:briefView>

<celldesigner:innerPosition x="0.0" y="0.0"/>

<celldesigner:boxSize width="80.0" height="60.0"/>

<celldesigner:singleLine width="1.0"/>

<celldesigner:paint color="3fff0000" scheme="Color"/>

</celldesigner:briefView>

<celldesigner:info state="empty" angle="-1.5707963267948966"/>

</celldesigner:speciesAlias>

<celldesigner:speciesAlias id="sa429" species="s424">

<celldesigner:activity>inactive</celldesigner:activity>

<celldesigner:bounds x="833.0" y="481.5" w="54.0" h="17.0"/>

<celldesigner:font size="12"/>

<celldesigner:view state="usual"/>

<celldesigner:usualView>

<celldesigner:innerPosition x="0.0" y="0.0"/>

<celldesigner:boxSize width="54.0" height="17.0"/>

<celldesigner:singleLine width="1.0"/>

<celldesigner:paint color="ffccff66" scheme="Color"/>

</celldesigner:usualView>

<celldesigner:briefView>

<celldesigner:innerPosition x="0.0" y="0.0"/>

<celldesigner:boxSize width="80.0" height="60.0"/>

<celldesigner:singleLine width="1.0"/>

<celldesigner:paint color="3fff0000" scheme="Color"/>

</celldesigner:briefView>

<celldesigner:info state="empty" angle="-1.5707963267948966"/>
</celldesigner:speciesAlias>
<celldesigner:speciesAlias id="sa430" species="s131">
<celldesigner:activity>inactive</celldesigner:activity>
<celldesigner:bounds x="994.5" y="461.0" w="51.0" h="18.0"/>
<celldesigner:font size="12"/>
<celldesigner:view state="usual"/>
<celldesigner:usualView>
<celldesigner:innerPosition x="0.0" y="0.0"/>
<celldesigner:boxSize width="51.0" height="18.0"/>
<celldesigner:singleLine width="1.0"/>
<celldesigner:paint color="ffccff66" scheme="Color"/>
</celldesigner:usualView>
<celldesigner:briefView>
<celldesigner:innerPosition x="0.0" y="0.0"/>
<celldesigner:boxSize width="80.0" height="60.0"/>
<celldesigner:singleLine width="1.0"/>
<celldesigner:paint color="3fff0000" scheme="Color"/>
</celldesigner:briefView>
<celldesigner:info state="empty" angle="-1.5707963267948966"/>
</celldesigner:speciesAlias>
<celldesigner:speciesAlias id="sa431" species="s132">
<celldesigner:activity>inactive</celldesigner:activity>
<celldesigner:bounds x="974.5" y="521.0" w="51.0" h="18.0"/>
<celldesigner:font size="12"/>
<celldesigner:view state="usual"/>
<celldesigner:usualView>

<celldesigner:innerPosition x="0.0" y="0.0"/>

<celldesigner:boxSize width="51.0" height="18.0"/>

<celldesigner:singleLine width="1.0"/>

<celldesigner:paint color="ffffff33" scheme="Color"/>

</celldesigner:usualView>

<celldesigner:briefView>

<celldesigner:innerPosition x="0.0" y="0.0"/>

<celldesigner:boxSize width="80.0" height="60.0"/>

<celldesigner:singleLine width="1.0"/>

<celldesigner:paint color="3fff0000" scheme="Color"/>

</celldesigner:briefView>

<celldesigner:info state="empty" angle="-1.5707963267948966"/>

</celldesigner:speciesAlias>

<celldesigner:speciesAlias id="sa432" species="s426">

<celldesigner:activity>inactive</celldesigner:activity>

<celldesigner:bounds x="765.5" y="232.0" w="49.0" h="16.0"/>

<celldesigner:font size="12"/>

<celldesigner:view state="usual"/>

<celldesigner:usualView>

<celldesigner:innerPosition x="0.0" y="0.0"/>

<celldesigner:boxSize width="49.0" height="16.0"/>

<celldesigner:singleLine width="1.0"/>

<celldesigner:paint color="ffccff66" scheme="Color"/>

</celldesigner:usualView>

<celldesigner:briefView>

<celldesigner:innerPosition x="0.0" y="0.0"/>

<celldesigner:boxSize width="80.0" height="60.0"/>

<celldesigner:singleLine width="1.0"/>

<celldesigner:paint color="3fff0000" scheme="Color"/>

</celldesigner:briefView>

<celldesigner:info state="empty" angle="-1.5707963267948966"/>

</celldesigner:speciesAlias>

<celldesigner:speciesAlias id="sa433" species="s427">

<celldesigner:activity>inactive</celldesigner:activity>

<celldesigner:bounds x="966.5" y="570.5" w="67.0" h="19.0"/>

<celldesigner:font size="12"/>

<celldesigner:view state="usual"/>

<celldesigner:usualView>

<celldesigner:innerPosition x="0.0" y="0.0"/>

<celldesigner:boxSize width="67.0" height="19.0"/>

<celldesigner:singleLine width="1.0"/>

<celldesigner:paint color="ffccff66" scheme="Color"/>

</celldesigner:usualView>

<celldesigner:briefView>

<celldesigner:innerPosition x="0.0" y="0.0"/>

<celldesigner:boxSize width="80.0" height="60.0"/>

<celldesigner:singleLine width="1.0"/>

<celldesigner:paint color="3fff0000" scheme="Color"/>

</celldesigner:briefView>

<celldesigner:info state="empty" angle="-1.5707963267948966"/>

</celldesigner:speciesAlias>

<celldesigner:speciesAlias id="sa434" species="s428">

<celldesigner:activity>inactive</celldesigner:activity>

<celldesigner:bounds x="953.0" y="641.5" w="54.0" h="17.0"/>

<celldesigner:font size="12"/>

<celldesigner:view state="usual"/>

<celldesigner:usualView>

<celldesigner:innerPosition x="0.0" y="0.0"/>

<celldesigner:boxSize width="54.0" height="17.0"/>

<celldesigner:singleLine width="1.0"/>

<celldesigner:paint color="ffccff66" scheme="Color"/>

</celldesigner:usualView>

<celldesigner:briefView>

<celldesigner:innerPosition x="0.0" y="0.0"/>

<celldesigner:boxSize width="80.0" height="60.0"/>

<celldesigner:singleLine width="1.0"/>

<celldesigner:paint color="3fff0000" scheme="Color"/>

</celldesigner:briefView>

<celldesigner:info state="empty" angle="-1.5707963267948966"/>

</celldesigner:speciesAlias>

<celldesigner:speciesAlias id="sa435" species="s120">

<celldesigner:activity>inactive</celldesigner:activity>

<celldesigner:bounds x="881.25" y="611.0" w="77.5" h="18.0"/>

<celldesigner:font size="12"/>

<celldesigner:view state="usual"/>

<celldesigner:usualView>

<celldesigner:innerPosition x="0.0" y="0.0"/>

<celldesigner:boxSize width="77.5" height="18.0"/>

<celldesigner:singleLine width="1.0"/>

<celldesigner:paint color="ffccff66" scheme="Color"/>

</celldesigner:usualView>

<celldesigner:briefView>

<celldesigner:innerPosition x="0.0" y="0.0"/>

<celldesigner:boxSize width="80.0" height="60.0"/>

<celldesigner:singleLine width="1.0"/>

<celldesigner:paint color="3fff0000" scheme="Color"/>

</celldesigner:briefView>

<celldesigner:info state="empty" angle="-1.5707963267948966"/>

</celldesigner:speciesAlias>

<celldesigner:speciesAlias id="sa437" species="s90">

<celldesigner:activity>inactive</celldesigner:activity>

<celldesigner:bounds x="904.0" y="669.5" w="32.0" h="21.0"/>

<celldesigner:font size="12"/>

<celldesigner:view state="usual"/>

<celldesigner:usualView>

<celldesigner:innerPosition x="0.0" y="0.0"/>

<celldesigner:boxSize width="32.0" height="21.0"/>

<celldesigner:singleLine width="1.0"/>

<celldesigner:paint color="ffcc0066" scheme="Color"/>

</celldesigner:usualView>

<celldesigner:briefView>

<celldesigner:innerPosition x="0.0" y="0.0"/>

<celldesigner:boxSize width="80.0" height="60.0"/>

<celldesigner:singleLine width="1.0"/>

<celldesigner:paint color="3fff0000" scheme="Color"/>

</celldesigner:briefView>

<celldesigner:info state="empty" angle="-1.5707963267948966"/>

</celldesigner:speciesAlias>

<celldesigner:speciesAlias id="sa438" species="s478">

<celldesigner:activity>inactive</celldesigner:activity>

<celldesigner:bounds x="897.5" y="748.75" w="45.0" h="22.5"/>

<celldesigner:font size="12"/>

<celldesigner:view state="usual"/>

<celldesigner:usualView>

<celldesigner:innerPosition x="0.0" y="0.0"/>

<celldesigner:boxSize width="45.0" height="22.5"/>

<celldesigner:singleLine width="1.0"/>

<celldesigner:paint color="ffccff66" scheme="Color"/>

</celldesigner:usualView>

<celldesigner:briefView>

<celldesigner:innerPosition x="0.0" y="0.0"/>

<celldesigner:boxSize width="80.0" height="60.0"/>

<celldesigner:singleLine width="1.0"/>

<celldesigner:paint color="3fff0000" scheme="Color"/>

</celldesigner:briefView>

<celldesigner:info state="empty" angle="-1.5707963267948966"/>

</celldesigner:speciesAlias>

<celldesigner:speciesAlias id="sa439" species="s479">

<celldesigner:activity>inactive</celldesigner:activity>

<celldesigner:bounds x="827.5" y="738.75" w="45.0" h="22.5"/>

<celldesigner:font size="12"/>

<celldesigner:view state="usual"/>

<celldesigner:usualView>

<celldesigner:innerPosition x="0.0" y="0.0"/>

<celldesigner:boxSize width="45.0" height="22.5"/>

<celldesigner:singleLine width="1.0"/>

<celldesigner:paint color="ffccff66" scheme="Color"/>

</celldesigner:usualView>

<celldesigner:briefView>

<celldesigner:innerPosition x="0.0" y="0.0"/>

<celldesigner:boxSize width="80.0" height="60.0"/>

<celldesigner:singleLine width="1.0"/>

<celldesigner:paint color="3fff0000" scheme="Color"/>

</celldesigner:briefView>

<celldesigner:info state="empty" angle="-1.5707963267948966"/>

</celldesigner:speciesAlias>

<celldesigner:speciesAlias id="sa440" species="s471">

<celldesigner:activity>inactive</celldesigner:activity>

<celldesigner:bounds x="1047.5" y="718.75" w="45.0" h="22.5"/>

<celldesigner:font size="12"/>

<celldesigner:view state="usual"/>

<celldesigner:usualView>

<celldesigner:innerPosition x="0.0" y="0.0"/>

<celldesigner:boxSize width="45.0" height="22.5"/>

<celldesigner:singleLine width="1.0"/>

<celldesigner:paint color="ffccff66" scheme="Color"/>

</celldesigner:usualView>

<celldesigner:briefView>

<celldesigner:innerPosition x="0.0" y="0.0"/>

<celldesigner:boxSize width="80.0" height="60.0"/>

<celldesigner:singleLine width="1.0"/>

<celldesigner:paint color="3fff0000" scheme="Color"/>

</celldesigner:briefView>

<celldesigner:info state="empty" angle="-1.5707963267948966"/>

</celldesigner:speciesAlias>

<celldesigner:speciesAlias id="sa441" species="s108">

<celldesigner:activity>inactive</celldesigner:activity>

<celldesigner:bounds x="953.75" y="719.375" w="52.5" h="21.25"/>

<celldesigner:font size="12"/>

<celldesigner:view state="usual"/>

<celldesigner:usualView>

<celldesigner:innerPosition x="0.0" y="0.0"/>

<celldesigner:boxSize width="52.5" height="21.25"/>

<celldesigner:singleLine width="1.0"/>

<celldesigner:paint color="ffccff66" scheme="Color"/>

</celldesigner:usualView>

<celldesigner:briefView>

<celldesigner:innerPosition x="0.0" y="0.0"/>

<celldesigner:boxSize width="80.0" height="60.0"/>

<celldesigner:singleLine width="1.0"/>

<celldesigner:paint color="3fff0000" scheme="Color"/>

</celldesigner:briefView>

<celldesigner:info state="empty" angle="-1.5707963267948966"/>

</celldesigner:speciesAlias>

<celldesigner:speciesAlias id="sa442" species="s480">

<celldesigner:activity>inactive</celldesigner:activity>

<celldesigner:bounds x="948.75" y="769.375" w="62.5" h="21.25"/>

<celldesigner:font size="12"/>

<celldesigner:view state="usual"/>

<celldesigner:usualView>

<celldesigner:innerPosition x="0.0" y="0.0"/>

<celldesigner:boxSize width="62.5" height="21.25"/>

<celldesigner:singleLine width="1.0"/>

<celldesigner:paint color="ffccff66" scheme="Color"/>

</celldesigner:usualView>

<celldesigner:briefView>

<celldesigner:innerPosition x="0.0" y="0.0"/>

<celldesigner:boxSize width="80.0" height="60.0"/>

<celldesigner:singleLine width="1.0"/>

<celldesigner:paint color="3fff0000" scheme="Color"/>

</celldesigner:briefView>

<celldesigner:info state="empty" angle="-1.5707963267948966"/>

</celldesigner:speciesAlias>

<celldesigner:speciesAlias id="sa443" species="s481">

<celldesigner:activity>inactive</celldesigner:activity>

<celldesigner:bounds x="897.5" y="808.75" w="45.0" h="22.5"/>

<celldesigner:font size="12"/>

<celldesigner:view state="usual"/>

<celldesigner:usualView>

<celldesigner:innerPosition x="0.0" y="0.0"/>

<celldesigner:boxSize width="45.0" height="22.5"/>

<celldesigner:singleLine width="1.0"/>

<celldesigner:paint color="ffccff66" scheme="Color"/>

</celldesigner:usualView>

<celldesigner:briefView>

<celldesigner:innerPosition x="0.0" y="0.0"/>

<celldesigner:boxSize width="80.0" height="60.0"/>

<celldesigner:singleLine width="1.0"/>

<celldesigner:paint color="3fff0000" scheme="Color"/>

</celldesigner:briefView>

<celldesigner:info state="empty" angle="-1.5707963267948966"/>

</celldesigner:speciesAlias>

<celldesigner:speciesAlias id="sa444" species="s482">

<celldesigner:activity>inactive</celldesigner:activity>

<celldesigner:bounds x="738.75" y="969.375" w="62.5" h="21.25"/>

<celldesigner:font size="12"/>

<celldesigner:view state="usual"/>

<celldesigner:usualView>

<celldesigner:innerPosition x="0.0" y="0.0"/>

<celldesigner:boxSize width="62.5" height="21.25"/>

<celldesigner:singleLine width="1.0"/>

<celldesigner:paint color="ffccff66" scheme="Color"/>

</celldesigner:usualView>

<celldesigner:briefView>

<celldesigner:innerPosition x="0.0" y="0.0"/>

<celldesigner:boxSize width="80.0" height="60.0"/>

<celldesigner:singleLine width="1.0"/>

<celldesigner:paint color="3fff0000" scheme="Color"/>

</celldesigner:briefView>

<celldesigner:info state="empty" angle="-1.5707963267948966"/>

</celldesigner:speciesAlias>

<celldesigner:speciesAlias id="sa446" species="s483">

<celldesigner:activity>inactive</celldesigner:activity>

<celldesigner:bounds x="759.375" y="839.6875" w="41.25" h="20.625"/>

<celldesigner:font size="12"/>

<celldesigner:view state="usual"/>

<celldesigner:usualView>

<celldesigner:innerPosition x="0.0" y="0.0"/>

<celldesigner:boxSize width="41.25" height="20.625"/>

<celldesigner:singleLine width="1.0"/>

<celldesigner:paint color="ffccff66" scheme="Color"/>

</celldesigner:usualView>

<celldesigner:briefView>

<celldesigner:innerPosition x="0.0" y="0.0"/>

<celldesigner:boxSize width="80.0" height="60.0"/>

<celldesigner:singleLine width="1.0"/>

<celldesigner:paint color="3fff0000" scheme="Color"/>

</celldesigner:briefView>

<celldesigner:info state="empty" angle="-1.5707963267948966"/>

</celldesigner:speciesAlias>

<celldesigner:speciesAlias id="sa447" species="s485">

<celldesigner:activity>inactive</celldesigner:activity>

<celldesigner:bounds x="947.5" y="848.75" w="45.0" h="22.5"/>

<celldesigner:font size="12"/>

<celldesigner:view state="usual"/>

<celldesigner:usualView>

<celldesigner:innerPosition x="0.0" y="0.0"/>

<celldesigner:boxSize width="45.0" height="22.5"/>

<celldesigner:singleLine width="1.0"/>

<celldesigner:paint color="ffccff66" scheme="Color"/>

</celldesigner:usualView>
<celldesigner:briefView>
<celldesigner:innerPosition x="0.0" y="0.0"/>
<celldesigner:boxSize width="80.0" height="60.0"/>
<celldesigner:singleLine width="1.0"/>
<celldesigner:paint color="3fff0000" scheme="Color"/>
</celldesigner:briefView>
<celldesigner:info state="empty" angle="-1.5707963267948966"/>
</celldesigner:speciesAlias>
<celldesigner:speciesAlias id="sa448" species="s486">
<celldesigner:activity>inactive</celldesigner:activity>
<celldesigner:bounds x="759.375" y="739.6875" w="41.25" h="20.625"/>
<celldesigner:font size="12"/>
<celldesigner:view state="usual"/>
<celldesigner:usualView>
<celldesigner:innerPosition x="0.0" y="0.0"/>
<celldesigner:boxSize width="41.25" height="20.625"/>
<celldesigner:singleLine width="1.0"/>
<celldesigner:paint color="ffccff66" scheme="Color"/>
</celldesigner:usualView>
<celldesigner:briefView>
<celldesigner:innerPosition x="0.0" y="0.0"/>
<celldesigner:boxSize width="80.0" height="60.0"/>
<celldesigner:singleLine width="1.0"/>
<celldesigner:paint color="3fff0000" scheme="Color"/>
</celldesigner:briefView>
<celldesigner:info state="empty" angle="-1.5707963267948966"/>

</celldesigner:speciesAlias>

<celldesigner:speciesAlias id="sa451" species="s306">

<celldesigner:activity>inactive</celldesigner:activity>

<celldesigner:bounds x="765.5" y="621.5" w="49.0" h="17.0"/>

<celldesigner:font size="12"/>

<celldesigner:view state="usual"/>

<celldesigner:usualView>

<celldesigner:innerPosition x="0.0" y="0.0"/>

<celldesigner:boxSize width="49.0" height="17.0"/>

<celldesigner:singleLine width="1.0"/>

<celldesigner:paint color="ffccff66" scheme="Color"/>

</celldesigner:usualView>

<celldesigner:briefView>

<celldesigner:innerPosition x="0.0" y="0.0"/>

<celldesigner:boxSize width="80.0" height="60.0"/>

<celldesigner:singleLine width="1.0"/>

<celldesigner:paint color="3fff0000" scheme="Color"/>

</celldesigner:briefView>

<celldesigner:info state="empty" angle="-1.5707963267948966"/>

</celldesigner:speciesAlias>

<celldesigner:speciesAlias id="sa456" species="s68">

<celldesigner:activity>inactive</celldesigner:activity>

<celldesigner:bounds x="587.5" y="538.75" w="45.0" h="22.5"/>

<celldesigner:font size="12"/>

<celldesigner:view state="usual"/>

<celldesigner:usualView>

<celldesigner:innerPosition x="0.0" y="0.0"/>

<celldesigner:boxSize width="45.0" height="22.5"/>

<celldesigner:singleLine width="1.0"/>

<celldesigner:paint color="ffccff66" scheme="Color"/>

</celldesigner:usualView>

<celldesigner:briefView>

<celldesigner:innerPosition x="0.0" y="0.0"/>

<celldesigner:boxSize width="80.0" height="60.0"/>

<celldesigner:singleLine width="1.0"/>

<celldesigner:paint color="3fff0000" scheme="Color"/>

</celldesigner:briefView>

<celldesigner:info state="empty" angle="-1.5707963267948966"/>

</celldesigner:speciesAlias>

<celldesigner:speciesAlias id="sa458" species="s70">

<celldesigner:activity>inactive</celldesigner:activity>

<celldesigner:bounds x="677.5" y="618.75" w="45.0" h="22.5"/>

<celldesigner:font size="12"/>

<celldesigner:view state="usual"/>

<celldesigner:usualView>

<celldesigner:innerPosition x="0.0" y="0.0"/>

<celldesigner:boxSize width="45.0" height="22.5"/>

<celldesigner:singleLine width="1.0"/>

<celldesigner:paint color="ffccff66" scheme="Color"/>

</celldesigner:usualView>

<celldesigner:briefView>

<celldesigner:innerPosition x="0.0" y="0.0"/>

<celldesigner:boxSize width="80.0" height="60.0"/>

<celldesigner:singleLine width="1.0"/>

<celldesigner:paint color="3fff0000" scheme="Color"/>

</celldesigner:briefView>

<celldesigner:info state="empty" angle="-1.5707963267948966"/>

</celldesigner:speciesAlias>

<celldesigner:speciesAlias id="sa459" species="s69">

<celldesigner:activity>inactive</celldesigner:activity>

<celldesigner:bounds x="587.5" y="638.75" w="45.0" h="22.5"/>

<celldesigner:font size="12"/>

<celldesigner:view state="usual"/>

<celldesigner:usualView>

<celldesigner:innerPosition x="0.0" y="0.0"/>

<celldesigner:boxSize width="45.0" height="22.5"/>

<celldesigner:singleLine width="1.0"/>

<celldesigner:paint color="ffccff66" scheme="Color"/>

</celldesigner:usualView>

<celldesigner:briefView>

<celldesigner:innerPosition x="0.0" y="0.0"/>

<celldesigner:boxSize width="80.0" height="60.0"/>

<celldesigner:singleLine width="1.0"/>

<celldesigner:paint color="3fff0000" scheme="Color"/>

</celldesigner:briefView>

<celldesigner:info state="empty" angle="-1.5707963267948966"/>

</celldesigner:speciesAlias>

<celldesigner:speciesAlias id="sa461" species="s71">

<celldesigner:activity>inactive</celldesigner:activity>

<celldesigner:bounds x="578.75" y="709.375" w="62.5" h="21.25"/>

<celldesigner:font size="12"/>

<celldesigner:view state="usual"/>

<celldesigner:usualView>

<celldesigner:innerPosition x="0.0" y="0.0"/>

<celldesigner:boxSize width="62.5" height="21.25"/>

<celldesigner:singleLine width="1.0"/>

<celldesigner:paint color="ffccff66" scheme="Color"/>

</celldesigner:usualView>

<celldesigner:briefView>

<celldesigner:innerPosition x="0.0" y="0.0"/>

<celldesigner:boxSize width="80.0" height="60.0"/>

<celldesigner:singleLine width="1.0"/>

<celldesigner:paint color="3fff0000" scheme="Color"/>

</celldesigner:briefView>

<celldesigner:info state="empty" angle="-1.5707963267948966"/>

</celldesigner:speciesAlias>

<celldesigner:speciesAlias id="sa466" species="s72">

<celldesigner:activity>inactive</celldesigner:activity>

<celldesigner:bounds x="587.5" y="778.75" w="45.0" h="22.5"/>

<celldesigner:font size="12"/>

<celldesigner:view state="usual"/>

<celldesigner:usualView>

<celldesigner:innerPosition x="0.0" y="0.0"/>

<celldesigner:boxSize width="45.0" height="22.5"/>

<celldesigner:singleLine width="1.0"/>

<celldesigner:paint color="ffccff66" scheme="Color"/>

</celldesigner:usualView>

<celldesigner:briefView>

<celldesigner:innerPosition x="0.0" y="0.0"/>
<celldesigner:boxSize width="80.0" height="60.0"/>
<celldesigner:singleLine width="1.0"/>
<celldesigner:paint color="3fff0000" scheme="Color"/>
</celldesigner:briefView>
<celldesigner:info state="empty" angle="-1.5707963267948966"/>
</celldesigner:speciesAlias>
<celldesigner:speciesAlias id="sa467" species="s487">
<celldesigner:activity>inactive</celldesigner:activity>
<celldesigner:bounds x="420.0" y="539.375" w="100.0" h="21.25"/>
<celldesigner:font size="12"/>
<celldesigner:view state="usual"/>
<celldesigner:usualView>
<celldesigner:innerPosition x="0.0" y="0.0"/>
<celldesigner:boxSize width="100.0" height="21.25"/>
<celldesigner:singleLine width="1.0"/>
<celldesigner:paint color="ffccff66" scheme="Color"/>
</celldesigner:usualView>
<celldesigner:briefView>
<celldesigner:innerPosition x="0.0" y="0.0"/>
<celldesigner:boxSize width="80.0" height="60.0"/>
<celldesigner:singleLine width="1.0"/>
<celldesigner:paint color="3fff0000" scheme="Color"/>
</celldesigner:briefView>
<celldesigner:info state="empty" angle="-1.5707963267948966"/>
</celldesigner:speciesAlias>
<celldesigner:speciesAlias id="sa468" species="s488">

<celldesigner:activity>inactive</celldesigner:activity>

<celldesigner:bounds x="377.5" y="508.75" w="45.0" h="22.5"/>

<celldesigner:font size="12"/>

<celldesigner:view state="usual"/>

<celldesigner:usualView>

<celldesigner:innerPosition x="0.0" y="0.0"/>

<celldesigner:boxSize width="45.0" height="22.5"/>

<celldesigner:singleLine width="1.0"/>

<celldesigner:paint color="ffccff66" scheme="Color"/>

</celldesigner:usualView>

<celldesigner:briefView>

<celldesigner:innerPosition x="0.0" y="0.0"/>

<celldesigner:boxSize width="80.0" height="60.0"/>

<celldesigner:singleLine width="1.0"/>

<celldesigner:paint color="3fff0000" scheme="Color"/>

</celldesigner:briefView>

<celldesigner:info state="empty" angle="-1.5707963267948966"/>

</celldesigner:speciesAlias>

<celldesigner:speciesAlias id="sa470" species="s1091">

<celldesigner:activity>inactive</celldesigner:activity>

<celldesigner:bounds x="1295.0" y="61.0" w="70.0" h="18.0"/>

<celldesigner:font size="12"/>

<celldesigner:view state="usual"/>

<celldesigner:usualView>

<celldesigner:innerPosition x="0.0" y="0.0"/>

<celldesigner:boxSize width="70.0" height="18.0"/>

<celldesigner:singleLine width="1.0"/>

<celldesigner:paint color="ffccff66" scheme="Color"/>

</celldesigner:usualView>

<celldesigner:briefView>

<celldesigner:innerPosition x="0.0" y="0.0"/>

<celldesigner:boxSize width="80.0" height="60.0"/>

<celldesigner:singleLine width="1.0"/>

<celldesigner:paint color="3fff0000" scheme="Color"/>

</celldesigner:briefView>

<celldesigner:info state="empty" angle="-1.5707963267948966"/>

</celldesigner:speciesAlias>

<celldesigner:speciesAlias id="sa471" species="s436">

<celldesigner:activity>inactive</celldesigner:activity>

<celldesigner:bounds x="1346.0" y="80.0" w="48.0" h="20.0"/>

<celldesigner:font size="12"/>

<celldesigner:view state="usual"/>

<celldesigner:usualView>

<celldesigner:innerPosition x="0.0" y="0.0"/>

<celldesigner:boxSize width="48.0" height="20.0"/>

<celldesigner:singleLine width="1.0"/>

<celldesigner:paint color="ff009999" scheme="Color"/>

</celldesigner:usualView>

<celldesigner:briefView>

<celldesigner:innerPosition x="0.0" y="0.0"/>

<celldesigner:boxSize width="80.0" height="60.0"/>

<celldesigner:singleLine width="1.0"/>

<celldesigner:paint color="3fff0000" scheme="Color"/>

</celldesigner:briefView>

<celldesigner:info state="empty" angle="-1.5707963267948966"/>

</celldesigner:speciesAlias>

<celldesigner:speciesAlias id="sa472" species="s437">

<celldesigner:activity>inactive</celldesigner:activity>

<celldesigner:bounds x="1298.5" y="121.0" w="63.0" h="18.0"/>

<celldesigner:font size="12"/>

<celldesigner:view state="usual"/>

<celldesigner:usualView>

<celldesigner:innerPosition x="0.0" y="0.0"/>

<celldesigner:boxSize width="63.0" height="18.0"/>

<celldesigner:singleLine width="1.0"/>

<celldesigner:paint color="ffccff66" scheme="Color"/>

</celldesigner:usualView>

<celldesigner:briefView>

<celldesigner:innerPosition x="0.0" y="0.0"/>

<celldesigner:boxSize width="80.0" height="60.0"/>

<celldesigner:singleLine width="1.0"/>

<celldesigner:paint color="3fff0000" scheme="Color"/>

</celldesigner:briefView>

<celldesigner:info state="empty" angle="-1.5707963267948966"/>

</celldesigner:speciesAlias>

<celldesigner:speciesAlias id="sa473" species="s713">

<celldesigner:activity>inactive</celldesigner:activity>

<celldesigner:bounds x="1300.5" y="170.5" w="59.0" h="19.0"/>

<celldesigner:font size="12"/>

<celldesigner:view state="usual"/>

<celldesigner:usualView>

<celldesigner:innerPosition x="0.0" y="0.0"/>

<celldesigner:boxSize width="59.0" height="19.0"/>

<celldesigner:singleLine width="1.0"/>

<celldesigner:paint color="ff009999" scheme="Color"/>

</celldesigner:usualView>

<celldesigner:briefView>

<celldesigner:innerPosition x="0.0" y="0.0"/>

<celldesigner:boxSize width="80.0" height="60.0"/>

<celldesigner:singleLine width="1.0"/>

<celldesigner:paint color="3fff0000" scheme="Color"/>

</celldesigner:briefView>

<celldesigner:info state="empty" angle="-1.5707963267948966"/>

</celldesigner:speciesAlias>

<celldesigner:speciesAlias id="sa474" species="s714">

<celldesigner:activity>inactive</celldesigner:activity>

<celldesigner:bounds x="1302.0" y="230.5" w="56.0" h="19.0"/>

<celldesigner:font size="12"/>

<celldesigner:view state="usual"/>

<celldesigner:usualView>

<celldesigner:innerPosition x="0.0" y="0.0"/>

<celldesigner:boxSize width="56.0" height="19.0"/>

<celldesigner:singleLine width="1.0"/>

<celldesigner:paint color="ffccff66" scheme="Color"/>

</celldesigner:usualView>

<celldesigner:briefView>

<celldesigner:innerPosition x="0.0" y="0.0"/>

<celldesigner:boxSize width="80.0" height="60.0"/>

<celldesigner:singleLine width="1.0"/>

<celldesigner:paint color="3fff0000" scheme="Color"/>

</celldesigner:briefView>

<celldesigner:info state="empty" angle="-1.5707963267948966"/>

</celldesigner:speciesAlias>

<celldesigner:speciesAlias id="sa475" species="s717">

<celldesigner:activity>inactive</celldesigner:activity>

<celldesigner:bounds x="1295.0" y="291.0" w="70.0" h="18.0"/>

<celldesigner:font size="12"/>

<celldesigner:view state="usual"/>

<celldesigner:usualView>

<celldesigner:innerPosition x="0.0" y="0.0"/>

<celldesigner:boxSize width="70.0" height="18.0"/>

<celldesigner:singleLine width="1.0"/>

<celldesigner:paint color="ffccff66" scheme="Color"/>

</celldesigner:usualView>

<celldesigner:briefView>

<celldesigner:innerPosition x="0.0" y="0.0"/>

<celldesigner:boxSize width="80.0" height="60.0"/>

<celldesigner:singleLine width="1.0"/>

<celldesigner:paint color="3fff0000" scheme="Color"/>

</celldesigner:briefView>

<celldesigner:info state="empty" angle="-1.5707963267948966"/>

</celldesigner:speciesAlias>

<celldesigner:speciesAlias id="sa476" species="s438">

<celldesigner:activity>inactive</celldesigner:activity>

<celldesigner:bounds x="1295.0" y="361.0" w="70.0" h="18.0"/>

<celldesigner:font size="12"/>

<celldesigner:view state="usual"/>

<celldesigner:usualView>

<celldesigner:innerPosition x="0.0" y="0.0"/>

<celldesigner:boxSize width="70.0" height="18.0"/>

<celldesigner:singleLine width="1.0"/>

<celldesigner:paint color="ffccff66" scheme="Color"/>

</celldesigner:usualView>

<celldesigner:briefView>

<celldesigner:innerPosition x="0.0" y="0.0"/>

<celldesigner:boxSize width="80.0" height="60.0"/>

<celldesigner:singleLine width="1.0"/>

<celldesigner:paint color="3fff0000" scheme="Color"/>

</celldesigner:briefView>

<celldesigner:info state="empty" angle="-1.5707963267948966"/>

</celldesigner:speciesAlias>

<celldesigner:speciesAlias id="sa477" species="s1098">

<celldesigner:activity>inactive</celldesigner:activity>

<celldesigner:bounds x="1295.0" y="431.0" w="70.0" h="18.0"/>

<celldesigner:font size="12"/>

<celldesigner:view state="usual"/>

<celldesigner:usualView>

<celldesigner:innerPosition x="0.0" y="0.0"/>

<celldesigner:boxSize width="70.0" height="18.0"/>

<celldesigner:singleLine width="1.0"/>

<celldesigner:paint color="ffccff66" scheme="Color"/>

</celldesigner:usualView>

<celldesigner:briefView>

<celldesigner:innerPosition x="0.0" y="0.0"/>

<celldesigner:boxSize width="80.0" height="60.0"/>

<celldesigner:singleLine width="1.0"/>

<celldesigner:paint color="3fff0000" scheme="Color"/>

</celldesigner:briefView>

<celldesigner:info state="empty" angle="-1.5707963267948966"/>

</celldesigner:speciesAlias>

<celldesigner:speciesAlias id="sa478" species="s1097">

<celldesigner:activity>inactive</celldesigner:activity>

<celldesigner:bounds x="1296.9375" y="492.875" w="66.125" h="34.25"/>

<celldesigner:font size="12"/>

<celldesigner:view state="usual"/>

<celldesigner:usualView>

<celldesigner:innerPosition x="0.0" y="0.0"/>

<celldesigner:boxSize width="66.125" height="34.25"/>

<celldesigner:singleLine width="1.0"/>

<celldesigner:paint color="ffccff66" scheme="Color"/>

</celldesigner:usualView>

<celldesigner:briefView>

<celldesigner:innerPosition x="0.0" y="0.0"/>

<celldesigner:boxSize width="80.0" height="60.0"/>

<celldesigner:singleLine width="1.0"/>

<celldesigner:paint color="3fff0000" scheme="Color"/>

</celldesigner:briefView>

<celldesigner:info state="empty" angle="-1.5707963267948966"/>

</celldesigner:speciesAlias>

<celldesigner:speciesAlias id="sa479" species="s1102">
<celldesigner:activity>inactive</celldesigner:activity>
<celldesigner:bounds x="1310.9375" y="566.125" w="44.25" h="22.0"/>
<celldesigner:font size="12"/>
<celldesigner:view state="usual"/>
<celldesigner:usualView>
<celldesigner:innerPosition x="0.0" y="0.0"/>
<celldesigner:boxSize width="44.25" height="22.0"/>
<celldesigner:singleLine width="1.0"/>
<celldesigner:paint color="ffccff66" scheme="Color"/>
</celldesigner:usualView>
<celldesigner:briefView>
<celldesigner:innerPosition x="0.0" y="0.0"/>
<celldesigner:boxSize width="80.0" height="60.0"/>
<celldesigner:singleLine width="1.0"/>
<celldesigner:paint color="3fff0000" scheme="Color"/>
</celldesigner:briefView>
<celldesigner:info state="empty" angle="-1.5707963267948966"/>
</celldesigner:speciesAlias>
<celldesigner:speciesAlias id="sa480" species="s439">
<celldesigner:activity>inactive</celldesigner:activity>
<celldesigner:bounds x="1295.0" y="621.0" w="70.0" h="18.0"/>
<celldesigner:font size="12"/>
<celldesigner:view state="usual"/>
<celldesigner:usualView>
<celldesigner:innerPosition x="0.0" y="0.0"/>
<celldesigner:boxSize width="70.0" height="18.0"/>

<celldesigner:singleLine width="1.0"/>

<celldesigner:paint color="ffccff66" scheme="Color"/>

</celldesigner:usualView>

<celldesigner:briefView>

<celldesigner:innerPosition x="0.0" y="0.0"/>

<celldesigner:boxSize width="80.0" height="60.0"/>

<celldesigner:singleLine width="1.0"/>

<celldesigner:paint color="3fff0000" scheme="Color"/>

</celldesigner:briefView>

<celldesigner:info state="empty" angle="-1.5707963267948966"/>

</celldesigner:speciesAlias>

<celldesigner:speciesAlias id="sa481" species="s1104">

<celldesigner:activity>inactive</celldesigner:activity>

<celldesigner:bounds x="1295.0" y="681.0" w="70.0" h="18.0"/>

<celldesigner:font size="12"/>

<celldesigner:view state="usual"/>

<celldesigner:usualView>

<celldesigner:innerPosition x="0.0" y="0.0"/>

<celldesigner:boxSize width="70.0" height="18.0"/>

<celldesigner:singleLine width="1.0"/>

<celldesigner:paint color="ffccff66" scheme="Color"/>

</celldesigner:usualView>

<celldesigner:briefView>

<celldesigner:innerPosition x="0.0" y="0.0"/>

<celldesigner:boxSize width="80.0" height="60.0"/>

<celldesigner:singleLine width="1.0"/>

<celldesigner:paint color="3fff0000" scheme="Color"/>

</celldesigner:briefView>

<celldesigner:info state="empty" angle="-1.5707963267948966"/>

</celldesigner:speciesAlias>

<celldesigner:speciesAlias id="sa482" species="s730">

<celldesigner:activity>inactive</celldesigner:activity>

<celldesigner:bounds x="1395.0" y="741.0" w="70.0" h="18.0"/>

<celldesigner:font size="12"/>

<celldesigner:view state="usual"/>

<celldesigner:usualView>

<celldesigner:innerPosition x="0.0" y="0.0"/>

<celldesigner:boxSize width="70.0" height="18.0"/>

<celldesigner:singleLine width="1.0"/>

<celldesigner:paint color="ffccff66" scheme="Color"/>

</celldesigner:usualView>

<celldesigner:briefView>

<celldesigner:innerPosition x="0.0" y="0.0"/>

<celldesigner:boxSize width="80.0" height="60.0"/>

<celldesigner:singleLine width="1.0"/>

<celldesigner:paint color="3fff0000" scheme="Color"/>

</celldesigner:briefView>

<celldesigner:info state="empty" angle="-1.5707963267948966"/>

</celldesigner:speciesAlias>

<celldesigner:speciesAlias id="sa483" species="s1103">

<celldesigner:activity>inactive</celldesigner:activity>

<celldesigner:bounds x="1295.0" y="741.0" w="70.0" h="18.0"/>

<celldesigner:font size="12"/>

<celldesigner:view state="usual"/>

<celldesigner:usualView>
<celldesigner:innerPosition x="0.0" y="0.0"/>
<celldesigner:boxSize width="70.0" height="18.0"/>
<celldesigner:singleLine width="1.0"/>
<celldesigner:paint color="ffccff66" scheme="Color"/>
</celldesigner:usualView>
<celldesigner:briefView>
<celldesigner:innerPosition x="0.0" y="0.0"/>
<celldesigner:boxSize width="80.0" height="60.0"/>
<celldesigner:singleLine width="1.0"/>
<celldesigner:paint color="3fff0000" scheme="Color"/>
</celldesigner:briefView>
<celldesigner:info state="empty" angle="-1.5707963267948966"/>
</celldesigner:speciesAlias>
<celldesigner:speciesAlias id="sa484" species="s1073">
<celldesigner:activity>inactive</celldesigner:activity>
<celldesigner:bounds x="1395.0" y="801.0" w="70.0" h="18.0"/>
<celldesigner:font size="12"/>
<celldesigner:view state="usual"/>
<celldesigner:usualView>
<celldesigner:innerPosition x="0.0" y="0.0"/>
<celldesigner:boxSize width="70.0" height="18.0"/>
<celldesigner:singleLine width="1.0"/>
<celldesigner:paint color="ffccff66" scheme="Color"/>
</celldesigner:usualView>
<celldesigner:briefView>
<celldesigner:innerPosition x="0.0" y="0.0"/>

<celldesigner:boxSize width="80.0" height="60.0"/>

<celldesigner:singleLine width="1.0"/>

<celldesigner:paint color="3fff0000" scheme="Color"/>

</celldesigner:briefView>

<celldesigner:info state="empty" angle="-1.5707963267948966"/>

</celldesigner:speciesAlias>

<celldesigner:speciesAlias id="sa485" species="s96">

<celldesigner:activity>inactive</celldesigner:activity>

<celldesigner:bounds x="1295.0" y="801.0" w="70.0" h="18.0"/>

<celldesigner:font size="12"/>

<celldesigner:view state="usual"/>

<celldesigner:usualView>

<celldesigner:innerPosition x="0.0" y="0.0"/>

<celldesigner:boxSize width="70.0" height="18.0"/>

<celldesigner:singleLine width="1.0"/>

<celldesigner:paint color="ffccff66" scheme="Color"/>

</celldesigner:usualView>

<celldesigner:briefView>

<celldesigner:innerPosition x="0.0" y="0.0"/>

<celldesigner:boxSize width="80.0" height="60.0"/>

<celldesigner:singleLine width="1.0"/>

<celldesigner:paint color="3fff0000" scheme="Color"/>

</celldesigner:briefView>

<celldesigner:info state="empty" angle="-1.5707963267948966"/>

</celldesigner:speciesAlias>

<celldesigner:speciesAlias id="sa486" species="s732">

<celldesigner:activity>inactive</celldesigner:activity>

<celldesigner:bounds x="1295.0" y="991.0" w="70.0" h="18.0"/>
<celldesigner:font size="12"/>
<celldesigner:view state="usual"/>
<celldesigner:usualView>
<celldesigner:innerPosition x="0.0" y="0.0"/>
<celldesigner:boxSize width="70.0" height="18.0"/>
<celldesigner:singleLine width="1.0"/>
<celldesigner:paint color="ffccff66" scheme="Color"/>
</celldesigner:usualView>
<celldesigner:briefView>
<celldesigner:innerPosition x="0.0" y="0.0"/>
<celldesigner:boxSize width="80.0" height="60.0"/>
<celldesigner:singleLine width="1.0"/>
<celldesigner:paint color="3fff0000" scheme="Color"/>
</celldesigner:briefView>
<celldesigner:info state="empty" angle="-1.5707963267948966"/>
</celldesigner:speciesAlias>
<celldesigner:speciesAlias id="sa487" species="s733">
<celldesigner:activity>inactive</celldesigner:activity>
<celldesigner:bounds x="1295.0" y="1051.0" w="70.0" h="18.0"/>
<celldesigner:font size="12"/>
<celldesigner:view state="usual"/>
<celldesigner:usualView>
<celldesigner:innerPosition x="0.0" y="0.0"/>
<celldesigner:boxSize width="70.0" height="18.0"/>
<celldesigner:singleLine width="1.0"/>
<celldesigner:paint color="ffccff66" scheme="Color"/>

</celldesigner:usualView>

<celldesigner:briefView>

<celldesigner:innerPosition x="0.0" y="0.0"/>

<celldesigner:boxSize width="80.0" height="60.0"/>

<celldesigner:singleLine width="1.0"/>

<celldesigner:paint color="3fff0000" scheme="Color"/>

</celldesigner:briefView>

<celldesigner:info state="empty" angle="-1.5707963267948966"/>

</celldesigner:speciesAlias>

<celldesigner:speciesAlias id="sa488" species="s734">

<celldesigner:activity>inactive</celldesigner:activity>

<celldesigner:bounds x="1395.0" y="871.0" w="70.0" h="18.0"/>

<celldesigner:font size="12"/>

<celldesigner:view state="usual"/>

<celldesigner:usualView>

<celldesigner:innerPosition x="0.0" y="0.0"/>

<celldesigner:boxSize width="70.0" height="18.0"/>

<celldesigner:singleLine width="1.0"/>

<celldesigner:paint color="ffccff66" scheme="Color"/>

</celldesigner:usualView>

<celldesigner:briefView>

<celldesigner:innerPosition x="0.0" y="0.0"/>

<celldesigner:boxSize width="80.0" height="60.0"/>

<celldesigner:singleLine width="1.0"/>

<celldesigner:paint color="3fff0000" scheme="Color"/>

</celldesigner:briefView>

<celldesigner:info state="empty" angle="-1.5707963267948966"/>

</celldesigner:speciesAlias>

<celldesigner:speciesAlias id="sa489" species="s736">

<celldesigner:activity>inactive</celldesigner:activity>

<celldesigner:bounds x="1387.5" y="931.0" w="85.0" h="18.0"/>

<celldesigner:font size="12"/>

<celldesigner:view state="usual"/>

<celldesigner:usualView>

<celldesigner:innerPosition x="0.0" y="0.0"/>

<celldesigner:boxSize width="85.0" height="18.0"/>

<celldesigner:singleLine width="1.0"/>

<celldesigner:paint color="ffccff66" scheme="Color"/>

</celldesigner:usualView>

<celldesigner:briefView>

<celldesigner:innerPosition x="0.0" y="0.0"/>

<celldesigner:boxSize width="80.0" height="60.0"/>

<celldesigner:singleLine width="1.0"/>

<celldesigner:paint color="3fff0000" scheme="Color"/>

</celldesigner:briefView>

<celldesigner:info state="empty" angle="-1.5707963267948966"/>

</celldesigner:speciesAlias>

<celldesigner:speciesAlias id="sa490" species="s737">

<celldesigner:activity>inactive</celldesigner:activity>

<celldesigner:bounds x="1295.0" y="921.0" w="70.0" h="18.0"/>

<celldesigner:font size="12"/>

<celldesigner:view state="usual"/>

<celldesigner:usualView>

<celldesigner:innerPosition x="0.0" y="0.0"/>

<celldesigner:boxSize width="70.0" height="18.0"/>

<celldesigner:singleLine width="1.0"/>

<celldesigner:paint color="ffccff66" scheme="Color"/>

</celldesigner:usualView>

<celldesigner:briefView>

<celldesigner:innerPosition x="0.0" y="0.0"/>

<celldesigner:boxSize width="80.0" height="60.0"/>

<celldesigner:singleLine width="1.0"/>

<celldesigner:paint color="3fff0000" scheme="Color"/>

</celldesigner:briefView>

<celldesigner:info state="empty" angle="-1.5707963267948966"/>

</celldesigner:speciesAlias>

<celldesigner:speciesAlias id="sa491" species="s97">

<celldesigner:activity>inactive</celldesigner:activity>

<celldesigner:bounds x="1295.0" y="861.0" w="70.0" h="18.0"/>

<celldesigner:font size="12"/>

<celldesigner:view state="usual"/>

<celldesigner:usualView>

<celldesigner:innerPosition x="0.0" y="0.0"/>

<celldesigner:boxSize width="70.0" height="18.0"/>

<celldesigner:singleLine width="1.0"/>

<celldesigner:paint color="ffccff66" scheme="Color"/>

</celldesigner:usualView>

<celldesigner:briefView>

<celldesigner:innerPosition x="0.0" y="0.0"/>

<celldesigner:boxSize width="80.0" height="60.0"/>

<celldesigner:singleLine width="1.0"/>

<celldesigner:paint color="3fff0000" scheme="Color"/>
</celldesigner:briefView>
<celldesigner:info state="empty" angle="-1.5707963267948966"/>
</celldesigner:speciesAlias>
<celldesigner:speciesAlias id="sa492" species="s440">
<celldesigner:activity>inactive</celldesigner:activity>
<celldesigner:bounds x="331.125" y="907.2792653580709" w="26.0" h="16.0"/>
<celldesigner:font size="12"/>
<celldesigner:view state="usual"/>
<celldesigner:usualView>
<celldesigner:innerPosition x="0.0" y="0.0"/>
<celldesigner:boxSize width="26.0" height="16.0"/>
<celldesigner:singleLine width="1.0"/>
<celldesigner:paint color="ff00cccc" scheme="Color"/>
</celldesigner:usualView>
<celldesigner:briefView>
<celldesigner:innerPosition x="0.0" y="0.0"/>
<celldesigner:boxSize width="80.0" height="60.0"/>
<celldesigner:singleLine width="1.0"/>
<celldesigner:paint color="3fff0000" scheme="Color"/>
</celldesigner:briefView>
<celldesigner:info state="empty" angle="-1.5707963267948966"/>
</celldesigner:speciesAlias>
<celldesigner:speciesAlias id="sa493" species="s1027">
<celldesigner:activity>inactive</celldesigner:activity>
<celldesigner:bounds x="550.625" y="956.2792653580709" w="27.0" h="18.0"/>
<celldesigner:font size="12"/>

<celldesigner:view state="usual"/>

<celldesigner:usualView>

<celldesigner:innerPosition x="0.0" y="0.0"/>

<celldesigner:boxSize width="27.0" height="18.0"/>

<celldesigner:singleLine width="1.0"/>

<celldesigner:paint color="ffffff00" scheme="Color"/>

</celldesigner:usualView>

<celldesigner:briefView>

<celldesigner:innerPosition x="0.0" y="0.0"/>

<celldesigner:boxSize width="80.0" height="60.0"/>

<celldesigner:singleLine width="1.0"/>

<celldesigner:paint color="3fff0000" scheme="Color"/>

</celldesigner:briefView>

<celldesigner:info state="empty" angle="-1.5707963267948966"/>

</celldesigner:speciesAlias>

<celldesigner:speciesAlias id="sa494" species="s1000">

<celldesigner:activity>inactive</celldesigner:activity>

<celldesigner:bounds x="476.5" y="981.0" w="27.0" h="18.0"/>

<celldesigner:font size="12"/>

<celldesigner:view state="usual"/>

<celldesigner:usualView>

<celldesigner:innerPosition x="0.0" y="0.0"/>

<celldesigner:boxSize width="27.0" height="18.0"/>

<celldesigner:singleLine width="1.0"/>

<celldesigner:paint color="ffffff00" scheme="Color"/>

</celldesigner:usualView>

<celldesigner:briefView>

<celldesigner:innerPosition x="0.0" y="0.0"/>

<celldesigner:boxSize width="80.0" height="60.0"/>

<celldesigner:singleLine width="1.0"/>

<celldesigner:paint color="3fff0000" scheme="Color"/>

</celldesigner:briefView>

<celldesigner:info state="empty" angle="-1.5707963267948966"/>

</celldesigner:speciesAlias>

<celldesigner:speciesAlias id="sa495" species="s1092">

<celldesigner:activity>inactive</celldesigner:activity>

<celldesigner:bounds x="330.625" y="976.7792653580709" w="27.0" h="17.0"/>

<celldesigner:font size="12"/>

<celldesigner:view state="usual"/>

<celldesigner:usualView>

<celldesigner:innerPosition x="0.0" y="0.0"/>

<celldesigner:boxSize width="27.0" height="17.0"/>

<celldesigner:singleLine width="1.0"/>

<celldesigner:paint color="ff00cccc" scheme="Color"/>

</celldesigner:usualView>

<celldesigner:briefView>

<celldesigner:innerPosition x="0.0" y="0.0"/>

<celldesigner:boxSize width="80.0" height="60.0"/>

<celldesigner:singleLine width="1.0"/>

<celldesigner:paint color="3fff0000" scheme="Color"/>

</celldesigner:briefView>

<celldesigner:info state="empty" angle="-1.5707963267948966"/>

</celldesigner:speciesAlias>

<celldesigner:speciesAlias id="sa496" species="s1089">

<celldesigner:activity>inactive</celldesigner:activity>

<celldesigner:bounds x="631.0" y="905.0" w="27.0" h="18.0"/>

<celldesigner:font size="12"/>

<celldesigner:view state="usual"/>

<celldesigner:usualView>

<celldesigner:innerPosition x="0.0" y="0.0"/>

<celldesigner:boxSize width="27.0" height="18.0"/>

<celldesigner:singleLine width="1.0"/>

<celldesigner:paint color="ffccff66" scheme="Color"/>

</celldesigner:usualView>

<celldesigner:briefView>

<celldesigner:innerPosition x="0.0" y="0.0"/>

<celldesigner:boxSize width="80.0" height="60.0"/>

<celldesigner:singleLine width="1.0"/>

<celldesigner:paint color="3fff0000" scheme="Color"/>

</celldesigner:briefView>

<celldesigner:info state="empty" angle="-1.5707963267948966"/>

</celldesigner:speciesAlias>

<celldesigner:speciesAlias id="sa497" species="s1090">

<celldesigner:activity>inactive</celldesigner:activity>

<celldesigner:bounds x="628.0" y="989.0" w="31.0" h="17.0"/>

<celldesigner:font size="12"/>

<celldesigner:view state="usual"/>

<celldesigner:usualView>

<celldesigner:innerPosition x="0.0" y="0.0"/>

<celldesigner:boxSize width="31.0" height="17.0"/>

<celldesigner:singleLine width="1.0"/>

<celldesigner:paint color="ffccff66" scheme="Color"/>

</celldesigner:usualView>

<celldesigner:briefView>

<celldesigner:innerPosition x="0.0" y="0.0"/>

<celldesigner:boxSize width="80.0" height="60.0"/>

<celldesigner:singleLine width="1.0"/>

<celldesigner:paint color="3fff0000" scheme="Color"/>

</celldesigner:briefView>

<celldesigner:info state="empty" angle="-1.5707963267948966"/>

</celldesigner:speciesAlias>

<celldesigner:speciesAlias id="sa498" species="s1038">

<celldesigner:activity>inactive</celldesigner:activity>

<celldesigner:bounds x="235.125" y="1026.279265358071" w="38.0" h="18.0"/>

<celldesigner:font size="12"/>

<celldesigner:view state="usual"/>

<celldesigner:usualView>

<celldesigner:innerPosition x="0.0" y="0.0"/>

<celldesigner:boxSize width="38.0" height="18.0"/>

<celldesigner:singleLine width="1.0"/>

<celldesigner:paint color="ffffff00" scheme="Color"/>

</celldesigner:usualView>

<celldesigner:briefView>

<celldesigner:innerPosition x="0.0" y="0.0"/>

<celldesigner:boxSize width="80.0" height="60.0"/>

<celldesigner:singleLine width="1.0"/>

<celldesigner:paint color="3fff0000" scheme="Color"/>

</celldesigner:briefView>

<celldesigner:info state="empty" angle="-1.5707963267948966"/>

</celldesigner:speciesAlias>

<celldesigner:speciesAlias id="sa499" species="s441">

<celldesigner:activity>inactive</celldesigner:activity>

<celldesigner:bounds x="330.625" y="1026.279265358071" w="27.0" h="18.0"/>

<celldesigner:font size="12"/>

<celldesigner:view state="usual"/>

<celldesigner:usualView>

<celldesigner:innerPosition x="0.0" y="0.0"/>

<celldesigner:boxSize width="27.0" height="18.0"/>

<celldesigner:singleLine width="1.0"/>

<celldesigner:paint color="ffccff66" scheme="Color"/>

</celldesigner:usualView>

<celldesigner:briefView>

<celldesigner:innerPosition x="0.0" y="0.0"/>

<celldesigner:boxSize width="80.0" height="60.0"/>

<celldesigner:singleLine width="1.0"/>

<celldesigner:paint color="3fff0000" scheme="Color"/>

</celldesigner:briefView>

<celldesigner:info state="empty" angle="-1.5707963267948966"/>

</celldesigner:speciesAlias>

<celldesigner:speciesAlias id="sa500" species="s443">

<celldesigner:activity>inactive</celldesigner:activity>

<celldesigner:bounds x="463.0" y="1101.0" w="54.0" h="18.0"/>

<celldesigner:font size="12"/>

<celldesigner:view state="usual"/>

<celldesigner:usualView>

<celldesigner:innerPosition x="0.0" y="0.0"/>

<celldesigner:boxSize width="54.0" height="18.0"/>

<celldesigner:singleLine width="1.0"/>

<celldesigner:paint color="ffccff66" scheme="Color"/>

</celldesigner:usualView>

<celldesigner:briefView>

<celldesigner:innerPosition x="0.0" y="0.0"/>

<celldesigner:boxSize width="80.0" height="60.0"/>

<celldesigner:singleLine width="1.0"/>

<celldesigner:paint color="3fff0000" scheme="Color"/>

</celldesigner:briefView>

<celldesigner:info state="empty" angle="-1.5707963267948966"/>

</celldesigner:speciesAlias>

<celldesigner:speciesAlias id="sa501" species="s1100">

<celldesigner:activity>inactive</celldesigner:activity>

<celldesigner:bounds x="478.5" y="1040.5" w="27.0" h="18.0"/>

<celldesigner:font size="12"/>

<celldesigner:view state="usual"/>

<celldesigner:usualView>

<celldesigner:innerPosition x="0.0" y="0.0"/>

<celldesigner:boxSize width="27.0" height="18.0"/>

<celldesigner:singleLine width="1.0"/>

<celldesigner:paint color="ffccff66" scheme="Color"/>

</celldesigner:usualView>

<celldesigner:briefView>

<celldesigner:innerPosition x="0.0" y="0.0"/>

<celldesigner:boxSize width="80.0" height="60.0"/>

<celldesigner:singleLine width="1.0"/>

<celldesigner:paint color="3fff0000" scheme="Color"/>

</celldesigner:briefView>

<celldesigner:info state="empty" angle="-1.5707963267948966"/>

</celldesigner:speciesAlias>

<celldesigner:speciesAlias id="sa502" species="s1096">

<celldesigner:activity>inactive</celldesigner:activity>

<celldesigner:bounds x="629.0" y="1054.0" w="27.0" h="18.0"/>

<celldesigner:font size="12"/>

<celldesigner:view state="usual"/>

<celldesigner:usualView>

<celldesigner:innerPosition x="0.0" y="0.0"/>

<celldesigner:boxSize width="27.0" height="18.0"/>

<celldesigner:singleLine width="1.0"/>

<celldesigner:paint color="ffccff66" scheme="Color"/>

</celldesigner:usualView>

<celldesigner:briefView>

<celldesigner:innerPosition x="0.0" y="0.0"/>

<celldesigner:boxSize width="80.0" height="60.0"/>

<celldesigner:singleLine width="1.0"/>

<celldesigner:paint color="3fff0000" scheme="Color"/>

</celldesigner:briefView>

<celldesigner:info state="empty" angle="-1.5707963267948966"/>

</celldesigner:speciesAlias>

<celldesigner:speciesAlias id="sa503" species="s1095">

<celldesigner:activity>inactive</celldesigner:activity>

<celldesigner:bounds x="548.5" y="1050.5" w="27.0" h="18.0"/>

<celldesigner:font size="12"/>

<celldesigner:view state="usual"/>

<celldesigner:usualView>

<celldesigner:innerPosition x="0.0" y="0.0"/>

<celldesigner:boxSize width="27.0" height="18.0"/>

<celldesigner:singleLine width="1.0"/>

<celldesigner:paint color="ffccff66" scheme="Color"/>

</celldesigner:usualView>

<celldesigner:briefView>

<celldesigner:innerPosition x="0.0" y="0.0"/>

<celldesigner:boxSize width="80.0" height="60.0"/>

<celldesigner:singleLine width="1.0"/>

<celldesigner:paint color="3fff0000" scheme="Color"/>

</celldesigner:briefView>

<celldesigner:info state="empty" angle="-1.5707963267948966"/>

</celldesigner:speciesAlias>

<celldesigner:speciesAlias id="sa504" species="s433">

<celldesigner:activity>inactive</celldesigner:activity>

<celldesigner:bounds x="393.0" y="1131.5" w="54.0" h="17.0"/>

<celldesigner:font size="12"/>

<celldesigner:view state="usual"/>

<celldesigner:usualView>

<celldesigner:innerPosition x="0.0" y="0.0"/>

<celldesigner:boxSize width="54.0" height="17.0"/>

<celldesigner:singleLine width="1.0"/>

<celldesigner:paint color="ffccff66" scheme="Color"/>

</celldesigner:usualView>

<celldesigner:briefView>

<celldesigner:innerPosition x="0.0" y="0.0"/>

<celldesigner:boxSize width="80.0" height="60.0"/>

<celldesigner:singleLine width="1.0"/>

<celldesigner:paint color="3fff0000" scheme="Color"/>

</celldesigner:briefView>

<celldesigner:info state="empty" angle="-1.5707963267948966"/>

</celldesigner:speciesAlias>

<celldesigner:speciesAlias id="sa505" species="s445">

<celldesigner:activity>inactive</celldesigner:activity>

<celldesigner:bounds x="537.125" y="1136.279265358071" w="54.0" h="18.0"/>

<celldesigner:font size="12"/>

<celldesigner:view state="usual"/>

<celldesigner:usualView>

<celldesigner:innerPosition x="0.0" y="0.0"/>

<celldesigner:boxSize width="54.0" height="18.0"/>

<celldesigner:singleLine width="1.0"/>

<celldesigner:paint color="ffccff66" scheme="Color"/>

</celldesigner:usualView>

<celldesigner:briefView>

<celldesigner:innerPosition x="0.0" y="0.0"/>

<celldesigner:boxSize width="80.0" height="60.0"/>

<celldesigner:singleLine width="1.0"/>

<celldesigner:paint color="3fff0000" scheme="Color"/>

</celldesigner:briefView>

<celldesigner:info state="empty" angle="-1.5707963267948966"/>

</celldesigner:speciesAlias>

<celldesigner:speciesAlias id="sa506" species="s1037">
<celldesigner:activity>inactive</celldesigner:activity>
<celldesigner:bounds x="623.0" y="1130.5" w="38.0" h="18.0"/>
<celldesigner:font size="12"/>
<celldesigner:view state="usual"/>
<celldesigner:usualView>
<celldesigner:innerPosition x="0.0" y="0.0"/>
<celldesigner:boxSize width="38.0" height="18.0"/>
<celldesigner:singleLine width="1.0"/>
<celldesigner:paint color="ffffff00" scheme="Color"/>
</celldesigner:usualView>
<celldesigner:briefView>
<celldesigner:innerPosition x="0.0" y="0.0"/>
<celldesigner:boxSize width="80.0" height="60.0"/>
<celldesigner:singleLine width="1.0"/>
<celldesigner:paint color="3fff0000" scheme="Color"/>
</celldesigner:briefView>
<celldesigner:info state="empty" angle="-1.5707963267948966"/>
</celldesigner:speciesAlias>
<celldesigner:speciesAlias id="sa508" species="s269">
<celldesigner:activity>inactive</celldesigner:activity>
<celldesigner:bounds x="282.0" y="1175.5" w="38.0" h="18.0"/>
<celldesigner:font size="12"/>
<celldesigner:view state="usual"/>
<celldesigner:usualView>
<celldesigner:innerPosition x="0.0" y="0.0"/>
<celldesigner:boxSize width="38.0" height="18.0"/>

<celldesigner:singleLine width="1.0"/>

<celldesigner:paint color="ffccff66" scheme="Color"/>

</celldesigner:usualView>

<celldesigner:briefView>

<celldesigner:innerPosition x="0.0" y="0.0"/>

<celldesigner:boxSize width="80.0" height="60.0"/>

<celldesigner:singleLine width="1.0"/>

<celldesigner:paint color="3fff0000" scheme="Color"/>

</celldesigner:briefView>

<celldesigner:info state="empty" angle="-1.5707963267948966"/>

</celldesigner:speciesAlias>

<celldesigner:speciesAlias id="sa509" species="s1004">

<celldesigner:activity>inactive</celldesigner:activity>

<celldesigner:bounds x="252.0" y="1197.5" w="37.0" h="16.0"/>

<celldesigner:font size="12"/>

<celldesigner:view state="usual"/>

<celldesigner:usualView>

<celldesigner:innerPosition x="0.0" y="0.0"/>

<celldesigner:boxSize width="37.0" height="16.0"/>

<celldesigner:singleLine width="1.0"/>

<celldesigner:paint color="ffffff00" scheme="Color"/>

</celldesigner:usualView>

<celldesigner:briefView>

<celldesigner:innerPosition x="0.0" y="0.0"/>

<celldesigner:boxSize width="80.0" height="60.0"/>

<celldesigner:singleLine width="1.0"/>

<celldesigner:paint color="3fff0000" scheme="Color"/>

</celldesigner:briefView>

<celldesigner:info state="empty" angle="-1.5707963267948966"/>

</celldesigner:speciesAlias>

<celldesigner:speciesAlias id="sa510" species="s278">

<celldesigner:activity>inactive</celldesigner:activity>

<celldesigner:bounds x="559.625" y="1193.2792653580707" w="37.0" h="18.0"/>

<celldesigner:font size="12"/>

<celldesigner:view state="usual"/>

<celldesigner:usualView>

<celldesigner:innerPosition x="0.0" y="0.0"/>

<celldesigner:boxSize width="37.0" height="18.0"/>

<celldesigner:singleLine width="1.0"/>

<celldesigner:paint color="ffccff66" scheme="Color"/>

</celldesigner:usualView>

<celldesigner:briefView>

<celldesigner:innerPosition x="0.0" y="0.0"/>

<celldesigner:boxSize width="80.0" height="60.0"/>

<celldesigner:singleLine width="1.0"/>

<celldesigner:paint color="3fff0000" scheme="Color"/>

</celldesigner:briefView>

<celldesigner:info state="empty" angle="-1.5707963267948966"/>

</celldesigner:speciesAlias>

<celldesigner:speciesAlias id="sa511" species="s1013">

<celldesigner:activity>inactive</celldesigner:activity>

<celldesigner:bounds x="452.625" y="1188.2792653580707" w="39.0" h="18.0"/>

<celldesigner:font size="12"/>

<celldesigner:view state="usual"/>

<celldesigner:usualView>
<celldesigner:innerPosition x="0.0" y="0.0"/>
<celldesigner:boxSize width="39.0" height="18.0"/>
<celldesigner:singleLine width="1.0"/>
<celldesigner:paint color="ffccff66" scheme="Color"/>
</celldesigner:usualView>
<celldesigner:briefView>
<celldesigner:innerPosition x="0.0" y="0.0"/>
<celldesigner:boxSize width="80.0" height="60.0"/>
<celldesigner:singleLine width="1.0"/>
<celldesigner:paint color="3fff0000" scheme="Color"/>
</celldesigner:briefView>
<celldesigner:info state="empty" angle="-1.5707963267948966"/>
</celldesigner:speciesAlias>
<celldesigner:speciesAlias id="sa512" species="s1108">
<celldesigner:activity>inactive</celldesigner:activity>
<celldesigner:bounds x="660.125" y="1202.2792653580707" w="37.0" h="18.0"/>
<celldesigner:font size="12"/>
<celldesigner:view state="usual"/>
<celldesigner:usualView>
<celldesigner:innerPosition x="0.0" y="0.0"/>
<celldesigner:boxSize width="37.0" height="18.0"/>
<celldesigner:singleLine width="1.0"/>
<celldesigner:paint color="ffccff66" scheme="Color"/>
</celldesigner:usualView>
<celldesigner:briefView>
<celldesigner:innerPosition x="0.0" y="0.0"/>

<celldesigner:boxSize width="80.0" height="60.0"/>

<celldesigner:singleLine width="1.0"/>

<celldesigner:paint color="3fff0000" scheme="Color"/>

</celldesigner:briefView>

<celldesigner:info state="empty" angle="-1.5707963267948966"/>

</celldesigner:speciesAlias>

<celldesigner:speciesAlias id="sa513" species="s270">

<celldesigner:activity>inactive</celldesigner:activity>

<celldesigner:bounds x="269.0" y="1251.5" w="62.0" h="17.0"/>

<celldesigner:font size="12"/>

<celldesigner:view state="usual"/>

<celldesigner:usualView>

<celldesigner:innerPosition x="0.0" y="0.0"/>

<celldesigner:boxSize width="62.0" height="17.0"/>

<celldesigner:singleLine width="1.0"/>

<celldesigner:paint color="ffccff66" scheme="Color"/>

</celldesigner:usualView>

<celldesigner:briefView>

<celldesigner:innerPosition x="0.0" y="0.0"/>

<celldesigner:boxSize width="80.0" height="60.0"/>

<celldesigner:singleLine width="1.0"/>

<celldesigner:paint color="3fff0000" scheme="Color"/>

</celldesigner:briefView>

<celldesigner:info state="empty" angle="-1.5707963267948966"/>

</celldesigner:speciesAlias>

<celldesigner:speciesAlias id="sa514" species="s1050">

<celldesigner:activity>inactive</celldesigner:activity>

<celldesigner:bounds x="659.125" y="1276.2792653580707" w="37.0" h="18.0"/>

<celldesigner:font size="12"/>

<celldesigner:view state="usual"/>

<celldesigner:usualView>

<celldesigner:innerPosition x="0.0" y="0.0"/>

<celldesigner:boxSize width="37.0" height="18.0"/>

<celldesigner:singleLine width="1.0"/>

<celldesigner:paint color="ffffff00" scheme="Color"/>

</celldesigner:usualView>

<celldesigner:briefView>

<celldesigner:innerPosition x="0.0" y="0.0"/>

<celldesigner:boxSize width="80.0" height="60.0"/>

<celldesigner:singleLine width="1.0"/>

<celldesigner:paint color="3fff0000" scheme="Color"/>

</celldesigner:briefView>

<celldesigner:info state="empty" angle="-1.5707963267948966"/>

</celldesigner:speciesAlias>

<celldesigner:speciesAlias id="sa515" species="s277">

<celldesigner:activity>inactive</celldesigner:activity>

<celldesigner:bounds x="418.625" y="1295.779265358071" w="49.0" h="19.0"/>

<celldesigner:font size="12"/>

<celldesigner:view state="usual"/>

<celldesigner:usualView>

<celldesigner:innerPosition x="0.0" y="0.0"/>

<celldesigner:boxSize width="49.0" height="19.0"/>

<celldesigner:singleLine width="1.0"/>

<celldesigner:paint color="ffccff66" scheme="Color"/>

</celldesigner:usualView>

<celldesigner:briefView>

<celldesigner:innerPosition x="0.0" y="0.0"/>

<celldesigner:boxSize width="80.0" height="60.0"/>

<celldesigner:singleLine width="1.0"/>

<celldesigner:paint color="3fff0000" scheme="Color"/>

</celldesigner:briefView>

<celldesigner:info state="empty" angle="-1.5707963267948966"/>

</celldesigner:speciesAlias>

<celldesigner:speciesAlias id="sa516" species="s1012">

<celldesigner:activity>inactive</celldesigner:activity>

<celldesigner:bounds x="563.0" y="1290.5" w="38.0" h="18.0"/>

<celldesigner:font size="12"/>

<celldesigner:view state="usual"/>

<celldesigner:usualView>

<celldesigner:innerPosition x="0.0" y="0.0"/>

<celldesigner:boxSize width="38.0" height="18.0"/>

<celldesigner:singleLine width="1.0"/>

<celldesigner:paint color="ffffff00" scheme="Color"/>

</celldesigner:usualView>

<celldesigner:briefView>

<celldesigner:innerPosition x="0.0" y="0.0"/>

<celldesigner:boxSize width="80.0" height="60.0"/>

<celldesigner:singleLine width="1.0"/>

<celldesigner:paint color="3fff0000" scheme="Color"/>

</celldesigner:briefView>

<celldesigner:info state="empty" angle="-1.5707963267948966"/>

</celldesigner:speciesAlias>

<celldesigner:speciesAlias id="sa517" species="s271">

<celldesigner:activity>inactive</celldesigner:activity>

<celldesigner:bounds x="280.0" y="1318.5" w="38.0" h="18.0"/>

<celldesigner:font size="12"/>

<celldesigner:view state="usual"/>

<celldesigner:usualView>

<celldesigner:innerPosition x="0.0" y="0.0"/>

<celldesigner:boxSize width="38.0" height="18.0"/>

<celldesigner:singleLine width="1.0"/>

<celldesigner:paint color="ffccff66" scheme="Color"/>

</celldesigner:usualView>

<celldesigner:briefView>

<celldesigner:innerPosition x="0.0" y="0.0"/>

<celldesigner:boxSize width="80.0" height="60.0"/>

<celldesigner:singleLine width="1.0"/>

<celldesigner:paint color="3fff0000" scheme="Color"/>

</celldesigner:briefView>

<celldesigner:info state="empty" angle="-1.5707963267948966"/>

</celldesigner:speciesAlias>

<celldesigner:speciesAlias id="sa519" species="s1051">

<celldesigner:activity>inactive</celldesigner:activity>

<celldesigner:bounds x="685.125" y="1376.279265358071" w="38.0" h="18.0"/>

<celldesigner:font size="12"/>

<celldesigner:view state="usual"/>

<celldesigner:usualView>

<celldesigner:innerPosition x="0.0" y="0.0"/>

<celldesigner:boxSize width="38.0" height="18.0"/>

<celldesigner:singleLine width="1.0"/>

<celldesigner:paint color="ffffff00" scheme="Color"/>

</celldesigner:usualView>

<celldesigner:briefView>

<celldesigner:innerPosition x="0.0" y="0.0"/>

<celldesigner:boxSize width="80.0" height="60.0"/>

<celldesigner:singleLine width="1.0"/>

<celldesigner:paint color="3fff0000" scheme="Color"/>

</celldesigner:briefView>

<celldesigner:info state="empty" angle="-1.5707963267948966"/>

</celldesigner:speciesAlias>

<celldesigner:speciesAlias id="sa520" species="s276">

<celldesigner:activity>inactive</celldesigner:activity>

<celldesigner:bounds x="419.125" y="1378.2792653580707" w="46.0" h="18.0"/>

<celldesigner:font size="12"/>

<celldesigner:view state="usual"/>

<celldesigner:usualView>

<celldesigner:innerPosition x="0.0" y="0.0"/>

<celldesigner:boxSize width="46.0" height="18.0"/>

<celldesigner:singleLine width="1.0"/>

<celldesigner:paint color="ffccff66" scheme="Color"/>

</celldesigner:usualView>

<celldesigner:briefView>

<celldesigner:innerPosition x="0.0" y="0.0"/>

<celldesigner:boxSize width="80.0" height="60.0"/>

<celldesigner:singleLine width="1.0"/>

<celldesigner:paint color="3fff0000" scheme="Color"/>
</celldesigner:briefView>
<celldesigner:info state="empty" angle="-1.5707963267948966"/>
</celldesigner:speciesAlias>
<celldesigner:speciesAlias id="sa521" species="s272">
<celldesigner:activity>inactive</celldesigner:activity>
<celldesigner:bounds x="277.0" y="1387.5" w="38.0" h="18.0"/>
<celldesigner:font size="12"/>
<celldesigner:view state="usual"/>
<celldesigner:usualView>
<celldesigner:innerPosition x="0.0" y="0.0"/>
<celldesigner:boxSize width="38.0" height="18.0"/>
<celldesigner:singleLine width="1.0"/>
<celldesigner:paint color="ffccff66" scheme="Color"/>
</celldesigner:usualView>
<celldesigner:briefView>
<celldesigner:innerPosition x="0.0" y="0.0"/>
<celldesigner:boxSize width="80.0" height="60.0"/>
<celldesigner:singleLine width="1.0"/>
<celldesigner:paint color="3fff0000" scheme="Color"/>
</celldesigner:briefView>
<celldesigner:info state="empty" angle="-1.5707963267948966"/>
</celldesigner:speciesAlias>
<celldesigner:speciesAlias id="sa522" species="s28">
<celldesigner:activity>inactive</celldesigner:activity>
<celldesigner:bounds x="230.25" y="1376.0" w="51.0" h="18.0"/>
<celldesigner:font size="12"/>

<celldesigner:view state="usual"/>

<celldesigner:usualView>

<celldesigner:innerPosition x="0.0" y="0.0"/>

<celldesigner:boxSize width="51.0" height="18.0"/>

<celldesigner:singleLine width="1.0"/>

<celldesigner:paint color="ffccff66" scheme="Color"/>

</celldesigner:usualView>

<celldesigner:briefView>

<celldesigner:innerPosition x="0.0" y="0.0"/>

<celldesigner:boxSize width="80.0" height="60.0"/>

<celldesigner:singleLine width="1.0"/>

<celldesigner:paint color="3fff0000" scheme="Color"/>

</celldesigner:briefView>

<celldesigner:info state="empty" angle="-1.5707963267948966"/>

</celldesigner:speciesAlias>

<celldesigner:speciesAlias id="sa523" species="s273">

<celldesigner:activity>inactive</celldesigner:activity>

<celldesigner:bounds x="274.0" y="1460.5" w="53.0" h="17.0"/>

<celldesigner:font size="12"/>

<celldesigner:view state="usual"/>

<celldesigner:usualView>

<celldesigner:innerPosition x="0.0" y="0.0"/>

<celldesigner:boxSize width="53.0" height="17.0"/>

<celldesigner:singleLine width="1.0"/>

<celldesigner:paint color="ffccff66" scheme="Color"/>

</celldesigner:usualView>

<celldesigner:briefView>

<celldesigner:innerPosition x="0.0" y="0.0"/>

<celldesigner:boxSize width="80.0" height="60.0"/>

<celldesigner:singleLine width="1.0"/>

<celldesigner:paint color="3fff0000" scheme="Color"/>

</celldesigner:briefView>

<celldesigner:info state="empty" angle="-1.5707963267948966"/>

</celldesigner:speciesAlias>

<celldesigner:speciesAlias id="sa524" species="s1111">

<celldesigner:activity>inactive</celldesigner:activity>

<celldesigner:bounds x="637.125" y="1534.2792653580711" w="38.0" h="18.0"/>

<celldesigner:font size="12"/>

<celldesigner:view state="usual"/>

<celldesigner:usualView>

<celldesigner:innerPosition x="0.0" y="0.0"/>

<celldesigner:boxSize width="38.0" height="18.0"/>

<celldesigner:singleLine width="1.0"/>

<celldesigner:paint color="ffccff66" scheme="Color"/>

</celldesigner:usualView>

<celldesigner:briefView>

<celldesigner:innerPosition x="0.0" y="0.0"/>

<celldesigner:boxSize width="80.0" height="60.0"/>

<celldesigner:singleLine width="1.0"/>

<celldesigner:paint color="3fff0000" scheme="Color"/>

</celldesigner:briefView>

<celldesigner:info state="empty" angle="-1.5707963267948966"/>

</celldesigner:speciesAlias>

<celldesigner:speciesAlias id="sa525" species="s1114">

<celldesigner:activity>inactive</celldesigner:activity>

<celldesigner:bounds x="277.0" y="1527.5" w="38.0" h="18.0"/>

<celldesigner:font size="12"/>

<celldesigner:view state="usual"/>

<celldesigner:usualView>

<celldesigner:innerPosition x="0.0" y="0.0"/>

<celldesigner:boxSize width="38.0" height="18.0"/>

<celldesigner:singleLine width="1.0"/>

<celldesigner:paint color="ffffff00" scheme="Color"/>

</celldesigner:usualView>

<celldesigner:briefView>

<celldesigner:innerPosition x="0.0" y="0.0"/>

<celldesigner:boxSize width="80.0" height="60.0"/>

<celldesigner:singleLine width="1.0"/>

<celldesigner:paint color="3fff0000" scheme="Color"/>

</celldesigner:briefView>

<celldesigner:info state="empty" angle="-1.5707963267948966"/>

</celldesigner:speciesAlias>

<celldesigner:speciesAlias id="sa526" species="s1113">

<celldesigner:activity>inactive</celldesigner:activity>

<celldesigner:bounds x="635.125" y="1606.279265358071" w="38.0" h="18.0"/>

<celldesigner:font size="12"/>

<celldesigner:view state="usual"/>

<celldesigner:usualView>

<celldesigner:innerPosition x="0.0" y="0.0"/>

<celldesigner:boxSize width="38.0" height="18.0"/>

<celldesigner:singleLine width="1.0"/>

<celldesigner:paint color="ffccff66" scheme="Color"/>

</celldesigner:usualView>

<celldesigner:briefView>

<celldesigner:innerPosition x="0.0" y="0.0"/>

<celldesigner:boxSize width="80.0" height="60.0"/>

<celldesigner:singleLine width="1.0"/>

<celldesigner:paint color="3fff0000" scheme="Color"/>

</celldesigner:briefView>

<celldesigner:info state="empty" angle="-1.5707963267948966"/>

</celldesigner:speciesAlias>

<celldesigner:speciesAlias id="sa527" species="s442">

<celldesigner:activity>inactive</celldesigner:activity>

<celldesigner:bounds x="273.0" y="1600.5" w="38.0" h="18.0"/>

<celldesigner:font size="12"/>

<celldesigner:view state="usual"/>

<celldesigner:usualView>

<celldesigner:innerPosition x="0.0" y="0.0"/>

<celldesigner:boxSize width="38.0" height="18.0"/>

<celldesigner:singleLine width="1.0"/>

<celldesigner:paint color="ffccff66" scheme="Color"/>

</celldesigner:usualView>

<celldesigner:briefView>

<celldesigner:innerPosition x="0.0" y="0.0"/>

<celldesigner:boxSize width="80.0" height="60.0"/>

<celldesigner:singleLine width="1.0"/>

<celldesigner:paint color="3fff0000" scheme="Color"/>

</celldesigner:briefView>

<celldesigner:info state="empty" angle="-1.5707963267948966"/>

</celldesigner:speciesAlias>

<celldesigner:speciesAlias id="sa531" species="s64">

<celldesigner:activity>inactive</celldesigner:activity>

<celldesigner:bounds x="2187.5" y="689.0" w="45.0" h="22.0"/>

<celldesigner:font size="12"/>

<celldesigner:view state="usual"/>

<celldesigner:usualView>

<celldesigner:innerPosition x="0.0" y="0.0"/>

<celldesigner:boxSize width="45.0" height="22.0"/>

<celldesigner:singleLine width="1.0"/>

<celldesigner:paint color="ff009999" scheme="Color"/>

</celldesigner:usualView>

<celldesigner:briefView>

<celldesigner:innerPosition x="0.0" y="0.0"/>

<celldesigner:boxSize width="80.0" height="60.0"/>

<celldesigner:singleLine width="1.0"/>

<celldesigner:paint color="3fff0000" scheme="Color"/>

</celldesigner:briefView>

<celldesigner:info state="empty" angle="-1.5707963267948966"/>

</celldesigner:speciesAlias>

<celldesigner:speciesAlias id="sa532" species="s114">

<celldesigner:activity>inactive</celldesigner:activity>

<celldesigner:bounds x="1554.0" y="491.75" w="32.0" h="17.0"/>

<celldesigner:font size="12"/>

<celldesigner:view state="usual"/>

<celldesigner:usualView>

<celldesigner:innerPosition x="0.0" y="0.0"/>

<celldesigner:boxSize width="32.0" height="17.0"/>

<celldesigner:singleLine width="1.0"/>

<celldesigner:paint color="ff009999" scheme="Color"/>

</celldesigner:usualView>

<celldesigner:briefView>

<celldesigner:innerPosition x="0.0" y="0.0"/>

<celldesigner:boxSize width="80.0" height="60.0"/>

<celldesigner:singleLine width="1.0"/>

<celldesigner:paint color="3fff0000" scheme="Color"/>

</celldesigner:briefView>

<celldesigner:info state="empty" angle="-1.5707963267948966"/>

</celldesigner:speciesAlias>

<celldesigner:speciesAlias id="sa533" species="s115">

<celldesigner:activity>inactive</celldesigner:activity>

<celldesigner:bounds x="2055.5" y="528.5" w="79.0" h="28.0"/>

<celldesigner:font size="12"/>

<celldesigner:view state="usual"/>

<celldesigner:usualView>

<celldesigner:innerPosition x="0.0" y="0.0"/>

<celldesigner:boxSize width="79.0" height="28.0"/>

<celldesigner:singleLine width="1.0"/>

<celldesigner:paint color="ff006633" scheme="Color"/>

</celldesigner:usualView>

<celldesigner:briefView>

<celldesigner:innerPosition x="0.0" y="0.0"/>

<celldesigner:boxSize width="80.0" height="60.0"/>

<celldesigner:singleLine width="1.0"/>

<celldesigner:paint color="3fff0000" scheme="Color"/>

</celldesigner:briefView>

<celldesigner:info state="empty" angle="-1.5707963267948966"/>

</celldesigner:speciesAlias>

<celldesigner:speciesAlias id="sa534" species="s471">

<celldesigner:activity>inactive</celldesigner:activity>

<celldesigner:bounds x="2642.0" y="458.5" w="45.0" h="20.0"/>

<celldesigner:font size="12"/>

<celldesigner:view state="usual"/>

<celldesigner:usualView>

<celldesigner:innerPosition x="0.0" y="0.0"/>

<celldesigner:boxSize width="45.0" height="20.0"/>

<celldesigner:singleLine width="1.0"/>

<celldesigner:paint color="ffcc0066" scheme="Color"/>

</celldesigner:usualView>

<celldesigner:briefView>

<celldesigner:innerPosition x="0.0" y="0.0"/>

<celldesigner:boxSize width="80.0" height="60.0"/>

<celldesigner:singleLine width="1.0"/>

<celldesigner:paint color="3fff0000" scheme="Color"/>

</celldesigner:briefView>

<celldesigner:info state="empty" angle="-1.5707963267948966"/>

</celldesigner:speciesAlias>

<celldesigner:speciesAlias id="sa535" species="s472">

<celldesigner:activity>inactive</celldesigner:activity>

<celldesigner:bounds x="2532.0" y="397.25" w="45.0" h="22.5"/>

<celldesigner:font size="12"/>

<celldesigner:view state="usual"/>

<celldesigner:usualView>

<celldesigner:innerPosition x="0.0" y="0.0"/>

<celldesigner:boxSize width="45.0" height="22.5"/>

<celldesigner:singleLine width="1.0"/>

<celldesigner:paint color="ffccff66" scheme="Color"/>

</celldesigner:usualView>

<celldesigner:briefView>

<celldesigner:innerPosition x="0.0" y="0.0"/>

<celldesigner:boxSize width="80.0" height="60.0"/>

<celldesigner:singleLine width="1.0"/>

<celldesigner:paint color="3fff0000" scheme="Color"/>

</celldesigner:briefView>

<celldesigner:info state="empty" angle="-1.5707963267948966"/>

</celldesigner:speciesAlias>

<celldesigner:speciesAlias id="sa536" species="s473">

<celldesigner:activity>inactive</celldesigner:activity>

<celldesigner:bounds x="2378.5" y="738.0" w="32.0" h="21.0"/>

<celldesigner:font size="12"/>

<celldesigner:view state="usual"/>

<celldesigner:usualView>

<celldesigner:innerPosition x="0.0" y="0.0"/>

<celldesigner:boxSize width="32.0" height="21.0"/>

<celldesigner:singleLine width="1.0"/>

<celldesigner:paint color="ff009999" scheme="Color"/>

</celldesigner:usualView>

<celldesigner:briefView>

<celldesigner:innerPosition x="0.0" y="0.0"/>

<celldesigner:boxSize width="80.0" height="60.0"/>

<celldesigner:singleLine width="1.0"/>

<celldesigner:paint color="3fff0000" scheme="Color"/>

</celldesigner:briefView>

<celldesigner:info state="empty" angle="-1.5707963267948966"/>

</celldesigner:speciesAlias>

<celldesigner:speciesAlias id="sa537" species="s475">

<celldesigner:activity>inactive</celldesigner:activity>

<celldesigner:bounds x="2654.0" y="619.5" w="32.0" h="21.0"/>

<celldesigner:font size="12"/>

<celldesigner:view state="usual"/>

<celldesigner:usualView>

<celldesigner:innerPosition x="0.0" y="0.0"/>

<celldesigner:boxSize width="32.0" height="21.0"/>

<celldesigner:singleLine width="1.0"/>

<celldesigner:paint color="ffcc0066" scheme="Color"/>

</celldesigner:usualView>

<celldesigner:briefView>

<celldesigner:innerPosition x="0.0" y="0.0"/>

<celldesigner:boxSize width="80.0" height="60.0"/>

<celldesigner:singleLine width="1.0"/>

<celldesigner:paint color="3fff0000" scheme="Color"/>

</celldesigner:briefView>

<celldesigner:info state="empty" angle="-1.5707963267948966"/>

</celldesigner:speciesAlias>

<celldesigner:speciesAlias id="sa538" species="s444">

<celldesigner:activity>inactive</celldesigner:activity>

<celldesigner:bounds x="2382.0" y="617.5" w="45.0" h="22.0"/>

<celldesigner:font size="12"/>

<celldesigner:view state="usual"/>

<celldesigner:usualView>

<celldesigner:innerPosition x="0.0" y="0.0"/>

<celldesigner:boxSize width="45.0" height="22.0"/>

<celldesigner:singleLine width="1.0"/>

<celldesigner:paint color="ff006633" scheme="Color"/>

</celldesigner:usualView>

<celldesigner:briefView>

<celldesigner:innerPosition x="0.0" y="0.0"/>

<celldesigner:boxSize width="80.0" height="60.0"/>

<celldesigner:singleLine width="1.0"/>

<celldesigner:paint color="3fff0000" scheme="Color"/>

</celldesigner:briefView>

<celldesigner:info state="empty" angle="-1.5707963267948966"/>

</celldesigner:speciesAlias>

<celldesigner:speciesAlias id="sa539" species="s16">

<celldesigner:activity>inactive</celldesigner:activity>

<celldesigner:bounds x="2247.0" y="527.5" w="45.0" h="22.0"/>

<celldesigner:font size="12"/>

<celldesigner:view state="usual"/>

<celldesigner:usualView>

<celldesigner:innerPosition x="0.0" y="0.0"/>

<celldesigner:boxSize width="45.0" height="22.0"/>

<celldesigner:singleLine width="1.0"/>

<celldesigner:paint color="ffff00ff" scheme="Color"/>

</celldesigner:usualView>

<celldesigner:briefView>

<celldesigner:innerPosition x="0.0" y="0.0"/>

<celldesigner:boxSize width="80.0" height="60.0"/>

<celldesigner:singleLine width="1.0"/>

<celldesigner:paint color="3fff0000" scheme="Color"/>

</celldesigner:briefView>

<celldesigner:info state="empty" angle="-1.5707963267948966"/>

</celldesigner:speciesAlias>

<celldesigner:speciesAlias id="sa540" species="s446">

<celldesigner:activity>inactive</celldesigner:activity>

<celldesigner:bounds x="2188.5" y="298.0" w="32.0" h="21.0"/>

<celldesigner:font size="12"/>

<celldesigner:view state="usual"/>

<celldesigner:usualView>

<celldesigner:innerPosition x="0.0" y="0.0"/>

<celldesigner:boxSize width="32.0" height="21.0"/>

<celldesigner:singleLine width="1.0"/>

<celldesigner:paint color="ffcc0066" scheme="Color"/>

</celldesigner:usualView>

<celldesigner:briefView>

<celldesigner:innerPosition x="0.0" y="0.0"/>

<celldesigner:boxSize width="80.0" height="60.0"/>

<celldesigner:singleLine width="1.0"/>

<celldesigner:paint color="3fff0000" scheme="Color"/>

</celldesigner:briefView>

<celldesigner:info state="empty" angle="-1.5707963267948966"/>

</celldesigner:speciesAlias>

<celldesigner:speciesAlias id="sa541" species="s979">

<celldesigner:activity>inactive</celldesigner:activity>

<celldesigner:bounds x="1204.0" y="1149.5" w="32.0" h="21.0"/>

<celldesigner:font size="12"/>

<celldesigner:view state="usual"/>

<celldesigner:usualView>

<celldesigner:innerPosition x="0.0" y="0.0"/>

<celldesigner:boxSize width="32.0" height="21.0"/>

<celldesigner:singleLine width="1.0"/>

<celldesigner:paint color="ffcc0066" scheme="Color"/>

</celldesigner:usualView>

<celldesigner:briefView>

<celldesigner:innerPosition x="0.0" y="0.0"/>

<celldesigner:boxSize width="80.0" height="60.0"/>

<celldesigner:singleLine width="1.0"/>

<celldesigner:paint color="3fff0000" scheme="Color"/>

</celldesigner:briefView>

<celldesigner:info state="empty" angle="-1.5707963267948966"/>

</celldesigner:speciesAlias>

<celldesigner:speciesAlias id="sa542" species="s490">

<celldesigner:activity>inactive</celldesigner:activity>

<celldesigner:bounds x="2378.25" y="838.0" w="32.5" h="21.0"/>

<celldesigner:font size="12"/>

<celldesigner:view state="usual"/>

<celldesigner:usualView>
<celldesigner:innerPosition x="0.0" y="0.0"/>
<celldesigner:boxSize width="32.5" height="21.0"/>
<celldesigner:singleLine width="1.0"/>
<celldesigner:paint color="ffcc0066" scheme="Color"/>
</celldesigner:usualView>
<celldesigner:briefView>
<celldesigner:innerPosition x="0.0" y="0.0"/>
<celldesigner:boxSize width="80.0" height="60.0"/>
<celldesigner:singleLine width="1.0"/>
<celldesigner:paint color="3fff0000" scheme="Color"/>
</celldesigner:briefView>
<celldesigner:info state="empty" angle="-1.5707963267948966"/>
</celldesigner:speciesAlias>
<celldesigner:speciesAlias id="sa543" species="s489">
<celldesigner:activity>inactive</celldesigner:activity>
<celldesigner:bounds x="2332.0" y="787.5" w="45.0" h="22.0"/>
<celldesigner:font size="12"/>
<celldesigner:view state="usual"/>
<celldesigner:usualView>
<celldesigner:innerPosition x="0.0" y="0.0"/>
<celldesigner:boxSize width="45.0" height="22.0"/>
<celldesigner:singleLine width="1.0"/>
<celldesigner:paint color="ff006633" scheme="Color"/>
</celldesigner:usualView>
<celldesigner:briefView>
<celldesigner:innerPosition x="0.0" y="0.0"/>

<celldesigner:boxSize width="80.0" height="60.0"/>
<celldesigner:singleLine width="1.0"/>
<celldesigner:paint color="3fff0000" scheme="Color"/>
</celldesigner:briefView>
<celldesigner:info state="empty" angle="-1.5707963267948966"/>
</celldesigner:speciesAlias>
<celldesigner:speciesAlias id="sa544" species="s198">
<celldesigner:activity>inactive</celldesigner:activity>
<celldesigner:bounds x="2373.0" y="1200.5" w="43.0" h="16.0"/>
<celldesigner:font size="12"/>
<celldesigner:view state="usual"/>
<celldesigner:usualView>
<celldesigner:innerPosition x="0.0" y="0.0"/>
<celldesigner:boxSize width="43.0" height="16.0"/>
<celldesigner:singleLine width="1.0"/>
<celldesigner:paint color="ffcc0066" scheme="Color"/>
</celldesigner:usualView>
<celldesigner:briefView>
<celldesigner:innerPosition x="0.0" y="0.0"/>
<celldesigner:boxSize width="80.0" height="60.0"/>
<celldesigner:singleLine width="1.0"/>
<celldesigner:paint color="3fff0000" scheme="Color"/>
</celldesigner:briefView>
<celldesigner:info state="empty" angle="-1.5707963267948966"/>
</celldesigner:speciesAlias>
<celldesigner:speciesAlias id="sa545" species="s231">
<celldesigner:activity>inactive</celldesigner:activity>

<celldesigner:bounds x="1644.25" y="281.25" w="31.0" h="18.0"/>

<celldesigner:font size="12"/>

<celldesigner:view state="usual"/>

<celldesigner:usualView>

<celldesigner:innerPosition x="0.0" y="0.0"/>

<celldesigner:boxSize width="31.0" height="18.0"/>

<celldesigner:singleLine width="1.0"/>

<celldesigner:paint color="ff006633" scheme="Color"/>

</celldesigner:usualView>

<celldesigner:briefView>

<celldesigner:innerPosition x="0.0" y="0.0"/>

<celldesigner:boxSize width="80.0" height="60.0"/>

<celldesigner:singleLine width="1.0"/>

<celldesigner:paint color="3fff0000" scheme="Color"/>

</celldesigner:briefView>

<celldesigner:info state="empty" angle="-1.5707963267948966"/>

</celldesigner:speciesAlias>

<celldesigner:speciesAlias id="sa546" species="s217">

<celldesigner:activity>inactive</celldesigner:activity>

<celldesigner:bounds x="2068.5" y="692.0" w="43.0" h="16.0"/>

<celldesigner:font size="12"/>

<celldesigner:view state="usual"/>

<celldesigner:usualView>

<celldesigner:innerPosition x="0.0" y="0.0"/>

<celldesigner:boxSize width="43.0" height="16.0"/>

<celldesigner:singleLine width="1.0"/>

<celldesigner:paint color="ff006633" scheme="Color"/>

```
</celldesigner:usualView>
<celldesigner:briefView>
<celldesigner:innerPosition x="0.0" y="0.0"/>
<celldesigner:boxSize width="80.0" height="60.0"/>
<celldesigner:singleLine width="1.0"/>
<celldesigner:paint color="3fff0000" scheme="Color"/>
</celldesigner:briefView>
<celldesigner:info state="empty" angle="-1.5707963267948966"/>
</celldesigner:speciesAlias>
<celldesigner:speciesAlias id="sa547" species="s525">
<celldesigner:activity>inactive</celldesigner:activity>
<celldesigner:bounds x="2538.25" y="307.875" w="32.0" h="21.0"/>
<celldesigner:font size="12"/>
<celldesigner:view state="usual"/>
<celldesigner:usualView>
<celldesigner:innerPosition x="0.0" y="0.0"/>
<celldesigner:boxSize width="32.0" height="21.0"/>
<celldesigner:singleLine width="1.0"/>
<celldesigner:paint color="ff006633" scheme="Color"/>
</celldesigner:usualView>
<celldesigner:briefView>
<celldesigner:innerPosition x="0.0" y="0.0"/>
<celldesigner:boxSize width="80.0" height="60.0"/>
<celldesigner:singleLine width="1.0"/>
<celldesigner:paint color="3fff0000" scheme="Color"/>
</celldesigner:briefView>
<celldesigner:info state="empty" angle="-1.5707963267948966"/>
```

</celldesigner:speciesAlias>

<celldesigner:speciesAlias id="sa548" species="s526">

<celldesigner:activity>inactive</celldesigner:activity>

<celldesigner:bounds x="2528.5" y="248.0" w="32.0" h="21.0"/>

<celldesigner:font size="12"/>

<celldesigner:view state="usual"/>

<celldesigner:usualView>

<celldesigner:innerPosition x="0.0" y="0.0"/>

<celldesigner:boxSize width="32.0" height="21.0"/>

<celldesigner:singleLine width="1.0"/>

<celldesigner:paint color="ff006633" scheme="Color"/>

</celldesigner:usualView>

<celldesigner:briefView>

<celldesigner:innerPosition x="0.0" y="0.0"/>

<celldesigner:boxSize width="80.0" height="60.0"/>

<celldesigner:singleLine width="1.0"/>

<celldesigner:paint color="3fff0000" scheme="Color"/>

</celldesigner:briefView>

<celldesigner:info state="empty" angle="-1.5707963267948966"/>

</celldesigner:speciesAlias>

<celldesigner:speciesAlias id="sa549" species="s527">

<celldesigner:activity>inactive</celldesigner:activity>

<celldesigner:bounds x="2538.5" y="198.0" w="32.0" h="21.0"/>

<celldesigner:font size="12"/>

<celldesigner:view state="usual"/>

<celldesigner:usualView>

<celldesigner:innerPosition x="0.0" y="0.0"/>

<celldesigner:boxSize width="32.0" height="21.0"/>

<celldesigner:singleLine width="1.0"/>

<celldesigner:paint color="ff006633" scheme="Color"/>

</celldesigner:usualView>

<celldesigner:briefView>

<celldesigner:innerPosition x="0.0" y="0.0"/>

<celldesigner:boxSize width="80.0" height="60.0"/>

<celldesigner:singleLine width="1.0"/>

<celldesigner:paint color="3fff0000" scheme="Color"/>

</celldesigner:briefView>

<celldesigner:info state="empty" angle="-1.5707963267948966"/>

</celldesigner:speciesAlias>

<celldesigner:speciesAlias id="sa550" species="s528">

<celldesigner:activity>inactive</celldesigner:activity>

<celldesigner:bounds x="2627.5" y="389.0" w="45.0" h="22.0"/>

<celldesigner:font size="12"/>

<celldesigner:view state="usual"/>

<celldesigner:usualView>

<celldesigner:innerPosition x="0.0" y="0.0"/>

<celldesigner:boxSize width="45.0" height="22.0"/>

<celldesigner:singleLine width="1.0"/>

<celldesigner:paint color="ff006633" scheme="Color"/>

</celldesigner:usualView>

<celldesigner:briefView>

<celldesigner:innerPosition x="0.0" y="0.0"/>

<celldesigner:boxSize width="80.0" height="60.0"/>

<celldesigner:singleLine width="1.0"/>

<celldesigner:paint color="3fff0000" scheme="Color"/>

</celldesigner:briefView>

<celldesigner:info state="empty" angle="-1.5707963267948966"/>

</celldesigner:speciesAlias>

<celldesigner:speciesAlias id="sa551" species="s834">

<celldesigner:activity>inactive</celldesigner:activity>

<celldesigner:bounds x="2748.75" y="389.375" w="62.5" h="21.25"/>

<celldesigner:font size="12"/>

<celldesigner:view state="usual"/>

<celldesigner:usualView>

<celldesigner:innerPosition x="0.0" y="0.0"/>

<celldesigner:boxSize width="62.5" height="21.25"/>

<celldesigner:singleLine width="1.0"/>

<celldesigner:paint color="ffccff66" scheme="Color"/>

</celldesigner:usualView>

<celldesigner:briefView>

<celldesigner:innerPosition x="0.0" y="0.0"/>

<celldesigner:boxSize width="80.0" height="60.0"/>

<celldesigner:singleLine width="1.0"/>

<celldesigner:paint color="3fff0000" scheme="Color"/>

</celldesigner:briefView>

<celldesigner:info state="empty" angle="-1.5707963267948966"/>

</celldesigner:speciesAlias>

<celldesigner:speciesAlias id="sa552" species="s215">

<celldesigner:activity>inactive</celldesigner:activity>

<celldesigner:bounds x="1998.5" y="682.0" w="43.0" h="16.0"/>

<celldesigner:font size="12"/>

<celldesigner:view state="usual"/>

<celldesigner:usualView>

<celldesigner:innerPosition x="0.0" y="0.0"/>

<celldesigner:boxSize width="43.0" height="16.0"/>

<celldesigner:singleLine width="1.0"/>

<celldesigner:paint color="ff006633" scheme="Color"/>

</celldesigner:usualView>

<celldesigner:briefView>

<celldesigner:innerPosition x="0.0" y="0.0"/>

<celldesigner:boxSize width="80.0" height="60.0"/>

<celldesigner:singleLine width="1.0"/>

<celldesigner:paint color="3fff0000" scheme="Color"/>

</celldesigner:briefView>

<celldesigner:info state="empty" angle="-1.5707963267948966"/>

</celldesigner:speciesAlias>

<celldesigner:speciesAlias id="sa553" species="s835">

<celldesigner:activity>inactive</celldesigner:activity>

<celldesigner:bounds x="2187.0" y="607.5" w="55.0" h="22.0"/>

<celldesigner:font size="12"/>

<celldesigner:view state="usual"/>

<celldesigner:usualView>

<celldesigner:innerPosition x="0.0" y="0.0"/>

<celldesigner:boxSize width="55.0" height="22.0"/>

<celldesigner:singleLine width="1.0"/>

<celldesigner:paint color="ff006633" scheme="Color"/>

</celldesigner:usualView>

<celldesigner:briefView>

<celldesigner:innerPosition x="0.0" y="0.0"/>
<celldesigner:boxSize width="80.0" height="60.0"/>
<celldesigner:singleLine width="1.0"/>
<celldesigner:paint color="3fff0000" scheme="Color"/>
</celldesigner:briefView>
<celldesigner:info state="empty" angle="-1.5707963267948966"/>
</celldesigner:speciesAlias>
<celldesigner:speciesAlias id="sa554" species="s836">
<celldesigner:activity>inactive</celldesigner:activity>
<celldesigner:bounds x="2292.0" y="597.25" w="45.0" h="22.0"/>
<celldesigner:font size="12"/>
<celldesigner:view state="usual"/>
<celldesigner:usualView>
<celldesigner:innerPosition x="0.0" y="0.0"/>
<celldesigner:boxSize width="45.0" height="22.0"/>
<celldesigner:singleLine width="1.0"/>
<celldesigner:paint color="ff006633" scheme="Color"/>
</celldesigner:usualView>
<celldesigner:briefView>
<celldesigner:innerPosition x="0.0" y="0.0"/>
<celldesigner:boxSize width="80.0" height="60.0"/>
<celldesigner:singleLine width="1.0"/>
<celldesigner:paint color="3fff0000" scheme="Color"/>
</celldesigner:briefView>
<celldesigner:info state="empty" angle="-1.5707963267948966"/>
</celldesigner:speciesAlias>
<celldesigner:speciesAlias id="sa555" species="s837">

<celldesigner:activity>inactive</celldesigner:activity>

<celldesigner:bounds x="2522.5" y="619.0" w="55.0" h="22.0"/>

<celldesigner:font size="12"/>

<celldesigner:view state="usual"/>

<celldesigner:usualView>

<celldesigner:innerPosition x="0.0" y="0.0"/>

<celldesigner:boxSize width="55.0" height="22.0"/>

<celldesigner:singleLine width="1.0"/>

<celldesigner:paint color="ff006633" scheme="Color"/>

</celldesigner:usualView>

<celldesigner:briefView>

<celldesigner:innerPosition x="0.0" y="0.0"/>

<celldesigner:boxSize width="80.0" height="60.0"/>

<celldesigner:singleLine width="1.0"/>

<celldesigner:paint color="3fff0000" scheme="Color"/>

</celldesigner:briefView>

<celldesigner:info state="empty" angle="-1.5707963267948966"/>

</celldesigner:speciesAlias>

<celldesigner:speciesAlias id="sa556" species="s1004">

<celldesigner:activity>inactive</celldesigner:activity>

<celldesigner:bounds x="2379.0" y="1127.5" w="31.0" h="22.0"/>

<celldesigner:font size="12"/>

<celldesigner:view state="usual"/>

<celldesigner:usualView>

<celldesigner:innerPosition x="0.0" y="0.0"/>

<celldesigner:boxSize width="31.0" height="22.0"/>

<celldesigner:singleLine width="1.0"/>

<celldesigner:paint color="ff009999" scheme="Color"/>

</celldesigner:usualView>

<celldesigner:briefView>

<celldesigner:innerPosition x="0.0" y="0.0"/>

<celldesigner:boxSize width="80.0" height="60.0"/>

<celldesigner:singleLine width="1.0"/>

<celldesigner:paint color="3fff0000" scheme="Color"/>

</celldesigner:briefView>

<celldesigner:info state="empty" angle="-1.5707963267948966"/>

</celldesigner:speciesAlias>

<celldesigner:speciesAlias id="sa557" species="s840">

<celldesigner:activity>inactive</celldesigner:activity>

<celldesigner:bounds x="2372.0" y="1057.5" w="45.0" h="22.0"/>

<celldesigner:font size="12"/>

<celldesigner:view state="usual"/>

<celldesigner:usualView>

<celldesigner:innerPosition x="0.0" y="0.0"/>

<celldesigner:boxSize width="45.0" height="22.0"/>

<celldesigner:singleLine width="1.0"/>

<celldesigner:paint color="ff006633" scheme="Color"/>

</celldesigner:usualView>

<celldesigner:briefView>

<celldesigner:innerPosition x="0.0" y="0.0"/>

<celldesigner:boxSize width="80.0" height="60.0"/>

<celldesigner:singleLine width="1.0"/>

<celldesigner:paint color="3fff0000" scheme="Color"/>

</celldesigner:briefView>

<celldesigner:info state="empty" angle="-1.5707963267948966"/>
</celldesigner:speciesAlias>
<celldesigner:speciesAlias id="sa558" species="s841">
<celldesigner:activity>inactive</celldesigner:activity>
<celldesigner:bounds x="2377.0" y="997.5" w="35.0" h="22.0"/>
<celldesigner:font size="12"/>
<celldesigner:view state="usual"/>
<celldesigner:usualView>
<celldesigner:innerPosition x="0.0" y="0.0"/>
<celldesigner:boxSize width="35.0" height="22.0"/>
<celldesigner:singleLine width="1.0"/>
<celldesigner:paint color="ff006633" scheme="Color"/>
</celldesigner:usualView>
<celldesigner:briefView>
<celldesigner:innerPosition x="0.0" y="0.0"/>
<celldesigner:boxSize width="80.0" height="60.0"/>
<celldesigner:singleLine width="1.0"/>
<celldesigner:paint color="3fff0000" scheme="Color"/>
</celldesigner:briefView>
<celldesigner:info state="empty" angle="-1.5707963267948966"/>
</celldesigner:speciesAlias>
<celldesigner:speciesAlias id="sa559" species="s842">
<celldesigner:activity>inactive</celldesigner:activity>
<celldesigner:bounds x="2377.0" y="907.5" w="35.0" h="22.0"/>
<celldesigner:font size="12"/>
<celldesigner:view state="usual"/>
<celldesigner:usualView>

<celldesigner:innerPosition x="0.0" y="0.0"/>
<celldesigner:boxSize width="35.0" height="22.0"/>
<celldesigner:singleLine width="1.0"/>
<celldesigner:paint color="ff006633" scheme="Color"/>
</celldesigner:usualView>
<celldesigner:briefView>
<celldesigner:innerPosition x="0.0" y="0.0"/>
<celldesigner:boxSize width="80.0" height="60.0"/>
<celldesigner:singleLine width="1.0"/>
<celldesigner:paint color="3fff0000" scheme="Color"/>
</celldesigner:briefView>
<celldesigner:info state="empty" angle="-1.5707963267948966"/>
</celldesigner:speciesAlias>
<celldesigner:speciesAlias id="sa560" species="s843">
<celldesigner:activity>inactive</celldesigner:activity>
<celldesigner:bounds x="2177.0" y="367.5" w="35.0" h="22.0"/>
<celldesigner:font size="12"/>
<celldesigner:view state="usual"/>
<celldesigner:usualView>
<celldesigner:innerPosition x="0.0" y="0.0"/>
<celldesigner:boxSize width="35.0" height="22.0"/>
<celldesigner:singleLine width="1.0"/>
<celldesigner:paint color="ff006633" scheme="Color"/>
</celldesigner:usualView>
<celldesigner:briefView>
<celldesigner:innerPosition x="0.0" y="0.0"/>
<celldesigner:boxSize width="80.0" height="60.0"/>

<celldesigner:singleLine width="1.0"/>

<celldesigner:paint color="3fff0000" scheme="Color"/>

</celldesigner:briefView>

<celldesigner:info state="empty" angle="-1.5707963267948966"/>

</celldesigner:speciesAlias>

<celldesigner:speciesAlias id="sa561" species="s844">

<celldesigner:activity>inactive</celldesigner:activity>

<celldesigner:bounds x="2507.0" y="577.5" w="55.0" h="22.0"/>

<celldesigner:font size="12"/>

<celldesigner:view state="usual"/>

<celldesigner:usualView>

<celldesigner:innerPosition x="0.0" y="0.0"/>

<celldesigner:boxSize width="55.0" height="22.0"/>

<celldesigner:singleLine width="1.0"/>

<celldesigner:paint color="ff006633" scheme="Color"/>

</celldesigner:usualView>

<celldesigner:briefView>

<celldesigner:innerPosition x="0.0" y="0.0"/>

<celldesigner:boxSize width="80.0" height="60.0"/>

<celldesigner:singleLine width="1.0"/>

<celldesigner:paint color="3fff0000" scheme="Color"/>

</celldesigner:briefView>

<celldesigner:info state="empty" angle="-1.5707963267948966"/>

</celldesigner:speciesAlias>

<celldesigner:speciesAlias id="sa562" species="s845">

<celldesigner:activity>inactive</celldesigner:activity>

<celldesigner:bounds x="2607.5" y="549.0" w="45.0" h="22.0"/>

<celldesigner:font size="12"/>

<celldesigner:view state="usual"/>

<celldesigner:usualView>

<celldesigner:innerPosition x="0.0" y="0.0"/>

<celldesigner:boxSize width="45.0" height="22.0"/>

<celldesigner:singleLine width="1.0"/>

<celldesigner:paint color="ff006633" scheme="Color"/>

</celldesigner:usualView>

<celldesigner:briefView>

<celldesigner:innerPosition x="0.0" y="0.0"/>

<celldesigner:boxSize width="80.0" height="60.0"/>

<celldesigner:singleLine width="1.0"/>

<celldesigner:paint color="3fff0000" scheme="Color"/>

</celldesigner:briefView>

<celldesigner:info state="empty" angle="-1.5707963267948966"/>

</celldesigner:speciesAlias>

<celldesigner:speciesAlias id="sa563" species="s846">

<celldesigner:activity>inactive</celldesigner:activity>

<celldesigner:bounds x="2007.5" y="619.0" w="45.0" h="22.0"/>

<celldesigner:font size="12"/>

<celldesigner:view state="usual"/>

<celldesigner:usualView>

<celldesigner:innerPosition x="0.0" y="0.0"/>

<celldesigner:boxSize width="45.0" height="22.0"/>

<celldesigner:singleLine width="1.0"/>

<celldesigner:paint color="ff006633" scheme="Color"/>

</celldesigner:usualView>

<celldesigner:briefView>

<celldesigner:innerPosition x="0.0" y="0.0"/>

<celldesigner:boxSize width="80.0" height="60.0"/>

<celldesigner:singleLine width="1.0"/>

<celldesigner:paint color="3fff0000" scheme="Color"/>

</celldesigner:briefView>

<celldesigner:info state="empty" angle="-1.5707963267948966"/>

</celldesigner:speciesAlias>

<celldesigner:speciesAlias id="sa564" species="s847">

<celldesigner:activity>inactive</celldesigner:activity>

<celldesigner:bounds x="2062.5" y="738.75" w="35.0" h="22.5"/>

<celldesigner:font size="12"/>

<celldesigner:view state="usual"/>

<celldesigner:usualView>

<celldesigner:innerPosition x="0.0" y="0.0"/>

<celldesigner:boxSize width="35.0" height="22.5"/>

<celldesigner:singleLine width="1.0"/>

<celldesigner:paint color="ffccff66" scheme="Color"/>

</celldesigner:usualView>

<celldesigner:briefView>

<celldesigner:innerPosition x="0.0" y="0.0"/>

<celldesigner:boxSize width="80.0" height="60.0"/>

<celldesigner:singleLine width="1.0"/>

<celldesigner:paint color="3fff0000" scheme="Color"/>

</celldesigner:briefView>

<celldesigner:info state="empty" angle="-1.5707963267948966"/>

</celldesigner:speciesAlias>

<celldesigner:speciesAlias id="sa565" species="s848">

<celldesigner:activity>inactive</celldesigner:activity>

<celldesigner:bounds x="1895.0" y="217.5" w="70.0" h="25.0"/>

<celldesigner:font size="12"/>

<celldesigner:view state="usual"/>

<celldesigner:usualView>

<celldesigner:innerPosition x="0.0" y="0.0"/>

<celldesigner:boxSize width="70.0" height="25.0"/>

<celldesigner:singleLine width="1.0"/>

<celldesigner:paint color="ff006633" scheme="Color"/>

</celldesigner:usualView>

<celldesigner:briefView>

<celldesigner:innerPosition x="0.0" y="0.0"/>

<celldesigner:boxSize width="80.0" height="60.0"/>

<celldesigner:singleLine width="1.0"/>

<celldesigner:paint color="3fff0000" scheme="Color"/>

</celldesigner:briefView>

<celldesigner:info state="empty" angle="-1.5707963267948966"/>

</celldesigner:speciesAlias>

<celldesigner:speciesAlias id="sa566" species="s849">

<celldesigner:activity>inactive</celldesigner:activity>

<celldesigner:bounds x="1877.5" y="328.75" w="45.0" h="22.5"/>

<celldesigner:font size="12"/>

<celldesigner:view state="usual"/>

<celldesigner:usualView>

<celldesigner:innerPosition x="0.0" y="0.0"/>

<celldesigner:boxSize width="45.0" height="22.5"/>

<celldesigner:singleLine width="1.0"/>

<celldesigner:paint color="ffccff66" scheme="Color"/>

</celldesigner:usualView>

<celldesigner:briefView>

<celldesigner:innerPosition x="0.0" y="0.0"/>

<celldesigner:boxSize width="80.0" height="60.0"/>

<celldesigner:singleLine width="1.0"/>

<celldesigner:paint color="3fff0000" scheme="Color"/>

</celldesigner:briefView>

<celldesigner:info state="empty" angle="-1.5707963267948966"/>

</celldesigner:speciesAlias>

<celldesigner:speciesAlias id="sa567" species="s850">

<celldesigner:activity>inactive</celldesigner:activity>

<celldesigner:bounds x="2015.0" y="267.5" w="70.0" h="25.0"/>

<celldesigner:font size="12"/>

<celldesigner:view state="usual"/>

<celldesigner:usualView>

<celldesigner:innerPosition x="0.0" y="0.0"/>

<celldesigner:boxSize width="70.0" height="25.0"/>

<celldesigner:singleLine width="1.0"/>

<celldesigner:paint color="ff006633" scheme="Color"/>

</celldesigner:usualView>

<celldesigner:briefView>

<celldesigner:innerPosition x="0.0" y="0.0"/>

<celldesigner:boxSize width="80.0" height="60.0"/>

<celldesigner:singleLine width="1.0"/>

<celldesigner:paint color="3fff0000" scheme="Color"/>

</celldesigner:briefView>

<celldesigner:info state="empty" angle="-1.5707963267948966"/>

</celldesigner:speciesAlias>

<celldesigner:speciesAlias id="sa568" species="s851">

<celldesigner:activity>inactive</celldesigner:activity>

<celldesigner:bounds x="2127.5" y="258.75" w="45.0" h="22.5"/>

<celldesigner:font size="12"/>

<celldesigner:view state="usual"/>

<celldesigner:usualView>

<celldesigner:innerPosition x="0.0" y="0.0"/>

<celldesigner:boxSize width="45.0" height="22.5"/>

<celldesigner:singleLine width="1.0"/>

<celldesigner:paint color="ffccff66" scheme="Color"/>

</celldesigner:usualView>

<celldesigner:briefView>

<celldesigner:innerPosition x="0.0" y="0.0"/>

<celldesigner:boxSize width="80.0" height="60.0"/>

<celldesigner:singleLine width="1.0"/>

<celldesigner:paint color="3fff0000" scheme="Color"/>

</celldesigner:briefView>

<celldesigner:info state="empty" angle="-1.5707963267948966"/>

</celldesigner:speciesAlias>

<celldesigner:speciesAlias id="sa569" species="s852">

<celldesigner:activity>inactive</celldesigner:activity>

<celldesigner:bounds x="2245.0" y="357.5" w="70.0" h="25.0"/>

<celldesigner:font size="12"/>

<celldesigner:view state="usual"/>

<celldesigner:usualView>
<celldesigner:innerPosition x="0.0" y="0.0"/>
<celldesigner:boxSize width="70.0" height="25.0"/>
<celldesigner:singleLine width="1.0"/>
<celldesigner:paint color="ff006633" scheme="Color"/>
</celldesigner:usualView>
<celldesigner:briefView>
<celldesigner:innerPosition x="0.0" y="0.0"/>
<celldesigner:boxSize width="80.0" height="60.0"/>
<celldesigner:singleLine width="1.0"/>
<celldesigner:paint color="3fff0000" scheme="Color"/>
</celldesigner:briefView>
<celldesigner:info state="empty" angle="-1.5707963267948966"/>
</celldesigner:speciesAlias>
<celldesigner:speciesAlias id="sa570" species="s853">
<celldesigner:activity>inactive</celldesigner:activity>
<celldesigner:bounds x="2227.5" y="458.75" w="45.0" h="22.5"/>
<celldesigner:font size="12"/>
<celldesigner:view state="usual"/>
<celldesigner:usualView>
<celldesigner:innerPosition x="0.0" y="0.0"/>
<celldesigner:boxSize width="45.0" height="22.5"/>
<celldesigner:singleLine width="1.0"/>
<celldesigner:paint color="ffccff66" scheme="Color"/>
</celldesigner:usualView>
<celldesigner:briefView>
<celldesigner:innerPosition x="0.0" y="0.0"/>

<celldesigner:boxSize width="80.0" height="60.0"/>
<celldesigner:singleLine width="1.0"/>
<celldesigner:paint color="3fff0000" scheme="Color"/>
</celldesigner:briefView>
<celldesigner:info state="empty" angle="-1.5707963267948966"/>
</celldesigner:speciesAlias>
<celldesigner:speciesAlias id="sa571" species="s256">
<celldesigner:activity>inactive</celldesigner:activity>
<celldesigner:bounds x="1633.0" y="349.25" w="57.0" h="17.0"/>
<celldesigner:font size="12"/>
<celldesigner:view state="usual"/>
<celldesigner:usualView>
<celldesigner:innerPosition x="2162.5" y="735.0"/>
<celldesigner:boxSize width="57.0" height="17.0"/>
<celldesigner:singleLine width="1.0"/>
<celldesigner:paint color="ff006633" scheme="Color"/>
</celldesigner:usualView>
<celldesigner:briefView>
<celldesigner:innerPosition x="0.0" y="0.0"/>
<celldesigner:boxSize width="80.0" height="60.0"/>
<celldesigner:singleLine width="1.0"/>
<celldesigner:paint color="3fff0000" scheme="Color"/>
</celldesigner:briefView>
<celldesigner:info state="empty" angle="-1.5707963267948966"/>
</celldesigner:speciesAlias>
<celldesigner:speciesAlias id="sa572" species="s257">
<celldesigner:activity>inactive</celldesigner:activity>

<celldesigner:bounds x="1631.5" y="431.75" w="57.0" h="17.0"/>

<celldesigner:font size="12"/>

<celldesigner:view state="usual"/>

<celldesigner:usualView>

<celldesigner:innerPosition x="2288.5" y="748.0"/>

<celldesigner:boxSize width="57.0" height="17.0"/>

<celldesigner:singleLine width="1.0"/>

<celldesigner:paint color="ff006633" scheme="Color"/>

</celldesigner:usualView>

<celldesigner:briefView>

<celldesigner:innerPosition x="0.0" y="0.0"/>

<celldesigner:boxSize width="80.0" height="60.0"/>

<celldesigner:singleLine width="1.0"/>

<celldesigner:paint color="3fff0000" scheme="Color"/>

</celldesigner:briefView>

<celldesigner:info state="empty" angle="-1.5707963267948966"/>

</celldesigner:speciesAlias>

<celldesigner:speciesAlias id="sa573" species="s476">

<celldesigner:activity>inactive</celldesigner:activity>

<celldesigner:bounds x="1684.5" y="411.25" w="51.0" h="18.0"/>

<celldesigner:font size="12"/>

<celldesigner:view state="usual"/>

<celldesigner:usualView>

<celldesigner:innerPosition x="2247.5" y="771.0"/>

<celldesigner:boxSize width="51.0" height="18.0"/>

<celldesigner:singleLine width="1.0"/>

<celldesigner:paint color="ffccff66" scheme="Color"/>

</celldesigner:usualView>

<celldesigner:briefView>

<celldesigner:innerPosition x="0.0" y="0.0"/>

<celldesigner:boxSize width="80.0" height="60.0"/>

<celldesigner:singleLine width="1.0"/>

<celldesigner:paint color="3fff0000" scheme="Color"/>

</celldesigner:briefView>

<celldesigner:info state="empty" angle="-1.5707963267948966"/>

</celldesigner:speciesAlias>

<celldesigner:speciesAlias id="sa574" species="s491">

<celldesigner:activity>inactive</celldesigner:activity>

<celldesigner:bounds x="1631.5" y="501.75" w="57.0" h="17.0"/>

<celldesigner:font size="12"/>

<celldesigner:view state="usual"/>

<celldesigner:usualView>

<celldesigner:innerPosition x="2409.5" y="754.0"/>

<celldesigner:boxSize width="57.0" height="17.0"/>

<celldesigner:singleLine width="1.0"/>

<celldesigner:paint color="ff006666" scheme="Color"/>

</celldesigner:usualView>

<celldesigner:briefView>

<celldesigner:innerPosition x="0.0" y="0.0"/>

<celldesigner:boxSize width="80.0" height="60.0"/>

<celldesigner:singleLine width="1.0"/>

<celldesigner:paint color="3fff0000" scheme="Color"/>

</celldesigner:briefView>

<celldesigner:info state="empty" angle="-1.5707963267948966"/>

</celldesigner:speciesAlias>

<celldesigner:speciesAlias id="sa578" species="s246">

<celldesigner:activity>inactive</celldesigner:activity>

<celldesigner:bounds x="2068.5" y="857.5" w="78.0" h="16.0"/>

<celldesigner:font size="12"/>

<celldesigner:view state="usual"/>

<celldesigner:usualView>

<celldesigner:innerPosition x="0.0" y="0.0"/>

<celldesigner:boxSize width="78.0" height="16.0"/>

<celldesigner:singleLine width="1.0"/>

<celldesigner:paint color="ff006633" scheme="Color"/>

</celldesigner:usualView>

<celldesigner:briefView>

<celldesigner:innerPosition x="0.0" y="0.0"/>

<celldesigner:boxSize width="80.0" height="60.0"/>

<celldesigner:singleLine width="1.0"/>

<celldesigner:paint color="3fff0000" scheme="Color"/>

</celldesigner:briefView>

<celldesigner:info state="empty" angle="-1.5707963267948966"/>

</celldesigner:speciesAlias>

<celldesigner:speciesAlias id="sa579" species="s247">

<celldesigner:activity>inactive</celldesigner:activity>

<celldesigner:bounds x="2201.5" y="863.5" w="78.0" h="16.0"/>

<celldesigner:font size="12"/>

<celldesigner:view state="usual"/>

<celldesigner:usualView>

<celldesigner:innerPosition x="0.0" y="0.0"/>

<celldesigner:boxSize width="78.0" height="16.0"/>

<celldesigner:singleLine width="1.0"/>

<celldesigner:paint color="ff006633" scheme="Color"/>

</celldesigner:usualView>

<celldesigner:briefView>

<celldesigner:innerPosition x="0.0" y="0.0"/>

<celldesigner:boxSize width="80.0" height="60.0"/>

<celldesigner:singleLine width="1.0"/>

<celldesigner:paint color="3fff0000" scheme="Color"/>

</celldesigner:briefView>

<celldesigner:info state="empty" angle="-1.5707963267948966"/>

</celldesigner:speciesAlias>

<celldesigner:speciesAlias id="sa580" species="s248">

<celldesigner:activity>inactive</celldesigner:activity>

<celldesigner:bounds x="1935.5" y="850.5" w="78.0" h="16.0"/>

<celldesigner:font size="12"/>

<celldesigner:view state="usual"/>

<celldesigner:usualView>

<celldesigner:innerPosition x="0.0" y="0.0"/>

<celldesigner:boxSize width="78.0" height="16.0"/>

<celldesigner:singleLine width="1.0"/>

<celldesigner:paint color="ff006633" scheme="Color"/>

</celldesigner:usualView>

<celldesigner:briefView>

<celldesigner:innerPosition x="0.0" y="0.0"/>

<celldesigner:boxSize width="80.0" height="60.0"/>

<celldesigner:singleLine width="1.0"/>

<celldesigner:paint color="3fff0000" scheme="Color"/>

</celldesigner:briefView>

<celldesigner:info state="empty" angle="-1.5707963267948966"/>

</celldesigner:speciesAlias>

<celldesigner:speciesAlias id="sa581" species="s249">

<celldesigner:activity>inactive</celldesigner:activity>

<celldesigner:bounds x="1936.5" y="922.5" w="78.0" h="16.0"/>

<celldesigner:font size="12"/>

<celldesigner:view state="usual"/>

<celldesigner:usualView>

<celldesigner:innerPosition x="0.0" y="0.0"/>

<celldesigner:boxSize width="78.0" height="16.0"/>

<celldesigner:singleLine width="1.0"/>

<celldesigner:paint color="ff006633" scheme="Color"/>

</celldesigner:usualView>

<celldesigner:briefView>

<celldesigner:innerPosition x="0.0" y="0.0"/>

<celldesigner:boxSize width="80.0" height="60.0"/>

<celldesigner:singleLine width="1.0"/>

<celldesigner:paint color="3fff0000" scheme="Color"/>

</celldesigner:briefView>

<celldesigner:info state="empty" angle="-1.5707963267948966"/>

</celldesigner:speciesAlias>

<celldesigner:speciesAlias id="sa582" species="s389">

<celldesigner:activity>inactive</celldesigner:activity>

<celldesigner:bounds x="2204.5" y="931.5" w="78.0" h="16.0"/>

<celldesigner:font size="12"/>

<celldesigner:view state="usual"/>

<celldesigner:usualView>

<celldesigner:innerPosition x="0.0" y="0.0"/>

<celldesigner:boxSize width="78.0" height="16.0"/>

<celldesigner:singleLine width="1.0"/>

<celldesigner:paint color="ff006633" scheme="Color"/>

</celldesigner:usualView>

<celldesigner:briefView>

<celldesigner:innerPosition x="0.0" y="0.0"/>

<celldesigner:boxSize width="80.0" height="60.0"/>

<celldesigner:singleLine width="1.0"/>

<celldesigner:paint color="3fff0000" scheme="Color"/>

</celldesigner:briefView>

<celldesigner:info state="empty" angle="-1.5707963267948966"/>

</celldesigner:speciesAlias>

<celldesigner:speciesAlias id="sa583" species="s254">

<celldesigner:activity>inactive</celldesigner:activity>

<celldesigner:bounds x="2068.5" y="923.5" w="78.0" h="16.0"/>

<celldesigner:font size="12"/>

<celldesigner:view state="usual"/>

<celldesigner:usualView>

<celldesigner:innerPosition x="0.0" y="0.0"/>

<celldesigner:boxSize width="78.0" height="16.0"/>

<celldesigner:singleLine width="1.0"/>

<celldesigner:paint color="ff006633" scheme="Color"/>

</celldesigner:usualView>

<celldesigner:briefView>

<celldesigner:innerPosition x="0.0" y="0.0"/>

<celldesigner:boxSize width="80.0" height="60.0"/>

<celldesigner:singleLine width="1.0"/>

<celldesigner:paint color="3fff0000" scheme="Color"/>

</celldesigner:briefView>

<celldesigner:info state="empty" angle="-1.5707963267948966"/>

</celldesigner:speciesAlias>

<celldesigner:speciesAlias id="sa584" species="s250">

<celldesigner:activity>inactive</celldesigner:activity>

<celldesigner:bounds x="1938.5" y="986.5" w="78.0" h="16.0"/>

<celldesigner:font size="12"/>

<celldesigner:view state="usual"/>

<celldesigner:usualView>

<celldesigner:innerPosition x="0.0" y="0.0"/>

<celldesigner:boxSize width="78.0" height="16.0"/>

<celldesigner:singleLine width="1.0"/>

<celldesigner:paint color="ff006633" scheme="Color"/>

</celldesigner:usualView>

<celldesigner:briefView>

<celldesigner:innerPosition x="0.0" y="0.0"/>

<celldesigner:boxSize width="80.0" height="60.0"/>

<celldesigner:singleLine width="1.0"/>

<celldesigner:paint color="3fff0000" scheme="Color"/>

</celldesigner:briefView>

<celldesigner:info state="empty" angle="-1.5707963267948966"/>

</celldesigner:speciesAlias>

<celldesigner:speciesAlias id="sa585" species="s447">

<celldesigner:activity>inactive</celldesigner:activity>

<celldesigner:bounds x="2201.5" y="999.5" w="78.0" h="16.0"/>

<celldesigner:font size="12"/>

<celldesigner:view state="usual"/>

<celldesigner:usualView>

<celldesigner:innerPosition x="0.0" y="0.0"/>

<celldesigner:boxSize width="78.0" height="16.0"/>

<celldesigner:singleLine width="1.0"/>

<celldesigner:paint color="ff006633" scheme="Color"/>

</celldesigner:usualView>

<celldesigner:briefView>

<celldesigner:innerPosition x="0.0" y="0.0"/>

<celldesigner:boxSize width="80.0" height="60.0"/>

<celldesigner:singleLine width="1.0"/>

<celldesigner:paint color="3fff0000" scheme="Color"/>

</celldesigner:briefView>

<celldesigner:info state="empty" angle="-1.5707963267948966"/>

</celldesigner:speciesAlias>

<celldesigner:speciesAlias id="sa586" species="s255">

<celldesigner:activity>inactive</celldesigner:activity>

<celldesigner:bounds x="2066.5" y="990.5" w="78.0" h="16.0"/>

<celldesigner:font size="12"/>

<celldesigner:view state="usual"/>

<celldesigner:usualView>

<celldesigner:innerPosition x="0.0" y="0.0"/>

<celldesigner:boxSize width="78.0" height="16.0"/>

<celldesigner:singleLine width="1.0"/>

<celldesigner:paint color="ff006633" scheme="Color"/>

</celldesigner:usualView>

<celldesigner:briefView>

<celldesigner:innerPosition x="0.0" y="0.0"/>

<celldesigner:boxSize width="80.0" height="60.0"/>

<celldesigner:singleLine width="1.0"/>

<celldesigner:paint color="3fff0000" scheme="Color"/>

</celldesigner:briefView>

<celldesigner:info state="empty" angle="-1.5707963267948966"/>

</celldesigner:speciesAlias>

<celldesigner:speciesAlias id="sa587" species="s400">

<celldesigner:activity>inactive</celldesigner:activity>

<celldesigner:bounds x="2195.5" y="1067.5" w="78.0" h="16.0"/>

<celldesigner:font size="12"/>

<celldesigner:view state="usual"/>

<celldesigner:usualView>

<celldesigner:innerPosition x="0.0" y="0.0"/>

<celldesigner:boxSize width="78.0" height="16.0"/>

<celldesigner:singleLine width="1.0"/>

<celldesigner:paint color="ff006633" scheme="Color"/>

</celldesigner:usualView>

<celldesigner:briefView>

<celldesigner:innerPosition x="0.0" y="0.0"/>

<celldesigner:boxSize width="80.0" height="60.0"/>

<celldesigner:singleLine width="1.0"/>

<celldesigner:paint color="3fff0000" scheme="Color"/>

</celldesigner:briefView>

<celldesigner:info state="empty" angle="-1.5707963267948966"/>

</celldesigner:speciesAlias>

<celldesigner:speciesAlias id="sa588" species="s392">

<celldesigner:activity>inactive</celldesigner:activity>

<celldesigner:bounds x="1935.5" y="1065.5" w="78.0" h="16.0"/>

<celldesigner:font size="12"/>

<celldesigner:view state="usual"/>

<celldesigner:usualView>

<celldesigner:innerPosition x="0.0" y="0.0"/>

<celldesigner:boxSize width="78.0" height="16.0"/>

<celldesigner:singleLine width="1.0"/>

<celldesigner:paint color="ff006633" scheme="Color"/>

</celldesigner:usualView>

<celldesigner:briefView>

<celldesigner:innerPosition x="0.0" y="0.0"/>

<celldesigner:boxSize width="80.0" height="60.0"/>

<celldesigner:singleLine width="1.0"/>

<celldesigner:paint color="3fff0000" scheme="Color"/>

</celldesigner:briefView>

<celldesigner:info state="empty" angle="-1.5707963267948966"/>

</celldesigner:speciesAlias>

<celldesigner:speciesAlias id="sa589" species="s395">

<celldesigner:activity>inactive</celldesigner:activity>

<celldesigner:bounds x="2068.5" y="1049.5" w="78.0" h="16.0"/>

<celldesigner:font size="12"/>

<celldesigner:view state="usual"/>

<celldesigner:usualView>

<celldesigner:innerPosition x="0.0" y="0.0"/>

<celldesigner:boxSize width="78.0" height="16.0"/>

<celldesigner:singleLine width="1.0"/>

<celldesigner:paint color="ff006633" scheme="Color"/>

</celldesigner:usualView>

<celldesigner:briefView>

<celldesigner:innerPosition x="0.0" y="0.0"/>

<celldesigner:boxSize width="80.0" height="60.0"/>

<celldesigner:singleLine width="1.0"/>

<celldesigner:paint color="3fff0000" scheme="Color"/>

</celldesigner:briefView>

<celldesigner:info state="empty" angle="-1.5707963267948966"/>

</celldesigner:speciesAlias>

<celldesigner:speciesAlias id="sa591" species="s396">

<celldesigner:activity>inactive</celldesigner:activity>

<celldesigner:bounds x="2067.5" y="1109.5" w="78.0" h="16.0"/>

<celldesigner:font size="12"/>

<celldesigner:view state="usual"/>

<celldesigner:usualView>

<celldesigner:innerPosition x="0.0" y="0.0"/>

<celldesigner:boxSize width="78.0" height="16.0"/>

<celldesigner:singleLine width="1.0"/>

<celldesigner:paint color="ff006633" scheme="Color"/>

</celldesigner:usualView>

<celldesigner:briefView>

<celldesigner:innerPosition x="0.0" y="0.0"/>

<celldesigner:boxSize width="80.0" height="60.0"/>

<celldesigner:singleLine width="1.0"/>

<celldesigner:paint color="3fff0000" scheme="Color"/>

</celldesigner:briefView>

<celldesigner:info state="empty" angle="-1.5707963267948966"/>

</celldesigner:speciesAlias>

<celldesigner:speciesAlias id="sa592" species="s401">

<celldesigner:activity>inactive</celldesigner:activity>

<celldesigner:bounds x="2192.5" y="1119.5" w="78.0" h="16.0"/>

<celldesigner:font size="12"/>

<celldesigner:view state="usual"/>

<celldesigner:usualView>

<celldesigner:innerPosition x="0.0" y="0.0"/>

<celldesigner:boxSize width="78.0" height="16.0"/>

<celldesigner:singleLine width="1.0"/>

<celldesigner:paint color="ff006633" scheme="Color"/>

</celldesigner:usualView>

<celldesigner:briefView>

<celldesigner:innerPosition x="0.0" y="0.0"/>

<celldesigner:boxSize width="80.0" height="60.0"/>

<celldesigner:singleLine width="1.0"/>

<celldesigner:paint color="3fff0000" scheme="Color"/>

</celldesigner:briefView>

<celldesigner:info state="empty" angle="-1.5707963267948966"/>

</celldesigner:speciesAlias>

<celldesigner:speciesAlias id="sa593" species="s397">

<celldesigner:activity>inactive</celldesigner:activity>

<celldesigner:bounds x="2075.5" y="1169.5" w="78.0" h="16.0"/>

<celldesigner:font size="12"/>

<celldesigner:view state="usual"/>

<celldesigner:usualView>

<celldesigner:innerPosition x="0.0" y="0.0"/>

<celldesigner:boxSize width="78.0" height="16.0"/>

<celldesigner:singleLine width="1.0"/>

<celldesigner:paint color="ff006633" scheme="Color"/>

</celldesigner:usualView>

<celldesigner:briefView>

<celldesigner:innerPosition x="0.0" y="0.0"/>

<celldesigner:boxSize width="80.0" height="60.0"/>

<celldesigner:singleLine width="1.0"/>

<celldesigner:paint color="3fff0000" scheme="Color"/>

</celldesigner:briefView>

<celldesigner:info state="empty" angle="-1.5707963267948966"/>

</celldesigner:speciesAlias>

<celldesigner:speciesAlias id="sa594" species="s402">

<celldesigner:activity>inactive</celldesigner:activity>

<celldesigner:bounds x="2195.5" y="1197.5" w="78.0" h="16.0"/>

<celldesigner:font size="12"/>

<celldesigner:view state="usual"/>

<celldesigner:usualView>

<celldesigner:innerPosition x="0.0" y="0.0"/>

<celldesigner:boxSize width="78.0" height="16.0"/>

<celldesigner:singleLine width="1.0"/>

<celldesigner:paint color="ff006633" scheme="Color"/>

</celldesigner:usualView>

<celldesigner:briefView>

<celldesigner:innerPosition x="0.0" y="0.0"/>

<celldesigner:boxSize width="80.0" height="60.0"/>

<celldesigner:singleLine width="1.0"/>

<celldesigner:paint color="3fff0000" scheme="Color"/>

</celldesigner:briefView>

<celldesigner:info state="empty" angle="-1.5707963267948966"/>

</celldesigner:speciesAlias>

<celldesigner:speciesAlias id="sa595" species="s398">

<celldesigner:activity>inactive</celldesigner:activity>

<celldesigner:bounds x="2061.5" y="1239.5" w="78.0" h="16.0"/>

<celldesigner:font size="12"/>

<celldesigner:view state="usual"/>

<celldesigner:usualView>

<celldesigner:innerPosition x="0.0" y="0.0"/>

<celldesigner:boxSize width="78.0" height="16.0"/>

<celldesigner:singleLine width="1.0"/>

<celldesigner:paint color="ff006633" scheme="Color"/>

</celldesigner:usualView>

<celldesigner:briefView>

<celldesigner:innerPosition x="0.0" y="0.0"/>

<celldesigner:boxSize width="80.0" height="60.0"/>

<celldesigner:singleLine width="1.0"/>

<celldesigner:paint color="3fff0000" scheme="Color"/>

</celldesigner:briefView>

<celldesigner:info state="empty" angle="-1.5707963267948966"/>

</celldesigner:speciesAlias>

<celldesigner:speciesAlias id="sa596" species="s399">

<celldesigner:activity>inactive</celldesigner:activity>

<celldesigner:bounds x="2062.5" y="1320.5" w="78.0" h="16.0"/>

<celldesigner:font size="12"/>

<celldesigner:view state="usual"/>

<celldesigner:usualView>

<celldesigner:innerPosition x="0.0" y="0.0"/>

<celldesigner:boxSize width="78.0" height="16.0"/>

<celldesigner:singleLine width="1.0"/>

<celldesigner:paint color="ff006633" scheme="Color"/>

</celldesigner:usualView>

<celldesigner:briefView>

<celldesigner:innerPosition x="0.0" y="0.0"/>

<celldesigner:boxSize width="80.0" height="60.0"/>

<celldesigner:singleLine width="1.0"/>

<celldesigner:paint color="3fff0000" scheme="Color"/>

</celldesigner:briefView>

<celldesigner:info state="empty" angle="-1.5707963267948966"/>

</celldesigner:speciesAlias>

<celldesigner:speciesAlias id="sa597" species="s7">

<celldesigner:activity>inactive</celldesigner:activity>

<celldesigner:bounds x="2210.0" y="1330.0" w="51.0" h="18.0"/>

<celldesigner:font size="12"/>

<celldesigner:view state="usual"/>

<celldesigner:usualView>

<celldesigner:innerPosition x="0.0" y="0.0"/>

<celldesigner:boxSize width="51.0" height="18.0"/>

<celldesigner:singleLine width="1.0"/>

<celldesigner:paint color="ff99ffff" scheme="Color"/>

</celldesigner:usualView>

<celldesigner:briefView>

<celldesigner:innerPosition x="0.0" y="0.0"/>

<celldesigner:boxSize width="80.0" height="60.0"/>

<celldesigner:singleLine width="1.0"/>

<celldesigner:paint color="3fff0000" scheme="Color"/>

</celldesigner:briefView>

<celldesigner:info state="empty" angle="-1.5707963267948966"/>

</celldesigner:speciesAlias>

<celldesigner:speciesAlias id="sa598" species="s404">

<celldesigner:activity>inactive</celldesigner:activity>

<celldesigner:bounds x="2276.5" y="1289.0" w="56.0" h="19.0"/>

<celldesigner:font size="12"/>

<celldesigner:view state="usual"/>

<celldesigner:usualView>

<celldesigner:innerPosition x="0.0" y="0.0"/>

<celldesigner:boxSize width="56.0" height="19.0"/>

<celldesigner:singleLine width="1.0"/>

<celldesigner:paint color="ffccff66" scheme="Color"/>

</celldesigner:usualView>

<celldesigner:briefView>

<celldesigner:innerPosition x="0.0" y="0.0"/>

<celldesigner:boxSize width="80.0" height="60.0"/>

<celldesigner:singleLine width="1.0"/>

<celldesigner:paint color="3fff0000" scheme="Color"/>

</celldesigner:briefView>
<celldesigner:info state="empty" angle="-1.5707963267948966"/>
</celldesigner:speciesAlias>
<celldesigner:speciesAlias id="sa599" species="s1091">
<celldesigner:activity>inactive</celldesigner:activity>
<celldesigner:bounds x="2065.5" y="1390.5" w="78.0" h="16.0"/>
<celldesigner:font size="12"/>
<celldesigner:view state="usual"/>
<celldesigner:usualView>
<celldesigner:innerPosition x="0.0" y="0.0"/>
<celldesigner:boxSize width="78.0" height="16.0"/>
<celldesigner:singleLine width="1.0"/>
<celldesigner:paint color="ff006633" scheme="Color"/>
</celldesigner:usualView>
<celldesigner:briefView>
<celldesigner:innerPosition x="0.0" y="0.0"/>
<celldesigner:boxSize width="80.0" height="60.0"/>
<celldesigner:singleLine width="1.0"/>
<celldesigner:paint color="3fff0000" scheme="Color"/>
</celldesigner:briefView>
<celldesigner:info state="empty" angle="-1.5707963267948966"/>
</celldesigner:speciesAlias>
<celldesigner:speciesAlias id="sa611" species="s241">
<celldesigner:activity>inactive</celldesigner:activity>
<celldesigner:bounds x="2538.5" y="830.0" w="52.0" h="17.0"/>
<celldesigner:font size="12"/>
<celldesigner:view state="usual"/>

<celldesigner:usualView>
<celldesigner:innerPosition x="0.0" y="0.0"/>
<celldesigner:boxSize width="52.0" height="17.0"/>
<celldesigner:singleLine width="1.0"/>
<celldesigner:paint color="ff006633" scheme="Color"/>
</celldesigner:usualView>
<celldesigner:briefView>
<celldesigner:innerPosition x="0.0" y="0.0"/>
<celldesigner:boxSize width="80.0" height="60.0"/>
<celldesigner:singleLine width="1.0"/>
<celldesigner:paint color="3fff0000" scheme="Color"/>
</celldesigner:briefView>
<celldesigner:info state="empty" angle="-1.5707963267948966"/>
</celldesigner:speciesAlias>
<celldesigner:speciesAlias id="sa612" species="s243">
<celldesigner:activity>inactive</celldesigner:activity>
<celldesigner:bounds x="2758.5" y="820.0" w="72.0" h="17.0"/>
<celldesigner:font size="12"/>
<celldesigner:view state="usual"/>
<celldesigner:usualView>
<celldesigner:innerPosition x="0.0" y="0.0"/>
<celldesigner:boxSize width="72.0" height="17.0"/>
<celldesigner:singleLine width="1.0"/>
<celldesigner:paint color="ff006633" scheme="Color"/>
</celldesigner:usualView>
<celldesigner:briefView>
<celldesigner:innerPosition x="0.0" y="0.0"/>

<celldesigner:boxSize width="80.0" height="60.0"/>

<celldesigner:singleLine width="1.0"/>

<celldesigner:paint color="3fff0000" scheme="Color"/>

</celldesigner:briefView>

<celldesigner:info state="empty" angle="-1.5707963267948966"/>

</celldesigner:speciesAlias>

<celldesigner:speciesAlias id="sa613" species="s240">

<celldesigner:activity>inactive</celldesigner:activity>

<celldesigner:bounds x="2627.5" y="739.5" w="74.0" h="18.0"/>

<celldesigner:font size="12"/>

<celldesigner:view state="usual"/>

<celldesigner:usualView>

<celldesigner:innerPosition x="0.0" y="0.0"/>

<celldesigner:boxSize width="74.0" height="18.0"/>

<celldesigner:singleLine width="1.0"/>

<celldesigner:paint color="ff006633" scheme="Color"/>

</celldesigner:usualView>

<celldesigner:briefView>

<celldesigner:innerPosition x="0.0" y="0.0"/>

<celldesigner:boxSize width="80.0" height="60.0"/>

<celldesigner:singleLine width="1.0"/>

<celldesigner:paint color="3fff0000" scheme="Color"/>

</celldesigner:briefView>

<celldesigner:info state="empty" angle="-1.5707963267948966"/>

</celldesigner:speciesAlias>

<celldesigner:speciesAlias id="sa614" species="s474">

<celldesigner:activity>inactive</celldesigner:activity>

<celldesigner:bounds x="2806.0" y="740.0" w="57.0" h="17.0"/>

<celldesigner:font size="12"/>

<celldesigner:view state="usual"/>

<celldesigner:usualView>

<celldesigner:innerPosition x="0.0" y="0.0"/>

<celldesigner:boxSize width="57.0" height="17.0"/>

<celldesigner:singleLine width="1.0"/>

<celldesigner:paint color="ffccff66" scheme="Color"/>

</celldesigner:usualView>

<celldesigner:briefView>

<celldesigner:innerPosition x="0.0" y="0.0"/>

<celldesigner:boxSize width="80.0" height="60.0"/>

<celldesigner:singleLine width="1.0"/>

<celldesigner:paint color="3fff0000" scheme="Color"/>

</celldesigner:briefView>

<celldesigner:info state="empty" angle="-1.5707963267948966"/>

</celldesigner:speciesAlias>

<celldesigner:speciesAlias id="sa615" species="s259">

<celldesigner:activity>inactive</celldesigner:activity>

<celldesigner:bounds x="1609.0" y="621.5" w="82.0" h="17.0"/>

<celldesigner:font size="12"/>

<celldesigner:view state="usual"/>

<celldesigner:usualView>

<celldesigner:innerPosition x="0.0" y="0.0"/>

<celldesigner:boxSize width="82.0" height="17.0"/>

<celldesigner:singleLine width="1.0"/>

<celldesigner:paint color="ffccff66" scheme="Color"/>

</celldesigner:usualView>

<celldesigner:briefView>

<celldesigner:innerPosition x="0.0" y="0.0"/>

<celldesigner:boxSize width="80.0" height="60.0"/>

<celldesigner:singleLine width="1.0"/>

<celldesigner:paint color="3fff0000" scheme="Color"/>

</celldesigner:briefView>

<celldesigner:info state="empty" angle="-1.5707963267948966"/>

</celldesigner:speciesAlias>

<celldesigner:speciesAlias id="sa616" species="s234">

<celldesigner:activity>inactive</celldesigner:activity>

<celldesigner:bounds x="2515.5" y="1130.0" w="58.0" h="17.0"/>

<celldesigner:font size="12"/>

<celldesigner:view state="usual"/>

<celldesigner:usualView>

<celldesigner:innerPosition x="0.0" y="0.0"/>

<celldesigner:boxSize width="58.0" height="17.0"/>

<celldesigner:singleLine width="1.0"/>

<celldesigner:paint color="ffccff66" scheme="Color"/>

</celldesigner:usualView>

<celldesigner:briefView>

<celldesigner:innerPosition x="0.0" y="0.0"/>

<celldesigner:boxSize width="80.0" height="60.0"/>

<celldesigner:singleLine width="1.0"/>

<celldesigner:paint color="3fff0000" scheme="Color"/>

</celldesigner:briefView>

<celldesigner:info state="empty" angle="-1.5707963267948966"/>

</celldesigner:speciesAlias>

<celldesigner:speciesAlias id="sa617" species="s28">

<celldesigner:activity>inactive</celldesigner:activity>

<celldesigner:bounds x="2635.5" y="1130.0" w="58.0" h="17.0"/>

<celldesigner:font size="12"/>

<celldesigner:view state="usual"/>

<celldesigner:usualView>

<celldesigner:innerPosition x="0.0" y="0.0"/>

<celldesigner:boxSize width="58.0" height="17.0"/>

<celldesigner:singleLine width="1.0"/>

<celldesigner:paint color="ffccff66" scheme="Color"/>

</celldesigner:usualView>

<celldesigner:briefView>

<celldesigner:innerPosition x="0.0" y="0.0"/>

<celldesigner:boxSize width="80.0" height="60.0"/>

<celldesigner:singleLine width="1.0"/>

<celldesigner:paint color="3fff0000" scheme="Color"/>

</celldesigner:briefView>

<celldesigner:info state="empty" angle="-1.5707963267948966"/>

</celldesigner:speciesAlias>

<celldesigner:speciesAlias id="sa618" species="s244">

<celldesigner:activity>inactive</celldesigner:activity>

<celldesigner:bounds x="2755.5" y="580.75" w="69.0" h="18.5"/>

<celldesigner:font size="12"/>

<celldesigner:view state="usual"/>

<celldesigner:usualView>

<celldesigner:innerPosition x="0.0" y="0.0"/>

<celldesigner:boxSize width="69.0" height="18.5"/>

<celldesigner:singleLine width="1.0"/>

<celldesigner:paint color="ffccff66" scheme="Color"/>

</celldesigner:usualView>

<celldesigner:briefView>

<celldesigner:innerPosition x="0.0" y="0.0"/>

<celldesigner:boxSize width="80.0" height="60.0"/>

<celldesigner:singleLine width="1.0"/>

<celldesigner:paint color="3fff0000" scheme="Color"/>

</celldesigner:briefView>

<celldesigner:info state="empty" angle="-1.5707963267948966"/>

</celldesigner:speciesAlias>

</celldesigner:listOfSpeciesAliases>

<celldesigner:listOfGroups/>

<celldesigner:listOfProteins/>

<celldesigner:listOfGenes/>

<celldesigner:listOfRNAs/>

<celldesigner:listOfAntisenseRNAs/>

<celldesigner:listOfLayers/>

<celldesigner:listOfBlockDiagrams/>

</celldesigner:extension>

</annotation>

<listOfUnitDefinitions>

<unitDefinition metaid="substance" id="substance" name="substance">

<listOfUnits>

<unit metaid="CDMT00245" kind="mole"/>

</listOfUnits>

</unitDefinition>

<unitDefinition metaid="volume" id="volume" name="volume">

<listOfUnits>

<unit metaid="CDMT00246" kind="litre"/>

</listOfUnits>

</unitDefinition>

<unitDefinition metaid="area" id="area" name="area">

<listOfUnits>

<unit metaid="CDMT00368" kind="metre" exponent="2"/>

</listOfUnits>

</unitDefinition>

<unitDefinition metaid="length" id="length" name="length">

<listOfUnits>

<unit metaid="CDMT00369" kind="metre"/>

</listOfUnits>

</unitDefinition>

<unitDefinition metaid="time" id="time" name="time">

<listOfUnits>

<unit metaid="CDMT00370" kind="second"/>

</listOfUnits>

</unitDefinition>

</listOfUnitDefinitions>

<listOfCompartments>

<compartment metaid="default" id="default" size="1" units="volume"/>

</listOfCompartments>

<listOfSpecies>

<species metaid="s52" id="s52" name="b-D-glc" compartment="default" initialAmount="0">

<annotation>

<celldesigner:extension>

<celldesigner:positionToCompartment>inside</celldesigner:positionToCompartment>

<celldesigner:speciesIdentity>

<celldesigner:class>SIMPLE_MOLECULE</celldesigner:class>

<celldesigner:name>b-D-glc</celldesigner:name>

</celldesigner:speciesIdentity>

</celldesigner:extension>

</annotation>

</species>

<species metaid="s433" id="s433" name="prpp" compartment="default" initialAmount="0">

<annotation>

<celldesigner:extension>

<celldesigner:positionToCompartment>inside</celldesigner:positionToCompartment>

<celldesigner:speciesIdentity>

<celldesigner:class>SIMPLE_MOLECULE</celldesigner:class>

<celldesigner:name>prpp</celldesigner:name>

</celldesigner:speciesIdentity>

</celldesigner:extension>

</annotation>

</species>

<species metaid="s432" id="s432" name="r5p" compartment="default" initialAmount="0">

<annotation>

<celldesigner:extension>

<celldesigner:positionToCompartment>inside</celldesigner:positionToCompartment>

<celldesigner:speciesIdentity>

<celldesigner:class>SIMPLE_MOLECULE</celldesigner:class>

<celldesigner:name>r5p</celldesigner:name>

</celldesigner:speciesIdentity>

</celldesigner:extension>

</annotation>

</species>

<species metaid="s84" id="s84" name="gdpman" compartment="default" initialAmount="0">

<annotation>

<celldesigner:extension>

<celldesigner:positionToCompartment>inside</celldesigner:positionToCompartment>

<celldesigner:speciesIdentity>

<celldesigner:class>SIMPLE_MOLECULE</celldesigner:class>

<celldesigner:name>gdpman</celldesigner:name>

</celldesigner:speciesIdentity>

</celldesigner:extension>

</annotation>

</species>

<species metaid="s83" id="s83" name="man1p" compartment="default" initialAmount="0">

<annotation>

<celldesigner:extension>

<celldesigner:positionToCompartment>inside</celldesigner:positionToCompartment>

<celldesigner:speciesIdentity>

<celldesigner:class>SIMPLE_MOLECULE</celldesigner:class>

<celldesigner:name>man1p</celldesigner:name>

</celldesigner:speciesIdentity>

</celldesigner:extension>

</annotation>

</species>

<species metaid="s82" id="s82" name="man6p" compartment="default" initialAmount="0">

<annotation>

<celldesigner:extension>

<celldesigner:positionToCompartment>inside</celldesigner:positionToCompartment>

<celldesigner:speciesIdentity>

<celldesigner:class>SIMPLE_MOLECULE</celldesigner:class>

<celldesigner:name>man6p</celldesigner:name>

</celldesigner:speciesIdentity>

</celldesigner:extension>

</annotation>

</species>

<species metaid="s57" id="s57" name="b-D-fr6p" compartment="default" initialAmount="0">

<annotation>

<celldesigner:extension>

<celldesigner:positionToCompartment>inside</celldesigner:positionToCompartment>

<celldesigner:speciesIdentity>

<celldesigner:class>SIMPLE_MOLECULE</celldesigner:class>

<celldesigner:name>b-D-fr6p</celldesigner:name>

</celldesigner:speciesIdentity>

</celldesigner:extension>

</annotation>

</species>

<species metaid="s54" id="s54" name="b-D-glc6p" compartment="default" initialAmount="0">

<annotation>

<celldesigner:extension>

<celldesigner:positionToCompartment>inside</celldesigner:positionToCompartment>

<celldesigner:speciesIdentity>

<celldesigner:class>SIMPLE_MOLECULE</celldesigner:class>

<celldesigner:name>b-D-glc6p</celldesigner:name>

</celldesigner:speciesIdentity>

</celldesigner:extension>

</annotation>

</species>

<species metaid="s16" id="s16" name="akg" compartment="default" initialAmount="0">

<annotation>

<celldesigner:extension>

<celldesigner:positionToCompartment>inside</celldesigner:positionToCompartment>

<celldesigner:speciesIdentity>

<celldesigner:class>SIMPLE_MOLECULE</celldesigner:class>

<celldesigner:name>akg</celldesigner:name>

</celldesigner:speciesIdentity>

</celldesigner:extension>

</annotation>

</species>

<species metaid="s85" id="s85" name="mannan" compartment="default" initialAmount="0">

<annotation>

<celldesigner:extension>

<celldesigner:positionToCompartment>inside</celldesigner:positionToCompartment>

<celldesigner:speciesIdentity>

<celldesigner:class>SIMPLE_MOLECULE</celldesigner:class>

<celldesigner:name>mannan</celldesigner:name>

</celldesigner:speciesIdentity>

</celldesigner:extension>

</annotation>

</species>
<species metaid="s58" id="s58" name="b-D-fr16p" compartment="default" initialAmount="0">
<annotation>
<celldesigner:extension>
<celldesigner:positionToCompartment>inside</celldesigner:positionToCompartment>
<celldesigner:speciesIdentity>
<celldesigner:class>SIMPLE_MOLECULE</celldesigner:class>
<celldesigner:name>b-D-fr16p</celldesigner:name>
</celldesigner:speciesIdentity>
</celldesigner:extension>
</annotation>
</species>
<species metaid="s60" id="s60" name="dhap" compartment="default" initialAmount="0">
<annotation>
<celldesigner:extension>
<celldesigner:positionToCompartment>inside</celldesigner:positionToCompartment>
<celldesigner:speciesIdentity>
<celldesigner:class>SIMPLE_MOLECULE</celldesigner:class>
<celldesigner:name>dhap</celldesigner:name>
</celldesigner:speciesIdentity>
</celldesigner:extension>
</annotation>
</species>
<species metaid="s59" id="s59" name="ga3p" compartment="default" initialAmount="0">
<annotation>
<celldesigner:extension>
<celldesigner:positionToCompartment>inside</celldesigner:positionToCompartment>

```
<celldesigner:speciesIdentity>
<celldesigner:class>SIMPLE_MOLECULE</celldesigner:class>
<celldesigner:name>ga3p</celldesigner:name>
</celldesigner:speciesIdentity>
</celldesigner:extension>
</annotation>
</species>
<species metaid="s24" id="s24" name="suc" compartment="default" initialAmount="0">
<annotation>
<celldesigner:extension>
<celldesigner:positionToCompartment>inside</celldesigner:positionToCompartment>
<celldesigner:speciesIdentity>
<celldesigner:class>SIMPLE_MOLECULE</celldesigner:class>
<celldesigner:name>suc</celldesigner:name>
</celldesigner:speciesIdentity>
</celldesigner:extension>
</annotation>
</species>
<species metaid="s63" id="s63" name="bpg" compartment="default" initialAmount="0">
<annotation>
<celldesigner:extension>
<celldesigner:positionToCompartment>inside</celldesigner:positionToCompartment>
<celldesigner:speciesIdentity>
<celldesigner:class>SIMPLE_MOLECULE</celldesigner:class>
<celldesigner:name>bpg</celldesigner:name>
</celldesigner:speciesIdentity>
</celldesigner:extension>
```

</annotation>
</species>
<species metaid="s28" id="s28" name="fum" compartment="default" initialAmount="0">
<annotation>
<celldesigner:extension>
<celldesigner:positionToCompartment>inside</celldesigner:positionToCompartment>
<celldesigner:speciesIdentity>
<celldesigner:class>SIMPLE_MOLECULE</celldesigner:class>
<celldesigner:name>fum</celldesigner:name>
</celldesigner:speciesIdentity>
</celldesigner:extension>
</annotation>
</species>
<species metaid="s64" id="s64" name="3pga" compartment="default" initialAmount="0">
<annotation>
<celldesigner:extension>
<celldesigner:positionToCompartment>inside</celldesigner:positionToCompartment>
<celldesigner:speciesIdentity>
<celldesigner:class>SIMPLE_MOLECULE</celldesigner:class>
<celldesigner:name>3pga</celldesigner:name>
</celldesigner:speciesIdentity>
</celldesigner:extension>
</annotation>
</species>
<species metaid="s33" id="s33" name="mal" compartment="default" initialAmount="0">
<annotation>
<celldesigner:extension>

<celldesigner:positionToCompartment>inside</celldesigner:positionToCompartment>

<celldesigner:speciesIdentity>

<celldesigner:class>SIMPLE_MOLECULE</celldesigner:class>

<celldesigner:name>mal</celldesigner:name>

</celldesigner:speciesIdentity>

</celldesigner:extension>

</annotation>

</species>

<species metaid="s65" id="s65" name="2pga" compartment="default" initialAmount="0">

<annotation>

<celldesigner:extension>

<celldesigner:positionToCompartment>inside</celldesigner:positionToCompartment>

<celldesigner:speciesIdentity>

<celldesigner:class>SIMPLE_MOLECULE</celldesigner:class>

<celldesigner:name>2pga</celldesigner:name>

</celldesigner:speciesIdentity>

</celldesigner:extension>

</annotation>

</species>

<species metaid="s13" id="s13" name="oaa" compartment="default" initialAmount="0">

<annotation>

<celldesigner:extension>

<celldesigner:positionToCompartment>inside</celldesigner:positionToCompartment>

<celldesigner:speciesIdentity>

<celldesigner:class>SIMPLE_MOLECULE</celldesigner:class>

<celldesigner:name>oaa</celldesigner:name>

</celldesigner:speciesIdentity>

</celldesigner:extension>

</annotation>

</species>

<species metaid="s66" id="s66" name="pep" compartment="default" initialAmount="0">

<annotation>

<celldesigner:extension>

<celldesigner:positionToCompartment>inside</celldesigner:positionToCompartment>

<celldesigner:speciesIdentity>

<celldesigner:class>SIMPLE_MOLECULE</celldesigner:class>

<celldesigner:name>pep</celldesigner:name>

</celldesigner:speciesIdentity>

</celldesigner:extension>

</annotation>

</species>

<species metaid="s2" id="s2" name="pyr" compartment="default" initialAmount="0">

<annotation>

<celldesigner:extension>

<celldesigner:positionToCompartment>inside</celldesigner:positionToCompartment>

<celldesigner:speciesIdentity>

<celldesigner:class>SIMPLE_MOLECULE</celldesigner:class>

<celldesigner:name>pyr</celldesigner:name>

</celldesigner:speciesIdentity>

</celldesigner:extension>

</annotation>

</species>

<species metaid="s7" id="s7" name="acoa" compartment="default" initialAmount="0">

<annotation>

<celldesigner:extension>

<celldesigner:positionToCompartment>inside</celldesigner:positionToCompartment>

<celldesigner:speciesIdentity>

<celldesigner:class>SIMPLE_MOLECULE</celldesigner:class>

<celldesigner:name>acoa</celldesigner:name>

</celldesigner:speciesIdentity>

</celldesigner:extension>

</annotation>

</species>

<species metaid="s22" id="s22" name="succoa" compartment="default" initialAmount="0">

<annotation>

<celldesigner:extension>

<celldesigner:positionToCompartment>inside</celldesigner:positionToCompartment>

<celldesigner:speciesIdentity>

<celldesigner:class>SIMPLE_MOLECULE</celldesigner:class>

<celldesigner:name>succoa</celldesigner:name>

</celldesigner:speciesIdentity>

</celldesigner:extension>

</annotation>

</species>

<species metaid="s37" id="s37" name="ac" compartment="default" initialAmount="0">

<annotation>

<celldesigner:extension>

<celldesigner:positionToCompartment>inside</celldesigner:positionToCompartment>

<celldesigner:speciesIdentity>

<celldesigner:class>SIMPLE_MOLECULE</celldesigner:class>

<celldesigner:name>ac</celldesigner:name>

</celldesigner:speciesIdentity>
</celldesigner:extension>
</annotation>
</species>
<species metaid="s15" id="s15" name="isocit" compartment="default" initialAmount="0">
<annotation>
<celldesigner:extension>
<celldesigner:positionToCompartment>inside</celldesigner:positionToCompartment>
<celldesigner:speciesIdentity>
<celldesigner:class>SIMPLE_MOLECULE</celldesigner:class>
<celldesigner:name>isocit</celldesigner:name>
</celldesigner:speciesIdentity>
</celldesigner:extension>
</annotation>
</species>
<species metaid="s14" id="s14" name="cisaco" compartment="default" initialAmount="0">
<annotation>
<celldesigner:extension>
<celldesigner:positionToCompartment>inside</celldesigner:positionToCompartment>
<celldesigner:speciesIdentity>
<celldesigner:class>SIMPLE_MOLECULE</celldesigner:class>
<celldesigner:name>cisaco</celldesigner:name>
</celldesigner:speciesIdentity>
</celldesigner:extension>
</annotation>
</species>
<species metaid="s11" id="s11" name="cit" compartment="default" initialAmount="0">

<annotation>
<celldesigner:extension>
<celldesigner:positionToCompartment>inside</celldesigner:positionToCompartment>
<celldesigner:speciesIdentity>
<celldesigner:class>SIMPLE_MOLECULE</celldesigner:class>
<celldesigner:name>cit</celldesigner:name>
</celldesigner:speciesIdentity>
</celldesigner:extension>
</annotation>
</species>
<species metaid="s1" id="s1" name="g15l6p" compartment="default" initialAmount="0">
<annotation>
<celldesigner:extension>
<celldesigner:positionToCompartment>inside</celldesigner:positionToCompartment>
<celldesigner:speciesIdentity>
<celldesigner:class>SIMPLE_MOLECULE</celldesigner:class>
<celldesigner:name>g15l6p</celldesigner:name>
</celldesigner:speciesIdentity>
</celldesigner:extension>
</annotation>
</species>
<species metaid="s3" id="s3" name="gl6p" compartment="default" initialAmount="0">
<annotation>
<celldesigner:extension>
<celldesigner:positionToCompartment>inside</celldesigner:positionToCompartment>
<celldesigner:speciesIdentity>
<celldesigner:class>SIMPLE_MOLECULE</celldesigner:class>

<celldesigner:name>gl6p</celldesigner:name>

</celldesigner:speciesIdentity>

</celldesigner:extension>

</annotation>

</species>

<species metaid="s5" id="s5" name="ribl5p" compartment="default" initialAmount="0">

<annotation>

<celldesigner:extension>

<celldesigner:positionToCompartment>inside</celldesigner:positionToCompartment>

<celldesigner:speciesIdentity>

<celldesigner:class>SIMPLE_MOLECULE</celldesigner:class>

<celldesigner:name>ribl5p</celldesigner:name>

</celldesigner:speciesIdentity>

</celldesigner:extension>

</annotation>

</species>

<species metaid="s21" id="s21" name="sdhpt7p" compartment="default" initialAmount="0">

<annotation>

<celldesigner:extension>

<celldesigner:positionToCompartment>inside</celldesigner:positionToCompartment>

<celldesigner:speciesIdentity>

<celldesigner:class>SIMPLE_MOLECULE</celldesigner:class>

<celldesigner:name>sdhpt7p</celldesigner:name>

</celldesigner:speciesIdentity>

</celldesigner:extension>

</annotation>

</species>

<species metaid="s23" id="s23" name="erth4p" compartment="default" initialAmount="0">
<annotation>
<celldesigner:extension>
<celldesigner:positionToCompartment>inside</celldesigner:positionToCompartment>
<celldesigner:speciesIdentity>
<celldesigner:class>SIMPLE_MOLECULE</celldesigner:class>
<celldesigner:name>erth4p</celldesigner:name>
</celldesigner:speciesIdentity>
</celldesigner:extension>
</annotation>
</species>
<species metaid="s34" id="s34" name="xyl5p" compartment="default" initialAmount="0">
<annotation>
<celldesigner:extension>
<celldesigner:positionToCompartment>inside</celldesigner:positionToCompartment>
<celldesigner:speciesIdentity>
<celldesigner:class>SIMPLE_MOLECULE</celldesigner:class>
<celldesigner:name>xyl5p</celldesigner:name>
</celldesigner:speciesIdentity>
</celldesigner:extension>
</annotation>
</species>
<species metaid="s35" id="s35" name="f6p" compartment="default" initialAmount="0">
<annotation>
<celldesigner:extension>
<celldesigner:positionToCompartment>inside</celldesigner:positionToCompartment>
<celldesigner:speciesIdentity>

<celldesigner:class>SIMPLE_MOLECULE</celldesigner:class>

<celldesigner:name>f6p</celldesigner:name>

</celldesigner:speciesIdentity>

</celldesigner:extension>

</annotation>

</species>

<species metaid="s36" id="s36" name="f16p" compartment="default" initialAmount="0">

<annotation>

<celldesigner:extension>

<celldesigner:positionToCompartment>inside</celldesigner:positionToCompartment>

<celldesigner:speciesIdentity>

<celldesigner:class>SIMPLE_MOLECULE</celldesigner:class>

<celldesigner:name>f16p</celldesigner:name>

</celldesigner:speciesIdentity>

</celldesigner:extension>

</annotation>

</species>

<species metaid="s61" id="s61" name="g3p" compartment="default" initialAmount="0">

<annotation>

<celldesigner:extension>

<celldesigner:positionToCompartment>inside</celldesigner:positionToCompartment>

<celldesigner:speciesIdentity>

<celldesigner:class>SIMPLE_MOLECULE</celldesigner:class>

<celldesigner:name>g3p</celldesigner:name>

</celldesigner:speciesIdentity>

</celldesigner:extension>

</annotation>

</species>

<species metaid="s434" id="s434" name="mannose" compartment="default" initialAmount="0">

<annotation>

<celldesigner:extension>

<celldesigner:positionToCompartment>inside</celldesigner:positionToCompartment>

<celldesigner:speciesIdentity>

<celldesigner:class>SIMPLE_MOLECULE</celldesigner:class>

<celldesigner:name>mannose</celldesigner:name>

</celldesigner:speciesIdentity>

</celldesigner:extension>

</annotation>

</species>

<species metaid="s435" id="s435" name="fructose" compartment="default" initialAmount="0">

<annotation>

<celldesigner:extension>

<celldesigner:positionToCompartment>inside</celldesigner:positionToCompartment>

<celldesigner:speciesIdentity>

<celldesigner:class>SIMPLE_MOLECULE</celldesigner:class>

<celldesigner:name>fructose</celldesigner:name>

</celldesigner:speciesIdentity>

</celldesigner:extension>

</annotation>

</species>

<species metaid="s456" id="s456" name="3mgcoa" compartment="default" initialAmount="0">

<annotation>

<celldesigner:extension>

<celldesigner:positionToCompartment>inside</celldesigner:positionToCompartment>

<celldesigner:speciesIdentity>

<celldesigner:class>SIMPLE_MOLECULE</celldesigner:class>

<celldesigner:name>3mgcoa</celldesigner:name>

</celldesigner:speciesIdentity>

</celldesigner:extension>

</annotation>

</species>

<species metaid="s459" id="s459" name="acacylcoa" compartment="default" initialAmount="0" charge="0">

<annotation>

<celldesigner:extension>

<celldesigner:positionToCompartment>inside</celldesigner:positionToCompartment>

<celldesigner:speciesIdentity>

<celldesigner:class>SIMPLE_MOLECULE</celldesigner:class>

<celldesigner:name>acacylcoa</celldesigner:name>

</celldesigner:speciesIdentity>

</celldesigner:extension>

</annotation>

</species>

<species metaid="s159" id="s159" name="hmgcoa" compartment="default" initialAmount="0">

<annotation>

<celldesigner:extension>

<celldesigner:positionToCompartment>inside</celldesigner:positionToCompartment>

<celldesigner:speciesIdentity>

<celldesigner:class>SIMPLE_MOLECULE</celldesigner:class>

<celldesigner:name>hmgcoa</celldesigner:name>

</celldesigner:speciesIdentity>

</celldesigner:extension>

</annotation>

</species>

<species metaid="s461" id="s461" name="mev" compartment="default" initialAmount="0" charge="0">

<annotation>

<celldesigner:extension>

<celldesigner:positionToCompartment>inside</celldesigner:positionToCompartment>

<celldesigner:speciesIdentity>

<celldesigner:class>SIMPLE_MOLECULE</celldesigner:class>

<celldesigner:name>mev</celldesigner:name>

</celldesigner:speciesIdentity>

</celldesigner:extension>

</annotation>

</species>

<species metaid="s163" id="s163" name="mev5p" compartment="default" initialAmount="0">

<annotation>

<celldesigner:extension>

<celldesigner:positionToCompartment>inside</celldesigner:positionToCompartment>

<celldesigner:speciesIdentity>

<celldesigner:class>SIMPLE_MOLECULE</celldesigner:class>

<celldesigner:name>mev5p</celldesigner:name>

</celldesigner:speciesIdentity>

</celldesigner:extension>

</annotation>

</species>

<species metaid="s462" id="s462" name="mev5pp" compartment="default" initialAmount="0" charge="0">

<annotation>

<celldesigner:extension>

<celldesigner:positionToCompartment>inside</celldesigner:positionToCompartment>

<celldesigner:speciesIdentity>

<celldesigner:class>SIMPLE_MOLECULE</celldesigner:class>

<celldesigner:name>mev5pp</celldesigner:name>

</celldesigner:speciesIdentity>

</celldesigner:extension>

</annotation>

</species>

<species metaid="s165" id="s165" name="ipdp" compartment="default" initialAmount="0">

<annotation>

<celldesigner:extension>

<celldesigner:positionToCompartment>inside</celldesigner:positionToCompartment>

<celldesigner:speciesIdentity>

<celldesigner:class>SIMPLE_MOLECULE</celldesigner:class>

<celldesigner:name>ipdp</celldesigner:name>

</celldesigner:speciesIdentity>

</celldesigner:extension>

</annotation>

</species>

<species metaid="s476" id="s476" name="formate" compartment="default" initialAmount="0">

<annotation>

<celldesigner:extension>

<celldesigner:positionToCompartment>inside</celldesigner:positionToCompartment>

<celldesigner:speciesIdentity>

<celldesigner:class>SIMPLE_MOLECULE</celldesigner:class>

<celldesigner:name>formate</celldesigner:name>

</celldesigner:speciesIdentity>

</celldesigner:extension>

</annotation>

</species>

<species metaid="s172" id="s172" name="lanostr" compartment="default" initialAmount="0">

<annotation>

<celldesigner:extension>

<celldesigner:positionToCompartment>inside</celldesigner:positionToCompartment>

<celldesigner:speciesIdentity>

<celldesigner:class>SIMPLE_MOLECULE</celldesigner:class>

<celldesigner:name>lanostr</celldesigner:name>

</celldesigner:speciesIdentity>

</celldesigner:extension>

</annotation>

</species>

<species metaid="s173" id="s173" name="44mctr" compartment="default" initialAmount="0">

<annotation>

<celldesigner:extension>

<celldesigner:positionToCompartment>inside</celldesigner:positionToCompartment>

<celldesigner:speciesIdentity>

<celldesigner:class>SIMPLE_MOLECULE</celldesigner:class>

<celldesigner:name>44mctr</celldesigner:name>

</celldesigner:speciesIdentity>

</celldesigner:extension>

</annotation>

</species>

<species metaid="s174" id="s174" name="14dmls" compartment="default" initialAmount="0">

<annotation>

<celldesigner:extension>

<celldesigner:positionToCompartment>inside</celldesigner:positionToCompartment>

<celldesigner:speciesIdentity>

<celldesigner:class>SIMPLE_MOLECULE</celldesigner:class>

<celldesigner:name>14dmls</celldesigner:name>

</celldesigner:speciesIdentity>

</celldesigner:extension>

</annotation>

</species>

<species metaid="s1099" id="s1099" name="zym" compartment="default" initialAmount="0">

<annotation>

<celldesigner:extension>

<celldesigner:positionToCompartment>inside</celldesigner:positionToCompartment>

<celldesigner:speciesIdentity>

<celldesigner:class>SIMPLE_MOLECULE</celldesigner:class>

<celldesigner:name>zym</celldesigner:name>

</celldesigner:speciesIdentity>

</celldesigner:extension>

</annotation>

</species>

<species metaid="s463" id="s463" name="fecostr" compartment="default" initialAmount="0" charge="0">

<annotation>

<celldesigner:extension>

<celldesigner:positionToCompartment>inside</celldesigner:positionToCompartment>

<celldesigner:speciesIdentity>

<celldesigner:class>SIMPLE_MOLECULE</celldesigner:class>

<celldesigner:name>fecostr</celldesigner:name>

</celldesigner:speciesIdentity>

</celldesigner:extension>

</annotation>

</species>

<species metaid="s178" id="s178" name="ergsttr" compartment="default" initialAmount="0">

<annotation>

<celldesigner:extension>

<celldesigner:positionToCompartment>inside</celldesigner:positionToCompartment>

<celldesigner:speciesIdentity>

<celldesigner:class>SIMPLE_MOLECULE</celldesigner:class>

<celldesigner:name>ergsttr</celldesigner:name>

</celldesigner:speciesIdentity>

</celldesigner:extension>

</annotation>

</species>

<species metaid="s464" id="s464" name="ergsttet" compartment="default" initialAmount="0" charge="0">

<annotation>

<celldesigner:extension>

<celldesigner:positionToCompartment>inside</celldesigner:positionToCompartment>

<celldesigner:speciesIdentity>

<celldesigner:class>SIMPLE_MOLECULE</celldesigner:class>

<celldesigner:name>ergsttet</celldesigner:name>

</celldesigner:speciesIdentity>

</celldesigner:extension>

</annotation>

</species>

<species metaid="s465" id="s465" name="ergstr" compartment="default" initialAmount="0" charge="0">

<annotation>

<celldesigner:extension>

<celldesigner:positionToCompartment>inside</celldesigner:positionToCompartment>

<celldesigner:speciesIdentity>

<celldesigner:class>SIMPLE_MOLECULE</celldesigner:class>

<celldesigner:name>ergstr</celldesigner:name>

</celldesigner:speciesIdentity>

</celldesigner:extension>

</annotation>

</species>

<species metaid="s166" id="s166" name="dmpp" compartment="default" initialAmount="0">

<annotation>

<celldesigner:extension>

<celldesigner:positionToCompartment>inside</celldesigner:positionToCompartment>

<celldesigner:speciesIdentity>

<celldesigner:class>SIMPLE_MOLECULE</celldesigner:class>

<celldesigner:name>dmpp</celldesigner:name>

</celldesigner:speciesIdentity>

</celldesigner:extension>

</annotation>

</species>

<species metaid="s167" id="s167" name="geranylpp" compartment="default" initialAmount="0">

<annotation>

<celldesigner:extension>

<celldesigner:positionToCompartment>inside</celldesigner:positionToCompartment>

<celldesigner:speciesIdentity>

<celldesigner:class>SIMPLE_MOLECULE</celldesigner:class>

<celldesigner:name>geranylpp</celldesigner:name>

</celldesigner:speciesIdentity>

</celldesigner:extension>

</annotation>

</species>

<species metaid="s168" id="s168" name="farnasylpp" compartment="default" initialAmount="0">

<annotation>

<celldesigner:extension>

<celldesigner:positionToCompartment>inside</celldesigner:positionToCompartment>

<celldesigner:speciesIdentity>

<celldesigner:class>SIMPLE_MOLECULE</celldesigner:class>

<celldesigner:name>farnasylpp</celldesigner:name>

</celldesigner:speciesIdentity>

</celldesigner:extension>

</annotation>

</species>

<species metaid="s169" id="s169" name="sql" compartment="default" initialAmount="0">

<annotation>

<celldesigner:extension>

<celldesigner:positionToCompartment>inside</celldesigner:positionToCompartment>

<celldesigner:speciesIdentity>

<celldesigner:class>SIMPLE_MOLECULE</celldesigner:class>

<celldesigner:name>sql</celldesigner:name>

</celldesigner:speciesIdentity>

</celldesigner:extension>

</annotation>

</species>

<species metaid="s466" id="s466" name="sql23epx" compartment="default" initialAmount="0" charge="0">

<annotation>

<celldesigner:extension>

<celldesigner:positionToCompartment>inside</celldesigner:positionToCompartment>

<celldesigner:speciesIdentity>

<celldesigner:class>SIMPLE_MOLECULE</celldesigner:class>

<celldesigner:name>sql23epx</celldesigner:name>

</celldesigner:speciesIdentity>

</celldesigner:extension>

</annotation>

</species>

<species metaid="s467" id="s467" name="epistr" compartment="default" initialAmount="0" charge="0">

<annotation>

<celldesigner:extension>

<celldesigner:positionToCompartment>inside</celldesigner:positionToCompartment>

<celldesigner:speciesIdentity>

<celldesigner:class>SIMPLE_MOLECULE</celldesigner:class>

<celldesigner:name>epistr</celldesigner:name>

</celldesigner:speciesIdentity>

</celldesigner:extension>

</annotation>

</species>

<species metaid="s420" id="s420" name="chol" compartment="default" initialAmount="0" charge="0">

<annotation>

<celldesigner:extension>

<celldesigner:positionToCompartment>inside</celldesigner:positionToCompartment>

<celldesigner:speciesIdentity>

<celldesigner:class>SIMPLE_MOLECULE</celldesigner:class>

<celldesigner:name>chol</celldesigner:name>

</celldesigner:speciesIdentity>

</celldesigner:extension>

</annotation>

</species>

<species metaid="s62" id="s62" name="glycerol" compartment="default" initialAmount="0">

<annotation>

<celldesigner:extension>

<celldesigner:positionToCompartment>inside</celldesigner:positionToCompartment>

<celldesigner:speciesIdentity>

<celldesigner:class>SIMPLE_MOLECULE</celldesigner:class>

<celldesigner:name>glycerol</celldesigner:name>

</celldesigner:speciesIdentity>

</celldesigner:extension>

</annotation>

</species>

<species metaid="s120" id="s120" name="fa(pmtacid)" compartment="default" initialAmount="0" charge="0">

<annotation>

<celldesigner:extension>

<celldesigner:positionToCompartment>inside</celldesigner:positionToCompartment>

<celldesigner:speciesIdentity>

<celldesigner:class>SIMPLE_MOLECULE</celldesigner:class>

<celldesigner:name>fa(pmtacid)</celldesigner:name>

</celldesigner:speciesIdentity>

</celldesigner:extension>

</annotation>

</species>

<species metaid="s175" id="s175" name="1ag3p" compartment="default" initialAmount="0" charge="0">

<annotation>

<celldesigner:extension>

<celldesigner:positionToCompartment>inside</celldesigner:positionToCompartment>

<celldesigner:speciesIdentity>

<celldesigner:class>SIMPLE_MOLECULE</celldesigner:class>

<celldesigner:name>1ag3p</celldesigner:name>

</celldesigner:speciesIdentity>

</celldesigner:extension>

</annotation>

</species>

<species metaid="s139" id="s139" name="clpn" compartment="default" initialAmount="0">

<annotation>

<celldesigner:extension>

<celldesigner:positionToCompartment>inside</celldesigner:positionToCompartment>

<celldesigner:speciesIdentity>

<celldesigner:class>SIMPLE_MOLECULE</celldesigner:class>

<celldesigner:name>clpn</celldesigner:name>

</celldesigner:speciesIdentity>

</celldesigner:extension>

</annotation>

</species>

<species metaid="s140" id="s140" name="pg" compartment="default" initialAmount="0">

<annotation>

<celldesigner:extension>

<celldesigner:positionToCompartment>inside</celldesigner:positionToCompartment>

<celldesigner:speciesIdentity>

<celldesigner:class>SIMPLE_MOLECULE</celldesigner:class>

<celldesigner:name>pg</celldesigner:name>

</celldesigner:speciesIdentity>

</celldesigner:extension>

</annotation>

</species>

<species metaid="s137" id="s137" name="ptd1ino" compartment="default" initialAmount="0">

<annotation>

<celldesigner:extension>

<celldesigner:positionToCompartment>inside</celldesigner:positionToCompartment>

<celldesigner:speciesIdentity>

<celldesigner:class>SIMPLE_MOLECULE</celldesigner:class>

<celldesigner:name>ptd1ino</celldesigner:name>

</celldesigner:speciesIdentity>

</celldesigner:extension>

</annotation>

</species>

<species metaid="s176" id="s176" name="pa" compartment="default" initialAmount="0" charge="0">

<annotation>

<celldesigner:extension>

<celldesigner:positionToCompartment>inside</celldesigner:positionToCompartment>

<celldesigner:speciesIdentity>

<celldesigner:class>SIMPLE_MOLECULE</celldesigner:class>

<celldesigner:name>pa</celldesigner:name>

</celldesigner:speciesIdentity>

</celldesigner:extension>

</annotation>

</species>

<species metaid="s135" id="s135" name="cdpdag" compartment="default" initialAmount="0">

<annotation>

<celldesigner:extension>

<celldesigner:positionToCompartment>inside</celldesigner:positionToCompartment>

<celldesigner:speciesIdentity>

<celldesigner:class>SIMPLE_MOLECULE</celldesigner:class>

<celldesigner:name>cdpdag</celldesigner:name>

</celldesigner:speciesIdentity>

</celldesigner:extension>

</annotation>

</species>

<species metaid="s1101" id="s1101" name="acylcoa" compartment="default" initialAmount="0" charge="0">

<annotation>

<celldesigner:extension>

<celldesigner:positionToCompartment>inside</celldesigner:positionToCompartment>

<celldesigner:speciesIdentity>

<celldesigner:class>SIMPLE_MOLECULE</celldesigner:class>

<celldesigner:name>acylcoa</celldesigner:name>

</celldesigner:speciesIdentity>

</celldesigner:extension>

</annotation>

</species>

<species metaid="s138" id="s138" name="mino" compartment="default" initialAmount="0">

<annotation>

<celldesigner:extension>

<celldesigner:positionToCompartment>inside</celldesigner:positionToCompartment>

<celldesigner:speciesIdentity>

<celldesigner:class>SIMPLE_MOLECULE</celldesigner:class>

<celldesigner:name>mino</celldesigner:name>

</celldesigner:speciesIdentity>

</celldesigner:extension>

</annotation>

</species>

<species metaid="s118" id="s118" name="tgr" compartment="default" initialAmount="0" charge="0">

<annotation>

<celldesigner:extension>

<celldesigner:positionToCompartment>inside</celldesigner:positionToCompartment>

<celldesigner:speciesIdentity>

<celldesigner:class>SIMPLE_MOLECULE</celldesigner:class>

<celldesigner:name>tgr</celldesigner:name>

</celldesigner:speciesIdentity>

</celldesigner:extension>

</annotation>

</species>

<species metaid="s177" id="s177" name="12dgr" compartment="default" initialAmount="0" charge="0">

<annotation>

<celldesigner:extension>

<celldesigner:positionToCompartment>inside</celldesigner:positionToCompartment>

<celldesigner:speciesIdentity>

<celldesigner:class>SIMPLE_MOLECULE</celldesigner:class>

<celldesigner:name>12dgr</celldesigner:name>

</celldesigner:speciesIdentity>

</celldesigner:extension>

</annotation>

</species>

<species metaid="s119" id="s119" name="mag" compartment="default" initialAmount="0" charge="0">

<annotation>

<celldesigner:extension>

<celldesigner:positionToCompartment>inside</celldesigner:positionToCompartment>

<celldesigner:speciesIdentity>

<celldesigner:class>SIMPLE_MOLECULE</celldesigner:class>

<celldesigner:name>mag</celldesigner:name>

</celldesigner:speciesIdentity>

</celldesigner:extension>

</annotation>

</species>

<species metaid="s125" id="s125" name="peth" compartment="default" initialAmount="0" charge="0">

<annotation>

<celldesigner:extension>

<celldesigner:positionToCompartment>inside</celldesigner:positionToCompartment>

<celldesigner:speciesIdentity>

<celldesigner:class>SIMPLE_MOLECULE</celldesigner:class>

<celldesigner:name>peth</celldesigner:name>

</celldesigner:speciesIdentity>

</celldesigner:extension>

</annotation>

</species>

<species metaid="s122" id="s122" name="cdpe" compartment="default" initialAmount="0" charge="0">

<annotation>

<celldesigner:extension>

<celldesigner:positionToCompartment>inside</celldesigner:positionToCompartment>

<celldesigner:speciesIdentity>

<celldesigner:class>SIMPLE_MOLECULE</celldesigner:class>

<celldesigner:name>cdpe</celldesigner:name>

</celldesigner:speciesIdentity>

</celldesigner:extension>

</annotation>

</species>

<species metaid="s422" id="s422" name="carboxylate" compartment="default" initialAmount="0" charge="0">

<annotation>

<celldesigner:extension>

<celldesigner:positionToCompartment>inside</celldesigner:positionToCompartment>

<celldesigner:speciesIdentity>

<celldesigner:class>SIMPLE_MOLECULE</celldesigner:class>

<celldesigner:name>carboxylate</celldesigner:name>

</celldesigner:speciesIdentity>

</celldesigner:extension>

</annotation>

</species>

<species metaid="s423" id="s423" name="1g3pe" compartment="default" initialAmount="0" charge="0">

<annotation>

<celldesigner:extension>

<celldesigner:positionToCompartment>inside</celldesigner:positionToCompartment>

<celldesigner:speciesIdentity>

<celldesigner:class>SIMPLE_MOLECULE</celldesigner:class>

<celldesigner:name>1g3pe</celldesigner:name>

</celldesigner:speciesIdentity>

</celldesigner:extension>

</annotation>

</species>

<species metaid="s121" id="s121" name="pe" compartment="default" initialAmount="0" charge="0">

<annotation>

<celldesigner:extension>

<celldesigner:positionToCompartment>inside</celldesigner:positionToCompartment>

<celldesigner:speciesIdentity>

<celldesigner:class>SIMPLE_MOLECULE</celldesigner:class>

<celldesigner:name>pe</celldesigner:name>

</celldesigner:speciesIdentity>

</celldesigner:extension>

</annotation>

</species>

<species metaid="s128" id="s128" name="mmpe" compartment="default" initialAmount="0" charge="0">

<annotation>

<celldesigner:extension>

<celldesigner:positionToCompartment>inside</celldesigner:positionToCompartment>

<celldesigner:speciesIdentity>

<celldesigner:class>SIMPLE_MOLECULE</celldesigner:class>

<celldesigner:name>mmpe</celldesigner:name>

</celldesigner:speciesIdentity>

</celldesigner:extension>

</annotation>

</species>

<species metaid="s424" id="s424" name="g3pe" compartment="default" initialAmount="0" charge="0">

<annotation>

<celldesigner:extension>

<celldesigner:positionToCompartment>inside</celldesigner:positionToCompartment>

<celldesigner:speciesIdentity>

<celldesigner:class>SIMPLE_MOLECULE</celldesigner:class>

<celldesigner:name>g3pe</celldesigner:name>

</celldesigner:speciesIdentity>

</celldesigner:extension>

</annotation>

</species>

<species metaid="s131" id="s131" name="dimpe" compartment="default" initialAmount="0" charge="0">

<annotation>

<celldesigner:extension>

<celldesigner:positionToCompartment>inside</celldesigner:positionToCompartment>

<celldesigner:speciesIdentity>

<celldesigner:class>SIMPLE_MOLECULE</celldesigner:class>

<celldesigner:name>dimpe</celldesigner:name>

</celldesigner:speciesIdentity>

</celldesigner:extension>

</annotation>

</species>

<species metaid="s132" id="s132" name="pc" compartment="default" initialAmount="0" charge="0">

<annotation>

<celldesigner:extension>

<celldesigner:positionToCompartment>inside</celldesigner:positionToCompartment>

<celldesigner:speciesIdentity>

<celldesigner:class>SIMPLE_MOLECULE</celldesigner:class>

<celldesigner:name>pc</celldesigner:name>

</celldesigner:speciesIdentity>

</celldesigner:extension>

</annotation>

</species>

<species metaid="s426" id="s426" name="ethan" compartment="default" initialAmount="0" charge="0">

<annotation>

<celldesigner:extension>

<celldesigner:positionToCompartment>inside</celldesigner:positionToCompartment>

<celldesigner:speciesIdentity>

<celldesigner:class>SIMPLE_MOLECULE</celldesigner:class>

<celldesigner:name>ethan</celldesigner:name>

</celldesigner:speciesIdentity>

</celldesigner:extension>

</annotation>

</species>

<species metaid="s427" id="s427" name="1g3pc" compartment="default" initialAmount="0" charge="0">

<annotation>

<celldesigner:extension>

<celldesigner:positionToCompartment>inside</celldesigner:positionToCompartment>

<celldesigner:speciesIdentity>

<celldesigner:class>SIMPLE_MOLECULE</celldesigner:class>

<celldesigner:name>1g3pc</celldesigner:name>

</celldesigner:speciesIdentity>

</celldesigner:extension>

</annotation>

</species>

<species metaid="s428" id="s428" name="g3pc" compartment="default" initialAmount="0" charge="0">

<annotation>

<celldesigner:extension>

<celldesigner:positionToCompartment>inside</celldesigner:positionToCompartment>

<celldesigner:speciesIdentity>

<celldesigner:class>SIMPLE_MOLECULE</celldesigner:class>

<celldesigner:name>g3pc</celldesigner:name>

</celldesigner:speciesIdentity>

</celldesigner:extension>

</annotation>

</species>

<species metaid="s90" id="s90" name="arg" compartment="default" initialAmount="0">

<annotation>

<celldesigner:extension>

<celldesigner:positionToCompartment>inside</celldesigner:positionToCompartment>

<celldesigner:speciesIdentity>

<celldesigner:class>SIMPLE_MOLECULE</celldesigner:class>

<celldesigner:name>arg</celldesigner:name>

</celldesigner:speciesIdentity>

</celldesigner:extension>

</annotation>

</species>

<species metaid="s478" id="s478" name="orn" compartment="default" initialAmount="0" charge="0">

<annotation>

<celldesigner:extension>

<celldesigner:positionToCompartment>inside</celldesigner:positionToCompartment>

<celldesigner:speciesIdentity>

<celldesigner:class>SIMPLE_MOLECULE</celldesigner:class>

<celldesigner:name>orn</celldesigner:name>

</celldesigner:speciesIdentity>

</celldesigner:extension>

</annotation>

</species>

<species metaid="s479" id="s479" name="urea" compartment="default" initialAmount="0" charge="0">

<annotation>

<celldesigner:extension>

<celldesigner:positionToCompartment>inside</celldesigner:positionToCompartment>

<celldesigner:speciesIdentity>

<celldesigner:class>SIMPLE_MOLECULE</celldesigner:class>

<celldesigner:name>urea</celldesigner:name>

</celldesigner:speciesIdentity>

</celldesigner:extension>

</annotation>

</species>

<species metaid="s471" id="s471" name="met" compartment="default" initialAmount="0" charge="0">

<annotation>

<celldesigner:extension>

<celldesigner:positionToCompartment>inside</celldesigner:positionToCompartment>

<celldesigner:speciesIdentity>

<celldesigner:class>SIMPLE_MOLECULE</celldesigner:class>

<celldesigner:name>met</celldesigner:name>

</celldesigner:speciesIdentity>

</celldesigner:extension>

</annotation>

</species>

<species metaid="s108" id="s108" name="adomet" compartment="default" initialAmount="0">

<annotation>

<celldesigner:extension>

<celldesigner:positionToCompartment>inside</celldesigner:positionToCompartment>

<celldesigner:speciesIdentity>

<celldesigner:class>SIMPLE_MOLECULE</celldesigner:class>

<celldesigner:name>adomet</celldesigner:name>

</celldesigner:speciesIdentity>

</celldesigner:extension>

</annotation>

</species>

<species metaid="s480" id="s480" name="adometam" compartment="default" initialAmount="0" charge="0">

<annotation>

<celldesigner:extension>

<celldesigner:positionToCompartment>inside</celldesigner:positionToCompartment>

<celldesigner:speciesIdentity>

<celldesigner:class>SIMPLE_MOLECULE</celldesigner:class>

<celldesigner:name>adometam</celldesigner:name>

</celldesigner:speciesIdentity>

</celldesigner:extension>

</annotation>

</species>

<species metaid="s481" id="s481" name="put" compartment="default" initialAmount="0" charge="0">

<annotation>

<celldesigner:extension>

<celldesigner:positionToCompartment>inside</celldesigner:positionToCompartment>

<celldesigner:speciesIdentity>

<celldesigner:class>SIMPLE_MOLECULE</celldesigner:class>

<celldesigner:name>put</celldesigner:name>

</celldesigner:speciesIdentity>

</celldesigner:extension>

</annotation>

</species>

<species metaid="s115" id="s115" name="glu" compartment="default" initialAmount="0">

<annotation>

<celldesigner:extension>

<celldesigner:positionToCompartment>inside</celldesigner:positionToCompartment>

<celldesigner:speciesIdentity>

<celldesigner:class>SIMPLE_MOLECULE</celldesigner:class>

<celldesigner:name>glu</celldesigner:name>

</celldesigner:speciesIdentity>

</celldesigner:extension>

</annotation>

</species>

<species metaid="s483" id="s483" name="gsh" compartment="default" initialAmount="0" charge="0">

<annotation>

<celldesigner:extension>

<celldesigner:positionToCompartment>inside</celldesigner:positionToCompartment>

<celldesigner:speciesIdentity>

<celldesigner:class>SIMPLE_MOLECULE</celldesigner:class>

<celldesigner:name>gsh</celldesigner:name>

</celldesigner:speciesIdentity>

</celldesigner:extension>

</annotation>

</species>

<species metaid="s485" id="s485" name="spd" compartment="default" initialAmount="0" charge="0">

<annotation>

<celldesigner:extension>

<celldesigner:positionToCompartment>inside</celldesigner:positionToCompartment>

<celldesigner:speciesIdentity>

<celldesigner:class>SIMPLE_MOLECULE</celldesigner:class>

<celldesigner:name>spd</celldesigner:name>

</celldesigner:speciesIdentity>

</celldesigner:extension>

</annotation>

</species>

<species metaid="s486" id="s486" name="gspd" compartment="default" initialAmount="0" charge="0">

<annotation>

<celldesigner:extension>

<celldesigner:positionToCompartment>inside</celldesigner:positionToCompartment>

<celldesigner:speciesIdentity>

<celldesigner:class>SIMPLE_MOLECULE</celldesigner:class>

<celldesigner:name>gspd</celldesigner:name>

</celldesigner:speciesIdentity>

</celldesigner:extension>

</annotation>

</species>

<species metaid="s475" id="s475" name="cys" compartment="default" initialAmount="0" charge="0">

<annotation>

<celldesigner:extension>

<celldesigner:positionToCompartment>inside</celldesigner:positionToCompartment>

<celldesigner:speciesIdentity>

<celldesigner:class>SIMPLE_MOLECULE</celldesigner:class>

<celldesigner:name>cys</celldesigner:name>

</celldesigner:speciesIdentity>

</celldesigner:extension>

</annotation>

</species>

<species metaid="s473" id="s473" name="gly" compartment="default" initialAmount="0" charge="0">

<annotation>

<celldesigner:extension>

<celldesigner:positionToCompartment>inside</celldesigner:positionToCompartment>

<celldesigner:speciesIdentity>

<celldesigner:class>SIMPLE_MOLECULE</celldesigner:class>

<celldesigner:name>gly</celldesigner:name>

</celldesigner:speciesIdentity>

</celldesigner:extension>

</annotation>

</species>

<species metaid="s306" id="s306" name="ts2" compartment="default" initialAmount="0">

<annotation>

<celldesigner:extension>

<celldesigner:positionToCompartment>inside</celldesigner:positionToCompartment>

<celldesigner:speciesIdentity>

<celldesigner:class>SIMPLE_MOLECULE</celldesigner:class>

<celldesigner:name>ts2</celldesigner:name>

</celldesigner:speciesIdentity>

</celldesigner:extension>

</annotation>

</species>

<species metaid="s68" id="s68" name="mg" compartment="default" initialAmount="0">

<annotation>

<celldesigner:extension>

<celldesigner:positionToCompartment>inside</celldesigner:positionToCompartment>

<celldesigner:speciesIdentity>

<celldesigner:class>SIMPLE_MOLECULE</celldesigner:class>

<celldesigner:name>mg</celldesigner:name>

</celldesigner:speciesIdentity>

</celldesigner:extension>

</annotation>

</species>

<species metaid="s70" id="s70" name="t[sh]2" compartment="default" initialAmount="0">

<annotation>

<celldesigner:extension>
<celldesigner:positionToCompartment>inside</celldesigner:positionToCompartment>
<celldesigner:speciesIdentity>
<celldesigner:class>SIMPLE_MOLECULE</celldesigner:class>
<celldesigner:name>t[sh]2</celldesigner:name>
</celldesigner:speciesIdentity>
</celldesigner:extension>
</annotation>
</species>
<species metaid="s69" id="s69" name="hta" compartment="default" initialAmount="0">
<annotation>
<celldesigner:extension>
<celldesigner:positionToCompartment>inside</celldesigner:positionToCompartment>
<celldesigner:speciesIdentity>
<celldesigner:class>SIMPLE_MOLECULE</celldesigner:class>
<celldesigner:name>hta</celldesigner:name>
</celldesigner:speciesIdentity>
</celldesigner:extension>
</annotation>
</species>
<species metaid="s71" id="s71" name="D-lacgsh" compartment="default" initialAmount="0">
<annotation>
<celldesigner:extension>
<celldesigner:positionToCompartment>inside</celldesigner:positionToCompartment>
<celldesigner:speciesIdentity>
<celldesigner:class>SIMPLE_MOLECULE</celldesigner:class>
<celldesigner:name>D-lacgsh</celldesigner:name>

</celldesigner:speciesIdentity>
</celldesigner:extension>
</annotation>
</species>
<species metaid="s72" id="s72" name="D-lac" compartment="default" initialAmount="0">
<annotation>
<celldesigner:extension>
<celldesigner:positionToCompartment>inside</celldesigner:positionToCompartment>
<celldesigner:speciesIdentity>
<celldesigner:class>SIMPLE_MOLECULE</celldesigner:class>
<celldesigner:name>D-lac</celldesigner:name>
</celldesigner:speciesIdentity>
</celldesigner:extension>
</annotation>
</species>
<species metaid="s487" id="s487" name="L-lactald" compartment="default" initialAmount="0">
<annotation>
<celldesigner:extension>
<celldesigner:positionToCompartment>inside</celldesigner:positionToCompartment>
<celldesigner:speciesIdentity>
<celldesigner:class>SIMPLE_MOLECULE</celldesigner:class>
<celldesigner:name>L-lactald</celldesigner:name>
</celldesigner:speciesIdentity>
</celldesigner:extension>
</annotation>
</species>
<species metaid="s488" id="s488" name="L-lac" compartment="default" initialAmount="0">

<annotation>

<celldesigner:extension>

<celldesigner:positionToCompartment>inside</celldesigner:positionToCompartment>

<celldesigner:speciesIdentity>

<celldesigner:class>SIMPLE_MOLECULE</celldesigner:class>

<celldesigner:name>L-lac</celldesigner:name>

</celldesigner:speciesIdentity>

</celldesigner:extension>

</annotation>

</species>

<species metaid="s1091" id="s1091" name="malcoa" compartment="default" initialAmount="0">

<annotation>

<celldesigner:extension>

<celldesigner:positionToCompartment>inside</celldesigner:positionToCompartment>

<celldesigner:speciesIdentity>

<celldesigner:class>SIMPLE_MOLECULE</celldesigner:class>

<celldesigner:name>malcoa</celldesigner:name>

</celldesigner:speciesIdentity>

</celldesigner:extension>

</annotation>

</species>

<species metaid="s436" id="s436" name="btcoa" compartment="default" initialAmount="0" charge="0">

<annotation>

<celldesigner:extension>

<celldesigner:positionToCompartment>inside</celldesigner:positionToCompartment>

<celldesigner:speciesIdentity>

<celldesigner:class>SIMPLE_MOLECULE</celldesigner:class>

<celldesigner:name>btcoa</celldesigner:name>

</celldesigner:speciesIdentity>

</celldesigner:extension>

</annotation>

</species>

<species metaid="s437" id="s437" name="hxcoa" compartment="default" initialAmount="0" charge="0">

<annotation>

<celldesigner:extension>

<celldesigner:positionToCompartment>inside</celldesigner:positionToCompartment>

<celldesigner:speciesIdentity>

<celldesigner:class>SIMPLE_MOLECULE</celldesigner:class>

<celldesigner:name>hxcoa</celldesigner:name>

</celldesigner:speciesIdentity>

</celldesigner:extension>

</annotation>

</species>

<species metaid="s713" id="s713" name="occoa" compartment="default" initialAmount="0" charge="0">

<annotation>

<celldesigner:extension>

<celldesigner:positionToCompartment>inside</celldesigner:positionToCompartment>

<celldesigner:speciesIdentity>

<celldesigner:class>SIMPLE_MOLECULE</celldesigner:class>

<celldesigner:name>occoa</celldesigner:name>

</celldesigner:speciesIdentity>

</celldesigner:extension>

</annotation>

</species>

<species metaid="s714" id="s714" name="dccoa" compartment="default" initialAmount="0" charge="0">

<annotation>

<celldesigner:extension>

<celldesigner:positionToCompartment>inside</celldesigner:positionToCompartment>

<celldesigner:speciesIdentity>

<celldesigner:class>SIMPLE_MOLECULE</celldesigner:class>

<celldesigner:name>dccoa</celldesigner:name>

</celldesigner:speciesIdentity>

</celldesigner:extension>

</annotation>

</species>

<species metaid="s717" id="s717" name="ddcoa" compartment="default" initialAmount="0" charge="0">

<annotation>

<celldesigner:extension>

<celldesigner:positionToCompartment>inside</celldesigner:positionToCompartment>

<celldesigner:speciesIdentity>

<celldesigner:class>SIMPLE_MOLECULE</celldesigner:class>

<celldesigner:name>ddcoa</celldesigner:name>

</celldesigner:speciesIdentity>

</celldesigner:extension>

</annotation>

</species>

<species metaid="s438" id="s438" name="tdcoa" compartment="default" initialAmount="0" charge="0">

<annotation>

<celldesigner:extension>

<celldesigner:positionToCompartment>inside</celldesigner:positionToCompartment>

<celldesigner:speciesIdentity>

<celldesigner:class>SIMPLE_MOLECULE</celldesigner:class>

<celldesigner:name>tdcoa</celldesigner:name>

</celldesigner:speciesIdentity>

</celldesigner:extension>

</annotation>

</species>

<species metaid="s1098" id="s1098" name="pmtcoa" compartment="default" initialAmount="0">

<annotation>

<celldesigner:extension>

<celldesigner:positionToCompartment>inside</celldesigner:positionToCompartment>

<celldesigner:speciesIdentity>

<celldesigner:class>SIMPLE_MOLECULE</celldesigner:class>

<celldesigner:name>pmtcoa</celldesigner:name>

</celldesigner:speciesIdentity>

</celldesigner:extension>

</annotation>

</species>

<species metaid="s1097" id="s1097" name="strcoa" compartment="default" initialAmount="0">

<annotation>

<celldesigner:extension>

<celldesigner:positionToCompartment>inside</celldesigner:positionToCompartment>

<celldesigner:speciesIdentity>

<celldesigner:class>SIMPLE_MOLECULE</celldesigner:class>

<celldesigner:name>strcoa</celldesigner:name>

</celldesigner:speciesIdentity>

</celldesigner:extension>

</annotation>

</species>

<species metaid="s1102" id="s1102" name="olcoa" compartment="default" initialAmount="0">

<annotation>

<celldesigner:extension>

<celldesigner:positionToCompartment>inside</celldesigner:positionToCompartment>

<celldesigner:speciesIdentity>

<celldesigner:class>SIMPLE_MOLECULE</celldesigner:class>

<celldesigner:name>olcoa</celldesigner:name>

</celldesigner:speciesIdentity>

</celldesigner:extension>

</annotation>

</species>

<species metaid="s439" id="s439" name="lincoa" compartment="default" initialAmount="0" charge="0">

<annotation>

<celldesigner:extension>

<celldesigner:positionToCompartment>inside</celldesigner:positionToCompartment>

<celldesigner:speciesIdentity>

<celldesigner:class>SIMPLE_MOLECULE</celldesigner:class>

<celldesigner:name>lincoa</celldesigner:name>

</celldesigner:speciesIdentity>

</celldesigner:extension>

</annotation>

</species>

<species metaid="s1104" id="s1104" name="a-lincoa" compartment="default" initialAmount="0">

<annotation>

<celldesigner:extension>

<celldesigner:positionToCompartment>inside</celldesigner:positionToCompartment>

<celldesigner:speciesIdentity>

<celldesigner:class>SIMPLE_MOLECULE</celldesigner:class>

<celldesigner:name>a-lincoa</celldesigner:name>

</celldesigner:speciesIdentity>

</celldesigner:extension>

</annotation>

</species>

<species metaid="s730" id="s730" name="eictrcoa" compartment="default" initialAmount="0" charge="0">

<annotation>

<celldesigner:extension>

<celldesigner:positionToCompartment>inside</celldesigner:positionToCompartment>

<celldesigner:speciesIdentity>

<celldesigner:class>SIMPLE_MOLECULE</celldesigner:class>

<celldesigner:name>eictrcoa</celldesigner:name>

</celldesigner:speciesIdentity>

</celldesigner:extension>

</annotation>

</species>

<species metaid="s1103" id="s1103" name="glincoa" compartment="default" initialAmount="0">

<annotation>

<celldesigner:extension>

<celldesigner:positionToCompartment>inside</celldesigner:positionToCompartment>

<celldesigner:speciesIdentity>

<celldesigner:class>SIMPLE_MOLECULE</celldesigner:class>

<celldesigner:name>glincoa</celldesigner:name>

</celldesigner:speciesIdentity>

</celldesigner:extension>

</annotation>

</species>

<species metaid="s1073" id="s1073" name="acdcoa" compartment="default" initialAmount="0">

<annotation>

<celldesigner:extension>

<celldesigner:positionToCompartment>inside</celldesigner:positionToCompartment>

<celldesigner:speciesIdentity>

<celldesigner:class>SIMPLE_MOLECULE</celldesigner:class>

<celldesigner:name>acdcoa</celldesigner:name>

</celldesigner:speciesIdentity>

</celldesigner:extension>

</annotation>

</species>

<species metaid="s96" id="s96" name="stdcoa" compartment="default" initialAmount="0">

<annotation>

<celldesigner:extension>

<celldesigner:positionToCompartment>inside</celldesigner:positionToCompartment>

<celldesigner:speciesIdentity>

<celldesigner:class>SIMPLE_MOLECULE</celldesigner:class>

<celldesigner:name>stdcoa</celldesigner:name>

</celldesigner:speciesIdentity>

</celldesigner:extension>

</annotation>

</species>

<species metaid="s732" id="s732" name="dcopcoa" compartment="default" initialAmount="0" charge="0">

<annotation>

<celldesigner:extension>

<celldesigner:positionToCompartment>inside</celldesigner:positionToCompartment>

<celldesigner:speciesIdentity>

<celldesigner:class>SIMPLE_MOLECULE</celldesigner:class>

<celldesigner:name>dcopcoa</celldesigner:name>

</celldesigner:speciesIdentity>

</celldesigner:extension>

</annotation>

</species>

<species metaid="s733" id="s733" name="dchxcoa" compartment="default" initialAmount="0" charge="0">

<annotation>

<celldesigner:extension>

<celldesigner:positionToCompartment>inside</celldesigner:positionToCompartment>

<celldesigner:speciesIdentity>

<celldesigner:class>SIMPLE_MOLECULE</celldesigner:class>

<celldesigner:name>dchxcoa</celldesigner:name>

</celldesigner:speciesIdentity>

</celldesigner:extension>

</annotation>

</species>

<species metaid="s734" id="s734" name="dcotcoa" compartment="default" initialAmount="0" charge="0">

<annotation>

<celldesigner:extension>

<celldesigner:positionToCompartment>inside</celldesigner:positionToCompartment>

<celldesigner:speciesIdentity>

<celldesigner:class>SIMPLE_MOLECULE</celldesigner:class>

<celldesigner:name>dcotcoa</celldesigner:name>

</celldesigner:speciesIdentity>

</celldesigner:extension>

</annotation>

</species>

<species metaid="s736" id="s736" name="dc4pcoa" compartment="default" initialAmount="0" charge="0">

<annotation>

<celldesigner:extension>

<celldesigner:positionToCompartment>inside</celldesigner:positionToCompartment>

<celldesigner:speciesIdentity>

<celldesigner:class>SIMPLE_MOLECULE</celldesigner:class>

<celldesigner:name>dc4pcoa</celldesigner:name>

</celldesigner:speciesIdentity>

</celldesigner:extension>

</annotation>

</species>

<species metaid="s737" id="s737" name="eicpcoa" compartment="default" initialAmount="0" charge="0">

<annotation>

<celldesigner:extension>

<celldesigner:positionToCompartment>inside</celldesigner:positionToCompartment>

<celldesigner:speciesIdentity>

<celldesigner:class>SIMPLE_MOLECULE</celldesigner:class>

<celldesigner:name>eicpcoa</celldesigner:name>

</celldesigner:speciesIdentity>

</celldesigner:extension>

</annotation>

</species>

<species metaid="s97" id="s97" name="eictcoa" compartment="default" initialAmount="0">

<annotation>

<celldesigner:extension>

<celldesigner:positionToCompartment>inside</celldesigner:positionToCompartment>

<celldesigner:speciesIdentity>

<celldesigner:class>SIMPLE_MOLECULE</celldesigner:class>

<celldesigner:name>eictcoa</celldesigner:name>

</celldesigner:speciesIdentity>

</celldesigner:extension>

</annotation>

</species>

<species metaid="s440" id="s440" name="atp" compartment="default" initialAmount="0" charge="0">

<annotation>

<celldesigner:extension>

<celldesigner:positionToCompartment>inside</celldesigner:positionToCompartment>

<celldesigner:speciesIdentity>

<celldesigner:class>SIMPLE_MOLECULE</celldesigner:class>

<celldesigner:name>atp</celldesigner:name>

</celldesigner:speciesIdentity>

</celldesigner:extension>

</annotation>

</species>

<species metaid="s1027" id="s1027" name="gmp" compartment="default" initialAmount="0">

<annotation>

<celldesigner:extension>

<celldesigner:positionToCompartment>inside</celldesigner:positionToCompartment>

<celldesigner:speciesIdentity>

<celldesigner:class>SIMPLE_MOLECULE</celldesigner:class>

<celldesigner:name>gmp</celldesigner:name>

</celldesigner:speciesIdentity>

</celldesigner:extension>

</annotation>

</species>

<species metaid="s1000" id="s1000" name="amp" compartment="default" initialAmount="0">

<annotation>

<celldesigner:extension>

<celldesigner:positionToCompartment>inside</celldesigner:positionToCompartment>

<celldesigner:speciesIdentity>

<celldesigner:class>SIMPLE_MOLECULE</celldesigner:class>

<celldesigner:name>amp</celldesigner:name>

</celldesigner:speciesIdentity>

</celldesigner:extension>

</annotation>

</species>

<species metaid="s1092" id="s1092" name="adp" compartment="default" initialAmount="0">

<annotation>

<celldesigner:extension>

<celldesigner:positionToCompartment>inside</celldesigner:positionToCompartment>

<celldesigner:speciesIdentity>

<celldesigner:class>SIMPLE_MOLECULE</celldesigner:class>

<celldesigner:name>adp</celldesigner:name>

</celldesigner:speciesIdentity>

</celldesigner:extension>

</annotation>

</species>

<species metaid="s1089" id="s1089" name="gtp" compartment="default" initialAmount="0">

<annotation>

<celldesigner:extension>

<celldesigner:positionToCompartment>inside</celldesigner:positionToCompartment>

<celldesigner:speciesIdentity>

<celldesigner:class>SIMPLE_MOLECULE</celldesigner:class>

<celldesigner:name>gtp</celldesigner:name>

</celldesigner:speciesIdentity>

</celldesigner:extension>

</annotation>

</species>

<species metaid="s1090" id="s1090" name="gdp" compartment="default" initialAmount="0">

<annotation>

<celldesigner:extension>

<celldesigner:positionToCompartment>inside</celldesigner:positionToCompartment>

<celldesigner:speciesIdentity>

<celldesigner:class>SIMPLE_MOLECULE</celldesigner:class>

<celldesigner:name>gdp</celldesigner:name>

</celldesigner:speciesIdentity>

</celldesigner:extension>

</annotation>

</species>

<species metaid="s1038" id="s1038" name="damp" compartment="default" initialAmount="0">

<annotation>

<celldesigner:extension>

<celldesigner:positionToCompartment>inside</celldesigner:positionToCompartment>

<celldesigner:speciesIdentity>

<celldesigner:class>SIMPLE_MOLECULE</celldesigner:class>

<celldesigner:name>damp</celldesigner:name>

</celldesigner:speciesIdentity>

</celldesigner:extension>

</annotation>

</species>

<species metaid="s441" id="s441" name="dadp" compartment="default" initialAmount="0" charge="0">

<annotation>

<celldesigner:extension>

<celldesigner:positionToCompartment>inside</celldesigner:positionToCompartment>

<celldesigner:speciesIdentity>

<celldesigner:class>SIMPLE_MOLECULE</celldesigner:class>

<celldesigner:name>dadp</celldesigner:name>

</celldesigner:speciesIdentity>

</celldesigner:extension>

</annotation>

</species>

<species metaid="s443" id="s443" name="hxan" compartment="default" initialAmount="0">

<annotation>

<celldesigner:extension>

<celldesigner:positionToCompartment>inside</celldesigner:positionToCompartment>

<celldesigner:speciesIdentity>

<celldesigner:class>SIMPLE_MOLECULE</celldesigner:class>

<celldesigner:name>hxan</celldesigner:name>

</celldesigner:speciesIdentity>

</celldesigner:extension>

</annotation>

</species>

<species metaid="s1100" id="s1100" name="imp" compartment="default" initialAmount="0">

<annotation>

<celldesigner:extension>

<celldesigner:positionToCompartment>inside</celldesigner:positionToCompartment>

<celldesigner:speciesIdentity>

<celldesigner:class>SIMPLE_MOLECULE</celldesigner:class>

<celldesigner:name>imp</celldesigner:name>

</celldesigner:speciesIdentity>

</celldesigner:extension>

</annotation>

</species>

<species metaid="s1096" id="s1096" name="dgdp" compartment="default" initialAmount="0">

<annotation>

<celldesigner:extension>

<celldesigner:positionToCompartment>inside</celldesigner:positionToCompartment>

<celldesigner:speciesIdentity>

<celldesigner:class>SIMPLE_MOLECULE</celldesigner:class>

<celldesigner:name>dgdp</celldesigner:name>

</celldesigner:speciesIdentity>

</celldesigner:extension>

</annotation>

</species>

<species metaid="s1095" id="s1095" name="xmp" compartment="default" initialAmount="0">

<annotation>

<celldesigner:extension>

<celldesigner:positionToCompartment>inside</celldesigner:positionToCompartment>

<celldesigner:speciesIdentity>

<celldesigner:class>SIMPLE_MOLECULE</celldesigner:class>

<celldesigner:name>xmp</celldesigner:name>

</celldesigner:speciesIdentity>

</celldesigner:extension>

</annotation>

</species>

<species metaid="s445" id="s445" name="xan" compartment="default" initialAmount="0">

<annotation>

<celldesigner:extension>

<celldesigner:positionToCompartment>inside</celldesigner:positionToCompartment>

<celldesigner:speciesIdentity>

<celldesigner:class>SIMPLE_MOLECULE</celldesigner:class>

<celldesigner:name>xan</celldesigner:name>

</celldesigner:speciesIdentity>

</celldesigner:extension>

</annotation>

</species>

<species metaid="s1037" id="s1037" name="dgmp" compartment="default" initialAmount="0">

<annotation>

<celldesigner:extension>

<celldesigner:positionToCompartment>inside</celldesigner:positionToCompartment>

<celldesigner:speciesIdentity>

<celldesigner:class>SIMPLE_MOLECULE</celldesigner:class>

<celldesigner:name>dgmp</celldesigner:name>
</celldesigner:speciesIdentity>
</celldesigner:extension>
</annotation>
</species>
<species metaid="s979" id="s979" name="gln" compartment="default" initialAmount="0">
<annotation>
<celldesigner:extension>
<celldesigner:positionToCompartment>inside</celldesigner:positionToCompartment>
<celldesigner:speciesIdentity>
<celldesigner:class>SIMPLE_MOLECULE</celldesigner:class>
<celldesigner:name>gln</celldesigner:name>
</celldesigner:speciesIdentity>
</celldesigner:extension>
</annotation>
</species>
<species metaid="s269" id="s269" name="carbp" compartment="default" initialAmount="0">
<annotation>
<celldesigner:extension>
<celldesigner:positionToCompartment>inside</celldesigner:positionToCompartment>
<celldesigner:speciesIdentity>
<celldesigner:class>SIMPLE_MOLECULE</celldesigner:class>
<celldesigner:name>carbp</celldesigner:name>
</celldesigner:speciesIdentity>
</celldesigner:extension>
</annotation>
</species>

<species metaid="s1004" id="s1004" name="asp" compartment="default" initialAmount="0">
<annotation>
<celldesigner:extension>
<celldesigner:positionToCompartment>inside</celldesigner:positionToCompartment>
<celldesigner:speciesIdentity>
<celldesigner:class>SIMPLE_MOLECULE</celldesigner:class>
<celldesigner:name>asp</celldesigner:name>
</celldesigner:speciesIdentity>
</celldesigner:extension>
</annotation>
</species>
<species metaid="s278" id="s278" name="cdp" compartment="default" initialAmount="0">
<annotation>
<celldesigner:extension>
<celldesigner:positionToCompartment>inside</celldesigner:positionToCompartment>
<celldesigner:speciesIdentity>
<celldesigner:class>SIMPLE_MOLECULE</celldesigner:class>
<celldesigner:name>cdp</celldesigner:name>
</celldesigner:speciesIdentity>
</celldesigner:extension>
</annotation>
</species>
<species metaid="s1013" id="s1013" name="ctp" compartment="default" initialAmount="0">
<annotation>
<celldesigner:extension>
<celldesigner:positionToCompartment>inside</celldesigner:positionToCompartment>
<celldesigner:speciesIdentity>

<celldesigner:class>SIMPLE_MOLECULE</celldesigner:class>

<celldesigner:name>ctp</celldesigner:name>

</celldesigner:speciesIdentity>

</celldesigner:extension>

</annotation>

</species>

<species metaid="s1108" id="s1108" name="dcdp" compartment="default" initialAmount="0">

<annotation>

<celldesigner:extension>

<celldesigner:positionToCompartment>inside</celldesigner:positionToCompartment>

<celldesigner:speciesIdentity>

<celldesigner:class>SIMPLE_MOLECULE</celldesigner:class>

<celldesigner:name>dcdp</celldesigner:name>

</celldesigner:speciesIdentity>

</celldesigner:extension>

</annotation>

</species>

<species metaid="s270" id="s270" name="carblasp" compartment="default" initialAmount="0">

<annotation>

<celldesigner:extension>

<celldesigner:positionToCompartment>inside</celldesigner:positionToCompartment>

<celldesigner:speciesIdentity>

<celldesigner:class>SIMPLE_MOLECULE</celldesigner:class>

<celldesigner:name>carblasp</celldesigner:name>

</celldesigner:speciesIdentity>

</celldesigner:extension>

</annotation>

</species>
<species metaid="s1050" id="s1050" name="dcmp" compartment="default" initialAmount="0">
<annotation>
<celldesigner:extension>
<celldesigner:positionToCompartment>inside</celldesigner:positionToCompartment>
<celldesigner:speciesIdentity>
<celldesigner:class>SIMPLE_MOLECULE</celldesigner:class>
<celldesigner:name>dcmp</celldesigner:name>
</celldesigner:speciesIdentity>
</celldesigner:extension>
</annotation>
</species>
<species metaid="s277" id="s277" name="cytidine" compartment="default" initialAmount="0">
<annotation>
<celldesigner:extension>
<celldesigner:positionToCompartment>inside</celldesigner:positionToCompartment>
<celldesigner:speciesIdentity>
<celldesigner:class>SIMPLE_MOLECULE</celldesigner:class>
<celldesigner:name>cytidine</celldesigner:name>
</celldesigner:speciesIdentity>
</celldesigner:extension>
</annotation>
</species>
<species metaid="s1012" id="s1012" name="cmp" compartment="default" initialAmount="0">
<annotation>
<celldesigner:extension>
<celldesigner:positionToCompartment>inside</celldesigner:positionToCompartment>

<celldesigner:speciesIdentity>

<celldesigner:class>SIMPLE_MOLECULE</celldesigner:class>

<celldesigner:name>cmp</celldesigner:name>

</celldesigner:speciesIdentity>

</celldesigner:extension>

</annotation>

</species>

<species metaid="s271" id="s271" name="dhort" compartment="default" initialAmount="0">

<annotation>

<celldesigner:extension>

<celldesigner:positionToCompartment>inside</celldesigner:positionToCompartment>

<celldesigner:speciesIdentity>

<celldesigner:class>SIMPLE_MOLECULE</celldesigner:class>

<celldesigner:name>dhort</celldesigner:name>

</celldesigner:speciesIdentity>

</celldesigner:extension>

</annotation>

</species>

<species metaid="s1051" id="s1051" name="dtmp" compartment="default" initialAmount="0">

<annotation>

<celldesigner:extension>

<celldesigner:positionToCompartment>inside</celldesigner:positionToCompartment>

<celldesigner:speciesIdentity>

<celldesigner:class>SIMPLE_MOLECULE</celldesigner:class>

<celldesigner:name>dtmp</celldesigner:name>

</celldesigner:speciesIdentity>

</celldesigner:extension>

</annotation>
</species>
<species metaid="s276" id="s276" name="uridine" compartment="default" initialAmount="0">
<annotation>
<celldesigner:extension>
<celldesigner:positionToCompartment>inside</celldesigner:positionToCompartment>
<celldesigner:speciesIdentity>
<celldesigner:class>SIMPLE_MOLECULE</celldesigner:class>
<celldesigner:name>uridine</celldesigner:name>
</celldesigner:speciesIdentity>
</celldesigner:extension>
</annotation>
</species>
<species metaid="s272" id="s272" name="ort" compartment="default" initialAmount="0">
<annotation>
<celldesigner:extension>
<celldesigner:positionToCompartment>inside</celldesigner:positionToCompartment>
<celldesigner:speciesIdentity>
<celldesigner:class>SIMPLE_MOLECULE</celldesigner:class>
<celldesigner:name>ort</celldesigner:name>
</celldesigner:speciesIdentity>
</celldesigner:extension>
</annotation>
</species>
<species metaid="s273" id="s273" name="ortdn5p" compartment="default" initialAmount="0">
<annotation>
<celldesigner:extension>

<celldesigner:positionToCompartment>inside</celldesigner:positionToCompartment>

<celldesigner:speciesIdentity>

<celldesigner:class>SIMPLE_MOLECULE</celldesigner:class>

<celldesigner:name>ortdn5p</celldesigner:name>

</celldesigner:speciesIdentity>

</celldesigner:extension>

</annotation>

</species>

<species metaid="s1111" id="s1111" name="dudp" compartment="default" initialAmount="0">

<annotation>

<celldesigner:extension>

<celldesigner:positionToCompartment>inside</celldesigner:positionToCompartment>

<celldesigner:speciesIdentity>

<celldesigner:class>SIMPLE_MOLECULE</celldesigner:class>

<celldesigner:name>dudp</celldesigner:name>

</celldesigner:speciesIdentity>

</celldesigner:extension>

</annotation>

</species>

<species metaid="s1114" id="s1114" name="ump" compartment="default" initialAmount="0">

<annotation>

<celldesigner:extension>

<celldesigner:positionToCompartment>inside</celldesigner:positionToCompartment>

<celldesigner:speciesIdentity>

<celldesigner:class>SIMPLE_MOLECULE</celldesigner:class>

<celldesigner:name>ump</celldesigner:name>

</celldesigner:speciesIdentity>

</celldesigner:extension>

</annotation>

</species>

<species metaid="s1113" id="s1113" name="dutp" compartment="default" initialAmount="0">

<annotation>

<celldesigner:extension>

<celldesigner:positionToCompartment>inside</celldesigner:positionToCompartment>

<celldesigner:speciesIdentity>

<celldesigner:class>SIMPLE_MOLECULE</celldesigner:class>

<celldesigner:name>dutp</celldesigner:name>

</celldesigner:speciesIdentity>

</celldesigner:extension>

</annotation>

</species>

<species metaid="s442" id="s442" name="udp" compartment="default" initialAmount="0" charge="0">

<annotation>

<celldesigner:extension>

<celldesigner:positionToCompartment>inside</celldesigner:positionToCompartment>

<celldesigner:speciesIdentity>

<celldesigner:class>SIMPLE_MOLECULE</celldesigner:class>

<celldesigner:name>udp</celldesigner:name>

</celldesigner:speciesIdentity>

</celldesigner:extension>

</annotation>

</species>

<species metaid="s114" id="s114" name="ala" compartment="default" initialAmount="0">

<annotation>

<celldesigner:extension>

<celldesigner:positionToCompartment>inside</celldesigner:positionToCompartment>

<celldesigner:speciesIdentity>

<celldesigner:class>SIMPLE_MOLECULE</celldesigner:class>

<celldesigner:name>ala</celldesigner:name>

</celldesigner:speciesIdentity>

</celldesigner:extension>

</annotation>

</species>

<species metaid="s472" id="s472" name="thf" compartment="default" initialAmount="0" charge="0">

<annotation>

<celldesigner:extension>

<celldesigner:positionToCompartment>inside</celldesigner:positionToCompartment>

<celldesigner:speciesIdentity>

<celldesigner:class>SIMPLE_MOLECULE</celldesigner:class>

<celldesigner:name>thf</celldesigner:name>

</celldesigner:speciesIdentity>

</celldesigner:extension>

</annotation>

</species>

<species metaid="s444" id="s444" name="ser" compartment="default" initialAmount="0" charge="0">

<annotation>

<celldesigner:extension>

<celldesigner:positionToCompartment>inside</celldesigner:positionToCompartment>

<celldesigner:speciesIdentity>

<celldesigner:class>SIMPLE_MOLECULE</celldesigner:class>

<celldesigner:name>ser</celldesigner:name>

</celldesigner:speciesIdentity>

</celldesigner:extension>

</annotation>

</species>

<species metaid="s446" id="s446" name="pro" compartment="default" initialAmount="0" charge="0">

<annotation>

<celldesigner:extension>

<celldesigner:positionToCompartment>inside</celldesigner:positionToCompartment>

<celldesigner:speciesIdentity>

<celldesigner:class>SIMPLE_MOLECULE</celldesigner:class>

<celldesigner:name>pro</celldesigner:name>

</celldesigner:speciesIdentity>

</celldesigner:extension>

</annotation>

</species>

<species metaid="s490" id="s490" name="thr" compartment="default" initialAmount="0">

<annotation>

<celldesigner:extension>

<celldesigner:positionToCompartment>inside</celldesigner:positionToCompartment>

<celldesigner:speciesIdentity>

<celldesigner:class>SIMPLE_MOLECULE</celldesigner:class>

<celldesigner:name>thr</celldesigner:name>

</celldesigner:speciesIdentity>

</celldesigner:extension>

</annotation>

</species>

<species metaid="s489" id="s489" name="acald" compartment="default" initialAmount="0">

<annotation>

<celldesigner:extension>

<celldesigner:positionToCompartment>inside</celldesigner:positionToCompartment>

<celldesigner:speciesIdentity>

<celldesigner:class>SIMPLE_MOLECULE</celldesigner:class>

<celldesigner:name>acald</celldesigner:name>

</celldesigner:speciesIdentity>

</celldesigner:extension>

</annotation>

</species>

<species metaid="s198" id="s198" name="asn" compartment="default" initialAmount="0">

<annotation>

<celldesigner:extension>

<celldesigner:positionToCompartment>inside</celldesigner:positionToCompartment>

<celldesigner:speciesIdentity>

<celldesigner:class>SIMPLE_MOLECULE</celldesigner:class>

<celldesigner:name>asn</celldesigner:name>

</celldesigner:speciesIdentity>

</celldesigner:extension>

</annotation>

</species>

<species metaid="s231" id="s231" name="trp" compartment="default" initialAmount="0">

<annotation>

<celldesigner:extension>

<celldesigner:positionToCompartment>inside</celldesigner:positionToCompartment>

<celldesigner:speciesIdentity>

<celldesigner:class>SIMPLE_MOLECULE</celldesigner:class>

<celldesigner:name>trp</celldesigner:name>

</celldesigner:speciesIdentity>

</celldesigner:extension>

</annotation>

</species>

<species metaid="s217" id="s217" name="tyr" compartment="default" initialAmount="0">

<annotation>

<celldesigner:extension>

<celldesigner:positionToCompartment>inside</celldesigner:positionToCompartment>

<celldesigner:speciesIdentity>

<celldesigner:class>SIMPLE_MOLECULE</celldesigner:class>

<celldesigner:name>tyr</celldesigner:name>

</celldesigner:speciesIdentity>

</celldesigner:extension>

</annotation>

</species>

<species metaid="s525" id="s525" name="fol" compartment="default" initialAmount="0">

<annotation>

<celldesigner:extension>

<celldesigner:positionToCompartment>inside</celldesigner:positionToCompartment>

<celldesigner:speciesIdentity>

<celldesigner:class>SIMPLE_MOLECULE</celldesigner:class>

<celldesigner:name>fol</celldesigner:name>

</celldesigner:speciesIdentity>

</celldesigner:extension>

</annotation>

</species>

<species metaid="s526" id="s526" name="dhf" compartment="default" initialAmount="0">

<annotation>

<celldesigner:extension>

<celldesigner:positionToCompartment>inside</celldesigner:positionToCompartment>

<celldesigner:speciesIdentity>

<celldesigner:class>SIMPLE_MOLECULE</celldesigner:class>

<celldesigner:name>dhf</celldesigner:name>

</celldesigner:speciesIdentity>

</celldesigner:extension>

</annotation>

</species>

<species metaid="s527" id="s527" name="dhpt" compartment="default" initialAmount="0">

<annotation>

<celldesigner:extension>

<celldesigner:positionToCompartment>inside</celldesigner:positionToCompartment>

<celldesigner:speciesIdentity>

<celldesigner:class>SIMPLE_MOLECULE</celldesigner:class>

<celldesigner:name>dhpt</celldesigner:name>

</celldesigner:speciesIdentity>

</celldesigner:extension>

</annotation>

</species>

<species metaid="s528" id="s528" name="thfglu" compartment="default" initialAmount="0">

<annotation>

<celldesigner:extension>

<celldesigner:positionToCompartment>inside</celldesigner:positionToCompartment>

<celldesigner:speciesIdentity>

<celldesigner:class>SIMPLE_MOLECULE</celldesigner:class>

<celldesigner:name>thfglu</celldesigner:name>

</celldesigner:speciesIdentity>

</celldesigner:extension>

</annotation>

</species>

<species metaid="s834" id="s834" name="thfpolyglu" compartment="default" initialAmount="0">

<annotation>

<celldesigner:extension>

<celldesigner:positionToCompartment>inside</celldesigner:positionToCompartment>

<celldesigner:speciesIdentity>

<celldesigner:class>SIMPLE_MOLECULE</celldesigner:class>

<celldesigner:name>thfpolyglu</celldesigner:name>

</celldesigner:speciesIdentity>

</celldesigner:extension>

</annotation>

</species>

<species metaid="s215" id="s215" name="phe" compartment="default" initialAmount="0">

<annotation>

<celldesigner:extension>

<celldesigner:positionToCompartment>inside</celldesigner:positionToCompartment>

<celldesigner:speciesIdentity>

<celldesigner:class>SIMPLE_MOLECULE</celldesigner:class>

<celldesigner:name>phe</celldesigner:name>

</celldesigner:speciesIdentity>

</celldesigner:extension>

</annotation>
</species>
<species metaid="s835" id="s835" name="3phpyr" compartment="default" initialAmount="0">
<annotation>
<celldesigner:extension>
<celldesigner:positionToCompartment>inside</celldesigner:positionToCompartment>
<celldesigner:speciesIdentity>
<celldesigner:class>SIMPLE_MOLECULE</celldesigner:class>
<celldesigner:name>3phpyr</celldesigner:name>
</celldesigner:speciesIdentity>
</celldesigner:extension>
</annotation>
</species>
<species metaid="s836" id="s836" name="3pser" compartment="default" initialAmount="0">
<annotation>
<celldesigner:extension>
<celldesigner:positionToCompartment>inside</celldesigner:positionToCompartment>
<celldesigner:speciesIdentity>
<celldesigner:class>SIMPLE_MOLECULE</celldesigner:class>
<celldesigner:name>3pser</celldesigner:name>
</celldesigner:speciesIdentity>
</celldesigner:extension>
</annotation>
</species>
<species metaid="s837" id="s837" name="oacser" compartment="default" initialAmount="0">
<annotation>
<celldesigner:extension>

<celldesigner:positionToCompartment>inside</celldesigner:positionToCompartment>

<celldesigner:speciesIdentity>

<celldesigner:class>SIMPLE_MOLECULE</celldesigner:class>

<celldesigner:name>oacser</celldesigner:name>

</celldesigner:speciesIdentity>

</celldesigner:extension>

</annotation>

</species>

<species metaid="s840" id="s840" name="aspsa" compartment="default" initialAmount="0">

<annotation>

<celldesigner:extension>

<celldesigner:positionToCompartment>inside</celldesigner:positionToCompartment>

<celldesigner:speciesIdentity>

<celldesigner:class>SIMPLE_MOLECULE</celldesigner:class>

<celldesigner:name>aspsa</celldesigner:name>

</celldesigner:speciesIdentity>

</celldesigner:extension>

</annotation>

</species>

<species metaid="s841" id="s841" name="hser" compartment="default" initialAmount="0">

<annotation>

<celldesigner:extension>

<celldesigner:positionToCompartment>inside</celldesigner:positionToCompartment>

<celldesigner:speciesIdentity>

<celldesigner:class>SIMPLE_MOLECULE</celldesigner:class>

<celldesigner:name>hser</celldesigner:name>

</celldesigner:speciesIdentity>

</celldesigner:extension>

</annotation>

</species>

<species metaid="s842" id="s842" name="phser" compartment="default" initialAmount="0">

<annotation>

<celldesigner:extension>

<celldesigner:positionToCompartment>inside</celldesigner:positionToCompartment>

<celldesigner:speciesIdentity>

<celldesigner:class>SIMPLE_MOLECULE</celldesigner:class>

<celldesigner:name>phser</celldesigner:name>

</celldesigner:speciesIdentity>

</celldesigner:extension>

</annotation>

</species>

<species metaid="s843" id="s843" name="1pl5c" compartment="default" initialAmount="0">

<annotation>

<celldesigner:extension>

<celldesigner:positionToCompartment>inside</celldesigner:positionToCompartment>

<celldesigner:speciesIdentity>

<celldesigner:class>SIMPLE_MOLECULE</celldesigner:class>

<celldesigner:name>1pl5c</celldesigner:name>

</celldesigner:speciesIdentity>

</celldesigner:extension>

</annotation>

</species>

<species metaid="s844" id="s844" name="cysthion" compartment="default" initialAmount="0">

<annotation>

<celldesigner:extension>

<celldesigner:positionToCompartment>inside</celldesigner:positionToCompartment>

<celldesigner:speciesIdentity>

<celldesigner:class>SIMPLE_MOLECULE</celldesigner:class>

<celldesigner:name>cysthion</celldesigner:name>

</celldesigner:speciesIdentity>

</celldesigner:extension>

</annotation>

</species>

<species metaid="s845" id="s845" name="hcys" compartment="default" initialAmount="0">

<annotation>

<celldesigner:extension>

<celldesigner:positionToCompartment>inside</celldesigner:positionToCompartment>

<celldesigner:speciesIdentity>

<celldesigner:class>SIMPLE_MOLECULE</celldesigner:class>

<celldesigner:name>hcys</celldesigner:name>

</celldesigner:speciesIdentity>

</celldesigner:extension>

</annotation>

</species>

<species metaid="s846" id="s846" name="phepyr" compartment="default" initialAmount="0">

<annotation>

<celldesigner:extension>

<celldesigner:positionToCompartment>inside</celldesigner:positionToCompartment>

<celldesigner:speciesIdentity>

<celldesigner:class>SIMPLE_MOLECULE</celldesigner:class>

<celldesigner:name>phepyr</celldesigner:name>

</celldesigner:speciesIdentity>
</celldesigner:extension>
</annotation>
</species>
<species metaid="s847" id="s847" name="34hpp" compartment="default" initialAmount="0">
<annotation>
<celldesigner:extension>
<celldesigner:positionToCompartment>inside</celldesigner:positionToCompartment>
<celldesigner:speciesIdentity>
<celldesigner:class>SIMPLE_MOLECULE</celldesigner:class>
<celldesigner:name>34hpp</celldesigner:name>
</celldesigner:speciesIdentity>
</celldesigner:extension>
</annotation>
</species>
<species metaid="s848" id="s848" name="4m2opent" compartment="default" initialAmount="0">
<annotation>
<celldesigner:extension>
<celldesigner:positionToCompartment>inside</celldesigner:positionToCompartment>
<celldesigner:speciesIdentity>
<celldesigner:class>SIMPLE_MOLECULE</celldesigner:class>
<celldesigner:name>4m2opent</celldesigner:name>
</celldesigner:speciesIdentity>
</celldesigner:extension>
</annotation>
</species>
<species metaid="s849" id="s849" name="leu" compartment="default" initialAmount="0">

<annotation>

<celldesigner:extension>

<celldesigner:positionToCompartment>inside</celldesigner:positionToCompartment>

<celldesigner:speciesIdentity>

<celldesigner:class>SIMPLE_MOLECULE</celldesigner:class>

<celldesigner:name>leu</celldesigner:name>

</celldesigner:speciesIdentity>

</celldesigner:extension>

</annotation>

</species>

<species metaid="s850" id="s850" name="2oivlrt" compartment="default" initialAmount="0">

<annotation>

<celldesigner:extension>

<celldesigner:positionToCompartment>inside</celldesigner:positionToCompartment>

<celldesigner:speciesIdentity>

<celldesigner:class>SIMPLE_MOLECULE</celldesigner:class>

<celldesigner:name>2oivlrt</celldesigner:name>

</celldesigner:speciesIdentity>

</celldesigner:extension>

</annotation>

</species>

<species metaid="s851" id="s851" name="val" compartment="default" initialAmount="0">

<annotation>

<celldesigner:extension>

<celldesigner:positionToCompartment>inside</celldesigner:positionToCompartment>

<celldesigner:speciesIdentity>

<celldesigner:class>SIMPLE_MOLECULE</celldesigner:class>

<celldesigner:name>val</celldesigner:name>

</celldesigner:speciesIdentity>

</celldesigner:extension>

</annotation>

</species>

<species metaid="s852" id="s852" name="3m2opent" compartment="default" initialAmount="0">

<annotation>

<celldesigner:extension>

<celldesigner:positionToCompartment>inside</celldesigner:positionToCompartment>

<celldesigner:speciesIdentity>

<celldesigner:class>SIMPLE_MOLECULE</celldesigner:class>

<celldesigner:name>3m2opent</celldesigner:name>

</celldesigner:speciesIdentity>

</celldesigner:extension>

</annotation>

</species>

<species metaid="s853" id="s853" name="ile" compartment="default" initialAmount="0">

<annotation>

<celldesigner:extension>

<celldesigner:positionToCompartment>inside</celldesigner:positionToCompartment>

<celldesigner:speciesIdentity>

<celldesigner:class>SIMPLE_MOLECULE</celldesigner:class>

<celldesigner:name>ile</celldesigner:name>

</celldesigner:speciesIdentity>

</celldesigner:extension>

</annotation>

</species>

<species metaid="s256" id="s256" name="nflk" compartment="default" initialAmount="0" charge="0">

<annotation>

<celldesigner:extension>

<celldesigner:positionToCompartment>inside</celldesigner:positionToCompartment>

<celldesigner:speciesIdentity>

<celldesigner:class>SIMPLE_MOLECULE</celldesigner:class>

<celldesigner:name>nflk</celldesigner:name>

</celldesigner:speciesIdentity>

</celldesigner:extension>

</annotation>

</species>

<species metaid="s257" id="s257" name="l-kyn" compartment="default" initialAmount="0" charge="0">

<annotation>

<celldesigner:extension>

<celldesigner:positionToCompartment>inside</celldesigner:positionToCompartment>

<celldesigner:speciesIdentity>

<celldesigner:class>SIMPLE_MOLECULE</celldesigner:class>

<celldesigner:name>l-kyn</celldesigner:name>

</celldesigner:speciesIdentity>

</celldesigner:extension>

</annotation>

</species>

<species metaid="s491" id="s491" name="anthra" compartment="default" initialAmount="0">

<annotation>

<celldesigner:extension>

<celldesigner:positionToCompartment>inside</celldesigner:positionToCompartment>

<celldesigner:speciesIdentity>

<celldesigner:class>SIMPLE_MOLECULE</celldesigner:class>

<celldesigner:name>anthra</celldesigner:name>

</celldesigner:speciesIdentity>

</celldesigner:extension>

</annotation>

</species>

<species metaid="s246" id="s246" name="3mob" compartment="default" initialAmount="0" charge="0">

<annotation>

<celldesigner:extension>

<celldesigner:positionToCompartment>inside</celldesigner:positionToCompartment>

<celldesigner:speciesIdentity>

<celldesigner:class>SIMPLE_MOLECULE</celldesigner:class>

<celldesigner:name>3mob</celldesigner:name>

</celldesigner:speciesIdentity>

</celldesigner:extension>

</annotation>

</species>

<species metaid="s247" id="s247" name="3mop" compartment="default" initialAmount="0">

<annotation>

<celldesigner:extension>

<celldesigner:positionToCompartment>inside</celldesigner:positionToCompartment>

<celldesigner:speciesIdentity>

<celldesigner:class>SIMPLE_MOLECULE</celldesigner:class>

<celldesigner:name>3mop</celldesigner:name>

</celldesigner:speciesIdentity>

</celldesigner:extension>

</annotation>

</species>

<species metaid="s248" id="s248" name="4mop" compartment="default" initialAmount="0" charge="0">

<annotation>

<celldesigner:extension>

<celldesigner:positionToCompartment>inside</celldesigner:positionToCompartment>

<celldesigner:speciesIdentity>

<celldesigner:class>SIMPLE_MOLECULE</celldesigner:class>

<celldesigner:name>4mop</celldesigner:name>

</celldesigner:speciesIdentity>

</celldesigner:extension>

</annotation>

</species>

<species metaid="s249" id="s249" name="3mbutdhla" compartment="default" initialAmount="0" charge="0">

<annotation>

<celldesigner:extension>

<celldesigner:positionToCompartment>inside</celldesigner:positionToCompartment>

<celldesigner:speciesIdentity>

<celldesigner:class>SIMPLE_MOLECULE</celldesigner:class>

<celldesigner:name>3mbutdhla</celldesigner:name>

</celldesigner:speciesIdentity>

</celldesigner:extension>

</annotation>

</species>

<species metaid="s389" id="s389" name="2mbutdhla" compartment="default" initialAmount="0">

<annotation>

<celldesigner:extension>

<celldesigner:positionToCompartment>inside</celldesigner:positionToCompartment>

<celldesigner:speciesIdentity>

<celldesigner:class>SIMPLE_MOLECULE</celldesigner:class>

<celldesigner:name>2mbutdhla</celldesigner:name>

</celldesigner:speciesIdentity>

</celldesigner:extension>

</annotation>

</species>

<species metaid="s254" id="s254" name="2mpdhla" compartment="default" initialAmount="0" charge="0">

<annotation>

<celldesigner:extension>

<celldesigner:positionToCompartment>inside</celldesigner:positionToCompartment>

<celldesigner:speciesIdentity>

<celldesigner:class>SIMPLE_MOLECULE</celldesigner:class>

<celldesigner:name>2mpdhla</celldesigner:name>

</celldesigner:speciesIdentity>

</celldesigner:extension>

</annotation>

</species>

<species metaid="s250" id="s250" name="ivcoa" compartment="default" initialAmount="0" charge="0">

<annotation>

<celldesigner:extension>

<celldesigner:positionToCompartment>inside</celldesigner:positionToCompartment>

<celldesigner:speciesIdentity>

<celldesigner:class>SIMPLE_MOLECULE</celldesigner:class>

<celldesigner:name>ivcoa</celldesigner:name>

</celldesigner:speciesIdentity>

</celldesigner:extension>

</annotation>

</species>

<species metaid="s447" id="s447" name="2mbcoa" compartment="default" initialAmount="0" charge="0">

<annotation>

<celldesigner:extension>

<celldesigner:positionToCompartment>inside</celldesigner:positionToCompartment>

<celldesigner:speciesIdentity>

<celldesigner:class>SIMPLE_MOLECULE</celldesigner:class>

<celldesigner:name>2mbcoa</celldesigner:name>

</celldesigner:speciesIdentity>

</celldesigner:extension>

</annotation>

</species>

<species metaid="s255" id="s255" name="ibcoa" compartment="default" initialAmount="0" charge="0">

<annotation>

<celldesigner:extension>

<celldesigner:positionToCompartment>inside</celldesigner:positionToCompartment>

<celldesigner:speciesIdentity>

<celldesigner:class>SIMPLE_MOLECULE</celldesigner:class>

<celldesigner:name>ibcoa</celldesigner:name>

</celldesigner:speciesIdentity>

</celldesigner:extension>

</annotation>

</species>

<species metaid="s400" id="s400" name="2mbecoa" compartment="default" initialAmount="0">

<annotation>

<celldesigner:extension>

<celldesigner:positionToCompartment>inside</celldesigner:positionToCompartment>

<celldesigner:speciesIdentity>

<celldesigner:class>SIMPLE_MOLECULE</celldesigner:class>

<celldesigner:name>2mbecoa</celldesigner:name>

</celldesigner:speciesIdentity>

</celldesigner:extension>

</annotation>

</species>

<species metaid="s392" id="s392" name="3mb2coa" compartment="default" initialAmount="0" charge="0">

<annotation>

<celldesigner:extension>

<celldesigner:positionToCompartment>inside</celldesigner:positionToCompartment>

<celldesigner:speciesIdentity>

<celldesigner:class>SIMPLE_MOLECULE</celldesigner:class>

<celldesigner:name>3mb2coa</celldesigner:name>

</celldesigner:speciesIdentity>

</celldesigner:extension>

</annotation>

</species>

<species metaid="s395" id="s395" name="macoa" compartment="default" initialAmount="0" charge="0">

<annotation>

<celldesigner:extension>

<celldesigner:positionToCompartment>inside</celldesigner:positionToCompartment>

<celldesigner:speciesIdentity>

<celldesigner:class>SIMPLE_MOLECULE</celldesigner:class>

<celldesigner:name>macoa</celldesigner:name>

</celldesigner:speciesIdentity>

</celldesigner:extension>

</annotation>

</species>

<species metaid="s396" id="s396" name="3hibcoa" compartment="default" initialAmount="0" charge="0">

<annotation>

<celldesigner:extension>

<celldesigner:positionToCompartment>inside</celldesigner:positionToCompartment>

<celldesigner:speciesIdentity>

<celldesigner:class>SIMPLE_MOLECULE</celldesigner:class>

<celldesigner:name>3hibcoa</celldesigner:name>

</celldesigner:speciesIdentity>

</celldesigner:extension>

</annotation>

</species>

<species metaid="s401" id="s401" name="3hmbcoa" compartment="default" initialAmount="0">

<annotation>

<celldesigner:extension>

<celldesigner:positionToCompartment>inside</celldesigner:positionToCompartment>

<celldesigner:speciesIdentity>

<celldesigner:class>SIMPLE_MOLECULE</celldesigner:class>

<celldesigner:name>3hmbcoa</celldesigner:name>

</celldesigner:speciesIdentity>

</celldesigner:extension>

</annotation>

</species>

<species metaid="s397" id="s397" name="3hib" compartment="default" initialAmount="0" charge="0">

<annotation>

<celldesigner:extension>

<celldesigner:positionToCompartment>inside</celldesigner:positionToCompartment>

<celldesigner:speciesIdentity>

<celldesigner:class>SIMPLE_MOLECULE</celldesigner:class>

<celldesigner:name>3hib</celldesigner:name>

</celldesigner:speciesIdentity>

</celldesigner:extension>

</annotation>

</species>

<species metaid="s402" id="s402" name="2maacoa" compartment="default" initialAmount="0">

<annotation>

<celldesigner:extension>

<celldesigner:positionToCompartment>inside</celldesigner:positionToCompartment>

<celldesigner:speciesIdentity>

<celldesigner:class>SIMPLE_MOLECULE</celldesigner:class>

<celldesigner:name>2maacoa</celldesigner:name>

</celldesigner:speciesIdentity>

</celldesigner:extension>

</annotation>

</species>

<species metaid="s398" id="s398" name="mmsald" compartment="default" initialAmount="0" charge="0">

<annotation>
<celldesigner:extension>
<celldesigner:positionToCompartment>inside</celldesigner:positionToCompartment>
<celldesigner:speciesIdentity>
<celldesigner:class>SIMPLE_MOLECULE</celldesigner:class>
<celldesigner:name>mmsald</celldesigner:name>
</celldesigner:speciesIdentity>
</celldesigner:extension>
</annotation>
</species>
<species metaid="s399" id="s399" name="mmalonat" compartment="default" initialAmount="0" charge="0">
<annotation>
<celldesigner:extension>
<celldesigner:positionToCompartment>inside</celldesigner:positionToCompartment>
<celldesigner:speciesIdentity>
<celldesigner:class>SIMPLE_MOLECULE</celldesigner:class>
<celldesigner:name>mmalonat</celldesigner:name>
</celldesigner:speciesIdentity>
</celldesigner:extension>
</annotation>
</species>
<species metaid="s404" id="s404" name="ppcoa" compartment="default" initialAmount="0">
<annotation>
<celldesigner:extension>
<celldesigner:positionToCompartment>inside</celldesigner:positionToCompartment>
<celldesigner:speciesIdentity>
<celldesigner:class>SIMPLE_MOLECULE</celldesigner:class>

<celldesigner:name>ppcoa</celldesigner:name>

</celldesigner:speciesIdentity>

</celldesigner:extension>

</annotation>

</species>

<species metaid="s241" id="s241" name="lplprot" compartment="default" initialAmount="0">

<annotation>

<celldesigner:extension>

<celldesigner:positionToCompartment>inside</celldesigner:positionToCompartment>

<celldesigner:speciesIdentity>

<celldesigner:class>SIMPLE_MOLECULE</celldesigner:class>

<celldesigner:name>lplprot</celldesigner:name>

</celldesigner:speciesIdentity>

</celldesigner:extension>

</annotation>

</species>

<species metaid="s243" id="s243" name="dhlplprot" compartment="default" initialAmount="0">

<annotation>

<celldesigner:extension>

<celldesigner:positionToCompartment>inside</celldesigner:positionToCompartment>

<celldesigner:speciesIdentity>

<celldesigner:class>SIMPLE_MOLECULE</celldesigner:class>

<celldesigner:name>dhlplprot</celldesigner:name>

</celldesigner:speciesIdentity>

</celldesigner:extension>

</annotation>

</species>

<species metaid="s240" id="s240" name="amlplprot" compartment="default" initialAmount="0">

<annotation>

<celldesigner:extension>

<celldesigner:positionToCompartment>inside</celldesigner:positionToCompartment>

<celldesigner:speciesIdentity>

<celldesigner:class>SIMPLE_MOLECULE</celldesigner:class>

<celldesigner:name>amlplprot</celldesigner:name>

</celldesigner:speciesIdentity>

</celldesigner:extension>

</annotation>

</species>

<species metaid="s474" id="s474" name="mthf" compartment="default" initialAmount="0" charge="0">

<annotation>

<celldesigner:extension>

<celldesigner:positionToCompartment>inside</celldesigner:positionToCompartment>

<celldesigner:speciesIdentity>

<celldesigner:class>SIMPLE_MOLECULE</celldesigner:class>

<celldesigner:name>mthf</celldesigner:name>

</celldesigner:speciesIdentity>

</celldesigner:extension>

</annotation>

</species>

<species metaid="s259" id="s259" name="42aphdob" compartment="default" initialAmount="0">

<annotation>

<celldesigner:extension>

<celldesigner:positionToCompartment>inside</celldesigner:positionToCompartment>

<celldesigner:speciesIdentity>

<celldesigner:class>SIMPLE_MOLECULE</celldesigner:class>

<celldesigner:name>42aphdob</celldesigner:name>

</celldesigner:speciesIdentity>

</celldesigner:extension>

</annotation>

</species>

<species metaid="s234" id="s234" name="adesuc" compartment="default" initialAmount="0">

<annotation>

<celldesigner:extension>

<celldesigner:positionToCompartment>inside</celldesigner:positionToCompartment>

<celldesigner:speciesIdentity>

<celldesigner:class>SIMPLE_MOLECULE</celldesigner:class>

<celldesigner:name>adesuc</celldesigner:name>

</celldesigner:speciesIdentity>

</celldesigner:extension>

</annotation>

</species>

<species metaid="s244" id="s244" name="3mercpyr" compartment="default" initialAmount="0">

<annotation>

<celldesigner:extension>

<celldesigner:positionToCompartment>inside</celldesigner:positionToCompartment>

<celldesigner:speciesIdentity>

<celldesigner:class>SIMPLE_MOLECULE</celldesigner:class>

<celldesigner:name>3mercpyr</celldesigner:name>

</celldesigner:speciesIdentity>

</celldesigner:extension>

</annotation>

</species>

<species metaid="s482" id="s482" name="g-glucys" compartment="default" initialAmount="0" charge="0">

<annotation>

<celldesigner:extension>

<celldesigner:positionToCompartment>inside</celldesigner:positionToCompartment>

<celldesigner:speciesIdentity>

<celldesigner:class>SIMPLE_MOLECULE</celldesigner:class>

<celldesigner:name>g-glucys</celldesigner:name>

</celldesigner:speciesIdentity>

</celldesigner:extension>

</annotation>

</species>

</listOfSpecies>

<listOfReactions>

<reaction metaid="re4" id="re1" name="PDHComplex" reversible="false">

<annotation>

<celldesigner:extension>

<celldesigner:name>PDHComplex</celldesigner:name>

<celldesigner:reactionType>STATE_TRANSITION</celldesigner:reactionType>

<celldesigner:baseReactants>

<celldesigner:baseReactant species="s2" alias="sa143"/>

</celldesigner:baseReactants>

<celldesigner:baseProducts>

<celldesigner:baseProduct species="s7" alias="sa247"/>

</celldesigner:baseProducts>

<celldesigner:connectScheme connectPolicy="direct" rectangleIndex="0">

<celldesigner:listOfLineDirection>

<celldesigner:lineDirection index="0" value="unknown"/>

</celldesigner:listOfLineDirection>

</celldesigner:connectScheme>

<celldesigner:line width="1.0" color="ffff6633"/>

</celldesigner:extension>

</annotation>

<listOfReactants>

<speciesReference metaid="CDMT00001" species="s2">

<annotation>

<celldesigner:extension>

<celldesigner:alias>sa143</celldesigner:alias>

</celldesigner:extension>

</annotation>

</speciesReference>

</listOfReactants>

<listOfProducts>

<speciesReference metaid="CDMT00006" species="s7">

<annotation>

<celldesigner:extension>

<celldesigner:alias>sa247</celldesigner:alias>

</celldesigner:extension>

</annotation>

</speciesReference>

</listOfProducts>

</reaction>

<reaction metaid="re5" id="re2" name="CitS" reversible="false">

<annotation>

<celldesigner:extension>

<celldesigner:name>CitS</celldesigner:name>

<celldesigner:reactionType>STATE_TRANSITION</celldesigner:reactionType>

<celldesigner:baseReactants>

<celldesigner:baseReactant species="s7" alias="sa247"/>

</celldesigner:baseReactants>

<celldesigner:baseProducts>

<celldesigner:baseProduct species="s11" alias="sa245"/>

</celldesigner:baseProducts>

<celldesigner:listOfProductLinks>

<celldesigner:productLink product="s13" alias="sa243" targetLineIndex="-1,1">

<celldesigner:connectScheme connectPolicy="direct">

<celldesigner:listOfLineDirection>

<celldesigner:lineDirection index="0" value="unknown"/>

</celldesigner:listOfLineDirection>

</celldesigner:connectScheme>

<celldesigner:line width="1.0" color="ffff6633" type="Straight"/>

</celldesigner:productLink>

</celldesigner:listOfProductLinks>

<celldesigner:connectScheme connectPolicy="direct" rectangleIndex="0">

<celldesigner:listOfLineDirection>

<celldesigner:lineDirection index="0" value="unknown"/>

</celldesigner:listOfLineDirection>

</celldesigner:connectScheme>

<celldesigner:line width="1.0" color="ffff6633"/>

</celldesigner:extension>

</annotation>

<listOfReactants>

<speciesReference metaid="CDMT00009" species="s7">

<annotation>

<celldesigner:extension>

<celldesigner:alias>sa247</celldesigner:alias>

</celldesigner:extension>

</annotation>

</speciesReference>

</listOfReactants>

<listOfProducts>

<speciesReference metaid="CDMT00011" species="s11">

<annotation>

<celldesigner:extension>

<celldesigner:alias>sa245</celldesigner:alias>

</celldesigner:extension>

</annotation>

</speciesReference>

<speciesReference metaid="CDMT00012" species="s13">

<annotation>

<celldesigner:extension>

<celldesigner:alias>sa243</celldesigner:alias>

</celldesigner:extension>

</annotation>

</speciesReference>

</listOfProducts>

</reaction>

<reaction metaid="re6" id="re3" name="Aconitase" reversible="false">

<annotation>

<celldesigner:extension>

<celldesigner:name>Aconitase</celldesigner:name>

<celldesigner:reactionType>STATE_TRANSITION</celldesigner:reactionType>

<celldesigner:baseReactants>

<celldesigner:baseReactant species="s11" alias="sa245"/>

</celldesigner:baseReactants>

<celldesigner:baseProducts>

<celldesigner:baseProduct species="s14" alias="sa242"/>

</celldesigner:baseProducts>

<celldesigner:connectScheme connectPolicy="direct" rectangleIndex="0">

<celldesigner:listOfLineDirection>

<celldesigner:lineDirection index="0" value="unknown"/>

</celldesigner:listOfLineDirection>

</celldesigner:connectScheme>

<celldesigner:line width="1.0" color="ffff6633"/>

</celldesigner:extension>

</annotation>

<listOfReactants>

<speciesReference metaid="CDMT00014" species="s11">

<annotation>

<celldesigner:extension>

<celldesigner:alias>sa245</celldesigner:alias>

</celldesigner:extension>

</annotation>

</speciesReference>

</listOfReactants>

<listOfProducts>

<speciesReference metaid="CDMT00015" species="s14">

<annotation>

<celldesigner:extension>

<celldesigner:alias>sa242</celldesigner:alias>

</celldesigner:extension>

</annotation>

</speciesReference>

</listOfProducts>

</reaction>

<reaction metaid="re7" id="re4" name="Aconitase" reversible="false">

<annotation>

<celldesigner:extension>

<celldesigner:name>Aconitase</celldesigner:name>

<celldesigner:reactionType>STATE_TRANSITION</celldesigner:reactionType>

<celldesigner:baseReactants>

<celldesigner:baseReactant species="s14" alias="sa242"/>

</celldesigner:baseReactants>

<celldesigner:baseProducts>

<celldesigner:baseProduct species="s15" alias="sa241"/>

</celldesigner:baseProducts>

<celldesigner:connectScheme connectPolicy="direct" rectangleIndex="0">

<celldesigner:listOfLineDirection>

<celldesigner:lineDirection index="0" value="unknown"/>

</celldesigner:listOfLineDirection>

</celldesigner:connectScheme>

<celldesigner:line width="1.0" color="ff000000"/>

</celldesigner:extension>

</annotation>

<listOfReactants>

<speciesReference metaid="CDMT00017" species="s14">

<annotation>

<celldesigner:extension>

<celldesigner:alias>sa242</celldesigner:alias>

</celldesigner:extension>

</annotation>

</speciesReference>

</listOfReactants>

<listOfProducts>

<speciesReference metaid="CDMT00019" species="s15">

<annotation>

<celldesigner:extension>

<celldesigner:alias>sa241</celldesigner:alias>

</celldesigner:extension>

</annotation>

</speciesReference>

</listOfProducts>

</reaction>

<reaction metaid="re8" id="re5" name="IcitDH" reversible="false">

<annotation>

<celldesigner:extension>

<celldesigner:name>IcitDH</celldesigner:name>

<celldesigner:reactionType>STATE_TRANSITION</celldesigner:reactionType>

<celldesigner:baseReactants>

<celldesigner:baseReactant species="s15" alias="sa241"/>

</celldesigner:baseReactants>

<celldesigner:baseProducts>

<celldesigner:baseProduct species="s16" alias="sa238"/>

</celldesigner:baseProducts>

<celldesigner:connectScheme connectPolicy="direct" rectangleIndex="0">

<celldesigner:listOfLineDirection>

<celldesigner:lineDirection index="0" value="unknown"/>

</celldesigner:listOfLineDirection>

</celldesigner:connectScheme>

<celldesigner:line width="1.0" color="ffff0066"/>

</celldesigner:extension>

</annotation>

<listOfReactants>

<speciesReference metaid="CDMT00020" species="s15">

<annotation>

<celldesigner:extension>

<celldesigner:alias>sa241</celldesigner:alias>

</celldesigner:extension>

</annotation>

</speciesReference>

</listOfReactants>

<listOfProducts>

<speciesReference metaid="CDMT00022" species="s16">

<annotation>

<celldesigner:extension>

<celldesigner:alias>sa238</celldesigner:alias>

</celldesigner:extension>
</annotation>
</speciesReference>
</listOfProducts>
</reaction>
<reaction metaid="re9" id="re6" name="aKGDHComplex" reversible="false">
<annotation>
<celldesigner:extension>
<celldesigner:name>aKGDHComplex</celldesigner:name>
<celldesigner:reactionType>STATE_TRANSITION</celldesigner:reactionType>
<celldesigner:baseReactants>
<celldesigner:baseReactant species="s16" alias="sa238"/>
</celldesigner:baseReactants>
<celldesigner:baseProducts>
<celldesigner:baseProduct species="s22" alias="sa235"/>
</celldesigner:baseProducts>
<celldesigner:connectScheme connectPolicy="direct" rectangleIndex="0">
<celldesigner:listOfLineDirection>
<celldesigner:lineDirection index="0" value="unknown"/>
</celldesigner:listOfLineDirection>
</celldesigner:connectScheme>
<celldesigner:line width="1.0" color="ff000000"/>
</celldesigner:extension>
</annotation>
<listOfReactants>
<speciesReference metaid="CDMT00025" species="s16">
<annotation>

<celldesigner:extension>

<celldesigner:alias>sa238</celldesigner:alias>

</celldesigner:extension>

</annotation>

</speciesReference>

</listOfReactants>

<listOfProducts>

<speciesReference metaid="CDMT00028" species="s22">

<annotation>

<celldesigner:extension>

<celldesigner:alias>sa235</celldesigner:alias>

</celldesigner:extension>

</annotation>

</speciesReference>

</listOfProducts>

</reaction>

<reaction metaid="re14" id="re7" name="ScCoaS" reversible="false">

<annotation>

<celldesigner:extension>

<celldesigner:name>ScCoaS</celldesigner:name>

<celldesigner:reactionType>STATE_TRANSITION</celldesigner:reactionType>

<celldesigner:baseReactants>

<celldesigner:baseReactant species="s22" alias="sa235"/>

</celldesigner:baseReactants>

<celldesigner:baseProducts>

<celldesigner:baseProduct species="s24" alias="sa234"/>

</celldesigner:baseProducts>

<celldesigner:connectScheme connectPolicy="direct" rectangleIndex="0">

<celldesigner:listOfLineDirection>

<celldesigner:lineDirection index="0" value="unknown"/>

</celldesigner:listOfLineDirection>

</celldesigner:connectScheme>

<celldesigner:line width="1.0" color="ff000000"/>

</celldesigner:extension>

</annotation>

<listOfReactants>

<speciesReference metaid="CDMT00032" species="s22">

<annotation>

<celldesigner:extension>

<celldesigner:alias>sa235</celldesigner:alias>

</celldesigner:extension>

</annotation>

</speciesReference>

</listOfReactants>

<listOfProducts>

<speciesReference metaid="CDMT00035" species="s24">

<annotation>

<celldesigner:extension>

<celldesigner:alias>sa234</celldesigner:alias>

</celldesigner:extension>

</annotation>

</speciesReference>

</listOfProducts>

</reaction>

```
<reaction metaid="re15" id="re8" name="sDH(CII)" reversible="false">
<annotation>
<celldesigner:extension>
<celldesigner:name>sDH(CII)</celldesigner:name>
<celldesigner:reactionType>STATE_TRANSITION</celldesigner:reactionType>
<celldesigner:baseReactants>
<celldesigner:baseReactant species="s24" alias="sa234">
<celldesigner:linkAnchor position="NNE"/>
</celldesigner:baseReactant>
</celldesigner:baseReactants>
<celldesigner:baseProducts>
<celldesigner:baseProduct species="s28" alias="sa230">
<celldesigner:linkAnchor position="SSE"/>
</celldesigner:baseProduct>
</celldesigner:baseProducts>
<celldesigner:connectScheme connectPolicy="direct" rectangleIndex="0">
<celldesigner:listOfLineDirection>
<celldesigner:lineDirection index="0" value="unknown"/>
</celldesigner:listOfLineDirection>
</celldesigner:connectScheme>
<celldesigner:line width="1.0" color="ffff0066"/>
</celldesigner:extension>
</annotation>
<listOfReactants>
<speciesReference metaid="CDMT00037" species="s24">
<annotation>
<celldesigner:extension>
```

```
<celldesigner:alias>sa234</celldesigner:alias>
</celldesigner:extension>
</annotation>
</speciesReference>
</listOfReactants>
<listOfProducts>
<speciesReference metaid="CDMT00039" species="s28">
<annotation>
<celldesigner:extension>
<celldesigner:alias>sa230</celldesigner:alias>
</celldesigner:extension>
</annotation>
</speciesReference>
</listOfProducts>
</reaction>
<reaction metaid="re17" id="re10" name="mFumerase" reversible="false">
<annotation>
<celldesigner:extension>
<celldesigner:name>mFumerase</celldesigner:name>
<celldesigner:reactionType>STATE_TRANSITION</celldesigner:reactionType>
<celldesigner:baseReactants>
<celldesigner:baseReactant species="s28" alias="sa230"/>
</celldesigner:baseReactants>
<celldesigner:baseProducts>
<celldesigner:baseProduct species="s33" alias="sa226"/>
</celldesigner:baseProducts>
<celldesigner:connectScheme connectPolicy="direct" rectangleIndex="0">
```

<celldesigner:listOfLineDirection>
<celldesigner:lineDirection index="0" value="unknown"/>
</celldesigner:listOfLineDirection>
</celldesigner:connectScheme>
<celldesigner:line width="1.0" color="ffff0066"/>
</celldesigner:extension>
</annotation>
<listOfReactants>
<speciesReference metaid="CDMT00046" species="s28">
<annotation>
<celldesigner:extension>
<celldesigner:alias>sa230</celldesigner:alias>
</celldesigner:extension>
</annotation>
</speciesReference>
</listOfReactants>
<listOfProducts>
<speciesReference metaid="CDMT00048" species="s33">
<annotation>
<celldesigner:extension>
<celldesigner:alias>sa226</celldesigner:alias>
</celldesigner:extension>
</annotation>
</speciesReference>
</listOfProducts>
</reaction>
<reaction metaid="re19" id="re12" name="MalicEnzyme" reversible="false">

<annotation>
<celldesigner:extension>
<celldesigner:name>MalicEnzyme</celldesigner:name>
<celldesigner:reactionType>STATE_TRANSITION</celldesigner:reactionType>
<celldesigner:baseReactants>
<celldesigner:baseReactant species="s33" alias="sa226"/>
</celldesigner:baseReactants>
<celldesigner:baseProducts>
<celldesigner:baseProduct species="s2" alias="sa143">
<celldesigner:linkAnchor position="SE"/>
</celldesigner:baseProduct>
</celldesigner:baseProducts>
<celldesigner:connectScheme connectPolicy="direct" rectangleIndex="0">
<celldesigner:listOfLineDirection>
<celldesigner:lineDirection index="0" value="unknown"/>
</celldesigner:listOfLineDirection>
</celldesigner:connectScheme>
<celldesigner:line width="1.0" color="ffff6633"/>
</celldesigner:extension>
</annotation>
<listOfReactants>
<speciesReference metaid="CDMT00054" species="s33">
<annotation>
<celldesigner:extension>
<celldesigner:alias>sa226</celldesigner:alias>
</celldesigner:extension>
</annotation>

</speciesReference>

</listOfReactants>

<listOfProducts>

<speciesReference metaid="CDMT00056" species="s2">

<annotation>

<celldesigner:extension>

<celldesigner:alias>sa143</celldesigner:alias>

</celldesigner:extension>

</annotation>

</speciesReference>

</listOfProducts>

</reaction>

<reaction metaid="re24" id="re13" name="ACoATr" reversible="false">

<annotation>

<celldesigner:extension>

<celldesigner:name>ACoATr</celldesigner:name>

<celldesigner:reactionType>STATE_TRANSITION</celldesigner:reactionType>

<celldesigner:baseReactants>

<celldesigner:baseReactant species="s7" alias="sa247"/>

</celldesigner:baseReactants>

<celldesigner:baseProducts>

<celldesigner:baseProduct species="s37" alias="sa222"/>

</celldesigner:baseProducts>

<celldesigner:listOfReactantLinks>

<celldesigner:reactantLink reactant="s24" alias="sa234" targetLineIndex="-1,0">

<celldesigner:connectScheme connectPolicy="direct">

<celldesigner:listOfLineDirection>

<celldesigner:lineDirection index="0" value="unknown"/>

<celldesigner:lineDirection index="1" value="unknown"/>

<celldesigner:lineDirection index="2" value="unknown"/>

</celldesigner:listOfLineDirection>

</celldesigner:connectScheme>

<celldesigner:editPoints>0.3684321907644317,-0.13094234569930463 0.9642575954793693,-0.3683694190062772</celldesigner:editPoints>

<celldesigner:line width="1.0" color="ffff6633" type="Straight"/>

</celldesigner:reactantLink>

</celldesigner:listOfReactantLinks>

<celldesigner:connectScheme connectPolicy="direct" rectangleIndex="0">

<celldesigner:listOfLineDirection>

<celldesigner:lineDirection index="0" value="unknown"/>

</celldesigner:listOfLineDirection>

</celldesigner:connectScheme>

<celldesigner:line width="1.0" color="ffff6633"/>

</celldesigner:extension>

</annotation>

<listOfReactants>

<speciesReference metaid="CDMT00059" species="s7">

<annotation>

<celldesigner:extension>

<celldesigner:alias>sa247</celldesigner:alias>

</celldesigner:extension>

</annotation>

</speciesReference>

<speciesReference metaid="CDMT00060" species="s24">

<annotation>

<celldesigner:extension>

<celldesigner:alias>sa234</celldesigner:alias>

</celldesigner:extension>

</annotation>

</speciesReference>

</listOfReactants>

<listOfProducts>

<speciesReference metaid="CDMT00061" species="s37">

<annotation>

<celldesigner:extension>

<celldesigner:alias>sa222</celldesigner:alias>

</celldesigner:extension>

</annotation>

</speciesReference>

</listOfProducts>

</reaction>

<reaction metaid="re46" id="re15" name="HK[bDglc6p]" reversible="false">

<annotation>

<celldesigner:extension>

<celldesigner:name>HK[bDglc6p]</celldesigner:name>

<celldesigner:reactionType>STATE_TRANSITION</celldesigner:reactionType>

<celldesigner:baseReactants>

<celldesigner:baseReactant species="s52" alias="sa3"/>

</celldesigner:baseReactants>

<celldesigner:baseProducts>

<celldesigner:baseProduct species="s54" alias="sa23"/>

</celldesigner:baseProducts>

<celldesigner:connectScheme connectPolicy="direct" rectangleIndex="0">
<celldesigner:listOfLineDirection>
<celldesigner:lineDirection index="0" value="unknown"/>
</celldesigner:listOfLineDirection>
</celldesigner:connectScheme>
<celldesigner:line width="1.0" color="ffff6633"/>
</celldesigner:extension>
</annotation>
<listOfReactants>
<speciesReference metaid="CDMT00066" species="s52">
<annotation>
<celldesigner:extension>
<celldesigner:alias>sa3</celldesigner:alias>
</celldesigner:extension>
</annotation>
</speciesReference>
</listOfReactants>
<listOfProducts>
<speciesReference metaid="CDMT00068" species="s54">
<annotation>
<celldesigner:extension>
<celldesigner:alias>sa23</celldesigner:alias>
</celldesigner:extension>
</annotation>
</speciesReference>
</listOfProducts>
</reaction>

<reaction metaid="re48" id="re17" name="HK[bDglc6p-bDfr6p]" reversible="false">

<annotation>

<celldesigner:extension>

<celldesigner:name>HK[bDglc6p-bDfr6p]</celldesigner:name>

<celldesigner:reactionType>STATE_TRANSITION</celldesigner:reactionType>

<celldesigner:baseReactants>

<celldesigner:baseReactant species="s54" alias="sa23"/>

</celldesigner:baseReactants>

<celldesigner:baseProducts>

<celldesigner:baseProduct species="s57" alias="sa22"/>

</celldesigner:baseProducts>

<celldesigner:connectScheme connectPolicy="direct" rectangleIndex="0">

<celldesigner:listOfLineDirection>

<celldesigner:lineDirection index="0" value="unknown"/>

</celldesigner:listOfLineDirection>

</celldesigner:connectScheme>

<celldesigner:line width="1.0" color="ffff6633"/>

</celldesigner:extension>

</annotation>

<listOfReactants>

<speciesReference metaid="CDMT00074" species="s54">

<annotation>

<celldesigner:extension>

<celldesigner:alias>sa23</celldesigner:alias>

</celldesigner:extension>

</annotation>

</speciesReference>

</listOfReactants>

<listOfProducts>

<speciesReference metaid="CDMT00076" species="s57">

<annotation>

<celldesigner:extension>

<celldesigner:alias>sa22</celldesigner:alias>

</celldesigner:extension>

</annotation>

</speciesReference>

</listOfProducts>

</reaction>

<reaction metaid="re49" id="re18" name="PFK" reversible="false">

<annotation>

<celldesigner:extension>

<celldesigner:name>PFK</celldesigner:name>

<celldesigner:reactionType>STATE_TRANSITION</celldesigner:reactionType>

<celldesigner:baseReactants>

<celldesigner:baseReactant species="s57" alias="sa22">

<celldesigner:linkAnchor position="SSE"/>

</celldesigner:baseReactant>

</celldesigner:baseReactants>

<celldesigner:baseProducts>

<celldesigner:baseProduct species="s58" alias="sa35"/>

</celldesigner:baseProducts>

<celldesigner:connectScheme connectPolicy="direct" rectangleIndex="0">

<celldesigner:listOfLineDirection>

<celldesigner:lineDirection index="0" value="unknown"/>

</celldesigner:listOfLineDirection>

</celldesigner:connectScheme>

<celldesigner:line width="1.0" color="ffff6633"/>

</celldesigner:extension>

</annotation>

<listOfReactants>

<speciesReference metaid="CDMT00078" species="s57">

<annotation>

<celldesigner:extension>

<celldesigner:alias>sa22</celldesigner:alias>

</celldesigner:extension>

</annotation>

</speciesReference>

</listOfReactants>

<listOfProducts>

<speciesReference metaid="CDMT00080" species="s58">

<annotation>

<celldesigner:extension>

<celldesigner:alias>sa35</celldesigner:alias>

</celldesigner:extension>

</annotation>

</speciesReference>

</listOfProducts>

</reaction>

<reaction metaid="re51" id="re20" name="ALD" reversible="false">

<annotation>

<celldesigner:extension>

<celldesigner:name>ALD</celldesigner:name>

<celldesigner:reactionType>STATE_TRANSITION</celldesigner:reactionType>

<celldesigner:baseReactants>

<celldesigner:baseReactant species="s58" alias="sa35"/>

</celldesigner:baseReactants>

<celldesigner:baseProducts>

<celldesigner:baseProduct species="s59" alias="sa44"/>

</celldesigner:baseProducts>

<celldesigner:listOfProductLinks>

<celldesigner:productLink product="s60" alias="sa43" targetLineIndex="-1,1">

<celldesigner:connectScheme connectPolicy="direct">

<celldesigner:listOfLineDirection>

<celldesigner:lineDirection index="0" value="unknown"/>

</celldesigner:listOfLineDirection>

</celldesigner:connectScheme>

<celldesigner:line width="1.0" color="ffff0066" type="Straight"/>

</celldesigner:productLink>

</celldesigner:listOfProductLinks>

<celldesigner:connectScheme connectPolicy="direct" rectangleIndex="0">

<celldesigner:listOfLineDirection>

<celldesigner:lineDirection index="0" value="unknown"/>

</celldesigner:listOfLineDirection>

</celldesigner:connectScheme>

<celldesigner:line width="1.0" color="ffff6633"/>

</celldesigner:extension>

</annotation>

<listOfReactants>

<speciesReference metaid="CDMT00086" species="s58">

<annotation>

<celldesigner:extension>

<celldesigner:alias>sa35</celldesigner:alias>

</celldesigner:extension>

</annotation>

</speciesReference>

</listOfReactants>

<listOfProducts>

<speciesReference metaid="CDMT00087" species="s59">

<annotation>

<celldesigner:extension>

<celldesigner:alias>sa44</celldesigner:alias>

</celldesigner:extension>

</annotation>

</speciesReference>

<speciesReference metaid="CDMT00088" species="s60">

<annotation>

<celldesigner:extension>

<celldesigner:alias>sa43</celldesigner:alias>

</celldesigner:extension>

</annotation>

</speciesReference>

</listOfProducts>

</reaction>

<reaction metaid="re54" id="re22" name="G3PORD" reversible="false">

<annotation>

<celldesigner:extension>

<celldesigner:name>G3PORD</celldesigner:name>

<celldesigner:reactionType>STATE_TRANSITION</celldesigner:reactionType>

<celldesigner:baseReactants>

<celldesigner:baseReactant species="s60" alias="sa43"/>

</celldesigner:baseReactants>

<celldesigner:baseProducts>

<celldesigner:baseProduct species="s61" alias="sa409"/>

</celldesigner:baseProducts>

<celldesigner:connectScheme connectPolicy="direct" rectangleIndex="0">

<celldesigner:listOfLineDirection>

<celldesigner:lineDirection index="0" value="unknown"/>

</celldesigner:listOfLineDirection>

</celldesigner:connectScheme>

<celldesigner:line width="1.0" color="ff00ffff"/>

</celldesigner:extension>

</annotation>

<listOfReactants>

<speciesReference metaid="CDMT00091" species="s60">

<annotation>

<celldesigner:extension>

<celldesigner:alias>sa43</celldesigner:alias>

</celldesigner:extension>

</annotation>

</speciesReference>

</listOfReactants>

<listOfProducts>

<speciesReference metaid="CDMT00093" species="s61">
<annotation>
<celldesigner:extension>
<celldesigner:alias>sa409</celldesigner:alias>
</celldesigner:extension>
</annotation>
</speciesReference>
</listOfProducts>
</reaction>
<reaction metaid="re56" id="re23" name="GAPDH" reversible="false">
<annotation>
<celldesigner:extension>
<celldesigner:name>GAPDH</celldesigner:name>
<celldesigner:reactionType>STATE_TRANSITION</celldesigner:reactionType>
<celldesigner:baseReactants>
<celldesigner:baseReactant species="s59" alias="sa44"/>
</celldesigner:baseReactants>
<celldesigner:baseProducts>
<celldesigner:baseProduct species="s63" alias="sa60"/>
</celldesigner:baseProducts>
<celldesigner:connectScheme connectPolicy="direct" rectangleIndex="0">
<celldesigner:listOfLineDirection>
<celldesigner:lineDirection index="0" value="unknown"/>
</celldesigner:listOfLineDirection>
</celldesigner:connectScheme>
<celldesigner:line width="1.0" color="ffff6633"/>
</celldesigner:extension>

</annotation>
<listOfReactants>
<speciesReference metaid="CDMT00095" species="s59">
<annotation>
<celldesigner:extension>
<celldesigner:alias>sa44</celldesigner:alias>
</celldesigner:extension>
</annotation>
</speciesReference>
</listOfReactants>
<listOfProducts>
<speciesReference metaid="CDMT00098" species="s63">
<annotation>
<celldesigner:extension>
<celldesigner:alias>sa60</celldesigner:alias>
</celldesigner:extension>
</annotation>
</speciesReference>
</listOfProducts>
</reaction>
<reaction metaid="re57" id="re24" name="PGK" reversible="false">
<annotation>
<celldesigner:extension>
<celldesigner:name>PGK</celldesigner:name>
<celldesigner:reactionType>STATE_TRANSITION</celldesigner:reactionType>
<celldesigner:baseReactants>
<celldesigner:baseReactant species="s63" alias="sa60"/>

</celldesigner:baseReactants>
<celldesigner:baseProducts>
<celldesigner:baseProduct species="s64" alias="sa74"/>
</celldesigner:baseProducts>
<celldesigner:connectScheme connectPolicy="direct" rectangleIndex="0">
<celldesigner:listOfLineDirection>
<celldesigner:lineDirection index="0" value="unknown"/>
</celldesigner:listOfLineDirection>
</celldesigner:connectScheme>
<celldesigner:line width="1.0" color="ffff6633"/>
</celldesigner:extension>
</annotation>
<listOfReactants>
<speciesReference metaid="CDMT00101" species="s63">
<annotation>
<celldesigner:extension>
<celldesigner:alias>sa60</celldesigner:alias>
</celldesigner:extension>
</annotation>
</speciesReference>
</listOfReactants>
<listOfProducts>
<speciesReference metaid="CDMT00103" species="s64">
<annotation>
<celldesigner:extension>
<celldesigner:alias>sa74</celldesigner:alias>
</celldesigner:extension>

</annotation>

</speciesReference>

</listOfProducts>

</reaction>

<reaction metaid="re58" id="re25" name="PGM" reversible="false">

<annotation>

<celldesigner:extension>

<celldesigner:name>PGM</celldesigner:name>

<celldesigner:reactionType>STATE_TRANSITION</celldesigner:reactionType>

<celldesigner:baseReactants>

<celldesigner:baseReactant species="s64" alias="sa74"/>

</celldesigner:baseReactants>

<celldesigner:baseProducts>

<celldesigner:baseProduct species="s65" alias="sa89"/>

</celldesigner:baseProducts>

<celldesigner:connectScheme connectPolicy="direct" rectangleIndex="0">

<celldesigner:listOfLineDirection>

<celldesigner:lineDirection index="0" value="unknown"/>

</celldesigner:listOfLineDirection>

</celldesigner:connectScheme>

<celldesigner:line width="1.0" color="ffff6633"/>

</celldesigner:extension>

</annotation>

<listOfReactants>

<speciesReference metaid="CDMT00105" species="s64">

<annotation>

<celldesigner:extension>

<celldesigner:alias>sa74</celldesigner:alias>

</celldesigner:extension>

</annotation>

</speciesReference>

</listOfReactants>

<listOfProducts>

<speciesReference metaid="CDMT00106" species="s65">

<annotation>

<celldesigner:extension>

<celldesigner:alias>sa89</celldesigner:alias>

</celldesigner:extension>

</annotation>

</speciesReference>

</listOfProducts>

</reaction>

<reaction metaid="re59" id="re26" name="Enolase" reversible="false">

<annotation>

<celldesigner:extension>

<celldesigner:name>Enolase</celldesigner:name>

<celldesigner:reactionType>STATE_TRANSITION</celldesigner:reactionType>

<celldesigner:baseReactants>

<celldesigner:baseReactant species="s65" alias="sa89"/>

</celldesigner:baseReactants>

<celldesigner:baseProducts>

<celldesigner:baseProduct species="s66" alias="sa108"/>

</celldesigner:baseProducts>

<celldesigner:connectScheme connectPolicy="direct" rectangleIndex="0">

<celldesigner:listOfLineDirection>

<celldesigner:lineDirection index="0" value="unknown"/>

</celldesigner:listOfLineDirection>

</celldesigner:connectScheme>

<celldesigner:line width="1.0" color="ffff6633"/>

</celldesigner:extension>

</annotation>

<listOfReactants>

<speciesReference metaid="CDMT00107" species="s65">

<annotation>

<celldesigner:extension>

<celldesigner:alias>sa89</celldesigner:alias>

</celldesigner:extension>

</annotation>

</speciesReference>

</listOfReactants>

<listOfProducts>

<speciesReference metaid="CDMT00108" species="s66">

<annotation>

<celldesigner:extension>

<celldesigner:alias>sa108</celldesigner:alias>

</celldesigner:extension>

</annotation>

</speciesReference>

</listOfProducts>

</reaction>

<reaction metaid="re60" id="re27" name="PK" reversible="false">

<annotation>
<celldesigner:extension>
<celldesigner:name>PK</celldesigner:name>
<celldesigner:reactionType>STATE_TRANSITION</celldesigner:reactionType>
<celldesigner:baseReactants>
<celldesigner:baseReactant species="s66" alias="sa108"/>
</celldesigner:baseReactants>
<celldesigner:baseProducts>
<celldesigner:baseProduct species="s2" alias="sa143">
<celldesigner:linkAnchor position="N"/>
</celldesigner:baseProduct>
</celldesigner:baseProducts>
<celldesigner:connectScheme connectPolicy="direct" rectangleIndex="0">
<celldesigner:listOfLineDirection>
<celldesigner:lineDirection index="0" value="unknown"/>
</celldesigner:listOfLineDirection>
</celldesigner:connectScheme>
<celldesigner:line width="1.0" color="ffff6633"/>
</celldesigner:extension>
</annotation>
<listOfReactants>
<speciesReference metaid="CDMT00110" species="s66">
<annotation>
<celldesigner:extension>
<celldesigner:alias>sa108</celldesigner:alias>
</celldesigner:extension>
</annotation>

</speciesReference>

</listOfReactants>

<listOfProducts>

<speciesReference metaid="CDMT00213" species="s2" stoichiometry="2">

<annotation>

<celldesigner:extension>

<celldesigner:alias>sa143</celldesigner:alias>

</celldesigner:extension>

</annotation>

</speciesReference>

</listOfProducts>

</reaction>

<reaction metaid="re76" id="re34" name="PMI" reversible="false">

<annotation>

<celldesigner:extension>

<celldesigner:name>PMI</celldesigner:name>

<celldesigner:reactionType>STATE_TRANSITION</celldesigner:reactionType>

<celldesigner:baseReactants>

<celldesigner:baseReactant species="s57" alias="sa22"/>

</celldesigner:baseReactants>

<celldesigner:baseProducts>

<celldesigner:baseProduct species="s82" alias="sa19"/>

</celldesigner:baseProducts>

<celldesigner:connectScheme connectPolicy="direct" rectangleIndex="0">

<celldesigner:listOfLineDirection>

<celldesigner:lineDirection index="0" value="unknown"/>

</celldesigner:listOfLineDirection>

</celldesigner:connectScheme>

<celldesigner:line width="1.0" color="ffff6633"/>

</celldesigner:extension>

</annotation>

<listOfReactants>

<speciesReference metaid="CDMT00134" species="s57">

<annotation>

<celldesigner:extension>

<celldesigner:alias>sa22</celldesigner:alias>

</celldesigner:extension>

</annotation>

</speciesReference>

</listOfReactants>

<listOfProducts>

<speciesReference metaid="CDMT00135" species="s82">

<annotation>

<celldesigner:extension>

<celldesigner:alias>sa19</celldesigner:alias>

</celldesigner:extension>

</annotation>

</speciesReference>

</listOfProducts>

</reaction>

<reaction metaid="re77" id="re35" name="PMM" reversible="false">

<annotation>

<celldesigner:extension>

<celldesigner:name>PMM</celldesigner:name>

<celldesigner:reactionType>STATE_TRANSITION</celldesigner:reactionType>
<celldesigner:baseReactants>
<celldesigner:baseReactant species="s82" alias="sa19"/>
</celldesigner:baseReactants>
<celldesigner:baseProducts>
<celldesigner:baseProduct species="s83" alias="sa18"/>
</celldesigner:baseProducts>
<celldesigner:connectScheme connectPolicy="direct" rectangleIndex="0">
<celldesigner:listOfLineDirection>
<celldesigner:lineDirection index="0" value="unknown"/>
</celldesigner:listOfLineDirection>
</celldesigner:connectScheme>
<celldesigner:line width="1.0" color="ffff6633"/>
</celldesigner:extension>
</annotation>
<listOfReactants>
<speciesReference metaid="CDMT00136" species="s82">
<annotation>
<celldesigner:extension>
<celldesigner:alias>sa19</celldesigner:alias>
</celldesigner:extension>
</annotation>
</speciesReference>
</listOfReactants>
<listOfProducts>
<speciesReference metaid="CDMT00137" species="s83">
<annotation>

<celldesigner:extension>

<celldesigner:alias>sa18</celldesigner:alias>

</celldesigner:extension>

</annotation>

</speciesReference>

</listOfProducts>

</reaction>

<reaction metaid="re78" id="re36" name="Man1PGuanylylTr" reversible="false">

<annotation>

<celldesigner:extension>

<celldesigner:name>Man1PGuanylylTr</celldesigner:name>

<celldesigner:reactionType>STATE_TRANSITION</celldesigner:reactionType>

<celldesigner:baseReactants>

<celldesigner:baseReactant species="s83" alias="sa18"/>

</celldesigner:baseReactants>

<celldesigner:baseProducts>

<celldesigner:baseProduct species="s84" alias="sa17"/>

</celldesigner:baseProducts>

<celldesigner:connectScheme connectPolicy="direct" rectangleIndex="0">

<celldesigner:listOfLineDirection>

<celldesigner:lineDirection index="0" value="unknown"/>

</celldesigner:listOfLineDirection>

</celldesigner:connectScheme>

<celldesigner:line width="1.0" color="ffff6633"/>

</celldesigner:extension>

</annotation>

<listOfReactants>

<speciesReference metaid="CDMT00138" species="s83">

<annotation>

<celldesigner:extension>

<celldesigner:alias>sa18</celldesigner:alias>

</celldesigner:extension>

</annotation>

</speciesReference>

</listOfReactants>

<listOfProducts>

<speciesReference metaid="CDMT00140" species="s84">

<annotation>

<celldesigner:extension>

<celldesigner:alias>sa17</celldesigner:alias>

</celldesigner:extension>

</annotation>

</speciesReference>

</listOfProducts>

</reaction>

<reaction metaid="re79" id="re37" name="MannanSynthesis" reversible="false">

<annotation>

<celldesigner:extension>

<celldesigner:name>MannanSynthesis</celldesigner:name>

<celldesigner:reactionType>STATE_TRANSITION</celldesigner:reactionType>

<celldesigner:baseReactants>

<celldesigner:baseReactant species="s84" alias="sa17"/>

</celldesigner:baseReactants>

<celldesigner:baseProducts>

<celldesigner:baseProduct species="s85" alias="sa30"/>

</celldesigner:baseProducts>

<celldesigner:connectScheme connectPolicy="direct" rectangleIndex="0">

<celldesigner:listOfLineDirection>

<celldesigner:lineDirection index="0" value="unknown"/>

</celldesigner:listOfLineDirection>

</celldesigner:connectScheme>

<celldesigner:line width="1.0" color="ffff6633"/>

</celldesigner:extension>

</annotation>

<listOfReactants>

<speciesReference metaid="CDMT00143" species="s84">

<annotation>

<celldesigner:extension>

<celldesigner:alias>sa17</celldesigner:alias>

</celldesigner:extension>

</annotation>

</speciesReference>

</listOfReactants>

<listOfProducts>

<speciesReference metaid="CDMT00144" species="s85">

<annotation>

<celldesigner:extension>

<celldesigner:alias>sa30</celldesigner:alias>

</celldesigner:extension>

</annotation>

</speciesReference>

</listOfProducts>
</reaction>
<reaction metaid="re83" id="re83" name="G6PDH" reversible="false">
<annotation>
<celldesigner:extension>
<celldesigner:name>G6PDH</celldesigner:name>
<celldesigner:reactionType>STATE_TRANSITION</celldesigner:reactionType>
<celldesigner:baseReactants>
<celldesigner:baseReactant species="s54" alias="sa23"/>
</celldesigner:baseReactants>
<celldesigner:baseProducts>
<celldesigner:baseProduct species="s1" alias="sa321"/>
</celldesigner:baseProducts>
<celldesigner:connectScheme connectPolicy="direct" rectangleIndex="0">
<celldesigner:listOfLineDirection>
<celldesigner:lineDirection index="0" value="unknown"/>
</celldesigner:listOfLineDirection>
</celldesigner:connectScheme>
<celldesigner:line width="1.0" color="ffff6633"/>
</celldesigner:extension>
</annotation>
<listOfReactants>
<speciesReference metaid="CDMT00169" species="s54">
<annotation>
<celldesigner:extension>
<celldesigner:alias>sa23</celldesigner:alias>
</celldesigner:extension>

</annotation>

</speciesReference>

</listOfReactants>

<listOfProducts>

<speciesReference metaid="CDMT00171" species="s1">

<annotation>

<celldesigner:extension>

<celldesigner:alias>sa321</celldesigner:alias>

</celldesigner:extension>

</annotation>

</speciesReference>

</listOfProducts>

</reaction>

<reaction metaid="re84" id="re84" name="Gluconolactonase" reversible="false">

<annotation>

<celldesigner:extension>

<celldesigner:name>Gluconolactonase</celldesigner:name>

<celldesigner:reactionType>STATE_TRANSITION</celldesigner:reactionType>

<celldesigner:baseReactants>

<celldesigner:baseReactant species="s1" alias="sa321"/>

</celldesigner:baseReactants>

<celldesigner:baseProducts>

<celldesigner:baseProduct species="s3" alias="sa322"/>

</celldesigner:baseProducts>

<celldesigner:connectScheme connectPolicy="direct" rectangleIndex="0">

<celldesigner:listOfLineDirection>

<celldesigner:lineDirection index="0" value="unknown"/>

</celldesigner:listOfLineDirection>

</celldesigner:connectScheme>

<celldesigner:line width="1.0" color="ffff6633"/>

</celldesigner:extension>

</annotation>

<listOfReactants>

<speciesReference metaid="CDMT00174" species="s1">

<annotation>

<celldesigner:extension>

<celldesigner:alias>sa321</celldesigner:alias>

</celldesigner:extension>

</annotation>

</speciesReference>

</listOfReactants>

<listOfProducts>

<speciesReference metaid="CDMT00176" species="s3">

<annotation>

<celldesigner:extension>

<celldesigner:alias>sa322</celldesigner:alias>

</celldesigner:extension>

</annotation>

</speciesReference>

</listOfProducts>

</reaction>

<reaction metaid="re85" id="re85" name="6PGDH" reversible="false">

<annotation>

<celldesigner:extension>

<celldesigner:name>6PGDH</celldesigner:name>

<celldesigner:reactionType>STATE_TRANSITION</celldesigner:reactionType>

<celldesigner:baseReactants>

<celldesigner:baseReactant species="s3" alias="sa322"/>

</celldesigner:baseReactants>

<celldesigner:baseProducts>

<celldesigner:baseProduct species="s5" alias="sa333"/>

</celldesigner:baseProducts>

<celldesigner:connectScheme connectPolicy="direct" rectangleIndex="0">

<celldesigner:listOfLineDirection>

<celldesigner:lineDirection index="0" value="unknown"/>

</celldesigner:listOfLineDirection>

</celldesigner:connectScheme>

<celldesigner:line width="1.0" color="ffff6633"/>

</celldesigner:extension>

</annotation>

<listOfReactants>

<speciesReference metaid="CDMT00178" species="s3">

<annotation>

<celldesigner:extension>

<celldesigner:alias>sa322</celldesigner:alias>

</celldesigner:extension>

</annotation>

</speciesReference>

</listOfReactants>

<listOfProducts>

<speciesReference metaid="CDMT00180" species="s5">

<annotation>
<celldesigner:extension>
<celldesigner:alias>sa333</celldesigner:alias>
</celldesigner:extension>
</annotation>
</speciesReference>
</listOfProducts>
</reaction>
<reaction metaid="re86" id="re86" name="R5PIB" reversible="false">
<annotation>
<celldesigner:extension>
<celldesigner:name>R5PIB</celldesigner:name>
<celldesigner:reactionType>STATE_TRANSITION</celldesigner:reactionType>
<celldesigner:baseReactants>
<celldesigner:baseReactant species="s5" alias="sa333"/>
</celldesigner:baseReactants>
<celldesigner:baseProducts>
<celldesigner:baseProduct species="s432" alias="sa349"/>
</celldesigner:baseProducts>
<celldesigner:connectScheme connectPolicy="direct" rectangleIndex="0">
<celldesigner:listOfLineDirection>
<celldesigner:lineDirection index="0" value="unknown"/>
</celldesigner:listOfLineDirection>
</celldesigner:connectScheme>
<celldesigner:line width="1.0" color="ffff6633"/>
</celldesigner:extension>
</annotation>

<listOfReactants>
<speciesReference metaid="CDMT00184" species="s5">
<annotation>
<celldesigner:extension>
<celldesigner:alias>sa333</celldesigner:alias>
</celldesigner:extension>
</annotation>
</speciesReference>
</listOfReactants>
<listOfProducts>
<speciesReference metaid="CDMT00185" species="s432">
<annotation>
<celldesigner:extension>
<celldesigner:alias>sa349</celldesigner:alias>
</celldesigner:extension>
</annotation>
</speciesReference>
</listOfProducts>
</reaction>
<reaction metaid="re87" id="re87" name="RPPPK" reversible="false">
<annotation>
<celldesigner:extension>
<celldesigner:name>RPPPK</celldesigner:name>
<celldesigner:reactionType>STATE_TRANSITION</celldesigner:reactionType>
<celldesigner:baseReactants>
<celldesigner:baseReactant species="s432" alias="sa349"/>
</celldesigner:baseReactants>

<celldesigner:baseProducts>
<celldesigner:baseProduct species="s433" alias="sa504">
<celldesigner:linkAnchor position="NNE"/>
</celldesigner:baseProduct>
</celldesigner:baseProducts>
<celldesigner:connectScheme connectPolicy="direct" rectangleIndex="0">
<celldesigner:listOfLineDirection>
<celldesigner:lineDirection index="0" value="unknown"/>
</celldesigner:listOfLineDirection>
</celldesigner:connectScheme>
<celldesigner:line width="1.0" color="ff00ffff"/>
</celldesigner:extension>
</annotation>
<listOfReactants>
<speciesReference metaid="CDMT00186" species="s432">
<annotation>
<celldesigner:extension>
<celldesigner:alias>sa349</celldesigner:alias>
</celldesigner:extension>
</annotation>
</speciesReference>
</listOfReactants>
<listOfProducts>
<speciesReference metaid="CDMT00188" species="s433">
<annotation>
<celldesigner:extension>
<celldesigner:alias>sa504</celldesigner:alias>

</celldesigner:extension>

</annotation>

</speciesReference>

</listOfProducts>

</reaction>

<reaction metaid="re88" id="re88" name="TK" reversible="false">

<annotation>

<celldesigner:extension>

<celldesigner:name>TK</celldesigner:name>

<celldesigner:reactionType>STATE_TRANSITION</celldesigner:reactionType>

<celldesigner:baseReactants>

<celldesigner:baseReactant species="s432" alias="sa349"/>

</celldesigner:baseReactants>

<celldesigner:baseProducts>

<celldesigner:baseProduct species="s21" alias="sa351"/>

</celldesigner:baseProducts>

<celldesigner:listOfReactantLinks>

<celldesigner:reactantLink reactant="s34" alias="sa353" targetLineIndex="-1,0">

<celldesigner:connectScheme connectPolicy="direct">

<celldesigner:listOfLineDirection>

<celldesigner:lineDirection index="0" value="unknown"/>

</celldesigner:listOfLineDirection>

</celldesigner:connectScheme>

<celldesigner:line width="1.0" color="ffff6633" type="Straight"/>

</celldesigner:reactantLink>

</celldesigner:listOfReactantLinks>

<celldesigner:connectScheme connectPolicy="direct" rectangleIndex="0">

<celldesigner:listOfLineDirection>

<celldesigner:lineDirection index="0" value="unknown"/>

</celldesigner:listOfLineDirection>

</celldesigner:connectScheme>

<celldesigner:line width="1.0" color="ffff6633"/>

</celldesigner:extension>

</annotation>

<listOfReactants>

<speciesReference metaid="CDMT00190" species="s432">

<annotation>

<celldesigner:extension>

<celldesigner:alias>sa349</celldesigner:alias>

</celldesigner:extension>

</annotation>

</speciesReference>

<speciesReference metaid="CDMT00191" species="s34">

<annotation>

<celldesigner:extension>

<celldesigner:alias>sa353</celldesigner:alias>

</celldesigner:extension>

</annotation>

</speciesReference>

</listOfReactants>

<listOfProducts>

<speciesReference metaid="CDMT00192" species="s21">

<annotation>

<celldesigner:extension>

<celldesigner:alias>sa351</celldesigner:alias>

</celldesigner:extension>

</annotation>

</speciesReference>

</listOfProducts>

</reaction>

<reaction metaid="re89" id="re89" reversible="false">

<annotation>

<celldesigner:extension>

<celldesigner:reactionType>STATE_TRANSITION</celldesigner:reactionType>

<celldesigner:baseReactants>

<celldesigner:baseReactant species="s23" alias="sa352"/>

</celldesigner:baseReactants>

<celldesigner:baseProducts>

<celldesigner:baseProduct species="s21" alias="sa351"/>

</celldesigner:baseProducts>

<celldesigner:connectScheme connectPolicy="direct" rectangleIndex="0">

<celldesigner:listOfLineDirection>

<celldesigner:lineDirection index="0" value="unknown"/>

</celldesigner:listOfLineDirection>

</celldesigner:connectScheme>

<celldesigner:line width="1.0" color="ffff6633"/>

</celldesigner:extension>

</annotation>

<listOfReactants>

<speciesReference metaid="CDMT00194" species="s23">

<annotation>

<celldesigner:extension>
<celldesigner:alias>sa352</celldesigner:alias>
</celldesigner:extension>
</annotation>
</speciesReference>
</listOfReactants>
<listOfProducts>
<speciesReference metaid="CDMT00195" species="s21">
<annotation>
<celldesigner:extension>
<celldesigner:alias>sa351</celldesigner:alias>
</celldesigner:extension>
</annotation>
</speciesReference>
</listOfProducts>
</reaction>
<reaction metaid="re90" id="re90" name="R5epi" reversible="false">
<annotation>
<celldesigner:extension>
<celldesigner:name>R5epi</celldesigner:name>
<celldesigner:reactionType>STATE_TRANSITION</celldesigner:reactionType>
<celldesigner:baseReactants>
<celldesigner:baseReactant species="s5" alias="sa333"/>
</celldesigner:baseReactants>
<celldesigner:baseProducts>
<celldesigner:baseProduct species="s34" alias="sa353"/>
</celldesigner:baseProducts>

<celldesigner:connectScheme connectPolicy="direct" rectangleIndex="0">

<celldesigner:listOfLineDirection>

<celldesigner:lineDirection index="0" value="unknown"/>

</celldesigner:listOfLineDirection>

</celldesigner:connectScheme>

<celldesigner:line width="1.0" color="ffff6633"/>

</celldesigner:extension>

</annotation>

<listOfReactants>

<speciesReference metaid="CDMT00196" species="s5">

<annotation>

<celldesigner:extension>

<celldesigner:alias>sa333</celldesigner:alias>

</celldesigner:extension>

</annotation>

</speciesReference>

</listOfReactants>

<listOfProducts>

<speciesReference metaid="CDMT00197" species="s34">

<annotation>

<celldesigner:extension>

<celldesigner:alias>sa353</celldesigner:alias>

</celldesigner:extension>

</annotation>

</speciesReference>

</listOfProducts>

</reaction>

<reaction metaid="re91" id="re91" name="TK1" reversible="false">

<annotation>

<celldesigner:extension>

<celldesigner:name>TK1</celldesigner:name>

<celldesigner:reactionType>STATE_TRANSITION</celldesigner:reactionType>

<celldesigner:baseReactants>

<celldesigner:baseReactant species="s34" alias="sa353"/>

</celldesigner:baseReactants>

<celldesigner:baseProducts>

<celldesigner:baseProduct species="s35" alias="sa354"/>

</celldesigner:baseProducts>

<celldesigner:listOfReactantLinks>

<celldesigner:reactantLink reactant="s23" alias="sa352" targetLineIndex="-1,0">

<celldesigner:connectScheme connectPolicy="direct">

<celldesigner:listOfLineDirection>

<celldesigner:lineDirection index="0" value="unknown"/>

</celldesigner:listOfLineDirection>

</celldesigner:connectScheme>

<celldesigner:line width="1.0" color="ffff6633" type="Straight"/>

</celldesigner:reactantLink>

</celldesigner:listOfReactantLinks>

<celldesigner:connectScheme connectPolicy="direct" rectangleIndex="0">

<celldesigner:listOfLineDirection>

<celldesigner:lineDirection index="0" value="unknown"/>

</celldesigner:listOfLineDirection>

</celldesigner:connectScheme>

<celldesigner:line width="1.0" color="ffff6633"/>

</celldesigner:extension>

</annotation>

<listOfReactants>

<speciesReference metaid="CDMT00198" species="s34">

<annotation>

<celldesigner:extension>

<celldesigner:alias>sa353</celldesigner:alias>

</celldesigner:extension>

</annotation>

</speciesReference>

<speciesReference metaid="CDMT00199" species="s23">

<annotation>

<celldesigner:extension>

<celldesigner:alias>sa352</celldesigner:alias>

</celldesigner:extension>

</annotation>

</speciesReference>

</listOfReactants>

<listOfProducts>

<speciesReference metaid="CDMT00200" species="s35">

<annotation>

<celldesigner:extension>

<celldesigner:alias>sa354</celldesigner:alias>

</celldesigner:extension>

</annotation>

</speciesReference>

</listOfProducts>

</reaction>
<reaction metaid="re92" id="re92" name="F6P-F16P" reversible="false">
<annotation>
<celldesigner:extension>
<celldesigner:name>F6P-F16P</celldesigner:name>
<celldesigner:reactionType>STATE_TRANSITION</celldesigner:reactionType>
<celldesigner:baseReactants>
<celldesigner:baseReactant species="s35" alias="sa354"/>
</celldesigner:baseReactants>
<celldesigner:baseProducts>
<celldesigner:baseProduct species="s36" alias="sa355"/>
</celldesigner:baseProducts>
<celldesigner:connectScheme connectPolicy="direct" rectangleIndex="0">
<celldesigner:listOfLineDirection>
<celldesigner:lineDirection index="0" value="unknown"/>
</celldesigner:listOfLineDirection>
</celldesigner:connectScheme>
<celldesigner:line width="1.0" color="ffff6633"/>
</celldesigner:extension>
</annotation>
<listOfReactants>
<speciesReference metaid="CDMT00202" species="s35">
<annotation>
<celldesigner:extension>
<celldesigner:alias>sa354</celldesigner:alias>
</celldesigner:extension>
</annotation>

</speciesReference>

</listOfReactants>

<listOfProducts>

<speciesReference metaid="CDMT00203" species="s36">

<annotation>

<celldesigner:extension>

<celldesigner:alias>sa355</celldesigner:alias>

</celldesigner:extension>

</annotation>

</speciesReference>

</listOfProducts>

</reaction>

<reaction metaid="re93" id="re93" name="TrAld" reversible="false">

<annotation>

<celldesigner:extension>

<celldesigner:name>TrAld</celldesigner:name>

<celldesigner:reactionType>STATE_TRANSITION</celldesigner:reactionType>

<celldesigner:baseReactants>

<celldesigner:baseReactant species="s35" alias="sa354"/>

</celldesigner:baseReactants>

<celldesigner:baseProducts>

<celldesigner:baseProduct species="s21" alias="sa351"/>

</celldesigner:baseProducts>

<celldesigner:listOfReactantLinks>

<celldesigner:reactantLink reactant="s23" alias="sa352" targetLineIndex="-1,0">

<celldesigner:connectScheme connectPolicy="direct">

<celldesigner:listOfLineDirection>

<celldesigner:lineDirection index="0" value="unknown"/>

</celldesigner:listOfLineDirection>

</celldesigner:connectScheme>

<celldesigner:line width="1.0" color="ff000000" type="Straight"/>

</celldesigner:reactantLink>

</celldesigner:listOfReactantLinks>

<celldesigner:connectScheme connectPolicy="direct" rectangleIndex="0">

<celldesigner:listOfLineDirection>

<celldesigner:lineDirection index="0" value="unknown"/>

</celldesigner:listOfLineDirection>

</celldesigner:connectScheme>

<celldesigner:line width="1.0" color="ffff6633"/>

</celldesigner:extension>

</annotation>

<listOfReactants>

<speciesReference metaid="CDMT00204" species="s35">

<annotation>

<celldesigner:extension>

<celldesigner:alias>sa354</celldesigner:alias>

</celldesigner:extension>

</annotation>

</speciesReference>

<speciesReference metaid="CDMT00205" species="s23">

<annotation>

<celldesigner:extension>

<celldesigner:alias>sa352</celldesigner:alias>

</celldesigner:extension>

</annotation>
</speciesReference>
</listOfReactants>
<listOfProducts>
<speciesReference metaid="CDMT00206" species="s21">
<annotation>
<celldesigner:extension>
<celldesigner:alias>sa351</celldesigner:alias>
</celldesigner:extension>
</annotation>
</speciesReference>
</listOfProducts>
</reaction>
<reaction metaid="re94" id="re94" reversible="false">
<annotation>
<celldesigner:extension>
<celldesigner:reactionType>STATE_TRANSITION</celldesigner:reactionType>
<celldesigner:baseReactants>
<celldesigner:baseReactant species="s434" alias="sa359"/>
</celldesigner:baseReactants>
<celldesigner:baseProducts>
<celldesigner:baseProduct species="s82" alias="sa19"/>
</celldesigner:baseProducts>
<celldesigner:connectScheme connectPolicy="direct" rectangleIndex="0">
<celldesigner:listOfLineDirection>
<celldesigner:lineDirection index="0" value="unknown"/>
</celldesigner:listOfLineDirection>

</celldesigner:connectScheme>

<celldesigner:line width="1.0" color="ff000000"/>

</celldesigner:extension>

</annotation>

<listOfReactants>

<speciesReference metaid="CDMT00112" species="s434">

<annotation>

<celldesigner:extension>

<celldesigner:alias>sa359</celldesigner:alias>

</celldesigner:extension>

</annotation>

</speciesReference>

</listOfReactants>

<listOfProducts>

<speciesReference metaid="CDMT00114" species="s82">

<annotation>

<celldesigner:extension>

<celldesigner:alias>sa19</celldesigner:alias>

</celldesigner:extension>

</annotation>

</speciesReference>

</listOfProducts>

</reaction>

<reaction metaid="re95" id="re95" reversible="false">

<annotation>

<celldesigner:extension>

<celldesigner:reactionType>STATE_TRANSITION</celldesigner:reactionType>

<celldesigner:baseReactants>

<celldesigner:baseReactant species="s435" alias="sa360"/>

</celldesigner:baseReactants>

<celldesigner:baseProducts>

<celldesigner:baseProduct species="s57" alias="sa22"/>

</celldesigner:baseProducts>

<celldesigner:connectScheme connectPolicy="direct" rectangleIndex="0">

<celldesigner:listOfLineDirection>

<celldesigner:lineDirection index="0" value="unknown"/>

</celldesigner:listOfLineDirection>

</celldesigner:connectScheme>

<celldesigner:line width="1.0" color="ffff6633"/>

</celldesigner:extension>

</annotation>

<listOfReactants>

<speciesReference metaid="CDMT00115" species="s435">

<annotation>

<celldesigner:extension>

<celldesigner:alias>sa360</celldesigner:alias>

</celldesigner:extension>

</annotation>

</speciesReference>

</listOfReactants>

<listOfProducts>

<speciesReference metaid="CDMT00116" species="s57">

<annotation>

<celldesigner:extension>

<celldesigner:alias>sa22</celldesigner:alias>

</celldesigner:extension>

</annotation>

</speciesReference>

</listOfProducts>

</reaction>

<reaction metaid="re267" id="re96" name="PEPCK" reversible="false">

<annotation>

<celldesigner:extension>

<celldesigner:name>PEPCK</celldesigner:name>

<celldesigner:reactionType>STATE_TRANSITION</celldesigner:reactionType>

<celldesigner:baseReactants>

<celldesigner:baseReactant species="s66" alias="sa108"/>

</celldesigner:baseReactants>

<celldesigner:baseProducts>

<celldesigner:baseProduct species="s13" alias="sa243"/>

</celldesigner:baseProducts>

<celldesigner:connectScheme connectPolicy="direct" rectangleIndex="1">

<celldesigner:listOfLineDirection>

<celldesigner:lineDirection index="0" value="unknown"/>

<celldesigner:lineDirection index="1" value="unknown"/>

</celldesigner:listOfLineDirection>

</celldesigner:connectScheme>

<celldesigner:editPoints>0.490779298036883,-0.5145746579417027</celldesigner:editPoints>

<celldesigner:line width="1.0" color="ffff6633"/>

</celldesigner:extension>

</annotation>

<listOfReactants>

<speciesReference metaid="CDMT00004" species="s66">

<annotation>

<celldesigner:extension>

<celldesigner:alias>sa108</celldesigner:alias>

</celldesigner:extension>

</annotation>

</speciesReference>

</listOfReactants>

<listOfProducts>

<speciesReference metaid="CDMT00005" species="s13">

<annotation>

<celldesigner:extension>

<celldesigner:alias>sa243</celldesigner:alias>

</celldesigner:extension>

</annotation>

</speciesReference>

</listOfProducts>

</reaction>

<reaction metaid="re97" id="re97" reversible="false">

<annotation>

<celldesigner:extension>

<celldesigner:reactionType>STATE_TRANSITION</celldesigner:reactionType>

<celldesigner:baseReactants>

<celldesigner:baseReactant species="s13" alias="sa243"/>

</celldesigner:baseReactants>

<celldesigner:baseProducts>

<celldesigner:baseProduct species="s33" alias="sa226"/>

</celldesigner:baseProducts>

<celldesigner:connectScheme connectPolicy="direct" rectangleIndex="0">

<celldesigner:listOfLineDirection>

<celldesigner:lineDirection index="0" value="unknown"/>

</celldesigner:listOfLineDirection>

</celldesigner:connectScheme>

<celldesigner:line width="1.0" color="ffff6633"/>

</celldesigner:extension>

</annotation>

<listOfReactants>

<speciesReference metaid="CDMT00007" species="s13">

<annotation>

<celldesigner:extension>

<celldesigner:alias>sa243</celldesigner:alias>

</celldesigner:extension>

</annotation>

</speciesReference>

</listOfReactants>

<listOfProducts>

<speciesReference metaid="CDMT00008" species="s33">

<annotation>

<celldesigner:extension>

<celldesigner:alias>sa226</celldesigner:alias>

</celldesigner:extension>

</annotation>

</speciesReference>

</listOfProducts>

</reaction>

<reaction metaid="re154" id="re118" name="HMGCoAS" reversible="false">

<annotation>

<celldesigner:extension>

<celldesigner:name>HMGCoAS</celldesigner:name>

<celldesigner:reactionType>STATE_TRANSITION</celldesigner:reactionType>

<celldesigner:baseReactants>

<celldesigner:baseReactant species="s7" alias="sa247">

<celldesigner:linkAnchor position="N"/>

</celldesigner:baseReactant>

</celldesigner:baseReactants>

<celldesigner:baseProducts>

<celldesigner:baseProduct species="s159" alias="sa387"/>

</celldesigner:baseProducts>

<celldesigner:listOfReactantLinks>

<celldesigner:reactantLink reactant="s459" alias="sa385" targetLineIndex="-1,0">

<celldesigner:connectScheme connectPolicy="direct">

<celldesigner:listOfLineDirection>

<celldesigner:lineDirection index="0" value="unknown"/>

</celldesigner:listOfLineDirection>

</celldesigner:connectScheme>

<celldesigner:line width="1.0" color="ff000000" type="Straight"/>

</celldesigner:reactantLink>

</celldesigner:listOfReactantLinks>

<celldesigner:connectScheme connectPolicy="direct" rectangleIndex="0">

<celldesigner:listOfLineDirection>

<celldesigner:lineDirection index="0" value="unknown"/>
</celldesigner:listOfLineDirection>
</celldesigner:connectScheme>
<celldesigner:line width="1.0" color="ff00ffff"/>
</celldesigner:extension>
</annotation>
<listOfReactants>
<speciesReference metaid="CDMT00002" species="s7">
<annotation>
<celldesigner:extension>
<celldesigner:alias>sa247</celldesigner:alias>
</celldesigner:extension>
</annotation>
</speciesReference>
<speciesReference metaid="CDMT00003" species="s459">
<annotation>
<celldesigner:extension>
<celldesigner:alias>sa385</celldesigner:alias>
</celldesigner:extension>
</annotation>
</speciesReference>
</listOfReactants>
<listOfProducts>
<speciesReference metaid="CDMT00010" species="s159">
<annotation>
<celldesigner:extension>
<celldesigner:alias>sa387</celldesigner:alias>

</celldesigner:extension>

</annotation>

</speciesReference>

</listOfProducts>

</reaction>

<reaction metaid="re155" id="re119" name="HMGCoARD" reversible="false">

<annotation>

<celldesigner:extension>

<celldesigner:name>HMGCoARD</celldesigner:name>

<celldesigner:reactionType>STATE_TRANSITION</celldesigner:reactionType>

<celldesigner:baseReactants>

<celldesigner:baseReactant species="s159" alias="sa387">

<celldesigner:linkAnchor position="S"/>

</celldesigner:baseReactant>

</celldesigner:baseReactants>

<celldesigner:baseProducts>

<celldesigner:baseProduct species="s461" alias="sa388"/>

</celldesigner:baseProducts>

<celldesigner:connectScheme connectPolicy="direct" rectangleIndex="0">

<celldesigner:listOfLineDirection>

<celldesigner:lineDirection index="0" value="unknown"/>

</celldesigner:listOfLineDirection>

</celldesigner:connectScheme>

<celldesigner:line width="1.0" color="ff000000"/>

</celldesigner:extension>

</annotation>

<listOfReactants>

<speciesReference metaid="CDMT00013" species="s159">

<annotation>

<celldesigner:extension>

<celldesigner:alias>sa387</celldesigner:alias>

</celldesigner:extension>

</annotation>

</speciesReference>

</listOfReactants>

<listOfProducts>

<speciesReference metaid="CDMT00016" species="s461">

<annotation>

<celldesigner:extension>

<celldesigner:alias>sa388</celldesigner:alias>

</celldesigner:extension>

</annotation>

</speciesReference>

</listOfProducts>

</reaction>

<reaction metaid="re156" id="re120" name="MEVK" reversible="false">

<annotation>

<celldesigner:extension>

<celldesigner:name>MEVK</celldesigner:name>

<celldesigner:reactionType>STATE_TRANSITION</celldesigner:reactionType>

<celldesigner:baseReactants>

<celldesigner:baseReactant species="s461" alias="sa388">

<celldesigner:linkAnchor position="S"/>

</celldesigner:baseReactant>

</celldesigner:baseReactants>

<celldesigner:baseProducts>

<celldesigner:baseProduct species="s163" alias="sa389"/>

</celldesigner:baseProducts>

<celldesigner:connectScheme connectPolicy="direct" rectangleIndex="0">

<celldesigner:listOfLineDirection>

<celldesigner:lineDirection index="0" value="unknown"/>

</celldesigner:listOfLineDirection>

</celldesigner:connectScheme>

<celldesigner:line width="1.0" color="ff000000"/>

</celldesigner:extension>

</annotation>

<listOfReactants>

<speciesReference metaid="CDMT00018" species="s461">

<annotation>

<celldesigner:extension>

<celldesigner:alias>sa388</celldesigner:alias>

</celldesigner:extension>

</annotation>

</speciesReference>

</listOfReactants>

<listOfProducts>

<speciesReference metaid="CDMT00021" species="s163">

<annotation>

<celldesigner:extension>

<celldesigner:alias>sa389</celldesigner:alias>

</celldesigner:extension>

</annotation>

</speciesReference>

</listOfProducts>

</reaction>

<reaction metaid="re157" id="re121" name="PMEVK" reversible="false">

<annotation>

<celldesigner:extension>

<celldesigner:name>PMEVK</celldesigner:name>

<celldesigner:reactionType>STATE_TRANSITION</celldesigner:reactionType>

<celldesigner:baseReactants>

<celldesigner:baseReactant species="s163" alias="sa389">

<celldesigner:linkAnchor position="S"/>

</celldesigner:baseReactant>

</celldesigner:baseReactants>

<celldesigner:baseProducts>

<celldesigner:baseProduct species="s462" alias="sa390"/>

</celldesigner:baseProducts>

<celldesigner:connectScheme connectPolicy="direct" rectangleIndex="0">

<celldesigner:listOfLineDirection>

<celldesigner:lineDirection index="0" value="unknown"/>

</celldesigner:listOfLineDirection>

</celldesigner:connectScheme>

<celldesigner:line width="1.0" color="ff000000"/>

</celldesigner:extension>

</annotation>

<listOfReactants>

<speciesReference metaid="CDMT00023" species="s163">

<annotation>
<celldesigner:extension>
<celldesigner:alias>sa389</celldesigner:alias>
</celldesigner:extension>
</annotation>
</speciesReference>
</listOfReactants>
<listOfProducts>
<speciesReference metaid="CDMT00024" species="s462">
<annotation>
<celldesigner:extension>
<celldesigner:alias>sa390</celldesigner:alias>
</celldesigner:extension>
</annotation>
</speciesReference>
</listOfProducts>
</reaction>
<reaction metaid="re158" id="re122" name="DPMEVDC" reversible="false">
<annotation>
<celldesigner:extension>
<celldesigner:name>DPMEVDC</celldesigner:name>
<celldesigner:reactionType>STATE_TRANSITION</celldesigner:reactionType>
<celldesigner:baseReactants>
<celldesigner:baseReactant species="s462" alias="sa390"/>
</celldesigner:baseReactants>
<celldesigner:baseProducts>
<celldesigner:baseProduct species="s165" alias="sa391"/>

</celldesigner:baseProducts>

<celldesigner:connectScheme connectPolicy="direct" rectangleIndex="0">

<celldesigner:listOfLineDirection>

<celldesigner:lineDirection index="0" value="unknown"/>

</celldesigner:listOfLineDirection>

</celldesigner:connectScheme>

<celldesigner:line width="1.0" color="ff000000"/>

</celldesigner:extension>

</annotation>

<listOfReactants>

<speciesReference metaid="CDMT00026" species="s462">

<annotation>

<celldesigner:extension>

<celldesigner:alias>sa390</celldesigner:alias>

</celldesigner:extension>

</annotation>

</speciesReference>

</listOfReactants>

<listOfProducts>

<speciesReference metaid="CDMT00027" species="s165">

<annotation>

<celldesigner:extension>

<celldesigner:alias>sa391</celldesigner:alias>

</celldesigner:extension>

</annotation>

</speciesReference>

</listOfProducts>

</reaction>

<reaction metaid="re159" id="re123" name="IPDPI" reversible="false">

<annotation>

<celldesigner:extension>

<celldesigner:name>IPDPI</celldesigner:name>

<celldesigner:reactionType>STATE_TRANSITION</celldesigner:reactionType>

<celldesigner:baseReactants>

<celldesigner:baseReactant species="s165" alias="sa391"/>

</celldesigner:baseReactants>

<celldesigner:baseProducts>

<celldesigner:baseProduct species="s166" alias="sa401"/>

</celldesigner:baseProducts>

<celldesigner:connectScheme connectPolicy="direct" rectangleIndex="0">

<celldesigner:listOfLineDirection>

<celldesigner:lineDirection index="0" value="unknown"/>

</celldesigner:listOfLineDirection>

</celldesigner:connectScheme>

<celldesigner:line width="1.0" color="ffff0033"/>

</celldesigner:extension>

</annotation>

<listOfReactants>

<speciesReference metaid="CDMT00029" species="s165">

<annotation>

<celldesigner:extension>

<celldesigner:alias>sa391</celldesigner:alias>

</celldesigner:extension>

</annotation>

</speciesReference>

</listOfReactants>

<listOfProducts>

<speciesReference metaid="CDMT00030" species="s166">

<annotation>

<celldesigner:extension>

<celldesigner:alias>sa401</celldesigner:alias>

</celldesigner:extension>

</annotation>

</speciesReference>

</listOfProducts>

</reaction>

<reaction metaid="re160" id="re124" name="DMALT" reversible="false">

<annotation>

<celldesigner:extension>

<celldesigner:name>DMALT</celldesigner:name>

<celldesigner:reactionType>STATE_TRANSITION</celldesigner:reactionType>

<celldesigner:baseReactants>

<celldesigner:baseReactant species="s166" alias="sa401"/>

</celldesigner:baseReactants>

<celldesigner:baseProducts>

<celldesigner:baseProduct species="s167" alias="sa402"/>

</celldesigner:baseProducts>

<celldesigner:listOfReactantLinks>

<celldesigner:reactantLink reactant="s165" alias="sa391" targetLineIndex="-1,0">

<celldesigner:linkAnchor position="SW"/>

<celldesigner:connectScheme connectPolicy="direct">

<celldesigner:listOfLineDirection>
<celldesigner:lineDirection index="0" value="unknown"/>
<celldesigner:lineDirection index="1" value="unknown"/>
</celldesigner:listOfLineDirection>
</celldesigner:connectScheme>
<celldesigner:editPoints>0.8379976201573793,0.399169622472598</celldesigner:editPoints>
<celldesigner:line width="1.0" color="ffff0033" type="Straight"/>
</celldesigner:reactantLink>
</celldesigner:listOfReactantLinks>
<celldesigner:connectScheme connectPolicy="direct" rectangleIndex="0">
<celldesigner:listOfLineDirection>
<celldesigner:lineDirection index="0" value="unknown"/>
</celldesigner:listOfLineDirection>
</celldesigner:connectScheme>
<celldesigner:line width="1.0" color="ffff0033"/>
</celldesigner:extension>
</annotation>
<listOfReactants>
<speciesReference metaid="CDMT00031" species="s166">
<annotation>
<celldesigner:extension>
<celldesigner:alias>sa401</celldesigner:alias>
</celldesigner:extension>
</annotation>
</speciesReference>
<speciesReference metaid="CDMT00033" species="s165">
<annotation>

<celldesigner:extension>
<celldesigner:alias>sa391</celldesigner:alias>
</celldesigner:extension>
</annotation>
</speciesReference>
</listOfReactants>
<listOfProducts>
<speciesReference metaid="CDMT00034" species="s167">
<annotation>
<celldesigner:extension>
<celldesigner:alias>sa402</celldesigner:alias>
</celldesigner:extension>
</annotation>
</speciesReference>
</listOfProducts>
</reaction>
<reaction metaid="re161" id="re125" name="GeranylT" reversible="false">
<annotation>
<celldesigner:extension>
<celldesigner:name>GeranylT</celldesigner:name>
<celldesigner:reactionType>STATE_TRANSITION</celldesigner:reactionType>
<celldesigner:baseReactants>
<celldesigner:baseReactant species="s167" alias="sa402">
<celldesigner:linkAnchor position="S"/>
</celldesigner:baseReactant>
</celldesigner:baseReactants>
<celldesigner:baseProducts>

<celldesigner:baseProduct species="s168" alias="sa403">

<celldesigner:linkAnchor position="N"/>

</celldesigner:baseProduct>

</celldesigner:baseProducts>

<celldesigner:listOfReactantLinks>

<celldesigner:reactantLink reactant="s165" alias="sa391" targetLineIndex="-1,0">

<celldesigner:linkAnchor position="WSW"/>

<celldesigner:connectScheme connectPolicy="direct">

<celldesigner:listOfLineDirection>

<celldesigner:lineDirection index="0" value="unknown"/>

<celldesigner:lineDirection index="1" value="unknown"/>

</celldesigner:listOfLineDirection>

</celldesigner:connectScheme>

<celldesigner:editPoints>0.8593251865726055,0.33663108489440885</celldesigner:editPoints>

<celldesigner:line width="1.0" color="ffff0033" type="Straight"/>

</celldesigner:reactantLink>

</celldesigner:listOfReactantLinks>

<celldesigner:connectScheme connectPolicy="direct" rectangleIndex="0">

<celldesigner:listOfLineDirection>

<celldesigner:lineDirection index="0" value="unknown"/>

</celldesigner:listOfLineDirection>

</celldesigner:connectScheme>

<celldesigner:line width="1.0" color="ffff0033"/>

</celldesigner:extension>

</annotation>

<listOfReactants>

<speciesReference metaid="CDMT00036" species="s167">

<annotation>
<celldesigner:extension>
<celldesigner:alias>sa402</celldesigner:alias>
</celldesigner:extension>
</annotation>
</speciesReference>
<speciesReference metaid="CDMT00038" species="s165">
<annotation>
<celldesigner:extension>
<celldesigner:alias>sa391</celldesigner:alias>
</celldesigner:extension>
</annotation>
</speciesReference>
</listOfReactants>
<listOfProducts>
<speciesReference metaid="CDMT00040" species="s168">
<annotation>
<celldesigner:extension>
<celldesigner:alias>sa403</celldesigner:alias>
</celldesigner:extension>
</annotation>
</speciesReference>
</listOfProducts>
</reaction>
<reaction metaid="re162" id="re126" name="SqlS" reversible="false">
<annotation>
<celldesigner:extension>

<celldesigner:name>SqlS</celldesigner:name>

<celldesigner:reactionType>STATE_TRANSITION</celldesigner:reactionType>

<celldesigner:baseReactants>

<celldesigner:baseReactant species="s168" alias="sa403"/>

</celldesigner:baseReactants>

<celldesigner:baseProducts>

<celldesigner:baseProduct species="s169" alias="sa404"/>

</celldesigner:baseProducts>

<celldesigner:connectScheme connectPolicy="direct" rectangleIndex="0">

<celldesigner:listOfLineDirection>

<celldesigner:lineDirection index="0" value="unknown"/>

</celldesigner:listOfLineDirection>

</celldesigner:connectScheme>

<celldesigner:line width="1.0" color="ff000000"/>

</celldesigner:extension>

</annotation>

<listOfReactants>

<speciesReference metaid="CDMT00041" species="s168">

<annotation>

<celldesigner:extension>

<celldesigner:alias>sa403</celldesigner:alias>

</celldesigner:extension>

</annotation>

</speciesReference>

</listOfReactants>

<listOfProducts>

<speciesReference metaid="CDMT00042" species="s169">

<annotation>

<celldesigner:extension>

<celldesigner:alias>sa404</celldesigner:alias>

</celldesigner:extension>

</annotation>

</speciesReference>

</listOfProducts>

</reaction>

<reaction metaid="re163" id="re127" name="SqlEpx" reversible="false">

<annotation>

<celldesigner:extension>

<celldesigner:name>SqlEpx</celldesigner:name>

<celldesigner:reactionType>STATE_TRANSITION</celldesigner:reactionType>

<celldesigner:baseReactants>

<celldesigner:baseReactant species="s169" alias="sa404">

<celldesigner:linkAnchor position="S"/>

</celldesigner:baseReactant>

</celldesigner:baseReactants>

<celldesigner:baseProducts>

<celldesigner:baseProduct species="s466" alias="sa405"/>

</celldesigner:baseProducts>

<celldesigner:connectScheme connectPolicy="direct" rectangleIndex="0">

<celldesigner:listOfLineDirection>

<celldesigner:lineDirection index="0" value="unknown"/>

</celldesigner:listOfLineDirection>

</celldesigner:connectScheme>

<celldesigner:line width="1.0" color="ff000000"/>

</celldesigner:extension>

</annotation>

<listOfReactants>

<speciesReference metaid="CDMT00043" species="s169">

<annotation>

<celldesigner:extension>

<celldesigner:alias>sa404</celldesigner:alias>

</celldesigner:extension>

</annotation>

</speciesReference>

</listOfReactants>

<listOfProducts>

<speciesReference metaid="CDMT00044" species="s466">

<annotation>

<celldesigner:extension>

<celldesigner:alias>sa405</celldesigner:alias>

</celldesigner:extension>

</annotation>

</speciesReference>

</listOfProducts>

</reaction>

<reaction metaid="re165" id="re128" name="LanoS" reversible="false">

<annotation>

<celldesigner:extension>

<celldesigner:name>LanoS</celldesigner:name>

<celldesigner:reactionType>STATE_TRANSITION</celldesigner:reactionType>

<celldesigner:baseReactants>

<celldesigner:baseReactant species="s466" alias="sa405">

<celldesigner:linkAnchor position="S"/>

</celldesigner:baseReactant>

</celldesigner:baseReactants>

<celldesigner:baseProducts>

<celldesigner:baseProduct species="s172" alias="sa393">

<celldesigner:linkAnchor position="N"/>

</celldesigner:baseProduct>

</celldesigner:baseProducts>

<celldesigner:connectScheme connectPolicy="direct" rectangleIndex="0">

<celldesigner:listOfLineDirection>

<celldesigner:lineDirection index="0" value="unknown"/>

</celldesigner:listOfLineDirection>

</celldesigner:connectScheme>

<celldesigner:line width="1.0" color="ff000000"/>

</celldesigner:extension>

</annotation>

<listOfReactants>

<speciesReference metaid="CDMT00045" species="s466">

<annotation>

<celldesigner:extension>

<celldesigner:alias>sa405</celldesigner:alias>

</celldesigner:extension>

</annotation>

</speciesReference>

</listOfReactants>

<listOfProducts>

<speciesReference metaid="CDMT00047" species="s172">

<annotation>

<celldesigner:extension>

<celldesigner:alias>sa393</celldesigner:alias>

</celldesigner:extension>

</annotation>

</speciesReference>

</listOfProducts>

</reaction>

<reaction metaid="re166" id="re129" name="Str14DM" reversible="false">

<annotation>

<celldesigner:extension>

<celldesigner:name>Str14DM</celldesigner:name>

<celldesigner:reactionType>STATE_TRANSITION</celldesigner:reactionType>

<celldesigner:baseReactants>

<celldesigner:baseReactant species="s172" alias="sa393">

<celldesigner:linkAnchor position="S"/>

</celldesigner:baseReactant>

</celldesigner:baseReactants>

<celldesigner:baseProducts>

<celldesigner:baseProduct species="s173" alias="sa394"/>

</celldesigner:baseProducts>

<celldesigner:listOfProductLinks>

<celldesigner:productLink product="s476" alias="sa392" targetLineIndex="-1,1">

<celldesigner:linkAnchor position="WSW"/>

<celldesigner:connectScheme connectPolicy="direct">

<celldesigner:listOfLineDirection>

<celldesigner:lineDirection index="0" value="unknown"/>

</celldesigner:listOfLineDirection>

</celldesigner:connectScheme>

<celldesigner:line width="1.0" color="ff000000" type="Straight"/>

</celldesigner:productLink>

</celldesigner:listOfProductLinks>

<celldesigner:connectScheme connectPolicy="direct" rectangleIndex="0">

<celldesigner:listOfLineDirection>

<celldesigner:lineDirection index="0" value="unknown"/>

</celldesigner:listOfLineDirection>

</celldesigner:connectScheme>

<celldesigner:line width="1.0" color="ff000000"/>

</celldesigner:extension>

</annotation>

<listOfReactants>

<speciesReference metaid="CDMT00049" species="s172">

<annotation>

<celldesigner:extension>

<celldesigner:alias>sa393</celldesigner:alias>

</celldesigner:extension>

</annotation>

</speciesReference>

</listOfReactants>

<listOfProducts>

<speciesReference metaid="CDMT00050" species="s173">

<annotation>

<celldesigner:extension>

<celldesigner:alias>sa394</celldesigner:alias>

</celldesigner:extension>

</annotation>

</speciesReference>

<speciesReference metaid="CDMT00051" species="s476">

<annotation>

<celldesigner:extension>

<celldesigner:alias>sa392</celldesigner:alias>

</celldesigner:extension>

</annotation>

</speciesReference>

</listOfProducts>

</reaction>

<reaction metaid="re167" id="re130" name="14StrRD" reversible="false">

<annotation>

<celldesigner:extension>

<celldesigner:name>14StrRD</celldesigner:name>

<celldesigner:reactionType>STATE_TRANSITION</celldesigner:reactionType>

<celldesigner:baseReactants>

<celldesigner:baseReactant species="s173" alias="sa394"/>

</celldesigner:baseReactants>

<celldesigner:baseProducts>

<celldesigner:baseProduct species="s174" alias="sa395"/>

</celldesigner:baseProducts>

<celldesigner:connectScheme connectPolicy="direct" rectangleIndex="0">

<celldesigner:listOfLineDirection>

<celldesigner:lineDirection index="0" value="unknown"/>

</celldesigner:listOfLineDirection>

</celldesigner:connectScheme>

<celldesigner:line width="1.0" color="ff000000"/>

</celldesigner:extension>

</annotation>

<listOfReactants>

<speciesReference metaid="CDMT00052" species="s173">

<annotation>

<celldesigner:extension>

<celldesigner:alias>sa394</celldesigner:alias>

</celldesigner:extension>

</annotation>

</speciesReference>

</listOfReactants>

<listOfProducts>

<speciesReference metaid="CDMT00053" species="s174">

<annotation>

<celldesigner:extension>

<celldesigner:alias>sa395</celldesigner:alias>

</celldesigner:extension>

</annotation>

</speciesReference>

</listOfProducts>

</reaction>

<reaction metaid="re168" id="re131" name="KetoSRD" reversible="false">

<annotation>

<celldesigner:extension>

<celldesigner:name>KetoSRD</celldesigner:name>

<celldesigner:reactionType>STATE_TRANSITION</celldesigner:reactionType>

<celldesigner:baseReactants>

<celldesigner:baseReactant species="s174" alias="sa395">

<celldesigner:linkAnchor position="S"/>

</celldesigner:baseReactant>

</celldesigner:baseReactants>

<celldesigner:baseProducts>

<celldesigner:baseProduct species="s1099" alias="sa396"/>

</celldesigner:baseProducts>

<celldesigner:connectScheme connectPolicy="direct" rectangleIndex="0">

<celldesigner:listOfLineDirection>

<celldesigner:lineDirection index="0" value="unknown"/>

</celldesigner:listOfLineDirection>

</celldesigner:connectScheme>

<celldesigner:line width="1.0" color="ff000000"/>

</celldesigner:extension>

</annotation>

<listOfReactants>

<speciesReference metaid="CDMT00055" species="s174">

<annotation>

<celldesigner:extension>

<celldesigner:alias>sa395</celldesigner:alias>

</celldesigner:extension>

</annotation>

</speciesReference>

</listOfReactants>

<listOfProducts>
<speciesReference metaid="CDMT00057" species="s1099">
<annotation>
<celldesigner:extension>
<celldesigner:alias>sa396</celldesigner:alias>
</celldesigner:extension>
</annotation>
</speciesReference>
</listOfProducts>
</reaction>
<reaction metaid="re169" id="re132" name="ERG6" reversible="false">
<annotation>
<celldesigner:extension>
<celldesigner:name>ERG6</celldesigner:name>
<celldesigner:reactionType>STATE_TRANSITION</celldesigner:reactionType>
<celldesigner:baseReactants>
<celldesigner:baseReactant species="s1099" alias="sa396">
<celldesigner:linkAnchor position="S"/>
</celldesigner:baseReactant>
</celldesigner:baseReactants>
<celldesigner:baseProducts>
<celldesigner:baseProduct species="s463" alias="sa397">
<celldesigner:linkAnchor position="N"/>
</celldesigner:baseProduct>
</celldesigner:baseProducts>
<celldesigner:connectScheme connectPolicy="direct" rectangleIndex="0">
<celldesigner:listOfLineDirection>

<celldesigner:lineDirection index="0" value="unknown"/>

</celldesigner:listOfLineDirection>

</celldesigner:connectScheme>

<celldesigner:line width="1.0" color="ff000000"/>

</celldesigner:extension>

</annotation>

<listOfReactants>

<speciesReference metaid="CDMT00058" species="s1099">

<annotation>

<celldesigner:extension>

<celldesigner:alias>sa396</celldesigner:alias>

</celldesigner:extension>

</annotation>

</speciesReference>

</listOfReactants>

<listOfProducts>

<speciesReference metaid="CDMT00062" species="s463">

<annotation>

<celldesigner:extension>

<celldesigner:alias>sa397</celldesigner:alias>

</celldesigner:extension>

</annotation>

</speciesReference>

</listOfProducts>

</reaction>

<reaction metaid="re170" id="re133" name="ERG2" reversible="false">

<annotation>

<celldesigner:extension>

<celldesigner:name>ERG2</celldesigner:name>

<celldesigner:reactionType>STATE_TRANSITION</celldesigner:reactionType>

<celldesigner:baseReactants>

<celldesigner:baseReactant species="s463" alias="sa397"/>

</celldesigner:baseReactants>

<celldesigner:baseProducts>

<celldesigner:baseProduct species="s467" alias="sa406"/>

</celldesigner:baseProducts>

<celldesigner:connectScheme connectPolicy="direct" rectangleIndex="0">

<celldesigner:listOfLineDirection>

<celldesigner:lineDirection index="0" value="unknown"/>

</celldesigner:listOfLineDirection>

</celldesigner:connectScheme>

<celldesigner:line width="1.0" color="ff000000"/>

</celldesigner:extension>

</annotation>

<listOfReactants>

<speciesReference metaid="CDMT00063" species="s463">

<annotation>

<celldesigner:extension>

<celldesigner:alias>sa397</celldesigner:alias>

</celldesigner:extension>

</annotation>

</speciesReference>

</listOfReactants>

<listOfProducts>

<speciesReference metaid="CDMT00064" species="s467">
<annotation>
<celldesigner:extension>
<celldesigner:alias>sa406</celldesigner:alias>
</celldesigner:extension>
</annotation>
</speciesReference>
</listOfProducts>
</reaction>
<reaction metaid="re171" id="re134" name="ERG3" reversible="false">
<annotation>
<celldesigner:extension>
<celldesigner:name>ERG3</celldesigner:name>
<celldesigner:reactionType>STATE_TRANSITION</celldesigner:reactionType>
<celldesigner:baseReactants>
<celldesigner:baseReactant species="s467" alias="sa406"/>
</celldesigner:baseReactants>
<celldesigner:baseProducts>
<celldesigner:baseProduct species="s178" alias="sa398"/>
</celldesigner:baseProducts>
<celldesigner:connectScheme connectPolicy="direct" rectangleIndex="0">
<celldesigner:listOfLineDirection>
<celldesigner:lineDirection index="0" value="unknown"/>
</celldesigner:listOfLineDirection>
</celldesigner:connectScheme>
<celldesigner:line width="1.0" color="ff000000"/>
</celldesigner:extension>

</annotation>

<listOfReactants>

<speciesReference metaid="CDMT00065" species="s467">

<annotation>

<celldesigner:extension>

<celldesigner:alias>sa406</celldesigner:alias>

</celldesigner:extension>

</annotation>

</speciesReference>

</listOfReactants>

<listOfProducts>

<speciesReference metaid="CDMT00067" species="s178">

<annotation>

<celldesigner:extension>

<celldesigner:alias>sa398</celldesigner:alias>

</celldesigner:extension>

</annotation>

</speciesReference>

</listOfProducts>

</reaction>

<reaction metaid="re172" id="re135" name="ERG5" reversible="false">

<annotation>

<celldesigner:extension>

<celldesigner:name>ERG5</celldesigner:name>

<celldesigner:reactionType>STATE_TRANSITION</celldesigner:reactionType>

<celldesigner:baseReactants>

<celldesigner:baseReactant species="s178" alias="sa398"/>

</celldesigner:baseReactants>

<celldesigner:baseProducts>

<celldesigner:baseProduct species="s464" alias="sa399"/>

</celldesigner:baseProducts>

<celldesigner:connectScheme connectPolicy="direct" rectangleIndex="0">

<celldesigner:listOfLineDirection>

<celldesigner:lineDirection index="0" value="unknown"/>

</celldesigner:listOfLineDirection>

</celldesigner:connectScheme>

<celldesigner:line width="1.0" color="ff000000"/>

</celldesigner:extension>

</annotation>

<listOfReactants>

<speciesReference metaid="CDMT00069" species="s178">

<annotation>

<celldesigner:extension>

<celldesigner:alias>sa398</celldesigner:alias>

</celldesigner:extension>

</annotation>

</speciesReference>

</listOfReactants>

<listOfProducts>

<speciesReference metaid="CDMT00070" species="s464">

<annotation>

<celldesigner:extension>

<celldesigner:alias>sa399</celldesigner:alias>

</celldesigner:extension>

</annotation>
</speciesReference>
</listOfProducts>
</reaction>
<reaction metaid="re173" id="re136" name="ERG4" reversible="false">
<annotation>
<celldesigner:extension>
<celldesigner:name>ERG4</celldesigner:name>
<celldesigner:reactionType>STATE_TRANSITION</celldesigner:reactionType>
<celldesigner:baseReactants>
<celldesigner:baseReactant species="s464" alias="sa399"/>
</celldesigner:baseReactants>
<celldesigner:baseProducts>
<celldesigner:baseProduct species="s465" alias="sa400"/>
</celldesigner:baseProducts>
<celldesigner:connectScheme connectPolicy="direct" rectangleIndex="0">
<celldesigner:listOfLineDirection>
<celldesigner:lineDirection index="0" value="unknown"/>
</celldesigner:listOfLineDirection>
</celldesigner:connectScheme>
<celldesigner:line width="1.0" color="ff000000"/>
</celldesigner:extension>
</annotation>
<listOfReactants>
<speciesReference metaid="CDMT00071" species="s464">
<annotation>
<celldesigner:extension>

<celldesigner:alias>sa399</celldesigner:alias>

</celldesigner:extension>

</annotation>

</speciesReference>

</listOfReactants>

<listOfProducts>

<speciesReference metaid="CDMT00072" species="s465">

<annotation>

<celldesigner:extension>

<celldesigner:alias>sa400</celldesigner:alias>

</celldesigner:extension>

</annotation>

</speciesReference>

</listOfProducts>

</reaction>

<reaction metaid="re433" id="re137" name="methylglutaconyl-CoA hydratase" reversible="false">

<annotation>

<celldesigner:extension>

<celldesigner:name>methylglutaconyl-CoA hydratase</celldesigner:name>

<celldesigner:reactionType>STATE_TRANSITION</celldesigner:reactionType>

<celldesigner:baseReactants>

<celldesigner:baseReactant species="s456" alias="sa384"/>

</celldesigner:baseReactants>

<celldesigner:baseProducts>

<celldesigner:baseProduct species="s159" alias="sa387">

<celldesigner:linkAnchor position="NNE"/>

</celldesigner:baseProduct>

</celldesigner:baseProducts>

<celldesigner:connectScheme connectPolicy="direct" rectangleIndex="0">

<celldesigner:listOfLineDirection>

<celldesigner:lineDirection index="0" value="unknown"/>

</celldesigner:listOfLineDirection>

</celldesigner:connectScheme>

<celldesigner:line width="1.0" color="ff000000"/>

</celldesigner:extension>

</annotation>

<listOfReactants>

<speciesReference metaid="CDMT00073" species="s456">

<annotation>

<celldesigner:extension>

<celldesigner:alias>sa384</celldesigner:alias>

</celldesigner:extension>

</annotation>

</speciesReference>

</listOfReactants>

<listOfProducts>

<speciesReference metaid="CDMT00075" species="s159">

<annotation>

<celldesigner:extension>

<celldesigner:alias>sa387</celldesigner:alias>

</celldesigner:extension>

</annotation>

</speciesReference>

</listOfProducts>

</reaction>
<reaction metaid="re101" id="re138" name="AcT" reversible="false">
<annotation>
<celldesigner:extension>
<celldesigner:name>AcT</celldesigner:name>
<celldesigner:reactionType>STATE_TRANSITION</celldesigner:reactionType>
<celldesigner:baseReactants>
<celldesigner:baseReactant species="s175" alias="sa411">
<celldesigner:linkAnchor position="S"/>
</celldesigner:baseReactant>
</celldesigner:baseReactants>
<celldesigner:baseProducts>
<celldesigner:baseProduct species="s176" alias="sa415"/>
</celldesigner:baseProducts>
<celldesigner:listOfReactantLinks>
<celldesigner:reactantLink reactant="s1101" alias="sa417" targetLineIndex="-1,0">
<celldesigner:connectScheme connectPolicy="direct">
<celldesigner:listOfLineDirection>
<celldesigner:lineDirection index="0" value="unknown"/>
</celldesigner:listOfLineDirection>
</celldesigner:connectScheme>
<celldesigner:line width="1.0" color="ffffcc00" type="Straight"/>
</celldesigner:reactantLink>
</celldesigner:listOfReactantLinks>
<celldesigner:connectScheme connectPolicy="direct" rectangleIndex="0">
<celldesigner:listOfLineDirection>
<celldesigner:lineDirection index="0" value="unknown"/>

</celldesigner:listOfLineDirection>

</celldesigner:connectScheme>

<celldesigner:line width="1.0" color="ffffcc00"/>

</celldesigner:extension>

</annotation>

<listOfReactants>

<speciesReference metaid="CDMT00077" species="s175">

<annotation>

<celldesigner:extension>

<celldesigner:alias>sa411</celldesigner:alias>

</celldesigner:extension>

</annotation>

</speciesReference>

<speciesReference metaid="CDMT00079" species="s1101">

<annotation>

<celldesigner:extension>

<celldesigner:alias>sa417</celldesigner:alias>

</celldesigner:extension>

</annotation>

</speciesReference>

</listOfReactants>

<listOfProducts>

<speciesReference metaid="CDMT00081" species="s176">

<annotation>

<celldesigner:extension>

<celldesigner:alias>sa415</celldesigner:alias>

</celldesigner:extension>

</annotation>
</speciesReference>
</listOfProducts>
</reaction>
<reaction metaid="re103" id="re139" name="PaP" reversible="false">
<annotation>
<celldesigner:extension>
<celldesigner:name>PaP</celldesigner:name>
<celldesigner:reactionType>STATE_TRANSITION</celldesigner:reactionType>
<celldesigner:baseReactants>
<celldesigner:baseReactant species="s176" alias="sa415">
<celldesigner:linkAnchor position="S"/>
</celldesigner:baseReactant>
</celldesigner:baseReactants>
<celldesigner:baseProducts>
<celldesigner:baseProduct species="s177" alias="sa420">
<celldesigner:linkAnchor position="N"/>
</celldesigner:baseProduct>
</celldesigner:baseProducts>
<celldesigner:connectScheme connectPolicy="direct" rectangleIndex="0">
<celldesigner:listOfLineDirection>
<celldesigner:lineDirection index="0" value="unknown"/>
</celldesigner:listOfLineDirection>
</celldesigner:connectScheme>
<celldesigner:line width="1.0" color="ffffcc00"/>
</celldesigner:extension>
</annotation>

<listOfReactants>

<speciesReference metaid="CDMT00082" species="s176">

<annotation>

<celldesigner:extension>

<celldesigner:alias>sa415</celldesigner:alias>

</celldesigner:extension>

</annotation>

</speciesReference>

</listOfReactants>

<listOfProducts>

<speciesReference metaid="CDMT00083" species="s177">

<annotation>

<celldesigner:extension>

<celldesigner:alias>sa420</celldesigner:alias>

</celldesigner:extension>

</annotation>

</speciesReference>

</listOfProducts>

</reaction>

<reaction metaid="re218" id="re140" name="DGK_Lumped" reversible="false">

<annotation>

<celldesigner:extension>

<celldesigner:name>DGK_Lumped</celldesigner:name>

<celldesigner:reactionType>STATE_TRANSITION</celldesigner:reactionType>

<celldesigner:baseReactants>

<celldesigner:baseReactant species="s177" alias="sa420">

<celldesigner:linkAnchor position="NNE"/>

</celldesigner:baseReactant>

</celldesigner:baseReactants>

<celldesigner:baseProducts>

<celldesigner:baseProduct species="s176" alias="sa415">

<celldesigner:linkAnchor position="SSE"/>

</celldesigner:baseProduct>

</celldesigner:baseProducts>

<celldesigner:connectScheme connectPolicy="direct" rectangleIndex="0">

<celldesigner:listOfLineDirection>

<celldesigner:lineDirection index="0" value="unknown"/>

</celldesigner:listOfLineDirection>

</celldesigner:connectScheme>

<celldesigner:line width="1.0" color="ffffcc00"/>

</celldesigner:extension>

</annotation>

<listOfReactants>

<speciesReference metaid="CDMT00084" species="s177">

<annotation>

<celldesigner:extension>

<celldesigner:alias>sa420</celldesigner:alias>

</celldesigner:extension>

</annotation>

</speciesReference>

</listOfReactants>

<listOfProducts>

<speciesReference metaid="CDMT00085" species="s176">

<annotation>

<celldesigner:extension>

<celldesigner:alias>sa415</celldesigner:alias>

</celldesigner:extension>

</annotation>

</speciesReference>

</listOfProducts>

</reaction>

<reaction metaid="re221" id="re141" name="DAT" reversible="false">

<annotation>

<celldesigner:extension>

<celldesigner:name>DAT</celldesigner:name>

<celldesigner:reactionType>STATE_TRANSITION</celldesigner:reactionType>

<celldesigner:baseReactants>

<celldesigner:baseReactant species="s177" alias="sa420"/>

</celldesigner:baseReactants>

<celldesigner:baseProducts>

<celldesigner:baseProduct species="s118" alias="sa419"/>

</celldesigner:baseProducts>

<celldesigner:connectScheme connectPolicy="direct" rectangleIndex="0">

<celldesigner:listOfLineDirection>

<celldesigner:lineDirection index="0" value="unknown"/>

</celldesigner:listOfLineDirection>

</celldesigner:connectScheme>

<celldesigner:line width="1.0" color="ffffcc00"/>

</celldesigner:extension>

</annotation>

<listOfReactants>

<speciesReference metaid="CDMT00089" species="s177">
<annotation>
<celldesigner:extension>
<celldesigner:alias>sa420</celldesigner:alias>
</celldesigner:extension>
</annotation>
</speciesReference>
</listOfReactants>
<listOfProducts>
<speciesReference metaid="CDMT00090" species="s118">
<annotation>
<celldesigner:extension>
<celldesigner:alias>sa419</celldesigner:alias>
</celldesigner:extension>
</annotation>
</speciesReference>
</listOfProducts>
</reaction>
<reaction metaid="re222" id="re142" name="PLA2 (12dgr-magLipase)" reversible="false">
<annotation>
<celldesigner:extension>
<celldesigner:name>PLA2 (12dgr-magLipase)</celldesigner:name>
<celldesigner:reactionType>STATE_TRANSITION</celldesigner:reactionType>
<celldesigner:baseReactants>
<celldesigner:baseReactant species="s177" alias="sa420"/>
</celldesigner:baseReactants>
<celldesigner:baseProducts>

<celldesigner:baseProduct species="s119" alias="sa421"/>

</celldesigner:baseProducts>

<celldesigner:listOfProductLinks>

<celldesigner:productLink product="s120" alias="sa422" targetLineIndex="-1,1">

<celldesigner:connectScheme connectPolicy="direct">

<celldesigner:listOfLineDirection>

<celldesigner:lineDirection index="0" value="unknown"/>

</celldesigner:listOfLineDirection>

</celldesigner:connectScheme>

<celldesigner:line width="1.0" color="ffffcc00" type="Straight"/>

</celldesigner:productLink>

</celldesigner:listOfProductLinks>

<celldesigner:connectScheme connectPolicy="direct" rectangleIndex="0">

<celldesigner:listOfLineDirection>

<celldesigner:lineDirection index="0" value="unknown"/>

</celldesigner:listOfLineDirection>

</celldesigner:connectScheme>

<celldesigner:line width="1.0" color="ffffcc00"/>

</celldesigner:extension>

</annotation>

<listOfReactants>

<speciesReference metaid="CDMT00092" species="s177">

<annotation>

<celldesigner:extension>

<celldesigner:alias>sa420</celldesigner:alias>

</celldesigner:extension>

</annotation>

</speciesReference>

</listOfReactants>

<listOfProducts>

<speciesReference metaid="CDMT00094" species="s119">

<annotation>

<celldesigner:extension>

<celldesigner:alias>sa421</celldesigner:alias>

</celldesigner:extension>

</annotation>

</speciesReference>

<speciesReference metaid="CDMT00096" species="s120">

<annotation>

<celldesigner:extension>

<celldesigner:alias>sa422</celldesigner:alias>

</celldesigner:extension>

</annotation>

</speciesReference>

</listOfProducts>

</reaction>

<reaction metaid="re224" id="re143" name="DGRAT" reversible="false">

<annotation>

<celldesigner:extension>

<celldesigner:name>DGRAT</celldesigner:name>

<celldesigner:reactionType>STATE_TRANSITION</celldesigner:reactionType>

<celldesigner:baseReactants>

<celldesigner:baseReactant species="s119" alias="sa421"/>

</celldesigner:baseReactants>

<celldesigner:baseProducts>

<celldesigner:baseProduct species="s177" alias="sa420">

<celldesigner:linkAnchor position="NNW"/>

</celldesigner:baseProduct>

</celldesigner:baseProducts>

<celldesigner:listOfReactantLinks>

<celldesigner:reactantLink reactant="s1101" alias="sa417" targetLineIndex="-1,0">

<celldesigner:connectScheme connectPolicy="direct">

<celldesigner:listOfLineDirection>

<celldesigner:lineDirection index="0" value="unknown"/>

</celldesigner:listOfLineDirection>

</celldesigner:connectScheme>

<celldesigner:line width="1.0" color="ffffcc00" type="Straight"/>

</celldesigner:reactantLink>

</celldesigner:listOfReactantLinks>

<celldesigner:connectScheme connectPolicy="direct" rectangleIndex="1">

<celldesigner:listOfLineDirection>

<celldesigner:lineDirection index="0" value="unknown"/>

<celldesigner:lineDirection index="1" value="unknown"/>

<celldesigner:lineDirection index="2" value="unknown"/>

</celldesigner:listOfLineDirection>

</celldesigner:connectScheme>

<celldesigner:editPoints>0.2619422647938183,-0.34114789687576863 0.7780426764461028,-0.33530980842425295</celldesigner:editPoints>

<celldesigner:line width="1.0" color="ffffcc00"/>

</celldesigner:extension>

</annotation>

<listOfReactants>

<speciesReference metaid="CDMT00097" species="s119">
<annotation>
<celldesigner:extension>
<celldesigner:alias>sa421</celldesigner:alias>
</celldesigner:extension>
</annotation>
</speciesReference>
<speciesReference metaid="CDMT00099" species="s1101">
<annotation>
<celldesigner:extension>
<celldesigner:alias>sa417</celldesigner:alias>
</celldesigner:extension>
</annotation>
</speciesReference>
</listOfReactants>
<listOfProducts>
<speciesReference metaid="CDMT00100" species="s177">
<annotation>
<celldesigner:extension>
<celldesigner:alias>sa420</celldesigner:alias>
</celldesigner:extension>
</annotation>
</speciesReference>
</listOfProducts>
</reaction>
<reaction metaid="re226" id="re144" name="Mag Lipase (mag-glycerol)" reversible="false">
<annotation>

<celldesigner:extension>

<celldesigner:name>Mag Lipase (mag-glycerol)</celldesigner:name>

<celldesigner:reactionType>STATE_TRANSITION</celldesigner:reactionType>

<celldesigner:baseReactants>

<celldesigner:baseReactant species="s119" alias="sa421"/>

</celldesigner:baseReactants>

<celldesigner:baseProducts>

<celldesigner:baseProduct species="s62" alias="sa408"/>

</celldesigner:baseProducts>

<celldesigner:listOfProductLinks>

<celldesigner:productLink product="s120" alias="sa410" targetLineIndex="-1,1">

<celldesigner:connectScheme connectPolicy="direct">

<celldesigner:listOfLineDirection>

<celldesigner:lineDirection index="0" value="unknown"/>

</celldesigner:listOfLineDirection>

</celldesigner:connectScheme>

<celldesigner:line width="1.0" color="ff000000" type="Straight"/>

</celldesigner:productLink>

</celldesigner:listOfProductLinks>

<celldesigner:connectScheme connectPolicy="direct" rectangleIndex="0">

<celldesigner:listOfLineDirection>

<celldesigner:lineDirection index="0" value="unknown"/>

</celldesigner:listOfLineDirection>

</celldesigner:connectScheme>

<celldesigner:line width="1.0" color="ff000000"/>

</celldesigner:extension>

</annotation>

<listOfReactants>
<speciesReference metaid="CDMT00102" species="s119">
<annotation>
<celldesigner:extension>
<celldesigner:alias>sa421</celldesigner:alias>
</celldesigner:extension>
</annotation>
</speciesReference>
</listOfReactants>
<listOfProducts>
<speciesReference metaid="CDMT00104" species="s62">
<annotation>
<celldesigner:extension>
<celldesigner:alias>sa408</celldesigner:alias>
</celldesigner:extension>
</annotation>
</speciesReference>
<speciesReference metaid="CDMT00109" species="s120">
<annotation>
<celldesigner:extension>
<celldesigner:alias>sa410</celldesigner:alias>
</celldesigner:extension>
</annotation>
</speciesReference>
</listOfProducts>
</reaction>
<reaction metaid="re114" id="re145" name="CEPT" reversible="false">

<annotation>

<celldesigner:extension>

<celldesigner:name>CEPT</celldesigner:name>

<celldesigner:reactionType>STATE_TRANSITION</celldesigner:reactionType>

<celldesigner:baseReactants>

<celldesigner:baseReactant species="s177" alias="sa420">

<celldesigner:linkAnchor position="SSE"/>

</celldesigner:baseReactant>

</celldesigner:baseReactants>

<celldesigner:baseProducts>

<celldesigner:baseProduct species="s121" alias="sa427"/>

</celldesigner:baseProducts>

<celldesigner:listOfReactantLinks>

<celldesigner:reactantLink reactant="s122" alias="sa424" targetLineIndex="-1,0">

<celldesigner:connectScheme connectPolicy="direct">

<celldesigner:listOfLineDirection>

<celldesigner:lineDirection index="0" value="unknown"/>

</celldesigner:listOfLineDirection>

</celldesigner:connectScheme>

<celldesigner:line width="1.0" color="ffffcc00" type="Straight"/>

</celldesigner:reactantLink>

</celldesigner:listOfReactantLinks>

<celldesigner:connectScheme connectPolicy="direct" rectangleIndex="0">

<celldesigner:listOfLineDirection>

<celldesigner:lineDirection index="0" value="unknown"/>

</celldesigner:listOfLineDirection>

</celldesigner:connectScheme>

<celldesigner:line width="1.0" color="ffffcc00"/>

</celldesigner:extension>

</annotation>

<listOfReactants>

<speciesReference metaid="CDMT00111" species="s177">

<annotation>

<celldesigner:extension>

<celldesigner:alias>sa420</celldesigner:alias>

</celldesigner:extension>

</annotation>

</speciesReference>

<speciesReference metaid="CDMT00113" species="s122">

<annotation>

<celldesigner:extension>

<celldesigner:alias>sa424</celldesigner:alias>

</celldesigner:extension>

</annotation>

</speciesReference>

</listOfReactants>

<listOfProducts>

<speciesReference metaid="CDMT00117" species="s121">

<annotation>

<celldesigner:extension>

<celldesigner:alias>sa427</celldesigner:alias>

</celldesigner:extension>

</annotation>

</speciesReference>

</listOfProducts>

</reaction>

<reaction metaid="re116" id="re146" name="EPCT" reversible="false">

<annotation>

<celldesigner:extension>

<celldesigner:name>EPCT</celldesigner:name>

<celldesigner:reactionType>STATE_TRANSITION</celldesigner:reactionType>

<celldesigner:baseReactants>

<celldesigner:baseReactant species="s125" alias="sa423"/>

</celldesigner:baseReactants>

<celldesigner:baseProducts>

<celldesigner:baseProduct species="s122" alias="sa424"/>

</celldesigner:baseProducts>

<celldesigner:connectScheme connectPolicy="direct" rectangleIndex="0">

<celldesigner:listOfLineDirection>

<celldesigner:lineDirection index="0" value="unknown"/>

</celldesigner:listOfLineDirection>

</celldesigner:connectScheme>

<celldesigner:line width="1.0" color="ffffcc00"/>

</celldesigner:extension>

</annotation>

<listOfReactants>

<speciesReference metaid="CDMT00118" species="s125">

<annotation>

<celldesigner:extension>

<celldesigner:alias>sa423</celldesigner:alias>

</celldesigner:extension>

</annotation>
</speciesReference>
</listOfReactants>
<listOfProducts>
<speciesReference metaid="CDMT00119" species="s122">
<annotation>
<celldesigner:extension>
<celldesigner:alias>sa424</celldesigner:alias>
</celldesigner:extension>
</annotation>
</speciesReference>
</listOfProducts>
</reaction>
<reaction metaid="re118" id="re147" name="PEMT" reversible="false">
<annotation>
<celldesigner:extension>
<celldesigner:name>PEMT</celldesigner:name>
<celldesigner:reactionType>STATE_TRANSITION</celldesigner:reactionType>
<celldesigner:baseReactants>
<celldesigner:baseReactant species="s121" alias="sa427">
<celldesigner:linkAnchor position="S"/>
</celldesigner:baseReactant>
</celldesigner:baseReactants>
<celldesigner:baseProducts>
<celldesigner:baseProduct species="s128" alias="sa428">
<celldesigner:linkAnchor position="N"/>
</celldesigner:baseProduct>

</celldesigner:baseProducts>
<celldesigner:connectScheme connectPolicy="direct" rectangleIndex="0">
<celldesigner:listOfLineDirection>
<celldesigner:lineDirection index="0" value="unknown"/>
</celldesigner:listOfLineDirection>
</celldesigner:connectScheme>
<celldesigner:line width="1.0" color="ffffcc00"/>
</celldesigner:extension>
</annotation>
<listOfReactants>
<speciesReference metaid="CDMT00120" species="s121">
<annotation>
<celldesigner:extension>
<celldesigner:alias>sa427</celldesigner:alias>
</celldesigner:extension>
</annotation>
</speciesReference>
</listOfReactants>
<listOfProducts>
<speciesReference metaid="CDMT00121" species="s128">
<annotation>
<celldesigner:extension>
<celldesigner:alias>sa428</celldesigner:alias>
</celldesigner:extension>
</annotation>
</speciesReference>
</listOfProducts>

</reaction>

<reaction metaid="re120" id="re148" name="PEMT_1" reversible="false">

<annotation>

<celldesigner:extension>

<celldesigner:name>PEMT_1</celldesigner:name>

<celldesigner:reactionType>STATE_TRANSITION</celldesigner:reactionType>

<celldesigner:baseReactants>

<celldesigner:baseReactant species="s128" alias="sa428"/>

</celldesigner:baseReactants>

<celldesigner:baseProducts>

<celldesigner:baseProduct species="s131" alias="sa430"/>

</celldesigner:baseProducts>

<celldesigner:connectScheme connectPolicy="direct" rectangleIndex="0">

<celldesigner:listOfLineDirection>

<celldesigner:lineDirection index="0" value="unknown"/>

</celldesigner:listOfLineDirection>

</celldesigner:connectScheme>

<celldesigner:line width="1.0" color="ffffcc00"/>

</celldesigner:extension>

</annotation>

<listOfReactants>

<speciesReference metaid="CDMT00122" species="s128">

<annotation>

<celldesigner:extension>

<celldesigner:alias>sa428</celldesigner:alias>

</celldesigner:extension>

</annotation>

</speciesReference>

</listOfReactants>

<listOfProducts>

<speciesReference metaid="CDMT00123" species="s131">

<annotation>

<celldesigner:extension>

<celldesigner:alias>sa430</celldesigner:alias>

</celldesigner:extension>

</annotation>

</speciesReference>

</listOfProducts>

</reaction>

<reaction metaid="re122" id="re149" name="PEMT_2" reversible="false">

<annotation>

<celldesigner:extension>

<celldesigner:name>PEMT_2</celldesigner:name>

<celldesigner:reactionType>STATE_TRANSITION</celldesigner:reactionType>

<celldesigner:baseReactants>

<celldesigner:baseReactant species="s131" alias="sa430">

<celldesigner:linkAnchor position="S"/>

</celldesigner:baseReactant>

</celldesigner:baseReactants>

<celldesigner:baseProducts>

<celldesigner:baseProduct species="s132" alias="sa431"/>

</celldesigner:baseProducts>

<celldesigner:connectScheme connectPolicy="direct" rectangleIndex="0">

<celldesigner:listOfLineDirection>

<celldesigner:lineDirection index="0" value="unknown"/>
</celldesigner:listOfLineDirection>
</celldesigner:connectScheme>
<celldesigner:line width="1.0" color="ff000000"/>
</celldesigner:extension>
</annotation>
<listOfReactants>
<speciesReference metaid="CDMT00124" species="s131">
<annotation>
<celldesigner:extension>
<celldesigner:alias>sa430</celldesigner:alias>
</celldesigner:extension>
</annotation>
</speciesReference>
</listOfReactants>
<listOfProducts>
<speciesReference metaid="CDMT00125" species="s132">
<annotation>
<celldesigner:extension>
<celldesigner:alias>sa431</celldesigner:alias>
</celldesigner:extension>
</annotation>
</speciesReference>
</listOfProducts>
</reaction>
<reaction metaid="re124" id="re150" name="CDPDAGS-like" reversible="false">
<annotation>

<celldesigner:extension>

<celldesigner:name>CDPDAGS-like</celldesigner:name>

<celldesigner:reactionType>STATE_TRANSITION</celldesigner:reactionType>

<celldesigner:baseReactants>

<celldesigner:baseReactant species="s176" alias="sa415">

<celldesigner:linkAnchor position="ESE"/>

</celldesigner:baseReactant>

</celldesigner:baseReactants>

<celldesigner:baseProducts>

<celldesigner:baseProduct species="s135" alias="sa416">

<celldesigner:linkAnchor position="WNW"/>

</celldesigner:baseProduct>

</celldesigner:baseProducts>

<celldesigner:connectScheme connectPolicy="direct" rectangleIndex="0">

<celldesigner:listOfLineDirection>

<celldesigner:lineDirection index="0" value="unknown"/>

</celldesigner:listOfLineDirection>

</celldesigner:connectScheme>

<celldesigner:line width="1.0" color="ffffcc00"/>

</celldesigner:extension>

</annotation>

<listOfReactants>

<speciesReference metaid="CDMT00126" species="s176">

<annotation>

<celldesigner:extension>

<celldesigner:alias>sa415</celldesigner:alias>

</celldesigner:extension>

</annotation>

</speciesReference>

</listOfReactants>

<listOfProducts>

<speciesReference metaid="CDMT00127" species="s135">

<annotation>

<celldesigner:extension>

<celldesigner:alias>sa416</celldesigner:alias>

</celldesigner:extension>

</annotation>

</speciesReference>

</listOfProducts>

</reaction>

<reaction metaid="re126" id="re151" name="InoPT" reversible="false">

<annotation>

<celldesigner:extension>

<celldesigner:name>InoPT</celldesigner:name>

<celldesigner:reactionType>STATE_TRANSITION</celldesigner:reactionType>

<celldesigner:baseReactants>

<celldesigner:baseReactant species="s135" alias="sa416">

<celldesigner:linkAnchor position="SE"/>

</celldesigner:baseReactant>

</celldesigner:baseReactants>

<celldesigner:baseProducts>

<celldesigner:baseProduct species="s137" alias="sa414"/>

</celldesigner:baseProducts>

<celldesigner:listOfReactantLinks>

<celldesigner:reactantLink reactant="s138" alias="sa418" targetLineIndex="-1,0">

<celldesigner:connectScheme connectPolicy="direct">

<celldesigner:listOfLineDirection>

<celldesigner:lineDirection index="0" value="unknown"/>

</celldesigner:listOfLineDirection>

</celldesigner:connectScheme>

<celldesigner:line width="1.0" color="ffffcc00" type="Straight"/>

</celldesigner:reactantLink>

</celldesigner:listOfReactantLinks>

<celldesigner:connectScheme connectPolicy="direct" rectangleIndex="0">

<celldesigner:listOfLineDirection>

<celldesigner:lineDirection index="0" value="unknown"/>

</celldesigner:listOfLineDirection>

</celldesigner:connectScheme>

<celldesigner:line width="1.0" color="ffffcc00"/>

</celldesigner:extension>

</annotation>

<listOfReactants>

<speciesReference metaid="CDMT00128" species="s135">

<annotation>

<celldesigner:extension>

<celldesigner:alias>sa416</celldesigner:alias>

</celldesigner:extension>

</annotation>

</speciesReference>

<speciesReference metaid="CDMT00129" species="s138">

<annotation>

<celldesigner:extension>

<celldesigner:alias>sa418</celldesigner:alias>

</celldesigner:extension>

</annotation>

</speciesReference>

</listOfReactants>

<listOfProducts>

<speciesReference metaid="CDMT00130" species="s137">

<annotation>

<celldesigner:extension>

<celldesigner:alias>sa414</celldesigner:alias>

</celldesigner:extension>

</annotation>

</speciesReference>

</listOfProducts>

</reaction>

<reaction metaid="re127" id="re152" name="PG3PT" reversible="false">

<annotation>

<celldesigner:extension>

<celldesigner:name>PG3PT</celldesigner:name>

<celldesigner:reactionType>STATE_TRANSITION</celldesigner:reactionType>

<celldesigner:baseReactants>

<celldesigner:baseReactant species="s135" alias="sa416">

<celldesigner:linkAnchor position="N"/>

</celldesigner:baseReactant>

</celldesigner:baseReactants>

<celldesigner:baseProducts>

<celldesigner:baseProduct species="s139" alias="sa412">

<celldesigner:linkAnchor position="S"/>

</celldesigner:baseProduct>

</celldesigner:baseProducts>

<celldesigner:listOfReactantLinks>

<celldesigner:reactantLink reactant="s140" alias="sa413" targetLineIndex="-1,0">

<celldesigner:linkAnchor position="WNW"/>

<celldesigner:connectScheme connectPolicy="direct">

<celldesigner:listOfLineDirection>

<celldesigner:lineDirection index="0" value="unknown"/>

</celldesigner:listOfLineDirection>

</celldesigner:connectScheme>

<celldesigner:line width="1.0" color="ffffcc00" type="Straight"/>

</celldesigner:reactantLink>

</celldesigner:listOfReactantLinks>

<celldesigner:connectScheme connectPolicy="direct" rectangleIndex="0">

<celldesigner:listOfLineDirection>

<celldesigner:lineDirection index="0" value="unknown"/>

</celldesigner:listOfLineDirection>

</celldesigner:connectScheme>

<celldesigner:line width="1.0" color="ffffcc00"/>

</celldesigner:extension>

</annotation>

<listOfReactants>

<speciesReference metaid="CDMT00131" species="s135">

<annotation>

<celldesigner:extension>

<celldesigner:alias>sa416</celldesigner:alias>

</celldesigner:extension>

</annotation>

</speciesReference>

<speciesReference metaid="CDMT00132" species="s140">

<annotation>

<celldesigner:extension>

<celldesigner:alias>sa413</celldesigner:alias>

</celldesigner:extension>

</annotation>

</speciesReference>

</listOfReactants>

<listOfProducts>

<speciesReference metaid="CDMT00133" species="s139">

<annotation>

<celldesigner:extension>

<celldesigner:alias>sa412</celldesigner:alias>

</celldesigner:extension>

</annotation>

</speciesReference>

</listOfProducts>

</reaction>

<reaction metaid="re465" id="re153" name="PE2AcylHydrolase[c]" reversible="false">

<annotation>

<celldesigner:extension>

<celldesigner:name>PE2AcylHydrolase[c]</celldesigner:name>

<celldesigner:reactionType>STATE_TRANSITION</celldesigner:reactionType>

<celldesigner:baseReactants>

<celldesigner:baseReactant species="s121" alias="sa427"/>

</celldesigner:baseReactants>

<celldesigner:baseProducts>

<celldesigner:baseProduct species="s423" alias="sa426"/>

</celldesigner:baseProducts>

<celldesigner:listOfProductLinks>

<celldesigner:productLink product="s422" alias="sa425" targetLineIndex="-1,1">

<celldesigner:connectScheme connectPolicy="direct">

<celldesigner:listOfLineDirection>

<celldesigner:lineDirection index="0" value="unknown"/>

</celldesigner:listOfLineDirection>

</celldesigner:connectScheme>

<celldesigner:line width="1.0" color="ffffcc00" type="Straight"/>

</celldesigner:productLink>

</celldesigner:listOfProductLinks>

<celldesigner:connectScheme connectPolicy="direct" rectangleIndex="0">

<celldesigner:listOfLineDirection>

<celldesigner:lineDirection index="0" value="unknown"/>

</celldesigner:listOfLineDirection>

</celldesigner:connectScheme>

<celldesigner:line width="1.0" color="ffffcc00"/>

</celldesigner:extension>

</annotation>

<listOfReactants>

<speciesReference metaid="CDMT00139" species="s121">

<annotation>

<celldesigner:extension>
<celldesigner:alias>sa427</celldesigner:alias>
</celldesigner:extension>
</annotation>
</speciesReference>
</listOfReactants>
<listOfProducts>
<speciesReference metaid="CDMT00141" species="s423">
<annotation>
<celldesigner:extension>
<celldesigner:alias>sa426</celldesigner:alias>
</celldesigner:extension>
</annotation>
</speciesReference>
<speciesReference metaid="CDMT00142" species="s422">
<annotation>
<celldesigner:extension>
<celldesigner:alias>sa425</celldesigner:alias>
</celldesigner:extension>
</annotation>
</speciesReference>
</listOfProducts>
</reaction>
<reaction metaid="re466" id="re154" name="LysoPhosphoLipaseI[c[" reversible="false">
<annotation>
<celldesigner:extension>
<celldesigner:name>LysoPhosphoLipaseI[c[</celldesigner:name>

<celldesigner:reactionType>STATE_TRANSITION</celldesigner:reactionType>

<celldesigner:baseReactants>

<celldesigner:baseReactant species="s423" alias="sa426"/>

</celldesigner:baseReactants>

<celldesigner:baseProducts>

<celldesigner:baseProduct species="s424" alias="sa429"/>

</celldesigner:baseProducts>

<celldesigner:listOfProductLinks>

<celldesigner:productLink product="s120" alias="sa422" targetLineIndex="-1,1">

<celldesigner:connectScheme connectPolicy="direct">

<celldesigner:listOfLineDirection>

<celldesigner:lineDirection index="0" value="unknown"/>

</celldesigner:listOfLineDirection>

</celldesigner:connectScheme>

<celldesigner:line width="1.0" color="ffffcc00" type="Straight"/>

</celldesigner:productLink>

</celldesigner:listOfProductLinks>

<celldesigner:connectScheme connectPolicy="direct" rectangleIndex="0">

<celldesigner:listOfLineDirection>

<celldesigner:lineDirection index="0" value="unknown"/>

</celldesigner:listOfLineDirection>

</celldesigner:connectScheme>

<celldesigner:line width="1.0" color="ffffcc00"/>

</celldesigner:extension>

</annotation>

<listOfReactants>

<speciesReference metaid="CDMT00145" species="s423">

<annotation>
<celldesigner:extension>
<celldesigner:alias>sa426</celldesigner:alias>
</celldesigner:extension>
</annotation>
</speciesReference>
</listOfReactants>
<listOfProducts>
<speciesReference metaid="CDMT00146" species="s424">
<annotation>
<celldesigner:extension>
<celldesigner:alias>sa429</celldesigner:alias>
</celldesigner:extension>
</annotation>
</speciesReference>
<speciesReference metaid="CDMT00147" species="s120">
<annotation>
<celldesigner:extension>
<celldesigner:alias>sa422</celldesigner:alias>
</celldesigner:extension>
</annotation>
</speciesReference>
</listOfProducts>
</reaction>
<reaction metaid="re467" id="re155" name="GPDPD[c]" reversible="false">
<annotation>
<celldesigner:extension>

<celldesigner:name>GPDPD[c]</celldesigner:name>

<celldesigner:reactionType>STATE_TRANSITION</celldesigner:reactionType>

<celldesigner:baseReactants>

<celldesigner:baseReactant species="s424" alias="sa429"/>

</celldesigner:baseReactants>

<celldesigner:baseProducts>

<celldesigner:baseProduct species="s61" alias="sa409"/>

</celldesigner:baseProducts>

<celldesigner:listOfProductLinks>

<celldesigner:productLink product="s426" alias="sa432" targetLineIndex="-1,1">

<celldesigner:linkAnchor position="NNW"/>

<celldesigner:connectScheme connectPolicy="direct">

<celldesigner:listOfLineDirection>

<celldesigner:lineDirection index="0" value="unknown"/>

</celldesigner:listOfLineDirection>

</celldesigner:connectScheme>

<celldesigner:line width="1.0" color="ffffcc00" type="Straight"/>

</celldesigner:productLink>

</celldesigner:listOfProductLinks>

<celldesigner:connectScheme connectPolicy="direct" rectangleIndex="1">

<celldesigner:listOfLineDirection>

<celldesigner:lineDirection index="0" value="unknown"/>

<celldesigner:lineDirection index="1" value="unknown"/>

<celldesigner:lineDirection index="2" value="unknown"/>

</celldesigner:listOfLineDirection>

</celldesigner:connectScheme>

<celldesigner:editPoints>0.057934508816121166,-0.35012594458438295 0.8780856423173805,-0.48060453400503844</celldesigner:editPoints>

<celldesigner:line width="1.0" color="ffffcc00"/>

</celldesigner:extension>

</annotation>

<listOfReactants>

<speciesReference metaid="CDMT00148" species="s424">

<annotation>

<celldesigner:extension>

<celldesigner:alias>sa429</celldesigner:alias>

</celldesigner:extension>

</annotation>

</speciesReference>

</listOfReactants>

<listOfProducts>

<speciesReference metaid="CDMT00149" species="s61">

<annotation>

<celldesigner:extension>

<celldesigner:alias>sa409</celldesigner:alias>

</celldesigner:extension>

</annotation>

</speciesReference>

<speciesReference metaid="CDMT00150" species="s426">

<annotation>

<celldesigner:extension>

<celldesigner:alias>sa432</celldesigner:alias>

</celldesigner:extension>

</annotation>

</speciesReference>

</listOfProducts>
</reaction>
<reaction metaid="re468" id="re156" name="PC2AcylHydrolase[c]" reversible="false">
<annotation>
<celldesigner:extension>
<celldesigner:name>PC2AcylHydrolase[c]</celldesigner:name>
<celldesigner:reactionType>STATE_TRANSITION</celldesigner:reactionType>
<celldesigner:baseReactants>
<celldesigner:baseReactant species="s132" alias="sa431"/>
</celldesigner:baseReactants>
<celldesigner:baseProducts>
<celldesigner:baseProduct species="s427" alias="sa433"/>
</celldesigner:baseProducts>
<celldesigner:listOfProductLinks>
<celldesigner:productLink product="s422" alias="sa425" targetLineIndex="-1,1">
<celldesigner:connectScheme connectPolicy="direct">
<celldesigner:listOfLineDirection>
<celldesigner:lineDirection index="0" value="unknown"/>
</celldesigner:listOfLineDirection>
</celldesigner:connectScheme>
<celldesigner:line width="1.0" color="ffffcc00" type="Straight"/>
</celldesigner:productLink>
</celldesigner:listOfProductLinks>
<celldesigner:connectScheme connectPolicy="direct" rectangleIndex="0">
<celldesigner:listOfLineDirection>
<celldesigner:lineDirection index="0" value="unknown"/>
</celldesigner:listOfLineDirection>

</celldesigner:connectScheme>

<celldesigner:line width="1.0" color="ffffcc00"/>

</celldesigner:extension>

</annotation>

<listOfReactants>

<speciesReference metaid="CDMT00151" species="s132">

<annotation>

<celldesigner:extension>

<celldesigner:alias>sa431</celldesigner:alias>

</celldesigner:extension>

</annotation>

</speciesReference>

</listOfReactants>

<listOfProducts>

<speciesReference metaid="CDMT00152" species="s427">

<annotation>

<celldesigner:extension>

<celldesigner:alias>sa433</celldesigner:alias>

</celldesigner:extension>

</annotation>

</speciesReference>

<speciesReference metaid="CDMT00153" species="s422">

<annotation>

<celldesigner:extension>

<celldesigner:alias>sa425</celldesigner:alias>

</celldesigner:extension>

</annotation>

</speciesReference>

</listOfProducts>

</reaction>

<reaction metaid="re469" id="re157" name="LysoPhosphoLipaseI[c]" reversible="false">

<annotation>

<celldesigner:extension>

<celldesigner:name>LysoPhosphoLipaseI[c]</celldesigner:name>

<celldesigner:reactionType>STATE_TRANSITION</celldesigner:reactionType>

<celldesigner:baseReactants>

<celldesigner:baseReactant species="s427" alias="sa433"/>

</celldesigner:baseReactants>

<celldesigner:baseProducts>

<celldesigner:baseProduct species="s428" alias="sa434"/>

</celldesigner:baseProducts>

<celldesigner:listOfProductLinks>

<celldesigner:productLink product="s120" alias="sa435" targetLineIndex="-1,1">

<celldesigner:connectScheme connectPolicy="direct">

<celldesigner:listOfLineDirection>

<celldesigner:lineDirection index="0" value="unknown"/>

</celldesigner:listOfLineDirection>

</celldesigner:connectScheme>

<celldesigner:line width="1.0" color="ff000000" type="Straight"/>

</celldesigner:productLink>

</celldesigner:listOfProductLinks>

<celldesigner:connectScheme connectPolicy="direct" rectangleIndex="0">

<celldesigner:listOfLineDirection>

<celldesigner:lineDirection index="0" value="unknown"/>

</celldesigner:listOfLineDirection>

</celldesigner:connectScheme>

<celldesigner:line width="1.0" color="ff000000"/>

</celldesigner:extension>

</annotation>

<listOfReactants>

<speciesReference metaid="CDMT00154" species="s427">

<annotation>

<celldesigner:extension>

<celldesigner:alias>sa433</celldesigner:alias>

</celldesigner:extension>

</annotation>

</speciesReference>

</listOfReactants>

<listOfProducts>

<speciesReference metaid="CDMT00155" species="s428">

<annotation>

<celldesigner:extension>

<celldesigner:alias>sa434</celldesigner:alias>

</celldesigner:extension>

</annotation>

</speciesReference>

<speciesReference metaid="CDMT00156" species="s120">

<annotation>

<celldesigner:extension>

<celldesigner:alias>sa435</celldesigner:alias>

</celldesigner:extension>

</annotation>

</speciesReference>

</listOfProducts>

</reaction>

<reaction metaid="re470" id="re158" name="GPDPD[m]" reversible="false">

<annotation>

<celldesigner:extension>

<celldesigner:name>GPDPD[m]</celldesigner:name>

<celldesigner:reactionType>STATE_TRANSITION</celldesigner:reactionType>

<celldesigner:baseReactants>

<celldesigner:baseReactant species="s428" alias="sa434"/>

</celldesigner:baseReactants>

<celldesigner:baseProducts>

<celldesigner:baseProduct species="s61" alias="sa409"/>

</celldesigner:baseProducts>

<celldesigner:listOfProductLinks>

<celldesigner:productLink product="s420" alias="sa407" targetLineIndex="-1,1">

<celldesigner:connectScheme connectPolicy="direct">

<celldesigner:listOfLineDirection>

<celldesigner:lineDirection index="0" value="unknown"/>

</celldesigner:listOfLineDirection>

</celldesigner:connectScheme>

<celldesigner:line width="1.0" color="ff000000" type="Straight"/>

</celldesigner:productLink>

</celldesigner:listOfProductLinks>

<celldesigner:connectScheme connectPolicy="direct" rectangleIndex="2">

<celldesigner:listOfLineDirection>

<celldesigner:lineDirection index="0" value="unknown"/>

<celldesigner:lineDirection index="1" value="unknown"/>

<celldesigner:lineDirection index="2" value="unknown"/>

</celldesigner:listOfLineDirection>

</celldesigner:connectScheme>

<celldesigner:editPoints>0.059310344827586015,0.08827586206896543 0.9696551724137918,0.1641379310344857</celldesigner:editPoints>

<celldesigner:line width="1.0" color="ff000000"/>

</celldesigner:extension>

</annotation>

<listOfReactants>

<speciesReference metaid="CDMT00157" species="s428">

<annotation>

<celldesigner:extension>

<celldesigner:alias>sa434</celldesigner:alias>

</celldesigner:extension>

</annotation>

</speciesReference>

</listOfReactants>

<listOfProducts>

<speciesReference metaid="CDMT00158" species="s61">

<annotation>

<celldesigner:extension>

<celldesigner:alias>sa409</celldesigner:alias>

</celldesigner:extension>

</annotation>

</speciesReference>

<speciesReference metaid="CDMT00159" species="s420">

<annotation>
<celldesigner:extension>
<celldesigner:alias>sa407</celldesigner:alias>
</celldesigner:extension>
</annotation>
</speciesReference>
</listOfProducts>
</reaction>
<reaction metaid="re258" id="re159" name="G3PAcT" reversible="false">
<annotation>
<celldesigner:extension>
<celldesigner:name>G3PAcT</celldesigner:name>
<celldesigner:reactionType>STATE_TRANSITION</celldesigner:reactionType>
<celldesigner:baseReactants>
<celldesigner:baseReactant species="s61" alias="sa409"/>
</celldesigner:baseReactants>
<celldesigner:baseProducts>
<celldesigner:baseProduct species="s175" alias="sa411"/>
</celldesigner:baseProducts>
<celldesigner:listOfReactantLinks>
<celldesigner:reactantLink reactant="s1101" alias="sa417" targetLineIndex="-1,0">
<celldesigner:connectScheme connectPolicy="direct">
<celldesigner:listOfLineDirection>
<celldesigner:lineDirection index="0" value="unknown"/>
</celldesigner:listOfLineDirection>
</celldesigner:connectScheme>
<celldesigner:line width="1.0" color="ffffcc00" type="Straight"/>

</celldesigner:reactantLink>

</celldesigner:listOfReactantLinks>

<celldesigner:connectScheme connectPolicy="direct" rectangleIndex="0">

<celldesigner:listOfLineDirection>

<celldesigner:lineDirection index="0" value="unknown"/>

</celldesigner:listOfLineDirection>

</celldesigner:connectScheme>

<celldesigner:line width="1.0" color="ffffcc00"/>

</celldesigner:extension>

</annotation>

<listOfReactants>

<speciesReference metaid="CDMT00160" species="s61">

<annotation>

<celldesigner:extension>

<celldesigner:alias>sa409</celldesigner:alias>

</celldesigner:extension>

</annotation>

</speciesReference>

<speciesReference metaid="CDMT00161" species="s1101">

<annotation>

<celldesigner:extension>

<celldesigner:alias>sa417</celldesigner:alias>

</celldesigner:extension>

</annotation>

</speciesReference>

</listOfReactants>

<listOfProducts>

<speciesReference metaid="CDMT00162" species="s175">
<annotation>
<celldesigner:extension>
<celldesigner:alias>sa411</celldesigner:alias>
</celldesigner:extension>
</annotation>
</speciesReference>
</listOfProducts>
</reaction>
<reaction metaid="re431" id="re160" name="GK" reversible="false">
<annotation>
<celldesigner:extension>
<celldesigner:name>GK</celldesigner:name>
<celldesigner:reactionType>STATE_TRANSITION</celldesigner:reactionType>
<celldesigner:baseReactants>
<celldesigner:baseReactant species="s62" alias="sa408"/>
</celldesigner:baseReactants>
<celldesigner:baseProducts>
<celldesigner:baseProduct species="s61" alias="sa409"/>
</celldesigner:baseProducts>
<celldesigner:connectScheme connectPolicy="direct" rectangleIndex="0">
<celldesigner:listOfLineDirection>
<celldesigner:lineDirection index="0" value="unknown"/>
</celldesigner:listOfLineDirection>
</celldesigner:connectScheme>
<celldesigner:line width="1.0" color="ff000000"/>
</celldesigner:extension>

</annotation>
<listOfReactants>
<speciesReference metaid="CDMT00163" species="s62">
<annotation>
<celldesigner:extension>
<celldesigner:alias>sa408</celldesigner:alias>
</celldesigner:extension>
</annotation>
</speciesReference>
</listOfReactants>
<listOfProducts>
<speciesReference metaid="CDMT00164" species="s61">
<annotation>
<celldesigner:extension>
<celldesigner:alias>sa409</celldesigner:alias>
</celldesigner:extension>
</annotation>
</speciesReference>
</listOfProducts>
</reaction>
<reaction metaid="re62" id="re170" name="Spontaneous MG synthesis" reversible="false">
<annotation>
<celldesigner:extension>
<celldesigner:name>Spontaneous MG synthesis</celldesigner:name>
<celldesigner:reactionType>STATE_TRANSITION</celldesigner:reactionType>
<celldesigner:baseReactants>
<celldesigner:baseReactant species="s60" alias="sa43"/>

</celldesigner:baseReactants>

<celldesigner:baseProducts>

<celldesigner:baseProduct species="s68" alias="sa456">

<celldesigner:linkAnchor position="N"/>

</celldesigner:baseProduct>

</celldesigner:baseProducts>

<celldesigner:connectScheme connectPolicy="direct" rectangleIndex="0">

<celldesigner:listOfLineDirection>

<celldesigner:lineDirection index="0" value="unknown"/>

</celldesigner:listOfLineDirection>

</celldesigner:connectScheme>

<celldesigner:line width="1.0" color="ff000000"/>

</celldesigner:extension>

</annotation>

<listOfReactants>

<speciesReference metaid="CDMT00165" species="s60">

<annotation>

<celldesigner:extension>

<celldesigner:alias>sa43</celldesigner:alias>

</celldesigner:extension>

</annotation>

</speciesReference>

</listOfReactants>

<listOfProducts>

<speciesReference metaid="CDMT00166" species="s68">

<annotation>

<celldesigner:extension>

<celldesigner:alias>sa456</celldesigner:alias>

</celldesigner:extension>

</annotation>

</speciesReference>

</listOfProducts>

</reaction>

<reaction metaid="re63" id="re171" name="HTA_SpontaneousSynthesis" reversible="false">

<annotation>

<celldesigner:extension>

<celldesigner:name>HTA_SpontaneousSynthesis</celldesigner:name>

<celldesigner:reactionType>STATE_TRANSITION</celldesigner:reactionType>

<celldesigner:baseReactants>

<celldesigner:baseReactant species="s68" alias="sa456"/>

</celldesigner:baseReactants>

<celldesigner:baseProducts>

<celldesigner:baseProduct species="s69" alias="sa459"/>

</celldesigner:baseProducts>

<celldesigner:listOfReactantLinks>

<celldesigner:reactantLink reactant="s70" alias="sa458" targetLineIndex="-1,0">

<celldesigner:connectScheme connectPolicy="direct">

<celldesigner:listOfLineDirection>

<celldesigner:lineDirection index="0" value="unknown"/>

</celldesigner:listOfLineDirection>

</celldesigner:connectScheme>

<celldesigner:line width="1.0" color="ff000000" type="Straight"/>

</celldesigner:reactantLink>

</celldesigner:listOfReactantLinks>

<celldesigner:connectScheme connectPolicy="direct" rectangleIndex="0">
<celldesigner:listOfLineDirection>
<celldesigner:lineDirection index="0" value="unknown"/>
</celldesigner:listOfLineDirection>
</celldesigner:connectScheme>
<celldesigner:line width="1.0" color="ff000000"/>
</celldesigner:extension>
</annotation>
<listOfReactants>
<speciesReference metaid="CDMT00167" species="s68">
<annotation>
<celldesigner:extension>
<celldesigner:alias>sa456</celldesigner:alias>
</celldesigner:extension>
</annotation>
</speciesReference>
<speciesReference metaid="CDMT00168" species="s70">
<annotation>
<celldesigner:extension>
<celldesigner:alias>sa458</celldesigner:alias>
</celldesigner:extension>
</annotation>
</speciesReference>
</listOfReactants>
<listOfProducts>
<speciesReference metaid="CDMT00170" species="s69">
<annotation>

<celldesigner:extension>

<celldesigner:alias>sa459</celldesigner:alias>

</celldesigner:extension>

</annotation>

</speciesReference>

</listOfProducts>

</reaction>

<reaction metaid="re64" id="re172" name="GLOI" reversible="false">

<annotation>

<celldesigner:extension>

<celldesigner:name>GLOI</celldesigner:name>

<celldesigner:reactionType>STATE_TRANSITION</celldesigner:reactionType>

<celldesigner:baseReactants>

<celldesigner:baseReactant species="s69" alias="sa459"/>

</celldesigner:baseReactants>

<celldesigner:baseProducts>

<celldesigner:baseProduct species="s71" alias="sa461"/>

</celldesigner:baseProducts>

<celldesigner:connectScheme connectPolicy="direct" rectangleIndex="0">

<celldesigner:listOfLineDirection>

<celldesigner:lineDirection index="0" value="unknown"/>

</celldesigner:listOfLineDirection>

</celldesigner:connectScheme>

<celldesigner:line width="1.0" color="ff000000"/>

</celldesigner:extension>

</annotation>

<listOfReactants>

<speciesReference metaid="CDMT00172" species="s69">
<annotation>
<celldesigner:extension>
<celldesigner:alias>sa459</celldesigner:alias>
</celldesigner:extension>
</annotation>
</speciesReference>
</listOfReactants>
<listOfProducts>
<speciesReference metaid="CDMT00173" species="s71">
<annotation>
<celldesigner:extension>
<celldesigner:alias>sa461</celldesigner:alias>
</celldesigner:extension>
</annotation>
</speciesReference>
</listOfProducts>
</reaction>
<reaction metaid="re65" id="re173" name="GLOII" reversible="false">
<annotation>
<celldesigner:extension>
<celldesigner:name>GLOII</celldesigner:name>
<celldesigner:reactionType>STATE_TRANSITION</celldesigner:reactionType>
<celldesigner:baseReactants>
<celldesigner:baseReactant species="s71" alias="sa461"/>
</celldesigner:baseReactants>
<celldesigner:baseProducts>

<celldesigner:baseProduct species="s72" alias="sa466"/>

</celldesigner:baseProducts>

<celldesigner:listOfProductLinks>

<celldesigner:productLink product="s70" alias="sa458" targetLineIndex="-1,1">

<celldesigner:connectScheme connectPolicy="direct">

<celldesigner:listOfLineDirection>

<celldesigner:lineDirection index="0" value="unknown"/>

<celldesigner:lineDirection index="1" value="unknown"/>

</celldesigner:listOfLineDirection>

</celldesigner:connectScheme>

<celldesigner:editPoints>0.497027182265545,0.26999084092708703</celldesigner:editPoints>

<celldesigner:line width="1.0" color="ff000000" type="Straight"/>

</celldesigner:productLink>

</celldesigner:listOfProductLinks>

<celldesigner:connectScheme connectPolicy="direct" rectangleIndex="0">

<celldesigner:listOfLineDirection>

<celldesigner:lineDirection index="0" value="unknown"/>

</celldesigner:listOfLineDirection>

</celldesigner:connectScheme>

<celldesigner:line width="1.0" color="ff000000"/>

</celldesigner:extension>

</annotation>

<listOfReactants>

<speciesReference metaid="CDMT00175" species="s71">

<annotation>

<celldesigner:extension>

<celldesigner:alias>sa461</celldesigner:alias>

</celldesigner:extension>
</annotation>
</speciesReference>
</listOfReactants>
<listOfProducts>
<speciesReference metaid="CDMT00177" species="s72">
<annotation>
<celldesigner:extension>
<celldesigner:alias>sa466</celldesigner:alias>
</celldesigner:extension>
</annotation>
</speciesReference>
<speciesReference metaid="CDMT00179" species="s70">
<annotation>
<celldesigner:extension>
<celldesigner:alias>sa458</celldesigner:alias>
</celldesigner:extension>
</annotation>
</speciesReference>
</listOfProducts>
</reaction>
<reaction metaid="re66" id="re174" name="DLDH" reversible="false">
<annotation>
<celldesigner:extension>
<celldesigner:name>DLDH</celldesigner:name>
<celldesigner:reactionType>STATE_TRANSITION</celldesigner:reactionType>
<celldesigner:baseReactants>

<celldesigner:baseReactant species="s72" alias="sa466"/>

</celldesigner:baseReactants>

<celldesigner:baseProducts>

<celldesigner:baseProduct species="s2" alias="sa143">

<celldesigner:linkAnchor position="S"/>

</celldesigner:baseProduct>

</celldesigner:baseProducts>

<celldesigner:connectScheme connectPolicy="direct" rectangleIndex="0">

<celldesigner:listOfLineDirection>

<celldesigner:lineDirection index="0" value="unknown"/>

</celldesigner:listOfLineDirection>

</celldesigner:connectScheme>

<celldesigner:line width="1.0" color="ff000000"/>

</celldesigner:extension>

</annotation>

<listOfReactants>

<speciesReference metaid="CDMT00181" species="s72">

<annotation>

<celldesigner:extension>

<celldesigner:alias>sa466</celldesigner:alias>

</celldesigner:extension>

</annotation>

</speciesReference>

</listOfReactants>

<listOfProducts>

<speciesReference metaid="CDMT00182" species="s2">

<annotation>

<celldesigner:extension>

<celldesigner:alias>sa143</celldesigner:alias>

</celldesigner:extension>

</annotation>

</speciesReference>

</listOfProducts>

</reaction>

<reaction metaid="re175" id="re175" name="arginase" reversible="false">

<annotation>

<celldesigner:extension>

<celldesigner:name>arginase</celldesigner:name>

<celldesigner:reactionType>STATE_TRANSITION</celldesigner:reactionType>

<celldesigner:baseReactants>

<celldesigner:baseReactant species="s90" alias="sa437"/>

</celldesigner:baseReactants>

<celldesigner:baseProducts>

<celldesigner:baseProduct species="s478" alias="sa438"/>

</celldesigner:baseProducts>

<celldesigner:listOfProductLinks>

<celldesigner:productLink product="s479" alias="sa439" targetLineIndex="-1,1">

<celldesigner:connectScheme connectPolicy="direct">

<celldesigner:listOfLineDirection>

<celldesigner:lineDirection index="0" value="unknown"/>

</celldesigner:listOfLineDirection>

</celldesigner:connectScheme>

<celldesigner:line width="1.0" color="ff000000" type="Straight"/>

</celldesigner:productLink>

</celldesigner:listOfProductLinks>

<celldesigner:connectScheme connectPolicy="direct" rectangleIndex="0">

<celldesigner:listOfLineDirection>

<celldesigner:lineDirection index="0" value="unknown"/>

</celldesigner:listOfLineDirection>

</celldesigner:connectScheme>

<celldesigner:line width="1.0" color="ff000000"/>

</celldesigner:extension>

</annotation>

<listOfReactants>

<speciesReference metaid="CDMT00183" species="s90">

<annotation>

<celldesigner:extension>

<celldesigner:alias>sa437</celldesigner:alias>

</celldesigner:extension>

</annotation>

</speciesReference>

</listOfReactants>

<listOfProducts>

<speciesReference metaid="CDMT00187" species="s478">

<annotation>

<celldesigner:extension>

<celldesigner:alias>sa438</celldesigner:alias>

</celldesigner:extension>

</annotation>

</speciesReference>

<speciesReference metaid="CDMT00189" species="s479">

<annotation>
<celldesigner:extension>
<celldesigner:alias>sa439</celldesigner:alias>
</celldesigner:extension>
</annotation>
</speciesReference>
</listOfProducts>
</reaction>
<reaction metaid="re362" id="re176" name="ODC" reversible="false">
<annotation>
<celldesigner:extension>
<celldesigner:name>ODC</celldesigner:name>
<celldesigner:reactionType>STATE_TRANSITION</celldesigner:reactionType>
<celldesigner:baseReactants>
<celldesigner:baseReactant species="s478" alias="sa438"/>
</celldesigner:baseReactants>
<celldesigner:baseProducts>
<celldesigner:baseProduct species="s481" alias="sa443"/>
</celldesigner:baseProducts>
<celldesigner:connectScheme connectPolicy="direct" rectangleIndex="0">
<celldesigner:listOfLineDirection>
<celldesigner:lineDirection index="0" value="unknown"/>
</celldesigner:listOfLineDirection>
</celldesigner:connectScheme>
<celldesigner:line width="1.0" color="ff000000"/>
</celldesigner:extension>
</annotation>

<listOfReactants>

<speciesReference metaid="CDMT00193" species="s478">

<annotation>

<celldesigner:extension>

<celldesigner:alias>sa438</celldesigner:alias>

</celldesigner:extension>

</annotation>

</speciesReference>

</listOfReactants>

<listOfProducts>

<speciesReference metaid="CDMT00201" species="s481">

<annotation>

<celldesigner:extension>

<celldesigner:alias>sa443</celldesigner:alias>

</celldesigner:extension>

</annotation>

</speciesReference>

</listOfProducts>

</reaction>

<reaction metaid="re363" id="re177" name="SAMS" reversible="false">

<annotation>

<celldesigner:extension>

<celldesigner:name>SAMS</celldesigner:name>

<celldesigner:reactionType>STATE_TRANSITION</celldesigner:reactionType>

<celldesigner:baseReactants>

<celldesigner:baseReactant species="s471" alias="sa440"/>

</celldesigner:baseReactants>

<celldesigner:baseProducts>

<celldesigner:baseProduct species="s108" alias="sa441"/>

</celldesigner:baseProducts>

<celldesigner:connectScheme connectPolicy="direct" rectangleIndex="0">

<celldesigner:listOfLineDirection>

<celldesigner:lineDirection index="0" value="unknown"/>

</celldesigner:listOfLineDirection>

</celldesigner:connectScheme>

<celldesigner:line width="1.0" color="ff000000"/>

</celldesigner:extension>

</annotation>

<listOfReactants>

<speciesReference metaid="CDMT00207" species="s471">

<annotation>

<celldesigner:extension>

<celldesigner:alias>sa440</celldesigner:alias>

</celldesigner:extension>

</annotation>

</speciesReference>

</listOfReactants>

<listOfProducts>

<speciesReference metaid="CDMT00208" species="s108">

<annotation>

<celldesigner:extension>

<celldesigner:alias>sa441</celldesigner:alias>

</celldesigner:extension>

</annotation>

</speciesReference>

</listOfProducts>

</reaction>

<reaction metaid="re364" id="re178" name="SAMDc" reversible="false">

<annotation>

<celldesigner:extension>

<celldesigner:name>SAMDc</celldesigner:name>

<celldesigner:reactionType>STATE_TRANSITION</celldesigner:reactionType>

<celldesigner:baseReactants>

<celldesigner:baseReactant species="s108" alias="sa441"/>

</celldesigner:baseReactants>

<celldesigner:baseProducts>

<celldesigner:baseProduct species="s480" alias="sa442"/>

</celldesigner:baseProducts>

<celldesigner:connectScheme connectPolicy="direct" rectangleIndex="0">

<celldesigner:listOfLineDirection>

<celldesigner:lineDirection index="0" value="unknown"/>

</celldesigner:listOfLineDirection>

</celldesigner:connectScheme>

<celldesigner:line width="1.0" color="ff000000"/>

</celldesigner:extension>

</annotation>

<listOfReactants>

<speciesReference metaid="CDMT00209" species="s108">

<annotation>

<celldesigner:extension>

<celldesigner:alias>sa441</celldesigner:alias>

</celldesigner:extension>

</annotation>

</speciesReference>

</listOfReactants>

<listOfProducts>

<speciesReference metaid="CDMT00210" species="s480">

<annotation>

<celldesigner:extension>

<celldesigner:alias>sa442</celldesigner:alias>

</celldesigner:extension>

</annotation>

</speciesReference>

</listOfProducts>

</reaction>

<reaction metaid="re365" id="re179" name="SpdS" reversible="false">

<annotation>

<celldesigner:extension>

<celldesigner:name>SpdS</celldesigner:name>

<celldesigner:reactionType>STATE_TRANSITION</celldesigner:reactionType>

<celldesigner:baseReactants>

<celldesigner:baseReactant species="s480" alias="sa442"/>

</celldesigner:baseReactants>

<celldesigner:baseProducts>

<celldesigner:baseProduct species="s485" alias="sa447"/>

</celldesigner:baseProducts>

<celldesigner:listOfReactantLinks>

<celldesigner:reactantLink reactant="s481" alias="sa443" targetLineIndex="-1,0">

<celldesigner:connectScheme connectPolicy="direct">

<celldesigner:listOfLineDirection>

<celldesigner:lineDirection index="0" value="unknown"/>

</celldesigner:listOfLineDirection>

</celldesigner:connectScheme>

<celldesigner:line width="1.0" color="ff000000" type="Straight"/>

</celldesigner:reactantLink>

</celldesigner:listOfReactantLinks>

<celldesigner:connectScheme connectPolicy="direct" rectangleIndex="0">

<celldesigner:listOfLineDirection>

<celldesigner:lineDirection index="0" value="unknown"/>

</celldesigner:listOfLineDirection>

</celldesigner:connectScheme>

<celldesigner:line width="1.0" color="ff000000"/>

</celldesigner:extension>

</annotation>

<listOfReactants>

<speciesReference metaid="CDMT00211" species="s480">

<annotation>

<celldesigner:extension>

<celldesigner:alias>sa442</celldesigner:alias>

</celldesigner:extension>

</annotation>

</speciesReference>

<speciesReference metaid="CDMT00212" species="s481">

<annotation>

<celldesigner:extension>

<celldesigner:alias>sa443</celldesigner:alias>

</celldesigner:extension>

</annotation>

</speciesReference>

</listOfReactants>

<listOfProducts>

<speciesReference metaid="CDMT00214" species="s485">

<annotation>

<celldesigner:extension>

<celldesigner:alias>sa447</celldesigner:alias>

</celldesigner:extension>

</annotation>

</speciesReference>

</listOfProducts>

</reaction>

<reaction metaid="re366" id="re180" name="GSH1" reversible="false">

<annotation>

<celldesigner:extension>

<celldesigner:name>GSH1</celldesigner:name>

<celldesigner:reactionType>STATE_TRANSITION</celldesigner:reactionType>

<celldesigner:baseReactants>

<celldesigner:baseReactant species="s115" alias="sa533">

<celldesigner:linkAnchor position="WSW"/>

</celldesigner:baseReactant>

</celldesigner:baseReactants>

<celldesigner:baseProducts>

<celldesigner:baseProduct species="s482" alias="sa444"/>

</celldesigner:baseProducts>

<celldesigner:listOfReactantLinks>

<celldesigner:reactantLink reactant="s475" alias="sa537" targetLineIndex="-1,0">

<celldesigner:connectScheme connectPolicy="direct">

<celldesigner:listOfLineDirection>

<celldesigner:lineDirection index="0" value="unknown"/>

<celldesigner:lineDirection index="1" value="unknown"/>

</celldesigner:listOfLineDirection>

</celldesigner:connectScheme>

<celldesigner:editPoints>0.9584159955331011,-0.06845058820548966</celldesigner:editPoints>

<celldesigner:line width="1.0" color="ff000000" type="Straight"/>

</celldesigner:reactantLink>

</celldesigner:listOfReactantLinks>

<celldesigner:connectScheme connectPolicy="direct" rectangleIndex="1">

<celldesigner:listOfLineDirection>

<celldesigner:lineDirection index="0" value="unknown"/>

<celldesigner:lineDirection index="1" value="unknown"/>

</celldesigner:listOfLineDirection>

</celldesigner:connectScheme>

<celldesigner:editPoints>0.9232615089458263,-0.02573673590006631</celldesigner:editPoints>

<celldesigner:line width="1.0" color="ff000000"/>

</celldesigner:extension>

</annotation>

<listOfReactants>

<speciesReference metaid="CDMT00215" species="s115">

<annotation>

<celldesigner:extension>

<celldesigner:alias>sa533</celldesigner:alias>

</celldesigner:extension>

</annotation>

</speciesReference>

<speciesReference metaid="CDMT00217" species="s475">

<annotation>

<celldesigner:extension>

<celldesigner:alias>sa537</celldesigner:alias>

</celldesigner:extension>

</annotation>

</speciesReference>

</listOfReactants>

<listOfProducts>

<speciesReference metaid="CDMT00218" species="s482">

<annotation>

<celldesigner:extension>

<celldesigner:alias>sa444</celldesigner:alias>

</celldesigner:extension>

</annotation>

</speciesReference>

</listOfProducts>

</reaction>

<reaction metaid="re367" id="re181" name="GSH2" reversible="false">

<annotation>

<celldesigner:extension>

<celldesigner:name>GSH2</celldesigner:name>

<celldesigner:reactionType>STATE_TRANSITION</celldesigner:reactionType>

<celldesigner:baseReactants>

<celldesigner:baseReactant species="s482" alias="sa444"/>

</celldesigner:baseReactants>

<celldesigner:baseProducts>

<celldesigner:baseProduct species="s483" alias="sa446"/>

</celldesigner:baseProducts>

<celldesigner:listOfReactantLinks>

<celldesigner:reactantLink reactant="s473" alias="sa536" targetLineIndex="-1,0">

<celldesigner:linkAnchor position="INACTIVE"/>

<celldesigner:connectScheme connectPolicy="direct">

<celldesigner:listOfLineDirection>

<celldesigner:lineDirection index="0" value="unknown"/>

</celldesigner:listOfLineDirection>

</celldesigner:connectScheme>

<celldesigner:line width="1.0" color="ff000000" type="Straight"/>

</celldesigner:reactantLink>

</celldesigner:listOfReactantLinks>

<celldesigner:connectScheme connectPolicy="direct" rectangleIndex="0">

<celldesigner:listOfLineDirection>

<celldesigner:lineDirection index="0" value="unknown"/>

</celldesigner:listOfLineDirection>

</celldesigner:connectScheme>

<celldesigner:line width="1.0" color="ff000000"/>

</celldesigner:extension>

</annotation>

<listOfReactants>

<speciesReference metaid="CDMT00219" species="s482">

<annotation>
<celldesigner:extension>
<celldesigner:alias>sa444</celldesigner:alias>
</celldesigner:extension>
</annotation>
</speciesReference>
<speciesReference metaid="CDMT00220" species="s473">
<annotation>
<celldesigner:extension>
<celldesigner:alias>sa536</celldesigner:alias>
</celldesigner:extension>
</annotation>
</speciesReference>
</listOfReactants>
<listOfProducts>
<speciesReference metaid="CDMT00221" species="s483">
<annotation>
<celldesigner:extension>
<celldesigner:alias>sa446</celldesigner:alias>
</celldesigner:extension>
</annotation>
</speciesReference>
</listOfProducts>
</reaction>
<reaction metaid="re368" id="re182" name="TryS(gspd Synthesis)" reversible="false">
<annotation>
<celldesigner:extension>

<celldesigner:name>TryS(gspd Synthesis)</celldesigner:name>
<celldesigner:reactionType>STATE_TRANSITION</celldesigner:reactionType>
<celldesigner:baseReactants>
<celldesigner:baseReactant species="s483" alias="sa446"/>
</celldesigner:baseReactants>
<celldesigner:baseProducts>
<celldesigner:baseProduct species="s486" alias="sa448"/>
</celldesigner:baseProducts>
<celldesigner:listOfReactantLinks>
<celldesigner:reactantLink reactant="s485" alias="sa447" targetLineIndex="-1,0">
<celldesigner:connectScheme connectPolicy="direct">
<celldesigner:listOfLineDirection>
<celldesigner:lineDirection index="0" value="unknown"/>
</celldesigner:listOfLineDirection>
</celldesigner:connectScheme>
<celldesigner:line width="1.0" color="ff000000" type="Straight"/>
</celldesigner:reactantLink>
</celldesigner:listOfReactantLinks>
<celldesigner:connectScheme connectPolicy="direct" rectangleIndex="0">
<celldesigner:listOfLineDirection>
<celldesigner:lineDirection index="0" value="unknown"/>
</celldesigner:listOfLineDirection>
</celldesigner:connectScheme>
<celldesigner:line width="1.0" color="ff000000"/>
</celldesigner:extension>
</annotation>
<listOfReactants>

<speciesReference metaid="CDMT00222" species="s483">
<annotation>
<celldesigner:extension>
<celldesigner:alias>sa446</celldesigner:alias>
</celldesigner:extension>
</annotation>
</speciesReference>
<speciesReference metaid="CDMT00223" species="s485">
<annotation>
<celldesigner:extension>
<celldesigner:alias>sa447</celldesigner:alias>
</celldesigner:extension>
</annotation>
</speciesReference>
</listOfReactants>
<listOfProducts>
<speciesReference metaid="CDMT00224" species="s486">
<annotation>
<celldesigner:extension>
<celldesigner:alias>sa448</celldesigner:alias>
</celldesigner:extension>
</annotation>
</speciesReference>
</listOfProducts>
</reaction>
<reaction metaid="re369" id="re183" name="TryS(tsh2 Synthesis)" reversible="false">
<annotation>

<celldesigner:extension>

<celldesigner:name>TryS(tsh2 Synthesis)</celldesigner:name>

<celldesigner:reactionType>STATE_TRANSITION</celldesigner:reactionType>

<celldesigner:baseReactants>

<celldesigner:baseReactant species="s486" alias="sa448"/>

</celldesigner:baseReactants>

<celldesigner:baseProducts>

<celldesigner:baseProduct species="s70" alias="sa458">

<celldesigner:linkAnchor position="SE"/>

</celldesigner:baseProduct>

</celldesigner:baseProducts>

<celldesigner:listOfReactantLinks>

<celldesigner:reactantLink reactant="s483" alias="sa446" targetLineIndex="-1,0">

<celldesigner:connectScheme connectPolicy="direct">

<celldesigner:listOfLineDirection>

<celldesigner:lineDirection index="0" value="unknown"/>

<celldesigner:lineDirection index="1" value="unknown"/>

</celldesigner:listOfLineDirection>

</celldesigner:connectScheme>

<celldesigner:editPoints>0.4540395348496409,-0.2532144614149914</celldesigner:editPoints>

<celldesigner:line width="1.0" color="ff000000" type="Straight"/>

</celldesigner:reactantLink>

</celldesigner:listOfReactantLinks>

<celldesigner:connectScheme connectPolicy="direct" rectangleIndex="0">

<celldesigner:listOfLineDirection>

<celldesigner:lineDirection index="0" value="unknown"/>

</celldesigner:listOfLineDirection>

</celldesigner:connectScheme>
<celldesigner:line width="1.0" color="ff000000"/>
</celldesigner:extension>
</annotation>
<listOfReactants>
<speciesReference metaid="CDMT00225" species="s486">
<annotation>
<celldesigner:extension>
<celldesigner:alias>sa448</celldesigner:alias>
</celldesigner:extension>
</annotation>
</speciesReference>
<speciesReference metaid="CDMT00226" species="s483">
<annotation>
<celldesigner:extension>
<celldesigner:alias>sa446</celldesigner:alias>
</celldesigner:extension>
</annotation>
</speciesReference>
</listOfReactants>
<listOfProducts>
<speciesReference metaid="CDMT00227" species="s70">
<annotation>
<celldesigner:extension>
<celldesigner:alias>sa458</celldesigner:alias>
</celldesigner:extension>
</annotation>

</speciesReference>

</listOfProducts>

</reaction>

<reaction metaid="re328" id="re184" name="TR" reversible="false">

<annotation>

<celldesigner:extension>

<celldesigner:name>TR</celldesigner:name>

<celldesigner:reactionType>STATE_TRANSITION</celldesigner:reactionType>

<celldesigner:baseReactants>

<celldesigner:baseReactant species="s306" alias="sa451"/>

</celldesigner:baseReactants>

<celldesigner:baseProducts>

<celldesigner:baseProduct species="s70" alias="sa458"/>

</celldesigner:baseProducts>

<celldesigner:connectScheme connectPolicy="direct" rectangleIndex="0">

<celldesigner:listOfLineDirection>

<celldesigner:lineDirection index="0" value="unknown"/>

</celldesigner:listOfLineDirection>

</celldesigner:connectScheme>

<celldesigner:line width="1.0" color="ff000000"/>

</celldesigner:extension>

</annotation>

<listOfReactants>

<speciesReference metaid="CDMT00228" species="s306">

<annotation>

<celldesigner:extension>

<celldesigner:alias>sa451</celldesigner:alias>

</celldesigner:extension>

</annotation>

</speciesReference>

</listOfReactants>

<listOfProducts>

<speciesReference metaid="CDMT00229" species="s70">

<annotation>

<celldesigner:extension>

<celldesigner:alias>sa458</celldesigner:alias>

</celldesigner:extension>

</annotation>

</speciesReference>

</listOfProducts>

</reaction>

<reaction metaid="re507" id="re185" reversible="false">

<annotation>

<celldesigner:extension>

<celldesigner:reactionType>STATE_TRANSITION</celldesigner:reactionType>

<celldesigner:baseReactants>

<celldesigner:baseReactant species="s59" alias="sa44"/>

</celldesigner:baseReactants>

<celldesigner:baseProducts>

<celldesigner:baseProduct species="s68" alias="sa456">

<celldesigner:linkAnchor position="W"/>

</celldesigner:baseProduct>

</celldesigner:baseProducts>

<celldesigner:connectScheme connectPolicy="direct" rectangleIndex="0">

<celldesigner:listOfLineDirection>

<celldesigner:lineDirection index="0" value="unknown"/>

</celldesigner:listOfLineDirection>

</celldesigner:connectScheme>

<celldesigner:line width="1.0" color="ff000000"/>

</celldesigner:extension>

</annotation>

<listOfReactants>

<speciesReference metaid="CDMT00230" species="s59">

<annotation>

<celldesigner:extension>

<celldesigner:alias>sa44</celldesigner:alias>

</celldesigner:extension>

</annotation>

</speciesReference>

</listOfReactants>

<listOfProducts>

<speciesReference metaid="CDMT00231" species="s68">

<annotation>

<celldesigner:extension>

<celldesigner:alias>sa456</celldesigner:alias>

</celldesigner:extension>

</annotation>

</speciesReference>

</listOfProducts>

</reaction>

<reaction metaid="re508" id="re186" reversible="false">

<annotation>
<celldesigner:extension>
<celldesigner:reactionType>STATE_TRANSITION</celldesigner:reactionType>
<celldesigner:baseReactants>
<celldesigner:baseReactant species="s68" alias="sa456">
<celldesigner:linkAnchor position="NE"/>
</celldesigner:baseReactant>
</celldesigner:baseReactants>
<celldesigner:baseProducts>
<celldesigner:baseProduct species="s60" alias="sa43">
<celldesigner:linkAnchor position="E"/>
</celldesigner:baseProduct>
</celldesigner:baseProducts>
<celldesigner:connectScheme connectPolicy="direct" rectangleIndex="0">
<celldesigner:listOfLineDirection>
<celldesigner:lineDirection index="0" value="unknown"/>
</celldesigner:listOfLineDirection>
</celldesigner:connectScheme>
<celldesigner:line width="1.0" color="ffff3333"/>
</celldesigner:extension>
</annotation>
<listOfReactants>
<speciesReference metaid="CDMT00232" species="s68">
<annotation>
<celldesigner:extension>
<celldesigner:alias>sa456</celldesigner:alias>
</celldesigner:extension>

</annotation>

</speciesReference>

</listOfReactants>

<listOfProducts>

<speciesReference metaid="CDMT00233" species="s60">

<annotation>

<celldesigner:extension>

<celldesigner:alias>sa43</celldesigner:alias>

</celldesigner:extension>

</annotation>

</speciesReference>

</listOfProducts>

</reaction>

<reaction metaid="re509" id="re187" reversible="false">

<annotation>

<celldesigner:extension>

<celldesigner:reactionType>STATE_TRANSITION</celldesigner:reactionType>

<celldesigner:baseReactants>

<celldesigner:baseReactant species="s68" alias="sa456">

<celldesigner:linkAnchor position="NW"/>

</celldesigner:baseReactant>

</celldesigner:baseReactants>

<celldesigner:baseProducts>

<celldesigner:baseProduct species="s59" alias="sa44">

<celldesigner:linkAnchor position="ENE"/>

</celldesigner:baseProduct>

</celldesigner:baseProducts>

<celldesigner:connectScheme connectPolicy="direct" rectangleIndex="0">

<celldesigner:listOfLineDirection>

<celldesigner:lineDirection index="0" value="unknown"/>

</celldesigner:listOfLineDirection>

</celldesigner:connectScheme>

<celldesigner:line width="1.0" color="ffff3333"/>

</celldesigner:extension>

</annotation>

<listOfReactants>

<speciesReference metaid="CDMT00234" species="s68">

<annotation>

<celldesigner:extension>

<celldesigner:alias>sa456</celldesigner:alias>

</celldesigner:extension>

</annotation>

</speciesReference>

</listOfReactants>

<listOfProducts>

<speciesReference metaid="CDMT00235" species="s59">

<annotation>

<celldesigner:extension>

<celldesigner:alias>sa44</celldesigner:alias>

</celldesigner:extension>

</annotation>

</speciesReference>

</listOfProducts>

</reaction>

<reaction metaid="re510" id="re188" reversible="false">
<annotation>
<celldesigner:extension>
<celldesigner:reactionType>STATE_TRANSITION</celldesigner:reactionType>
<celldesigner:baseReactants>
<celldesigner:baseReactant species="s69" alias="sa459">
<celldesigner:linkAnchor position="NNW"/>
</celldesigner:baseReactant>
</celldesigner:baseReactants>
<celldesigner:baseProducts>
<celldesigner:baseProduct species="s68" alias="sa456">
<celldesigner:linkAnchor position="SSW"/>
</celldesigner:baseProduct>
</celldesigner:baseProducts>
<celldesigner:listOfProductLinks>
<celldesigner:productLink product="s70" alias="sa458" targetLineIndex="-1,1">
<celldesigner:linkAnchor position="NW"/>
<celldesigner:connectScheme connectPolicy="direct">
<celldesigner:listOfLineDirection>
<celldesigner:lineDirection index="0" value="unknown"/>
<celldesigner:lineDirection index="1" value="unknown"/>
</celldesigner:listOfLineDirection>
</celldesigner:connectScheme>
<celldesigner:editPoints>0.4704213247832989,-0.3038288575565984</celldesigner:editPoints>
<celldesigner:line width="1.0" color="ffff3333" type="Straight"/>
</celldesigner:productLink>
</celldesigner:listOfProductLinks>

```
<celldesigner:connectScheme connectPolicy="direct" rectangleIndex="0">
<celldesigner:listOfLineDirection>
<celldesigner:lineDirection index="0" value="unknown"/>
</celldesigner:listOfLineDirection>
</celldesigner:connectScheme>
<celldesigner:line width="1.0" color="ffff3333"/>
</celldesigner:extension>
</annotation>
<listOfReactants>
<speciesReference metaid="CDMT00236" species="s69">
<annotation>
<celldesigner:extension>
<celldesigner:alias>sa459</celldesigner:alias>
</celldesigner:extension>
</annotation>
</speciesReference>
</listOfReactants>
<listOfProducts>
<speciesReference metaid="CDMT00237" species="s68">
<annotation>
<celldesigner:extension>
<celldesigner:alias>sa456</celldesigner:alias>
</celldesigner:extension>
</annotation>
</speciesReference>
<speciesReference metaid="CDMT00238" species="s70">
<annotation>
```

<celldesigner:extension>
<celldesigner:alias>sa458</celldesigner:alias>
</celldesigner:extension>
</annotation>
</speciesReference>
</listOfProducts>
</reaction>
<reaction metaid="re511" id="re189" name="MGR" reversible="false">
<annotation>
<celldesigner:extension>
<celldesigner:name>MGR</celldesigner:name>
<celldesigner:reactionType>STATE_TRANSITION</celldesigner:reactionType>
<celldesigner:baseReactants>
<celldesigner:baseReactant species="s68" alias="sa456">
<celldesigner:linkAnchor position="SW"/>
</celldesigner:baseReactant>
</celldesigner:baseReactants>
<celldesigner:baseProducts>
<celldesigner:baseProduct species="s487" alias="sa467"/>
</celldesigner:baseProducts>
<celldesigner:connectScheme connectPolicy="direct" rectangleIndex="0">
<celldesigner:listOfLineDirection>
<celldesigner:lineDirection index="0" value="unknown"/>
</celldesigner:listOfLineDirection>
</celldesigner:connectScheme>
<celldesigner:line width="1.0" color="ff000000"/>
</celldesigner:extension>

</annotation>

<listOfReactants>

<speciesReference metaid="CDMT00239" species="s68">

<annotation>

<celldesigner:extension>

<celldesigner:alias>sa456</celldesigner:alias>

</celldesigner:extension>

</annotation>

</speciesReference>

</listOfReactants>

<listOfProducts>

<speciesReference metaid="CDMT00240" species="s487">

<annotation>

<celldesigner:extension>

<celldesigner:alias>sa467</celldesigner:alias>

</celldesigner:extension>

</annotation>

</speciesReference>

</listOfProducts>

</reaction>

<reaction metaid="re512" id="re190" reversible="false">

<annotation>

<celldesigner:extension>

<celldesigner:reactionType>STATE_TRANSITION</celldesigner:reactionType>

<celldesigner:baseReactants>

<celldesigner:baseReactant species="s487" alias="sa467"/>

</celldesigner:baseReactants>

<celldesigner:baseProducts>

<celldesigner:baseProduct species="s488" alias="sa468"/>

</celldesigner:baseProducts>

<celldesigner:connectScheme connectPolicy="direct" rectangleIndex="0">

<celldesigner:listOfLineDirection>

<celldesigner:lineDirection index="0" value="unknown"/>

</celldesigner:listOfLineDirection>

</celldesigner:connectScheme>

<celldesigner:line width="1.0" color="ff000000"/>

</celldesigner:extension>

</annotation>

<listOfReactants>

<speciesReference metaid="CDMT00241" species="s487">

<annotation>

<celldesigner:extension>

<celldesigner:alias>sa467</celldesigner:alias>

</celldesigner:extension>

</annotation>

</speciesReference>

</listOfReactants>

<listOfProducts>

<speciesReference metaid="CDMT00242" species="s488">

<annotation>

<celldesigner:extension>

<celldesigner:alias>sa468</celldesigner:alias>

</celldesigner:extension>

</annotation>

</speciesReference>

</listOfProducts>

</reaction>

<reaction metaid="re68" id="re191" name="ACoAC" reversible="false">

<annotation>

<celldesigner:extension>

<celldesigner:name>ACoAC</celldesigner:name>

<celldesigner:reactionType>STATE_TRANSITION</celldesigner:reactionType>

<celldesigner:baseReactants>

<celldesigner:baseReactant species="s7" alias="sa247">

<celldesigner:linkAnchor position="ENE"/>

</celldesigner:baseReactant>

</celldesigner:baseReactants>

<celldesigner:baseProducts>

<celldesigner:baseProduct species="s1091" alias="sa470"/>

</celldesigner:baseProducts>

<celldesigner:connectScheme connectPolicy="direct" rectangleIndex="1">

<celldesigner:listOfLineDirection>

<celldesigner:lineDirection index="0" value="unknown"/>

<celldesigner:lineDirection index="1" value="unknown"/>

<celldesigner:lineDirection index="2" value="unknown"/>

<celldesigner:lineDirection index="3" value="unknown"/>

</celldesigner:listOfLineDirection>

</celldesigner:connectScheme>

<celldesigner:editPoints>0.11895950717092353,0.0311328939823452 0.48231620175935935,-0.3209492946769197 0.9395642577435441,-0.08050846936470535</celldesigner:editPoints>

<celldesigner:line width="1.0" color="ff00ffff"/>

</celldesigner:extension>

</annotation>
<listOfReactants>
<speciesReference metaid="CDMT00247" species="s7">
<annotation>
<celldesigner:extension>
<celldesigner:alias>sa247</celldesigner:alias>
</celldesigner:extension>
</annotation>
</speciesReference>
</listOfReactants>
<listOfProducts>
<speciesReference metaid="CDMT00248" species="s1091">
<annotation>
<celldesigner:extension>
<celldesigner:alias>sa470</celldesigner:alias>
</celldesigner:extension>
</annotation>
</speciesReference>
</listOfProducts>
</reaction>
<reaction metaid="re70" id="re192" name="FAS60CoAr" reversible="false">
<annotation>
<celldesigner:extension>
<celldesigner:name>FAS60CoAr</celldesigner:name>
<celldesigner:reactionType>STATE_TRANSITION</celldesigner:reactionType>
<celldesigner:baseReactants>
<celldesigner:baseReactant species="s1091" alias="sa470">

<celldesigner:linkAnchor position="S"/>

</celldesigner:baseReactant>

</celldesigner:baseReactants>

<celldesigner:baseProducts>

<celldesigner:baseProduct species="s437" alias="sa472"/>

</celldesigner:baseProducts>

<celldesigner:listOfReactantLinks>

<celldesigner:reactantLink reactant="s436" alias="sa471" targetLineIndex="-1,0">

<celldesigner:connectScheme connectPolicy="direct">

<celldesigner:listOfLineDirection>

<celldesigner:lineDirection index="0" value="unknown"/>

</celldesigner:listOfLineDirection>

</celldesigner:connectScheme>

<celldesigner:line width="1.0" color="ffff0000" type="Straight"/>

</celldesigner:reactantLink>

</celldesigner:listOfReactantLinks>

<celldesigner:connectScheme connectPolicy="direct" rectangleIndex="0">

<celldesigner:listOfLineDirection>

<celldesigner:lineDirection index="0" value="unknown"/>

</celldesigner:listOfLineDirection>

</celldesigner:connectScheme>

<celldesigner:line width="1.0" color="ffff0000"/>

</celldesigner:extension>

</annotation>

<listOfReactants>

<speciesReference metaid="CDMT00249" species="s1091">

<annotation>

<celldesigner:extension>

<celldesigner:alias>sa470</celldesigner:alias>

</celldesigner:extension>

</annotation>

</speciesReference>

<speciesReference metaid="CDMT00250" species="s436">

<annotation>

<celldesigner:extension>

<celldesigner:alias>sa471</celldesigner:alias>

</celldesigner:extension>

</annotation>

</speciesReference>

</listOfReactants>

<listOfProducts>

<speciesReference metaid="CDMT00251" species="s437">

<annotation>

<celldesigner:extension>

<celldesigner:alias>sa472</celldesigner:alias>

</celldesigner:extension>

</annotation>

</speciesReference>

</listOfProducts>

</reaction>

<reaction metaid="re71" id="re193" name="FAS80CoAr" reversible="false">

<annotation>

<celldesigner:extension>

<celldesigner:name>FAS80CoAr</celldesigner:name>

<celldesigner:reactionType>STATE_TRANSITION</celldesigner:reactionType>

<celldesigner:baseReactants>

<celldesigner:baseReactant species="s437" alias="sa472">

<celldesigner:linkAnchor position="S"/>

</celldesigner:baseReactant>

</celldesigner:baseReactants>

<celldesigner:baseProducts>

<celldesigner:baseProduct species="s713" alias="sa473"/>

</celldesigner:baseProducts>

<celldesigner:listOfReactantLinks>

<celldesigner:reactantLink reactant="s1091" alias="sa470" targetLineIndex="-1,0">

<celldesigner:linkAnchor position="WSW"/>

<celldesigner:connectScheme connectPolicy="direct">

<celldesigner:listOfLineDirection>

<celldesigner:lineDirection index="0" value="unknown"/>

<celldesigner:lineDirection index="1" value="unknown"/>

</celldesigner:listOfLineDirection>

</celldesigner:connectScheme>

<celldesigner:editPoints>1.0072639157913672,0.2609072641449419</celldesigner:editPoints>

<celldesigner:line width="1.0" color="ffff0000" type="Straight"/>

</celldesigner:reactantLink>

</celldesigner:listOfReactantLinks>

<celldesigner:connectScheme connectPolicy="direct" rectangleIndex="0">

<celldesigner:listOfLineDirection>

<celldesigner:lineDirection index="0" value="unknown"/>

</celldesigner:listOfLineDirection>

</celldesigner:connectScheme>

<celldesigner:line width="1.0" color="ffff0000"/>

</celldesigner:extension>

</annotation>

<listOfReactants>

<speciesReference metaid="CDMT00252" species="s437">

<annotation>

<celldesigner:extension>

<celldesigner:alias>sa472</celldesigner:alias>

</celldesigner:extension>

</annotation>

</speciesReference>

<speciesReference metaid="CDMT00253" species="s1091">

<annotation>

<celldesigner:extension>

<celldesigner:alias>sa470</celldesigner:alias>

</celldesigner:extension>

</annotation>

</speciesReference>

</listOfReactants>

<listOfProducts>

<speciesReference metaid="CDMT00254" species="s713">

<annotation>

<celldesigner:extension>

<celldesigner:alias>sa473</celldesigner:alias>

</celldesigner:extension>

</annotation>

</speciesReference>

</listOfProducts>

</reaction>

<reaction metaid="re72" id="re194" name="FAS100CoAr" reversible="false">

<annotation>

<celldesigner:extension>

<celldesigner:name>FAS100CoAr</celldesigner:name>

<celldesigner:reactionType>STATE_TRANSITION</celldesigner:reactionType>

<celldesigner:baseReactants>

<celldesigner:baseReactant species="s713" alias="sa473">

<celldesigner:linkAnchor position="S"/>

</celldesigner:baseReactant>

</celldesigner:baseReactants>

<celldesigner:baseProducts>

<celldesigner:baseProduct species="s714" alias="sa474">

<celldesigner:linkAnchor position="N"/>

</celldesigner:baseProduct>

</celldesigner:baseProducts>

<celldesigner:listOfReactantLinks>

<celldesigner:reactantLink reactant="s1091" alias="sa470" targetLineIndex="-1,0">

<celldesigner:linkAnchor position="WSW"/>

<celldesigner:connectScheme connectPolicy="direct">

<celldesigner:listOfLineDirection>

<celldesigner:lineDirection index="0" value="unknown"/>

<celldesigner:lineDirection index="1" value="unknown"/>

</celldesigner:listOfLineDirection>

</celldesigner:connectScheme>

<celldesigner:editPoints>0.9838157351493995,0.22427511739705785</celldesigner:editPoints>

<celldesigner:line width="1.0" color="ffff0000" type="Straight"/>

</celldesigner:reactantLink>

</celldesigner:listOfReactantLinks>

<celldesigner:connectScheme connectPolicy="direct" rectangleIndex="0">

<celldesigner:listOfLineDirection>

<celldesigner:lineDirection index="0" value="unknown"/>

</celldesigner:listOfLineDirection>

</celldesigner:connectScheme>

<celldesigner:line width="1.0" color="ffff0000"/>

</celldesigner:extension>

</annotation>

<listOfReactants>

<speciesReference metaid="CDMT00255" species="s713">

<annotation>

<celldesigner:extension>

<celldesigner:alias>sa473</celldesigner:alias>

</celldesigner:extension>

</annotation>

</speciesReference>

<speciesReference metaid="CDMT00256" species="s1091">

<annotation>

<celldesigner:extension>

<celldesigner:alias>sa470</celldesigner:alias>

</celldesigner:extension>

</annotation>

</speciesReference>

</listOfReactants>

<listOfProducts>

<speciesReference metaid="CDMT00257" species="s714">

<annotation>

<celldesigner:extension>

<celldesigner:alias>sa474</celldesigner:alias>

</celldesigner:extension>

</annotation>

</speciesReference>

</listOfProducts>

</reaction>

<reaction metaid="re73" id="re195" name="FAS120CoAr" reversible="false">

<annotation>

<celldesigner:extension>

<celldesigner:name>FAS120CoAr</celldesigner:name>

<celldesigner:reactionType>STATE_TRANSITION</celldesigner:reactionType>

<celldesigner:baseReactants>

<celldesigner:baseReactant species="s714" alias="sa474">

<celldesigner:linkAnchor position="S"/>

</celldesigner:baseReactant>

</celldesigner:baseReactants>

<celldesigner:baseProducts>

<celldesigner:baseProduct species="s717" alias="sa475">

<celldesigner:linkAnchor position="N"/>

</celldesigner:baseProduct>

</celldesigner:baseProducts>

<celldesigner:listOfReactantLinks>

<celldesigner:reactantLink reactant="s1091" alias="sa470" targetLineIndex="-1,0">

<celldesigner:linkAnchor position="WSW"/>

<celldesigner:connectScheme connectPolicy="direct">

<celldesigner:listOfLineDirection>

<celldesigner:lineDirection index="0" value="unknown"/>

<celldesigner:lineDirection index="1" value="unknown"/>

</celldesigner:listOfLineDirection>

</celldesigner:connectScheme>

<celldesigner:editPoints>1.032982111828599,0.2665011084248423</celldesigner:editPoints>

<celldesigner:line width="1.0" color="ffff0000" type="Straight"/>

</celldesigner:reactantLink>

</celldesigner:listOfReactantLinks>

<celldesigner:connectScheme connectPolicy="direct" rectangleIndex="0">

<celldesigner:listOfLineDirection>

<celldesigner:lineDirection index="0" value="unknown"/>

</celldesigner:listOfLineDirection>

</celldesigner:connectScheme>

<celldesigner:line width="1.0" color="ffff0000"/>

</celldesigner:extension>

</annotation>

<listOfReactants>

<speciesReference metaid="CDMT00258" species="s714">

<annotation>

<celldesigner:extension>

<celldesigner:alias>sa474</celldesigner:alias>

</celldesigner:extension>

</annotation>

</speciesReference>

<speciesReference metaid="CDMT00259" species="s1091">
<annotation>
<celldesigner:extension>
<celldesigner:alias>sa470</celldesigner:alias>
</celldesigner:extension>
</annotation>
</speciesReference>
</listOfReactants>
<listOfProducts>
<speciesReference metaid="CDMT00260" species="s717">
<annotation>
<celldesigner:extension>
<celldesigner:alias>sa475</celldesigner:alias>
</celldesigner:extension>
</annotation>
</speciesReference>
</listOfProducts>
</reaction>
<reaction metaid="re74" id="re196" name="FAS140CoAr" reversible="false">
<annotation>
<celldesigner:extension>
<celldesigner:name>FAS140CoAr</celldesigner:name>
<celldesigner:reactionType>STATE_TRANSITION</celldesigner:reactionType>
<celldesigner:baseReactants>
<celldesigner:baseReactant species="s717" alias="sa475">
<celldesigner:linkAnchor position="S"/>
</celldesigner:baseReactant>

</celldesigner:baseReactants>

<celldesigner:baseProducts>

<celldesigner:baseProduct species="s438" alias="sa476">

<celldesigner:linkAnchor position="N"/>

</celldesigner:baseProduct>

</celldesigner:baseProducts>

<celldesigner:listOfReactantLinks>

<celldesigner:reactantLink reactant="s1091" alias="sa470" targetLineIndex="-1,0">

<celldesigner:linkAnchor position="W"/>

<celldesigner:connectScheme connectPolicy="direct">

<celldesigner:listOfLineDirection>

<celldesigner:lineDirection index="0" value="unknown"/>

<celldesigner:lineDirection index="1" value="unknown"/>

</celldesigner:listOfLineDirection>

</celldesigner:connectScheme>

<celldesigner:editPoints>1.0456704716208223,0.2769766596004981</celldesigner:editPoints>

<celldesigner:line width="1.0" color="ffff0000" type="Straight"/>

</celldesigner:reactantLink>

</celldesigner:listOfReactantLinks>

<celldesigner:connectScheme connectPolicy="direct" rectangleIndex="0">

<celldesigner:listOfLineDirection>

<celldesigner:lineDirection index="0" value="unknown"/>

</celldesigner:listOfLineDirection>

</celldesigner:connectScheme>

<celldesigner:line width="1.0" color="ffff0000"/>

</celldesigner:extension>

</annotation>

<listOfReactants>

<speciesReference metaid="CDMT00261" species="s717">

<annotation>

<celldesigner:extension>

<celldesigner:alias>sa475</celldesigner:alias>

</celldesigner:extension>

</annotation>

</speciesReference>

<speciesReference metaid="CDMT00262" species="s1091">

<annotation>

<celldesigner:extension>

<celldesigner:alias>sa470</celldesigner:alias>

</celldesigner:extension>

</annotation>

</speciesReference>

</listOfReactants>

<listOfProducts>

<speciesReference metaid="CDMT00263" species="s438">

<annotation>

<celldesigner:extension>

<celldesigner:alias>sa476</celldesigner:alias>

</celldesigner:extension>

</annotation>

</speciesReference>

</listOfProducts>

</reaction>

<reaction metaid="re75" id="re197" name="FAS160CoAr" reversible="false">

<annotation>

<celldesigner:extension>

<celldesigner:name>FAS160CoAr</celldesigner:name>

<celldesigner:reactionType>STATE_TRANSITION</celldesigner:reactionType>

<celldesigner:baseReactants>

<celldesigner:baseReactant species="s438" alias="sa476"/>

</celldesigner:baseReactants>

<celldesigner:baseProducts>

<celldesigner:baseProduct species="s1098" alias="sa477"/>

</celldesigner:baseProducts>

<celldesigner:listOfReactantLinks>

<celldesigner:reactantLink reactant="s1091" alias="sa470" targetLineIndex="-1,0">

<celldesigner:linkAnchor position="WSW"/>

<celldesigner:connectScheme connectPolicy="direct">

<celldesigner:listOfLineDirection>

<celldesigner:lineDirection index="0" value="unknown"/>

<celldesigner:lineDirection index="1" value="unknown"/>

</celldesigner:listOfLineDirection>

</celldesigner:connectScheme>

<celldesigner:editPoints>0.9749853857710012,0.30538591936903803</celldesigner:editPoints>

<celldesigner:line width="1.0" color="ffff0000" type="Straight"/>

</celldesigner:reactantLink>

</celldesigner:listOfReactantLinks>

<celldesigner:connectScheme connectPolicy="direct" rectangleIndex="0">

<celldesigner:listOfLineDirection>

<celldesigner:lineDirection index="0" value="unknown"/>

</celldesigner:listOfLineDirection>

</celldesigner:connectScheme>

<celldesigner:line width="1.0" color="ffff0000"/>

</celldesigner:extension>

</annotation>

<listOfReactants>

<speciesReference metaid="CDMT00264" species="s438">

<annotation>

<celldesigner:extension>

<celldesigner:alias>sa476</celldesigner:alias>

</celldesigner:extension>

</annotation>

</speciesReference>

<speciesReference metaid="CDMT00265" species="s1091">

<annotation>

<celldesigner:extension>

<celldesigner:alias>sa470</celldesigner:alias>

</celldesigner:extension>

</annotation>

</speciesReference>

</listOfReactants>

<listOfProducts>

<speciesReference metaid="CDMT00266" species="s1098">

<annotation>

<celldesigner:extension>

<celldesigner:alias>sa477</celldesigner:alias>

</celldesigner:extension>

</annotation>

</speciesReference>
</listOfProducts>
</reaction>
<reaction metaid="re198" id="re198" name="FAS180CoAr" reversible="false">
<annotation>
<celldesigner:extension>
<celldesigner:name>FAS180CoAr</celldesigner:name>
<celldesigner:reactionType>STATE_TRANSITION</celldesigner:reactionType>
<celldesigner:baseReactants>
<celldesigner:baseReactant species="s1098" alias="sa477"/>
</celldesigner:baseReactants>
<celldesigner:baseProducts>
<celldesigner:baseProduct species="s1097" alias="sa478"/>
</celldesigner:baseProducts>
<celldesigner:listOfReactantLinks>
<celldesigner:reactantLink reactant="s1091" alias="sa470" targetLineIndex="-1,0">
<celldesigner:linkAnchor position="W"/>
<celldesigner:connectScheme connectPolicy="direct">
<celldesigner:listOfLineDirection>
<celldesigner:lineDirection index="0" value="unknown"/>
<celldesigner:lineDirection index="1" value="unknown"/>
</celldesigner:listOfLineDirection>
</celldesigner:connectScheme>
<celldesigner:editPoints>0.9565704062397681,0.3506931667448989</celldesigner:editPoints>
<celldesigner:line width="1.0" color="ffff0000" type="Straight"/>
</celldesigner:reactantLink>
</celldesigner:listOfReactantLinks>

<celldesigner:connectScheme connectPolicy="direct" rectangleIndex="0">
<celldesigner:listOfLineDirection>
<celldesigner:lineDirection index="0" value="unknown"/>
</celldesigner:listOfLineDirection>
</celldesigner:connectScheme>
<celldesigner:line width="1.0" color="ffff0000"/>
</celldesigner:extension>
</annotation>
<listOfReactants>
<speciesReference metaid="CDMT00267" species="s1098">
<annotation>
<celldesigner:extension>
<celldesigner:alias>sa477</celldesigner:alias>
</celldesigner:extension>
</annotation>
</speciesReference>
<speciesReference metaid="CDMT00268" species="s1091">
<annotation>
<celldesigner:extension>
<celldesigner:alias>sa470</celldesigner:alias>
</celldesigner:extension>
</annotation>
</speciesReference>
</listOfReactants>
<listOfProducts>
<speciesReference metaid="CDMT00269" species="s1097">
<annotation>

<celldesigner:extension>
<celldesigner:alias>sa478</celldesigner:alias>
</celldesigner:extension>
</annotation>
</speciesReference>
</listOfProducts>
</reaction>
<reaction metaid="re199" id="re199" name="DESAT181" reversible="false">
<annotation>
<celldesigner:extension>
<celldesigner:name>DESAT181</celldesigner:name>
<celldesigner:reactionType>STATE_TRANSITION</celldesigner:reactionType>
<celldesigner:baseReactants>
<celldesigner:baseReactant species="s1097" alias="sa478"/>
</celldesigner:baseReactants>
<celldesigner:baseProducts>
<celldesigner:baseProduct species="s1102" alias="sa479">
<celldesigner:linkAnchor position="N"/>
</celldesigner:baseProduct>
</celldesigner:baseProducts>
<celldesigner:connectScheme connectPolicy="direct" rectangleIndex="0">
<celldesigner:listOfLineDirection>
<celldesigner:lineDirection index="0" value="unknown"/>
</celldesigner:listOfLineDirection>
</celldesigner:connectScheme>
<celldesigner:line width="1.0" color="ffff0000"/>
</celldesigner:extension>

</annotation>
<listOfReactants>
<speciesReference metaid="CDMT00270" species="s1097">
<annotation>
<celldesigner:extension>
<celldesigner:alias>sa478</celldesigner:alias>
</celldesigner:extension>
</annotation>
</speciesReference>
</listOfReactants>
<listOfProducts>
<speciesReference metaid="CDMT00271" species="s1102">
<annotation>
<celldesigner:extension>
<celldesigner:alias>sa479</celldesigner:alias>
</celldesigner:extension>
</annotation>
</speciesReference>
</listOfProducts>
</reaction>
<reaction metaid="re200" id="re200" name="DESAT182" reversible="false">
<annotation>
<celldesigner:extension>
<celldesigner:name>DESAT182</celldesigner:name>
<celldesigner:reactionType>STATE_TRANSITION</celldesigner:reactionType>
<celldesigner:baseReactants>
<celldesigner:baseReactant species="s1102" alias="sa479">

<celldesigner:linkAnchor position="S"/>

</celldesigner:baseReactant>

</celldesigner:baseReactants>

<celldesigner:baseProducts>

<celldesigner:baseProduct species="s439" alias="sa480">

<celldesigner:linkAnchor position="N"/>

</celldesigner:baseProduct>

</celldesigner:baseProducts>

<celldesigner:connectScheme connectPolicy="direct" rectangleIndex="0">

<celldesigner:listOfLineDirection>

<celldesigner:lineDirection index="0" value="unknown"/>

</celldesigner:listOfLineDirection>

</celldesigner:connectScheme>

<celldesigner:line width="1.0" color="ff000000"/>

</celldesigner:extension>

</annotation>

<listOfReactants>

<speciesReference metaid="CDMT00272" species="s1102">

<annotation>

<celldesigner:extension>

<celldesigner:alias>sa479</celldesigner:alias>

</celldesigner:extension>

</annotation>

</speciesReference>

</listOfReactants>

<listOfProducts>

<speciesReference metaid="CDMT00273" species="s439">

<annotation>
<celldesigner:extension>
<celldesigner:alias>sa480</celldesigner:alias>
</celldesigner:extension>
</annotation>
</speciesReference>
</listOfProducts>
</reaction>
<reaction metaid="re201" id="re201" name="DESAT183" reversible="false">
<annotation>
<celldesigner:extension>
<celldesigner:name>DESAT183</celldesigner:name>
<celldesigner:reactionType>STATE_TRANSITION</celldesigner:reactionType>
<celldesigner:baseReactants>
<celldesigner:baseReactant species="s439" alias="sa480">
<celldesigner:linkAnchor position="S"/>
</celldesigner:baseReactant>
</celldesigner:baseReactants>
<celldesigner:baseProducts>
<celldesigner:baseProduct species="s1104" alias="sa481"/>
</celldesigner:baseProducts>
<celldesigner:connectScheme connectPolicy="direct" rectangleIndex="0">
<celldesigner:listOfLineDirection>
<celldesigner:lineDirection index="0" value="unknown"/>
</celldesigner:listOfLineDirection>
</celldesigner:connectScheme>
<celldesigner:line width="1.0" color="ffff0000"/>

</celldesigner:extension>
</annotation>
<listOfReactants>
<speciesReference metaid="CDMT00274" species="s439">
<annotation>
<celldesigner:extension>
<celldesigner:alias>sa480</celldesigner:alias>
</celldesigner:extension>
</annotation>
</speciesReference>
</listOfReactants>
<listOfProducts>
<speciesReference metaid="CDMT00275" species="s1104">
<annotation>
<celldesigner:extension>
<celldesigner:alias>sa481</celldesigner:alias>
</celldesigner:extension>
</annotation>
</speciesReference>
</listOfProducts>
</reaction>
<reaction metaid="re80" id="re202" name="DESAT183" reversible="false">
<annotation>
<celldesigner:extension>
<celldesigner:name>DESAT183</celldesigner:name>
<celldesigner:reactionType>STATE_TRANSITION</celldesigner:reactionType>
<celldesigner:baseReactants>

<celldesigner:baseReactant species="s1104" alias="sa481">

<celldesigner:linkAnchor position="S"/>

</celldesigner:baseReactant>

</celldesigner:baseReactants>

<celldesigner:baseProducts>

<celldesigner:baseProduct species="s1103" alias="sa483">

<celldesigner:linkAnchor position="N"/>

</celldesigner:baseProduct>

</celldesigner:baseProducts>

<celldesigner:connectScheme connectPolicy="direct" rectangleIndex="0">

<celldesigner:listOfLineDirection>

<celldesigner:lineDirection index="0" value="unknown"/>

</celldesigner:listOfLineDirection>

</celldesigner:connectScheme>

<celldesigner:line width="1.0" color="ffff0000"/>

</celldesigner:extension>

</annotation>

<listOfReactants>

<speciesReference metaid="CDMT00276" species="s1104">

<annotation>

<celldesigner:extension>

<celldesigner:alias>sa481</celldesigner:alias>

</celldesigner:extension>

</annotation>

</speciesReference>

</listOfReactants>

<listOfProducts>

<speciesReference metaid="CDMT00277" species="s1103">
<annotation>
<celldesigner:extension>
<celldesigner:alias>sa483</celldesigner:alias>
</celldesigner:extension>
</annotation>
</speciesReference>
</listOfProducts>
</reaction>
<reaction metaid="re81" id="re203" name="DESAT184" reversible="false">
<annotation>
<celldesigner:extension>
<celldesigner:name>DESAT184</celldesigner:name>
<celldesigner:reactionType>STATE_TRANSITION</celldesigner:reactionType>
<celldesigner:baseReactants>
<celldesigner:baseReactant species="s1103" alias="sa483">
<celldesigner:linkAnchor position="S"/>
</celldesigner:baseReactant>
</celldesigner:baseReactants>
<celldesigner:baseProducts>
<celldesigner:baseProduct species="s96" alias="sa485">
<celldesigner:linkAnchor position="N"/>
</celldesigner:baseProduct>
</celldesigner:baseProducts>
<celldesigner:connectScheme connectPolicy="direct" rectangleIndex="0">
<celldesigner:listOfLineDirection>
<celldesigner:lineDirection index="0" value="unknown"/>

</celldesigner:listOfLineDirection>
</celldesigner:connectScheme>
<celldesigner:line width="1.0" color="ffff0000"/>
</celldesigner:extension>
</annotation>
<listOfReactants>
<speciesReference metaid="CDMT00278" species="s1103">
<annotation>
<celldesigner:extension>
<celldesigner:alias>sa483</celldesigner:alias>
</celldesigner:extension>
</annotation>
</speciesReference>
</listOfReactants>
<listOfProducts>
<speciesReference metaid="CDMT00279" species="s96">
<annotation>
<celldesigner:extension>
<celldesigner:alias>sa485</celldesigner:alias>
</celldesigner:extension>
</annotation>
</speciesReference>
</listOfProducts>
</reaction>
<reaction metaid="re383" id="re204" name="DESAT204" reversible="false">
<annotation>
<celldesigner:extension>

<celldesigner:name>DESAT204</celldesigner:name>

<celldesigner:reactionType>STATE_TRANSITION</celldesigner:reactionType>

<celldesigner:baseReactants>

<celldesigner:baseReactant species="s1103" alias="sa483">

<celldesigner:linkAnchor position="ENE"/>

</celldesigner:baseReactant>

</celldesigner:baseReactants>

<celldesigner:baseProducts>

<celldesigner:baseProduct species="s730" alias="sa482">

<celldesigner:linkAnchor position="WNW"/>

</celldesigner:baseProduct>

</celldesigner:baseProducts>

<celldesigner:listOfReactantLinks>

<celldesigner:reactantLink reactant="s1091" alias="sa470" targetLineIndex="-1,0">

<celldesigner:linkAnchor position="ENE"/>

<celldesigner:connectScheme connectPolicy="direct">

<celldesigner:listOfLineDirection>

<celldesigner:lineDirection index="0" value="unknown"/>

<celldesigner:lineDirection index="1" value="unknown"/>

</celldesigner:listOfLineDirection>

</celldesigner:connectScheme>

<celldesigner:editPoints>0.03582871419783987,-0.06939639962122257</celldesigner:editPoints>

<celldesigner:line width="1.0" color="ffff0000" type="Straight"/>

</celldesigner:reactantLink>

</celldesigner:listOfReactantLinks>

<celldesigner:connectScheme connectPolicy="direct" rectangleIndex="0">

<celldesigner:listOfLineDirection>

<celldesigner:lineDirection index="0" value="unknown"/>
</celldesigner:listOfLineDirection>
</celldesigner:connectScheme>
<celldesigner:line width="1.0" color="ffff0000"/>
</celldesigner:extension>
</annotation>
<listOfReactants>
<speciesReference metaid="CDMT00280" species="s1103">
<annotation>
<celldesigner:extension>
<celldesigner:alias>sa483</celldesigner:alias>
</celldesigner:extension>
</annotation>
</speciesReference>
<speciesReference metaid="CDMT00281" species="s1091">
<annotation>
<celldesigner:extension>
<celldesigner:alias>sa470</celldesigner:alias>
</celldesigner:extension>
</annotation>
</speciesReference>
</listOfReactants>
<listOfProducts>
<speciesReference metaid="CDMT00282" species="s730">
<annotation>
<celldesigner:extension>
<celldesigner:alias>sa482</celldesigner:alias>

</celldesigner:extension>
</annotation>
</speciesReference>
</listOfProducts>
</reaction>
<reaction metaid="re205" id="re205" name="DESAT204" reversible="false">
<annotation>
<celldesigner:extension>
<celldesigner:name>DESAT204</celldesigner:name>
<celldesigner:reactionType>STATE_TRANSITION</celldesigner:reactionType>
<celldesigner:baseReactants>
<celldesigner:baseReactant species="s96" alias="sa485"/>
</celldesigner:baseReactants>
<celldesigner:baseProducts>
<celldesigner:baseProduct species="s97" alias="sa491">
<celldesigner:linkAnchor position="N"/>
</celldesigner:baseProduct>
</celldesigner:baseProducts>
<celldesigner:listOfReactantLinks>
<celldesigner:reactantLink reactant="s1091" alias="sa470" targetLineIndex="-1,0">
<celldesigner:linkAnchor position="WNW"/>
<celldesigner:connectScheme connectPolicy="direct">
<celldesigner:listOfLineDirection>
<celldesigner:lineDirection index="0" value="unknown"/>
<celldesigner:lineDirection index="1" value="unknown"/>
</celldesigner:listOfLineDirection>
</celldesigner:connectScheme>

<celldesigner:editPoints>0.5176872553832789,0.1881316351453095</celldesigner:editPoints>

<celldesigner:line width="1.0" color="ffff0000" type="Straight"/>

</celldesigner:reactantLink>

</celldesigner:listOfReactantLinks>

<celldesigner:connectScheme connectPolicy="direct" rectangleIndex="0">

<celldesigner:listOfLineDirection>

<celldesigner:lineDirection index="0" value="unknown"/>

</celldesigner:listOfLineDirection>

</celldesigner:connectScheme>

<celldesigner:line width="1.0" color="ffff0000"/>

</celldesigner:extension>

</annotation>

<listOfReactants>

<speciesReference metaid="CDMT00283" species="s96">

<annotation>

<celldesigner:extension>

<celldesigner:alias>sa485</celldesigner:alias>

</celldesigner:extension>

</annotation>

</speciesReference>

<speciesReference metaid="CDMT00284" species="s1091">

<annotation>

<celldesigner:extension>

<celldesigner:alias>sa470</celldesigner:alias>

</celldesigner:extension>

</annotation>

</speciesReference>

</listOfReactants>

<listOfProducts>

<speciesReference metaid="CDMT00285" species="s97">

<annotation>

<celldesigner:extension>

<celldesigner:alias>sa491</celldesigner:alias>

</celldesigner:extension>

</annotation>

</speciesReference>

</listOfProducts>

</reaction>

<reaction metaid="re206" id="re206" name="DESAT205" reversible="false">

<annotation>

<celldesigner:extension>

<celldesigner:name>DESAT205</celldesigner:name>

<celldesigner:reactionType>STATE_TRANSITION</celldesigner:reactionType>

<celldesigner:baseReactants>

<celldesigner:baseReactant species="s97" alias="sa491"/>

</celldesigner:baseReactants>

<celldesigner:baseProducts>

<celldesigner:baseProduct species="s737" alias="sa490">

<celldesigner:linkAnchor position="N"/>

</celldesigner:baseProduct>

</celldesigner:baseProducts>

<celldesigner:connectScheme connectPolicy="direct" rectangleIndex="0">

<celldesigner:listOfLineDirection>

<celldesigner:lineDirection index="0" value="unknown"/>

</celldesigner:listOfLineDirection>

</celldesigner:connectScheme>

<celldesigner:line width="1.0" color="ffff0000"/>

</celldesigner:extension>

</annotation>

<listOfReactants>

<speciesReference metaid="CDMT00286" species="s97">

<annotation>

<celldesigner:extension>

<celldesigner:alias>sa491</celldesigner:alias>

</celldesigner:extension>

</annotation>

</speciesReference>

</listOfReactants>

<listOfProducts>

<speciesReference metaid="CDMT00287" species="s737">

<annotation>

<celldesigner:extension>

<celldesigner:alias>sa490</celldesigner:alias>

</celldesigner:extension>

</annotation>

</speciesReference>

</listOfProducts>

</reaction>

<reaction metaid="re207" id="re207" name="DESAT204" reversible="false">

<annotation>

<celldesigner:extension>

<celldesigner:name>DESAT204</celldesigner:name>

<celldesigner:reactionType>STATE_TRANSITION</celldesigner:reactionType>

<celldesigner:baseReactants>

<celldesigner:baseReactant species="s730" alias="sa482">

<celldesigner:linkAnchor position="S"/>

</celldesigner:baseReactant>

</celldesigner:baseReactants>

<celldesigner:baseProducts>

<celldesigner:baseProduct species="s1073" alias="sa484">

<celldesigner:linkAnchor position="N"/>

</celldesigner:baseProduct>

</celldesigner:baseProducts>

<celldesigner:connectScheme connectPolicy="direct" rectangleIndex="0">

<celldesigner:listOfLineDirection>

<celldesigner:lineDirection index="0" value="unknown"/>

</celldesigner:listOfLineDirection>

</celldesigner:connectScheme>

<celldesigner:line width="1.0" color="ffff0000"/>

</celldesigner:extension>

</annotation>

<listOfReactants>

<speciesReference metaid="CDMT00288" species="s730">

<annotation>

<celldesigner:extension>

<celldesigner:alias>sa482</celldesigner:alias>

</celldesigner:extension>

</annotation>

</speciesReference>

</listOfReactants>

<listOfProducts>

<speciesReference metaid="CDMT00289" species="s1073">

<annotation>

<celldesigner:extension>

<celldesigner:alias>sa484</celldesigner:alias>

</celldesigner:extension>

</annotation>

</speciesReference>

</listOfProducts>

</reaction>

<reaction metaid="re208" id="re208" name="DESAT224" reversible="false">

<annotation>

<celldesigner:extension>

<celldesigner:name>DESAT224</celldesigner:name>

<celldesigner:reactionType>STATE_TRANSITION</celldesigner:reactionType>

<celldesigner:baseReactants>

<celldesigner:baseReactant species="s1073" alias="sa484"/>

</celldesigner:baseReactants>

<celldesigner:baseProducts>

<celldesigner:baseProduct species="s734" alias="sa488"/>

</celldesigner:baseProducts>

<celldesigner:listOfReactantLinks>

<celldesigner:reactantLink reactant="s1091" alias="sa470" targetLineIndex="-1,0">

<celldesigner:linkAnchor position="ENE"/>

<celldesigner:connectScheme connectPolicy="direct">

<celldesigner:listOfLineDirection>

<celldesigner:lineDirection index="0" value="unknown"/>

<celldesigner:lineDirection index="1" value="unknown"/>

<celldesigner:lineDirection index="2" value="unknown"/>

</celldesigner:listOfLineDirection>

</celldesigner:connectScheme>

<celldesigner:editPoints>0.02379071351745654,-0.07263815115160654 0.9693093711434833,-0.06748354550969382</celldesigner:editPoints>

<celldesigner:line width="1.0" color="ffff0000" type="Straight"/>

</celldesigner:reactantLink>

</celldesigner:listOfReactantLinks>

<celldesigner:connectScheme connectPolicy="direct" rectangleIndex="0">

<celldesigner:listOfLineDirection>

<celldesigner:lineDirection index="0" value="unknown"/>

</celldesigner:listOfLineDirection>

</celldesigner:connectScheme>

<celldesigner:line width="1.0" color="ffff0000"/>

</celldesigner:extension>

</annotation>

<listOfReactants>

<speciesReference metaid="CDMT00290" species="s1073">

<annotation>

<celldesigner:extension>

<celldesigner:alias>sa484</celldesigner:alias>

</celldesigner:extension>

</annotation>

</speciesReference>

<speciesReference metaid="CDMT00291" species="s1091">

<annotation>
<celldesigner:extension>
<celldesigner:alias>sa470</celldesigner:alias>
</celldesigner:extension>
</annotation>
</speciesReference>
</listOfReactants>
<listOfProducts>
<speciesReference metaid="CDMT00292" species="s734">
<annotation>
<celldesigner:extension>
<celldesigner:alias>sa488</celldesigner:alias>
</celldesigner:extension>
</annotation>
</speciesReference>
</listOfProducts>
</reaction>
<reaction metaid="re209" id="re209" name="DESAT225" reversible="false">
<annotation>
<celldesigner:extension>
<celldesigner:name>DESAT225</celldesigner:name>
<celldesigner:reactionType>STATE_TRANSITION</celldesigner:reactionType>
<celldesigner:baseReactants>
<celldesigner:baseReactant species="s734" alias="sa488">
<celldesigner:linkAnchor position="S"/>
</celldesigner:baseReactant>
</celldesigner:baseReactants>

<celldesigner:baseProducts>
<celldesigner:baseProduct species="s736" alias="sa489"/>
</celldesigner:baseProducts>
<celldesigner:connectScheme connectPolicy="direct" rectangleIndex="0">
<celldesigner:listOfLineDirection>
<celldesigner:lineDirection index="0" value="unknown"/>
</celldesigner:listOfLineDirection>
</celldesigner:connectScheme>
<celldesigner:line width="1.0" color="ffff0000"/>
</celldesigner:extension>
</annotation>
<listOfReactants>
<speciesReference metaid="CDMT00293" species="s734">
<annotation>
<celldesigner:extension>
<celldesigner:alias>sa488</celldesigner:alias>
</celldesigner:extension>
</annotation>
</speciesReference>
</listOfReactants>
<listOfProducts>
<speciesReference metaid="CDMT00294" species="s736">
<annotation>
<celldesigner:extension>
<celldesigner:alias>sa489</celldesigner:alias>
</celldesigner:extension>
</annotation>

</speciesReference>
</listOfProducts>
</reaction>
<reaction metaid="re210" id="re210" name="DESAT225" reversible="false">
<annotation>
<celldesigner:extension>
<celldesigner:name>DESAT225</celldesigner:name>
<celldesigner:reactionType>STATE_TRANSITION</celldesigner:reactionType>
<celldesigner:baseReactants>
<celldesigner:baseReactant species="s737" alias="sa490">
<celldesigner:linkAnchor position="S"/>
</celldesigner:baseReactant>
</celldesigner:baseReactants>
<celldesigner:baseProducts>
<celldesigner:baseProduct species="s732" alias="sa486"/>
</celldesigner:baseProducts>
<celldesigner:listOfReactantLinks>
<celldesigner:reactantLink reactant="s1091" alias="sa470" targetLineIndex="-1,0">
<celldesigner:linkAnchor position="WNW"/>
<celldesigner:connectScheme connectPolicy="direct">
<celldesigner:listOfLineDirection>
<celldesigner:lineDirection index="0" value="unknown"/>
<celldesigner:lineDirection index="1" value="unknown"/>
</celldesigner:listOfLineDirection>
</celldesigner:connectScheme>
<celldesigner:editPoints>0.5011027114218014,0.1948493036884129</celldesigner:editPoints>
<celldesigner:line width="1.0" color="ffff0000" type="Straight"/>

</celldesigner:reactantLink>
</celldesigner:listOfReactantLinks>
<celldesigner:connectScheme connectPolicy="direct" rectangleIndex="0">
<celldesigner:listOfLineDirection>
<celldesigner:lineDirection index="0" value="unknown"/>
</celldesigner:listOfLineDirection>
</celldesigner:connectScheme>
<celldesigner:line width="1.0" color="ffff0000"/>
</celldesigner:extension>
</annotation>
<listOfReactants>
<speciesReference metaid="CDMT00295" species="s737">
<annotation>
<celldesigner:extension>
<celldesigner:alias>sa490</celldesigner:alias>
</celldesigner:extension>
</annotation>
</speciesReference>
<speciesReference metaid="CDMT00296" species="s1091">
<annotation>
<celldesigner:extension>
<celldesigner:alias>sa470</celldesigner:alias>
</celldesigner:extension>
</annotation>
</speciesReference>
</listOfReactants>
<listOfProducts>

<speciesReference metaid="CDMT00297" species="s732">

<annotation>

<celldesigner:extension>

<celldesigner:alias>sa486</celldesigner:alias>

</celldesigner:extension>

</annotation>

</speciesReference>

</listOfProducts>

</reaction>

<reaction metaid="re211" id="re211" name="DESAT226" reversible="false">

<annotation>

<celldesigner:extension>

<celldesigner:name>DESAT226</celldesigner:name>

<celldesigner:reactionType>STATE_TRANSITION</celldesigner:reactionType>

<celldesigner:baseReactants>

<celldesigner:baseReactant species="s732" alias="sa486"/>

</celldesigner:baseReactants>

<celldesigner:baseProducts>

<celldesigner:baseProduct species="s733" alias="sa487"/>

</celldesigner:baseProducts>

<celldesigner:connectScheme connectPolicy="direct" rectangleIndex="0">

<celldesigner:listOfLineDirection>

<celldesigner:lineDirection index="0" value="unknown"/>

</celldesigner:listOfLineDirection>

</celldesigner:connectScheme>

<celldesigner:line width="1.0" color="ffff0000"/>

</celldesigner:extension>

</annotation>

<listOfReactants>

<speciesReference metaid="CDMT00298" species="s732">

<annotation>

<celldesigner:extension>

<celldesigner:alias>sa486</celldesigner:alias>

</celldesigner:extension>

</annotation>

</speciesReference>

</listOfReactants>

<listOfProducts>

<speciesReference metaid="CDMT00299" species="s733">

<annotation>

<celldesigner:extension>

<celldesigner:alias>sa487</celldesigner:alias>

</celldesigner:extension>

</annotation>

</speciesReference>

</listOfProducts>

</reaction>

<reaction metaid="re244" id="re212" name="NsDPKB" reversible="false">

<annotation>

<celldesigner:extension>

<celldesigner:name>NsDPKB</celldesigner:name>

<celldesigner:reactionType>STATE_TRANSITION</celldesigner:reactionType>

<celldesigner:baseReactants>

<celldesigner:baseReactant species="s440" alias="sa492"/>

</celldesigner:baseReactants>

<celldesigner:baseProducts>

<celldesigner:baseProduct species="s1092" alias="sa495"/>

</celldesigner:baseProducts>

<celldesigner:connectScheme connectPolicy="direct" rectangleIndex="0">

<celldesigner:listOfLineDirection>

<celldesigner:lineDirection index="0" value="unknown"/>

</celldesigner:listOfLineDirection>

</celldesigner:connectScheme>

<celldesigner:line width="1.0" color="ffff3333"/>

</celldesigner:extension>

</annotation>

<listOfReactants>

<speciesReference metaid="CDMT00300" species="s440">

<annotation>

<celldesigner:extension>

<celldesigner:alias>sa492</celldesigner:alias>

</celldesigner:extension>

</annotation>

</speciesReference>

</listOfReactants>

<listOfProducts>

<speciesReference metaid="CDMT00301" species="s1092">

<annotation>

<celldesigner:extension>

<celldesigner:alias>sa495</celldesigner:alias>

</celldesigner:extension>

</annotation>

</speciesReference>

</listOfProducts>

</reaction>

<reaction metaid="re245" id="re213" name="RR" reversible="false">

<annotation>

<celldesigner:extension>

<celldesigner:name>RR</celldesigner:name>

<celldesigner:reactionType>STATE_TRANSITION</celldesigner:reactionType>

<celldesigner:baseReactants>

<celldesigner:baseReactant species="s1092" alias="sa495"/>

</celldesigner:baseReactants>

<celldesigner:baseProducts>

<celldesigner:baseProduct species="s441" alias="sa499"/>

</celldesigner:baseProducts>

<celldesigner:connectScheme connectPolicy="direct" rectangleIndex="0">

<celldesigner:listOfLineDirection>

<celldesigner:lineDirection index="0" value="unknown"/>

</celldesigner:listOfLineDirection>

</celldesigner:connectScheme>

<celldesigner:line width="1.0" color="ffff3333"/>

</celldesigner:extension>

</annotation>

<listOfReactants>

<speciesReference metaid="CDMT00302" species="s1092">

<annotation>

<celldesigner:extension>

<celldesigner:alias>sa495</celldesigner:alias>

</celldesigner:extension>

</annotation>

</speciesReference>

</listOfReactants>

<listOfProducts>

<speciesReference metaid="CDMT00303" species="s441">

<annotation>

<celldesigner:extension>

<celldesigner:alias>sa499</celldesigner:alias>

</celldesigner:extension>

</annotation>

</speciesReference>

</listOfProducts>

</reaction>

<reaction metaid="re246" id="re214" name="AdK" reversible="false">

<annotation>

<celldesigner:extension>

<celldesigner:name>AdK</celldesigner:name>

<celldesigner:reactionType>STATE_TRANSITION</celldesigner:reactionType>

<celldesigner:baseReactants>

<celldesigner:baseReactant species="s1092" alias="sa495">

<celldesigner:linkAnchor position="ESE"/>

</celldesigner:baseReactant>

</celldesigner:baseReactants>

<celldesigner:baseProducts>

<celldesigner:baseProduct species="s1000" alias="sa494">

<celldesigner:linkAnchor position="WSW"/>

</celldesigner:baseProduct>

</celldesigner:baseProducts>

<celldesigner:connectScheme connectPolicy="direct" rectangleIndex="0">

<celldesigner:listOfLineDirection>

<celldesigner:lineDirection index="0" value="unknown"/>

</celldesigner:listOfLineDirection>

</celldesigner:connectScheme>

<celldesigner:line width="1.0" color="ffff3333"/>

</celldesigner:extension>

</annotation>

<listOfReactants>

<speciesReference metaid="CDMT00304" species="s1092">

<annotation>

<celldesigner:extension>

<celldesigner:alias>sa495</celldesigner:alias>

</celldesigner:extension>

</annotation>

</speciesReference>

</listOfReactants>

<listOfProducts>

<speciesReference metaid="CDMT00305" species="s1000">

<annotation>

<celldesigner:extension>

<celldesigner:alias>sa494</celldesigner:alias>

</celldesigner:extension>

</annotation>

</speciesReference>

</listOfProducts>

</reaction>

<reaction metaid="re247" id="re215" name="AdK" reversible="false">

<annotation>

<celldesigner:extension>

<celldesigner:name>AdK</celldesigner:name>

<celldesigner:reactionType>STATE_TRANSITION</celldesigner:reactionType>

<celldesigner:baseReactants>

<celldesigner:baseReactant species="s441" alias="sa499"/>

</celldesigner:baseReactants>

<celldesigner:baseProducts>

<celldesigner:baseProduct species="s1038" alias="sa498"/>

</celldesigner:baseProducts>

<celldesigner:connectScheme connectPolicy="direct" rectangleIndex="0">

<celldesigner:listOfLineDirection>

<celldesigner:lineDirection index="0" value="unknown"/>

</celldesigner:listOfLineDirection>

</celldesigner:connectScheme>

<celldesigner:line width="1.0" color="ffff3333"/>

</celldesigner:extension>

</annotation>

<listOfReactants>

<speciesReference metaid="CDMT00306" species="s441">

<annotation>

<celldesigner:extension>

<celldesigner:alias>sa499</celldesigner:alias>

</celldesigner:extension>

</annotation>

</speciesReference>

</listOfReactants>

<listOfProducts>

<speciesReference metaid="CDMT00307" species="s1038">

<annotation>

<celldesigner:extension>

<celldesigner:alias>sa498</celldesigner:alias>

</celldesigner:extension>

</annotation>

</speciesReference>

</listOfProducts>

</reaction>

<reaction metaid="re248" id="re216" name="AMPDeaminase" reversible="false">

<annotation>

<celldesigner:extension>

<celldesigner:name>AMPDeaminase</celldesigner:name>

<celldesigner:reactionType>STATE_TRANSITION</celldesigner:reactionType>

<celldesigner:baseReactants>

<celldesigner:baseReactant species="s1000" alias="sa494"/>

</celldesigner:baseReactants>

<celldesigner:baseProducts>

<celldesigner:baseProduct species="s1100" alias="sa501">

<celldesigner:linkAnchor position="NNW"/>

</celldesigner:baseProduct>

</celldesigner:baseProducts>

<celldesigner:connectScheme connectPolicy="direct" rectangleIndex="0">

<celldesigner:listOfLineDirection>

<celldesigner:lineDirection index="0" value="unknown"/>

</celldesigner:listOfLineDirection>

</celldesigner:connectScheme>

<celldesigner:line width="1.0" color="ffff3333"/>

</celldesigner:extension>

</annotation>

<listOfReactants>

<speciesReference metaid="CDMT00308" species="s1000">

<annotation>

<celldesigner:extension>

<celldesigner:alias>sa494</celldesigner:alias>

</celldesigner:extension>

</annotation>

</speciesReference>

</listOfReactants>

<listOfProducts>

<speciesReference metaid="CDMT00309" species="s1100">

<annotation>

<celldesigner:extension>

<celldesigner:alias>sa501</celldesigner:alias>

</celldesigner:extension>

</annotation>

</speciesReference>

</listOfProducts>

</reaction>

<reaction metaid="re249" id="re217" name="NsDPKB" reversible="false">

<annotation>

<celldesigner:extension>

<celldesigner:name>NsDPKB</celldesigner:name>

<celldesigner:reactionType>STATE_TRANSITION</celldesigner:reactionType>

<celldesigner:baseReactants>

<celldesigner:baseReactant species="s1089" alias="sa496"/>

</celldesigner:baseReactants>

<celldesigner:baseProducts>

<celldesigner:baseProduct species="s1090" alias="sa497"/>

</celldesigner:baseProducts>

<celldesigner:connectScheme connectPolicy="direct" rectangleIndex="0">

<celldesigner:listOfLineDirection>

<celldesigner:lineDirection index="0" value="unknown"/>

</celldesigner:listOfLineDirection>

</celldesigner:connectScheme>

<celldesigner:line width="1.0" color="ffff3333"/>

</celldesigner:extension>

</annotation>

<listOfReactants>

<speciesReference metaid="CDMT00310" species="s1089">

<annotation>

<celldesigner:extension>

<celldesigner:alias>sa496</celldesigner:alias>

</celldesigner:extension>

</annotation>

</speciesReference>

</listOfReactants>

<listOfProducts>

<speciesReference metaid="CDMT00311" species="s1090">

<annotation>

<celldesigner:extension>

<celldesigner:alias>sa497</celldesigner:alias>

</celldesigner:extension>

</annotation>

</speciesReference>

</listOfProducts>

</reaction>

<reaction metaid="re250" id="re218" name="RR" reversible="false">

<annotation>

<celldesigner:extension>

<celldesigner:name>RR</celldesigner:name>

<celldesigner:reactionType>STATE_TRANSITION</celldesigner:reactionType>

<celldesigner:baseReactants>

<celldesigner:baseReactant species="s1090" alias="sa497"/>

</celldesigner:baseReactants>

<celldesigner:baseProducts>

<celldesigner:baseProduct species="s1096" alias="sa502"/>

</celldesigner:baseProducts>

<celldesigner:connectScheme connectPolicy="direct" rectangleIndex="0">

<celldesigner:listOfLineDirection>

<celldesigner:lineDirection index="0" value="unknown"/>

</celldesigner:listOfLineDirection>

</celldesigner:connectScheme>

<celldesigner:line width="1.0" color="ffff3333"/>

</celldesigner:extension>

</annotation>

<listOfReactants>

<speciesReference metaid="CDMT00312" species="s1090">

<annotation>

<celldesigner:extension>

<celldesigner:alias>sa497</celldesigner:alias>

</celldesigner:extension>

</annotation>

</speciesReference>

</listOfReactants>

<listOfProducts>

<speciesReference metaid="CDMT00313" species="s1096">

<annotation>

<celldesigner:extension>

<celldesigner:alias>sa502</celldesigner:alias>

</celldesigner:extension>

</annotation>

</speciesReference>

</listOfProducts>

</reaction>

<reaction metaid="re251" id="re219" name="GuaK" reversible="false">

<annotation>

<celldesigner:extension>

<celldesigner:name>GuaK</celldesigner:name>

<celldesigner:reactionType>STATE_TRANSITION</celldesigner:reactionType>

<celldesigner:baseReactants>

<celldesigner:baseReactant species="s1096" alias="sa502"/>

</celldesigner:baseReactants>

<celldesigner:baseProducts>

<celldesigner:baseProduct species="s1037" alias="sa506"/>

</celldesigner:baseProducts>

<celldesigner:connectScheme connectPolicy="direct" rectangleIndex="0">

<celldesigner:listOfLineDirection>

<celldesigner:lineDirection index="0" value="unknown"/>

</celldesigner:listOfLineDirection>

</celldesigner:connectScheme>

<celldesigner:line width="1.0" color="ffff3333"/>

</celldesigner:extension>

</annotation>

<listOfReactants>

<speciesReference metaid="CDMT00314" species="s1096">

<annotation>

<celldesigner:extension>

<celldesigner:alias>sa502</celldesigner:alias>

</celldesigner:extension>

</annotation>

</speciesReference>

</listOfReactants>

<listOfProducts>

<speciesReference metaid="CDMT00315" species="s1037">

<annotation>

<celldesigner:extension>

<celldesigner:alias>sa506</celldesigner:alias>
</celldesigner:extension>
</annotation>
</speciesReference>
</listOfProducts>
</reaction>
<reaction metaid="re252" id="re220" name="GuaK" reversible="false">
<annotation>
<celldesigner:extension>
<celldesigner:name>GuaK</celldesigner:name>
<celldesigner:reactionType>STATE_TRANSITION</celldesigner:reactionType>
<celldesigner:baseReactants>
<celldesigner:baseReactant species="s1090" alias="sa497">
<celldesigner:linkAnchor position="WNW"/>
</celldesigner:baseReactant>
</celldesigner:baseReactants>
<celldesigner:baseProducts>
<celldesigner:baseProduct species="s1027" alias="sa493">
<celldesigner:linkAnchor position="E"/>
</celldesigner:baseProduct>
</celldesigner:baseProducts>
<celldesigner:connectScheme connectPolicy="direct" rectangleIndex="0">
<celldesigner:listOfLineDirection>
<celldesigner:lineDirection index="0" value="unknown"/>
</celldesigner:listOfLineDirection>
</celldesigner:connectScheme>
<celldesigner:line width="1.0" color="ffff3333"/>

</celldesigner:extension>

</annotation>

<listOfReactants>

<speciesReference metaid="CDMT00316" species="s1090">

<annotation>

<celldesigner:extension>

<celldesigner:alias>sa497</celldesigner:alias>

</celldesigner:extension>

</annotation>

</speciesReference>

</listOfReactants>

<listOfProducts>

<speciesReference metaid="CDMT00317" species="s1027">

<annotation>

<celldesigner:extension>

<celldesigner:alias>sa493</celldesigner:alias>

</celldesigner:extension>

</annotation>

</speciesReference>

</listOfProducts>

</reaction>

<reaction metaid="re253" id="re221" name="IMPDH" reversible="false">

<annotation>

<celldesigner:extension>

<celldesigner:name>IMPDH</celldesigner:name>

<celldesigner:reactionType>STATE_TRANSITION</celldesigner:reactionType>

<celldesigner:baseReactants>

```
<celldesigner:baseReactant species="s1100" alias="sa501"/>
</celldesigner:baseReactants>
<celldesigner:baseProducts>
<celldesigner:baseProduct species="s1095" alias="sa503">
<celldesigner:linkAnchor position="WNW"/>
</celldesigner:baseProduct>
</celldesigner:baseProducts>
<celldesigner:connectScheme connectPolicy="direct" rectangleIndex="0">
<celldesigner:listOfLineDirection>
<celldesigner:lineDirection index="0" value="unknown"/>
</celldesigner:listOfLineDirection>
</celldesigner:connectScheme>
<celldesigner:line width="1.0" color="ffff3333"/>
</celldesigner:extension>
</annotation>
<listOfReactants>
<speciesReference metaid="CDMT00318" species="s1100">
<annotation>
<celldesigner:extension>
<celldesigner:alias>sa501</celldesigner:alias>
</celldesigner:extension>
</annotation>
</speciesReference>
</listOfReactants>
<listOfProducts>
<speciesReference metaid="CDMT00319" species="s1095">
<annotation>
```

<celldesigner:extension>
<celldesigner:alias>sa503</celldesigner:alias>
</celldesigner:extension>
</annotation>
</speciesReference>
</listOfProducts>
</reaction>
<reaction metaid="re254" id="re222" name="GMPS" reversible="false">
<annotation>
<celldesigner:extension>
<celldesigner:name>GMPS</celldesigner:name>
<celldesigner:reactionType>STATE_TRANSITION</celldesigner:reactionType>
<celldesigner:baseReactants>
<celldesigner:baseReactant species="s1095" alias="sa503">
<celldesigner:linkAnchor position="NNE"/>
</celldesigner:baseReactant>
</celldesigner:baseReactants>
<celldesigner:baseProducts>
<celldesigner:baseProduct species="s1027" alias="sa493"/>
</celldesigner:baseProducts>
<celldesigner:listOfProductLinks>
<celldesigner:productLink product="s1000" alias="sa494" targetLineIndex="-1,1">
<celldesigner:linkAnchor position="ESE"/>
<celldesigner:connectScheme connectPolicy="direct">
<celldesigner:listOfLineDirection>
<celldesigner:lineDirection index="0" value="unknown"/>
</celldesigner:listOfLineDirection>

</celldesigner:connectScheme>
<celldesigner:line width="1.0" color="ffff3333" type="Straight"/>
</celldesigner:productLink>
</celldesigner:listOfProductLinks>
<celldesigner:connectScheme connectPolicy="direct" rectangleIndex="0">
<celldesigner:listOfLineDirection>
<celldesigner:lineDirection index="0" value="unknown"/>
</celldesigner:listOfLineDirection>
</celldesigner:connectScheme>
<celldesigner:line width="1.0" color="ffff3333"/>
</celldesigner:extension>
</annotation>
<listOfReactants>
<speciesReference metaid="CDMT00320" species="s1095">
<annotation>
<celldesigner:extension>
<celldesigner:alias>sa503</celldesigner:alias>
</celldesigner:extension>
</annotation>
</speciesReference>
</listOfReactants>
<listOfProducts>
<speciesReference metaid="CDMT00321" species="s1027">
<annotation>
<celldesigner:extension>
<celldesigner:alias>sa493</celldesigner:alias>
</celldesigner:extension>

</annotation>

</speciesReference>

<speciesReference metaid="CDMT00322" species="s1000">

<annotation>

<celldesigner:extension>

<celldesigner:alias>sa494</celldesigner:alias>

</celldesigner:extension>

</annotation>

</speciesReference>

</listOfProducts>

</reaction>

<reaction metaid="re255" id="re223" name="CarbPS" reversible="false">

<annotation>

<celldesigner:extension>

<celldesigner:name>CarbPS</celldesigner:name>

<celldesigner:reactionType>STATE_TRANSITION</celldesigner:reactionType>

<celldesigner:baseReactants>

<celldesigner:baseReactant species="s979" alias="sa541"/>

</celldesigner:baseReactants>

<celldesigner:baseProducts>

<celldesigner:baseProduct species="s269" alias="sa508"/>

</celldesigner:baseProducts>

<celldesigner:connectScheme connectPolicy="direct" rectangleIndex="0">

<celldesigner:listOfLineDirection>

<celldesigner:lineDirection index="0" value="unknown"/>

</celldesigner:listOfLineDirection>

</celldesigner:connectScheme>

<celldesigner:line width="1.0" color="ff000000"/>

</celldesigner:extension>

</annotation>

<listOfReactants>

<speciesReference metaid="CDMT00323" species="s979">

<annotation>

<celldesigner:extension>

<celldesigner:alias>sa541</celldesigner:alias>

</celldesigner:extension>

</annotation>

</speciesReference>

</listOfReactants>

<listOfProducts>

<speciesReference metaid="CDMT00324" species="s269">

<annotation>

<celldesigner:extension>

<celldesigner:alias>sa508</celldesigner:alias>

</celldesigner:extension>

</annotation>

</speciesReference>

</listOfProducts>

</reaction>

<reaction metaid="re256" id="re224" name="AspCarbTr" reversible="false">

<annotation>

<celldesigner:extension>

<celldesigner:name>AspCarbTr</celldesigner:name>

<celldesigner:reactionType>STATE_TRANSITION</celldesigner:reactionType>

<celldesigner:baseReactants>

<celldesigner:baseReactant species="s269" alias="sa508">

<celldesigner:linkAnchor position="S"/>

</celldesigner:baseReactant>

</celldesigner:baseReactants>

<celldesigner:baseProducts>

<celldesigner:baseProduct species="s270" alias="sa513"/>

</celldesigner:baseProducts>

<celldesigner:listOfReactantLinks>

<celldesigner:reactantLink reactant="s1004" alias="sa509" targetLineIndex="-1,0">

<celldesigner:linkAnchor position="ENE"/>

<celldesigner:connectScheme connectPolicy="direct">

<celldesigner:listOfLineDirection>

<celldesigner:lineDirection index="0" value="unknown"/>

</celldesigner:listOfLineDirection>

</celldesigner:connectScheme>

<celldesigner:line width="1.0" color="ff000000" type="Straight"/>

</celldesigner:reactantLink>

</celldesigner:listOfReactantLinks>

<celldesigner:connectScheme connectPolicy="direct" rectangleIndex="0">

<celldesigner:listOfLineDirection>

<celldesigner:lineDirection index="0" value="unknown"/>

</celldesigner:listOfLineDirection>

</celldesigner:connectScheme>

<celldesigner:line width="1.0" color="ff000000"/>

</celldesigner:extension>

</annotation>

<listOfReactants>

<speciesReference metaid="CDMT00325" species="s269">

<annotation>

<celldesigner:extension>

<celldesigner:alias>sa508</celldesigner:alias>

</celldesigner:extension>

</annotation>

</speciesReference>

<speciesReference metaid="CDMT00326" species="s1004">

<annotation>

<celldesigner:extension>

<celldesigner:alias>sa509</celldesigner:alias>

</celldesigner:extension>

</annotation>

</speciesReference>

</listOfReactants>

<listOfProducts>

<speciesReference metaid="CDMT00327" species="s270">

<annotation>

<celldesigner:extension>

<celldesigner:alias>sa513</celldesigner:alias>

</celldesigner:extension>

</annotation>

</speciesReference>

</listOfProducts>

</reaction>

<reaction metaid="re257" id="re225" name="DHORT" reversible="false">

<annotation>
<celldesigner:extension>
<celldesigner:name>DHORT</celldesigner:name>
<celldesigner:reactionType>STATE_TRANSITION</celldesigner:reactionType>
<celldesigner:baseReactants>
<celldesigner:baseReactant species="s270" alias="sa513">
<celldesigner:linkAnchor position="S"/>
</celldesigner:baseReactant>
</celldesigner:baseReactants>
<celldesigner:baseProducts>
<celldesigner:baseProduct species="s271" alias="sa517"/>
</celldesigner:baseProducts>
<celldesigner:connectScheme connectPolicy="direct" rectangleIndex="0">
<celldesigner:listOfLineDirection>
<celldesigner:lineDirection index="0" value="unknown"/>
</celldesigner:listOfLineDirection>
</celldesigner:connectScheme>
<celldesigner:line width="1.0" color="ff000000"/>
</celldesigner:extension>
</annotation>
<listOfReactants>
<speciesReference metaid="CDMT00328" species="s270">
<annotation>
<celldesigner:extension>
<celldesigner:alias>sa513</celldesigner:alias>
</celldesigner:extension>
</annotation>

</speciesReference>
</listOfReactants>
<listOfProducts>
<speciesReference metaid="CDMT00329" species="s271">
<annotation>
<celldesigner:extension>
<celldesigner:alias>sa517</celldesigner:alias>
</celldesigner:extension>
</annotation>
</speciesReference>
</listOfProducts>
</reaction>
<reaction metaid="CDMT00330" id="re226" name="DHOTDH" reversible="false">
<annotation>
<celldesigner:extension>
<celldesigner:name>DHOTDH</celldesigner:name>
<celldesigner:reactionType>STATE_TRANSITION</celldesigner:reactionType>
<celldesigner:baseReactants>
<celldesigner:baseReactant species="s271" alias="sa517">
<celldesigner:linkAnchor position="S"/>
</celldesigner:baseReactant>
</celldesigner:baseReactants>
<celldesigner:baseProducts>
<celldesigner:baseProduct species="s272" alias="sa521"/>
</celldesigner:baseProducts>
<celldesigner:listOfReactantLinks>
<celldesigner:reactantLink reactant="s24" alias="sa234" targetLineIndex="-1,0">

<celldesigner:linkAnchor position="SE"/>

<celldesigner:connectScheme connectPolicy="direct">

<celldesigner:listOfLineDirection>

<celldesigner:lineDirection index="0" value="unknown"/>

<celldesigner:lineDirection index="1" value="unknown"/>

<celldesigner:lineDirection index="2" value="unknown"/>

</celldesigner:listOfLineDirection>

</celldesigner:connectScheme>

<celldesigner:editPoints>0.4282187005674103,0.2479336121302913 0.8905594866299182,0.1496566687375476</celldesigner:editPoints>

<celldesigner:line width="1.0" color="ff000000" type="Straight"/>

</celldesigner:reactantLink>

</celldesigner:listOfReactantLinks>

<celldesigner:listOfProductLinks>

<celldesigner:productLink product="s28" alias="sa522" targetLineIndex="-1,1">

<celldesigner:connectScheme connectPolicy="direct">

<celldesigner:listOfLineDirection>

<celldesigner:lineDirection index="0" value="unknown"/>

</celldesigner:listOfLineDirection>

</celldesigner:connectScheme>

<celldesigner:line width="1.0" color="ff000000" type="Straight"/>

</celldesigner:productLink>

</celldesigner:listOfProductLinks>

<celldesigner:connectScheme connectPolicy="direct" rectangleIndex="0">

<celldesigner:listOfLineDirection>

<celldesigner:lineDirection index="0" value="unknown"/>

</celldesigner:listOfLineDirection>

</celldesigner:connectScheme>

<celldesigner:line width="1.0" color="ff000000"/>

</celldesigner:extension>

</annotation>

<listOfReactants>

<speciesReference metaid="CDMT00331" species="s271">

<annotation>

<celldesigner:extension>

<celldesigner:alias>sa517</celldesigner:alias>

</celldesigner:extension>

</annotation>

</speciesReference>

<speciesReference metaid="CDMT00332" species="s24">

<annotation>

<celldesigner:extension>

<celldesigner:alias>sa234</celldesigner:alias>

</celldesigner:extension>

</annotation>

</speciesReference>

</listOfReactants>

<listOfProducts>

<speciesReference metaid="CDMT00333" species="s272">

<annotation>

<celldesigner:extension>

<celldesigner:alias>sa521</celldesigner:alias>

</celldesigner:extension>

</annotation>

</speciesReference>

<speciesReference metaid="CDMT00334" species="s28">

<annotation>

<celldesigner:extension>

<celldesigner:alias>sa522</celldesigner:alias>

</celldesigner:extension>

</annotation>

</speciesReference>

</listOfProducts>

</reaction>

<reaction metaid="re259" id="re227" name="ORTPRT" reversible="false">

<annotation>

<celldesigner:extension>

<celldesigner:name>ORTPRT</celldesigner:name>

<celldesigner:reactionType>STATE_TRANSITION</celldesigner:reactionType>

<celldesigner:baseReactants>

<celldesigner:baseReactant species="s272" alias="sa521"/>

</celldesigner:baseReactants>

<celldesigner:baseProducts>

<celldesigner:baseProduct species="s273" alias="sa523"/>

</celldesigner:baseProducts>

<celldesigner:connectScheme connectPolicy="direct" rectangleIndex="0">

<celldesigner:listOfLineDirection>

<celldesigner:lineDirection index="0" value="unknown"/>

</celldesigner:listOfLineDirection>

</celldesigner:connectScheme>

<celldesigner:line width="1.0" color="ff000000"/>

</celldesigner:extension>

</annotation>
<listOfReactants>
<speciesReference metaid="CDMT00335" species="s272">
<annotation>
<celldesigner:extension>
<celldesigner:alias>sa521</celldesigner:alias>
</celldesigner:extension>
</annotation>
</speciesReference>
</listOfReactants>
<listOfProducts>
<speciesReference metaid="CDMT00336" species="s273">
<annotation>
<celldesigner:extension>
<celldesigner:alias>sa523</celldesigner:alias>
</celldesigner:extension>
</annotation>
</speciesReference>
</listOfProducts>
</reaction>
<reaction metaid="re260" id="re228" name="UrdMPS" reversible="false">
<annotation>
<celldesigner:extension>
<celldesigner:name>UrdMPS</celldesigner:name>
<celldesigner:reactionType>STATE_TRANSITION</celldesigner:reactionType>
<celldesigner:baseReactants>
<celldesigner:baseReactant species="s273" alias="sa523"/>

</celldesigner:baseReactants>

<celldesigner:baseProducts>

<celldesigner:baseProduct species="s1114" alias="sa525"/>

</celldesigner:baseProducts>

<celldesigner:listOfProductLinks>

<celldesigner:productLink product="s433" alias="sa504" targetLineIndex="-1,1">

<celldesigner:linkAnchor position="SSW"/>

<celldesigner:connectScheme connectPolicy="direct">

<celldesigner:listOfLineDirection>

<celldesigner:lineDirection index="0" value="unknown"/>

<celldesigner:lineDirection index="1" value="unknown"/>

</celldesigner:listOfLineDirection>

</celldesigner:connectScheme>

<celldesigner:editPoints>0.1723455257826143,0.07480017585722876</celldesigner:editPoints>

<celldesigner:line width="1.0" color="ff000000" type="Straight"/>

</celldesigner:productLink>

</celldesigner:listOfProductLinks>

<celldesigner:connectScheme connectPolicy="direct" rectangleIndex="0">

<celldesigner:listOfLineDirection>

<celldesigner:lineDirection index="0" value="unknown"/>

</celldesigner:listOfLineDirection>

</celldesigner:connectScheme>

<celldesigner:line width="1.0" color="ff000000"/>

</celldesigner:extension>

</annotation>

<listOfReactants>

<speciesReference metaid="CDMT00337" species="s273">

<annotation>
<celldesigner:extension>
<celldesigner:alias>sa523</celldesigner:alias>
</celldesigner:extension>
</annotation>
</speciesReference>
</listOfReactants>
<listOfProducts>
<speciesReference metaid="CDMT00338" species="s1114">
<annotation>
<celldesigner:extension>
<celldesigner:alias>sa525</celldesigner:alias>
</celldesigner:extension>
</annotation>
</speciesReference>
<speciesReference metaid="CDMT00339" species="s433">
<annotation>
<celldesigner:extension>
<celldesigner:alias>sa504</celldesigner:alias>
</celldesigner:extension>
</annotation>
</speciesReference>
</listOfProducts>
</reaction>
<reaction metaid="re261" id="re229" name="CytDeaminase" reversible="false">
<annotation>
<celldesigner:extension>

<celldesigner:name>CytDeaminase</celldesigner:name>
<celldesigner:reactionType>STATE_TRANSITION</celldesigner:reactionType>
<celldesigner:baseReactants>
<celldesigner:baseReactant species="s277" alias="sa515">
<celldesigner:linkAnchor position="S"/>
</celldesigner:baseReactant>
</celldesigner:baseReactants>
<celldesigner:baseProducts>
<celldesigner:baseProduct species="s276" alias="sa520"/>
</celldesigner:baseProducts>
<celldesigner:connectScheme connectPolicy="direct" rectangleIndex="0">
<celldesigner:listOfLineDirection>
<celldesigner:lineDirection index="0" value="unknown"/>
</celldesigner:listOfLineDirection>
</celldesigner:connectScheme>
<celldesigner:line width="1.0" color="ffff3333"/>
</celldesigner:extension>
</annotation>
<listOfReactants>
<speciesReference metaid="CDMT00340" species="s277">
<annotation>
<celldesigner:extension>
<celldesigner:alias>sa515</celldesigner:alias>
</celldesigner:extension>
</annotation>
</speciesReference>
</listOfReactants>

<listOfProducts>

<speciesReference metaid="CDMT00341" species="s276">

<annotation>

<celldesigner:extension>

<celldesigner:alias>sa520</celldesigner:alias>

</celldesigner:extension>

</annotation>

</speciesReference>

</listOfProducts>

</reaction>

<reaction metaid="re262" id="re230" name="Uri/CytK" reversible="false">

<annotation>

<celldesigner:extension>

<celldesigner:name>Uri/CytK</celldesigner:name>

<celldesigner:reactionType>STATE_TRANSITION</celldesigner:reactionType>

<celldesigner:baseReactants>

<celldesigner:baseReactant species="s276" alias="sa520"/>

</celldesigner:baseReactants>

<celldesigner:baseProducts>

<celldesigner:baseProduct species="s1114" alias="sa525">

<celldesigner:linkAnchor position="NNE"/>

</celldesigner:baseProduct>

</celldesigner:baseProducts>

<celldesigner:connectScheme connectPolicy="direct" rectangleIndex="0">

<celldesigner:listOfLineDirection>

<celldesigner:lineDirection index="0" value="unknown"/>

</celldesigner:listOfLineDirection>

</celldesigner:connectScheme>

<celldesigner:line width="1.0" color="ff000000"/>

</celldesigner:extension>

</annotation>

<listOfReactants>

<speciesReference metaid="CDMT00342" species="s276">

<annotation>

<celldesigner:extension>

<celldesigner:alias>sa520</celldesigner:alias>

</celldesigner:extension>

</annotation>

</speciesReference>

</listOfReactants>

<listOfProducts>

<speciesReference metaid="CDMT00343" species="s1114">

<annotation>

<celldesigner:extension>

<celldesigner:alias>sa525</celldesigner:alias>

</celldesigner:extension>

</annotation>

</speciesReference>

</listOfProducts>

</reaction>

<reaction metaid="re263" id="re231" name="NsDPKB" reversible="false">

<annotation>

<celldesigner:extension>

<celldesigner:name>NsDPKB</celldesigner:name>

<celldesigner:reactionType>STATE_TRANSITION</celldesigner:reactionType>

<celldesigner:baseReactants>

<celldesigner:baseReactant species="s442" alias="sa527"/>

</celldesigner:baseReactants>

<celldesigner:baseProducts>

<celldesigner:baseProduct species="s1114" alias="sa525"/>

</celldesigner:baseProducts>

<celldesigner:connectScheme connectPolicy="direct" rectangleIndex="0">

<celldesigner:listOfLineDirection>

<celldesigner:lineDirection index="0" value="unknown"/>

</celldesigner:listOfLineDirection>

</celldesigner:connectScheme>

<celldesigner:line width="1.0" color="ffff3333"/>

</celldesigner:extension>

</annotation>

<listOfReactants>

<speciesReference metaid="CDMT00344" species="s442">

<annotation>

<celldesigner:extension>

<celldesigner:alias>sa527</celldesigner:alias>

</celldesigner:extension>

</annotation>

</speciesReference>

</listOfReactants>

<listOfProducts>

<speciesReference metaid="CDMT00345" species="s1114">

<annotation>

<celldesigner:extension>

<celldesigner:alias>sa525</celldesigner:alias>

</celldesigner:extension>

</annotation>

</speciesReference>

</listOfProducts>

</reaction>

<reaction metaid="re265" id="re232" name="UMP/CMPK2" reversible="false">

<annotation>

<celldesigner:extension>

<celldesigner:name>UMP/CMPK2</celldesigner:name>

<celldesigner:reactionType>STATE_TRANSITION</celldesigner:reactionType>

<celldesigner:baseReactants>

<celldesigner:baseReactant species="s278" alias="sa510">

<celldesigner:linkAnchor position="S"/>

</celldesigner:baseReactant>

</celldesigner:baseReactants>

<celldesigner:baseProducts>

<celldesigner:baseProduct species="s1012" alias="sa516"/>

</celldesigner:baseProducts>

<celldesigner:connectScheme connectPolicy="direct" rectangleIndex="0">

<celldesigner:listOfLineDirection>

<celldesigner:lineDirection index="0" value="unknown"/>

</celldesigner:listOfLineDirection>

</celldesigner:connectScheme>

<celldesigner:line width="1.0" color="ffff3333"/>

</celldesigner:extension>

</annotation>

<listOfReactants>

<speciesReference metaid="CDMT00346" species="s278">

<annotation>

<celldesigner:extension>

<celldesigner:alias>sa510</celldesigner:alias>

</celldesigner:extension>

</annotation>

</speciesReference>

</listOfReactants>

<listOfProducts>

<speciesReference metaid="CDMT00347" species="s1012">

<annotation>

<celldesigner:extension>

<celldesigner:alias>sa516</celldesigner:alias>

</celldesigner:extension>

</annotation>

</speciesReference>

</listOfProducts>

</reaction>

<reaction metaid="re266" id="re233" name="NsDPKB" reversible="false">

<annotation>

<celldesigner:extension>

<celldesigner:name>NsDPKB</celldesigner:name>

<celldesigner:reactionType>STATE_TRANSITION</celldesigner:reactionType>

<celldesigner:baseReactants>

<celldesigner:baseReactant species="s1013" alias="sa511"/>

</celldesigner:baseReactants>

<celldesigner:baseProducts>

<celldesigner:baseProduct species="s278" alias="sa510"/>

</celldesigner:baseProducts>

<celldesigner:connectScheme connectPolicy="direct" rectangleIndex="0">

<celldesigner:listOfLineDirection>

<celldesigner:lineDirection index="0" value="unknown"/>

</celldesigner:listOfLineDirection>

</celldesigner:connectScheme>

<celldesigner:line width="1.0" color="ffff3333"/>

</celldesigner:extension>

</annotation>

<listOfReactants>

<speciesReference metaid="CDMT00348" species="s1013">

<annotation>

<celldesigner:extension>

<celldesigner:alias>sa511</celldesigner:alias>

</celldesigner:extension>

</annotation>

</speciesReference>

</listOfReactants>

<listOfProducts>

<speciesReference metaid="CDMT00349" species="s278">

<annotation>

<celldesigner:extension>

<celldesigner:alias>sa510</celldesigner:alias>

</celldesigner:extension>

</annotation>
</speciesReference>
</listOfProducts>
</reaction>
<reaction metaid="re234" id="re234" name="dTMPK" reversible="false">
<annotation>
<celldesigner:extension>
<celldesigner:name>dTMPK</celldesigner:name>
<celldesigner:reactionType>STATE_TRANSITION</celldesigner:reactionType>
<celldesigner:baseReactants>
<celldesigner:baseReactant species="s1111" alias="sa524">
<celldesigner:linkAnchor position="W"/>
</celldesigner:baseReactant>
</celldesigner:baseReactants>
<celldesigner:baseProducts>
<celldesigner:baseProduct species="s1114" alias="sa525">
<celldesigner:linkAnchor position="ESE"/>
</celldesigner:baseProduct>
</celldesigner:baseProducts>
<celldesigner:connectScheme connectPolicy="direct" rectangleIndex="0">
<celldesigner:listOfLineDirection>
<celldesigner:lineDirection index="0" value="unknown"/>
</celldesigner:listOfLineDirection>
</celldesigner:connectScheme>
<celldesigner:line width="1.0" color="ff000000"/>
</celldesigner:extension>
</annotation>

<listOfReactants>
<speciesReference metaid="CDMT00350" species="s1111">
<annotation>
<celldesigner:extension>
<celldesigner:alias>sa524</celldesigner:alias>
</celldesigner:extension>
</annotation>
</speciesReference>
</listOfReactants>
<listOfProducts>
<speciesReference metaid="CDMT00351" species="s1114">
<annotation>
<celldesigner:extension>
<celldesigner:alias>sa525</celldesigner:alias>
</celldesigner:extension>
</annotation>
</speciesReference>
</listOfProducts>
</reaction>
<reaction metaid="re268" id="re235" name="NsDPKB" reversible="false">
<annotation>
<celldesigner:extension>
<celldesigner:name>NsDPKB</celldesigner:name>
<celldesigner:reactionType>STATE_TRANSITION</celldesigner:reactionType>
<celldesigner:baseReactants>
<celldesigner:baseReactant species="s1113" alias="sa526"/>
</celldesigner:baseReactants>

<celldesigner:baseProducts>

<celldesigner:baseProduct species="s1111" alias="sa524"/>

</celldesigner:baseProducts>

<celldesigner:connectScheme connectPolicy="direct" rectangleIndex="0">

<celldesigner:listOfLineDirection>

<celldesigner:lineDirection index="0" value="unknown"/>

</celldesigner:listOfLineDirection>

</celldesigner:connectScheme>

<celldesigner:line width="1.0" color="ff000000"/>

</celldesigner:extension>

</annotation>

<listOfReactants>

<speciesReference metaid="CDMT00352" species="s1113">

<annotation>

<celldesigner:extension>

<celldesigner:alias>sa526</celldesigner:alias>

</celldesigner:extension>

</annotation>

</speciesReference>

</listOfReactants>

<listOfProducts>

<speciesReference metaid="CDMT00353" species="s1111">

<annotation>

<celldesigner:extension>

<celldesigner:alias>sa524</celldesigner:alias>

</celldesigner:extension>

</annotation>

</speciesReference>

</listOfProducts>

</reaction>

<reaction metaid="re269" id="re236" name="dTMPK" reversible="false">

<annotation>

<celldesigner:extension>

<celldesigner:name>dTMPK</celldesigner:name>

<celldesigner:reactionType>STATE_TRANSITION</celldesigner:reactionType>

<celldesigner:baseReactants>

<celldesigner:baseReactant species="s1114" alias="sa525">

<celldesigner:linkAnchor position="NE"/>

</celldesigner:baseReactant>

</celldesigner:baseReactants>

<celldesigner:baseProducts>

<celldesigner:baseProduct species="s1051" alias="sa519"/>

</celldesigner:baseProducts>

<celldesigner:connectScheme connectPolicy="direct" rectangleIndex="1">

<celldesigner:listOfLineDirection>

<celldesigner:lineDirection index="0" value="unknown"/>

<celldesigner:lineDirection index="1" value="unknown"/>

</celldesigner:listOfLineDirection>

</celldesigner:connectScheme>

<celldesigner:editPoints>0.6923976391316157,-0.11944127858564002</celldesigner:editPoints>

<celldesigner:line width="1.0" color="ffff3333"/>

</celldesigner:extension>

</annotation>

<listOfReactants>

<speciesReference metaid="CDMT00354" species="s1114">
<annotation>
<celldesigner:extension>
<celldesigner:alias>sa525</celldesigner:alias>
</celldesigner:extension>
</annotation>
</speciesReference>
</listOfReactants>
<listOfProducts>
<speciesReference metaid="CDMT00355" species="s1051">
<annotation>
<celldesigner:extension>
<celldesigner:alias>sa519</celldesigner:alias>
</celldesigner:extension>
</annotation>
</speciesReference>
</listOfProducts>
</reaction>
<reaction metaid="re270" id="re237" name="RR" reversible="false">
<annotation>
<celldesigner:extension>
<celldesigner:name>RR</celldesigner:name>
<celldesigner:reactionType>STATE_TRANSITION</celldesigner:reactionType>
<celldesigner:baseReactants>
<celldesigner:baseReactant species="s278" alias="sa510"/>
</celldesigner:baseReactants>
<celldesigner:baseProducts>

<celldesigner:baseProduct species="s1108" alias="sa512">

<celldesigner:linkAnchor position="NNW"/>

</celldesigner:baseProduct>

</celldesigner:baseProducts>

<celldesigner:connectScheme connectPolicy="direct" rectangleIndex="0">

<celldesigner:listOfLineDirection>

<celldesigner:lineDirection index="0" value="unknown"/>

</celldesigner:listOfLineDirection>

</celldesigner:connectScheme>

<celldesigner:line width="1.0" color="ffff3333"/>

</celldesigner:extension>

</annotation>

<listOfReactants>

<speciesReference metaid="CDMT00356" species="s278">

<annotation>

<celldesigner:extension>

<celldesigner:alias>sa510</celldesigner:alias>

</celldesigner:extension>

</annotation>

</speciesReference>

</listOfReactants>

<listOfProducts>

<speciesReference metaid="CDMT00357" species="s1108">

<annotation>

<celldesigner:extension>

<celldesigner:alias>sa512</celldesigner:alias>

</celldesigner:extension>

</annotation>

</speciesReference>

</listOfProducts>

</reaction>

<reaction metaid="re271" id="re238" name="UMP/CMPK2" reversible="false">

<annotation>

<celldesigner:extension>

<celldesigner:name>UMP/CMPK2</celldesigner:name>

<celldesigner:reactionType>STATE_TRANSITION</celldesigner:reactionType>

<celldesigner:baseReactants>

<celldesigner:baseReactant species="s1108" alias="sa512"/>

</celldesigner:baseReactants>

<celldesigner:baseProducts>

<celldesigner:baseProduct species="s1050" alias="sa514"/>

</celldesigner:baseProducts>

<celldesigner:connectScheme connectPolicy="direct" rectangleIndex="0">

<celldesigner:listOfLineDirection>

<celldesigner:lineDirection index="0" value="unknown"/>

</celldesigner:listOfLineDirection>

</celldesigner:connectScheme>

<celldesigner:line width="1.0" color="ff000000"/>

</celldesigner:extension>

</annotation>

<listOfReactants>

<speciesReference metaid="CDMT00358" species="s1108">

<annotation>

<celldesigner:extension>

<celldesigner:alias>sa512</celldesigner:alias>

</celldesigner:extension>

</annotation>

</speciesReference>

</listOfReactants>

<listOfProducts>

<speciesReference metaid="CDMT00359" species="s1050">

<annotation>

<celldesigner:extension>

<celldesigner:alias>sa514</celldesigner:alias>

</celldesigner:extension>

</annotation>

</speciesReference>

</listOfProducts>

</reaction>

<reaction metaid="re279" id="re239" name="Uri/CytK" reversible="false">

<annotation>

<celldesigner:extension>

<celldesigner:name>Uri/CytK</celldesigner:name>

<celldesigner:reactionType>STATE_TRANSITION</celldesigner:reactionType>

<celldesigner:baseReactants>

<celldesigner:baseReactant species="s277" alias="sa515">

<celldesigner:linkAnchor position="ESE"/>

</celldesigner:baseReactant>

</celldesigner:baseReactants>

<celldesigner:baseProducts>

<celldesigner:baseProduct species="s1012" alias="sa516">

<celldesigner:linkAnchor position="WSW"/>

</celldesigner:baseProduct>

</celldesigner:baseProducts>

<celldesigner:connectScheme connectPolicy="direct" rectangleIndex="0">

<celldesigner:listOfLineDirection>

<celldesigner:lineDirection index="0" value="unknown"/>

</celldesigner:listOfLineDirection>

</celldesigner:connectScheme>

<celldesigner:line width="1.0" color="ffff3333"/>

</celldesigner:extension>

</annotation>

<listOfReactants>

<speciesReference metaid="CDMT00360" species="s277">

<annotation>

<celldesigner:extension>

<celldesigner:alias>sa515</celldesigner:alias>

</celldesigner:extension>

</annotation>

</speciesReference>

</listOfReactants>

<listOfProducts>

<speciesReference metaid="CDMT00361" species="s1012">

<annotation>

<celldesigner:extension>

<celldesigner:alias>sa516</celldesigner:alias>

</celldesigner:extension>

</annotation>

</speciesReference>

</listOfProducts>

</reaction>

<reaction metaid="re478" id="re240" name="HxGuaPRibTr" reversible="false">

<annotation>

<celldesigner:extension>

<celldesigner:name>HxGuaPRibTr</celldesigner:name>

<celldesigner:reactionType>STATE_TRANSITION</celldesigner:reactionType>

<celldesigner:baseReactants>

<celldesigner:baseReactant species="s443" alias="sa500"/>

</celldesigner:baseReactants>

<celldesigner:baseProducts>

<celldesigner:baseProduct species="s1100" alias="sa501">

<celldesigner:linkAnchor position="SW"/>

</celldesigner:baseProduct>

</celldesigner:baseProducts>

<celldesigner:listOfReactantLinks>

<celldesigner:reactantLink reactant="s433" alias="sa504" targetLineIndex="-1,0">

<celldesigner:connectScheme connectPolicy="direct">

<celldesigner:listOfLineDirection>

<celldesigner:lineDirection index="0" value="unknown"/>

</celldesigner:listOfLineDirection>

</celldesigner:connectScheme>

<celldesigner:line width="1.0" color="ffff3333" type="Straight"/>

</celldesigner:reactantLink>

</celldesigner:listOfReactantLinks>

<celldesigner:connectScheme connectPolicy="direct" rectangleIndex="0">

<celldesigner:listOfLineDirection>
<celldesigner:lineDirection index="0" value="unknown"/>
</celldesigner:listOfLineDirection>
</celldesigner:connectScheme>
<celldesigner:line width="1.0" color="ffff3333"/>
</celldesigner:extension>
</annotation>
<listOfReactants>
<speciesReference metaid="CDMT00362" species="s443">
<annotation>
<celldesigner:extension>
<celldesigner:alias>sa500</celldesigner:alias>
</celldesigner:extension>
</annotation>
</speciesReference>
<speciesReference metaid="CDMT00363" species="s433">
<annotation>
<celldesigner:extension>
<celldesigner:alias>sa504</celldesigner:alias>
</celldesigner:extension>
</annotation>
</speciesReference>
</listOfReactants>
<listOfProducts>
<speciesReference metaid="CDMT00364" species="s1100">
<annotation>
<celldesigner:extension>

<celldesigner:alias>sa501</celldesigner:alias>

</celldesigner:extension>

</annotation>

</speciesReference>

</listOfProducts>

</reaction>

<reaction metaid="re295" id="re241" name="XPRibTr" reversible="false">

<annotation>

<celldesigner:extension>

<celldesigner:name>XPRibTr</celldesigner:name>

<celldesigner:reactionType>STATE_TRANSITION</celldesigner:reactionType>

<celldesigner:baseReactants>

<celldesigner:baseReactant species="s445" alias="sa505"/>

</celldesigner:baseReactants>

<celldesigner:baseProducts>

<celldesigner:baseProduct species="s1095" alias="sa503">

<celldesigner:linkAnchor position="S"/>

</celldesigner:baseProduct>

</celldesigner:baseProducts>

<celldesigner:listOfReactantLinks>

<celldesigner:reactantLink reactant="s433" alias="sa504" targetLineIndex="-1,0">

<celldesigner:linkAnchor position="ENE"/>

<celldesigner:connectScheme connectPolicy="direct">

<celldesigner:listOfLineDirection>

<celldesigner:lineDirection index="0" value="unknown"/>

</celldesigner:listOfLineDirection>

</celldesigner:connectScheme>

<celldesigner:line width="1.0" color="ffff3333" type="Straight"/>

</celldesigner:reactantLink>

</celldesigner:listOfReactantLinks>

<celldesigner:connectScheme connectPolicy="direct" rectangleIndex="0">

<celldesigner:listOfLineDirection>

<celldesigner:lineDirection index="0" value="unknown"/>

</celldesigner:listOfLineDirection>

</celldesigner:connectScheme>

<celldesigner:line width="1.0" color="ffff3333"/>

</celldesigner:extension>

</annotation>

<listOfReactants>

<speciesReference metaid="CDMT00365" species="s445">

<annotation>

<celldesigner:extension>

<celldesigner:alias>sa505</celldesigner:alias>

</celldesigner:extension>

</annotation>

</speciesReference>

<speciesReference metaid="CDMT00366" species="s433">

<annotation>

<celldesigner:extension>

<celldesigner:alias>sa504</celldesigner:alias>

</celldesigner:extension>

</annotation>

</speciesReference>

</listOfReactants>

<listOfProducts>

<speciesReference metaid="CDMT00367" species="s1095">

<annotation>

<celldesigner:extension>

<celldesigner:alias>sa503</celldesigner:alias>

</celldesigner:extension>

</annotation>

</speciesReference>

</listOfProducts>

</reaction>

<reaction metaid="re107" id="re244" name="AlaATr[ala-pyr]" reversible="false">

<annotation>

<celldesigner:extension>

<celldesigner:name>AlaATr[ala-pyr]</celldesigner:name>

<celldesigner:reactionType>STATE_TRANSITION</celldesigner:reactionType>

<celldesigner:baseReactants>

<celldesigner:baseReactant species="s114" alias="sa532">

<celldesigner:linkAnchor position="N"/>

</celldesigner:baseReactant>

</celldesigner:baseReactants>

<celldesigner:baseProducts>

<celldesigner:baseProduct species="s2" alias="sa143">

<celldesigner:linkAnchor position="E"/>

</celldesigner:baseProduct>

</celldesigner:baseProducts>

<celldesigner:connectScheme connectPolicy="direct" rectangleIndex="0">

<celldesigner:listOfLineDirection>

<celldesigner:lineDirection index="0" value="unknown"/>
</celldesigner:listOfLineDirection>
</celldesigner:connectScheme>
<celldesigner:line width="1.0" color="ff3333ff"/>
</celldesigner:extension>
</annotation>
<listOfReactants>
<speciesReference metaid="CDMT00372" species="s114">
<annotation>
<celldesigner:extension>
<celldesigner:alias>sa532</celldesigner:alias>
</celldesigner:extension>
</annotation>
</speciesReference>
</listOfReactants>
<listOfProducts>
<speciesReference metaid="CDMT00373" species="s2">
<annotation>
<celldesigner:extension>
<celldesigner:alias>sa143</celldesigner:alias>
</celldesigner:extension>
</annotation>
</speciesReference>
</listOfProducts>
</reaction>
<reaction metaid="CDMT00374" id="re245" name="AlaATr[pyr-ala]" reversible="false">
<annotation>

<celldesigner:extension>

<celldesigner:name>AlaATr[pyr-ala]</celldesigner:name>

<celldesigner:reactionType>STATE_TRANSITION</celldesigner:reactionType>

<celldesigner:baseReactants>

<celldesigner:baseReactant species="s2" alias="sa143">

<celldesigner:linkAnchor position="SE"/>

</celldesigner:baseReactant>

</celldesigner:baseReactants>

<celldesigner:baseProducts>

<celldesigner:baseProduct species="s114" alias="sa532"/>

</celldesigner:baseProducts>

<celldesigner:connectScheme connectPolicy="direct" rectangleIndex="0">

<celldesigner:listOfLineDirection>

<celldesigner:lineDirection index="0" value="unknown"/>

<celldesigner:lineDirection index="1" value="unknown"/>

</celldesigner:listOfLineDirection>

</celldesigner:connectScheme>

<celldesigner:editPoints>0.9935694446444596,0.08474645133429082</celldesigner:editPoints>

<celldesigner:line width="1.0" color="ff3333ff"/>

</celldesigner:extension>

</annotation>

<listOfReactants>

<speciesReference metaid="CDMT00375" species="s2">

<annotation>

<celldesigner:extension>

<celldesigner:alias>sa143</celldesigner:alias>

</celldesigner:extension>

</annotation>

</speciesReference>

</listOfReactants>

<listOfProducts>

<speciesReference metaid="CDMT00376" species="s114">

<annotation>

<celldesigner:extension>

<celldesigner:alias>sa532</celldesigner:alias>

</celldesigner:extension>

</annotation>

</speciesReference>

</listOfProducts>

</reaction>

<reaction metaid="CDMT00377" id="re246" name="AspATra" reversible="false">

<annotation>

<celldesigner:extension>

<celldesigner:name>AspATra</celldesigner:name>

<celldesigner:reactionType>STATE_TRANSITION</celldesigner:reactionType>

<celldesigner:baseReactants>

<celldesigner:baseReactant species="s1004" alias="sa556">

<celldesigner:linkAnchor position="W"/>

</celldesigner:baseReactant>

</celldesigner:baseReactants>

<celldesigner:baseProducts>

<celldesigner:baseProduct species="s13" alias="sa243">

<celldesigner:linkAnchor position="SSE"/>

</celldesigner:baseProduct>

</celldesigner:baseProducts>

<celldesigner:connectScheme connectPolicy="direct" rectangleIndex="1">

<celldesigner:listOfLineDirection>

<celldesigner:lineDirection index="0" value="unknown"/>

<celldesigner:lineDirection index="1" value="unknown"/>

<celldesigner:lineDirection index="2" value="unknown"/>

</celldesigner:listOfLineDirection>

</celldesigner:connectScheme>

<celldesigner:editPoints>0.0639231670018594,0.06422237439254269 0.297987961168823,0.025363843001101782</celldesigner:editPoints>

<celldesigner:line width="1.0" color="ff00ff33"/>

</celldesigner:extension>

</annotation>

<listOfReactants>

<speciesReference metaid="CDMT00378" species="s1004">

<annotation>

<celldesigner:extension>

<celldesigner:alias>sa556</celldesigner:alias>

</celldesigner:extension>

</annotation>

</speciesReference>

</listOfReactants>

<listOfProducts>

<speciesReference metaid="CDMT00379" species="s13">

<annotation>

<celldesigner:extension>

<celldesigner:alias>sa243</celldesigner:alias>

</celldesigner:extension>

</annotation>
</speciesReference>
</listOfProducts>
</reaction>
<reaction metaid="re108" id="re247" name="GluDH" reversible="false">
<annotation>
<celldesigner:extension>
<celldesigner:name>GluDH</celldesigner:name>
<celldesigner:reactionType>STATE_TRANSITION</celldesigner:reactionType>
<celldesigner:baseReactants>
<celldesigner:baseReactant species="s16" alias="sa539"/>
</celldesigner:baseReactants>
<celldesigner:baseProducts>
<celldesigner:baseProduct species="s115" alias="sa533"/>
</celldesigner:baseProducts>
<celldesigner:connectScheme connectPolicy="direct" rectangleIndex="0">
<celldesigner:listOfLineDirection>
<celldesigner:lineDirection index="0" value="unknown"/>
</celldesigner:listOfLineDirection>
</celldesigner:connectScheme>
<celldesigner:line width="1.0" color="ff00ff33"/>
</celldesigner:extension>
</annotation>
<listOfReactants>
<speciesReference metaid="CDMT00380" species="s16">
<annotation>
<celldesigner:extension>

<celldesigner:alias>sa539</celldesigner:alias>

</celldesigner:extension>

</annotation>

</speciesReference>

</listOfReactants>

<listOfProducts>

<speciesReference metaid="CDMT00381" species="s115">

<annotation>

<celldesigner:extension>

<celldesigner:alias>sa533</celldesigner:alias>

</celldesigner:extension>

</annotation>

</speciesReference>

</listOfProducts>

</reaction>

<reaction metaid="re356" id="re248" name="P-SerPhosphatase" reversible="false">

<annotation>

<celldesigner:extension>

<celldesigner:name>P-SerPhosphatase</celldesigner:name>

<celldesigner:reactionType>STATE_TRANSITION</celldesigner:reactionType>

<celldesigner:baseReactants>

<celldesigner:baseReactant species="s836" alias="sa554"/>

</celldesigner:baseReactants>

<celldesigner:baseProducts>

<celldesigner:baseProduct species="s444" alias="sa538"/>

</celldesigner:baseProducts>

<celldesigner:connectScheme connectPolicy="direct" rectangleIndex="0">

<celldesigner:listOfLineDirection>

<celldesigner:lineDirection index="0" value="unknown"/>

</celldesigner:listOfLineDirection>

</celldesigner:connectScheme>

<celldesigner:line width="1.0" color="ff000000"/>

</celldesigner:extension>

</annotation>

<listOfReactants>

<speciesReference metaid="CDMT00382" species="s836">

<annotation>

<celldesigner:extension>

<celldesigner:alias>sa554</celldesigner:alias>

</celldesigner:extension>

</annotation>

</speciesReference>

</listOfReactants>

<listOfProducts>

<speciesReference metaid="CDMT00383" species="s444">

<annotation>

<celldesigner:extension>

<celldesigner:alias>sa538</celldesigner:alias>

</celldesigner:extension>

</annotation>

</speciesReference>

</listOfProducts>

</reaction>

<reaction metaid="re358" id="re249" name="SHMT (Gly Synthesis)" reversible="false">

<annotation>

<celldesigner:extension>

<celldesigner:name>SHMT (Gly Synthesis)</celldesigner:name>

<celldesigner:reactionType>STATE_TRANSITION</celldesigner:reactionType>

<celldesigner:baseReactants>

<celldesigner:baseReactant species="s444" alias="sa538"/>

</celldesigner:baseReactants>

<celldesigner:baseProducts>

<celldesigner:baseProduct species="s473" alias="sa536"/>

</celldesigner:baseProducts>

<celldesigner:connectScheme connectPolicy="direct" rectangleIndex="0">

<celldesigner:listOfLineDirection>

<celldesigner:lineDirection index="0" value="unknown"/>

</celldesigner:listOfLineDirection>

</celldesigner:connectScheme>

<celldesigner:line width="1.0" color="ff000000"/>

</celldesigner:extension>

</annotation>

<listOfReactants>

<speciesReference metaid="CDMT00384" species="s444">

<annotation>

<celldesigner:extension>

<celldesigner:alias>sa538</celldesigner:alias>

</celldesigner:extension>

</annotation>

</speciesReference>

</listOfReactants>

<listOfProducts>

<speciesReference metaid="CDMT00385" species="s473">

<annotation>

<celldesigner:extension>

<celldesigner:alias>sa536</celldesigner:alias>

</celldesigner:extension>

</annotation>

</speciesReference>

</listOfProducts>

</reaction>

<reaction metaid="re359" id="re250" name="CysS" reversible="false">

<annotation>

<celldesigner:extension>

<celldesigner:name>CysS</celldesigner:name>

<celldesigner:reactionType>STATE_TRANSITION</celldesigner:reactionType>

<celldesigner:baseReactants>

<celldesigner:baseReactant species="s837" alias="sa555"/>

</celldesigner:baseReactants>

<celldesigner:baseProducts>

<celldesigner:baseProduct species="s475" alias="sa537"/>

</celldesigner:baseProducts>

<celldesigner:connectScheme connectPolicy="direct" rectangleIndex="0">

<celldesigner:listOfLineDirection>

<celldesigner:lineDirection index="0" value="unknown"/>

</celldesigner:listOfLineDirection>

</celldesigner:connectScheme>

<celldesigner:line width="1.0" color="ff000000"/>

</celldesigner:extension>

</annotation>

<listOfReactants>

<speciesReference metaid="CDMT00386" species="s837">

<annotation>

<celldesigner:extension>

<celldesigner:alias>sa555</celldesigner:alias>

</celldesigner:extension>

</annotation>

</speciesReference>

</listOfReactants>

<listOfProducts>

<speciesReference metaid="CDMT00387" species="s475">

<annotation>

<celldesigner:extension>

<celldesigner:alias>sa537</celldesigner:alias>

</celldesigner:extension>

</annotation>

</speciesReference>

</listOfProducts>

</reaction>

<reaction metaid="re100" id="re251" name="MetS" reversible="false">

<annotation>

<celldesigner:extension>

<celldesigner:name>MetS</celldesigner:name>

<celldesigner:reactionType>STATE_TRANSITION</celldesigner:reactionType>

<celldesigner:baseReactants>

<celldesigner:baseReactant species="s845" alias="sa562"/>

</celldesigner:baseReactants>

<celldesigner:baseProducts>

<celldesigner:baseProduct species="s471" alias="sa534"/>

</celldesigner:baseProducts>

<celldesigner:listOfReactantLinks>

<celldesigner:reactantLink reactant="s474" alias="sa614" targetLineIndex="-1,0">

<celldesigner:linkAnchor position="NNW"/>

<celldesigner:connectScheme connectPolicy="direct">

<celldesigner:listOfLineDirection>

<celldesigner:lineDirection index="0" value="unknown"/>

</celldesigner:listOfLineDirection>

</celldesigner:connectScheme>

<celldesigner:line width="1.0" color="ff000000" type="Straight"/>

</celldesigner:reactantLink>

</celldesigner:listOfReactantLinks>

<celldesigner:listOfProductLinks>

<celldesigner:productLink product="s472" alias="sa535" targetLineIndex="-1,1">

<celldesigner:connectScheme connectPolicy="direct">

<celldesigner:listOfLineDirection>

<celldesigner:lineDirection index="0" value="unknown"/>

</celldesigner:listOfLineDirection>

</celldesigner:connectScheme>

<celldesigner:line width="1.0" color="ff000000" type="Straight"/>

</celldesigner:productLink>

</celldesigner:listOfProductLinks>

<celldesigner:connectScheme connectPolicy="direct" rectangleIndex="0">

```
<celldesigner:listOfLineDirection>
<celldesigner:lineDirection index="0" value="unknown"/>
</celldesigner:listOfLineDirection>
</celldesigner:connectScheme>
<celldesigner:line width="1.0" color="ff000000"/>
</celldesigner:extension>
</annotation>
<listOfReactants>
<speciesReference metaid="CDMT00388" species="s845">
<annotation>
<celldesigner:extension>
<celldesigner:alias>sa562</celldesigner:alias>
</celldesigner:extension>
</annotation>
</speciesReference>
<speciesReference metaid="CDMT00389" species="s474">
<annotation>
<celldesigner:extension>
<celldesigner:alias>sa614</celldesigner:alias>
</celldesigner:extension>
</annotation>
</speciesReference>
</listOfReactants>
<listOfProducts>
<speciesReference metaid="CDMT00390" species="s471">
<annotation>
<celldesigner:extension>
```

<celldesigner:alias>sa534</celldesigner:alias>

</celldesigner:extension>

</annotation>

</speciesReference>

<speciesReference metaid="CDMT00391" species="s472">

<annotation>

<celldesigner:extension>

<celldesigner:alias>sa535</celldesigner:alias>

</celldesigner:extension>

</annotation>

</speciesReference>

</listOfProducts>

</reaction>

<reaction metaid="re379" id="re252" name="ProCRD" reversible="false">

<annotation>

<celldesigner:extension>

<celldesigner:name>ProCRD</celldesigner:name>

<celldesigner:reactionType>STATE_TRANSITION</celldesigner:reactionType>

<celldesigner:baseReactants>

<celldesigner:baseReactant species="s843" alias="sa560"/>

</celldesigner:baseReactants>

<celldesigner:baseProducts>

<celldesigner:baseProduct species="s446" alias="sa540"/>

</celldesigner:baseProducts>

<celldesigner:connectScheme connectPolicy="direct" rectangleIndex="0">

<celldesigner:listOfLineDirection>

<celldesigner:lineDirection index="0" value="unknown"/>

</celldesigner:listOfLineDirection>

</celldesigner:connectScheme>

<celldesigner:line width="1.0" color="ff000000"/>

</celldesigner:extension>

</annotation>

<listOfReactants>

<speciesReference metaid="CDMT00392" species="s843">

<annotation>

<celldesigner:extension>

<celldesigner:alias>sa560</celldesigner:alias>

</celldesigner:extension>

</annotation>

</speciesReference>

</listOfReactants>

<listOfProducts>

<speciesReference metaid="CDMT00393" species="s446">

<annotation>

<celldesigner:extension>

<celldesigner:alias>sa540</celldesigner:alias>

</celldesigner:extension>

</annotation>

</speciesReference>

</listOfProducts>

</reaction>

<reaction metaid="re380" id="re253" name="GlnS" reversible="false">

<annotation>

<celldesigner:extension>

<celldesigner:name>GlnS</celldesigner:name>

<celldesigner:reactionType>STATE_TRANSITION</celldesigner:reactionType>

<celldesigner:baseReactants>

<celldesigner:baseReactant species="s115" alias="sa533">

<celldesigner:linkAnchor position="SW"/>

</celldesigner:baseReactant>

</celldesigner:baseReactants>

<celldesigner:baseProducts>

<celldesigner:baseProduct species="s979" alias="sa541"/>

</celldesigner:baseProducts>

<celldesigner:connectScheme connectPolicy="direct" rectangleIndex="1">

<celldesigner:listOfLineDirection>

<celldesigner:lineDirection index="0" value="unknown"/>

<celldesigner:lineDirection index="1" value="unknown"/>

</celldesigner:listOfLineDirection>

</celldesigner:connectScheme>

<celldesigner:editPoints>0.5753622358650102,-0.11570496301015387</celldesigner:editPoints>

<celldesigner:line width="1.0" color="ff000000"/>

</celldesigner:extension>

</annotation>

<listOfReactants>

<speciesReference metaid="CDMT00394" species="s115">

<annotation>

<celldesigner:extension>

<celldesigner:alias>sa533</celldesigner:alias>

</celldesigner:extension>

</annotation>

</speciesReference>
</listOfReactants>
<listOfProducts>
<speciesReference metaid="CDMT00395" species="s979">
<annotation>
<celldesigner:extension>
<celldesigner:alias>sa541</celldesigner:alias>
</celldesigner:extension>
</annotation>
</speciesReference>
</listOfProducts>
</reaction>
<reaction metaid="re381" id="re254" name="ThrAld" reversible="false">
<annotation>
<celldesigner:extension>
<celldesigner:name>ThrAld</celldesigner:name>
<celldesigner:reactionType>STATE_TRANSITION</celldesigner:reactionType>
<celldesigner:baseReactants>
<celldesigner:baseReactant species="s473" alias="sa536"/>
</celldesigner:baseReactants>
<celldesigner:baseProducts>
<celldesigner:baseProduct species="s490" alias="sa542"/>
</celldesigner:baseProducts>
<celldesigner:listOfReactantLinks>
<celldesigner:reactantLink reactant="s489" alias="sa543" targetLineIndex="-1,0">
<celldesigner:connectScheme connectPolicy="direct">
<celldesigner:listOfLineDirection>

<celldesigner:lineDirection index="0" value="unknown"/>

</celldesigner:listOfLineDirection>

</celldesigner:connectScheme>

<celldesigner:line width="1.0" color="ff000000" type="Straight"/>

</celldesigner:reactantLink>

</celldesigner:listOfReactantLinks>

<celldesigner:connectScheme connectPolicy="direct" rectangleIndex="0">

<celldesigner:listOfLineDirection>

<celldesigner:lineDirection index="0" value="unknown"/>

</celldesigner:listOfLineDirection>

</celldesigner:connectScheme>

<celldesigner:line width="1.0" color="ff000000"/>

</celldesigner:extension>

</annotation>

<listOfReactants>

<speciesReference metaid="CDMT00396" species="s473">

<annotation>

<celldesigner:extension>

<celldesigner:alias>sa536</celldesigner:alias>

</celldesigner:extension>

</annotation>

</speciesReference>

<speciesReference metaid="CDMT00397" species="s489">

<annotation>

<celldesigner:extension>

<celldesigner:alias>sa543</celldesigner:alias>

</celldesigner:extension>

</annotation>

</speciesReference>

</listOfReactants>

<listOfProducts>

<speciesReference metaid="CDMT00398" species="s490">

<annotation>

<celldesigner:extension>

<celldesigner:alias>sa542</celldesigner:alias>

</celldesigner:extension>

</annotation>

</speciesReference>

</listOfProducts>

</reaction>

<reaction metaid="re182" id="re255" name="AsnS" reversible="false">

<annotation>

<celldesigner:extension>

<celldesigner:name>AsnS</celldesigner:name>

<celldesigner:reactionType>STATE_TRANSITION</celldesigner:reactionType>

<celldesigner:baseReactants>

<celldesigner:baseReactant species="s1004" alias="sa556"/>

</celldesigner:baseReactants>

<celldesigner:baseProducts>

<celldesigner:baseProduct species="s198" alias="sa544"/>

</celldesigner:baseProducts>

<celldesigner:connectScheme connectPolicy="direct" rectangleIndex="0">

<celldesigner:listOfLineDirection>

<celldesigner:lineDirection index="0" value="unknown"/>

</celldesigner:listOfLineDirection>

</celldesigner:connectScheme>

<celldesigner:line width="1.0" color="ff000000"/>

</celldesigner:extension>

</annotation>

<listOfReactants>

<speciesReference metaid="CDMT00399" species="s1004">

<annotation>

<celldesigner:extension>

<celldesigner:alias>sa556</celldesigner:alias>

</celldesigner:extension>

</annotation>

</speciesReference>

</listOfReactants>

<listOfProducts>

<speciesReference metaid="CDMT00400" species="s198">

<annotation>

<celldesigner:extension>

<celldesigner:alias>sa544</celldesigner:alias>

</celldesigner:extension>

</annotation>

</speciesReference>

</listOfProducts>

</reaction>

<reaction metaid="re202" id="re256" name="PheH" reversible="false">

<annotation>

<celldesigner:extension>

<celldesigner:name>PheH</celldesigner:name>

<celldesigner:reactionType>STATE_TRANSITION</celldesigner:reactionType>

<celldesigner:baseReactants>

<celldesigner:baseReactant species="s215" alias="sa552"/>

</celldesigner:baseReactants>

<celldesigner:baseProducts>

<celldesigner:baseProduct species="s217" alias="sa546"/>

</celldesigner:baseProducts>

<celldesigner:connectScheme connectPolicy="direct" rectangleIndex="0">

<celldesigner:listOfLineDirection>

<celldesigner:lineDirection index="0" value="unknown"/>

</celldesigner:listOfLineDirection>

</celldesigner:connectScheme>

<celldesigner:line width="1.0" color="ff000000"/>

</celldesigner:extension>

</annotation>

<listOfReactants>

<speciesReference metaid="CDMT00401" species="s215">

<annotation>

<celldesigner:extension>

<celldesigner:alias>sa552</celldesigner:alias>

</celldesigner:extension>

</annotation>

</speciesReference>

</listOfReactants>

<listOfProducts>

<speciesReference metaid="CDMT00402" species="s217">

<annotation>

<celldesigner:extension>

<celldesigner:alias>sa546</celldesigner:alias>

</celldesigner:extension>

</annotation>

</speciesReference>

</listOfProducts>

</reaction>

<reaction metaid="re484" id="re257" name="DHFR-TS_2" reversible="false">

<annotation>

<celldesigner:extension>

<celldesigner:name>DHFR-TS_2</celldesigner:name>

<celldesigner:reactionType>STATE_TRANSITION</celldesigner:reactionType>

<celldesigner:baseReactants>

<celldesigner:baseReactant species="s525" alias="sa547"/>

</celldesigner:baseReactants>

<celldesigner:baseProducts>

<celldesigner:baseProduct species="s472" alias="sa535"/>

</celldesigner:baseProducts>

<celldesigner:connectScheme connectPolicy="direct" rectangleIndex="0">

<celldesigner:listOfLineDirection>

<celldesigner:lineDirection index="0" value="unknown"/>

</celldesigner:listOfLineDirection>

</celldesigner:connectScheme>

<celldesigner:line width="1.0" color="ff000000"/>

</celldesigner:extension>

</annotation>

<listOfReactants>

<speciesReference metaid="CDMT00403" species="s525">

<annotation>

<celldesigner:extension>

<celldesigner:alias>sa547</celldesigner:alias>

</celldesigner:extension>

</annotation>

</speciesReference>

</listOfReactants>

<listOfProducts>

<speciesReference metaid="CDMT00404" species="s472">

<annotation>

<celldesigner:extension>

<celldesigner:alias>sa535</celldesigner:alias>

</celldesigner:extension>

</annotation>

</speciesReference>

</listOfProducts>

</reaction>

<reaction metaid="re485" id="re258" name="DHFR-TS_1" reversible="false">

<annotation>

<celldesigner:extension>

<celldesigner:name>DHFR-TS_1</celldesigner:name>

<celldesigner:reactionType>STATE_TRANSITION</celldesigner:reactionType>

<celldesigner:baseReactants>

<celldesigner:baseReactant species="s526" alias="sa548"/>

</celldesigner:baseReactants>

<celldesigner:baseProducts>

<celldesigner:baseProduct species="s525" alias="sa547"/>

</celldesigner:baseProducts>

<celldesigner:connectScheme connectPolicy="direct" rectangleIndex="0">

<celldesigner:listOfLineDirection>

<celldesigner:lineDirection index="0" value="unknown"/>

</celldesigner:listOfLineDirection>

</celldesigner:connectScheme>

<celldesigner:line width="1.0" color="ff000000"/>

</celldesigner:extension>

</annotation>

<listOfReactants>

<speciesReference metaid="CDMT00405" species="s526">

<annotation>

<celldesigner:extension>

<celldesigner:alias>sa548</celldesigner:alias>

</celldesigner:extension>

</annotation>

</speciesReference>

</listOfReactants>

<listOfProducts>

<speciesReference metaid="CDMT00406" species="s525">

<annotation>

<celldesigner:extension>

<celldesigner:alias>sa547</celldesigner:alias>

</celldesigner:extension>

</annotation>

</speciesReference>

</listOfProducts>

</reaction>

<reaction metaid="re486" id="re259" name="FPGS_1" reversible="false">

<annotation>

<celldesigner:extension>

<celldesigner:name>FPGS_1</celldesigner:name>

<celldesigner:reactionType>STATE_TRANSITION</celldesigner:reactionType>

<celldesigner:baseReactants>

<celldesigner:baseReactant species="s527" alias="sa549"/>

</celldesigner:baseReactants>

<celldesigner:baseProducts>

<celldesigner:baseProduct species="s526" alias="sa548"/>

</celldesigner:baseProducts>

<celldesigner:connectScheme connectPolicy="direct" rectangleIndex="0">

<celldesigner:listOfLineDirection>

<celldesigner:lineDirection index="0" value="unknown"/>

</celldesigner:listOfLineDirection>

</celldesigner:connectScheme>

<celldesigner:line width="1.0" color="ff000000"/>

</celldesigner:extension>

</annotation>

<listOfReactants>

<speciesReference metaid="CDMT00407" species="s527">

<annotation>

<celldesigner:extension>

<celldesigner:alias>sa549</celldesigner:alias>

</celldesigner:extension>

</annotation>

</speciesReference>

</listOfReactants>

<listOfProducts>

<speciesReference metaid="CDMT00408" species="s526">

<annotation>

<celldesigner:extension>

<celldesigner:alias>sa548</celldesigner:alias>

</celldesigner:extension>

</annotation>

</speciesReference>

</listOfProducts>

</reaction>

<reaction metaid="re487" id="re260" name="FPGS_2" reversible="false">

<annotation>

<celldesigner:extension>

<celldesigner:name>FPGS_2</celldesigner:name>

<celldesigner:reactionType>STATE_TRANSITION</celldesigner:reactionType>

<celldesigner:baseReactants>

<celldesigner:baseReactant species="s472" alias="sa535"/>

</celldesigner:baseReactants>

<celldesigner:baseProducts>

<celldesigner:baseProduct species="s528" alias="sa550"/>

</celldesigner:baseProducts>

<celldesigner:connectScheme connectPolicy="direct" rectangleIndex="0">

<celldesigner:listOfLineDirection>

<celldesigner:lineDirection index="0" value="unknown"/>
</celldesigner:listOfLineDirection>
</celldesigner:connectScheme>
<celldesigner:line width="1.0" color="ff000000"/>
</celldesigner:extension>
</annotation>
<listOfReactants>
<speciesReference metaid="CDMT00409" species="s472">
<annotation>
<celldesigner:extension>
<celldesigner:alias>sa535</celldesigner:alias>
</celldesigner:extension>
</annotation>
</speciesReference>
</listOfReactants>
<listOfProducts>
<speciesReference metaid="CDMT00410" species="s528">
<annotation>
<celldesigner:extension>
<celldesigner:alias>sa550</celldesigner:alias>
</celldesigner:extension>
</annotation>
</speciesReference>
</listOfProducts>
</reaction>
<reaction metaid="re488" id="re261" name="FPGS_3" reversible="false">
<annotation>

<celldesigner:extension>

<celldesigner:name>FPGS_3</celldesigner:name>

<celldesigner:reactionType>STATE_TRANSITION</celldesigner:reactionType>

<celldesigner:baseReactants>

<celldesigner:baseReactant species="s528" alias="sa550"/>

</celldesigner:baseReactants>

<celldesigner:baseProducts>

<celldesigner:baseProduct species="s834" alias="sa551"/>

</celldesigner:baseProducts>

<celldesigner:connectScheme connectPolicy="direct" rectangleIndex="0">

<celldesigner:listOfLineDirection>

<celldesigner:lineDirection index="0" value="unknown"/>

</celldesigner:listOfLineDirection>

</celldesigner:connectScheme>

<celldesigner:line width="1.0" color="ff000000"/>

</celldesigner:extension>

</annotation>

<listOfReactants>

<speciesReference metaid="CDMT00411" species="s528">

<annotation>

<celldesigner:extension>

<celldesigner:alias>sa550</celldesigner:alias>

</celldesigner:extension>

</annotation>

</speciesReference>

</listOfReactants>

<listOfProducts>

<speciesReference metaid="CDMT00412" species="s834">
<annotation>
<celldesigner:extension>
<celldesigner:alias>sa551</celldesigner:alias>
</celldesigner:extension>
</annotation>
</speciesReference>
</listOfProducts>
</reaction>
<reaction metaid="CDMT00413" id="re262" name="D3PGADH-like" reversible="false">
<annotation>
<celldesigner:extension>
<celldesigner:name>D3PGADH-like</celldesigner:name>
<celldesigner:reactionType>STATE_TRANSITION</celldesigner:reactionType>
<celldesigner:baseReactants>
<celldesigner:baseReactant species="s64" alias="sa531"/>
</celldesigner:baseReactants>
<celldesigner:baseProducts>
<celldesigner:baseProduct species="s835" alias="sa553"/>
</celldesigner:baseProducts>
<celldesigner:connectScheme connectPolicy="direct" rectangleIndex="0">
<celldesigner:listOfLineDirection>
<celldesigner:lineDirection index="0" value="unknown"/>
</celldesigner:listOfLineDirection>
</celldesigner:connectScheme>
<celldesigner:line width="1.0" color="ff000000"/>
</celldesigner:extension>

</annotation>
<listOfReactants>
<speciesReference metaid="CDMT00414" species="s64">
<annotation>
<celldesigner:extension>
<celldesigner:alias>sa531</celldesigner:alias>
</celldesigner:extension>
</annotation>
</speciesReference>
</listOfReactants>
<listOfProducts>
<speciesReference metaid="CDMT00415" species="s835">
<annotation>
<celldesigner:extension>
<celldesigner:alias>sa553</celldesigner:alias>
</celldesigner:extension>
</annotation>
</speciesReference>
</listOfProducts>
</reaction>
<reaction metaid="CDMT00416" id="re263" name="3PSerAminoTr" reversible="false">
<annotation>
<celldesigner:extension>
<celldesigner:name>3PSerAminoTr</celldesigner:name>
<celldesigner:reactionType>STATE_TRANSITION</celldesigner:reactionType>
<celldesigner:baseReactants>
<celldesigner:baseReactant species="s835" alias="sa553"/>

</celldesigner:baseReactants>

<celldesigner:baseProducts>

<celldesigner:baseProduct species="s836" alias="sa554"/>

</celldesigner:baseProducts>

<celldesigner:listOfProductLinks>

<celldesigner:productLink product="s16" alias="sa539" targetLineIndex="-1,1">

<celldesigner:connectScheme connectPolicy="direct">

<celldesigner:listOfLineDirection>

<celldesigner:lineDirection index="0" value="unknown"/>

</celldesigner:listOfLineDirection>

</celldesigner:connectScheme>

<celldesigner:line width="1.0" color="ff000000" type="Straight"/>

</celldesigner:productLink>

</celldesigner:listOfProductLinks>

<celldesigner:connectScheme connectPolicy="direct" rectangleIndex="0">

<celldesigner:listOfLineDirection>

<celldesigner:lineDirection index="0" value="unknown"/>

</celldesigner:listOfLineDirection>

</celldesigner:connectScheme>

<celldesigner:line width="1.0" color="ff000000"/>

</celldesigner:extension>

</annotation>

<listOfReactants>

<speciesReference metaid="CDMT00417" species="s835">

<annotation>

<celldesigner:extension>

<celldesigner:alias>sa553</celldesigner:alias>

</celldesigner:extension>

</annotation>

</speciesReference>

</listOfReactants>

<listOfProducts>

<speciesReference metaid="CDMT00418" species="s836">

<annotation>

<celldesigner:extension>

<celldesigner:alias>sa554</celldesigner:alias>

</celldesigner:extension>

</annotation>

</speciesReference>

<speciesReference metaid="CDMT00419" species="s16">

<annotation>

<celldesigner:extension>

<celldesigner:alias>sa539</celldesigner:alias>

</celldesigner:extension>

</annotation>

</speciesReference>

</listOfProducts>

</reaction>

<reaction metaid="re264" id="re264" name="SAT" reversible="false">

<annotation>

<celldesigner:extension>

<celldesigner:name>SAT</celldesigner:name>

<celldesigner:reactionType>STATE_TRANSITION</celldesigner:reactionType>

<celldesigner:baseReactants>

<celldesigner:baseReactant species="s444" alias="sa538"/>

</celldesigner:baseReactants>

<celldesigner:baseProducts>

<celldesigner:baseProduct species="s837" alias="sa555"/>

</celldesigner:baseProducts>

<celldesigner:listOfReactantLinks>

<celldesigner:reactantLink reactant="s7" alias="sa597" targetLineIndex="-1,0">

<celldesigner:linkAnchor position="ENE"/>

<celldesigner:connectScheme connectPolicy="direct">

<celldesigner:listOfLineDirection>

<celldesigner:lineDirection index="0" value="unknown"/>

<celldesigner:lineDirection index="1" value="unknown"/>

<celldesigner:lineDirection index="2" value="unknown"/>

</celldesigner:listOfLineDirection>

</celldesigner:connectScheme>

<celldesigner:editPoints>0.13889057787835613,0.25077143858589057
0.7383120147043188,0.12045994851455921</celldesigner:editPoints>

<celldesigner:line width="1.0" color="ff000000" type="Straight"/>

</celldesigner:reactantLink>

</celldesigner:listOfReactantLinks>

<celldesigner:connectScheme connectPolicy="direct" rectangleIndex="0">

<celldesigner:listOfLineDirection>

<celldesigner:lineDirection index="0" value="unknown"/>

</celldesigner:listOfLineDirection>

</celldesigner:connectScheme>

<celldesigner:line width="1.0" color="ff000000"/>

</celldesigner:extension>

</annotation>

<listOfReactants>
<speciesReference metaid="CDMT00420" species="s444">
<annotation>
<celldesigner:extension>
<celldesigner:alias>sa538</celldesigner:alias>
</celldesigner:extension>
</annotation>
</speciesReference>
<speciesReference metaid="CDMT00421" species="s7">
<annotation>
<celldesigner:extension>
<celldesigner:alias>sa597</celldesigner:alias>
</celldesigner:extension>
</annotation>
</speciesReference>
</listOfReactants>
<listOfProducts>
<speciesReference metaid="CDMT00422" species="s837">
<annotation>
<celldesigner:extension>
<celldesigner:alias>sa555</celldesigner:alias>
</celldesigner:extension>
</annotation>
</speciesReference>
</listOfProducts>
</reaction>
<reaction metaid="CDMT00423" id="re265" name="SHly" reversible="false">

<annotation>

<celldesigner:extension>

<celldesigner:name>SHly</celldesigner:name>

<celldesigner:reactionType>STATE_TRANSITION</celldesigner:reactionType>

<celldesigner:baseReactants>

<celldesigner:baseReactant species="s444" alias="sa538"/>

</celldesigner:baseReactants>

<celldesigner:baseProducts>

<celldesigner:baseProduct species="s475" alias="sa537"/>

</celldesigner:baseProducts>

<celldesigner:connectScheme connectPolicy="direct" rectangleIndex="1">

<celldesigner:listOfLineDirection>

<celldesigner:lineDirection index="0" value="unknown"/>

<celldesigner:lineDirection index="1" value="unknown"/>

<celldesigner:lineDirection index="2" value="unknown"/>

</celldesigner:listOfLineDirection>

</celldesigner:connectScheme>

<celldesigner:editPoints>0.2270454303649192,0.1870411745930447 0.980317055005834,0.18278540270242472</celldesigner:editPoints>

<celldesigner:line width="1.0" color="ff000000"/>

</celldesigner:extension>

</annotation>

<listOfReactants>

<speciesReference metaid="CDMT00424" species="s444">

<annotation>

<celldesigner:extension>

<celldesigner:alias>sa538</celldesigner:alias>

</celldesigner:extension>

</annotation>
</speciesReference>
</listOfReactants>
<listOfProducts>
<speciesReference metaid="CDMT00425" species="s475">
<annotation>
<celldesigner:extension>
<celldesigner:alias>sa537</celldesigner:alias>
</celldesigner:extension>
</annotation>
</speciesReference>
</listOfProducts>
</reaction>
<reaction metaid="CDMT00426" id="re266" name="AspSalORD" reversible="false">
<annotation>
<celldesigner:extension>
<celldesigner:name>AspSalORD</celldesigner:name>
<celldesigner:reactionType>STATE_TRANSITION</celldesigner:reactionType>
<celldesigner:baseReactants>
<celldesigner:baseReactant species="s1004" alias="sa556"/>
</celldesigner:baseReactants>
<celldesigner:baseProducts>
<celldesigner:baseProduct species="s840" alias="sa557"/>
</celldesigner:baseProducts>
<celldesigner:connectScheme connectPolicy="direct" rectangleIndex="0">
<celldesigner:listOfLineDirection>
<celldesigner:lineDirection index="0" value="unknown"/>

</celldesigner:listOfLineDirection>

</celldesigner:connectScheme>

<celldesigner:line width="1.0" color="ff000000"/>

</celldesigner:extension>

</annotation>

<listOfReactants>

<speciesReference metaid="CDMT00427" species="s1004">

<annotation>

<celldesigner:extension>

<celldesigner:alias>sa556</celldesigner:alias>

</celldesigner:extension>

</annotation>

</speciesReference>

</listOfReactants>

<listOfProducts>

<speciesReference metaid="CDMT00428" species="s840">

<annotation>

<celldesigner:extension>

<celldesigner:alias>sa557</celldesigner:alias>

</celldesigner:extension>

</annotation>

</speciesReference>

</listOfProducts>

</reaction>

<reaction metaid="re514" id="re267" name="HSDH" reversible="false">

<annotation>

<celldesigner:extension>

<celldesigner:name>HSDH</celldesigner:name>

<celldesigner:reactionType>STATE_TRANSITION</celldesigner:reactionType>

<celldesigner:baseReactants>

<celldesigner:baseReactant species="s840" alias="sa557"/>

</celldesigner:baseReactants>

<celldesigner:baseProducts>

<celldesigner:baseProduct species="s841" alias="sa558"/>

</celldesigner:baseProducts>

<celldesigner:connectScheme connectPolicy="direct" rectangleIndex="0">

<celldesigner:listOfLineDirection>

<celldesigner:lineDirection index="0" value="unknown"/>

</celldesigner:listOfLineDirection>

</celldesigner:connectScheme>

<celldesigner:line width="1.0" color="ff000000"/>

</celldesigner:extension>

</annotation>

<listOfReactants>

<speciesReference metaid="CDMT00429" species="s840">

<annotation>

<celldesigner:extension>

<celldesigner:alias>sa557</celldesigner:alias>

</celldesigner:extension>

</annotation>

</speciesReference>

</listOfReactants>

<listOfProducts>

<speciesReference metaid="CDMT00430" species="s841">

<annotation>
<celldesigner:extension>
<celldesigner:alias>sa558</celldesigner:alias>
</celldesigner:extension>
</annotation>
</speciesReference>
</listOfProducts>
</reaction>
<reaction metaid="re515" id="re268" name="HSK" reversible="false">
<annotation>
<celldesigner:extension>
<celldesigner:name>HSK</celldesigner:name>
<celldesigner:reactionType>STATE_TRANSITION</celldesigner:reactionType>
<celldesigner:baseReactants>
<celldesigner:baseReactant species="s841" alias="sa558"/>
</celldesigner:baseReactants>
<celldesigner:baseProducts>
<celldesigner:baseProduct species="s842" alias="sa559"/>
</celldesigner:baseProducts>
<celldesigner:connectScheme connectPolicy="direct" rectangleIndex="0">
<celldesigner:listOfLineDirection>
<celldesigner:lineDirection index="0" value="unknown"/>
</celldesigner:listOfLineDirection>
</celldesigner:connectScheme>
<celldesigner:line width="1.0" color="ff000000"/>
</celldesigner:extension>
</annotation>

<listOfReactants>

<speciesReference metaid="CDMT00431" species="s841">

<annotation>

<celldesigner:extension>

<celldesigner:alias>sa558</celldesigner:alias>

</celldesigner:extension>

</annotation>

</speciesReference>

</listOfReactants>

<listOfProducts>

<speciesReference metaid="CDMT00432" species="s842">

<annotation>

<celldesigner:extension>

<celldesigner:alias>sa559</celldesigner:alias>

</celldesigner:extension>

</annotation>

</speciesReference>

</listOfProducts>

</reaction>

<reaction metaid="re516" id="re269" name="ThrS" reversible="false">

<annotation>

<celldesigner:extension>

<celldesigner:name>ThrS</celldesigner:name>

<celldesigner:reactionType>STATE_TRANSITION</celldesigner:reactionType>

<celldesigner:baseReactants>

<celldesigner:baseReactant species="s842" alias="sa559"/>

</celldesigner:baseReactants>

<celldesigner:baseProducts>

<celldesigner:baseProduct species="s490" alias="sa542"/>

</celldesigner:baseProducts>

<celldesigner:connectScheme connectPolicy="direct" rectangleIndex="0">

<celldesigner:listOfLineDirection>

<celldesigner:lineDirection index="0" value="unknown"/>

</celldesigner:listOfLineDirection>

</celldesigner:connectScheme>

<celldesigner:line width="1.0" color="ff000000"/>

</celldesigner:extension>

</annotation>

<listOfReactants>

<speciesReference metaid="CDMT00433" species="s842">

<annotation>

<celldesigner:extension>

<celldesigner:alias>sa559</celldesigner:alias>

</celldesigner:extension>

</annotation>

</speciesReference>

</listOfReactants>

<listOfProducts>

<speciesReference metaid="CDMT00434" species="s490">

<annotation>

<celldesigner:extension>

<celldesigner:alias>sa542</celldesigner:alias>

</celldesigner:extension>

</annotation>

</speciesReference>

</listOfProducts>

</reaction>

<reaction metaid="re517" id="re270" name="1PCDH" reversible="false">

<annotation>

<celldesigner:extension>

<celldesigner:name>1PCDH</celldesigner:name>

<celldesigner:reactionType>STATE_TRANSITION</celldesigner:reactionType>

<celldesigner:baseReactants>

<celldesigner:baseReactant species="s115" alias="sa533"/>

</celldesigner:baseReactants>

<celldesigner:baseProducts>

<celldesigner:baseProduct species="s843" alias="sa560"/>

</celldesigner:baseProducts>

<celldesigner:connectScheme connectPolicy="direct" rectangleIndex="0">

<celldesigner:listOfLineDirection>

<celldesigner:lineDirection index="0" value="unknown"/>

</celldesigner:listOfLineDirection>

</celldesigner:connectScheme>

<celldesigner:line width="1.0" color="ff000000"/>

</celldesigner:extension>

</annotation>

<listOfReactants>

<speciesReference metaid="CDMT00435" species="s115">

<annotation>

<celldesigner:extension>

<celldesigner:alias>sa533</celldesigner:alias>

</celldesigner:extension>

</annotation>

</speciesReference>

</listOfReactants>

<listOfProducts>

<speciesReference metaid="CDMT00436" species="s843">

<annotation>

<celldesigner:extension>

<celldesigner:alias>sa560</celldesigner:alias>

</celldesigner:extension>

</annotation>

</speciesReference>

</listOfProducts>

</reaction>

<reaction metaid="re518" id="re271" name="CysbS" reversible="false">

<annotation>

<celldesigner:extension>

<celldesigner:name>CysbS</celldesigner:name>

<celldesigner:reactionType>STATE_TRANSITION</celldesigner:reactionType>

<celldesigner:baseReactants>

<celldesigner:baseReactant species="s444" alias="sa538"/>

</celldesigner:baseReactants>

<celldesigner:baseProducts>

<celldesigner:baseProduct species="s844" alias="sa561"/>

</celldesigner:baseProducts>

<celldesigner:connectScheme connectPolicy="direct" rectangleIndex="0">

<celldesigner:listOfLineDirection>

<celldesigner:lineDirection index="0" value="unknown"/>

</celldesigner:listOfLineDirection>

</celldesigner:connectScheme>

<celldesigner:line width="1.0" color="ff000000"/>

</celldesigner:extension>

</annotation>

<listOfReactants>

<speciesReference metaid="CDMT00437" species="s444">

<annotation>

<celldesigner:extension>

<celldesigner:alias>sa538</celldesigner:alias>

</celldesigner:extension>

</annotation>

</speciesReference>

</listOfReactants>

<listOfProducts>

<speciesReference metaid="CDMT00438" species="s844">

<annotation>

<celldesigner:extension>

<celldesigner:alias>sa561</celldesigner:alias>

</celldesigner:extension>

</annotation>

</speciesReference>

</listOfProducts>

</reaction>

<reaction metaid="re519" id="re272" name="Cysly" reversible="false">

<annotation>

<celldesigner:extension>

<celldesigner:name>Cysly</celldesigner:name>

<celldesigner:reactionType>STATE_TRANSITION</celldesigner:reactionType>

<celldesigner:baseReactants>

<celldesigner:baseReactant species="s844" alias="sa561"/>

</celldesigner:baseReactants>

<celldesigner:baseProducts>

<celldesigner:baseProduct species="s845" alias="sa562"/>

</celldesigner:baseProducts>

<celldesigner:listOfProductLinks>

<celldesigner:productLink product="s2" alias="sa143" targetLineIndex="-1,1">

<celldesigner:connectScheme connectPolicy="direct">

<celldesigner:listOfLineDirection>

<celldesigner:lineDirection index="0" value="unknown"/>

<celldesigner:lineDirection index="1" value="unknown"/>

<celldesigner:lineDirection index="2" value="unknown"/>

</celldesigner:listOfLineDirection>

</celldesigner:connectScheme>

<celldesigner:editPoints>0.10479941441023888,0.1573669626690029
0.395477614066966,0.15534127503000905</celldesigner:editPoints>

<celldesigner:line width="1.0" color="ff3333ff" type="Straight"/>

</celldesigner:productLink>

</celldesigner:listOfProductLinks>

<celldesigner:connectScheme connectPolicy="direct" rectangleIndex="0">

<celldesigner:listOfLineDirection>

<celldesigner:lineDirection index="0" value="unknown"/>

</celldesigner:listOfLineDirection>

</celldesigner:connectScheme>

<celldesigner:line width="1.0" color="ff000000"/>

</celldesigner:extension>

</annotation>

<listOfReactants>

<speciesReference metaid="CDMT00439" species="s844">

<annotation>

<celldesigner:extension>

<celldesigner:alias>sa561</celldesigner:alias>

</celldesigner:extension>

</annotation>

</speciesReference>

</listOfReactants>

<listOfProducts>

<speciesReference metaid="CDMT00440" species="s845">

<annotation>

<celldesigner:extension>

<celldesigner:alias>sa562</celldesigner:alias>

</celldesigner:extension>

</annotation>

</speciesReference>

<speciesReference metaid="CDMT00441" species="s2">

<annotation>

<celldesigner:extension>

<celldesigner:alias>sa143</celldesigner:alias>

</celldesigner:extension>

</annotation>

</speciesReference>

</listOfProducts>
</reaction>
<reaction metaid="re520" id="re273" name="PheAtr" reversible="false">
<annotation>
<celldesigner:extension>
<celldesigner:name>PheAtr</celldesigner:name>
<celldesigner:reactionType>STATE_TRANSITION</celldesigner:reactionType>
<celldesigner:baseReactants>
<celldesigner:baseReactant species="s846" alias="sa563"/>
</celldesigner:baseReactants>
<celldesigner:baseProducts>
<celldesigner:baseProduct species="s215" alias="sa552"/>
</celldesigner:baseProducts>
<celldesigner:listOfReactantLinks>
<celldesigner:reactantLink reactant="s115" alias="sa533" targetLineIndex="-1,0">
<celldesigner:linkAnchor position="SSW"/>
<celldesigner:connectScheme connectPolicy="direct">
<celldesigner:listOfLineDirection>
<celldesigner:lineDirection index="0" value="unknown"/>
<celldesigner:lineDirection index="1" value="unknown"/>
</celldesigner:listOfLineDirection>
</celldesigner:connectScheme>
<celldesigner:editPoints>0.650491562762511,-0.35154659746242345</celldesigner:editPoints>
<celldesigner:line width="1.0" color="ff000000" type="Straight"/>
</celldesigner:reactantLink>
</celldesigner:listOfReactantLinks>
<celldesigner:connectScheme connectPolicy="direct" rectangleIndex="0">

<celldesigner:listOfLineDirection>

<celldesigner:lineDirection index="0" value="unknown"/>

</celldesigner:listOfLineDirection>

</celldesigner:connectScheme>

<celldesigner:line width="1.0" color="ff000000"/>

</celldesigner:extension>

</annotation>

<listOfReactants>

<speciesReference metaid="CDMT00442" species="s846">

<annotation>

<celldesigner:extension>

<celldesigner:alias>sa563</celldesigner:alias>

</celldesigner:extension>

</annotation>

</speciesReference>

<speciesReference metaid="CDMT00443" species="s115">

<annotation>

<celldesigner:extension>

<celldesigner:alias>sa533</celldesigner:alias>

</celldesigner:extension>

</annotation>

</speciesReference>

</listOfReactants>

<listOfProducts>

<speciesReference metaid="CDMT00444" species="s215">

<annotation>

<celldesigner:extension>

<celldesigner:alias>sa552</celldesigner:alias>

</celldesigner:extension>

</annotation>

</speciesReference>

</listOfProducts>

</reaction>

<reaction metaid="re521" id="re274" name="TyrAtr" reversible="false">

<annotation>

<celldesigner:extension>

<celldesigner:name>TyrAtr</celldesigner:name>

<celldesigner:reactionType>STATE_TRANSITION</celldesigner:reactionType>

<celldesigner:baseReactants>

<celldesigner:baseReactant species="s217" alias="sa546"/>

</celldesigner:baseReactants>

<celldesigner:baseProducts>

<celldesigner:baseProduct species="s847" alias="sa564"/>

</celldesigner:baseProducts>

<celldesigner:connectScheme connectPolicy="direct" rectangleIndex="0">

<celldesigner:listOfLineDirection>

<celldesigner:lineDirection index="0" value="unknown"/>

</celldesigner:listOfLineDirection>

</celldesigner:connectScheme>

<celldesigner:line width="1.0" color="ff000000"/>

</celldesigner:extension>

</annotation>

<listOfReactants>

<speciesReference metaid="CDMT00445" species="s217">

<annotation>
<celldesigner:extension>
<celldesigner:alias>sa546</celldesigner:alias>
</celldesigner:extension>
</annotation>
</speciesReference>
</listOfReactants>
<listOfProducts>
<speciesReference metaid="CDMT00446" species="s847">
<annotation>
<celldesigner:extension>
<celldesigner:alias>sa564</celldesigner:alias>
</celldesigner:extension>
</annotation>
</speciesReference>
</listOfProducts>
</reaction>
<reaction metaid="re522" id="re275" name="LeuAtr" reversible="false">
<annotation>
<celldesigner:extension>
<celldesigner:name>LeuAtr</celldesigner:name>
<celldesigner:reactionType>STATE_TRANSITION</celldesigner:reactionType>
<celldesigner:baseReactants>
<celldesigner:baseReactant species="s848" alias="sa565"/>
</celldesigner:baseReactants>
<celldesigner:baseProducts>
<celldesigner:baseProduct species="s849" alias="sa566"/>

</celldesigner:baseProducts>

<celldesigner:listOfReactantLinks>

<celldesigner:reactantLink reactant="s115" alias="sa533" targetLineIndex="-1,0">

<celldesigner:connectScheme connectPolicy="direct">

<celldesigner:listOfLineDirection>

<celldesigner:lineDirection index="0" value="unknown"/>

</celldesigner:listOfLineDirection>

</celldesigner:connectScheme>

<celldesigner:line width="1.0" color="ff000000" type="Straight"/>

</celldesigner:reactantLink>

</celldesigner:listOfReactantLinks>

<celldesigner:connectScheme connectPolicy="direct" rectangleIndex="0">

<celldesigner:listOfLineDirection>

<celldesigner:lineDirection index="0" value="unknown"/>

</celldesigner:listOfLineDirection>

</celldesigner:connectScheme>

<celldesigner:line width="1.0" color="ff000000"/>

</celldesigner:extension>

</annotation>

<listOfReactants>

<speciesReference metaid="CDMT00447" species="s848">

<annotation>

<celldesigner:extension>

<celldesigner:alias>sa565</celldesigner:alias>

</celldesigner:extension>

</annotation>

</speciesReference>

<speciesReference metaid="CDMT00448" species="s115">
<annotation>
<celldesigner:extension>
<celldesigner:alias>sa533</celldesigner:alias>
</celldesigner:extension>
</annotation>
</speciesReference>
</listOfReactants>
<listOfProducts>
<speciesReference metaid="CDMT00449" species="s849">
<annotation>
<celldesigner:extension>
<celldesigner:alias>sa566</celldesigner:alias>
</celldesigner:extension>
</annotation>
</speciesReference>
</listOfProducts>
</reaction>
<reaction metaid="re523" id="re276" name="ValAtr" reversible="false">
<annotation>
<celldesigner:extension>
<celldesigner:name>ValAtr</celldesigner:name>
<celldesigner:reactionType>STATE_TRANSITION</celldesigner:reactionType>
<celldesigner:baseReactants>
<celldesigner:baseReactant species="s850" alias="sa567"/>
</celldesigner:baseReactants>
<celldesigner:baseProducts>

<celldesigner:baseProduct species="s851" alias="sa568"/>
</celldesigner:baseProducts>
<celldesigner:listOfReactantLinks>
<celldesigner:reactantLink reactant="s115" alias="sa533" targetLineIndex="-1,0">
<celldesigner:connectScheme connectPolicy="direct">
<celldesigner:listOfLineDirection>
<celldesigner:lineDirection index="0" value="unknown"/>
</celldesigner:listOfLineDirection>
</celldesigner:connectScheme>
<celldesigner:line width="1.0" color="ff000000" type="Straight"/>
</celldesigner:reactantLink>
</celldesigner:listOfReactantLinks>
<celldesigner:connectScheme connectPolicy="direct" rectangleIndex="0">
<celldesigner:listOfLineDirection>
<celldesigner:lineDirection index="0" value="unknown"/>
</celldesigner:listOfLineDirection>
</celldesigner:connectScheme>
<celldesigner:line width="1.0" color="ff000000"/>
</celldesigner:extension>
</annotation>
<listOfReactants>
<speciesReference metaid="CDMT00450" species="s850">
<annotation>
<celldesigner:extension>
<celldesigner:alias>sa567</celldesigner:alias>
</celldesigner:extension>
</annotation>

</speciesReference>
<speciesReference metaid="CDMT00451" species="s115">
<annotation>
<celldesigner:extension>
<celldesigner:alias>sa533</celldesigner:alias>
</celldesigner:extension>
</annotation>
</speciesReference>
</listOfReactants>
<listOfProducts>
<speciesReference metaid="CDMT00452" species="s851">
<annotation>
<celldesigner:extension>
<celldesigner:alias>sa568</celldesigner:alias>
</celldesigner:extension>
</annotation>
</speciesReference>
</listOfProducts>
</reaction>
<reaction metaid="re524" id="re277" name="IleAtr" reversible="false">
<annotation>
<celldesigner:extension>
<celldesigner:name>IleAtr</celldesigner:name>
<celldesigner:reactionType>STATE_TRANSITION</celldesigner:reactionType>
<celldesigner:baseReactants>
<celldesigner:baseReactant species="s852" alias="sa569"/>
</celldesigner:baseReactants>

<celldesigner:baseProducts>

<celldesigner:baseProduct species="s853" alias="sa570"/>

</celldesigner:baseProducts>

<celldesigner:listOfReactantLinks>

<celldesigner:reactantLink reactant="s115" alias="sa533" targetLineIndex="-1,0">

<celldesigner:connectScheme connectPolicy="direct">

<celldesigner:listOfLineDirection>

<celldesigner:lineDirection index="0" value="unknown"/>

</celldesigner:listOfLineDirection>

</celldesigner:connectScheme>

<celldesigner:line width="1.0" color="ff000000" type="Straight"/>

</celldesigner:reactantLink>

</celldesigner:listOfReactantLinks>

<celldesigner:connectScheme connectPolicy="direct" rectangleIndex="0">

<celldesigner:listOfLineDirection>

<celldesigner:lineDirection index="0" value="unknown"/>

</celldesigner:listOfLineDirection>

</celldesigner:connectScheme>

<celldesigner:line width="1.0" color="ff000000"/>

</celldesigner:extension>

</annotation>

<listOfReactants>

<speciesReference metaid="CDMT00453" species="s852">

<annotation>

<celldesigner:extension>

<celldesigner:alias>sa569</celldesigner:alias>

</celldesigner:extension>

</annotation>
</speciesReference>
<speciesReference metaid="CDMT00454" species="s115">
<annotation>
<celldesigner:extension>
<celldesigner:alias>sa533</celldesigner:alias>
</celldesigner:extension>
</annotation>
</speciesReference>
</listOfReactants>
<listOfProducts>
<speciesReference metaid="CDMT00455" species="s853">
<annotation>
<celldesigner:extension>
<celldesigner:alias>sa570</celldesigner:alias>
</celldesigner:extension>
</annotation>
</speciesReference>
</listOfProducts>
</reaction>
<reaction metaid="re240" id="re278" name="TrpORD" reversible="false">
<annotation>
<celldesigner:extension>
<celldesigner:name>TrpORD</celldesigner:name>
<celldesigner:reactionType>STATE_TRANSITION</celldesigner:reactionType>
<celldesigner:baseReactants>
<celldesigner:baseReactant species="s231" alias="sa545"/>

</celldesigner:baseReactants>

<celldesigner:baseProducts>

<celldesigner:baseProduct species="s256" alias="sa571"/>

</celldesigner:baseProducts>

<celldesigner:connectScheme connectPolicy="direct" rectangleIndex="0">

<celldesigner:listOfLineDirection>

<celldesigner:lineDirection index="0" value="unknown"/>

</celldesigner:listOfLineDirection>

</celldesigner:connectScheme>

<celldesigner:line width="1.0" color="ff000000"/>

</celldesigner:extension>

</annotation>

<listOfReactants>

<speciesReference metaid="CDMT00456" species="s231">

<annotation>

<celldesigner:extension>

<celldesigner:alias>sa545</celldesigner:alias>

</celldesigner:extension>

</annotation>

</speciesReference>

</listOfReactants>

<listOfProducts>

<speciesReference metaid="CDMT00457" species="s256">

<annotation>

<celldesigner:extension>

<celldesigner:alias>sa571</celldesigner:alias>

</celldesigner:extension>

</annotation>

</speciesReference>

</listOfProducts>

</reaction>

<reaction metaid="re241" id="re279" name="NFLKAH" reversible="false">

<annotation>

<celldesigner:extension>

<celldesigner:name>NFLKAH</celldesigner:name>

<celldesigner:reactionType>STATE_TRANSITION</celldesigner:reactionType>

<celldesigner:baseReactants>

<celldesigner:baseReactant species="s256" alias="sa571"/>

</celldesigner:baseReactants>

<celldesigner:baseProducts>

<celldesigner:baseProduct species="s257" alias="sa572"/>

</celldesigner:baseProducts>

<celldesigner:listOfProductLinks>

<celldesigner:productLink product="s476" alias="sa573" targetLineIndex="-1,1">

<celldesigner:connectScheme connectPolicy="direct">

<celldesigner:listOfLineDirection>

<celldesigner:lineDirection index="0" value="unknown"/>

</celldesigner:listOfLineDirection>

</celldesigner:connectScheme>

<celldesigner:line width="1.0" color="ff000000" type="Straight"/>

</celldesigner:productLink>

</celldesigner:listOfProductLinks>

<celldesigner:connectScheme connectPolicy="direct" rectangleIndex="0">

<celldesigner:listOfLineDirection>

<celldesigner:lineDirection index="0" value="unknown"/>
</celldesigner:listOfLineDirection>
</celldesigner:connectScheme>
<celldesigner:line width="1.0" color="ff000000"/>
</celldesigner:extension>
</annotation>
<listOfReactants>
<speciesReference metaid="CDMT00458" species="s256">
<annotation>
<celldesigner:extension>
<celldesigner:alias>sa571</celldesigner:alias>
</celldesigner:extension>
</annotation>
</speciesReference>
</listOfReactants>
<listOfProducts>
<speciesReference metaid="CDMT00459" species="s257">
<annotation>
<celldesigner:extension>
<celldesigner:alias>sa572</celldesigner:alias>
</celldesigner:extension>
</annotation>
</speciesReference>
<speciesReference metaid="CDMT00460" species="s476">
<annotation>
<celldesigner:extension>
<celldesigner:alias>sa573</celldesigner:alias>

</celldesigner:extension>

</annotation>

</speciesReference>

</listOfProducts>

</reaction>

<reaction metaid="re242" id="re280" name="LKH" reversible="false">

<annotation>

<celldesigner:extension>

<celldesigner:name>LKH</celldesigner:name>

<celldesigner:reactionType>STATE_TRANSITION</celldesigner:reactionType>

<celldesigner:baseReactants>

<celldesigner:baseReactant species="s257" alias="sa572"/>

</celldesigner:baseReactants>

<celldesigner:baseProducts>

<celldesigner:baseProduct species="s491" alias="sa574"/>

</celldesigner:baseProducts>

<celldesigner:listOfProductLinks>

<celldesigner:productLink product="s114" alias="sa532" targetLineIndex="-1,1">

<celldesigner:connectScheme connectPolicy="direct">

<celldesigner:listOfLineDirection>

<celldesigner:lineDirection index="0" value="unknown"/>

</celldesigner:listOfLineDirection>

</celldesigner:connectScheme>

<celldesigner:line width="1.0" color="ff000000" type="Straight"/>

</celldesigner:productLink>

</celldesigner:listOfProductLinks>

<celldesigner:connectScheme connectPolicy="direct" rectangleIndex="0">

<celldesigner:listOfLineDirection>

<celldesigner:lineDirection index="0" value="unknown"/>

</celldesigner:listOfLineDirection>

</celldesigner:connectScheme>

<celldesigner:line width="1.0" color="ff000000"/>

</celldesigner:extension>

</annotation>

<listOfReactants>

<speciesReference metaid="CDMT00461" species="s257">

<annotation>

<celldesigner:extension>

<celldesigner:alias>sa572</celldesigner:alias>

</celldesigner:extension>

</annotation>

</speciesReference>

</listOfReactants>

<listOfProducts>

<speciesReference metaid="CDMT00462" species="s491">

<annotation>

<celldesigner:extension>

<celldesigner:alias>sa574</celldesigner:alias>

</celldesigner:extension>

</annotation>

</speciesReference>

<speciesReference metaid="CDMT00463" species="s114">

<annotation>

<celldesigner:extension>

<celldesigner:alias>sa532</celldesigner:alias>

</celldesigner:extension>

</annotation>

</speciesReference>

</listOfProducts>

</reaction>

<reaction metaid="re281" id="re281" name="BCAAT(val)" reversible="false">

<notes>

<html xmlns="http://www.w3.org/1999/xhtml">

<head>

<title/>

</head>

<body>ValMetabolism

</body>

</html>

</notes>

<annotation>

<celldesigner:extension>

<celldesigner:name>BCAAT(val)</celldesigner:name>

<celldesigner:reactionType>STATE_TRANSITION</celldesigner:reactionType>

<celldesigner:baseReactants>

<celldesigner:baseReactant species="s851" alias="sa568"/>

</celldesigner:baseReactants>

<celldesigner:baseProducts>

<celldesigner:baseProduct species="s246" alias="sa578"/>

</celldesigner:baseProducts>

<celldesigner:connectScheme connectPolicy="direct" rectangleIndex="0">

<celldesigner:listOfLineDirection>
<celldesigner:lineDirection index="0" value="unknown"/>
</celldesigner:listOfLineDirection>
</celldesigner:connectScheme>
<celldesigner:line width="1.0" color="ff000000"/>
</celldesigner:extension>
</annotation>
<listOfReactants>
<speciesReference metaid="CDMT00464" species="s851">
<annotation>
<celldesigner:extension>
<celldesigner:alias>sa568</celldesigner:alias>
</celldesigner:extension>
</annotation>
</speciesReference>
</listOfReactants>
<listOfProducts>
<speciesReference metaid="CDMT00465" species="s246">
<annotation>
<celldesigner:extension>
<celldesigner:alias>sa578</celldesigner:alias>
</celldesigner:extension>
</annotation>
</speciesReference>
</listOfProducts>
</reaction>
<reaction metaid="re227" id="re282" name="BCAAT(ile)" reversible="false">

<notes>
<html xmlns="http://www.w3.org/1999/xhtml">
<head>
<title/>
</head>
<body>IleMetabolism
</body>
</html>
</notes>
<annotation>
<celldesigner:extension>
<celldesigner:name>BCAAT(ile)</celldesigner:name>
<celldesigner:reactionType>STATE_TRANSITION</celldesigner:reactionType>
<celldesigner:baseReactants>
<celldesigner:baseReactant species="s853" alias="sa570">
<celldesigner:linkAnchor position="SSW"/>
</celldesigner:baseReactant>
</celldesigner:baseReactants>
<celldesigner:baseProducts>
<celldesigner:baseProduct species="s247" alias="sa579"/>
</celldesigner:baseProducts>
<celldesigner:connectScheme connectPolicy="direct" rectangleIndex="0">
<celldesigner:listOfLineDirection>
<celldesigner:lineDirection index="0" value="unknown"/>
</celldesigner:listOfLineDirection>
</celldesigner:connectScheme>
<celldesigner:line width="1.0" color="ff000000"/>

</celldesigner:extension>

</annotation>

<listOfReactants>

<speciesReference metaid="CDMT00466" species="s853">

<annotation>

<celldesigner:extension>

<celldesigner:alias>sa570</celldesigner:alias>

</celldesigner:extension>

</annotation>

</speciesReference>

</listOfReactants>

<listOfProducts>

<speciesReference metaid="CDMT00467" species="s247">

<annotation>

<celldesigner:extension>

<celldesigner:alias>sa579</celldesigner:alias>

</celldesigner:extension>

</annotation>

</speciesReference>

</listOfProducts>

</reaction>

<reaction metaid="re228" id="re283" name="BCAAT(leu)" reversible="false">

<notes>

<html xmlns="http://www.w3.org/1999/xhtml">

<head>

<title/>

</head>

<body>LeuMetabolism

</body>

</html>

</notes>

<annotation>

<celldesigner:extension>

<celldesigner:name>BCAAT(leu)</celldesigner:name>

<celldesigner:reactionType>STATE_TRANSITION</celldesigner:reactionType>

<celldesigner:baseReactants>

<celldesigner:baseReactant species="s849" alias="sa566"/>

</celldesigner:baseReactants>

<celldesigner:baseProducts>

<celldesigner:baseProduct species="s248" alias="sa580"/>

</celldesigner:baseProducts>

<celldesigner:connectScheme connectPolicy="direct" rectangleIndex="0">

<celldesigner:listOfLineDirection>

<celldesigner:lineDirection index="0" value="unknown"/>

</celldesigner:listOfLineDirection>

</celldesigner:connectScheme>

<celldesigner:line width="1.0" color="ff000000"/>

</celldesigner:extension>

</annotation>

<listOfReactants>

<speciesReference metaid="CDMT00468" species="s849">

<annotation>

<celldesigner:extension>

<celldesigner:alias>sa566</celldesigner:alias>

</celldesigner:extension>

</annotation>

</speciesReference>

</listOfReactants>

<listOfProducts>

<speciesReference metaid="CDMT00469" species="s248">

<annotation>

<celldesigner:extension>

<celldesigner:alias>sa580</celldesigner:alias>

</celldesigner:extension>

</annotation>

</speciesReference>

</listOfProducts>

</reaction>

<reaction metaid="re229" id="re284" name="MBTCoaSynthesis(ile)" reversible="false">

<notes>

<html xmlns="http://www.w3.org/1999/xhtml">

<head>

<title/>

</head>

<body>IleMetabolism

</body>

</html>

</notes>

<annotation>

<celldesigner:extension>

<celldesigner:name>MBTCoaSynthesis(ile)</celldesigner:name>

<celldesigner:reactionType>STATE_TRANSITION</celldesigner:reactionType>

<celldesigner:baseReactants>

<celldesigner:baseReactant species="s247" alias="sa579"/>

</celldesigner:baseReactants>

<celldesigner:baseProducts>

<celldesigner:baseProduct species="s389" alias="sa582"/>

</celldesigner:baseProducts>

<celldesigner:connectScheme connectPolicy="direct" rectangleIndex="0">

<celldesigner:listOfLineDirection>

<celldesigner:lineDirection index="0" value="unknown"/>

</celldesigner:listOfLineDirection>

</celldesigner:connectScheme>

<celldesigner:line width="1.0" color="ff000000"/>

</celldesigner:extension>

</annotation>

<listOfReactants>

<speciesReference metaid="CDMT00470" species="s247">

<annotation>

<celldesigner:extension>

<celldesigner:alias>sa579</celldesigner:alias>

</celldesigner:extension>

</annotation>

</speciesReference>

</listOfReactants>

<listOfProducts>

<speciesReference metaid="CDMT00471" species="s389">

<annotation>

<celldesigner:extension>

<celldesigner:alias>sa582</celldesigner:alias>

</celldesigner:extension>

</annotation>

</speciesReference>

</listOfProducts>

</reaction>

<reaction metaid="re230" id="re285" name="MBTCoaSynthesis(leu)" reversible="false">

<notes>

<html xmlns="http://www.w3.org/1999/xhtml">

<head>

<title/>

</head>

<body>LeuMetabolism

</body>

</html>

</notes>

<annotation>

<celldesigner:extension>

<celldesigner:name>MBTCoaSynthesis(leu)</celldesigner:name>

<celldesigner:reactionType>STATE_TRANSITION</celldesigner:reactionType>

<celldesigner:baseReactants>

<celldesigner:baseReactant species="s248" alias="sa580"/>

</celldesigner:baseReactants>

<celldesigner:baseProducts>

<celldesigner:baseProduct species="s249" alias="sa581"/>

</celldesigner:baseProducts>

<celldesigner:connectScheme connectPolicy="direct" rectangleIndex="0">
<celldesigner:listOfLineDirection>
<celldesigner:lineDirection index="0" value="unknown"/>
</celldesigner:listOfLineDirection>
</celldesigner:connectScheme>
<celldesigner:line width="1.0" color="ff000000"/>
</celldesigner:extension>
</annotation>
<listOfReactants>
<speciesReference metaid="CDMT00472" species="s248">
<annotation>
<celldesigner:extension>
<celldesigner:alias>sa580</celldesigner:alias>
</celldesigner:extension>
</annotation>
</speciesReference>
</listOfReactants>
<listOfProducts>
<speciesReference metaid="CDMT00473" species="s249">
<annotation>
<celldesigner:extension>
<celldesigner:alias>sa581</celldesigner:alias>
</celldesigner:extension>
</annotation>
</speciesReference>
</listOfProducts>
</reaction>

<reaction metaid="re231" id="re286" name="DhlmBCTAc(leu)" reversible="false">
<notes>
<html xmlns="http://www.w3.org/1999/xhtml">
<head>
<title/>
</head>
<body>LeuMetabolism
</body>
</html>
</notes>
<annotation>
<celldesigner:extension>
<celldesigner:name>DhlmBCTAc(leu)</celldesigner:name>
<celldesigner:reactionType>STATE_TRANSITION</celldesigner:reactionType>
<celldesigner:baseReactants>
<celldesigner:baseReactant species="s249" alias="sa581">
<celldesigner:linkAnchor position="S"/>
</celldesigner:baseReactant>
</celldesigner:baseReactants>
<celldesigner:baseProducts>
<celldesigner:baseProduct species="s250" alias="sa584"/>
</celldesigner:baseProducts>
<celldesigner:connectScheme connectPolicy="direct" rectangleIndex="0">
<celldesigner:listOfLineDirection>
<celldesigner:lineDirection index="0" value="unknown"/>
</celldesigner:listOfLineDirection>
</celldesigner:connectScheme>

<celldesigner:line width="1.0" color="ff000000"/>

</celldesigner:extension>

</annotation>

<listOfReactants>

<speciesReference metaid="CDMT00474" species="s249">

<annotation>

<celldesigner:extension>

<celldesigner:alias>sa581</celldesigner:alias>

</celldesigner:extension>

</annotation>

</speciesReference>

</listOfReactants>

<listOfProducts>

<speciesReference metaid="CDMT00475" species="s250">

<annotation>

<celldesigner:extension>

<celldesigner:alias>sa584</celldesigner:alias>

</celldesigner:extension>

</annotation>

</speciesReference>

</listOfProducts>

</reaction>

<reaction metaid="re233" id="re287" name="DhlmBCTAc(ile)" reversible="false">

<notes>

<html xmlns="http://www.w3.org/1999/xhtml">

<head>

<title/>

</head>

<body>IleMetabolism

</body>

</html>

</notes>

<annotation>

<celldesigner:extension>

<celldesigner:name>DhlmBCTAc(ile)</celldesigner:name>

<celldesigner:reactionType>STATE_TRANSITION</celldesigner:reactionType>

<celldesigner:baseReactants>

<celldesigner:baseReactant species="s389" alias="sa582"/>

</celldesigner:baseReactants>

<celldesigner:baseProducts>

<celldesigner:baseProduct species="s447" alias="sa585"/>

</celldesigner:baseProducts>

<celldesigner:connectScheme connectPolicy="direct" rectangleIndex="0">

<celldesigner:listOfLineDirection>

<celldesigner:lineDirection index="0" value="unknown"/>

</celldesigner:listOfLineDirection>

</celldesigner:connectScheme>

<celldesigner:line width="1.0" color="ff000000"/>

</celldesigner:extension>

</annotation>

<listOfReactants>

<speciesReference metaid="CDMT00476" species="s389">

<annotation>

<celldesigner:extension>

<celldesigner:alias>sa582</celldesigner:alias>

</celldesigner:extension>

</annotation>

</speciesReference>

</listOfReactants>

<listOfProducts>

<speciesReference metaid="CDMT00477" species="s447">

<annotation>

<celldesigner:extension>

<celldesigner:alias>sa585</celldesigner:alias>

</celldesigner:extension>

</annotation>

</speciesReference>

</listOfProducts>

</reaction>

<reaction metaid="re235" id="re288" name="AcylCoaDH">

<notes>

<html xmlns="http://www.w3.org/1999/xhtml">

<head>

<title/>

</head>

<body>IleMetabolism

</body>

</html>

</notes>

<annotation>

<celldesigner:extension>

<celldesigner:name>AcylCoaDH</celldesigner:name>

<celldesigner:reactionType>STATE_TRANSITION</celldesigner:reactionType>

<celldesigner:baseReactants>

<celldesigner:baseReactant species="s447" alias="sa585"/>

</celldesigner:baseReactants>

<celldesigner:baseProducts>

<celldesigner:baseProduct species="s400" alias="sa587"/>

</celldesigner:baseProducts>

<celldesigner:connectScheme connectPolicy="direct" rectangleIndex="0">

<celldesigner:listOfLineDirection>

<celldesigner:lineDirection index="0" value="unknown"/>

</celldesigner:listOfLineDirection>

</celldesigner:connectScheme>

<celldesigner:line width="1.0" color="ff000000"/>

</celldesigner:extension>

</annotation>

<listOfReactants>

<speciesReference metaid="CDMT00478" species="s447">

<annotation>

<celldesigner:extension>

<celldesigner:alias>sa585</celldesigner:alias>

</celldesigner:extension>

</annotation>

</speciesReference>

</listOfReactants>

<listOfProducts>

<speciesReference metaid="CDMT00479" species="s400">

<annotation>

<celldesigner:extension>

<celldesigner:alias>sa587</celldesigner:alias>

</celldesigner:extension>

</annotation>

</speciesReference>

</listOfProducts>

</reaction>

<reaction metaid="re237" id="re289" name="DhlmBCTAc(Val)" reversible="false">

<notes>

<html xmlns="http://www.w3.org/1999/xhtml">

<head>

<title/>

</head>

<body>ValMetabolism

</body>

</html>

</notes>

<annotation>

<celldesigner:extension>

<celldesigner:name>DhlmBCTAc(Val)</celldesigner:name>

<celldesigner:reactionType>STATE_TRANSITION</celldesigner:reactionType>

<celldesigner:baseReactants>

<celldesigner:baseReactant species="s246" alias="sa578"/>

</celldesigner:baseReactants>

<celldesigner:baseProducts>

<celldesigner:baseProduct species="s254" alias="sa583"/>

</celldesigner:baseProducts>
<celldesigner:connectScheme connectPolicy="direct" rectangleIndex="0">
<celldesigner:listOfLineDirection>
<celldesigner:lineDirection index="0" value="unknown"/>
</celldesigner:listOfLineDirection>
</celldesigner:connectScheme>
<celldesigner:line width="1.0" color="ff000000"/>
</celldesigner:extension>
</annotation>
<listOfReactants>
<speciesReference metaid="CDMT00480" species="s246">
<annotation>
<celldesigner:extension>
<celldesigner:alias>sa578</celldesigner:alias>
</celldesigner:extension>
</annotation>
</speciesReference>
</listOfReactants>
<listOfProducts>
<speciesReference metaid="CDMT00481" species="s254">
<annotation>
<celldesigner:extension>
<celldesigner:alias>sa583</celldesigner:alias>
</celldesigner:extension>
</annotation>
</speciesReference>
</listOfProducts>

</reaction>
<reaction metaid="re238" id="re290" name="DhlmBCTAc(val1)" reversible="false">
<notes>
<html xmlns="http://www.w3.org/1999/xhtml">
<head>
<title/>
</head>
<body>ValMetabolism
</body>
</html>
</notes>
<annotation>
<celldesigner:extension>
<celldesigner:name>DhlmBCTAc(val1)</celldesigner:name>
<celldesigner:reactionType>STATE_TRANSITION</celldesigner:reactionType>
<celldesigner:baseReactants>
<celldesigner:baseReactant species="s254" alias="sa583"/>
</celldesigner:baseReactants>
<celldesigner:baseProducts>
<celldesigner:baseProduct species="s255" alias="sa586"/>
</celldesigner:baseProducts>
<celldesigner:connectScheme connectPolicy="direct" rectangleIndex="0">
<celldesigner:listOfLineDirection>
<celldesigner:lineDirection index="0" value="unknown"/>
</celldesigner:listOfLineDirection>
</celldesigner:connectScheme>
<celldesigner:line width="1.0" color="ff000000"/>

</celldesigner:extension>

</annotation>

<listOfReactants>

<speciesReference metaid="CDMT00482" species="s254">

<annotation>

<celldesigner:extension>

<celldesigner:alias>sa583</celldesigner:alias>

</celldesigner:extension>

</annotation>

</speciesReference>

</listOfReactants>

<listOfProducts>

<speciesReference metaid="CDMT00483" species="s255">

<annotation>

<celldesigner:extension>

<celldesigner:alias>sa586</celldesigner:alias>

</celldesigner:extension>

</annotation>

</speciesReference>

</listOfProducts>

</reaction>

<reaction metaid="re291" id="re291" name="isovaleryl-coA dehydrogenase" reversible="false">

<notes>

<html xmlns="http://www.w3.org/1999/xhtml">

<head>

<title/>

</head>

<body>LeuMetabolism
</body>
</html>
</notes>
<annotation>
<celldesigner:extension>
<celldesigner:name>isovaleryl-coA dehydrogenase</celldesigner:name>
<celldesigner:reactionType>STATE_TRANSITION</celldesigner:reactionType>
<celldesigner:baseReactants>
<celldesigner:baseReactant species="s250" alias="sa584">
<celldesigner:linkAnchor position="S"/>
</celldesigner:baseReactant>
</celldesigner:baseReactants>
<celldesigner:baseProducts>
<celldesigner:baseProduct species="s392" alias="sa588"/>
</celldesigner:baseProducts>
<celldesigner:connectScheme connectPolicy="direct" rectangleIndex="0">
<celldesigner:listOfLineDirection>
<celldesigner:lineDirection index="0" value="unknown"/>
</celldesigner:listOfLineDirection>
</celldesigner:connectScheme>
<celldesigner:line width="1.0" color="ff000000"/>
</celldesigner:extension>
</annotation>
<listOfReactants>
<speciesReference metaid="CDMT00484" species="s250">
<annotation>

<celldesigner:extension>

<celldesigner:alias>sa584</celldesigner:alias>

</celldesigner:extension>

</annotation>

</speciesReference>

</listOfReactants>

<listOfProducts>

<speciesReference metaid="CDMT00485" species="s392">

<annotation>

<celldesigner:extension>

<celldesigner:alias>sa588</celldesigner:alias>

</celldesigner:extension>

</annotation>

</speciesReference>

</listOfProducts>

</reaction>

<reaction metaid="re432" id="re292" name="3-methylcrotonoyl-CoA carboxylase beta subunit" reversible="false">

<notes>

<html xmlns="http://www.w3.org/1999/xhtml">

<head>

<title/>

</head>

<body>LeuMetabolism

</body>

</html>

</notes>

<annotation>

<celldesigner:extension>

<celldesigner:name>3-methylcrotonoyl-CoA carboxylase beta subunit</celldesigner:name>

<celldesigner:reactionType>STATE_TRANSITION</celldesigner:reactionType>

<celldesigner:baseReactants>

<celldesigner:baseReactant species="s392" alias="sa588"/>

</celldesigner:baseReactants>

<celldesigner:baseProducts>

<celldesigner:baseProduct species="s456" alias="sa384">

<celldesigner:linkAnchor position="SE"/>

</celldesigner:baseProduct>

</celldesigner:baseProducts>

<celldesigner:connectScheme connectPolicy="direct" rectangleIndex="1">

<celldesigner:listOfLineDirection>

<celldesigner:lineDirection index="0" value="unknown"/>

<celldesigner:lineDirection index="1" value="unknown"/>

<celldesigner:lineDirection index="2" value="unknown"/>

</celldesigner:listOfLineDirection>

</celldesigner:connectScheme>

<celldesigner:editPoints>-0.005737707213613333,-0.012001360217609391 0.9029044828994452,-0.24285739810187695</celldesigner:editPoints>

<celldesigner:line width="1.0" color="ff000000"/>

</celldesigner:extension>

</annotation>

<listOfReactants>

<speciesReference metaid="CDMT00486" species="s392">

<annotation>

<celldesigner:extension>

<celldesigner:alias>sa588</celldesigner:alias>

</celldesigner:extension>

</annotation>

</speciesReference>

</listOfReactants>

<listOfProducts>

<speciesReference metaid="CDMT00487" species="s456">

<annotation>

<celldesigner:extension>

<celldesigner:alias>sa384</celldesigner:alias>

</celldesigner:extension>

</annotation>

</speciesReference>

</listOfProducts>

</reaction>

<reaction metaid="re435" id="re293" name="AcylCoaDH" reversible="false">

<notes>

<html xmlns="http://www.w3.org/1999/xhtml">

<head>

<title/>

</head>

<body>ValMetabolism

</body>

</html>

</notes>

<annotation>

<celldesigner:extension>

<celldesigner:name>AcylCoaDH</celldesigner:name>

<celldesigner:reactionType>STATE_TRANSITION</celldesigner:reactionType>
<celldesigner:baseReactants>
<celldesigner:baseReactant species="s255" alias="sa586"/>
</celldesigner:baseReactants>
<celldesigner:baseProducts>
<celldesigner:baseProduct species="s395" alias="sa589"/>
</celldesigner:baseProducts>
<celldesigner:connectScheme connectPolicy="direct" rectangleIndex="0">
<celldesigner:listOfLineDirection>
<celldesigner:lineDirection index="0" value="unknown"/>
</celldesigner:listOfLineDirection>
</celldesigner:connectScheme>
<celldesigner:line width="1.0" color="ff000000"/>
</celldesigner:extension>
</annotation>
<listOfReactants>
<speciesReference metaid="CDMT00488" species="s255">
<annotation>
<celldesigner:extension>
<celldesigner:alias>sa586</celldesigner:alias>
</celldesigner:extension>
</annotation>
</speciesReference>
</listOfReactants>
<listOfProducts>
<speciesReference metaid="CDMT00489" species="s395">
<annotation>

<celldesigner:extension>

<celldesigner:alias>sa589</celldesigner:alias>

</celldesigner:extension>

</annotation>

</speciesReference>

</listOfProducts>

</reaction>

<reaction metaid="re436" id="re294" name="EnoylCoAHydratase" reversible="false">

<notes>

<html xmlns="http://www.w3.org/1999/xhtml">

<head>

<title/>

</head>

<body>ValMetabolism

</body>

</html>

</notes>

<annotation>

<celldesigner:extension>

<celldesigner:name>EnoylCoAHydratase</celldesigner:name>

<celldesigner:reactionType>STATE_TRANSITION</celldesigner:reactionType>

<celldesigner:baseReactants>

<celldesigner:baseReactant species="s395" alias="sa589"/>

</celldesigner:baseReactants>

<celldesigner:baseProducts>

<celldesigner:baseProduct species="s396" alias="sa591"/>

</celldesigner:baseProducts>

<celldesigner:connectScheme connectPolicy="direct" rectangleIndex="0">

<celldesigner:listOfLineDirection>

<celldesigner:lineDirection index="0" value="unknown"/>

</celldesigner:listOfLineDirection>

</celldesigner:connectScheme>

<celldesigner:line width="1.0" color="ff000000"/>

</celldesigner:extension>

</annotation>

<listOfReactants>

<speciesReference metaid="CDMT00490" species="s395">

<annotation>

<celldesigner:extension>

<celldesigner:alias>sa589</celldesigner:alias>

</celldesigner:extension>

</annotation>

</speciesReference>

</listOfReactants>

<listOfProducts>

<speciesReference metaid="CDMT00491" species="s396">

<annotation>

<celldesigner:extension>

<celldesigner:alias>sa591</celldesigner:alias>

</celldesigner:extension>

</annotation>

</speciesReference>

</listOfProducts>

</reaction>

<reaction metaid="re437" id="re295" name="3HibCoAHydrolase" reversible="false">

<notes>

<html xmlns="http://www.w3.org/1999/xhtml">

<head>

<title/>

</head>

<body>ValMetabolism

</body>

</html>

</notes>

<annotation>

<celldesigner:extension>

<celldesigner:name>3HibCoAHydrolase</celldesigner:name>

<celldesigner:reactionType>STATE_TRANSITION</celldesigner:reactionType>

<celldesigner:baseReactants>

<celldesigner:baseReactant species="s396" alias="sa591"/>

</celldesigner:baseReactants>

<celldesigner:baseProducts>

<celldesigner:baseProduct species="s397" alias="sa593"/>

</celldesigner:baseProducts>

<celldesigner:connectScheme connectPolicy="direct" rectangleIndex="0">

<celldesigner:listOfLineDirection>

<celldesigner:lineDirection index="0" value="unknown"/>

</celldesigner:listOfLineDirection>

</celldesigner:connectScheme>

<celldesigner:line width="1.0" color="ff000000"/>

</celldesigner:extension>

</annotation>
<listOfReactants>
<speciesReference metaid="CDMT00492" species="s396">
<annotation>
<celldesigner:extension>
<celldesigner:alias>sa591</celldesigner:alias>
</celldesigner:extension>
</annotation>
</speciesReference>
</listOfReactants>
<listOfProducts>
<speciesReference metaid="CDMT00493" species="s397">
<annotation>
<celldesigner:extension>
<celldesigner:alias>sa593</celldesigner:alias>
</celldesigner:extension>
</annotation>
</speciesReference>
</listOfProducts>
</reaction>
<reaction metaid="re438" id="re296" name="3HACoADH" reversible="false">
<notes>
<html xmlns="http://www.w3.org/1999/xhtml">
<head>
<title/>
</head>
<body>ValMetabolism

</body>

</html>

</notes>

<annotation>

<celldesigner:extension>

<celldesigner:name>3HACoADH</celldesigner:name>

<celldesigner:reactionType>STATE_TRANSITION</celldesigner:reactionType>

<celldesigner:baseReactants>

<celldesigner:baseReactant species="s397" alias="sa593"/>

</celldesigner:baseReactants>

<celldesigner:baseProducts>

<celldesigner:baseProduct species="s398" alias="sa595"/>

</celldesigner:baseProducts>

<celldesigner:connectScheme connectPolicy="direct" rectangleIndex="0">

<celldesigner:listOfLineDirection>

<celldesigner:lineDirection index="0" value="unknown"/>

</celldesigner:listOfLineDirection>

</celldesigner:connectScheme>

<celldesigner:line width="1.0" color="ff000000"/>

</celldesigner:extension>

</annotation>

<listOfReactants>

<speciesReference metaid="CDMT00494" species="s397">

<annotation>

<celldesigner:extension>

<celldesigner:alias>sa593</celldesigner:alias>

</celldesigner:extension>

</annotation>

</speciesReference>

</listOfReactants>

<listOfProducts>

<speciesReference metaid="CDMT00495" species="s398">

<annotation>

<celldesigner:extension>

<celldesigner:alias>sa595</celldesigner:alias>

</celldesigner:extension>

</annotation>

</speciesReference>

</listOfProducts>

</reaction>

<reaction metaid="re439" id="re297" name="AldDH" reversible="false">

<notes>

<html xmlns="http://www.w3.org/1999/xhtml">

<head>

<title/>

</head>

<body>ValMetabolism

</body>

</html>

</notes>

<annotation>

<celldesigner:extension>

<celldesigner:name>AldDH</celldesigner:name>

<celldesigner:reactionType>STATE_TRANSITION</celldesigner:reactionType>

<celldesigner:baseReactants>

<celldesigner:baseReactant species="s398" alias="sa595">

<celldesigner:linkAnchor position="S"/>

</celldesigner:baseReactant>

</celldesigner:baseReactants>

<celldesigner:baseProducts>

<celldesigner:baseProduct species="s399" alias="sa596">

<celldesigner:linkAnchor position="N"/>

</celldesigner:baseProduct>

</celldesigner:baseProducts>

<celldesigner:connectScheme connectPolicy="direct" rectangleIndex="0">

<celldesigner:listOfLineDirection>

<celldesigner:lineDirection index="0" value="unknown"/>

</celldesigner:listOfLineDirection>

</celldesigner:connectScheme>

<celldesigner:line width="1.0" color="ff000000"/>

</celldesigner:extension>

</annotation>

<listOfReactants>

<speciesReference metaid="CDMT00496" species="s398">

<annotation>

<celldesigner:extension>

<celldesigner:alias>sa595</celldesigner:alias>

</celldesigner:extension>

</annotation>

</speciesReference>

</listOfReactants>

<listOfProducts>

<speciesReference metaid="CDMT00497" species="s399">

<annotation>

<celldesigner:extension>

<celldesigner:alias>sa596</celldesigner:alias>

</celldesigner:extension>

</annotation>

</speciesReference>

</listOfProducts>

</reaction>

<reaction metaid="re440" id="re298" name="MMalCoaLig" reversible="false">

<notes>

<html xmlns="http://www.w3.org/1999/xhtml">

<head>

<title/>

</head>

<body>ValMetabolism

</body>

</html>

</notes>

<annotation>

<celldesigner:extension>

<celldesigner:name>MMalCoaLig</celldesigner:name>

<celldesigner:reactionType>STATE_TRANSITION</celldesigner:reactionType>

<celldesigner:baseReactants>

<celldesigner:baseReactant species="s399" alias="sa596"/>

</celldesigner:baseReactants>

<celldesigner:baseProducts>

<celldesigner:baseProduct species="s1091" alias="sa599"/>

</celldesigner:baseProducts>

<celldesigner:connectScheme connectPolicy="direct" rectangleIndex="0">

<celldesigner:listOfLineDirection>

<celldesigner:lineDirection index="0" value="unknown"/>

</celldesigner:listOfLineDirection>

</celldesigner:connectScheme>

<celldesigner:line width="1.0" color="ff000000"/>

</celldesigner:extension>

</annotation>

<listOfReactants>

<speciesReference metaid="CDMT00498" species="s399">

<annotation>

<celldesigner:extension>

<celldesigner:alias>sa596</celldesigner:alias>

</celldesigner:extension>

</annotation>

</speciesReference>

</listOfReactants>

<listOfProducts>

<speciesReference metaid="CDMT00499" species="s1091">

<annotation>

<celldesigner:extension>

<celldesigner:alias>sa599</celldesigner:alias>

</celldesigner:extension>

</annotation>

</speciesReference>
</listOfProducts>
</reaction>
<reaction metaid="re442" id="re299" name="EnoylCoAHydratase" reversible="false">
<notes>
<html xmlns="http://www.w3.org/1999/xhtml">
<head>
<title/>
</head>
<body>IleMetabolism
</body>
</html>
</notes>
<annotation>
<celldesigner:extension>
<celldesigner:name>EnoylCoAHydratase</celldesigner:name>
<celldesigner:reactionType>STATE_TRANSITION</celldesigner:reactionType>
<celldesigner:baseReactants>
<celldesigner:baseReactant species="s400" alias="sa587"/>
</celldesigner:baseReactants>
<celldesigner:baseProducts>
<celldesigner:baseProduct species="s401" alias="sa592"/>
</celldesigner:baseProducts>
<celldesigner:connectScheme connectPolicy="direct" rectangleIndex="0">
<celldesigner:listOfLineDirection>
<celldesigner:lineDirection index="0" value="unknown"/>
</celldesigner:listOfLineDirection>

</celldesigner:connectScheme>

<celldesigner:line width="1.0" color="ff000000"/>

</celldesigner:extension>

</annotation>

<listOfReactants>

<speciesReference metaid="CDMT00500" species="s400">

<annotation>

<celldesigner:extension>

<celldesigner:alias>sa587</celldesigner:alias>

</celldesigner:extension>

</annotation>

</speciesReference>

</listOfReactants>

<listOfProducts>

<speciesReference metaid="CDMT00501" species="s401">

<annotation>

<celldesigner:extension>

<celldesigner:alias>sa592</celldesigner:alias>

</celldesigner:extension>

</annotation>

</speciesReference>

</listOfProducts>

</reaction>

<reaction metaid="re443" id="re300" name="3HACoADH" reversible="false">

<notes>

<html xmlns="http://www.w3.org/1999/xhtml">

<head>

<title/>

</head>

<body>IleMetabolism

</body>

</html>

</notes>

<annotation>

<celldesigner:extension>

<celldesigner:name>3HACoADH</celldesigner:name>

<celldesigner:reactionType>STATE_TRANSITION</celldesigner:reactionType>

<celldesigner:baseReactants>

<celldesigner:baseReactant species="s401" alias="sa592"/>

</celldesigner:baseReactants>

<celldesigner:baseProducts>

<celldesigner:baseProduct species="s402" alias="sa594"/>

</celldesigner:baseProducts>

<celldesigner:connectScheme connectPolicy="direct" rectangleIndex="0">

<celldesigner:listOfLineDirection>

<celldesigner:lineDirection index="0" value="unknown"/>

</celldesigner:listOfLineDirection>

</celldesigner:connectScheme>

<celldesigner:line width="1.0" color="ff000000"/>

</celldesigner:extension>

</annotation>

<listOfReactants>

<speciesReference metaid="CDMT00502" species="s401">

<annotation>

<celldesigner:extension>

<celldesigner:alias>sa592</celldesigner:alias>

</celldesigner:extension>

</annotation>

</speciesReference>

</listOfReactants>

<listOfProducts>

<speciesReference metaid="CDMT00503" species="s402">

<annotation>

<celldesigner:extension>

<celldesigner:alias>sa594</celldesigner:alias>

</celldesigner:extension>

</annotation>

</speciesReference>

</listOfProducts>

</reaction>

<reaction metaid="re444" id="re301" name="ACoaAcylTr" reversible="false">

<notes>

<html xmlns="http://www.w3.org/1999/xhtml">

<head>

<title/>

</head>

<body>IleMetabolism

</body>

</html>

</notes>

<annotation>

<celldesigner:extension>

<celldesigner:name>ACoaAcylTr</celldesigner:name>

<celldesigner:reactionType>STATE_TRANSITION</celldesigner:reactionType>

<celldesigner:baseReactants>

<celldesigner:baseReactant species="s402" alias="sa594"/>

</celldesigner:baseReactants>

<celldesigner:baseProducts>

<celldesigner:baseProduct species="s7" alias="sa597"/>

</celldesigner:baseProducts>

<celldesigner:listOfProductLinks>

<celldesigner:productLink product="s404" alias="sa598" targetLineIndex="-1,1">

<celldesigner:connectScheme connectPolicy="direct">

<celldesigner:listOfLineDirection>

<celldesigner:lineDirection index="0" value="unknown"/>

</celldesigner:listOfLineDirection>

</celldesigner:connectScheme>

<celldesigner:line width="1.0" color="ff000000" type="Straight"/>

</celldesigner:productLink>

</celldesigner:listOfProductLinks>

<celldesigner:connectScheme connectPolicy="direct" rectangleIndex="0">

<celldesigner:listOfLineDirection>

<celldesigner:lineDirection index="0" value="unknown"/>

</celldesigner:listOfLineDirection>

</celldesigner:connectScheme>

<celldesigner:line width="1.0" color="ff000000"/>

</celldesigner:extension>

</annotation>

<listOfReactants>

<speciesReference metaid="CDMT00504" species="s402">

<annotation>

<celldesigner:extension>

<celldesigner:alias>sa594</celldesigner:alias>

</celldesigner:extension>

</annotation>

</speciesReference>

</listOfReactants>

<listOfProducts>

<speciesReference metaid="CDMT00505" species="s7">

<annotation>

<celldesigner:extension>

<celldesigner:alias>sa597</celldesigner:alias>

</celldesigner:extension>

</annotation>

</speciesReference>

<speciesReference metaid="CDMT00506" species="s404">

<annotation>

<celldesigner:extension>

<celldesigner:alias>sa598</celldesigner:alias>

</celldesigner:extension>

</annotation>

</speciesReference>

</listOfProducts>

</reaction>

<reaction metaid="re302" id="re302" name="OoaAT" reversible="false">

<annotation>

<celldesigner:extension>

<celldesigner:name>OoaAT</celldesigner:name>

<celldesigner:reactionType>STATE_TRANSITION</celldesigner:reactionType>

<celldesigner:baseReactants>

<celldesigner:baseReactant species="s491" alias="sa574"/>

</celldesigner:baseReactants>

<celldesigner:baseProducts>

<celldesigner:baseProduct species="s259" alias="sa615"/>

</celldesigner:baseProducts>

<celldesigner:connectScheme connectPolicy="direct" rectangleIndex="0">

<celldesigner:listOfLineDirection>

<celldesigner:lineDirection index="0" value="unknown"/>

</celldesigner:listOfLineDirection>

</celldesigner:connectScheme>

<celldesigner:line width="1.0" color="ff000000"/>

</celldesigner:extension>

</annotation>

<listOfReactants>

<speciesReference metaid="CDMT00507" species="s491">

<annotation>

<celldesigner:extension>

<celldesigner:alias>sa574</celldesigner:alias>

</celldesigner:extension>

</annotation>

</speciesReference>

</listOfReactants>

<listOfProducts>

<speciesReference metaid="CDMT00508" species="s259">

<annotation>

<celldesigner:extension>

<celldesigner:alias>sa615</celldesigner:alias>

</celldesigner:extension>

</annotation>

</speciesReference>

</listOfProducts>

</reaction>

<reaction metaid="re304" id="re304" name="GlyDH" reversible="false">

<annotation>

<celldesigner:extension>

<celldesigner:name>GlyDH</celldesigner:name>

<celldesigner:reactionType>STATE_TRANSITION</celldesigner:reactionType>

<celldesigner:baseReactants>

<celldesigner:baseReactant species="s473" alias="sa536">

<celldesigner:linkAnchor position="E"/>

</celldesigner:baseReactant>

</celldesigner:baseReactants>

<celldesigner:baseProducts>

<celldesigner:baseProduct species="s240" alias="sa613">

<celldesigner:linkAnchor position="W"/>

</celldesigner:baseProduct>

</celldesigner:baseProducts>

<celldesigner:listOfReactantLinks>

<celldesigner:reactantLink reactant="s241" alias="sa611" targetLineIndex="-1,0">

<celldesigner:connectScheme connectPolicy="direct">

<celldesigner:listOfLineDirection>

<celldesigner:lineDirection index="0" value="unknown"/>

</celldesigner:listOfLineDirection>

</celldesigner:connectScheme>

<celldesigner:line width="1.0" color="ff000000" type="Straight"/>

</celldesigner:reactantLink>

</celldesigner:listOfReactantLinks>

<celldesigner:connectScheme connectPolicy="direct" rectangleIndex="0">

<celldesigner:listOfLineDirection>

<celldesigner:lineDirection index="0" value="unknown"/>

</celldesigner:listOfLineDirection>

</celldesigner:connectScheme>

<celldesigner:line width="1.0" color="ff000000"/>

</celldesigner:extension>

</annotation>

<listOfReactants>

<speciesReference metaid="CDMT00511" species="s473">

<annotation>

<celldesigner:extension>

<celldesigner:alias>sa536</celldesigner:alias>

</celldesigner:extension>

</annotation>

</speciesReference>

<speciesReference metaid="CDMT00512" species="s241">

<annotation>

<celldesigner:extension>

<celldesigner:alias>sa611</celldesigner:alias>

</celldesigner:extension>

</annotation>

</speciesReference>

</listOfReactants>

<listOfProducts>

<speciesReference metaid="CDMT00513" species="s240">

<annotation>

<celldesigner:extension>

<celldesigner:alias>sa613</celldesigner:alias>

</celldesigner:extension>

</annotation>

</speciesReference>

</listOfProducts>

</reaction>

<reaction metaid="re219" id="re305" name="AMT" reversible="false">

<annotation>

<celldesigner:extension>

<celldesigner:name>AMT</celldesigner:name>

<celldesigner:reactionType>STATE_TRANSITION</celldesigner:reactionType>

<celldesigner:baseReactants>

<celldesigner:baseReactant species="s240" alias="sa613">

<celldesigner:linkAnchor position="E"/>

</celldesigner:baseReactant>

</celldesigner:baseReactants>

<celldesigner:baseProducts>

<celldesigner:baseProduct species="s474" alias="sa614">

<celldesigner:linkAnchor position="WNW"/>
</celldesigner:baseProduct>
</celldesigner:baseProducts>
<celldesigner:listOfProductLinks>
<celldesigner:productLink product="s243" alias="sa612" targetLineIndex="-1,1">
<celldesigner:connectScheme connectPolicy="direct">
<celldesigner:listOfLineDirection>
<celldesigner:lineDirection index="0" value="unknown"/>
</celldesigner:listOfLineDirection>
</celldesigner:connectScheme>
<celldesigner:line width="1.0" color="ff000000" type="Straight"/>
</celldesigner:productLink>
</celldesigner:listOfProductLinks>
<celldesigner:connectScheme connectPolicy="direct" rectangleIndex="0">
<celldesigner:listOfLineDirection>
<celldesigner:lineDirection index="0" value="unknown"/>
</celldesigner:listOfLineDirection>
</celldesigner:connectScheme>
<celldesigner:line width="1.0" color="ff000000"/>
</celldesigner:extension>
</annotation>
<listOfReactants>
<speciesReference metaid="CDMT00514" species="s240">
<annotation>
<celldesigner:extension>
<celldesigner:alias>sa613</celldesigner:alias>
</celldesigner:extension>

</annotation>

</speciesReference>

</listOfReactants>

<listOfProducts>

<speciesReference metaid="CDMT00515" species="s474">

<annotation>

<celldesigner:extension>

<celldesigner:alias>sa614</celldesigner:alias>

</celldesigner:extension>

</annotation>

</speciesReference>

<speciesReference metaid="CDMT00516" species="s243">

<annotation>

<celldesigner:extension>

<celldesigner:alias>sa612</celldesigner:alias>

</celldesigner:extension>

</annotation>

</speciesReference>

</listOfProducts>

</reaction>

<reaction metaid="re220" id="re306" name="DHLMDH" reversible="false">

<annotation>

<celldesigner:extension>

<celldesigner:name>DHLMDH</celldesigner:name>

<celldesigner:reactionType>STATE_TRANSITION</celldesigner:reactionType>

<celldesigner:baseReactants>

<celldesigner:baseReactant species="s243" alias="sa612">

<celldesigner:linkAnchor position="W"/>

</celldesigner:baseReactant>

</celldesigner:baseReactants>

<celldesigner:baseProducts>

<celldesigner:baseProduct species="s241" alias="sa611">

<celldesigner:linkAnchor position="ESE"/>

</celldesigner:baseProduct>

</celldesigner:baseProducts>

<celldesigner:connectScheme connectPolicy="direct" rectangleIndex="0">

<celldesigner:listOfLineDirection>

<celldesigner:lineDirection index="0" value="unknown"/>

</celldesigner:listOfLineDirection>

</celldesigner:connectScheme>

<celldesigner:line width="1.0" color="ff000000"/>

</celldesigner:extension>

</annotation>

<listOfReactants>

<speciesReference metaid="CDMT00517" species="s243">

<annotation>

<celldesigner:extension>

<celldesigner:alias>sa612</celldesigner:alias>

</celldesigner:extension>

</annotation>

</speciesReference>

</listOfReactants>

<listOfProducts>

<speciesReference metaid="CDMT00518" species="s241">

<annotation>
<celldesigner:extension>
<celldesigner:alias>sa611</celldesigner:alias>
</celldesigner:extension>
</annotation>
</speciesReference>
</listOfProducts>
</reaction>
<reaction metaid="re212" id="re315" name="AdsS" reversible="false">
<annotation>
<celldesigner:extension>
<celldesigner:name>AdsS</celldesigner:name>
<celldesigner:reactionType>STATE_TRANSITION</celldesigner:reactionType>
<celldesigner:baseReactants>
<celldesigner:baseReactant species="s1004" alias="sa556">
<celldesigner:linkAnchor position="E"/>
</celldesigner:baseReactant>
</celldesigner:baseReactants>
<celldesigner:baseProducts>
<celldesigner:baseProduct species="s234" alias="sa616">
<celldesigner:linkAnchor position="W"/>
</celldesigner:baseProduct>
</celldesigner:baseProducts>
<celldesigner:connectScheme connectPolicy="direct" rectangleIndex="0">
<celldesigner:listOfLineDirection>
<celldesigner:lineDirection index="0" value="unknown"/>
</celldesigner:listOfLineDirection>

</celldesigner:connectScheme>

<celldesigner:line width="1.0" color="ff000000"/>

</celldesigner:extension>

</annotation>

<listOfReactants>

<speciesReference metaid="CDMT00539" species="s1004">

<annotation>

<celldesigner:extension>

<celldesigner:alias>sa556</celldesigner:alias>

</celldesigner:extension>

</annotation>

</speciesReference>

</listOfReactants>

<listOfProducts>

<speciesReference metaid="CDMT00540" species="s234">

<annotation>

<celldesigner:extension>

<celldesigner:alias>sa616</celldesigner:alias>

</celldesigner:extension>

</annotation>

</speciesReference>

</listOfProducts>

</reaction>

<reaction metaid="re213" id="re316" name="AdsLyase" reversible="false">

<annotation>

<celldesigner:extension>

<celldesigner:name>AdsLyase</celldesigner:name>

<celldesigner:reactionType>STATE_TRANSITION</celldesigner:reactionType>

<celldesigner:baseReactants>

<celldesigner:baseReactant species="s234" alias="sa616">

<celldesigner:linkAnchor position="E"/>

</celldesigner:baseReactant>

</celldesigner:baseReactants>

<celldesigner:baseProducts>

<celldesigner:baseProduct species="s28" alias="sa617">

<celldesigner:linkAnchor position="W"/>

</celldesigner:baseProduct>

</celldesigner:baseProducts>

<celldesigner:connectScheme connectPolicy="direct" rectangleIndex="0">

<celldesigner:listOfLineDirection>

<celldesigner:lineDirection index="0" value="unknown"/>

</celldesigner:listOfLineDirection>

</celldesigner:connectScheme>

<celldesigner:line width="1.0" color="ff000000"/>

</celldesigner:extension>

</annotation>

<listOfReactants>

<speciesReference metaid="CDMT00541" species="s234">

<annotation>

<celldesigner:extension>

<celldesigner:alias>sa616</celldesigner:alias>

</celldesigner:extension>

</annotation>

</speciesReference>

</listOfReactants>
<listOfProducts>
<speciesReference metaid="CDMT00542" species="s28">
<annotation>
<celldesigner:extension>
<celldesigner:alias>sa617</celldesigner:alias>
</celldesigner:extension>
</annotation>
</speciesReference>
</listOfProducts>
</reaction>
<reaction metaid="re586" id="re317" reversible="false">
<annotation>
<celldesigner:extension>
<celldesigner:reactionType>STATE_TRANSITION</celldesigner:reactionType>
<celldesigner:baseReactants>
<celldesigner:baseReactant species="s475" alias="sa537"/>
</celldesigner:baseReactants>
<celldesigner:baseProducts>
<celldesigner:baseProduct species="s2" alias="sa143">
<celldesigner:linkAnchor position="N"/>
</celldesigner:baseProduct>
</celldesigner:baseProducts>
<celldesigner:connectScheme connectPolicy="direct" rectangleIndex="2">
<celldesigner:listOfLineDirection>
<celldesigner:lineDirection index="0" value="unknown"/>
<celldesigner:lineDirection index="1" value="unknown"/>

<celldesigner:lineDirection index="2" value="unknown"/>

<celldesigner:lineDirection index="3" value="unknown"/>

</celldesigner:listOfLineDirection>

</celldesigner:connectScheme>

<celldesigner:editPoints>-0.0038979621109191953,0.012583237732576968 -
0.018771496613008143,0.18435918300059015
0.43935293178213775,0.17457631760256828</celldesigner:editPoints>

<celldesigner:line width="1.0" color="ff3333ff"/>

</celldesigner:extension>

</annotation>

<listOfReactants>

<speciesReference metaid="CDMT00543" species="s475">

<annotation>

<celldesigner:extension>

<celldesigner:alias>sa537</celldesigner:alias>

</celldesigner:extension>

</annotation>

</speciesReference>

</listOfReactants>

<listOfProducts>

<speciesReference metaid="CDMT00544" species="s2">

<annotation>

<celldesigner:extension>

<celldesigner:alias>sa143</celldesigner:alias>

</celldesigner:extension>

</annotation>

</speciesReference>

</listOfProducts>

</reaction>

<reaction metaid="re318" id="re318" reversible="false">

<annotation>

<celldesigner:extension>

<celldesigner:reactionType>STATE_TRANSITION</celldesigner:reactionType>

<celldesigner:baseReactants>

<celldesigner:baseReactant species="s475" alias="sa537">

<celldesigner:linkAnchor position="NE"/>

</celldesigner:baseReactant>

</celldesigner:baseReactants>

<celldesigner:baseProducts>

<celldesigner:baseProduct species="s244" alias="sa618"/>

</celldesigner:baseProducts>

<celldesigner:connectScheme connectPolicy="direct" rectangleIndex="0">

<celldesigner:listOfLineDirection>

<celldesigner:lineDirection index="0" value="unknown"/>

</celldesigner:listOfLineDirection>

</celldesigner:connectScheme>

<celldesigner:line width="1.0" color="ff000000"/>

</celldesigner:extension>

</annotation>

<listOfReactants>

<speciesReference metaid="CDMT00243" species="s475">

<annotation>

<celldesigner:extension>

<celldesigner:alias>sa537</celldesigner:alias>

</celldesigner:extension>

</annotation>

</speciesReference>

</listOfReactants>

<listOfProducts>

<speciesReference metaid="CDMT00244" species="s244">

<annotation>

<celldesigner:extension>

<celldesigner:alias>sa618</celldesigner:alias>

</celldesigner:extension>

</annotation>

</speciesReference>

</listOfProducts>

</reaction>

</listOfReactions>

</model>

</sbml>
