# Supplementary material for: Integrative Computational Framework for Understanding Metabolic Modulation in Leishmania
Source: Front Bioeng Biotechnol. 2019 Nov 19;7:336. doi: 10.3389/fbioe.2019.00336 (PMC6877600; doi:10.3389/fbioe.2019.00336)
Supplement: Figure S1 — Depicting the internal connectivity in small scale models. The components are connected by backbone forming high curvature nodes (encircled in red color). Low curvature nodes are shown without encircled nodes. High curvature nodes act as bridges between major network communities and play important role in determining structural and functional organization of network. [file Image_1.pdf]

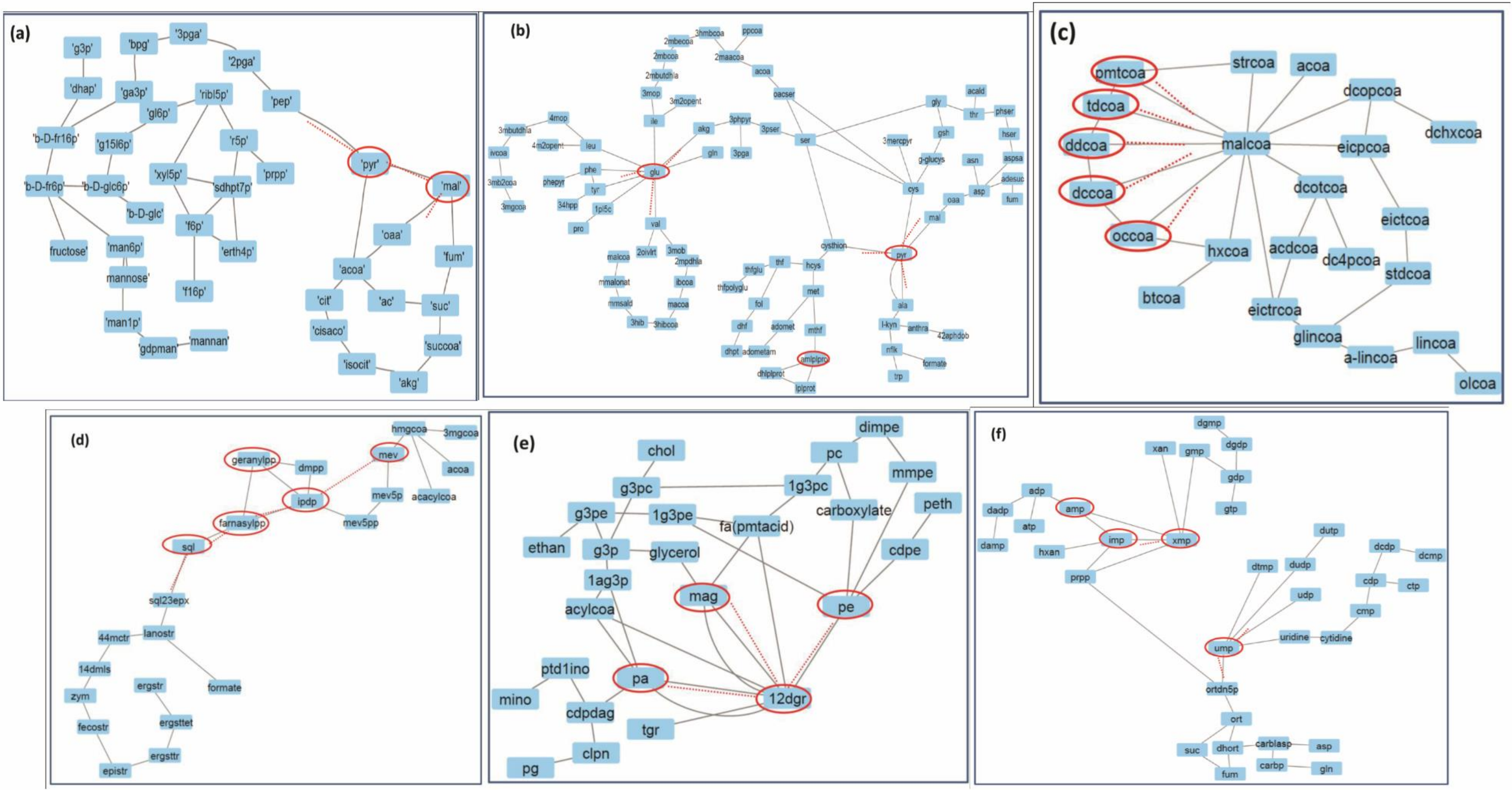

(a)
'g3p'
'bpg'
'3pga'
'2pga'
'dhap'
'ga3p'
'ribl5p'
'pep'
'gl6p'
'b-D-fr16p'
'g15l6p'
'r5p'
'xyl5p'
'prpp'
'pyr'
'b-D-fr6p'
'b-D-glc6p'
'sdhpt7p'
'mal'
'b-D-glc'
'f6p'
'oaa'
fructose'
'man6p'
'erth4p'
'fum'
mannose'
'acoa'
'f16p'
'cit'
'ac'
'suc'
'man1p'
'cisaco'
'gdpman'
'mannan'
'succoa'
'isocit'
'akg'
(b)
2mbecoa
3hmbcoa
ppcoa
2mbcoa
2maacoa
2mbutdhla
acoa
3mop
oacser
3m2opent
acald
ile
gly
4mop
akg
3phpyr
thr
phser
3mbutdhla
3pser
gsh
hser
leu
ser
ivcoa
4m2opent
gln
3mercpyr
asn
aspsa
phe
glu
3pga
g-glucys
phepyr
asp
adesuc
3mb2coa
tyr
cys
34hpp
1pl5c
oaa
fum
3mgcoa
val
mal
pro
2oivlrt
3mob
cysthion
malcoa
2mpdhla
thf
hcys
pyr
thfglu
mmalonat
ibcoa
thfpolyglu
met
ala
mmsald
fol
macoa
l-kyn
anthra
3hib
3hibcoa
dhf
adomet
mthf
42aphdob
dhpt
adometam
nflk
amlplpro
formate
dhlplprot
trp
lplprot
(c)
pmtcoa
strcoa
acoa
dcopcoa
tdcoa
dchxcoa
ddcoa
malcoa
eicpcoa
dccoa
dcotcoa
eictcoa
occoa
hxcoa
acdcoa
dc4pcoa
stdcoa
btcoa
eictrcoa
glincoa
lincoa
a-lincoa
olcoa
(d)
hmgcoa
3mgcoa
geranylpp
mev
dmpp
acoa
ipdp
mev5p
acacylcoa
farnasylpp
mev5pp
sql
sql23epx
lanostr
44mctr
14dmls
ergstr
formate
zym
ergsttet
fecostr
ergsttr
epistr
(e)
dimpe
chol
pc
mmpe
g3pc
1g3pc
peth
g3pe
1g3pe
carboxylate
fa(pmtacid)
ethan
g3p
glycerol
cdpe
1ag3p
mag
pe
acylcoa
ptd1ino
pa
12dgr
mino
cdpdag
tgr
clpn
pg
(f)
dgmp
xan
gmp
dgdp
gdp
adp
dadp
amp
atp
gtp
imp
xmp
dutp
damp
hxan
dcdp
dcmp
prpp
dtmp
dudp
cdp
ctp
udp
cmp
uridine
cytidine
ump
ortdn5p
ort
carblasp
asp
suc
dhort
carbp
gln
fum
