# Supplementary material for: Integrative Computational Framework for Understanding Metabolic Modulation in Leishmania
Source: Front Bioeng Biotechnol. 2019 Nov 19;7:336. doi: 10.3389/fbioe.2019.00336 (PMC6877600; doi:10.3389/fbioe.2019.00336)
Supplement: Supplementary file 9 [file Data_Sheet_1.PDF]

$$\frac{d([\text{Met}]\cdot V_{\text{cell}})}{dt} = -V_{\text{cell}}\cdot\left(\frac{\text{3.4e-009}\cdot[\text{Met}]^{2.3}}{0.00025^{2.3}+[\text{Met}]^{2.3}}\right)$$

$$\begin{aligned}\frac{d([\text{SAM}]\cdot V_{\text{cell}})}{dt} &= +V_{\text{cell}}\cdot\left(\frac{\text{3.4e-009}\cdot[\text{Met}]^{2.3}}{0.00025^{2.3}+[\text{Met}]^{2.3}}\right)\\ &\quad -V_{\text{cell}}\cdot\left(\frac{\text{5.2e-009}\cdot[\text{SAM}]}{0.00038+[\text{SAM}]}\right)\end{aligned}$$

$$\begin{aligned}\frac{d([\text{dcSAM}]\cdot V_{\text{cell}})}{dt} &= +V_{\text{cell}}\cdot\left(\frac{\text{5.2e-009}\cdot[\text{SAM}]}{0.00038+[\text{SAM}]}\right)\\ &\quad -V_{\text{cell}}\cdot\left(\frac{\text{1.98e-013}\cdot[\text{Put}]\cdot[\text{dcSAM}]}{\text{9e-008}\cdot[\text{Put}]+0.000205\cdot[\text{dcSAM}]+[\text{Put}]\cdot[\text{dcSAM}]+0.1\cdot 0.000205}\right)\end{aligned}$$

$$\begin{aligned}\frac{d([\text{Arg}]\cdot V_{\text{cell}})}{dt} &= -V_{\text{cell}}\cdot((0.0085\cdot[\text{Arg}]\cdot[\text{MGO}]-\text{4.4e-006}\cdot[\text{"Arg-MG"}]\cdot[\text{"MGO.-"}]))\\ &\quad -V_{\text{cell}}\cdot\left(\frac{0.0024\cdot[\text{Arg}]}{0.0215+[\text{Arg}]}\right)\\ &\quad -V_{\text{cell}}\cdot\left(\frac{\text{3.5e-006}\cdot[\text{Arg}]\cdot[\text{NADPH}]}{\text{7e-007}\cdot[\text{Arg}]+\text{4.9e-006}\cdot[\text{NADPH}]+[\text{Arg}]\cdot[\text{NADPH}]+0.1\cdot\text{7e-007}}\right)\end{aligned}$$

$$\begin{aligned}\frac{d([\text{Orn}]\cdot V_{\text{cell}})}{dt} &= +V_{\text{cell}}\cdot\left(\frac{0.0024\cdot[\text{Arg}]}{0.0215+[\text{Arg}]}\right)\\ &\quad -V_{\text{cell}}\cdot\left(\frac{0.025256\cdot[\text{Orn}]}{0.00042+[\text{Orn}]}\right)\end{aligned}$$

$$\begin{aligned}\frac{d([\text{Put}]\cdot V_{\text{cell}})}{dt} &= +V_{\text{cell}}\cdot\left(\frac{0.025256\cdot[\text{Orn}]}{0.00042+[\text{Orn}]}\right)\\ &\quad -V_{\text{cell}}\cdot\left(\frac{\text{1.98e-013}\cdot[\text{Put}]\cdot[\text{dcSAM}]}{\text{9e-008}\cdot[\text{Put}]+0.000205\cdot[\text{dcSAM}]+[\text{Put}]\cdot[\text{dcSAM}]+0.1\cdot 0.000205}\right)\end{aligned}$$

$$\begin{aligned}\frac{d([\text{Spd}]\cdot V_{\text{cell}})}{dt} &= +V_{\text{cell}}\cdot\left(\frac{\text{1.98e-013}\cdot[\text{Put}]\cdot[\text{dcSAM}]}{\text{9e-008}\cdot[\text{Put}]+0.000205\cdot[\text{dcSAM}]+[\text{Put}]\cdot[\text{dcSAM}]+0.1\cdot 0.000205}\right)\\ &\quad -V_{\text{cell}}\cdot\left(\frac{\text{8e-007}\cdot[\text{Spd}]\cdot[\text{GSH}]}{0.00094\cdot\text{8.9e-005}+[\text{Spd}]\cdot\text{8.9e-005}+[\text{GSH}]\cdot 0.00094+[\text{Spd}]\cdot[\text{GSH}]}\right)\end{aligned}$$

$$\frac{d([\text{Cys}]\cdot V_{\text{cell}})}{dt} = -V_{\text{cell}}\cdot\left(\frac{\text{1.8e-006}\cdot[\text{Cys}]\cdot[\text{Glu}]}{0.0017\cdot[\text{Cys}]+0.0092\cdot[\text{Glu}]+[\text{Cys}]\cdot[\text{Glu}]+\text{9.37e-005}\cdot 0.0017}\right)$$

$$\frac{d([\text{Glu}]\cdot V_{\text{cell}})}{dt} = -V_{\text{cell}}\cdot\left(\frac{\text{1.8e-006}\cdot[\text{Cys}]\cdot[\text{Glu}]}{0.0017\cdot[\text{Cys}]+0.0092\cdot[\text{Glu}]+[\text{Cys}]\cdot[\text{Glu}]+\text{9.37e-005}\cdot 0.0017}\right)$$

$$\begin{aligned}\frac{d([\text{GluCys}]\cdot V_{\text{cell}})}{dt} &= +V_{\text{cell}}\cdot\left(\frac{\text{1.8e-006}\cdot[\text{Cys}]\cdot[\text{Glu}]}{0.0017\cdot[\text{Cys}]+0.0092\cdot[\text{Glu}]+[\text{Cys}]\cdot[\text{Glu}]+\text{9.37e-005}\cdot 0.0017}\right)\\ &\quad -V_{\text{cell}}\cdot\left(\frac{\text{3.4e-008}\cdot[\text{Gly}]\cdot[\text{GluCys}]}{0.0012\cdot[\text{Gly}]+\text{4e-005}\cdot[\text{GluCys}]+[\text{Gly}]\cdot[\text{GluCys}]+\text{8e-005}\cdot 0.0012}\right)\end{aligned}$$

$$\begin{aligned}\frac{d([\text{GSH}]\cdot V_{\text{cell}})}{dt} &= +V_{\text{cell}}\cdot\left(\frac{\text{3.4e-008}\cdot[\text{Gly}]\cdot[\text{GluCys}]}{0.0012\cdot[\text{Gly}]+\text{4e-005}\cdot[\text{GluCys}]+[\text{Gly}]\cdot[\text{GluCys}]+\text{8e-005}\cdot 0.0012}\right)\\ &\quad -V_{\text{cell}}\cdot\left(\frac{\text{8e-007}\cdot[\text{Spd}]\cdot[\text{GSH}]}{0.00094\cdot\text{8.9e-005}+[\text{Spd}]\cdot\text{8.9e-005}+[\text{GSH}]\cdot 0.00094+[\text{Spd}]\cdot[\text{GSH}]}\right)\\ &\quad -V_{\text{cell}}\cdot\left(\frac{\text{8e-007}\cdot[\text{Gspd}]\cdot[\text{GSH}]}{\text{4e-005}\cdot\text{8.9e-005}+[\text{Gspd}]\cdot\text{8.9e-005}+[\text{GSH}]\cdot\text{4e-005}+[\text{Gspd}]\cdot[\text{GSH}]}\right)\end{aligned}$$

$$\frac{d([\text{Gly}]\cdot V_{\text{cell}})}{dt} = -V_{\text{cell}}\cdot\left(\frac{\text{3.4e-008}\cdot[\text{Gly}]\cdot[\text{GluCys}]}{0.0012\cdot[\text{Gly}]+\text{4e-005}\cdot[\text{GluCys}]+[\text{Gly}]\cdot[\text{GluCys}]+\text{8e-005}\cdot 0.0012}\right)$$

$$\begin{aligned}\frac{d([\text{Gspd}]\cdot V_{\text{cell}})}{dt} &= +V_{\text{cell}}\cdot\left(\frac{\text{8e-007}\cdot[\text{Spd}]\cdot[\text{GSH}]}{0.00094\cdot\text{8.9e-005}+[\text{Spd}]\cdot\text{8.9e-005}+[\text{GSH}]\cdot 0.00094+[\text{Spd}]\cdot[\text{GSH}]}\right)\\ &\quad -V_{\text{cell}}\cdot\left(\frac{\text{8e-007}\cdot[\text{Gspd}]\cdot[\text{GSH}]}{\text{4e-005}\cdot\text{8.9e-005}+[\text{Gspd}]\cdot\text{8.9e-005}+[\text{GSH}]\cdot\text{4e-005}+[\text{Gspd}]\cdot[\text{GSH}]}\right)\end{aligned}$$

$$\begin{aligned}\frac{d([\text{MGO}]\cdot V_{\text{cell}})}{dt} &= +V_{\text{cell}}\cdot(0.016\cdot[\text{HTA}])\\ &\quad -V_{\text{cell}}\cdot((0.0085\cdot[\text{Arg}]\cdot[\text{MGO}]-\text{4.4e-006}\cdot[\text{"Arg-MG"}]\cdot[\text{"MGO.-"}]))\\ &\quad -V_{\text{cell}}\cdot((0.0068\cdot[\text{Lys}]\cdot[\text{MGO}]-0.0005\cdot[\text{"Lys-MG"}]\cdot[\text{"MGO.-"}]))\\ &\quad +V_{\text{cell}}\cdot(\text{1e+006}\cdot[\text{LO2.}]\cdot[\text{LO2.}])\\ &\quad +V_{\text{cell}}\cdot(\text{1.31e-010}\cdot[\text{"MGO.-"}]\cdot[\text{O2}])\\ &\quad +V_{\text{cell}}\cdot(0.00011\cdot[\text{DHAP}])\\ &\quad +V_{\text{cell}}\cdot(\text{1.1e-005}\cdot[\text{GA3P}])\\ &\quad -V_{\text{cell}}\cdot(0.0056\cdot[\text{MGO}]\cdot[\text{T[SH]2}])\end{aligned}$$

$$\begin{aligned}\frac{d([\text{HTA}]\cdot V_{\text{cell}})}{dt} &= -V_{\text{cell}}\cdot(0.016\cdot[\text{HTA}])\\ &\quad -V_{\text{cell}}\cdot\left(\frac{0.000159\cdot[\text{HTA}]}{\text{3.2e-005}+[\text{HTA}]+\frac{\text{3.2e-005}\cdot[\text{BBGS}]}{\text{5.36e-007}}}\right)\\ &\quad +V_{\text{cell}}\cdot(0.0056\cdot[\text{MGO}]\cdot[\text{T[SH]2}])\end{aligned}$$

$$\frac{d([\text{DL}]\cdot V_{\text{cell}})}{dt} = +V_{\text{cell}}\cdot\left(\frac{\text{2.3e-009}\cdot[\text{SDLTSH}]}{\text{3.9e-005}+[\text{SDLTSH}]}\right)$$

$$\begin{aligned}\frac{d([\text{NADP}]\cdot V_{\text{cell}})}{dt} &= +V_{\text{cell}}\cdot\left(\frac{\text{3.33e-006}\cdot[\text{TS2}]\cdot[\text{NADPH}]}{\text{2e-005}\cdot[\text{TS2}]+\text{5e-005}\cdot[\text{NADPH}]+[\text{TS2}]\cdot[\text{NADPH}]+0.1\cdot\text{2e-005}}\right)\\ &\quad +V_{\text{cell}}\cdot\left(\frac{\text{3.5e-006}\cdot[\text{Arg}]\cdot[\text{NADPH}]}{\text{7e-007}\cdot[\text{Arg}]+\text{4.9e-006}\cdot[\text{NADPH}]+[\text{Arg}]\cdot[\text{NADPH}]+0.1\cdot\text{7e-007}}\right)\end{aligned}$$

$$\begin{aligned}\frac{d([\text{NADPH}]\cdot V_{\text{cell}})}{dt} &= -V_{\text{cell}}\cdot\left(\frac{\text{3.33e-006}\cdot[\text{TS2}]\cdot[\text{NADPH}]}{\text{2e-005}\cdot[\text{TS2}]+\text{5e-005}\cdot[\text{NADPH}]+[\text{TS2}]\cdot[\text{NADPH}]+0.1\cdot\text{2e-005}}\right)\\ &\quad -V_{\text{cell}}\cdot\left(\frac{\text{3.5e-006}\cdot[\text{Arg}]\cdot[\text{NADPH}]}{\text{7e-007}\cdot[\text{Arg}]+\text{4.9e-006}\cdot[\text{NADPH}]+[\text{Arg}]\cdot[\text{NADPH}]+0.1\cdot\text{7e-007}}\right)\end{aligned}$$

$$\begin{aligned}\frac{d([\text{TS2}]\cdot V_{\text{cell}})}{dt} &= -V_{\text{cell}}\cdot\left(\frac{\text{3.33e-006}\cdot[\text{TS2}]\cdot[\text{NADPH}]}{\text{2e-005}\cdot[\text{TS2}]+\text{5e-005}\cdot[\text{NADPH}]+[\text{TS2}]\cdot[\text{NADPH}]+0.1\cdot\text{2e-005}}\right)\\ &\quad +V_{\text{cell}}\cdot(200\cdot[\text{T[SH]2}]\cdot[\text{TXNo}])\\ &\quad +V_{\text{cell}}\cdot(7200\cdot[\text{T[SH]2}]\cdot[\text{"ONOO-"}])\end{aligned}$$

$$\begin{aligned}\frac{d([\text{T[SH]2}]\cdot V_{\text{cell}})}{dt} &= +V_{\text{cell}}\cdot\left(\frac{\text{3.33e-006}\cdot[\text{TS2}]\cdot[\text{NADPH}]}{\text{2e-005}\cdot[\text{TS2}]+\text{5e-005}\cdot[\text{NADPH}]+[\text{TS2}]\cdot[\text{NADPH}]+0.1\cdot\text{2e-005}}\right)\\ &\quad +V_{\text{cell}}\cdot(0.016\cdot[\text{HTA}])\\ &\quad +V_{\text{cell}}\cdot\left(\frac{\text{2.3e-009}\cdot[\text{SDLTSH}]}{\text{3.9e-005}+[\text{SDLTSH}]}\right)\\ &\quad -V_{\text{cell}}\cdot(200\cdot[\text{T[SH]2}]\cdot[\text{TXNo}])\\ &\quad -V_{\text{cell}}\cdot(7200\cdot[\text{T[SH]2}]\cdot[\text{"ONOO-"}])\\ &\quad +V_{\text{cell}}\cdot\left(\frac{\text{8e-007}\cdot[\text{Gspd}]\cdot[\text{GSH}]}{\text{4e-005}\cdot\text{8.9e-005}+[\text{Gspd}]\cdot\text{8.9e-005}+[\text{GSH}]\cdot\text{4e-005}+[\text{Gspd}]\cdot[\text{GSH}]}\right)\\ &\quad -V_{\text{cell}}\cdot(0.0056\cdot[\text{MGO}]\cdot[\text{T[SH]2}])\end{aligned}$$

$$\frac{d([\text{CO2}]\cdot V_{\text{cell}})}{dt} = +V_{\text{cell}}\cdot\left(\frac{0.025256\cdot[\text{Orn}]}{0.00042+[\text{Orn}]}\right)$$

$$\begin{aligned}\frac{d([\text{SDLTSH}]\cdot V_{\text{cell}})}{dt} &= +V_{\text{cell}}\cdot\left(\frac{0.000159\cdot[\text{HTA}]}{\text{3.2e-005}+[\text{HTA}]+\frac{\text{3.2e-005}\cdot[\text{BBGS}]}{\text{5.36e-007}}}\right)\\ &\quad -V_{\text{cell}}\cdot\left(\frac{\text{2.3e-009}\cdot[\text{SDLTSH}]}{\text{3.9e-005}+[\text{SDLTSH}]}\right)\end{aligned}$$

$$\frac{d([\text{"Arg-MG"}]\cdot V_{\text{cell}})}{dt} = +V_{\text{cell}}\cdot((0.0085\cdot[\text{Arg}]\cdot[\text{MGO}]-\text{4.4e-006}\cdot[\text{"Arg-MG"}]\cdot[\text{"MGO.-"}]))$$

$$\frac{d([\text{Lys}]\cdot V_{\text{cell}})}{dt} = -V_{\text{cell}}\cdot((0.0068\cdot[\text{Lys}]\cdot[\text{MGO}]-0.0005\cdot[\text{"Lys-MG"}]\cdot[\text{"MGO.-"}]))$$

$$\frac{d([\text{"Lys-MG"}]\cdot V_{\text{cell}})}{dt} = +V_{\text{cell}}\cdot((0.0068\cdot[\text{Lys}]\cdot[\text{MGO}]-0.0005\cdot[\text{"Lys-MG"}]\cdot[\text{"MGO.-"}]))$$

$$\begin{aligned}\frac{d([\text{TXNo}]\cdot V_{\text{cell}})}{dt} &= -V_{\text{cell}}\cdot(200\cdot[\text{T[SH]2}]\cdot[\text{TXNo}])\\ &\quad +V_{\text{cell}}\cdot\left(\frac{15.4\cdot[\text{TDPx}]\cdot[\text{H2O2}]\cdot[\text{TXNr}]}{\text{2.2e-006}\cdot[\text{H2O2}]+0.000193\cdot[\text{TXNr}]+[\text{H2O2}]\cdot[\text{TXNr}]}\right)\\ &\quad +V_{\text{cell}}\cdot\left(\frac{8.8\cdot[\text{TryP}]\cdot[\text{H2O2}]\cdot[\text{TXNr}]}{\text{4.9e-006}\cdot[\text{H2O2}]+\text{6.3e-006}\cdot[\text{TXNr}]+[\text{H2O2}]\cdot[\text{TXNr}]}\right)\\ &\quad +V_{\text{cell}}\cdot(220000\cdot[\text{TXNr}]\cdot[\text{TDPxo}])\\ &\quad +V_{\text{cell}}\cdot(3500\cdot[\text{TXNr}]\cdot[\text{"ONOO-"}])\end{aligned}$$

$$\begin{aligned}\frac{d([\text{TXNr}]\cdot V_{\text{cell}})}{dt} &= +V_{\text{cell}}\cdot(200\cdot[\text{T[SH]2}]\cdot[\text{TXNo}])\\ &\quad -V_{\text{cell}}\cdot\left(\frac{15.4\cdot[\text{TDPx}]\cdot[\text{H2O2}]\cdot[\text{TXNr}]}{\text{2.2e-006}\cdot[\text{H2O2}]+0.000193\cdot[\text{TXNr}]+[\text{H2O2}]\cdot[\text{TXNr}]}\right)\\ &\quad -V_{\text{cell}}\cdot\left(\frac{8.8\cdot[\text{TryP}]\cdot[\text{H2O2}]\cdot[\text{TXNr}]}{\text{4.9e-006}\cdot[\text{H2O2}]+\text{6.3e-006}\cdot[\text{TXNr}]+[\text{H2O2}]\cdot[\text{TXNr}]}\right)\\ &\quad -V_{\text{cell}}\cdot(220000\cdot[\text{TXNr}]\cdot[\text{TDPxo}])\\ &\quad -V_{\text{cell}}\cdot(3500\cdot[\text{TXNr}]\cdot[\text{"ONOO-"}])\end{aligned}$$

$$\begin{aligned}\frac{d([\text{H2O}]\cdot V_{\text{cell}})}{dt} &= +V_{\text{cell}}\cdot\left(\frac{15.4\cdot[\text{TDPx}]\cdot[\text{H2O2}]\cdot[\text{TXNr}]}{\text{2.2e-006}\cdot[\text{H2O2}]+0.000193\cdot[\text{TXNr}]+[\text{H2O2}]\cdot[\text{TXNr}]}\right)\\ &\quad +V_{\text{cell}}\cdot\left(\frac{8.8\cdot[\text{TryP}]\cdot[\text{H2O2}]\cdot[\text{TXNr}]}{\text{4.9e-006}\cdot[\text{H2O2}]+\text{6.3e-006}\cdot[\text{TXNr}]+[\text{H2O2}]\cdot[\text{TXNr}]}\right)\\ &\quad +V_{\text{cell}}\cdot(\text{1e+010}\cdot[\text{.OH}]\cdot[\text{LH}])\\ &\quad +V_{\text{cell}}\cdot(100000\cdot[\text{ONOOH}]\cdot[\text{H2O2}])\end{aligned}$$

$$\begin{aligned}\frac{d([\text{H2O2}]\cdot V_{\text{cell}})}{dt} &= -V_{\text{cell}}\cdot\left(\frac{15.4\cdot[\text{TDPx}]\cdot[\text{H2O2}]\cdot[\text{TXNr}]}{\text{2.2e-006}\cdot[\text{H2O2}]+0.000193\cdot[\text{TXNr}]+[\text{H2O2}]\cdot[\text{TXNr}]}\right)\\ &\quad -V_{\text{cell}}\cdot\left(\frac{8.8\cdot[\text{TryP}]\cdot[\text{H2O2}]\cdot[\text{TXNr}]}{\text{4.9e-006}\cdot[\text{H2O2}]+\text{6.3e-006}\cdot[\text{TXNr}]+[\text{H2O2}]\cdot[\text{TXNr}]}\right)\\ &\quad -V_{\text{cell}}\cdot(76\cdot[\text{H2O2}]\cdot[\text{Fe2}])\\ &\quad +V_{\text{cell}}\cdot(500000\cdot[\text{"O2.-"}]\cdot[\text{"O2.-"}]\cdot[\text{"H+"}])\\ &\quad +V_{\text{cell}}\cdot(\text{1.6e+009}\cdot[\text{"O2.-"}]\cdot[\text{"O2.-"}])\\ &\quad -V_{\text{cell}}\cdot(100000\cdot[\text{ONOOH}]\cdot[\text{H2O2}])\end{aligned}$$

$$\begin{aligned}\frac{d([\text{.OH}]\cdot V_{\text{cell}})}{dt} &= +V_{\text{cell}}\cdot(76\cdot[\text{H2O2}]\cdot[\text{Fe2}])\\ &\quad -V_{\text{cell}}\cdot(\text{1e+010}\cdot[\text{.OH}]\cdot[\text{LH}])\\ &\quad -V_{\text{cell}}\cdot(\text{1.4e+010}\cdot[\text{NO2.}]\cdot[\text{.OH}])\end{aligned}$$

$$\begin{aligned}\frac{d([\text{Fe2}]\cdot V_{\text{cell}})}{dt} &= -V_{\text{cell}}\cdot(76\cdot[\text{H2O2}]\cdot[\text{Fe2}])\\ &\quad -V_{\text{cell}}\cdot(10000\cdot[\text{LOOH}]\cdot[\text{Fe2}])\end{aligned}$$

$$\begin{aligned}\frac{d([\text{Fe3}]\cdot V_{\text{cell}})}{dt} &= +V_{\text{cell}}\cdot(76\cdot[\text{H2O2}]\cdot[\text{Fe2}])\\ &\quad +V_{\text{cell}}\cdot(10000\cdot[\text{LOOH}]\cdot[\text{Fe2}])\end{aligned}$$

$$\begin{aligned}\frac{d([\text{"O2.-"}]\cdot V_{\text{cell}})}{dt} &= -2\cdot V_{\text{cell}}\cdot(500000\cdot[\text{"O2.-"}]\cdot[\text{"O2.-"}]\cdot[\text{"H+"}])\\ &\quad -2\cdot V_{\text{cell}}\cdot(\text{1.6e+009}\cdot[\text{"O2.-"}]\cdot[\text{"O2.-"}])\\ &\quad +V_{\text{cell}}\cdot(100000\cdot[\text{ONOOH}]\cdot[\text{H2O2}])\\ &\quad -V_{\text{cell}}\cdot(\text{3.4e+007}\cdot[\text{NO2.}]\cdot[\text{"O2.-"}])\\ &\quad +V_{\text{cell}}\cdot(\text{1.31e-010}\cdot[\text{"MGO.-"}]\cdot[\text{O2}])\end{aligned}$$

$$\begin{aligned}\frac{d([\text{O2}]\cdot V_{\text{cell}})}{dt} &= +V_{\text{cell}}\cdot(500000\cdot[\text{"O2.-"}]\cdot[\text{"O2.-"}]\cdot[\text{"H+"}])\\ &\quad +V_{\text{cell}}\cdot(\text{1.6e+009}\cdot[\text{"O2.-"}]\cdot[\text{"O2.-"}])\\ &\quad -V_{\text{cell}}\cdot(\text{3e+008}\cdot[\text{L.}]\cdot[\text{O2}])\\ &\quad -V_{\text{cell}}\cdot(\text{1.31e-010}\cdot[\text{"MGO.-"}]\cdot[\text{O2}])\end{aligned}$$

$$\begin{aligned}\frac{d([\text{L.}]\cdot V_{\text{cell}})}{dt} &= +V_{\text{cell}}\cdot(\text{1e+010}\cdot[\text{.OH}]\cdot[\text{LH}])\\ &\quad -V_{\text{cell}}\cdot(\text{3e+008}\cdot[\text{L.}]\cdot[\text{O2}])\\ &\quad +V_{\text{cell}}\cdot(50\cdot[\text{LO2.}]\cdot[\text{LH}])\\ &\quad +V_{\text{cell}}\cdot(\text{1e+006}\cdot[\text{NO2.}]\cdot[\text{LH}])\end{aligned}$$

$$\begin{aligned}\frac{d([\text{LH}]\cdot V_{\text{cell}})}{dt} &= -V_{\text{cell}}\cdot(\text{1e+010}\cdot[\text{.OH}]\cdot[\text{LH}])\\ &\quad -V_{\text{cell}}\cdot(50\cdot[\text{LO2.}]\cdot[\text{LH}])\\ &\quad -V_{\text{cell}}\cdot(\text{1e+006}\cdot[\text{NO2.}]\cdot[\text{LH}])\end{aligned}$$

$$\begin{aligned}\frac{d([\text{LO2.}]\cdot V_{\text{cell}})}{dt} &= +V_{\text{cell}}\cdot(\text{3e+008}\cdot[\text{L.}]\cdot[\text{O2}])\\ &\quad -V_{\text{cell}}\cdot(50\cdot[\text{LO2.}]\cdot[\text{LH}])\\ &\quad +V_{\text{cell}}\cdot(10000\cdot[\text{LOOH}]\cdot[\text{Fe2}])\\ &\quad -2\cdot V_{\text{cell}}\cdot(\text{1e+006}\cdot[\text{LO2.}]\cdot[\text{LO2.}])\end{aligned}$$

$$\begin{aligned}\frac{d([\text{LOOH}]\cdot V_{\text{cell}})}{dt} &= +V_{\text{cell}}\cdot(50\cdot[\text{LO2.}]\cdot[\text{LH}])\\ &\quad -V_{\text{cell}}\cdot(10000\cdot[\text{LOOH}]\cdot[\text{Fe2}])\end{aligned}$$

$$\frac{d([\text{Citrulline}]\cdot V_{\text{cell}})}{dt} = +V_{\text{cell}}\cdot\left(\frac{\text{3.5e-006}\cdot[\text{Arg}]\cdot[\text{NADPH}]}{\text{7e-007}\cdot[\text{Arg}]+\text{4.9e-006}\cdot[\text{NADPH}]+[\text{Arg}]\cdot[\text{NADPH}]+0.1\cdot\text{7e-007}}\right)$$

$$\begin{aligned}\frac{d([\text{NO.}]\cdot V_{\text{cell}})}{dt} &= +V_{\text{cell}}\cdot\left(\frac{\text{3.5e-006}\cdot[\text{Arg}]\cdot[\text{NADPH}]}{\text{7e-007}\cdot[\text{Arg}]+\text{4.9e-006}\cdot[\text{NADPH}]+[\text{Arg}]\cdot[\text{NADPH}]+0.1\cdot\text{7e-007}}\right)\\ &\quad -V_{\text{cell}}\cdot(\text{3.5e+009}\cdot[\text{NO.}]\cdot[\text{O}])\end{aligned}$$

$$\begin{aligned}\frac{d([\text{NO2.}]\cdot V_{\text{cell}})}{dt} &= +V_{\text{cell}}\cdot(\text{3.5e+009}\cdot[\text{NO.}]\cdot[\text{O}])\\ &\quad -V_{\text{cell}}\cdot(\text{1.4e+010}\cdot[\text{NO2.}]\cdot[\text{.OH}])\\ &\quad +V_{\text{cell}}\cdot(100000\cdot[\text{ONOOH}]\cdot[\text{H2O2}])\\ &\quad -V_{\text{cell}}\cdot(\text{3.4e+007}\cdot[\text{NO2.}]\cdot[\text{"O2.-"}])\\ &\quad -V_{\text{cell}}\cdot(\text{1e+006}\cdot[\text{NO2.}]\cdot[\text{LH}])\end{aligned}$$

$$\frac{d([\text{O}]\cdot V_{\text{cell}})}{dt} = -V_{\text{cell}}\cdot(\text{3.5e+009}\cdot[\text{NO.}]\cdot[\text{O}])$$

$$\begin{aligned}\frac{d([\text{ONOOH}]\cdot V_{\text{cell}})}{dt} &= +V_{\text{cell}}\cdot(\text{1.4e+010}\cdot[\text{NO2.}]\cdot[\text{.OH}])\\ &\quad -V_{\text{cell}}\cdot(100000\cdot[\text{ONOOH}]\cdot[\text{H2O2}])\end{aligned}$$

$$\begin{aligned}\frac{d([\text{"H+"}]\cdot V_{\text{cell}})}{dt} &= -V_{\text{cell}}\cdot(500000\cdot[\text{"O2.-"}]\cdot[\text{"O2.-"}]\cdot[\text{"H+"}])\\ &\quad +V_{\text{cell}}\cdot(100000\cdot[\text{ONOOH}]\cdot[\text{H2O2}])\\ &\quad +V_{\text{cell}}\cdot(\text{1e+006}\cdot[\text{NO2.}]\cdot[\text{LH}])\end{aligned}$$

$$\begin{aligned}\frac{d([\text{"ONOO-"}]\cdot V_{\text{cell}})}{dt} &= +V_{\text{cell}}\cdot(\text{3.4e+007}\cdot[\text{NO2.}]\cdot[\text{"O2.-"}])\\ &\quad -V_{\text{cell}}\cdot(900000\cdot[\text{TDPxr}]\cdot[\text{"ONOO-"}])\\ &\quad -V_{\text{cell}}\cdot(3500\cdot[\text{TXNr}]\cdot[\text{"ONOO-"}])\\ &\quad -V_{\text{cell}}\cdot(7200\cdot[\text{T[SH]2}]\cdot[\text{"ONOO-"}])\end{aligned}$$

$$\begin{aligned}\frac{d([\text{TDPxo}]\cdot V_{\text{cell}})}{dt} &= -V_{\text{cell}}\cdot(220000\cdot[\text{TXNr}]\cdot[\text{TDPxo}])\\ &\quad +V_{\text{cell}}\cdot(900000\cdot[\text{TDPxr}]\cdot[\text{"ONOO-"}])\end{aligned}$$

$$\begin{aligned}\frac{d([\text{TDPxr}]\cdot V_{\text{cell}})}{dt} &= +V_{\text{cell}}\cdot(220000\cdot[\text{TXNr}]\cdot[\text{TDPxo}])\\ &\quad -V_{\text{cell}}\cdot(900000\cdot[\text{TDPxr}]\cdot[\text{"ONOO-"}])\end{aligned}$$

$$\begin{aligned}\frac{d([\text{"NO2-"}]\cdot V_{\text{cell}})}{dt} &= +V_{\text{cell}}\cdot(900000\cdot[\text{TDPxr}]\cdot[\text{"ONOO-"}])\\ &\quad +V_{\text{cell}}\cdot(3500\cdot[\text{TXNr}]\cdot[\text{"ONOO-"}])\\ &\quad +V_{\text{cell}}\cdot(7200\cdot[\text{T[SH]2}]\cdot[\text{"ONOO-"}])\\ &\quad +V_{\text{cell}}\cdot(\text{1e+006}\cdot[\text{NO2.}]\cdot[\text{LH}])\end{aligned}$$

$$\begin{aligned}\frac{d([\text{"MGO.-"}]\cdot V_{\text{cell}})}{dt} &= +V_{\text{cell}}\cdot((0.0085\cdot[\text{Arg}]\cdot[\text{MGO}]-\text{4.4e-006}\cdot[\text{"Arg-MG"}]\cdot[\text{"MGO.-"}]))\\ &\quad +V_{\text{cell}}\cdot((0.0068\cdot[\text{Lys}]\cdot[\text{MGO}]-0.0005\cdot[\text{"Lys-MG"}]\cdot[\text{"MGO.-"}]))\\ &\quad -V_{\text{cell}}\cdot(\text{1.31e-010}\cdot[\text{"MGO.-"}]\cdot[\text{O2}])\end{aligned}$$

$$\frac{d([\text{"D-GLC"}]\cdot V_{\text{cell}})}{dt} = -V_{\text{cell}}\cdot\left(\frac{\text{4.3e-007}\cdot[\text{"D-GLC"}]}{\text{6.6e-008}+[\text{"D-GLC"}]}\right)$$

$$\begin{aligned}\frac{d([\text{GLC6P}]\cdot V_{\text{cell}})}{dt} &= +V_{\text{cell}}\cdot\left(\frac{\text{4.3e-007}\cdot[\text{"D-GLC"}]}{\text{6.6e-008}+[\text{"D-GLC"}]}\right)\\ &\quad -V_{\text{cell}}\cdot\left(\frac{\text{8.33e-006}\cdot[\text{GLC6P}]}{0.0001425+[\text{GLC6P}]}\right)\end{aligned}$$

$$\begin{aligned}\frac{d([\text{FR6P}]\cdot V_{\text{cell}})}{dt} &= +V_{\text{cell}}\cdot\left(\frac{\text{8.33e-006}\cdot[\text{GLC6P}]}{0.0001425+[\text{GLC6P}]}\right)\\ &\quad -V_{\text{cell}}\cdot\left(\frac{\text{5e-007}\cdot[\text{FR6P}]}{0.000157+[\text{FR6P}]}\right)\end{aligned}$$

$$\begin{aligned}\frac{d([\text{DHAP}]\cdot V_{\text{cell}})}{dt} &= +V_{\text{cell}}\cdot\left(\frac{\text{6.2e-007}\cdot[\text{FR16BP}]}{\text{4.9e-005}+[\text{FR16BP}]}\right)\\ &\quad -V_{\text{cell}}\cdot(0.00011\cdot[\text{DHAP}])\\ &\quad +V_{\text{cell}}\cdot\left(\frac{\text{1.588e-011}\cdot[\text{GA3P}]}{0.00026+[\text{GA3P}]}\right)\\ &\quad -V_{\text{cell}}\cdot\left(\frac{\text{2.87e-010}\cdot[\text{DHAP}]}{0.009+[\text{DHAP}]}\right)\end{aligned}$$

$$\begin{aligned}\frac{d([\text{FR16BP}]\cdot V_{\text{cell}})}{dt} &= +V_{\text{cell}}\cdot\left(\frac{\text{5e-007}\cdot[\text{FR6P}]}{0.000157+[\text{FR6P}]}\right)\\ &\quad -V_{\text{cell}}\cdot\left(\frac{\text{6.2e-007}\cdot[\text{FR16BP}]}{\text{4.9e-005}+[\text{FR16BP}]}\right)\end{aligned}$$

$$\begin{aligned}\frac{d([\text{GA3P}]\cdot V_{\text{cell}})}{dt} &= +V_{\text{cell}}\cdot\left(\frac{\text{6.2e-007}\cdot[\text{FR16BP}]}{\text{4.9e-005}+[\text{FR16BP}]}\right)\\ &\quad -V_{\text{cell}}\cdot\left(\frac{\text{2.55e-006}\cdot[\text{GA3P}]}{0.000165+[\text{GA3P}]}\right)\\ &\quad -V_{\text{cell}}\cdot(\text{1.1e-005}\cdot[\text{GA3P}])\\ &\quad -V_{\text{cell}}\cdot\left(\frac{\text{1.588e-011}\cdot[\text{GA3P}]}{0.00026+[\text{GA3P}]}\right)\\ &\quad +V_{\text{cell}}\cdot\left(\frac{\text{2.87e-010}\cdot[\text{DHAP}]}{0.009+[\text{DHAP}]}\right)\end{aligned}$$

$$\begin{aligned}\frac{d([\text{BPG}]\cdot V_{\text{cell}})}{dt} &= +V_{\text{cell}}\cdot\left(\frac{\text{2.55e-006}\cdot[\text{GA3P}]}{0.000165+[\text{GA3P}]}\right)\\ &\quad -V_{\text{cell}}\cdot\left(\frac{\text{1.65e-006}\cdot[\text{BPG}]}{0.00202+[\text{BPG}]}\right)\end{aligned}$$

$$\begin{aligned}\frac{d([\text{2PGA}]\cdot V_{\text{cell}})}{dt} &= +V_{\text{cell}}\cdot\left(\frac{\text{1.67e-009}\cdot[\text{3PGA}]}{0.00027+[\text{3PGA}]}\right)\\ &\quad -V_{\text{cell}}\cdot\left(\frac{\text{1.34e-006}\cdot[\text{2PGA}]}{\text{5.1e-005}+[\text{2PGA}]}\right)\end{aligned}$$

$$\begin{aligned}\frac{d([\text{3PGA}]\cdot V_{\text{cell}})}{dt} &= +V_{\text{cell}}\cdot\left(\frac{\text{1.65e-006}\cdot[\text{BPG}]}{0.00202+[\text{BPG}]}\right)\\ &\quad -V_{\text{cell}}\cdot\left(\frac{\text{1.67e-009}\cdot[\text{3PGA}]}{0.00027+[\text{3PGA}]}\right)\end{aligned}$$

$$\begin{aligned}\frac{d([\text{PEP}]\cdot V_{\text{cell}})}{dt} &= +V_{\text{cell}}\cdot\left(\frac{\text{1.34e-006}\cdot[\text{2PGA}]}{\text{5.1e-005}+[\text{2PGA}]}\right)\\ &\quad -V_{\text{cell}}\cdot\left(\frac{\text{8.2e-006}\cdot[\text{PEP}]^{1.56}}{0.00116^{1.56}+[\text{PEP}]^{1.56}}\right)\end{aligned}$$

$$\frac{d([\text{PYR}]\cdot V_{\text{cell}})}{dt} = +V_{\text{cell}}\cdot\left(\frac{\text{8.2e-006}\cdot[\text{PEP}]^{1.56}}{0.00116^{1.56}+[\text{PEP}]^{1.56}}\right)$$
