# Supplementary material for: Integrative Computational Framework for Understanding Metabolic Modulation in Leishmania
Source: Front Bioeng Biotechnol. 2019 Nov 19;7:336. doi: 10.3389/fbioe.2019.00336 (PMC6877600; doi:10.3389/fbioe.2019.00336)
Supplement: Supplementary file 10 [file Data_Sheet_2.PDF]

$$\frac{d([\text{Met}]\cdot V_{cell})}{dt} = -V_{cell}\cdot\left(\frac{3.4e-009\cdot[\text{Met}]^{2.3}}{0.00025^{2.3}+[\text{Met}]^{2.3}}\right)$$

$$\begin{aligned}\frac{d([\text{SAM}]\cdot V_{cell})}{dt} &= +V_{cell}\cdot\left(\frac{3.4e-009\cdot[\text{Met}]^{2.3}}{0.00025^{2.3}+[\text{Met}]^{2.3}}\right)\\ &-V_{cell}\cdot\left(\frac{5.2e-009\cdot[\text{SAM}]}{0.00038+[\text{SAM}]}\right)\end{aligned}$$

$$\begin{aligned}\frac{d([\text{dcSAM}]\cdot V_{cell})}{dt} &= +V_{cell}\cdot\left(\frac{5.2e-009\cdot[\text{SAM}]}{0.00038+[\text{SAM}]}\right)\\ &-V_{cell}\cdot\left(\frac{1.98e-013\cdot[\text{Put}]\cdot[\text{dcSAM}]}{9e-008\cdot[\text{Put}]+0.000205\cdot[\text{dcSAM}]+[\text{Put}]\cdot[\text{dcSAM}]+0.1\cdot0.000205}\right)\end{aligned}$$

$$\begin{aligned}\frac{d([\text{Arg}]\cdot V_{cell})}{dt} &= -V_{cell}\cdot((0.0085\cdot[\text{Arg}]\cdot[\text{MGO}]-4.4e-006\cdot[\text{"Arg-MG"}]\cdot[\text{"MGO.-"}]))\\ &-V_{cell}\cdot\left(\frac{0.0024\cdot[\text{Arg}]}{0.0215+[\text{Arg}]}\right)\\ &-V_{cell}\cdot\left(\frac{3.5e-006\cdot[\text{Arg}]\cdot[\text{NADPH}]}{7e-007\cdot[\text{Arg}]+4.9e-006\cdot[\text{NADPH}]+[\text{Arg}]\cdot[\text{NADPH}]+0.1\cdot7e-007}\right)\end{aligned}$$

$$\begin{aligned}\frac{d([\text{Orn}]\cdot V_{cell})}{dt} &= +V_{cell}\cdot\left(\frac{0.0024\cdot[\text{Arg}]}{0.0215+[\text{Arg}]}\right)\\ &-V_{cell}\cdot\left(\frac{0.025256\cdot[\text{Orn}]}{0.00042+[\text{Orn}]}\right)\end{aligned}$$

$$\begin{aligned}\frac{d([\text{Put}]\cdot V_{cell})}{dt} &= +V_{cell}\cdot\left(\frac{0.025256\cdot[\text{Orn}]}{0.00042+[\text{Orn}]}\right)\\ &-V_{cell}\cdot\left(\frac{1.98e-013\cdot[\text{Put}]\cdot[\text{dcSAM}]}{9e-008\cdot[\text{Put}]+0.000205\cdot[\text{dcSAM}]+[\text{Put}]\cdot[\text{dcSAM}]+0.1\cdot0.000205}\right)\end{aligned}$$

$$\begin{aligned}\frac{d([\text{Spd}]\cdot V_{cell})}{dt} &= +V_{cell}\cdot\left(\frac{1.98e-013\cdot[\text{Put}]\cdot[\text{dcSAM}]}{9e-008\cdot[\text{Put}]+0.000205\cdot[\text{dcSAM}]+[\text{Put}]\cdot[\text{dcSAM}]+0.1\cdot0.000205}\right)\\ &-V_{cell}\cdot\left(\frac{8e-007\cdot[\text{Spd}]\cdot[\text{GSH}]}{0.00094\cdot8.9e-005+[\text{Spd}]\cdot8.9e-005+[\text{GSH}]\cdot0.00094+[\text{Spd}]\cdot[\text{GSH}]}\right)\end{aligned}$$

$$\frac{d([\text{Cys}]\cdot V_{cell})}{dt} = -V_{cell}\cdot\left(\frac{1.8e-006\cdot[\text{Cys}]\cdot[\text{Glu}]}{0.0017\cdot[\text{Cys}]+0.0092\cdot[\text{Glu}]+[\text{Cys}]\cdot[\text{Glu}]+9.37e-005\cdot0.0017}\right)$$

$$\frac{d([\text{Glu}]\cdot V_{cell})}{dt} = -V_{cell}\cdot\left(\frac{1.8e-006\cdot[\text{Cys}]\cdot[\text{Glu}]}{0.0017\cdot[\text{Cys}]+0.0092\cdot[\text{Glu}]+[\text{Cys}]\cdot[\text{Glu}]+9.37e-005\cdot0.0017}\right)$$

$$\begin{aligned}\frac{d([\text{GluCys}]\cdot V_{cell})}{dt} &= +V_{cell}\cdot\left(\frac{1.8e-006\cdot[\text{Cys}]\cdot[\text{Glu}]}{0.0017\cdot[\text{Cys}]+0.0092\cdot[\text{Glu}]+[\text{Cys}]\cdot[\text{Glu}]+9.37e-005\cdot0.0017}\right)\\ &-V_{cell}\cdot\left(\frac{3.4e-008\cdot[\text{Gly}]\cdot[\text{GluCys}]}{0.0012\cdot[\text{Gly}]+4e-005\cdot[\text{GluCys}]+[\text{Gly}]\cdot[\text{GluCys}]+8e-005\cdot0.0012}\right)\end{aligned}$$

$$\begin{aligned}\frac{d([\text{GSH}]\cdot V_{cell})}{dt} &= +V_{cell}\cdot\left(\frac{3.4e-008\cdot[\text{Gly}]\cdot[\text{GluCys}]}{0.0012\cdot[\text{Gly}]+4e-005\cdot[\text{GluCys}]+[\text{Gly}]\cdot[\text{GluCys}]+8e-005\cdot0.0012}\right)\\ &-V_{cell}\cdot\left(\frac{8e-007\cdot[\text{Spd}]\cdot[\text{GSH}]}{0.00094\cdot8.9e-005+[\text{Spd}]\cdot8.9e-005+[\text{GSH}]\cdot0.00094+[\text{Spd}]\cdot[\text{GSH}]}\right)\\ &-V_{cell}\cdot\left(\frac{8e-007\cdot[\text{Gspd}]\cdot[\text{GSH}]}{4e-005\cdot8.9e-005+[\text{Gspd}]\cdot8.9e-005+[\text{GSH}]\cdot4e-005+[\text{Gspd}]\cdot[\text{GSH}]}\right)\end{aligned}$$

$$\frac{d([\text{Gly}]\cdot V_{cell})}{dt} = -V_{cell}\cdot\left(\frac{3.4e-008\cdot[\text{Gly}]\cdot[\text{GluCys}]}{0.0012\cdot[\text{Gly}]+4e-005\cdot[\text{GluCys}]+[\text{Gly}]\cdot[\text{GluCys}]+8e-005\cdot0.0012}\right)$$

$$\begin{aligned}\frac{d([\text{Gspd}]\cdot V_{cell})}{dt} &= +V_{cell}\cdot\left(\frac{8e-007\cdot[\text{Spd}]\cdot[\text{GSH}]}{0.00094\cdot8.9e-005+[\text{Spd}]\cdot8.9e-005+[\text{GSH}]\cdot0.00094+[\text{Spd}]\cdot[\text{GSH}]}\right)\\ &-V_{cell}\cdot\left(\frac{8e-007\cdot[\text{Gspd}]\cdot[\text{GSH}]}{4e-005\cdot8.9e-005+[\text{Gspd}]\cdot8.9e-005+[\text{GSH}]\cdot4e-005+[\text{Gspd}]\cdot[\text{GSH}]}\right)\end{aligned}$$

$$\begin{aligned}\frac{d([\text{MGO}]\cdot V_{cell})}{dt} &= +V_{cell}\cdot(0.016\cdot[\text{HTA}])\\ &-V_{cell}\cdot((0.0085\cdot[\text{Arg}]\cdot[\text{MGO}]-4.4e-006\cdot[\text{"Arg-MG"}]\cdot[\text{"MGO.-"}]))\\ &-V_{cell}\cdot((0.0068\cdot[\text{Lys}]\cdot[\text{MGO}]-0.0005\cdot[\text{"Lys-MG"}]\cdot[\text{"MGO.-"}]))\\ &+V_{cell}\cdot(1e+006\cdot[\text{LO2.}]\cdot[\text{LO2.}])\\ &+V_{cell}\cdot(1.31e-010\cdot[\text{"MGO.-"}]\cdot[\text{O2}])\\ &+V_{cell}\cdot(0.00011\cdot[\text{DHAP}])\\ &+V_{cell}\cdot(1.1e-005\cdot[\text{GA3P}])\\ &-V_{cell}\cdot(0.0056\cdot[\text{MGO}]\cdot[\text{T[SH]2}])\end{aligned}$$

$$\begin{aligned}\frac{d([\text{HTA}]\cdot V_{cell})}{dt} &= -V_{cell}\cdot(0.016\cdot[\text{HTA}])\\ &-V_{cell}\cdot\left(\frac{0.000159\cdot[\text{HTA}]}{3.2e-005+[\text{HTA}]+\frac{3.2e-005\cdot[\text{DNPGS}]}{0.000669}}\right)\\ &+V_{cell}\cdot(0.0056\cdot[\text{MGO}]\cdot[\text{T[SH]2}])\end{aligned}$$

$$\frac{d([\text{DL}]\cdot V_{cell})}{dt} = +V_{cell}\cdot\left(\frac{2.3e-009\cdot[\text{SDLTSH}]}{3.9e-005+[\text{SDLTSH}]}\right)$$

$$\begin{aligned}\frac{d([\text{NADP}]\cdot V_{cell})}{dt} &= +V_{cell}\cdot\left(\frac{3.33e-006\cdot[\text{TS2}]\cdot[\text{NADPH}]}{2e-005\cdot[\text{TS2}]+5e-005\cdot[\text{NADPH}]+[\text{TS2}]\cdot[\text{NADPH}]+0.1\cdot2e-005}\right)\\ &+V_{cell}\cdot\left(\frac{3.5e-006\cdot[\text{Arg}]\cdot[\text{NADPH}]}{7e-007\cdot[\text{Arg}]+4.9e-006\cdot[\text{NADPH}]+[\text{Arg}]\cdot[\text{NADPH}]+0.1\cdot7e-007}\right)\end{aligned}$$

$$\begin{aligned}\frac{d([\text{NADPH}]\cdot V_{cell})}{dt} &= -V_{cell}\cdot\left(\frac{3.33e-006\cdot[\text{TS2}]\cdot[\text{NADPH}]}{2e-005\cdot[\text{TS2}]+5e-005\cdot[\text{NADPH}]+[\text{TS2}]\cdot[\text{NADPH}]+0.1\cdot2e-005}\right)\\ &-V_{cell}\cdot\left(\frac{3.5e-006\cdot[\text{Arg}]\cdot[\text{NADPH}]}{7e-007\cdot[\text{Arg}]+4.9e-006\cdot[\text{NADPH}]+[\text{Arg}]\cdot[\text{NADPH}]+0.1\cdot7e-007}\right)\end{aligned}$$

$$\begin{aligned}\frac{d([\text{TS2}]\cdot V_{cell})}{dt} &= -V_{cell}\cdot\left(\frac{3.33e-006\cdot[\text{TS2}]\cdot[\text{NADPH}]}{2e-005\cdot[\text{TS2}]+5e-005\cdot[\text{NADPH}]+[\text{TS2}]\cdot[\text{NADPH}]+0.1\cdot2e-005}\right)\\ &+V_{cell}\cdot(200\cdot[\text{T[SH]2}]\cdot[\text{TXNo}])\\ &+V_{cell}\cdot(7200\cdot[\text{T[SH]2}]\cdot[\text{"ONOO-"}])\end{aligned}$$

$$\begin{aligned}\frac{d([\text{T[SH]2}]\cdot V_{cell})}{dt} &= +V_{cell}\cdot\left(\frac{3.33e-006\cdot[\text{TS2}]\cdot[\text{NADPH}]}{2e-005\cdot[\text{TS2}]+5e-005\cdot[\text{NADPH}]+[\text{TS2}]\cdot[\text{NADPH}]+0.1\cdot2e-005}\right)\\ &+V_{cell}\cdot(0.016\cdot[\text{HTA}])\\ &+V_{cell}\cdot\left(\frac{2.3e-009\cdot[\text{SDLTSH}]}{3.9e-005+[\text{SDLTSH}]}\right)\\ &-V_{cell}\cdot(200\cdot[\text{T[SH]2}]\cdot[\text{TXNo}])\\ &-V_{cell}\cdot(7200\cdot[\text{T[SH]2}]\cdot[\text{"ONOO-"}])\\ &+V_{cell}\cdot\left(\frac{8e-007\cdot[\text{Gspd}]\cdot[\text{GSH}]}{4e-005\cdot8.9e-005+[\text{Gspd}]\cdot8.9e-005+[\text{GSH}]\cdot4e-005+[\text{Gspd}]\cdot[\text{GSH}]}\right)\\ &-V_{cell}\cdot(0.0056\cdot[\text{MGO}]\cdot[\text{T[SH]2}])\end{aligned}$$

$$\frac{d([\text{CO2}]\cdot V_{cell})}{dt} = +V_{cell}\cdot\left(\frac{0.025256\cdot[\text{Orn}]}{0.00042+[\text{Orn}]}\right)$$

$$\begin{aligned}\frac{d([\text{SDLTSH}]\cdot V_{cell})}{dt} &= +V_{cell}\cdot\left(\frac{0.000159\cdot[\text{HTA}]}{3.2e-005+[\text{HTA}]+\frac{3.2e-005\cdot[\text{DNPGS}]}{0.000669}}\right)\\ &-V_{cell}\cdot\left(\frac{2.3e-009\cdot[\text{SDLTSH}]}{3.9e-005+[\text{SDLTSH}]}\right)\end{aligned}$$

$$\frac{d([\text{"Arg-MG"}]\cdot V_{cell})}{dt} = +V_{cell}\cdot((0.0085\cdot[\text{Arg}]\cdot[\text{MGO}]-4.4e-006\cdot[\text{"Arg-MG"}]\cdot[\text{"MGO.-"}]))$$

$$\frac{d([\text{Lys}]\cdot V_{cell})}{dt} = -V_{cell}\cdot((0.0068\cdot[\text{Lys}]\cdot[\text{MGO}]-0.0005\cdot[\text{"Lys-MG"}]\cdot[\text{"MGO.-"}]))$$

$$\frac{d([\text{"Lys-MG"}]\cdot V_{cell})}{dt} = +V_{cell}\cdot((0.0068\cdot[\text{Lys}]\cdot[\text{MGO}]-0.0005\cdot[\text{"Lys-MG"}]\cdot[\text{"MGO.-"}]))$$

$$\begin{aligned}\frac{d([\text{TXNo}]\cdot V_{cell})}{dt} &= -V_{cell}\cdot(200\cdot[\text{T[SH]2}]\cdot[\text{TXNo}])\\ &+V_{cell}\cdot\left(\frac{15.4\cdot[\text{TDPx}]\cdot[\text{H2O2}]\cdot[\text{TXNr}]}{2.2e-006\cdot[\text{H2O2}]+0.000193\cdot[\text{TXNr}]+[\text{H2O2}]\cdot[\text{TXNr}]}\right)\\ &+V_{cell}\cdot\left(\frac{8.8\cdot[\text{TryP}]\cdot[\text{H2O2}]\cdot[\text{TXNr}]}{4.9e-006\cdot[\text{H2O2}]+6.3e-006\cdot[\text{TXNr}]+[\text{H2O2}]\cdot[\text{TXNr}]}\right)\\ &+V_{cell}\cdot(220000\cdot[\text{TXNr}]\cdot[\text{TDPxo}])\\ &+V_{cell}\cdot(3500\cdot[\text{TXNr}]\cdot[\text{"ONOO-"}])\end{aligned}$$

$$\begin{aligned}\frac{d([\text{TXNr}]\cdot V_{cell})}{dt} &= +V_{cell}\cdot(200\cdot[\text{T[SH]2}]\cdot[\text{TXNo}])\\ &-V_{cell}\cdot\left(\frac{15.4\cdot[\text{TDPx}]\cdot[\text{H2O2}]\cdot[\text{TXNr}]}{2.2e-006\cdot[\text{H2O2}]+0.000193\cdot[\text{TXNr}]+[\text{H2O2}]\cdot[\text{TXNr}]}\right)\\ &-V_{cell}\cdot\left(\frac{8.8\cdot[\text{TryP}]\cdot[\text{H2O2}]\cdot[\text{TXNr}]}{4.9e-006\cdot[\text{H2O2}]+6.3e-006\cdot[\text{TXNr}]+[\text{H2O2}]\cdot[\text{TXNr}]}\right)\\ &-V_{cell}\cdot(220000\cdot[\text{TXNr}]\cdot[\text{TDPxo}])\\ &-V_{cell}\cdot(3500\cdot[\text{TXNr}]\cdot[\text{"ONOO-"}])\end{aligned}$$

$$\begin{aligned}\frac{d([\text{H2O}]\cdot V_{cell})}{dt} &= +V_{cell}\cdot\left(\frac{15.4\cdot[\text{TDPx}]\cdot[\text{H2O2}]\cdot[\text{TXNr}]}{2.2e-006\cdot[\text{H2O2}]+0.000193\cdot[\text{TXNr}]+[\text{H2O2}]\cdot[\text{TXNr}]}\right)\\ &+V_{cell}\cdot\left(\frac{8.8\cdot[\text{TryP}]\cdot[\text{H2O2}]\cdot[\text{TXNr}]}{4.9e-006\cdot[\text{H2O2}]+6.3e-006\cdot[\text{TXNr}]+[\text{H2O2}]\cdot[\text{TXNr}]}\right)\\ &+V_{cell}\cdot(1e+010\cdot[\text{.OH}]\cdot[\text{LH}])\\ &+V_{cell}\cdot(100000\cdot[\text{ONOOH}]\cdot[\text{H2O2}])\end{aligned}$$

$$\begin{aligned}\frac{d([\text{H2O2}]\cdot V_{cell})}{dt} &= -V_{cell}\cdot\left(\frac{15.4\cdot[\text{TDPx}]\cdot[\text{H2O2}]\cdot[\text{TXNr}]}{2.2e-006\cdot[\text{H2O2}]+0.000193\cdot[\text{TXNr}]+[\text{H2O2}]\cdot[\text{TXNr}]}\right)\\ &-V_{cell}\cdot\left(\frac{8.8\cdot[\text{TryP}]\cdot[\text{H2O2}]\cdot[\text{TXNr}]}{4.9e-006\cdot[\text{H2O2}]+6.3e-006\cdot[\text{TXNr}]+[\text{H2O2}]\cdot[\text{TXNr}]}\right)\\ &-V_{cell}\cdot(76\cdot[\text{H2O2}]\cdot[\text{Fe2}])\\ &+V_{cell}\cdot(500000\cdot[\text{"O2.-"}]\cdot[\text{"O2.-"}]\cdot[\text{"H+"}])\\ &+V_{cell}\cdot(1.6e+009\cdot[\text{"O2.-"}]\cdot[\text{"O2.-"}])\\ &-V_{cell}\cdot(100000\cdot[\text{ONOOH}]\cdot[\text{H2O2}])\end{aligned}$$

$$\begin{aligned}\frac{d([\text{.OH}]\cdot V_{cell})}{dt} &= +V_{cell}\cdot(76\cdot[\text{H2O2}]\cdot[\text{Fe2}])\\ &-V_{cell}\cdot(1e+010\cdot[\text{.OH}]\cdot[\text{LH}])\\ &-V_{cell}\cdot(1.4e+010\cdot[\text{NO2.}]\cdot[\text{.OH}])\end{aligned}$$

$$\begin{aligned}\frac{d([\text{Fe2}]\cdot V_{cell})}{dt} &= -V_{cell}\cdot(76\cdot[\text{H2O2}]\cdot[\text{Fe2}])\\ &-V_{cell}\cdot(10000\cdot[\text{LOOH}]\cdot[\text{Fe2}])\end{aligned}$$

$$\begin{aligned}\frac{d([\text{Fe3}]\cdot V_{cell})}{dt} &= +V_{cell}\cdot(76\cdot[\text{H2O2}]\cdot[\text{Fe2}])\\ &+V_{cell}\cdot(10000\cdot[\text{LOOH}]\cdot[\text{Fe2}])\end{aligned}$$

$$\begin{aligned}\frac{d([\text{"O2.-"}]\cdot V_{cell})}{dt} &= -2\cdot V_{cell}\cdot(500000\cdot[\text{"O2.-"}]\cdot[\text{"O2.-"}]\cdot[\text{"H+"}])\\ &-2\cdot V_{cell}\cdot(1.6e+009\cdot[\text{"O2.-"}]\cdot[\text{"O2.-"}])\\ &+V_{cell}\cdot(100000\cdot[\text{ONOOH}]\cdot[\text{H2O2}])\\ &-V_{cell}\cdot(3.4e+007\cdot[\text{NO2.}]\cdot[\text{"O2.-"}])\\ &+V_{cell}\cdot(1.31e-010\cdot[\text{"MGO.-"}]\cdot[\text{O2}])\end{aligned}$$

$$\begin{aligned}\frac{d([\text{O2}]\cdot V_{cell})}{dt} &= +V_{cell}\cdot(500000\cdot[\text{"O2.-"}]\cdot[\text{"O2.-"}]\cdot[\text{"H+"}])\\ &+V_{cell}\cdot(1.6e+009\cdot[\text{"O2.-"}]\cdot[\text{"O2.-"}])\\ &-V_{cell}\cdot(3e+008\cdot[\text{L.}]\cdot[\text{O2}])\\ &-V_{cell}\cdot(1.31e-010\cdot[\text{"MGO.-"}]\cdot[\text{O2}])\end{aligned}$$

$$\begin{aligned}\frac{d([\text{L.}]\cdot V_{cell})}{dt} &= +V_{cell}\cdot(1e+010\cdot[\text{.OH}]\cdot[\text{LH}])\\ &-V_{cell}\cdot(3e+008\cdot[\text{L.}]\cdot[\text{O2}])\\ &+V_{cell}\cdot(50\cdot[\text{LO2.}]\cdot[\text{LH}])\\ &+V_{cell}\cdot(1e+006\cdot[\text{NO2.}]\cdot[\text{LH}])\end{aligned}$$

$$\begin{aligned}\frac{d([\text{LH}]\cdot V_{cell})}{dt} &= -V_{cell}\cdot(1e+010\cdot[\text{.OH}]\cdot[\text{LH}])\\ &-V_{cell}\cdot(50\cdot[\text{LO2.}]\cdot[\text{LH}])\\ &-V_{cell}\cdot(1e+006\cdot[\text{NO2.}]\cdot[\text{LH}])\end{aligned}$$

$$\begin{aligned}\frac{d([\text{LO2.}]\cdot V_{cell})}{dt} &= +V_{cell}\cdot(3e+008\cdot[\text{L.}]\cdot[\text{O2}])\\ &-V_{cell}\cdot(50\cdot[\text{LO2.}]\cdot[\text{LH}])\\ &+V_{cell}\cdot(10000\cdot[\text{LOOH}]\cdot[\text{Fe2}])\\ &-2\cdot V_{cell}\cdot(1e+006\cdot[\text{LO2.}]\cdot[\text{LO2.}])\end{aligned}$$

$$\begin{aligned}\frac{d([\text{LOOH}]\cdot V_{cell})}{dt} &= +V_{cell}\cdot(50\cdot[\text{LO2.}]\cdot[\text{LH}])\\ &-V_{cell}\cdot(10000\cdot[\text{LOOH}]\cdot[\text{Fe2}])\end{aligned}$$

$$\frac{d([\text{Citrulline}]\cdot V_{cell})}{dt} = +V_{cell}\cdot\left(\frac{3.5e-006\cdot[\text{Arg}]\cdot[\text{NADPH}]}{7e-007\cdot[\text{Arg}]+4.9e-006\cdot[\text{NADPH}]+[\text{Arg}]\cdot[\text{NADPH}]+0.1\cdot7e-007}\right)$$

$$\begin{aligned}\frac{d([\text{NO.}]\cdot V_{cell})}{dt} &= +V_{cell}\cdot\left(\frac{3.5e-006\cdot[\text{Arg}]\cdot[\text{NADPH}]}{7e-007\cdot[\text{Arg}]+4.9e-006\cdot[\text{NADPH}]+[\text{Arg}]\cdot[\text{NADPH}]+0.1\cdot7e-007}\right)\\ &-V_{cell}\cdot(3.5e+009\cdot[\text{NO.}]\cdot[\text{O}])\end{aligned}$$

$$\begin{aligned}\frac{d([\text{NO2.}]\cdot V_{cell})}{dt} &= +V_{cell}\cdot(3.5e+009\cdot[\text{NO.}]\cdot[\text{O}])\\ &-V_{cell}\cdot(1.4e+010\cdot[\text{NO2.}]\cdot[\text{.OH}])\\ &+V_{cell}\cdot(100000\cdot[\text{ONOOH}]\cdot[\text{H2O2}])\\ &-V_{cell}\cdot(3.4e+007\cdot[\text{NO2.}]\cdot[\text{"O2.-"}])\\ &-V_{cell}\cdot(1e+006\cdot[\text{NO2.}]\cdot[\text{LH}])\end{aligned}$$

$$\frac{d([\text{O}]\cdot V_{cell})}{dt} = -V_{cell}\cdot(3.5e+009\cdot[\text{NO.}]\cdot[\text{O}])$$

$$\begin{aligned}\frac{d([\text{ONOOH}]\cdot V_{cell})}{dt} &= +V_{cell}\cdot(1.4e+010\cdot[\text{NO2.}]\cdot[\text{.OH}])\\ &-V_{cell}\cdot(100000\cdot[\text{ONOOH}]\cdot[\text{H2O2}])\end{aligned}$$

$$\begin{aligned}\frac{d([\text{"H+"}]\cdot V_{cell})}{dt} &= -V_{cell}\cdot(500000\cdot[\text{"O2.-"}]\cdot[\text{"O2.-"}]\cdot[\text{"H+"}])\\ &+V_{cell}\cdot(100000\cdot[\text{ONOOH}]\cdot[\text{H2O2}])\\ &+V_{cell}\cdot(1e+006\cdot[\text{NO2.}]\cdot[\text{LH}])\end{aligned}$$

$$\begin{aligned}\frac{d([\text{"ONOO-"}]\cdot V_{cell})}{dt} &= +V_{cell}\cdot(3.4e+007\cdot[\text{NO2.}]\cdot[\text{"O2.-"}])\\ &-V_{cell}\cdot(900000\cdot[\text{TDPxr}]\cdot[\text{"ONOO-"}])\\ &-V_{cell}\cdot(3500\cdot[\text{TXNr}]\cdot[\text{"ONOO-"}])\\ &-V_{cell}\cdot(7200\cdot[\text{T[SH]2}]\cdot[\text{"ONOO-"}])\end{aligned}$$

$$\begin{aligned}\frac{d([\text{TDPxo}]\cdot V_{cell})}{dt} &= -V_{cell}\cdot(220000\cdot[\text{TXNr}]\cdot[\text{TDPxo}])\\ &+V_{cell}\cdot(900000\cdot[\text{TDPxr}]\cdot[\text{"ONOO-"}])\end{aligned}$$

$$\begin{aligned}\frac{d([\text{TDPxr}]\cdot V_{cell})}{dt} &= +V_{cell}\cdot(220000\cdot[\text{TXNr}]\cdot[\text{TDPxo}])\\ &-V_{cell}\cdot(900000\cdot[\text{TDPxr}]\cdot[\text{"ONOO-"}])\end{aligned}$$

$$\begin{aligned}\frac{d([\text{"NO2-"}]\cdot V_{cell})}{dt} &= +V_{cell}\cdot(900000\cdot[\text{TDPxr}]\cdot[\text{"ONOO-"}])\\ &+V_{cell}\cdot(3500\cdot[\text{TXNr}]\cdot[\text{"ONOO-"}])\\ &+V_{cell}\cdot(7200\cdot[\text{T[SH]2}]\cdot[\text{"ONOO-"}])\\ &+V_{cell}\cdot(1e+006\cdot[\text{NO2.}]\cdot[\text{LH}])\end{aligned}$$

$$\begin{aligned}\frac{d([\text{"MGO.-"}]\cdot V_{cell})}{dt} &= +V_{cell}\cdot((0.0085\cdot[\text{Arg}]\cdot[\text{MGO}]-4.4e-006\cdot[\text{"Arg-MG"}]\cdot[\text{"MGO.-"}]))\\ &+V_{cell}\cdot((0.0068\cdot[\text{Lys}]\cdot[\text{MGO}]-0.0005\cdot[\text{"Lys-MG"}]\cdot[\text{"MGO.-"}]))\\ &-V_{cell}\cdot(1.31e-010\cdot[\text{"MGO.-"}]\cdot[\text{O2}])\end{aligned}$$

$$\frac{d([\text{"D-GLC"}]\cdot V_{cell})}{dt} = -V_{cell}\cdot\left(\frac{4.3e-007\cdot[\text{"D-GLC"}]}{6.6e-008+[\text{"D-GLC"}]}\right)$$

$$\begin{aligned}\frac{d([\text{GLC6P}]\cdot V_{cell})}{dt} &= +V_{cell}\cdot\left(\frac{4.3e-007\cdot[\text{"D-GLC"}]}{6.6e-008+[\text{"D-GLC"}]}\right)\\ &-V_{cell}\cdot\left(\frac{8.33e-006\cdot[\text{GLC6P}]}{0.0001425+[\text{GLC6P}]}\right)\end{aligned}$$

$$\begin{aligned}\frac{d([\text{FR6P}]\cdot V_{cell})}{dt} &= +V_{cell}\cdot\left(\frac{8.33e-006\cdot[\text{GLC6P}]}{0.0001425+[\text{GLC6P}]}\right)\\ &-V_{cell}\cdot\left(\frac{5e-007\cdot[\text{FR6P}]}{0.000157+[\text{FR6P}]}\right)\end{aligned}$$

$$\begin{aligned}\frac{d([\text{DHAP}]\cdot V_{cell})}{dt} &= +V_{cell}\cdot\left(\frac{6.2e-007\cdot[\text{FR16BP}]}{4.9e-005+[\text{FR16BP}]}\right)\\ &-V_{cell}\cdot(0.00011\cdot[\text{DHAP}])\\ &+V_{cell}\cdot\left(\frac{1.588e-011\cdot[\text{GA3P}]}{0.00026+[\text{GA3P}]}\right)\\ &-V_{cell}\cdot\left(\frac{2.87e-010\cdot[\text{DHAP}]}{0.009+[\text{DHAP}]}\right)\end{aligned}$$

$$\begin{aligned}\frac{d([\text{FR16BP}]\cdot V_{cell})}{dt} &= +V_{cell}\cdot\left(\frac{5e-007\cdot[\text{FR6P}]}{0.000157+[\text{FR6P}]}\right)\\ &-V_{cell}\cdot\left(\frac{6.2e-007\cdot[\text{FR16BP}]}{4.9e-005+[\text{FR16BP}]}\right)\end{aligned}$$

$$\begin{aligned}\frac{d([\text{GA3P}]\cdot V_{cell})}{dt} &= +V_{cell}\cdot\left(\frac{6.2e-007\cdot[\text{FR16BP}]}{4.9e-005+[\text{FR16BP}]}\right)\\ &-V_{cell}\cdot\left(\frac{2.55e-006\cdot[\text{GA3P}]}{0.000165+[\text{GA3P}]}\right)\\ &-V_{cell}\cdot(1.1e-005\cdot[\text{GA3P}])\\ &-V_{cell}\cdot\left(\frac{1.588e-011\cdot[\text{GA3P}]}{0.00026+[\text{GA3P}]}\right)\\ &+V_{cell}\cdot\left(\frac{2.87e-010\cdot[\text{DHAP}]}{0.009+[\text{DHAP}]}\right)\end{aligned}$$

$$\begin{aligned}\frac{d([\text{BPG}]\cdot V_{cell})}{dt} &= +V_{cell}\cdot\left(\frac{2.55e-006\cdot[\text{GA3P}]}{0.000165+[\text{GA3P}]}\right)\\ &-V_{cell}\cdot\left(\frac{1.65e-006\cdot[\text{BPG}]}{0.00202+[\text{BPG}]}\right)\end{aligned}$$

$$\begin{aligned}\frac{d([\text{2PGA}]\cdot V_{cell})}{dt} &= +V_{cell}\cdot\left(\frac{1.67e-009\cdot[\text{3PGA}]}{0.00027+[\text{3PGA}]}\right)\\ &-V_{cell}\cdot\left(\frac{1.34e-006\cdot[\text{2PGA}]}{5.1e-005+[\text{2PGA}]}\right)\end{aligned}$$

$$\begin{aligned}\frac{d([\text{3PGA}]\cdot V_{cell})}{dt} &= +V_{cell}\cdot\left(\frac{1.65e-006\cdot[\text{BPG}]}{0.00202+[\text{BPG}]}\right)\\ &-V_{cell}\cdot\left(\frac{1.67e-009\cdot[\text{3PGA}]}{0.00027+[\text{3PGA}]}\right)\end{aligned}$$

$$\begin{aligned}\frac{d([\text{PEP}]\cdot V_{cell})}{dt} &= +V_{cell}\cdot\left(\frac{1.34e-006\cdot[\text{2PGA}]}{5.1e-005+[\text{2PGA}]}\right)\\ &-V_{cell}\cdot\left(\frac{8.2e-006\cdot[\text{PEP}]^{1.56}}{0.00116^{1.56}+[\text{PEP}]^{1.56}}\right)\end{aligned}$$

$$\frac{d([\text{PYR}]\cdot V_{cell})}{dt} = +V_{cell}\cdot\left(\frac{8.2e-006\cdot[\text{PEP}]^{1.56}}{0.00116^{1.56}+[\text{PEP}]^{1.56}}\right)$$
